# Supplementary material for: Stereoselective diversification of α-amino acids enabled by N-heterocyclic carbene catalysis
Source: Nat Commun. 2025 Oct 9;16:8991. doi: 10.1038/s41467-025-64024-7 (PMC12511568; doi:10.1038/s41467-025-64024-7)
Supplement: Supplementary file 1 — Supplementary Information [file 41467_2025_64024_MOESM1_ESM.pdf]

Supplementary Information

Stereoselective Diversification of α-Amino Acids Enabled by N-Heterocyclic Carbene Catalysis

Table of Contents

General information 3

Preparation of N^{α}-protected-α-amino acids and peptides 4

Reaction optimization 26

Reconstruction of (U)AAs ester 31

Racemization-free peptide synthesis 68

Mechanism study 88

Determination of absolute configuration 89

Supplementary information for computational studies 108

Chiral separation results 113

NMR spectra 178

References 288

General information

All solvents were distilled according to general practice before use. All reagents were purchased and used without further purification unless specified otherwise. Solvents for flash column chromatography were technical grade and distilled before use. Analytical thin-layer chromatography (TLC) was performed using Huanghai silica gel plates with HSGF 254. The developed chromatogram was visualized by UV absorbance (254 nm) and appropriate stains. Flash column chromatography was performed using standard techniques with Qingdao Haiyang Chemical HG/T2354-92 silica gel (200-300 mesh) and the indicated solvent system. ^{1}H NMR, ^{13}C NMR and ^{19}F NMR data were recorded on Bruker 400 MHz (101 MHz for ^{13}C, 376 MHz for ^{19}F) nuclear resonance spectrometers unless otherwise specified, respectively. Chemical shifts (δ) in ppm are reported as quoted relative to the residual signals of chloroform (^{1}H 7.26 ppm and ^{13}C 77.16 ppm). Multiplicities are described as s (singlet), d (doublet), t (triplet), q (quartet), m (multiplet), and coupling constants (*J*) are reported in Hertz (Hz). 13C NMR and 19F NMR spectra were recorded with total proton decoupling. Chiral HPLC was recorded on a Thermo Fisher Dionex UltiMate 3000 fast performance liquid chromatography or Shimadzu LC-20A spectrometer using Daicel ChiralcelTM columns. HRMS (ESI) analysis was performed by the Analytical Instrumentation Center at Peking University, Shenzhen Graduate School and HRMS data were reported with ion mass/charge (m/z) ratios as values in atomic mass units. Optical rotations were measured in $CDCl_3$ on a Hanon P850 polarimeter with a sodium lamp of wavelength 589 nm, and reported as follows: $[\alpha]_D^T$ (c g/100 mL, solvent).

Preparation of N$^{\alpha}$-protected-α-amino acids and peptides

Commercially available N$^{\alpha}$-acyl-protected α-amino acids and peptides were purchased from Bidepharm and Energy Chemical.

N$^{\alpha}$-acyl-protected α-amino acid substrates were synthesized according to the literature [1,2]:

Procedure 1:

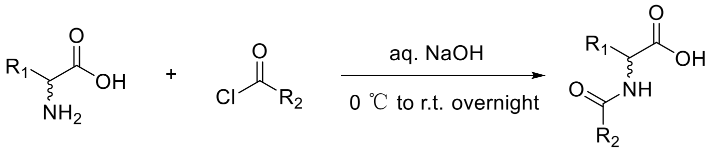

To a solution of α-amino acid (10 mmol) in water (20 mL) at room temperature, NaOH (30 mmol, 3 equiv) was added. The reaction mixture was stirred at room temperature until fully dissolved, and then the reaction solution was cooled to 0 °C, followed by the dropwise addition of acyl chloride (12 mmol, 1.2 equiv). The reaction mixture was allowed to warm to room temperature and stirred overnight. Upon completion, aqueous HCl solution (1 M) was added to the reaction mixture, and the pH was carefully adjusted to 2-4. Precipitation was collected via filtration, washed with water, dried over Na_2SO_4, filtered, and concentrated under reduced pressure. The resultant crude residue was purified by flash column chromatography using petroleum ether/EtOAc (1:2) as the eluent to yield **1**.

Procedure 2:

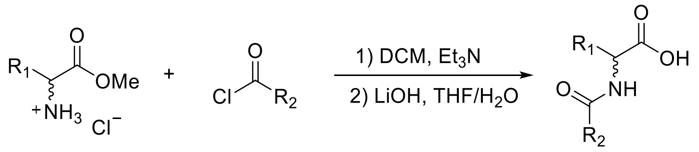

To a stirred solution of the α-amino acid ester (10 mmol) and Et_3N (15 mmol), the acyl chloride (10 mmol) was added dropwise, dissolved in DCM (10 mL) at 0 °C. The reaction mixture was stirred at room temperature for about 2-5 hours. After the reaction, the solvent was removed under reduced pressure, and the product was dissolved in 20 mL of water. Then, the mixture was extracted with ethyl acetate (EtOAc). The combined EtOAc layers were washed with brine, dried over Na_2SO_4, and filtered. The solvent was evaporated under reduced pressure. The residue was subjected to column chromatography using petroleum ether /EtOAc (4:1) to afford the desired Nα-acyl-

protected α-amino ester product.

To a solution of N^{α}-acyl-protected α-amino acid ester (5 mmol) in THF (6 mL) at 0 °C, aqueous LiOH (2 M, 3 mL, 1.2 equiv) was added. The reaction mixture was warmed to room temperature and stirred overnight. Upon completion, aqueous HCl solution (1 M) was added to the reaction mixture, and the pH was carefully adjusted to 2-4. The reaction was extracted with EtOAc (3 × 20 mL), and the combined organic layer was dried over Na_2SO_4, filtered, and concentrated under reduced pressure to yield **1**, which was used without further purification.

Procedure 3:

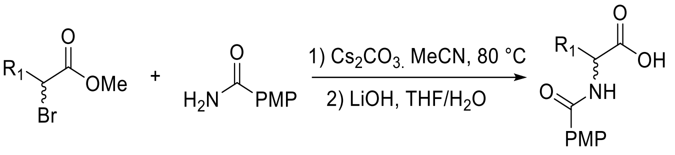

2-Bromopropionate (10 mmol) and 4-methoxybenzamide (15 mmol) were dissolved in MeCN (10 mL). The reaction mixture was stirred at 80 ºC overnight. After the reaction is complete, remove the solvent under reduced pressure. Then, the mixture was washed with water and extracted with ethyl acetate (EtOAc). The combined EtOAc layers were washed with brine, dried over Na_2SO_4, and filtered. The solvent was evaporated under reduced pressure, and the residue was subjected to column chromatography directly using a petroleum ether/EtOAc (4:1) mixture as the eluent, which afforded the desired α-amino ester. Then, an ester dissolved in THF (6 mL) at 0 °C was added to an aqueous solution of LiOH (2 M, 3 mL, 1.2 equiv). The reaction mixture was warmed to room temperature and stirred overnight. Upon completion, aqueous HCl solution (1 M) was added to the reaction mixture, and the pH was carefully adjusted to 2-4. The reaction was extracted with EtOAc (3 × 20 mL), and the combined organic layer was dried over Na_2SO_4, filtered, and concentrated under reduced pressure to yield **1**, which was used without further purification.

(4-methoxybenzoyl)-phenylalanine **1a**

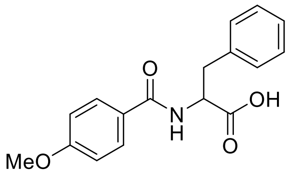

Following **General Procedure 1**, the desired product **1a** was obtained as a white solid

in a 90% yield (2.7 g).

TLC (100% EtOAc): R_f = 0.5

^{1}H NMR (400 MHz, Acetone-*d*$_6$) δ 7.9 – 7.8 (m, 2H), 7.4 – 7.3 (m, 2H), 7.3 (dd, *J* = 8.5, 6.7 Hz, 2H), 7.2 – 7.2 (m, 1H), 7.0 – 6.9 (m, 2H), 4.9 (dd, *J* = 9.1, 5.0 Hz, 1H), 3.9 (s, 3H), 3.3 (dd, *J* = 13.9, 5.0 Hz, 1H), 3.2 (dd, *J* = 13.9, 9.1 Hz, 1H).

^{13}C NMR (101 MHz, Acetone-*d*$_6$) δ 172.4, 162.3, 137.8, 129.2, 129.0, 128.3, 126.5, 113.5, 54.9, 53.9, 37.0.

HRMS (ESI-TOF) *m/z:* $[M+Na]^+$ Calcd for $C_{17}H_{77}NNaO_4^+$ 322.1050, found 322.1049

(4-methoxybenzoyl)tryptophan **1b**

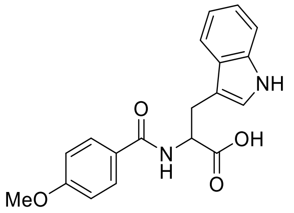

Following **General Procedure 2**, the desired product **1b** was obtained as a white solid with a 74% yield (2.4 g).

TLC (100% EtOAc): R_f = 0.3

^{1}H NMR (400 MHz, DMSO-*d*$_6$) δ 10.8 (d, *J* = 2.4 Hz, 1H), 8.5 (d, *J* = 7.8 Hz, 1H), 7.8 – 7.8 (m, 2H), 7.6 (d, *J* = 7.8 Hz, 1H), 7.3 (d, *J* = 8.0 Hz, 1H), 7.2 (d, *J* = 2.4 Hz, 1H), 7.1 (t, *J* = 7.5 Hz, 1H), 7.0 (dd, *J* = 8.1, 5.8 Hz, 3H), 4.6 (ddd, *J* = 9.7, 7.8, 4.6 Hz, 1H), 3.8 (s, 3H), 3.3 – 3.1 (m, 3H).

^{13}C NMR (101 MHz, DMSO-*d*$_6$) δ 174.2, 166.3, 162.1, 136.6, 129.7, 127.6, 126.6, 124.0, 121.4, 118.8, 118.6, 113.9, 111.9, 111.0, 55.8, 54.1, 27.1.

HRMS (ESI-TOF) *m/z:* $[M+Na]^+$ Calcd for $C_{19}H_{18}N_2NaO_4^+$ 361.1159, found 361.1160

(4-methoxybenzoyl)-alanine **1c**

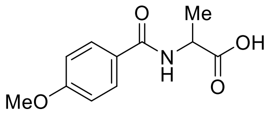

Following **General Procedure 1**, the desired product **1c** was obtained as a white solid with a 71% yield (1.6 g).

TLC (100% EtOAc): R_f = 0.5

^{1}H NMR (400 MHz, DMSO-*d*$_6$) δ 8.5 (d, *J* = 7.2 Hz, 1H), 7.9 (d, *J* = 8.9 Hz, 2H), 7.0 (d, *J* = 8.8 Hz, 2H), 4.4 (p, *J* = 7.3 Hz, 1H), 3.8 (s, 3H), 1.4 (d, *J* = 7.3 Hz, 3H).

^{13}C NMR (101 MHz, DMSO-d_6) δ 174.8, 166.1, 162.1, 131.8, 129.7, 113.9, 55.8, 48.5, 17.4.

HRMS (ESI-TOF) *m/z:* $[M+Na]^+$ Calcd for $C_{11}H_{13}NNaO_4^+$ 246.0737, found 246.0737

(4-methoxybenzoyl)-methionine **1d**

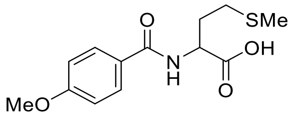

Following **General Procedure 2**, the desired product **1d** was obtained as a white solid with an 82% yield (2.3 g).

TLC (100% EtOAc): R_f = 0.5

^{1}H NMR (400 MHz, Acetone-d_6) δ 7.9 (d, *J* = 8.4 Hz, 2H), 7.8 (d, *J* = 8.1 Hz, 1H), 7.0 (d, *J* = 8.5 Hz, 2H), 4.8 (td, *J* = 8.5, 4.6 Hz, 1H), 3.9 (s, 3H), 2.7 (td, *J* = 8.0, 7.4, 4.9 Hz, 2H), 2.2 (dp, *J* = 13.2, 4.7, 3.9 Hz, 1H), 2.1 (s, 4H).

^{13}C NMR (101 MHz, Acetone-d_6) δ 172.8, 166.5, 162.4, 129.2, 126.6, 113.5, 54.9, 51.8, 31.1, 30.3, 14.3.

HRMS (ESI-TOF) *m/z:* $[M+H]^+$ Calcd for $C_{13}H_{18}NSO_4^+$ 284.0951, found 284.0947

2-(4-methoxybenzamido)-3-(4-methoxyphenyl)propanoic acid **1e**

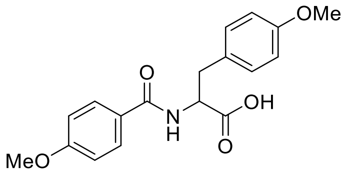

Following **General Procedure 2**, the desired product **1e** was obtained as a white solid with a 91% yield (3.0 g).

TLC (100% EtOAc): R_f = 0.5

^{1}H NMR (400 MHz, DMSO-d_6) δ 7.8 (d, *J* = 8.8 Hz, 2H), 7.2 (d, *J* = 8.6 Hz, 2H), 7.0 (d, *J* = 8.9 Hz, 2H), 6.8 (d, *J* = 8.6 Hz, 2H), 4.9 (td, *J* = 8.3, 3.8 Hz, 1H), 3.8 (s, 3H), 3.7 (s, 3H), 3.3 (dd, *J* = 14.0, 5.0 Hz, 1H), 3.1 (dd, *J* = 14.0, 8.8 Hz, 1H), 2.1 – 2.0 (m, 3H).

^{13}C NMR (101 MHz, DMSO-d_6) δ 172.6, 166.2, 162.3, 158.6, 130.2, 129.5, 129.1, 126.6, 126.6, 113.7, 113.5, 54.9, 54.5, 36.2.

HRMS (ESI-TOF) *m/z:* $[M+Na]^+$ Calcd for $C_{18}H_{19}NNaO_5^+$ 352.1161, found 352.1162.

3-(4-fluorophenyl)-2-(4-methoxybenzamido)propanoic acid **1f**

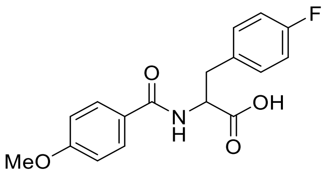

Following **General Procedure 1**, the desired product **1f** was obtained as a white solid with a 77% yield (2.4 g).

TLC (100% EtOAc): R_f = 0.4

^{1}H NMR (400 MHz, DMSO-*d*$_6$) δ 12.7 (s, 1H), 8.5 (d, *J* = 8.2 Hz, 1H), 7.8 (d, *J* = 8.4 Hz, 2H), 7.3 (dd, *J* = 8.2, 5.4 Hz, 2H), 7.2 – 6.7 (m, 4H), 4.7 – 4.5 (m, 1H), 3.8 (s, 3H), 3.3 – 2.9 (m, 2H).

^{13}C NMR (101 MHz, DMSO-*d*$_6$) δ 173.7, 166.3, 162.2, 161.4 (d, *J* = 241.7 Hz), 134.9 (d, *J* = 3.0 Hz), 131.4 (d, *J* = 7.9 Hz), 129.7, 126.5, 115.3 (d, *J* = 21.1 Hz), 113.9, 55.8, 54.6, 35.9.

^{19}F NMR (376 MHz, DMSO-*d*$_6$) δ -116.8.

HRMS (ESI-TOF) *m/z:* $[M+Na]^+$ Calcd for $C_{17}H_{16}FNNaO_4^+$ 340.0961, found 340.0960.

3-(4-chlorophenyl)-2-(4-methoxybenzamido)propanoic acid **1g**

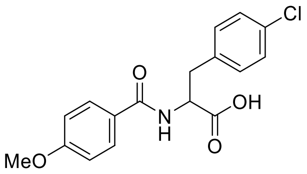

Following **General Procedure 1**, the desired product **1g** was obtained as a white solid with a 93% yield (3.1 g).

TLC (100% EtOAc): R_f = 0.4

^{1}H NMR (400 MHz, DMSO-*d*$_6$) δ 12.7 (s, 1H), 8.6 (d, *J* = 8.2 Hz, 1H), 7.8 (d, *J* = 8.4 Hz, 2H), 7.6 – 7.2 (m, 4H), 7.0 (d, *J* = 8.5 Hz, 2H), 4.8 – 4.5 (m, 1H), 3.8 (s, 3H), 3.3 – 2.9 (m, 2H).

^{13}C NMR (101 MHz, DMSO-*d*$_6$) δ 173.6, 166.3, 162.2, 137.8, 131.4, 129.7, 128.6, 126.5, 113.9, 55.8, 54.4, 36.1.

HRMS (ESI-TOF) *m/z:* $[M+H]^+$ Calcd for $C_{17}H_{17}ClNO_4^+$ 334.0841, found 334.0840.

3-(2-chlorophenyl)-2-(4-methoxybenzamido)propanoic acid **1h**

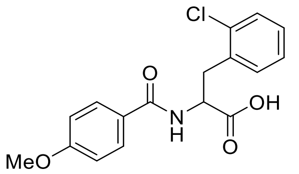

Following **General Procedure 2**, the desired product **1h** was obtained as a white solid with an 80% yield (2.7 g).

TLC (100% EtOAc): R_f = 0.5

^{1}H NMR (400 MHz, Acetone-*d*$_6$) δ 8.1 – 7.7 (m, 3H), 7.6 – 7.3 (m, 2H), 7.2 (d, *J* = 3.5 Hz, 2H), 6.9 (d, *J* = 8.3 Hz, 2H), 5.0 (d, *J* = 4.8 Hz, 1H), 3.8 (s, 3H), 3.5 (dd, *J* = 14.1, 4.8 Hz, 1H), 3.2 (dd, *J* = 14.1, 10.1 Hz, 1H).

^{13}C NMR (101 MHz, Acetone-*d*$_6$) δ 172.6, 166.4, 162.3, 135.6, 134.0, 131.7, 129.4, 129.1, 128.4, 126.9, 126.5, 113.5, 54.9, 52.3, 35.0.

HRMS (ESI-TOF) *m/z:* $[M+Na]^+$ Calcd for $C_{17}H_{17}ClNNaO_4^+$ 356.0660, found 356.0658.

3-(4-bromophenyl)-2-(4-methoxybenzamido)propanoic acid **1i**

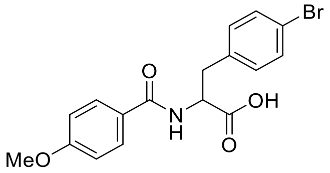

Following **General Procedure 1**, the desired product **1i** was obtained as a white solid with a 94% yield (3.5 g).

TLC (100% EtOAc): R_f = 0.6

^{1}H NMR (400 MHz, DMSO-*d*$_6$) δ 12.7 (s, 1H), 8.5 (d, *J* = 8.2 Hz, 1H), 7.8 (d, *J* = 8.8 Hz, 2H), 7.4 (d, *J* = 8.4 Hz, 2H), 7.3 (d, *J* = 8.4 Hz, 2H), 7.0 (d, *J* = 8.8 Hz, 2H), 4.6 (ddd, *J* = 10.6, 8.1, 4.4 Hz, 1H), 3.8 (s, 3H), 3.2 – 2.9 (m, 2H).

^{13}C NMR (101 MHz, DMSO-*d*$_6$) δ 173.6, 166.3, 162.2, 138.2, 131.8, 131.5, 129.7, 126.5, 120.0, 113.9, 55.8, 54.3, 36.1.

HRMS (ESI-TOF) *m/z:* $[M+Na]^+$ Calcd for $C_{17}H_{16}BrNNaO_4^+$ 400.0155, found 400.0155.

2-(4-methoxybenzamido)-3-(naphthalen-1-yl)propanoic acid **1j**

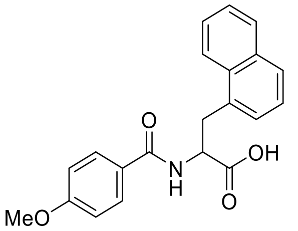

Following **General Procedure 1**, the desired product **1j** was obtained as a white solid with a 97% yield (3.4 g).

TLC (100% EtOAc): R_f = 0.5

^{1}H NMR (400 MHz, DMSO-*d*$_6$) δ 8.7 (d, *J* = 8.2 Hz, 1H), 8.2 (d, *J* = 8.4 Hz, 1H), 8.0 – 7.9 (m, 1H), 7.8 (dd, *J* = 8.6, 6.6 Hz, 3H), 7.6 (ddd, *J* = 8.5, 6.8, 1.4 Hz, 1H), 7.6 – 7.5 (m, 2H), 7.4 – 7.4 (m, 1H), 7.0 (d, *J* = 8.8 Hz, 2H), 4.8 (ddd, *J* = 10.5, 8.1, 4.0 Hz, 1H), 3.8 (s, 3H), 3.8 (dd, *J* = 14.1, 4.1 Hz, 1H), 3.5 (dd, *J* = 14.2, 10.6 Hz, 2H).

^{13}C NMR (101 MHz, DMSO-*d*$_6$) δ 173.9, 166.4, 162.2, 134.6, 133.9, 131.9, 129.7, 129.2, 127.8, 127.6, 126.8, 126.5, 126.1, 125.8, 123.7, 113.9, 55.8, 53.8, 34.2.

HRMS (ESI-TOF) *m/z:* $[M+Na]^+$ Calcd for $C_{21}H_{19}NNaO_4^+$ 372.1206, found 372.1204.

2-(4-methoxybenzamido)-3-(thiophen-2-yl)propanoic acid **1k**

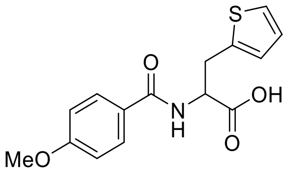

Following **General Procedure 2**, the desired product **1k** was obtained as a white solid with a 90% yield (2.7 g).

TLC (100% EtOAc): R_f = 0.3

^{1}H NMR (400 MHz, Chloroform-*d*) δ 9.2 (s, 1H), 7.8 – 7.6 (m, 2H), 7.1 (dd, *J* = 5.2, 1.2 Hz, 1H), 7.0 – 6.8 (m, 5H), 5.1 (dt, *J* = 7.4, 5.0 Hz, 1H), 3.8 (s, 3H), 3.6 – 3.4 (m, 2H).

^{13}C NMR (101 MHz, Chloroform-*d*) δ 174.4, 167.5, 162.7, 137.2, 129.2, 127.1, 127.0, 125.6, 125.0, 113.9, 55.4, 53.6, 31.7.

HRMS (ESI-TOF) *m/z:* $[M+Na]^+$ Calcd for $C_{15}H_{15}NNaO_4S^+$ 328.0614, found 328.0610.

2-(2-chlorophenyl)-2-(4-methoxybenzamido)acetic acid **1l**

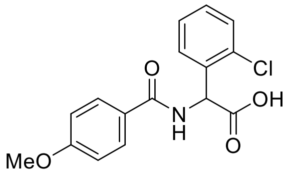

Following **General Procedure 2**, the desired product **1l** was obtained as a white solid with a 61% yield (1.9 g).

TLC (100% EtOAc): R_f = 0.6

^{1}H NMR (400 MHz, DMSO-*d*$_6$) δ 9.0 (d, *J* = 7.7 Hz, 1H), 7.9 (d, *J* = 8.8 Hz, 2H), 7.6 – 7.5 (m, 1H), 7.5 – 7.5 (m, 1H), 7.4 – 7.3 (m, 2H), 7.0 (d, *J* = 8.8 Hz, 2H), 6.0 (d, *J* = 7.7 Hz, 1H), 3.8 (s, 3H).

^{13}C NMR (101 MHz, DMSO-*d*$_6$) δ 171.7, 166.1, 162.3, 135.8, 133.8, 131.8, 130.0, 129.9, 129.8, 127.8, 126.2, 113.9, 55.8, 54.1.

HRMS (ESI-TOF) *m/z:* $[M+Na]^+$ Calcd for $C_{16}H_{14}ClNNaO_4^+$ 342.0504, found 342.0503.

2-(4-methoxybenzamido)-4-phenylbutanoic acid **1m**

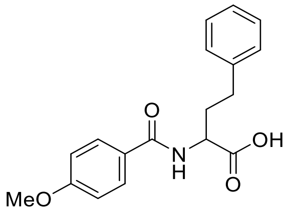

Following **General Procedure 2**, the desired product **1m** was obtained as a white solid with an 86% yield (2.7 g).

TLC (100% EtOAc): R_f = 0.5

^{1}H NMR (400 MHz, DMSO-*d*$_6$) δ 8.6 (d, *J* = 7.7 Hz, 1H), 7.9 (dd, *J* = 8.7, 7.1 Hz, 2H), 7.3 – 7.2 (m, 5H), 7.0 (d, *J* = 8.5 Hz, 2H), 4.3 (q, *J* = 7.4 Hz, 1H), 3.8 (s, 3H), 2.9 – 2.6 (m, 3H), 2.1 (q, *J* = 7.6 Hz, 2H).

^{13}C NMR (101 MHz, DMSO-*d*$_6$) δ 174.3, 166.6, 162.2, 141.6, 131.8, 129.9, 128.9, 128.8, 126.7, 126.4, 114.3, 113.9, 55.8, 52.6, 32.9, 32.3.

HRMS (ESI-TOF) *m/z:* $[M+Na]^+$ Calcd for $C_{18}H_{19}NNaO_4^+$ 336.1206, found 336.1208.

3-cyclohexyl-2-(4-methoxybenzamido)propanoic acid **1n**

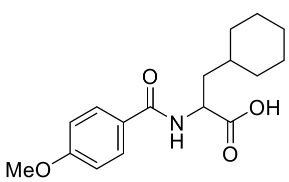

Following **General Procedure 2**, the desired product **1n** was obtained as a white solid with a 73% yield (2.2 g).

TLC (100% EtOAc): R_f = 0.6

^{1}H NMR (400 MHz, DMOS-*d*$_6$) δ 13.6 (s, 1H), 9.5 (d, *J* = 7.9 Hz, 1H), 9.0 (dd, *J* = 8.6, 5.2 Hz, 2H), 8.2 – 8.0 (m, 2H), 5.6 – 5.5 (m, 1H), 4.9 (d, *J* = 3.6 Hz, 3H), 2.9 – 2.6 (m, 6H), 2.6 – 2.4 (m, 1H), 2.4 – 2.1 (m, 4H), 2.1 – 1.9 (m, 2H).

^{13}C NMR (101 MHz, DMOS-*d*$_6$) δ 176.0, 167.4, 163.2, 132.8, 130.8, 127.7, 114.9, 56.8, 51.6, 39.5, 35.3, 34.7, 33.0, 27.6, 27.3, 27.1.

HRMS (ESI-TOF) *m/z:* $[M+Na]^+$ Calcd for $C_{17}H_{23}NNaO_4^+$ 328.1519, found 328.1521.

2-(4-methoxybenzamido)pentanoic acid **1o**

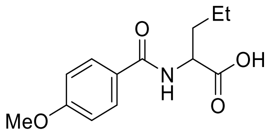

Following **General Procedure 3**, the desired product **1o** was afforded as a white solid with a 90% yield (2.2 g).

TLC (100% EtOAc): R_f = 0.5

^{1}H NMR (400 MHz, Acetone-*d*$_6$) δ 8.0 – 8.0 (m, 2H), 7.9 (d, *J* = 7.8 Hz, 1H), 7.1 – 7.0 (m, 2H), 4.8 – 4.7 (m, 1H), 3.9 (s, 3H), 2.0 – 1.8 (m, 2H), 1.6 – 1.5 (m, 2H), 1.0 (t, *J* = 7.4 Hz, 3H).

^{13}C NMR (101 MHz, Acetone-*d*$_6$) δ 173.7, 166.9, 162.4, 129.3, 126.4, 113.5, 55.0, 52.6, 33.6, 19.1, 13.2.

HRMS (ESI-TOF) *m/z:* $[M+Na]^+$ Calcd for $C_{13}H_{17}NNaO_4^+$ 274.1050, found 274.1048.

2-(4-methoxybenzamido)hexanoic acid **1p**

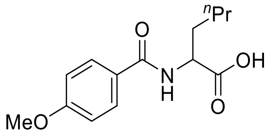

Following **General Procedure 2**, the desired product **1p** was obtained as a white solid with a yield of 98% (3.6 g).

TLC (100% EtOAc): R_f = 0.5

^{1}H NMR (400 MHz, Acetone-*d*$_6$) δ 8.0 – 7.9 (m, 2H), 7.0 – 7.0 (m, 2H), 4.7 – 4.6 (m, 1H), 3.9 (s, 3H), 2.0 – 1.9 (m, 1H), 1.9 – 1.8 (m, 1H), 1.5 – 1.3 (m, 4H), 0.9 (t, *J* = 7.2 Hz, 3H).

^{13}C NMR (101 MHz, Acetone-d_6) δ 173.3, 166.3, 162.3, 131.6, 129.1, 113.4, 54.9, 52.6, 31.3, 28.0, 22.1, 13.3.

HRMS (ESI-TOF) *m/z:* $[M+Na]^+$ Calcd for $C_{14}H_{19}NNaO_4^+$ 288.1206, found 288.1203.

2-(4-methoxybenzamido)pent-4-enoic acid **1q**

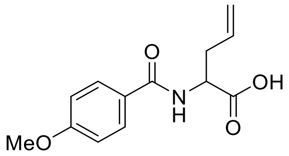

Following **General Procedure 2**, the desired product **1q** was obtained as a white solid with a 52% yield (1.3 g).

TLC (100% EtOAc): R_f = 0.4

^{1}H NMR (400 MHz, Acetone-d_6) δ 7.9 (d, J = 8.8 Hz, 2H), 7.0 (d, J = 8.9 Hz, 2H), 5.9 (ddt, J = 17.1, 10.2, 7.0 Hz, 1H), 5.2 – 5.0 (m, 2H), 4.7 (td, J = 8.2, 5.1 Hz, 1H), 3.9 (s, 3H), 2.8 – 2.7 (m, 1H), 2.7 – 2.6 (m, 1H).

^{13}C NMR (101 MHz, Acetone-d_6) δ 172.4, 166.1, 162.4, 134.1, 131.6, 129.1, 117.3, 113.5, 54.9, 52.3, 35.8.

HRMS (ESI-TOF) *m/z:* $[M+Na]^+$ Calcd for $C_{13}H_{15}NNaO_4^+$ 272.0893, found 272.0893.

2-(4-methoxybenzamido)pent-4-ynoic acid **1r**

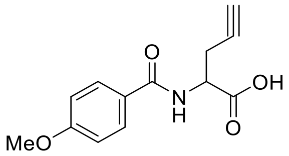

Following **General Procedure 2**, the desired product **1r** was obtained as a white solid with a 46% yield (1.1 g).

TLC (100% EtOAc): R_f = 0.4

^{1}H NMR (400 MHz, Acetone-d_6) δ 8.0 (d, J = 8.8 Hz, 2H), 7.1 (d, J = 8.8 Hz, 2H), 4.9 (td, J = 7.4, 5.3 Hz, 1H), 3.9 (s, 3H), 3.1 – 2.9 (m, 2H).

^{13}C NMR (101 MHz, Acetone-d_6) δ 171.3, 162.5, 131.6, 129.2, 113.6, 79.7, 71.4, 55.0, 54.9, 51.6, 21.3.

HRMS (ESI-TOF) *m/z:* $[M+Na]^+$ Calcd for $C_{13}H_{13}NNaO_4^+$ 270.0737, found 270.0737.

2-cyclohexyl-2-(4-methoxybenzamido)acetic acid **1s**

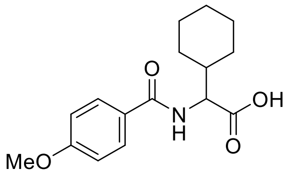

Following **General Procedure 2**, the desired product **1s** was obtained as a white solid with a 41% yield (1.2 g).

TLC (100% EtOAc): R_f = 0.4

^{1}H NMR (400 MHz, DMSO-*d*$_6$) δ 12.5 (s, 1H), 8.2 (d, *J* = 8.1 Hz, 1H), 8.0 – 7.8 (m, 2H), 7.2 – 6.8 (m, 2H), 4.3 (t, *J* = 7.7 Hz, 1H), 3.8 (s, 3H), 1.9 – 1.5 (m, 6H), 1.5 – 1.1 (m, 5H).

^{13}C NMR (101 MHz, DMSO-*d*$_6$) δ 173.7, 166.6, 162.1, 129.9, 126.8, 113.8, 58.2, 55.8, 29.8, 29.3, 26.2, 26.0.

HRMS (ESI-TOF) *m/z:* [M+Na]$^+$ Calcd for $C_{16}H_{21}NNaO_4^+$ 314.1363, found 314.1363.

(furan-2-carbonyl)phenylalanine **1ac**

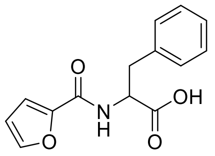

Following **General Procedure 2**, the desired product **1ac** was obtained as a white solid with an 81% yield (2.1 g).

TLC (100% EtOAc): R_f = 0.24

^{1}H NMR (400 MHz, DMSO-*d*$_6$) δ 8.5 (d, *J* = 8.3 Hz, 1H), 7.3 – 7.2 (m, 5H), 6.6 (ddd, *J* = 18.5, 3.5, 1.8 Hz, 2H), 4.6 (ddd, *J* = 10.3, 8.3, 4.5 Hz, 1H), 3.2 – 3.2 (m, 1H), 3.1 (dd, *J* = 13.9, 10.3 Hz, 1H).

^{13}C NMR (101 MHz, DMSO-*d*$_6$) δ 173.4, 158.1, 147.8, 145.6, 138.5, 129.5, 128.7, 126.8, 118.1, 112.5, 53.8, 36.6**.**

HRMS (ESI-TOF) *m/z:* [M+Na]$^+$ Calcd for $C_{14}H_{13}NNaO_4^+$ 282.0737, found 282.0733.

cinnamoylphenylalanine **1ad**

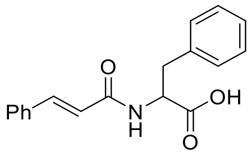

Following **General Procedure 2**, the desired product **1ad** was obtained as a white solid with a 42% yield (1.2 g).

TLC (100% EtOAc): R_f = 5

^{1}H NMR (400 MHz, DMSO-*d*$_6$) δ 12.7 (s, 1H), 8.4 (d, *J* = 8.1 Hz, 1H), 7.8 – 7.0 (m, 10H), 6.7 (d, *J* = 15.8 Hz, 1H), 4.6 (q, *J* = 7.6 Hz, 1H), 3.2 – 2.8 (m, 2H).

^{13}C NMR (101 MHz, DMSO-*d*$_6$) δ 173.5, 165.4, 139.7, 138.1, 135.2, 130.0, 129.5, 129.4, 128.7, 128.0, 126.9, 122.1, 54.1, 37.3.

HRMS (ESI-TOF) *m/z:* $[M+Na]^+$ Calcd for $C_{18}H_{17}NNaO_3^+$ 318.1101, found 318.1100.

(cyclohexanecarbonyl)phenylalanine **1ae**

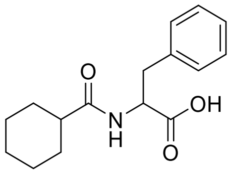

Following **General Procedure 2**, the desired product **1ae** was obtained as a colorless oil with a 76% yield (2.1 g).

TLC (100% EtOAc): R_f = 0.6

^{1}H NMR (400 MHz, DMSO-*d*$_6$) δ 8.0 (d, *J* = 8.4 Hz, 1H), 7.2 (dq, *J* = 13.9, 7.1 Hz, 5H), 4.4 (d, *J* = 6.9 Hz, 1H), 3.1 – 2.8 (m, 2H), 2.1 (s, 1H), 1.7 – 1.5 (m, 5H), 1.2 (dq, *J* = 32.9, 11.6 Hz, 5H).

^{13}C NMR (101 MHz, DMSO-*d*$_6$) δ 175.6, 173.7, 138.3, 129.6, 128.5, 126.8, 53.5, 44.0, 37.1, 29.5, 29.4, 25.9, 25.7, 25.6.

HRMS (ESI-TOF) *m/z:* $[M+Na]^+$ Calcd for $C_{16}H_{21}NNaO_3^+$ 298.1414, found 298.1411.

(diphenylcarbamoyl)phenylalanine **1af**

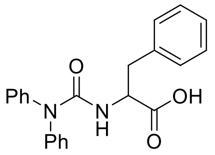

Following **General Procedure 2**, the desired product **1af** was obtained as a blue solid with a 73% yield (2.6 g).

TLC (100% EtOAc): R_f = 0.4

^{1}H NMR (400 MHz, DMSO-*d*$_6$) δ 7.4 – 7.2 (m, 9H), 7.1 – 7.0 (m, 6H), 5.8 (d, *J* = 7.9 Hz, 1H), 4.4 (td, *J* = 8.1, 4.9 Hz, 1H), 3.1 – 2.9 (m, 2H).

^{13}C NMR (101 MHz, DMSO-*d*$_6$) δ 173.6, 155.6, 143.2, 137.9, 129.7, 129.6, 128.6, 127.6, 126.9, 126.3, 117.2, 55.0, 36.8.

HRMS (ESI-TOF) *m/z:* $[M+Na]^+$ Calcd for $C_{22}H_{20}N_2NaO_3^+$ 383.1366, found 383.1360.

([1,1'-biphenyl]-4-carbonyl)phenylalanine **1al**

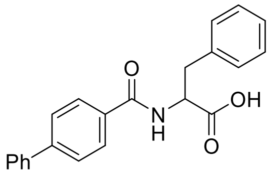

Following **General Procedure 1**, the desired product **1al** was afforded as a white solid with a 96% yield (3.3 g).

TLC (100% EtOAc): R_f = 0.5

^{1}H NMR (400 MHz, DMSO-*d*$_6$) δ 8.8 (d, *J* = 8.2 Hz, 1H), 7.9 (d, *J* = 8.3 Hz, 2H), 7.7 (dd, *J* = 14.9, 7.8 Hz, 4H), 7.5 (t, *J* = 6.7 Hz, 2H), 7.4 (q, *J* = 7.5 Hz, 1H), 7.4 – 7.2 (m, 4H), 7.2 (t, *J* = 7.2 Hz, 1H), 4.7 (ddd, *J* = 10.6, 8.1, 4.4 Hz, 1H), 3.2 (dd, *J* = 13.8, 4.5 Hz, 1H), 3.1 (dd, *J* = 13.7, 10.8 Hz, 1H).

^{13}C NMR (101 MHz, DMSO-*d*6) δ 173.7, 166.5, 143.4, 139.6, 138.7, 133.2, 130.4, 129.5, 129.5, 128.7, 128.5, 127.3, 126.9, 54.7, 36.7.

HRMS (ESI-TOF) *m/z:* $[M+Na]^+$ Calcd for $C_{22}H_{19}NNaO_3^+$ 368.1257, found 368.1258.

(4-(tert-butyl)benzoyl)phenylalanine **1am**

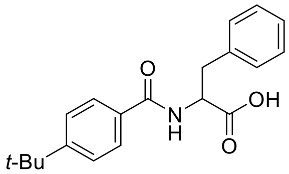

Following **General Procedure 1**, the desired product **1a** was obtained as a white solid with a 93% yield (3.0 g).

TLC (100% EtOAc): R_f = 0.5

^{1}H NMR (400 MHz, DMSO-*d*$_6$) δ 7.8 – 7.7 (m, 2H), 7.5 (d, *J* = 8.5 Hz, 2H), 7.4 (d, *J* = 7.1 Hz, 2H), 7.3 (t, *J* = 7.4 Hz, 2H), 7.2 (t, *J* = 7.1 Hz, 1H), 5.0 – 4.9 (m, 1H), 3.4 (dd, *J* = 13.9, 5.0 Hz, 2H), 3.2 (dd, *J* = 13.9, 9.0 Hz, 2H), 1.3 (s, 9H).

^{13}C NMR (101 MHz, DMSO-*d*$_6$) δ 172.4, 166.5, 154.6, 137.7, 131.7, 129.2, 128.3, 127.1, 126.5, 125.2, 53.9, 37.0, 34.5, 30.5, 30.5.

HRMS (ESI-TOF) *m/z:* $[M+Na]^+$ Calcd for $C_{20}H_{23}NNaO_3^+$ 348.1570, found 348.1567.

(4-bromobenzoyl)phenylalanine **1an**

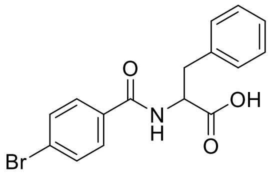

Following **General Procedure 1**, the desired product **1an** was afforded as a white solid with an 89% yield (3.1 g).

TLC (100% EtOAc): R_f = 0.6

^{1}H NMR (400 MHz, Acetone-d_6) δ 8.0 – 7.8 (m, 2H), 7.8 – 7.5 (m, 4H), 7.4 – 7.1 (m, 4H), 4.9 (dddd, J = 8.9, 7.5, 3.9, 2.2 Hz, 1H), 3.5 – 3.0 (m, 2H).

^{13}C NMR (101 MHz, Acetone-d_6) δ 172.3, 165.7, 137.7, 133.5, 131.7, 131.5, 131.4, 129.6, 129.2, 128.3, 126.6, 125.4, 54.1, 37.0.

HRMS (ESI-TOF) *m/z:* $[M+Na]^+$ Calcd for $C_{16}H_{14}BrNNaO_3^+$ 370.0049, found 370.0049.

(4-nitrobenzoyl)phenylalanine **1ao**

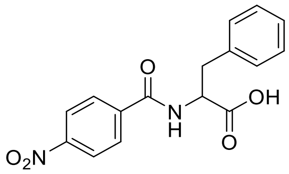

Following **General Procedure 2**, the desired product **1ao** was afforded as a yellow solid with a 53% yield (1.7 g).

TLC (100% EtOAc): R_f = 0.3

^{1}H NMR (400 MHz, DMSO-d_6) δ 8.4 (d, J = 8.6 Hz, 1H), 8.3 – 8.3 (m, 2H), 8.2 (d, J = 8.1 Hz, 1H), 8.1 (d, J = 8.6 Hz, 2H), 7.4 (d, J = 7.5 Hz, 2H), 7.3 (t, J = 7.5 Hz, 2H), 7.2 (t, J = 7.3 Hz, 1H), 5.0 (td, J = 8.8, 5.0 Hz, 1H), 3.4 (dd, J = 14.0, 4.9 Hz, 2H).

^{13}C NMR (101 MHz, DMSO-d_6) δ 130.9, 129.2, 128.7, 128.3, 126.6, 123.5, 123.4, 54.3, 37.0.

HRMS (ESI-TOF) *m/z:* $[M+Na]^+$ Calcd for $C_{16}H_{14}N_2NaO_5^+$ 337.0795, found 337.0795.

(4-(trifluoromethyl)benzoyl)phenylalanine **1ap**

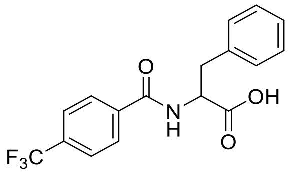

Following **General Procedure 2**, the desired product **1ap** was obtained as a white solid with a 60% yield (2.0 g).

TLC (100% EtOAc): R_f = 0.6

^{1}H NMR (400 MHz, DMSO-d_6) δ 13.1 (s, 1H), 9.0 (d, J = 8.2 Hz, 1H), 8.0 (d, J = 8.2 Hz, 2H), 7.8 (d, J = 8.2 Hz, 2H), 7.4 – 7.2 (m, 4H), 7.2 – 7.2 (m, 1H), 4.8 – 4.5 (m, 1H), 3.3 – 3.0 (m, 3H).

^{13}C NMR (101 MHz, DMSO-*d*$_6$) δ 173.4, 166.7, 165.7, 138.5, 138.1, 134.1 (d, *J* = 202.6 Hz), 131.7 (d, *J* = 31.5 Hz), 130.6, 129.5, 128.7, 125.8 (d, *J* = 3.8 Hz), 54.8, 36.7.
^{19}F NMR (376 MHz, DMSO-*d*$_6$) δ -61.4.
HRMS (ESI-TOF) *m/z:* $[M+Na]^+$ Calcd for $C_{17}H_{14}F_3NNaO_3^+$ 360.0818, found 360.0818.

(3-methoxybenzoyl)phenylalanine **1aq**

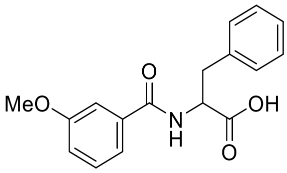

Following **General Procedure 2**, the desired product **1aq** was obtained as a white solid with a 72% yield (2.1 g).
TLC (100% EtOAc): R_f = 0.5
^{1}H NMR (400 MHz, DMSO-*d*$_6$) δ 8.7 (d, *J* = 8.2 Hz, 1H), 7.4 – 7.2 (m, 7H), 7.2 (t, *J* = 7.1 Hz, 1H), 7.1 (dt, *J* = 5.7, 2.9 Hz, 1H), 4.6 (ddd, *J* = 10.6, 8.1, 4.4 Hz, 1H), 3.8 (s, 3H), 3.2 (dd, *J* = 13.8, 4.5 Hz, 1H), 3.1 (dd, *J* = 13.8, 10.7 Hz, 1H).
^{13}C NMR (101 MHz, DMSO-*d*$_6$) δ 173.6, 166.6, 159.5, 138.6, 135.8, 129.9, 129.5, 128.7, 126.8, 120.0, 117.6, 113.0, 55.7, 54.7, 36.7.
HRMS (ESI-TOF) *m/z:* $[M+Na]^+$ Calcd for $C_{17}H_{17}NNaO_4^+$ 322.1050, found 322.1049.

(3,5-dimethoxybenzoyl)phenylalanine **1ar**

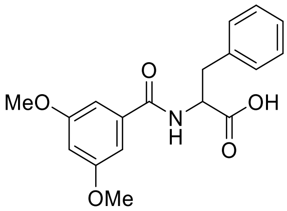

Following **General Procedure 1**, the desired product **1ar** was obtained as a white solid with a 72% yield (2.3 g).
TLC (100% EtOAc): R_f = 0.5
^{1}H NMR (400 MHz, Acetone-*d*$_6$) δ 7.9 (d, *J* = 8.2 Hz, 1H), 7.5 – 7.4 (m, 2H), 7.4 (t, *J* = 7.6 Hz, 2H), 7.3 – 7.2 (m, 1H), 7.0 (d, *J* = 2.3 Hz, 2H), 6.7 (t, *J* = 2.3 Hz, 1H), 5.0 (ddd, *J* = 9.3, 8.2, 4.9 Hz, 1H), 3.9 (s, 6H), 3.7 (q, *J* = 7.0 Hz, 1H), 3.4 (dd, *J* = 13.9, 4.9 Hz, 1H), 3.2 (dd, *J* = 13.9, 9.4 Hz, 1H).
^{13}C NMR (101 MHz, Acetone-*d*$_6$) δ 172.4, 166.4, 160.9, 137.8, 136.6, 129.3, 128.3, 126.6, 107.1, 105.1, 103.2, 54.9, 54.1, 37.0.

HRMS (ESI-TOF) *m/z:* $[M+Na]^+$ Calcd for $C_{18}H_{19}NNaO_5^+$ 352.1155, found 352.1155.

(1-naphthoyl)phenylalanine **1as**

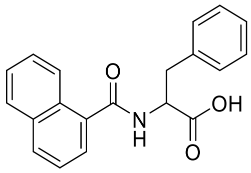

Following **General Procedure 1**, the desired product **1as** was afforded as a white solid with an 82% yield (2.6 g).

TLC (100% EtOAc): R_f = 0.5

^{1}H NMR (400 MHz, DMSO-*d*$_6$) δ 12.8 (s, 1H), 8.9 (d, *J* = 8.2 Hz, 1H), 8.4 (d, *J* = 1.7 Hz, 1H), 8.0 – 7.9 (m, 4H), 7.9 (dd, *J* = 8.6, 1.7 Hz, 1H), 7.7 – 7.6 (m, 2H), 7.4 (d, *J* = 7.1 Hz, 2H), 7.3 (t, *J* = 7.5 Hz, 2H), 7.2 (t, *J* = 7.2 Hz, 1H), 4.8 – 4.6 (m, 1H), 3.3 – 3.0 (m, 3H).

^{13}C NMR (101 MHz, DMSO-*d*$_6$) δ 173.7, 166.8, 138.7, 134.7, 132.5, 131.7, 129.6, 129.3, 128.7, 128.3, 128.1, 128.1, 127.2, 126.8, 124.7, 54.8, 36.8.

HRMS (ESI-TOF) *m/z:* $[M+Na]^+$ Calcd for $C_{20}H_{17}NNaO_3^+$ 342.1101, found 342.1101.

(cyclopropanecarbonyl)phenylalanine **1at**

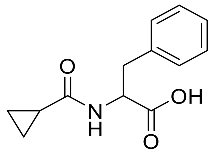

Following **General Procedure 2**, the desired product **1at** was obtained as a colorless oil with a 89% yield (2.0 g).

TLC (100% EtOAc): R_f = 0.5

^{1}H NMR (400 MHz, Acetone-*d*$_6$) δ 7.8 (d, *J* = 8.1 Hz, 1H), 7.5 – 7.2 (m, 5H), 4.9 (td, *J* = 8.1, 5.3 Hz, 1H), 3.4 – 3.1 (m, 2H), 1.8 (tt, *J* = 7.9, 4.6 Hz, 1H), 0.9 – 0.9 (m, 2H), 0.8 – 0.7 (m, 2H).

HRMS (ESI-TOF) *m/z:* $[M+Na]^+$ Calcd for $C_{13}H_{15}NNaO_3^+$ 256.0944, found 256.0943.

(thiophene-2-carbonyl)phenylalanine **1au**

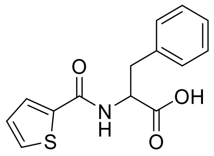

Following **General Procedure 2**, the desired product **1au** was obtained as a white solid with a 77% yield (2.1 g).

TLC (100% EtOAc): R_f = 0.4

^{1}H NMR (400 MHz, DMSO-*d*$_6$) δ 12.8 (s, 1H), 8.8 (d, *J* = 8.2 Hz, 1H), 7.8 (ddd, *J* = 35.5, 4.4, 1.1 Hz, 2H), 7.5 – 6.9 (m, 6H), 4.6 (ddd, *J* = 10.6, 8.2, 4.5 Hz, 1H), 3.2 – 2.9 (m, 2H).

^{13}C NMR (101 MHz, DMSO-*d*$_6$) δ 173.5, 161.6, 139.8, 138.5, 131.5, 129.5, 129.0, 128.7, 128.4, 126.9, 54.5, 36.7.

HRMS (ESI-TOF) *m/z:* $[M+Na]^+$ Calcd for $C_{14}H_{13}NNaO_3S^+$ 298.0508, found 298.0508.

Synthesis of peptides [3]

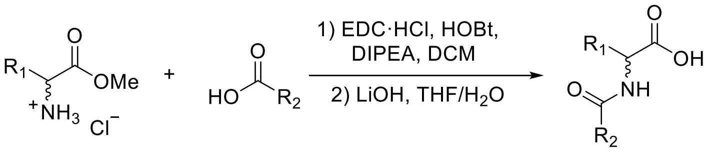

To a suspension of N-protected amino acid (3.00 mmol, 1.0 equiv) and amino acid methyl ester hydrochloride (3.30 mmol, 1.1 equiv) in CH_2Cl_2 (20 mL) at room temperature was added 1-(3-dimethylaminopropyl)-3-ethylcarbodiimide hydrochloride (EDC·HCl, 3.30 mmol, 1.1 equiv), 1-hydroxybenzotriazole (HOBt, 86% w/w, 3.00 mmol, 1.0 equiv) and N,Ndiisopropylethylamine (DIPEA, 9.0 mmol, 3.0 equiv) The reaction mixture was stirred vigorously at room temperature overnight. Upon completion, the reaction mixture was concentrated under reduced pressure. The resultant crude residue was purified by flash column chromatography to give the ester. To a solution of the ester (5 mmol) in THF (6 mL) at 0 °C, aqueous LiOH (2 M, 3 mL, 1.2 equiv) was added. The reaction mixture was warmed to room temperature and stirred overnight. Upon completion, aqueous HCl solution (1 M) was added to the reaction mixture, and the pH was carefully adjusted to 4-5. The reaction was extracted with EtOAc (3 × 20 mL), and the combined organic layer was dried over Na_2SO_4, filtered, and concentrated under reduced pressure to yield the peptides, which were used without further purification.

benzoylglycylphenylalanine **5a**

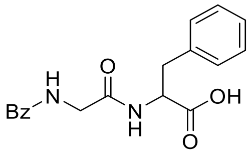

Following the **General Procedure**, the desired product **5a** was obtained as a white solid with a 71% yield (694 mg).

^{1}H NMR (400 MHz, DMSO-d_6) δ 8.7 (t, J = 6.0 Hz, 1H), 8.2 – 8.1 (m, 2H), 7.9 – 7.9 (m, 2H), 7.6 – 7.5 (m, 1H), 7.5 (dd, J = 8.2, 6.6 Hz, 2H), 7.2 (d, J = 5.9 Hz, 5H), 4.4 (ddd, J = 8.1, 6.7, 3.7 Hz, 2H), 3.1 (q, J = 5.0, 4.3 Hz, 2H), 2.9 (dd, J = 13.7, 8.4 Hz, 2H).

^{13}C NMR (101 MHz, DMSO-d_6) δ 173.3, 169.3, 166.9, 138.0, 134.4, 131.8, 129.7, 128.7, 127.8, 126.8, 116.3, 54.1, 42.8, 37.3.

HRMS (ESI-TOF) *m/z:* $[M+Na]^+$ Calcd for $C_{18}H_{18}N_2NaO_4^+$ 349.1159, found 349.1151.

(tert-butoxycarbonyl)-L-alanylphenylalanine **5b**

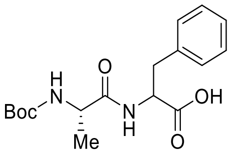

Following the **General Procedure**, the desired product **5b** was obtained as a white solid with a 69% yield (695 mg).

^{1}H NMR (400 MHz, Acetone-d_6) δ 7.7 (d, J = 8.1 Hz, 1H), 7.6 (d, J = 8.1 Hz, 1H), 7.3 (d, J = 3.1 Hz, 5H), 6.3 (d, J = 7.7 Hz, 1H), 4.9 (dt, J = 8.2, 4.2 Hz, 2H), 3.3 – 3.3 (m, 1H), 3.1 – 3.1 (m, 1H), 1.5 (s, 9H), 1.3 (d, J = 7.1 Hz, 3H).

^{13}C NMR (101 MHz, Acetone-d_6) δ 172.7, 172.1, 137.1, 137.0, 129.4, 128.3, 128.3, 126.6, 78.5, 53.1, 37.2, 29.7, 27.7, 18.3.

HRMS (ESI-TOF) *m/z:* $[M+Na]^+$ Calcd for $C_{17}H_{24}N_2NaO_5^+$ 359.1577, found 359.1576.

(tert-butoxycarbonyl)-L-phenylalanylglycylphenylalanine **5e**

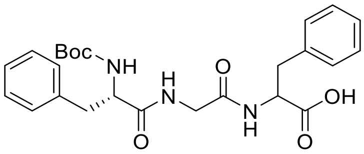

Following the **General Procedure**, the desired product **5e** was obtained as a white solid with a 47% yield (661 mg).

^{1}H NMR (400 MHz, DMSO-d_6) δ 8.2 (dd, J = 6.9, 4.5 Hz, 2H), 7.3 – 7.1 (m, 11H), 7.0 (d, J = 8.6 Hz, 1H), 4.4 (td, J = 8.5, 5.0 Hz, 2H), 3.8 – 3.7 (m, 2H), 3.1 – 2.8 (m, 4H), 1.3 (s, 9H).

^{13}C NMR (101 MHz, DMSO-d_6) δ 173.2, 169.1, 138.8, 138.0, 129.6, 128.6, 128.4, 126.9, 126.6, 78.5, 56.5, 55.4, 54.1, 37.9, 37.3, 28.6.

HRMS (ESI-TOF) *m/z:* $[M+Na]^+$ Calcd for $C_{25}H_{31}N_3NaO_6^+$ 492.2105, found 492.2107.

(tert-butoxycarbonyl)-L-phenylalanyl-L-alanylphenylalanine **5f**

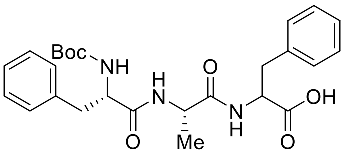

Following the **General Procedure**, the desired product **5f** was obtained as a white solid with a 47% yield (661 mg).

^{1}H NMR (400 MHz, Acetone-*d*$_6$) δ 7.3 – 7.1 (m, 12H), 4.7 – 4.7 (m, 1H), 4.4 (dt, *J* = 36.4, 6.4 Hz, 2H), 3.2 – 2.9 (m, 4H), 1.3 (d, *J* = 13.8 Hz, 12H).

^{13}C NMR (101 MHz, Acetone-*d*$_6$) δ 172.2, 171.9, 171.3, 137.9, 137.5, 137.2, 129.4, 129.3, 128.3, 128.2, 128.1, 126.6, 126.5, 126.3, 78.6, 55.6, 53.5, 48.6, 37.9, 37.1, 27.6, 21.7, 17.7.

HRMS (ESI-TOF) *m/z:* $[M+Na]^+$ Calcd for $C_{26}H_{33}N_3NaO_6^+$ 506.2262, found 506.2265.

(tert-butoxycarbonyl)-L-phenylalanylglycyl-L-tryptophan **5g**

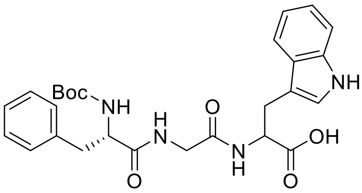

Following the **General Procedure**, the desired product **5g** was obtained as a white solid with a 53% yield (807 mg).

^{1}H NMR (400 MHz, DMSO-*d*$_6$) δ 10.9 (s, 1H), 8.1 (d, *J* = 8.5 Hz, 1H), 7.5 (d, *J* = 7.8 Hz, 1H), 7.4 – 7.3 (m, 1H), 7.3 (d, *J* = 4.4 Hz, 3H), 7.2 – 7.1 (m, 2H), 7.1 (t, *J* = 7.5 Hz, 1H), 7.0 – 6.9 (m, 2H), 4.5 (tt, *J* = 8.1, 3.8 Hz, 1H), 4.2 (td, *J* = 10.7, 10.3, 3.9 Hz, 1H), 3.7 (qdd, *J* = 14.1, 9.3, 5.5 Hz, 3H), 3.2 (dd, *J* = 14.6, 5.3 Hz, 2H), 3.1 – 3.0 (m, 2H), 2.8 – 2.7 (m, 1H), 1.3 (d, *J* = 2.5 Hz, 6H).

^{13}C NMR (101 MHz, DMSO-*d*$_6$) δ 173.6, 172.4, 169.0, 136.5, 129.6, 128.4, 127.7, 126.6, 124.2, 121.4, 118.8, 118.6, 111.8, 110.1, 78.5, 56.2, 53.5, 42.3, 28.6.

HRMS (ESI-TOF) *m/z:* $[M+Na]^+$ Calcd for $C_{27}H_{32}N_4NaO_6^+$ 531.2214, found 531.2214.

2-(2-benzamidoacetamido)pent-4-enoic acid **5h**

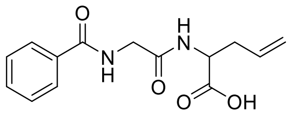

Following the **General Procedure**, the desired product **5h** was obtained as a white solid with an 82% yield (678 mg).

^{1}H NMR (400 MHz, Acetone-*d*$_6$) δ 7.9 (d, *J* = 7.0 Hz, 2H), 7.5 (t, *J* = 7.3 Hz, 1H), 7.5 (t, *J* = 7.4 Hz, 2H), 5.8 (ddt, *J* = 17.2, 10.2, 7.1 Hz, 1H), 5.1 – 5.1 (m, 1H), 5.0 (ddt, *J* = 10.3, 2.2, 1.1 Hz, 1H), 4.5 (td, *J* = 7.6, 5.2 Hz, 1H), 4.2 – 4.0 (m, 2H), 2.6 (dddt, *J* = 13.5, 6.6, 5.2, 1.3 Hz, 1H), 2.5 – 2.4 (m, 1H).

^{13}C NMR (101 MHz, Acetone-*d*$_6$) δ 134.4, 133.3, 131.4, 128.3, 127.2, 117.7, 51.7, 41.2, 35.9.

HRMS (ESI-TOF) *m/z:* $[M+Na]^+$ Calcd for $C_{14}H_{16}N_2KO_4^+$ 315.0742, found 315.0750.

(Z)-2-(2-acetamido-3-phenylacrylamido)-3-(4-nitrophenyl)propanoic acid **5i**

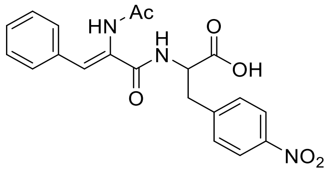

Following the **General Procedure**, the desired product **5i** was obtained as a white solid with a 52% yield (500 mg).

^{1}H NMR (400 MHz, Methanol-*d*$_4$) δ 8.2 (d, *J* = 8.8 Hz, 2H), 7.6 – 7.5 (m, 4H), 7.4 – 7.3 (m, 3H), 7.1 (s, 1H), 4.8 (dd, *J* = 8.1, 5.1 Hz, 1H), 3.4 (dd, *J* = 13.9, 5.1 Hz, 1H), 3.3 (dd, *J* = 13.9, 8.1 Hz, 1H), 2.1 (s, 3H).

^{13}C NMR (101 MHz, Methanol-*d*$_4$) δ 172.6, 171.9, 166.4, 147.0, 145.3, 133.7, 130.3, 129.9, 129.1, 128.8, 128.6, 128.3, 123.0, 53.7, 36.5, 21.2.

HRMS (ESI-TOF) *m/z:* $[M+Na]^+$ Calcd for $C_{20}H_{19}N_3NaO_6^+$ 420.1166, found 420.1167.

2-((S)-2-((((9H-fluoren-9-yl)methoxy)carbonyl)amino)propanamido)-4-phenylbutanoic acid **5j**

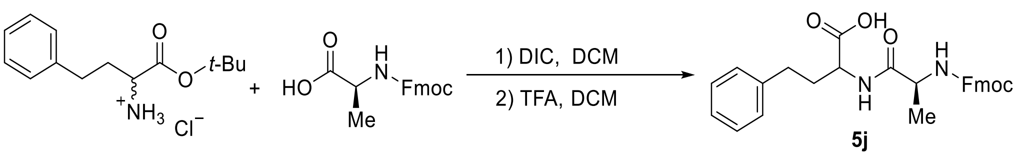

5j

To a suspension of N-protected amino acid (3.00 mmol, 1.0 equiv) and amino acid methyl ester hydrochloride (3.30 mmol, 1.1 equiv) in CH_2Cl_2 (20 mL), DIC (4 mmol,

1.2 equiv) was added at room temperature. The reaction mixture was stirred vigorously at room temperature overnight. Upon completion, the reaction mixture was concentrated under reduced pressure. The resultant crude residue was purified by flash column chromatography to give the ester.

A solution of ester in DCM (6 mL) at 0 °C was added to aqueous TFA (95%, 3 equiv). The reaction mixture was warmed to room temperature and stirred until the ester was consumed. The reaction was extracted with EtOAc (3 × 20 mL), and the combined organic layer was dried over Na_2SO_4, filtered, and concentrated under reduced pressure to yield peptides **5j** as a white solid in 71% yield (1.01 g), which was used without further purification.

^{1}H NMR (400 MHz, Acetone-*d*$_6$) δ 8.1 (d, *J* = 7.7 Hz, 1H), 7.9 (d, *J* = 7.6 Hz, 2H), 7.8 (dd, *J* = 14.0, 7.5 Hz, 2H), 7.5 (t, *J* = 7.5 Hz, 2H), 7.4 (t, *J* = 7.4 Hz, 2H), 7.3 – 7.2 (m, 6H), 4.6 – 4.4 (m, 3H), 4.3 (t, *J* = 7.4 Hz, 1H), 3.9 (p, *J* = 6.5 Hz, 1H), 2.8 (td, *J* = 10.6, 10.0, 5.6 Hz, 2H), 2.2 – 2.1 (m, 2H), 1.5 (d, *J* = 7.1 Hz, 3H).

^{13}C NMR (101 MHz, Acetone-*d*$_6$) δ 173.6, 170.5, 156.7, 144.3, 144.0, 141.7, 141.2, 128.4, 128.2, 127.7, 127.1, 125.7, 125.3, 119.9, 66.7, 59.8, 50.8, 47.0, 41.3, 31.9, 20.0.

HRMS (ESI-TOF) *m/z:* $[M+Na]^+$ Calcd for $C_{28}H_{28}N_2NaO_5^+$ 511.1630, found 511.1636.

Synthesis of internal intermediate azlactone Int-1a [4]

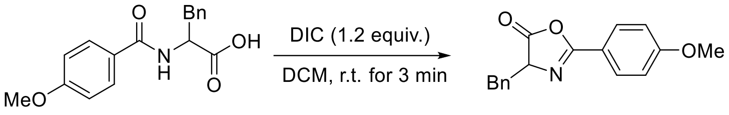

(*rac*)-**1a** Int-**1a**

A 15 mL single bottle charged with a PTFE-coated magnetic stir bar (*rac*)-**1a** (1 mmol) was added. The bottle was transferred into a nitrogen-filled purge box. Dry and degassed DCM (5.0 mL) and DIC (1.2 mmol, 1.2 equiv.) were added. The culture tube was then capped with a polypropylene screw cap with a PTFE-faced silicone septum and removed from the purge box. After 3 minutes, column chromatography was used to concentrate and purify the crude reaction mixture, yielding **Int-1a** (98% recovery).

4-benzyl-2-(4-methoxyphenyl)oxazol-5(4H)-one **Int-1a**

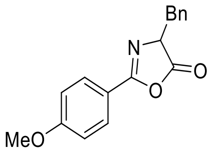

Following the **General Procedure**, the desired product **Int-1a** was obtained as a white solid with a 98% yield (275 mg).

TLC (6:1, PE: EA): R_f = 0.6

^{1}H NMR (400 MHz, $CDCl_3$) δ 7.9 (d, *J* = 8.9 Hz, 2H), 7.3 – 7.2 (m, 5H), 6.9 (d, *J* = 8.9 Hz, 2H), 4.7 (dd, *J* = 6.7, 4.9 Hz, 1H), 3.9 (s, 3H), 3.4 (dd, *J* = 14.0, 5.0 Hz, 1H), 3.2 (dd, *J* = 14.0, 6.7 Hz, 1H).

^{13}C NMR (101 MHz, $CDCl_3$) δ 177.8, 163.2, 161.5, 135.4, 129.8, 129.6, 128.4, 127.2, 118.1, 114.2, 66.4, 55.5, 37.4.

HRMS (ESI-TOF) *m/z:* $[M+Na]^+$ Calcd for $C_{17}H_{15}NNaO_3^+$ 304.0950, found 304.0951.

Reaction optimization

Table S1 General procedure for the screening of different NHC precursors.

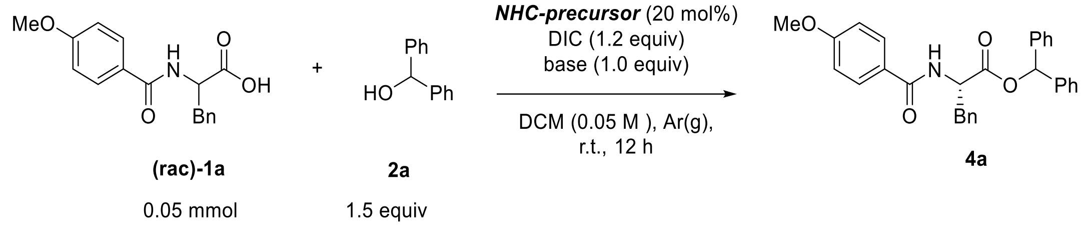

Entry	NHC	Yield of **4a** (%) [a]	ee (%) [b]
1	***NHC-3a***	91	99
2	***NHC-3b***	87	98
3	***NHC-3c***	84	91
4	***NHC-3d***	73	34
5	***NHC-3e***	22	98
6	***NHC-3f***	85	95
7	***NHC-3g***	88	90
8	***NHC-3h***	92	98
9	***NHC-3i***	15	84
10	***NHC-3j***	10	92
11	***NHC-3k***	88	11
12	***NHC-3l***	94	77
13	***NHC-3m***	98	92
14	***NHC-3n***	92	-1
15	***NHC-3o***	75	93
16	***NHC-3p***	35	90
17	***NHC-3q***	72	36
18	***NHC-3r***	86	65
19	***NHC-3s***	89	13
20	***NHC-3t***	78	-92
21	***NHC-3u***	61	71
22	***NHC-3v***	65	1
23	***NHC-3w***	74	84
21	***NHC-3a*** (10 mol%)	82	85

Reaction conditions: A mixture of **1a** (0.05 mmol, 1.0 eq.), diphenylmethanol (0.075 mmol, 1.5 eq.), NHC-precursor (20 mol%), Cs_2CO_3 (0.05 mmol, 1.0 eq.) and DIC (0.06 mmol, 1.2 eq.) dissolved in DCM (0.05 M) was stirred at r.t. for 2-10 hours. [a] Isolated yields are presented. [b] Chiral HPLC determined *ee* values.

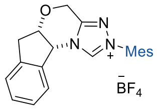
3a

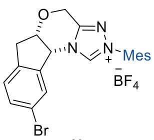
3b

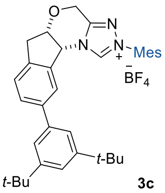
3c

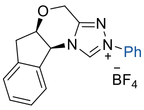
3d

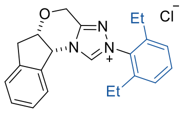
3e

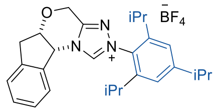
3f

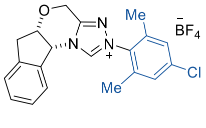
3g

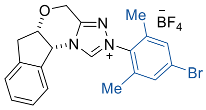
3h

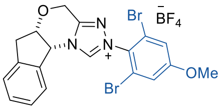
3i

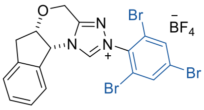
3j

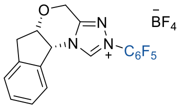
3k

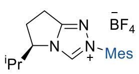
3l

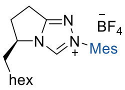
3m

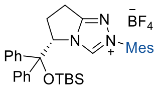
3n

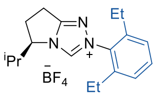
3o

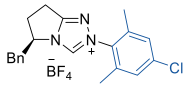
3p

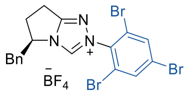
3q

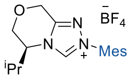
3r

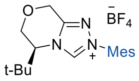
3s

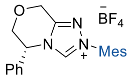
3t

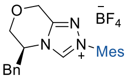
3u

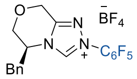
3v

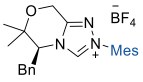
3w

Table S2 General procedure for screening the base.

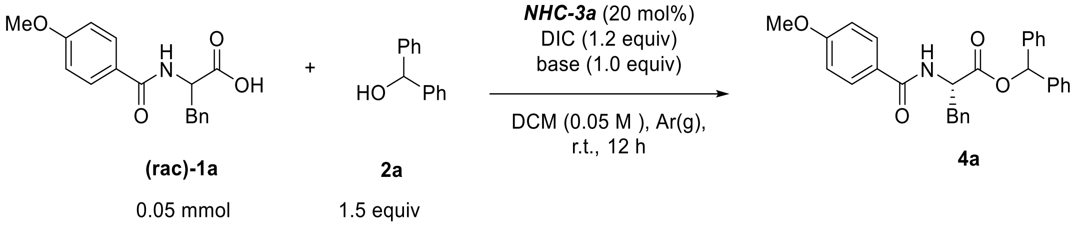

Entry	Base	Yield of **4a** (%) [a]	ee (%) [b]
1	K_2CO_3	99	96
2	Na*t*BuO	84	1
3	K_2PO_4	97	96
4	KH_2PO_4	81	98
5	KOAc	90	94
6	DABCO	53	74
7	Et_3N	30	97
8	DBU	98	1
9	LiOH	99	95
10	LiHMDS	99	24
11	Cs_2CO_3 (0.3 equiv.)	81	99
12	Cs_2CO_3 (0.5 equiv.)	90	99
13	Cs_2CO_3 (1 equiv.)	91	99
14	Cs_2CO_3 (1.5equiv.)	90	97

Reaction conditions: A mixture of **1a** (0.05 mmol, 1.0 eq.), and diphenylmethanol (0.075 mmol, 1.5 eq.), NHC-**3a** (20 mol%), Base (0.05 mmol, 1.0 eq.) and DIC (0.06 mmol, 1.2 eq.) dissolved in DCM (0.05 M) was stirred at r.t. for 2-10 hours. [a] Isolated yields are presented. [b] Chiral HPLC determined *ee* values.

Table S3 General procedure for the screening of the solvent.

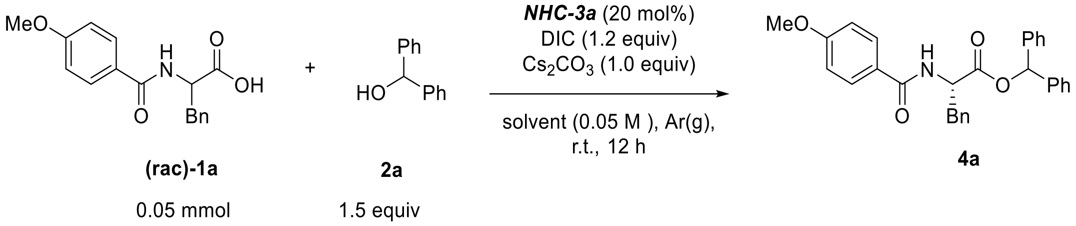

Entry	Solvent	Yield of **4a** (%) [a]	ee (%) [b]
1	DCE	97	97
2	1,4-dioxane	90	3
3	THF	82	0
4	Toluene	88	86
5	MeCN	99	0
6	DMF	54	6
7	EA	99	0
8	DMSO	Trace	62
9	Benzene	91	84
10	DCM, 0.2 eq. H_2O	97	97
11	DCM (0.025 M)	94	98
12	DCM (0.05 M)	91	99
13	DCM (0.1 M)	79	95
14	DCM (0.15 M)	88	95

Reaction conditions: A mixture of **1a** (0.05 mmol, 1.0 eq.), and diphenylmethanol (0.075 mmol, 1.5 eq.), NHC-**3a** (20 mol%), Cs_2CO_3 (0.05 mmol, 1.0 eq.) and DIC (0.06 mmol, 1.2 eq.) dissolved in solvent (0.05 M) was stirred at r.t. for 2-10 hours. [a] Isolated yields are presented. [b] Chiral HPLC determined *ee* values.

Figure S1 Process of the NHC-catalyst-controlled enantioselective transformation of oxazolone **Int-1a**.

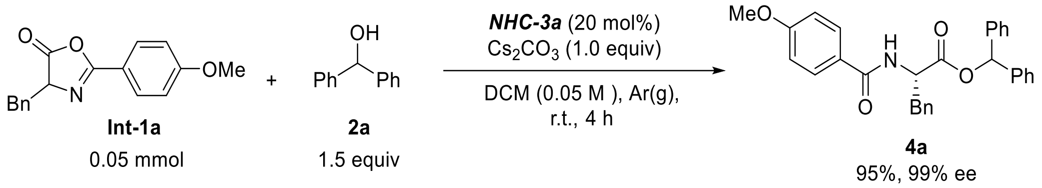

To a 10 mL flame-dry Schlenk reaction tube equipped with a magnetic stir bar, **Int-1a** (0.05 mmol), alcohol **2a** (0.075 mmol, 1.5 equiv.), **NHC-3a** (0.01 mmol, 20% mol) and Cs_2CO_3 (0.05 mmol, 1 equiv.) in the glove box, DCM (1 mL) was then added. The mixture was degassed and backfilled with argon (3x). The tube was sealed with a screw cap, and the reaction mixture was stirred vigorously at room temperature (25°C) for 6 hours. Upon complete consumption of **Int-1a**, the reaction was purified by flash column chromatography (eluent: PE/EA= 4/1) to afford the desired product **4**a.

Reconstruction of (U)AAs ester

Figure S2 General process of the NHC-catalyst-controlled enantioselective transformation of α-amino acids.

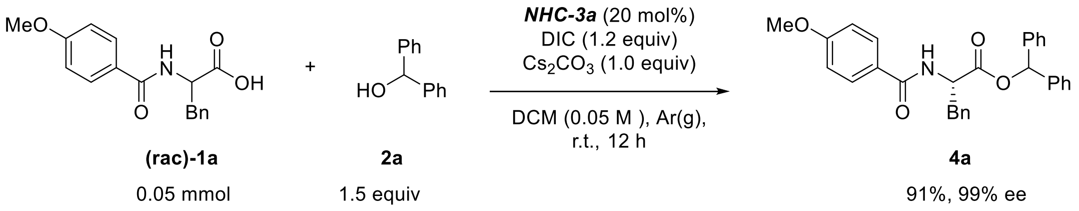

In an oven-dried 5 mL Schlenk tube equipped with a magnetic stir bar, α-amino acids (*rac*)-**1a** (0.05 mmol), NHC precatalyst **3a** (0.01 mmol, 20 mol%), alcohol **2** (0.075 mmol, 1.5 equiv), and Cs_2CO_3 (0.05 mmol, 1 equiv) were added, followed by 1 mL of dry dichloromethane (DCM). The resulting mixture was degassed and backfilled with argon (3 cycles), after which DIC (0.06 mmol) was added. The tube was sealed with a screw cap, and the reaction mixture was stirred vigorously at room temperature (25°C) for 10 hours. Upon completion (as monitored by TLC), the crude mixture was purified by flash column chromatography (petroleum ether/ethyl acetate = 10:1 to 4:1) to afford the corresponding ester products **4**a.

General process of synthesis of racemic amino ester.

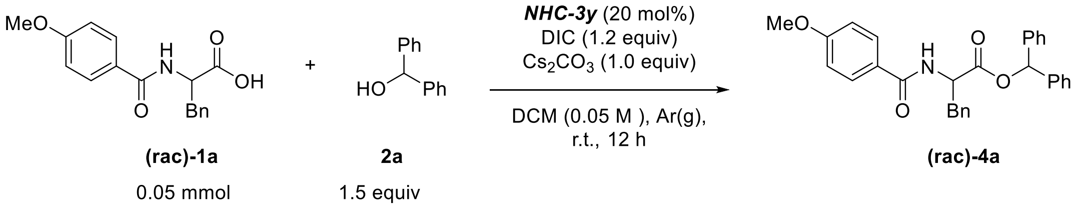

In an oven-dried 5 mL Schlenk tube equipped with a magnetic stir bar, α-amino acids (*rac*)-**1a** (0.05 mmol), NHC precatalyst **3y** (0.01 mmol, 20 mol%), alcohol **2** (0.075 mmol, 1.5 equiv), and Cs_2CO_3 (0.05 mmol, 1 equiv) were added, followed by 1 mL of dry dichloromethane (DCM). The resulting mixture was degassed and backfilled with argon (3 cycles), after which DIC (0.06 mmol) was added. The tube was sealed with a screw cap, and the reaction mixture was stirred vigorously at room temperature (25°C) for 10 hours. Upon completion (as monitored by TLC), the crude mixture was purified by flash column chromatography (petroleum ether/ethyl acetate = 10:1 to 4:1) to afford the corresponding ester products (*rac*)-**4**a.

Figure S3 Additional scope of different N-PGs and peptides.[a]

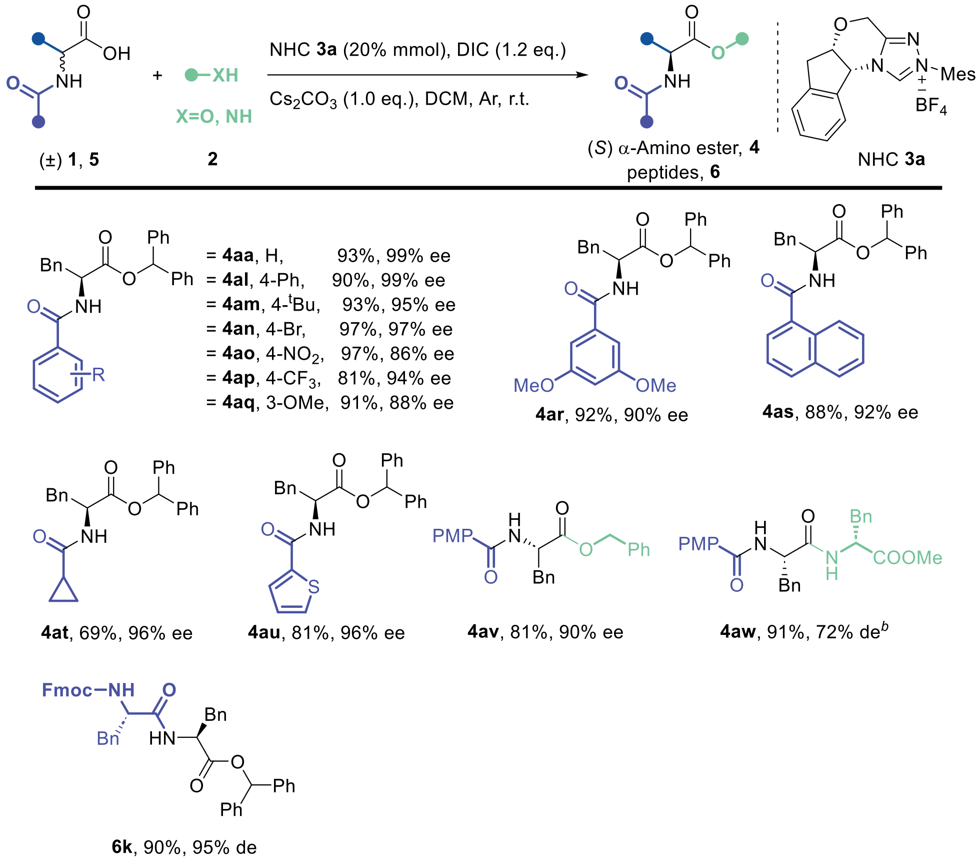

[a] Reaction conditions: A mixture of α-AAs **1** or peptides **5** (0.05 mmol), alcohol (0.075 mmol), NHC-**3a** (20 mol%), Cs_2CO_3 (0.05 mmol) and DIC (0.06 mmol) dissolved in DCM (0.05 M) was stirred at r.t. for 4-10 hours. NMR yields are presented. Isolated yields are presented. Chiral HPLC determined ee or de values. [b] KOAc instead of Cs_2CO_3, NHC-**3e** instead of NHC **3a**.

Figure S4 Additional scope of NHC-catalyst-controlled enantioselective transformation of activated p-nitrophenyl ester.[a]

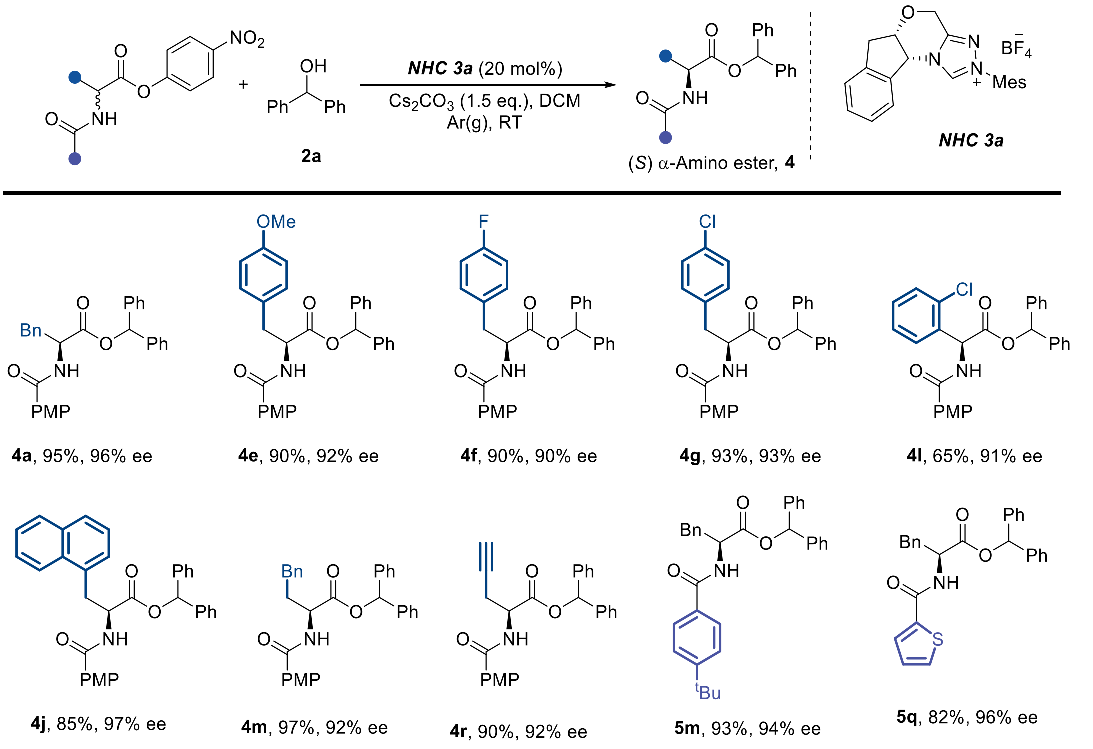

[a] Reactions were performed using *p*-nitrophenyl ester (0.05 mmol), alcohol **2a** (0.1 mmol), NHC precursor **3a** (20 mol%), and Cs_2CO_3 (1.5 equiv) in DCM (1.0 mL) at r.t. under argon for 2-3 h. Yields refer to isolated products. Ee was determined by chiral HPLC.

Figure S5 Challenge substrates.[a]

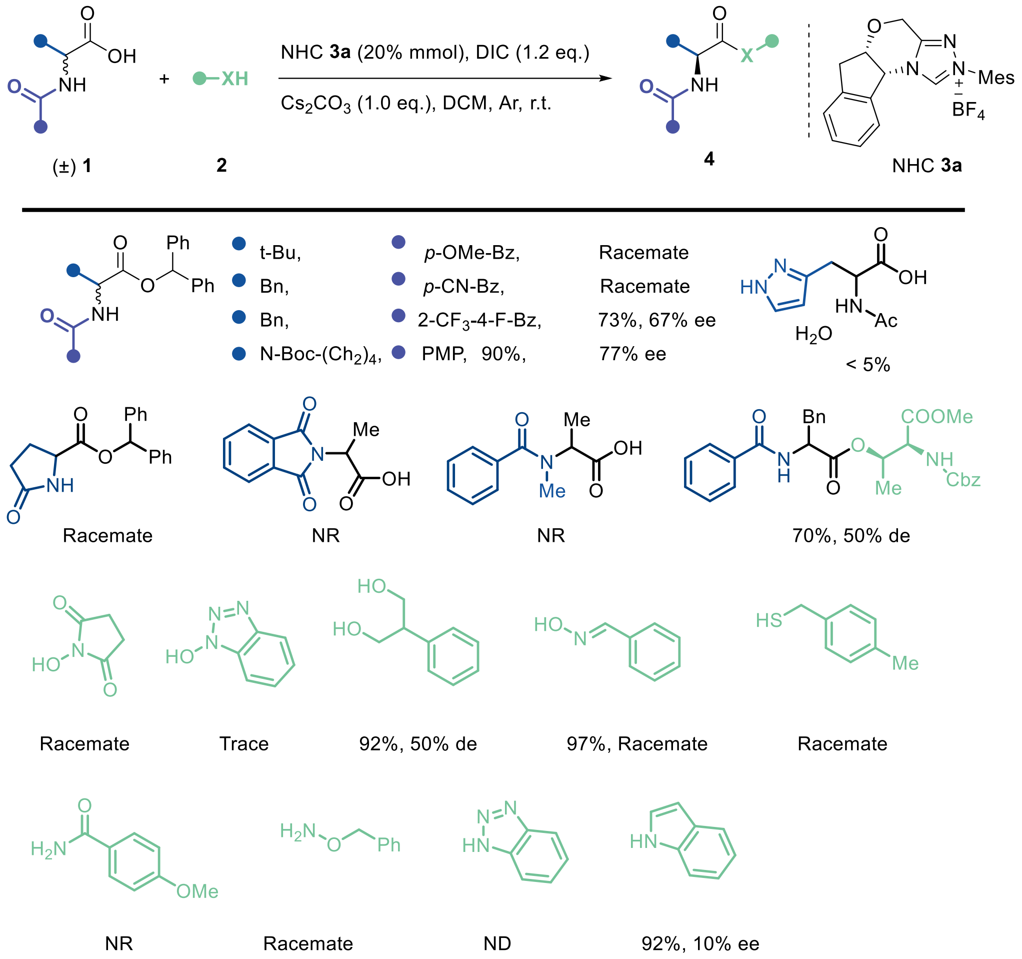

[a] Reaction conditions: A mixture of **1** (0.05 mmol, 1.0 eq.), and alcohol (0.075 mmol, 1.5 eq.), NHC-**3a** (20 mol%), Cs_2CO_3 (0.05 mmol, 1.0 eq.) and DIC (0.06 mmol, 1.2 eq.) dissolved in solvent (0.05 M) was stirred at r.t. for 2-4 hours. NMR yields are presented. Chiral HPLC determined ee and de values.

benzhydryl (4-methoxybenzoyl)-*L*-phenylalaninate **4a**

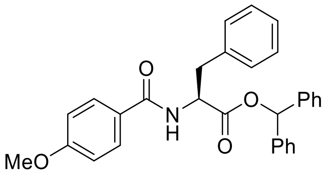

Following **General Procedure A** using α-amino acid **1a** (15 mg, 0.05 mmol, 1.0 equiv.) and diphenylmethanol (0.075 mmol, 1.5 equiv.), the crude product was purified by column chromatography petroleum ether/EtOAc (most use 8/1) as eluent to afford the desired product **4a** as a white solid in 91% yield (21.2 mg). **m.p.** 130-131 ℃.

TLC (5:1, PE: EtOAc): R_f = 0.22

^{1}H NMR (400 MHz, CDCl$_3$) δ 7.7 (d, *J* = 8.8 Hz, 2H), 7.4 – 7.3 (m, 11H), 7.2 – 7.1 (m, 3H), 7.0 – 6.8 (m, 5H), 6.5 (d, *J* = 7.6 Hz, 1H), 5.2 (ddd, *J* = 7.6, 6.0, 4.9 Hz, 1H), 3.8 (s, 3H), 3.4 – 3.1 (m, 2H).

^{13}C NMR (101 MHz, CDCl$_3$) δ 170.9, 166.3, 162.4, 139.4, 135.6, 129.5, 128.8, 128.7, 128.6, 128.5, 128.4, 128.1, 127.8, 127.0, 126.9, 126.2, 113.8, 78.3, 55.4, 53.4, 37.8.

HRMS (ESI-TOF) *m/z:* $[M+Na]^+$ Calcd for $C_{30}H_{27}NNaO_4^+$ 488.1832, found 488.1833.

HPLC (Chiralpak-IC column, Hexane/^{i}PrOH = 80/20, flow rate: 1.0 mL/min, wavelength = 254 nm) $t_{(major)}$ = 22.107 min; $t_{(minor)}$ = 24.640 min, indicated 99% ee.

$[\alpha]_D^{28}$ = +29.3 (c = 1 in $CHCl_3$).

benzhydryl (4-methoxybenzoyl)-L-tryptophanate **4b**

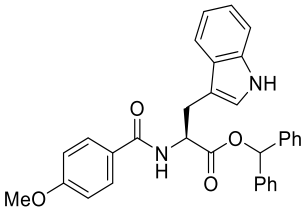

Following **General Procedure A** using α-amino acid **1b** (17 mg, 0.05 mmol, 1.0 equiv.) and diphenylmethanol (0.075 mmol, 1.5 equiv.), the crude product was purified by column chromatography petroleum ether/EtOAc (most use 6/1) as eluent to afford the desired product **4b** as a yellow solid in 92% yield (23.2 mg). **m.p.** 73-74 ℃.

TLC (5:1, PE: EtOAc): R_f = 0.4

^{1}H NMR (400 MHz, CDCl$_3$) δ 8.0 (s, 1H), 7.7 – 7.6 (m, 2H), 7.5 (d, *J* = 7.9 Hz, 1H), 7.4 – 7.3 (m, 10H), 7.2 – 7.1 (m, 1H), 7.1 – 7.0 (m, 1H), 6.9 (s, 1H), 6.8 (d, *J* = 8.8 Hz, 2H), 6.6 (d, *J* = 7.9 Hz, 1H), 6.5 (d, *J* = 2.4 Hz, 1H), 5.4 – 5.3 (m, 1H), 3.8 (s, 3H), 3.6 – 3.4 (m, 2H).

^{13}C NMR (101 MHz, CDCl$_3$) δ 171.2, 166.5, 162.3, 139.7, 139.6, 136.0, 129.0, 128.7, 128.5, 128.2, 128.0, 127.8, 127.6, 126.9, 126.2, 123.0, 122.2, 119.7, 118.8, 113.7, 111.2, 109.7, 55.4, 53.6, 27.7.

HRMS (ESI-TOF) *m/z:* $[M+Na]^+$ Calcd for $C_{32}H_{28}N_2NaO_4^+$ 527.1941, found 527.1943.

HPLC (Chiralpak-IA column, Hexane/^{i}PrOH = 90/10, flow rate: 1.0 mL/min, wavelength = 250 nm) $t_{(major)}$ = 7.161 min; $t_{(minor)}$ = 10.763 min, indicated 90% ee.

$[\alpha]_D^{28}$ = +36 (c = 1 in $CHCl_3$).

benzhydryl (4-methoxybenzoyl)-L-alaninate **4c**

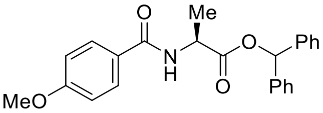

Following **General Procedure A** using α-amino acid **1c** (11.2 mg, 0.05 mmol, 1.0 equiv.) and diphenylmethanol (0.075 mmol, 1.5 equiv.), the crude product was purified by column chromatography petroleum ether/EtOAc (most use 5/1) as eluent to afford the desired product **4c** as a colorless oil in 71% yield (13.8 mg).

TLC (5:1, PE: EtOAc): R_f = 0.4

^{1}H NMR (400 MHz, CDCl$_3$) δ 7.8 – 7.7 (m, 2H), 7.4 – 7.3 (m, 10H), 6.9 – 6.8 (m, 3H), 6.7 (d, *J* = 7.3 Hz, 1H), 4.9 (p, *J* = 7.1 Hz, 1H), 3.8 (s, 3H), 1.6 (d, *J* = 7.1 Hz, 3H).

^{13}C NMR (101 MHz, CDCl$_3$) δ 172.5, 166.3, 162.4, 139.6, 139.4, 128.9, 128.7, 128.6, 128.2, 127.1, 127.0, 126.2, 113.8, 78.2, 55.4, 48.6, 18.8.

HRMS (ESI-TOF) *m/z:* $[M+Na]^+$ Calcd for $C_{24}H_{23}NNaO_4^+$ 412.1519, found 412.1519.

HPLC (Chiralpak-IB column, Hexane/^{i}PrOH = 70/30, flow rate: 1.0 mL/min, wavelength = 254 nm) $t_{(major)}$ = 6.887 min; $t_{(minor)}$ = 6.217 min, indicated 92% ee.

$[\alpha]_D^{28}$ = +17.7 (c = 1 in $CHCl_3$).

benzhydryl (4-methoxybenzoyl)-L-methioninate **4d**

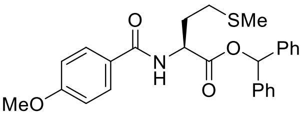

Following **General Procedure A** using α-amino acid **1d** (14.2 mg, 0.05 mmol, 1.0 equiv.) and diphenylmethanol (0.075 mmol, 1.5 equiv.), the crude product was purified by column chromatography petroleum ether/EtOAc (most use 5/1) as eluent to afford the desired product **4d** as a white solid in 90% yield (20.1 mg). **m.p.** 94-95 °C.

TLC (5:1, PE: EtOAc): R_f = 0.3

^{1}H NMR (400 MHz, $CDCl_3$) δ 7.8 – 7.7 (m, 2H), 7.4 – 7.2 (m, 10H), 7.0 – 6.9 (m, 4H), 5.1 (td, *J* = 7.2, 4.9 Hz, 1H), 3.8 (s, 3H), 2.6 – 2.4 (m, 2H), 2.4 – 2.2 (m, 1H), 2.2 – 2.1 (m, 1H), 2.0 (s, 3H).
^{13}C NMR (101 MHz, $CDCl_3$) δ 171.4, 166.6, 162.5, 139.5, 139.3, 129.0, 128.7, 128.6, 128.3, 128.2, 127.3, 127.0, 126.0, 113.8, 78.4, 55.4, 52.2, 31.7, 29.9, 15.5.
HRMS (ESI-TOF) *m/z:* $[M+Na]^+$ Calcd for $C_{26}H_{27}NNaSO_4^+$ 472.1553, found 472.1552.
HPLC (Chiralpak-IC column, Hexane/^{i}PrOH = 80/20, flow rate: 1.0 mL/min, wavelength = 206 nm) $t_{(major)}$ = 22.773 min; $t_{(minor)}$ = 24.864 min, indicated 95% ee.
$\mathbf{[\alpha]_D^{28}}$ = +24.5 (c = 1 in $CHCl_3$).

benzhydryl (S)-2-(4-methoxybenzamido)-3-(4-methoxyphenyl)propanoate **4e**

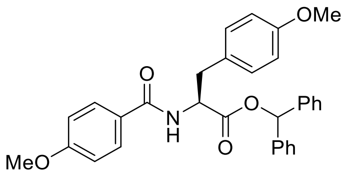

Following **General Procedure A** using α-amino acid **1e** (16.5 mg, 0.05 mmol, 1.0 equiv.) and diphenylmethanol (0.075 mmol, 1.5 equiv.), the crude product was purified by column chromatography petroleum ether/EtOAc (most use 4/1) as eluent to afford the desired product **4e** as a white solid in 90% yield (22.3 mg). **m.p.** 113-114 ℃.
TLC (5:1, PE: EtOAc): R_f = 0.3
^{1}H NMR (400 MHz, $CDCl_3$) δ 7.7 (d, *J* = 8.8 Hz, 2H), 7.4 – 7.3 (m, 10H), 7.0 – 6.9 (m, 3H), 6.8 (d, *J* = 8.6 Hz, 2H), 6.6 (d, *J* = 8.6 Hz, 2H), 6.5 (d, *J* = 7.7 Hz, 1H), 5.2 (ddd, *J* = 7.7, 5.8, 4.8 Hz, 1H), 3.8 (s, 3H), 3.7 (s, 3H), 3.3 – 3.1 (m, 2H).
^{13}C NMR (101 MHz, $CDCl_3$) δ 171.0, 166.3, 162.4, 158.6, 139.4, 139.4, 130.5, 128.8, 128.7, 128.6, 128.3, 128.0, 127.8, 127.5, 126.9, 126.2, 113.9, 113.8, 78.2, 55.4, 55.2, 53.6, 37.0.
HRMS (ESI-TOF) *m/z:* $[M+Na]^+$ Calcd for $C_{31}H_{29}NNaO_5^+$ 518.1938, found 518.1940.
HPLC (Chiralpak-AD-H column, Hexane/^{i}PrOH = 60/40, flow rate: 1.0 mL/min, wavelength = 254 nm) $t_{(major)}$ = 24.833 min; $t_{(minor)}$ = 11.957 min, indicated 96% ee.
$\mathbf{[\alpha]_D^{28}}$ = +19 (c = 0.2 in $CHCl_3$).

benzhydryl (S)-3-(4-fluorophenyl)-2-(4-methoxybenzamido)propanoate **4f**

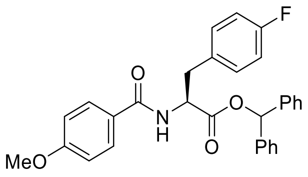

Following **General Procedure A** using α-amino acid **1f** (16 mg, 0.05 mmol, 1.0 equiv.) and diphenylmethanol (0.075 mmol, 1.5 equiv.), the crude product was purified by column chromatography petroleum ether/EtOAc (most use 5/1) as eluent to afford the desired product **4f** as a white solid in 90% yield (21.7 mg), **m.p.** 133-134 °C

TLC (5:1, PE: EtOAc): R_f = 0.3

^{1}H NMR (400 MHz, $CDCl_3$) δ 7.7 (d, *J* = 8.3 Hz, 2H), 7.3 (dd, *J* = 14.3, 3.8 Hz, 10H), 7.0 – 6.9 (m, 3H), 6.8 (dd, *J* = 7.2, 3.7 Hz, 4H), 6.6 (d, *J* = 7.5 Hz, 1H), 5.2 (d, *J* = 6.2 Hz, 1H), 3.8 (s, 3H), 3.2 (qd, *J* = 14.0, 5.5 Hz, 2H).

^{13}C NMR (101 MHz, $CDCl_3$) δ 170.9, 166.3, 162.5, 161.9 (d, *J* = 245.3 Hz), 139.3, 139.3, 131.3 (d, *J* = 3.3 Hz), 131.0 (d, *J* = 8.0 Hz), 128.9, 128.7, 128.6, 128.5, 128.1, 127.9, 126.8, 126.0, 115.3 (d, *J* = 21.2 Hz), 113.9, 78.3, 55.4, 53.5, 37.0.

^{19}F NMR (376 MHz, $CDCl_3$) δ -115.7.

HRMS (ESI-TOF) *m/z:* $[M+Na]^+$ Calcd for $C_{30}H_{26}FNNaO_4^+$ 506.1738, found 506.1740.

HPLC (Chiralpak-OD-3 column, Hexane/^{i}PrOH = 85/15, flow rate: 1.0 mL/min, wavelength = 254 nm) $t_{(major)}$ = 17.447 min; $t_{(minor)}$ = 12.987 min, indicated 90% ee.

$[\alpha]_D^{28}$ = +12.4 (c = 0.2 in $CHCl_3$).

benzhydryl (S)-3-(4-chlorophenyl)-2-(4-methoxybenzamido)propanoate **4g**

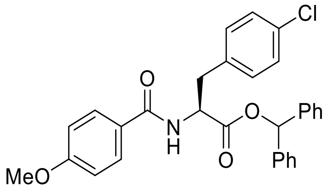

Following **General Procedure A** using α-amino acid **1g** (17 mg, 0.05 mmol, 1.0 equiv.) and diphenylmethanol (0.075 mmol, 1.5 equiv.), the crude product was purified by column chromatography petroleum ether/EtOAc (most use 5/1) as eluent to afford the desired product **4g** as a white solid in 93% yield (23.1 mg), **m.p.** 146-147 °C.

TLC (5:1, PE: EtOAc): R_f = 0.3

^{1}H NMR (400 MHz, $CDCl_3$) δ 7.7 (d, *J* = 8.8 Hz, 2H), 7.5 – 7.3 (m, 10H), 7.1 – 7.0 (m, 2H), 7.0 – 6.9 (m, 3H), 6.8 – 6.7 (m, 2H), 6.6 (d, *J* = 7.4 Hz, 1H), 5.2 (ddd, *J* = 7.6, 6.2, 4.7 Hz, 1H), 3.8 (s, 3H), 3.2 (qd, *J* = 13.9, 5.5 Hz, 2H).

^{13}C NMR (101 MHz, CDCl$_3$) δ 170.8, 166.3, 162.5, 139.2, 139.2, 134.1, 132.9, 130.8, 128.9, 128.8, 128.6, 128.5, 128.1, 127.9, 126.8, 126.0, 113.9, 78.4, 55.4, 53.4, 37.2.
HRMS (ESI-TOF) *m/z:* $[M+Na]^+$ Calcd for $C_{30}H_{26}ClNNaO_4^+$ 522.1443, found 522.1445.
HPLC (Chiralpak-OD-3 column, Hexane/^{i}PrOH = 85/15, flow rate: 1.0 mL/min, wavelength = 254 nm) $t_{(major)}$ = 17.963 min; $t_{(minor)}$ = 13.553 min, indicated 94% ee.
$[\alpha]_D^{28}$ = +59.5 (c = 2 in CHCl$_3$).

benzhydryl (S)-3-(3-chlorophenyl)-2-(4-methoxybenzamido)propanoate **4h**

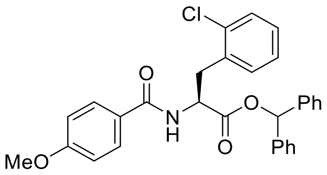

Following **General Procedure A** using α-amino acid **1h** (17 mg, 0.05 mmol, 1.0 equiv.) and diphenylmethanol (0.075 mmol, 1.5 equiv.), the crude product was purified by column chromatography petroleum ether/EtOAc (most use 6/1) as eluent to afford the desired product **4h** as a white solid in 44% yield (11.1 mg), **m.p.** 155-156 °C.
TLC (6:1, PE: EtOAc): R_f = 0.3
^{1}H NMR (400 MHz, CDCl$_3$) δ 7.7 (d, *J* = 8.8 Hz, 2H), 7.4 – 7.3 (m, 10H), 7.1 (ddd, *J* = 7.9, 6.0, 3.0 Hz, 1H), 7.1 – 7.0 (m, 2H), 6.9 (d, *J* = 9.0 Hz, 3H), 6.6 (d, *J* = 8.0 Hz, 1H), 5.2 (td, *J* = 7.6, 6.1 Hz, 1H), 3.8 (s, 3H), 3.5 (dd, *J* = 14.0, 6.1 Hz, 1H), 3.3 (dd, *J* = 14.0, 7.4 Hz, 1H).
^{13}C NMR (101 MHz, CDCl$_3$) δ 171.0, 166.6, 162.4, 139.6, 139.4, 134.5, 134.0, 131.4, 129.6, 128.9, 128.6, 128.6, 128.5, 128.2, 128.1, 127.4, 127.0, 126.9, 126.1, 113.8, 55.4, 53.1, 35.5.
HRMS (ESI-TOF) *m/z:* $[M+Na]^+$ Calcd for $C_{30}H_{26}ClNNaO_4^+$ 522.1443, found 522.1445.
HPLC (Chiralpak-AD-H column, Hexane/^{i}PrOH = 60/40, flow rate: 1.0 mL/min, wavelength = 254 nm) $t_{(major)}$ = 26.380 min; $t_{(minor)}$ = 12.693 min, indicated 94% ee.
$[\alpha]_D^{28}$ = +15.9 (c = 2 in CHCl$_3$).

benzhydryl (S)-3-(4-bromophenyl)-2-(4-methoxybenzamido)propanoate **4i**

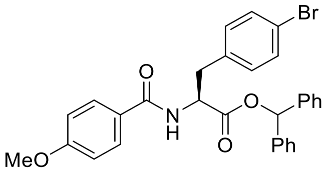

Following **General Procedure A** using α-amino acid **1i** (19 mg, 0.05 mmol, 1.0 equiv.) and diphenylmethanol (0.075 mmol, 1.5 equiv.), the crude product was purified by column chromatography petroleum ether/EtOAc (most use 6/1) as eluent to afford the desired product **4i** as a white solid in 96% yield (26.1 mg), **m.p.** 137-138 °C.

TLC (6:1, PE: EtOAc): R_f = 0.3

^{1}H NMR (400 MHz, $CDCl_3$) δ 7.7 (d, *J* = 8.8 Hz, 2H), 7.5 – 7.3 (m, 10H), 7.2 (d, *J* = 8.3 Hz, 2H), 7.0 – 6.9 (m, 3H), 6.7 (d, *J* = 8.1 Hz, 2H), 6.5 (d, *J* = 7.4 Hz, 1H), 5.2 (td, *J* = 6.6, 4.6 Hz, 1H), 3.8 (s, 3H), 3.3 – 3.1 (m, 2H).

^{13}C NMR (101 MHz, $CDCl_3$) δ 170.7, 166.3, 162.5, 139.2, 134.6, 131.5, 131.2, 128.8, 128.7, 128.6, 128.5, 128.1, 127.9, 126.8, 126.0, 121.0, 113.9, 78.4, 55.4, 53.3, 37.3.

HRMS (ESI-TOF) *m/z:* $[M+K]^+$ Calcd for $C_{30}H_{28}BrNKO_4^+$ 582.0677, found 582.0679.

HPLC (Chiralpak-IC column, Hexane/^{i}PrOH = 90/10, flow rate: 1.0 mL/min, wavelength = 254 nm) $t_{(major)}$ = 32.190 min; $t_{(minor)}$ = 37.570 min, indicated 92% ee.

$[\alpha]_D^{28}$ = +22.4 (c = 1.5 in $CHCl_3$).

benzhydryl (S)-2-(4-methoxybenzamido)-3-(naphthalen-1-yl)propanoate **4j**

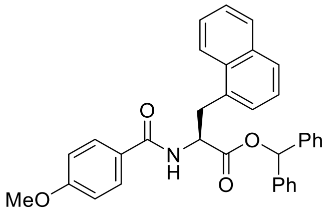

Following **General Procedure A** using α-amino acid **1j** (17.5 mg, 0.05 mmol, 1.0 equiv.) and diphenylmethanol (0.075 mmol, 1.5 equiv.), the crude product was purified by column chromatography petroleum ether/EtOAc (most use 4/1) as eluent to afford the desired product **4j** as a colorless oil in 85% yield (21.9 mg).

TLC (4:1, PE: EtOAc): R_f = 0.2

^{1}H NMR (400 MHz, $CDCl_3$) δ 7.7 (d, *J* = 8.8 Hz, 2H), 8.2 (dd, *J* = 8.4, 1.5 Hz, 1H), 7.8 (dd, *J* = 7.8, 1.6 Hz, 1H), 7.7 (d, *J* = 8.2 Hz, 1H), 7.6 (d, *J* = 8.8 Hz, 2H), 7.4 (ddd, *J* = 16.7, 8.3, 6.8, 1.4 Hz, 2H), 7.3 – 7.3 (m, 6H), 7.2 (dd, *J* = 7.9, 1.9 Hz, 2H), 7.2 (dd, *J* = 8.5, 7.1 Hz, 3H), 7.0 (dd, *J* = 7.1, 1.2 Hz, 1H), 6.9 – 6.8 (m, 3H), 6.5 (d, *J* = 7.7 Hz, 1H), 5.4 (td, *J* = 7.2, 5.9 Hz, 1H), 3.8 (s, 3H), 3.7 (t, *J* = 5.8 Hz, 2H).

^{13}C NMR (101 MHz, $CDCl_3$) δ 171.2, 166.6, 162.4, 139.5, 139.3, 133.8, 132.3, 132.1, 128.9, 128.8, 128.6, 128.5, 128.1, 128.0, 127.9, 127.7, 127.4, 126.9, 126.4, 126.1, 125.7, 125.3, 123.8, 113.7, 78.3, 55.4, 53.7, 35.3.

HRMS (ESI-TOF) *m/z:* $[M+Na]^+$ Calcd for $C_{34}H_{29}NNaO_4^+$ 538.1989, found 538.1992.

HPLC (Chiralpak-OD-3 column, Hexane/^{i}PrOH = 85/15, flow rate: 1.0 mL/min, wavelength = 254 nm) $t_{(major)}$ = 17.083 min; $t_{(minor)}$ = 14.337 min, indicated 97% ee.
$[\alpha]_D^{28}$ = +4.5 (c = 2 in $CHCl_3$).

benzhydryl (S)-2-(4-methoxybenzamido)-3-(thiophen-2-yl)propanoate **4k**

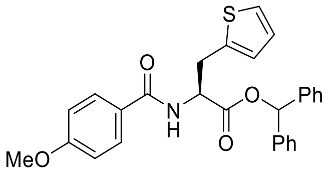

Following **General Procedure A** using α-amino acid **1k** (15.3 mg, 0.05 mmol, 1.0 equiv.) and diphenylmethanol (0.075 mmol, 1.5 equiv.), the crude product was purified by column chromatography petroleum ether/EtOAc (most use 4/1) as eluent to afford the desired product **4k** as a yellow oil in 87% yield (20.4 mg).
TLC (5:1, PE: EtOAc): R_f = 0.2
^{1}H NMR (400 MHz, $CDCl_3$) δ 7.8 – 7.7 (m, 2H), 7.4 – 7.3 (m, 10H), 7.1 (dd, *J* = 5.1, 1.2 Hz, 1H), 7.0 – 6.9 (m, 3H), 6.8 (dd, *J* = 5.2, 3.4 Hz, 1H), 6.7 (d, *J* = 7.5 Hz, 1H), 6.5 (dd, *J* = 3.5, 1.1 Hz, 1H), 5.2 (dt, *J* = 7.5, 4.8 Hz, 1H), 3.8 (s, 3H), 3.5 (d, *J* = 4.9 Hz, 2H).
^{13}C NMR (101 MHz, $CDCl_3$) δ 170.4, 166.4, 162.5, 139.4, 139.3, 136.9, 128.9, 128.7, 128.6, 128.3, 128.1, 127.6, 127.0, 127.0, 126.9, 126.1, 124.9, 113.9, 78.5, 55.4, 53.3, 32.1.
HRMS (ESI-TOF) *m/z:* $[M+Na]^+$ Calcd for $C_{28}H_{25}NSNaO_4^+$ 494.1397, found 494.1397.
HPLC (Chiralpak-IC column, Hexane/^{i}PrOH = 80/20, flow rate: 1.0 mL/min, wavelength = 203 nm) $t_{(major)}$ = 20.988 min; $t_{(minor)}$ = 22.689 min, indicated 94% ee.
$[\alpha]_D^{28}$ = +32.5 (c = 1 in $CHCl_3$).

benzhydryl (S)-2-(2-chlorophenyl)-2-(4-methoxybenzamido)acetate **4l**

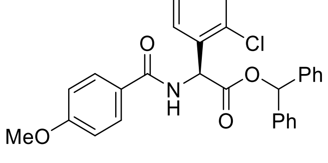

Following **General Procedure A** using α-amino acid **1l** (15 mg, 0.05 mmol, 1.0 equiv.) and diphenylmethanol (0.075 mmol, 1.5 equiv.), the crude product was purified by column chromatography petroleum ether/EtOAc (most use 3/1) as eluent to afford the

desired product **4l** as a white solid in 65% yield (15.7 mg), **m.p.** 148-149 °C.

TLC (4:1, PE: EtOAc): R_f = 0.2

^{1}H NMR (400 MHz, $CDCl_3$) δ 7.8 (d, *J* = 8.9 Hz, 2H), 7.4 (ddd, *J* = 16.3, 7.7, 1.6 Hz, 2H), 7.3 – 7.2 (m, 8H), 7.2 – 7.1 (m, 4H), 7.0 – 7.0 (m, 2H), 6.9 – 6.9 (m, 3H), 6.3 (d, *J* = 6.9 Hz, 1H), 3.8 (s, 3H).

^{13}C NMR (101 MHz, $CDCl_3$) δ 169.5, 166.0, 162.5, 139.4, 139.3, 135.0, 133.8, 130.2, 130.1, 129.7, 129.0, 128.6, 128.3, 128.2, 127.9, 127.3, 127.1, 126.5, 125.8, 113.8, 78.8, 55.4, 55.2.

HRMS (ESI-TOF) *m/z:* $[M+Na]^+$ Calcd for $C_{29}H_{24}ClNNaO_4^+$ 508.1286, found 508.1288.

HPLC (Chiralpak-OD-3 column, Hexane/^{i}PrOH = 70/30, flow rate: 1.0 mL/min, wavelength = 254 nm) $t_{(major)}$ = 13.763 min; $t_{(minor)}$ = 8.883 min, indicated 91% ee.

$[\alpha]_D^{28}$ = +59.5 (c = 2 in $CHCl_3$)

benzhydryl (S)-2-(4-methoxybenzamido)-4-phenylbutanoate **4m**

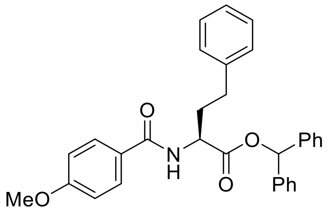

Following **General Procedure A** using α-amino acid **1m** (15.7 mg, 0.05 mmol, 1.0 equiv.) and diphenylmethanol (0.075 mmol, 1.5 equiv.), the crude product was purified by column chromatography petroleum ether/EtOAc (most use 5/1) as eluent to afford the desired product **4m** as a colorless oil in 85% yield (20.5 mg).

TLC (5:1, PE: EtOAc): R_f = 0.4

^{1}H NMR (400 MHz, $CDCl_3$) δ 7.7 (d, *J* = 8.9 Hz, 2H), 7.4 – 7.3 (m, 10H), 7.2 (d, *J* = 6.1 Hz, 1H), 7.2 – 7.1 (m, 1H), 7.1 – 7.0 (m, 2H), 7.0 (s, 1H), 6.9 – 6.9 (m, 2H), 6.7 (d, *J* = 7.7 Hz, 1H), 5.0 (ddd, *J* = 7.6, 6.4, 5.0 Hz, 1H), 3.8 (s, 3H), 2.8 – 2.5 (m, 2H), 2.4 (ddd, *J* = 13.8, 9.9, 6.6, 5.0 Hz, 1H), 2.2 – 2.1 (m, 1H).

^{13}C NMR (101 MHz, $CDCl_3$) δ 171.7, 166.5, 162.4, 140.8, 139.6, 139.3, 128.9, 128.7, 128.6, 128.5, 128.4, 128.3, 128.2, 127.4, 127.0, 126.2, 126.1, 113.8, 78.3, 55.4, 52.7, 34.1, 31.4.

HRMS (ESI-TOF) *m/z:* $[M+Na]^+$ Calcd for $C_{31}H_{29}NNaO_4^+$ 502.1989, found 502.1988.

HPLC (Chiralpak-IC column, Hexane/^{i}PrOH = 70/30, flow rate: 1.0 mL/min, wavelength = 254 nm) $t_{(major)}$ = 15.510 min; $t_{(minor)}$ = 13.250 min, indicated 92% ee.

$[\alpha]_D^{28}$ = +54.5 (c = 1 in $CHCl_3$).

benzhydryl (S)-4-cyclohexyl-2-(4-methoxybenzamido)butanoate **4n**

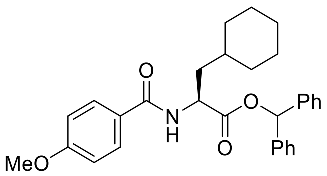

Following **General Procedure A** using α-amino acid **1n** (15.3 mg, 0.05 mmol, 1.0 equiv.) and diphenylmethanol (0.075 mmol, 1.5 equiv.), the crude product was purified by column chromatography petroleum ether/DCM (most use 2/1) as eluent to afford the desired product **4n** as a white solid in 79% yield (18.6 mg). **m.p.** 77-78 °C.

TLC (2:1, PE: DCM): R_f = 0.4

^{1}H NMR (400 MHz, $CDCl_3$) δ 7.7 (d, *J* = 8.8 Hz, 2H), 7.4 – 7.3 (m, 10H), 6.9 – 6.9 (m, 3H), 6.5 (d, *J* = 8.1 Hz, 1H), 5.0 (td, *J* = 8.1, 5.9 Hz, 1H), 3.8 (s, 3H), 1.8 – 1.8 (m, 2H), 1.7 – 1.6 (m, 4H), 1.6 – 1.6 (m, 1H), 1.4 – 1.3 (m, 2H), 1.1 (d, *J* = 7.7 Hz, 2H), 1.0 – 0.9 (m, 2H).

^{13}C NMR (101 MHz, $CDCl_3$) δ 172.5, 166.5, 162.4, 139.7, 139.5, 128.9, 128.6, 128.5, 128.2, 128.0, 127.4, 127.0, 126.4, 113.8, 78.0, 55.4, 50.8, 40.5, 34.2, 33.4, 32.9, 26.3, 26.0.

HRMS (ESI-TOF) *m/z:* $[M+Na]^+$ Calcd for $C_{30}H_{33}NNaO_4^+$ 494.2302, found 494.2304.

HPLC (Chiralpak-IA column, Hexane/^{i}PrOH = 80/20, flow rate: 1.0 mL/min, wavelength = 250 nm) $t_{(major)}$ = 17.254 min; $t_{(minor)}$ = 14.174 min, indicated 93% ee.

$[\alpha]_D^{28}$ = +14.5 (c = 0.2 in $CHCl_3$).

benzhydryl (S)-2-(4-methoxybenzamido)pentanoate **4o**

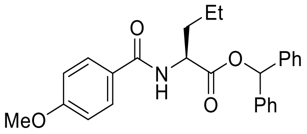

Following **General Procedure A** using α-amino acid **1o** (13 mg, 0.05 mmol, 1.0 equiv.) and diphenylmethanol (0.075 mmol, 1.5 equiv.), the crude product was purified by column chromatography petroleum ether/EtOAc (most use 4/1) as eluent to afford the desired product **4o** as a colorless oil in 91% yield (18.9 mg).

TLC (5:1, PE: EtOAc): R_f = 0.3

^{1}H NMR (400 MHz, $CDCl_3$) δ 7.7 (d, *J* = 8.8 Hz, 2H), 7.4 – 7.3 (m, 10H), 6.9 – 6.9 (m, 2H), 6.6 (d, *J* = 7.8 Hz, 1H), 5.0 (td, *J* = 7.2, 5.2 Hz, 1H), 3.8 (s, 3H), 2.0 (ddt, *J* =

13.7, 10.8, 5.5 Hz, 1H), 1.8 (ddd, J = 13.7, 10.5, 7.0, 5.1 Hz, 1H), 1.4 (dddd, J = 13.3, 10.6, 7.4, 5.9 Hz, 1H), 1.3 – 1.2 (m, 1H), 0.9 (t, J = 7.3 Hz, 3H).

^{13}C NMR (101 MHz, $CDCl_3$) δ 172.1, 166.5, 162.4, 139.6, 139.4, 128.9, 128.6, 128.2, 128.1, 127.2, 127.0, 126.3, 113.8, 78.1, 55.4, 52.5, 34.7, 18.4, 13.7.

HRMS (ESI-TOF) *m/z:* $[M+Na]^+$ Calcd for $C_{26}H_{27}NNaO_4^+$ 440.1832, found 440.1833.

HPLC (Chiralpak-IC column, Hexane/^{i}PrOH = 80/20, flow rate: 1.0 mL/min, wavelength = 254 nm) $t_{(major)}$ = 20.527 min; $t_{(minor)}$ = 18.110 min, indicated 97% ee.

$[\alpha]_D^{28}$ = +46.7 (c = 0.3 in $CHCl_3$)

benzhydryl (S)-2-(4-methoxybenzamido)hexanoate **4p**

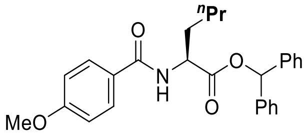

Following **General Procedure A** using α-amino acid **1p** (13.3 mg, 0.05 mmol, 1.0 equiv.) and diphenylmethanol (0.075 mmol, 1.5 equiv.), the crude product was purified by column chromatography petroleum ether/EtOAc (most use 4/1) as eluent to afford the desired product **4p** as a colorless oil in 92% yield (19.7 mg).

TLC (5:1, PE: EtOAc): R_f = 0.25

^{1}H NMR (400 MHz, $CDCl_3$) δ 7.8 – 7.7 (m, 2H), 7.4 – 7.3 (m, 10H), 6.9 (d, J = 8.6 Hz, 3H), 6.6 (d, J = 7.8 Hz, 1H), 5.0 – 4.9 (m, 1H), 3.8 (s, 3H), 2.0 (dq, J = 13.7, 5.4 Hz, 1H), 1.8 (ddt, J = 12.2, 6.0, 3.1 Hz, 1H), 1.6 (s, 1H), 1.3 – 1.3 (m, 2H), 1.2 – 1.1 (m, 1H), 0.8 (t, J = 7.1 Hz, 3H).

^{13}C NMR (101 MHz, $CDCl_3$) δ 172.1, 166.5, 162.4, 139.7, 139.4, 128.9, 128.6, 128.2, 128.1, 127.3, 127.0, 126.3, 113.8, 78.1, 55.4, 52.6, 32.4, 27.0, 22.4, 13.8.

HRMS (ESI-TOF) *m/z:* $[M+Na]^+$ Calcd for $C_{27}H_{29}NNaO_4^+$ 454.1989, found 454.1988.

HPLC (Chiralpak-IC column, Hexane/^{i}PrOH = 80/20, flow rate: 1.0 mL/min, wavelength = 254 nm) $t_{(major)}$ =20.790 min; $t_{(minor)}$ = 19.003 min, indicated 95% ee.

$[\alpha]_D^{28}$ = -3.3 (c = 0.1 in $CHCl_3$).

benzhydryl (S)-2-(4-methoxybenzamido)pent-4-enoate **4q**

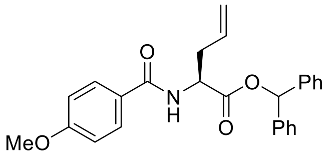

Following **General Procedure A** using α-amino acid **1q** (12.5 mg, 0.05 mmol, 1.0 equiv.) and diphenylmethanol (0.075 mmol, 1.5 equiv.), the crude product was purified

by column chromatography petroleum ether/EtOAc (most use 5/1) as eluent to afford the desired product **4q** as a colorless oil in 87% yield (18.1 mg).

TLC (5:1, PE: EtOAc): R_f = 0.3

^{1}H NMR (400 MHz, CDCl$_3$) δ 7.7 (d, *J* = 8.8 Hz, 2H), 7.4 – 7.3 (m, 10H), 7.0 – 6.8 (m, 3H), 6.6 (d, *J* = 7.7 Hz, 1H), 5.7 – 5.5 (m, 1H), 5.1 – 4.9 (m, 3H), 3.8 (s, 3H), 2.8 – 2.6 (m, 2H).

^{13}C NMR (101 MHz, CDCl$_3$) δ 171.2, 166.4, 162.4, 139.5, 139.4, 131.9, 128.9, 128.6, 128.3, 128.1, 127.3, 127.0, 126.2, 119.6, 113.8, 78.3, 55.4, 52.0, 36.6.

HRMS (ESI-TOF) *m/z:* [M+Na]$^+$ Calcd for $C_{26}H_{25}NNaO_4^+$ 438.1676, found 438.1674.

HPLC (Chiralpak-IC column, Hexane/^{i}PrOH = 70/30, flow rate: 1.0 mL/min, wavelength = 254 nm) $t_{(major)}$ = 13.713 min; $t_{(minor)}$ = 12.070 min, indicated 93% ee.

$[\alpha]_D^{28}$ = +39.5 (c = 1 in $CHCl_3$).

benzhydryl (S)-2-(4-methoxybenzamido)pent-4-ynoate **4r**

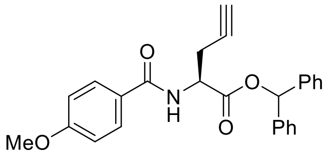

Following **General Procedure A** using α-amino acid **1r** (12.5 mg, 0.05 mmol, 1.0 equiv.) and diphenylmethanol (0.075 mmol, 1.5 equiv.), the crude product was purified by column chromatography petroleum ether/EtOAc (most use 4/1) as eluent to afford the desired product **4r** as a white solid in 72% yield (14.9 mg). **m.p.** 59-60 °C.

TLC (4:1, PE: EtOAc): R_f = 0.3

^{1}H NMR (400 MHz, CDCl$_3$) δ 8.1 (d, *J* = 9.1 Hz, 2H), 7.8 (d, *J* = 8.9 Hz, 2H), 7.4 – 7.3 (m, 10H), 6.9 (s, 1H), 6.9 (s, 1H), 6.9 (s, 1H), 5.1 – 5.0 (m, 1H), 3.9 (s, 3H), 3.0 (dd, *J* = 4.8, 2.6 Hz, 2H).

^{13}C NMR (101 MHz, CDCl$_3$) δ 169.8, 167.1, 162.8, 139.2, 129.0, 128.7, 128.6, 128.3, 127.2, 126.2, 125.6, 115.7, 114.0, 79.0, 78.2, 72.1, 55.5, 51.3, 22.6.

HRMS (ESI-TOF) *m/z:* [M+Na]$^+$ Calcd for $C_{26}H_{23}NNaO_4^+$ 436.1519, found 436.1520.

HPLC (Chiralpak-IA column, Hexane/^{i}PrOH = 80/20, flow rate: 1.0 mL/min, wavelength = 250 nm) $t_{(major)}$ = 14.605 min; $t_{(minor)}$ = 11.421 min, indicated 93% ee.

$[\alpha]_D^{28}$ = +5.0 (c = 0.5 in $CHCl_3$).

benzhydryl (S)-2-cyclohexyl-2-(4-methoxybenzamido)acetate **4s**

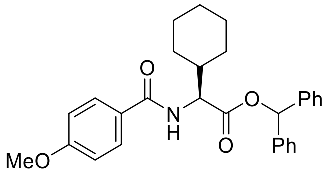

Following **General Procedure A** using α-amino acid **1s** (14.6 mg, 0.05 mmol, 1.0 equiv.), diphenylmethanol (0.075 mmol, 1.5 equiv.) and **NHC-3s** (0.01 mmol, 4.5 mg), the crude product was purified by column chromatography petroleum ether/EtOAc (most use 6/1) as eluent to afford the desired product **4s** as a colorless oil in 82% yield (18.8 mg).

TLC (5:1, PE: EtOAc): R_f = 0.4

^{1}H NMR (400 MHz, CDCl$_3$) δ 7.8 (d, *J* = 8.8 Hz, 2H), 7.5 – 7.2 (m, 10H), 7.0 – 6.8 (m, 3H), 6.6 (dd, *J* = 8.6, 2.0 Hz, 1H), 4.9 (dd, *J* = 8.6, 4.7 Hz, 1H), 3.8 (s, 3H), 2.0 (dt, *J* = 7.7, 4.1 Hz, 1H), 1.8 – 1.6 (m, 4H), 1.5 (d, *J* = 11.3 Hz, 1H), 1.3 – 1.0 (m, 5H).

^{13}C NMR (101 MHz, CDCl$_3$) δ 171.6, 166.7, 162.4, 139.7, 139.4, 128.9, 128.6, 128.5, 128.3, 128.1, 127.5, 127.0, 126.4, 113.8, 78.1, 57.0, 55.4, 41.6, 29.5, 28.0, 26.0.

HRMS (ESI-TOF) *m/z:* $[M+Na]^+$ Calcd for $C_{29}H_{31}NNaO_4^+$ 480.2145, found 480.2146.

HPLC (Chiralpak-OD-3 column, Hexane/^{i}PrOH = 70/30, flow rate: 1.0 mL/min, wavelength = 254 nm) $t_{(major)}$ = 19.353 min; $t_{(minor)}$ = 11.587 min, indicated 90% ee.

$[\alpha]_D^{28}$ = +33 (c = 0.76 in $CHCl_3$)

benzhydryl benzoyl-L-phenylalaninate **4aa**

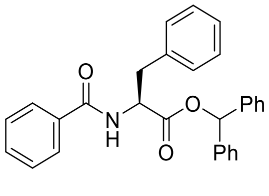

Following **General Procedure A** using α-amino acid **1aa** (13.5 mg, 0.05 mmol, 1.0 equiv.) and diphenylmethanol (0.075 mmol, 1.5 equiv.), the crude product was purified by column chromatography petroleum ether/EtOAc (most use 4/1) as eluent to afford the desired product **4aa** as a white solid in 93% yield (20.2 mg). **m.p.** 103-104 °C.

TLC (5:1, PE: EtOAc): R_f = 0.3

^{1}H NMR (400 MHz, CDCl$_3$) δ 7.8 – 7.7 (m, 2H), 7.5 (t, *J* = 7.4 Hz, 1H), 7.5 – 7.3 (m, 12H), 7.2 – 7.1 (m, 3H), 7.1 – 6.9 (m, 3H), 6.7 (d, *J* = 7.7 Hz, 1H), 5.3 (dt, *J* = 7.7, 5.6 Hz, 1H), 3.3 (qd, *J* = 13.9, 5.6 Hz, 2H).

^{13}C NMR (101 MHz, CDCl$_3$) δ 170.9, 167.0, 139.5, 139.5, 135.6, 134.0, 131.8, 129.5, 128.7, 128.7, 128.6, 128.6, 128.4, 128.1, 127.8, 127.1, 127.1, 127.0, 78.4, 53.6, 37.8.

HRMS (ESI-TOF) *m/z:* $[M+Na]^+$ Calcd for $C_{29}H_{25}NNaO_3^+$ 458.1727, found 458.1728.
HPLC (Chiralpak-OD-3 column, Hexane/^{i}PrOH = 70/30, flow rate: 1.0 mL/min, wavelength = 254 nm) $t_{(major)}$ = 11.213 min; $t_{(minor)}$ = 8.453 min, indicated 99% ee.
$[\alpha]_D^{28}$ = +59 (c = 2 in $CHCl_3$).

benzhydryl acetyl-L-phenylalaninate **4ab**

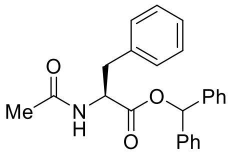

Following **General Procedure A** using α-amino acid **1ab** (10.5 mg, 0.05 mmol, 1.0 equiv.) and diphenylmethanol (0.075 mmol, 1.5 equiv.), the crude product was purified by column chromatography petroleum ether/EtOAc (most use 3/1) as eluent to afford the desired product **4ab** as a white solid in 80% yield (15.0 mg). **m.p.** 69-70 °C.
R-4ab m.p. 92-93 °C.(eti-)
TLC (3:1, PE: EtOAc): R_f = 0.25
^{1}H NMR (400 MHz, CDCl$_3$) δ 7.4 – 7.3 (m, 10H), 7.2 – 7.1 (m, 3H), 7.0 – 6.8 (m, 3H), 6.0 (d, *J* = 8.0 Hz, 1H), 5.0 (dt, *J* = 8.0, 5.8 Hz, 1H), 3.2 – 3.1 (m, 2H), 2.0 (s, 3H).
^{13}C NMR (101 MHz, CDCl$_3$) δ 170.9, 169.7, 139.4, 139.4, 135.5, 129.4, 128.7, 128.6, 128.5, 128.3, 128.1, 127.7, 127.0, 126.9, 78.2, 53.1, 37.7, 23.1.
HRMS (ESI-TOF) *m/z:* $[M+Na]^+$ Calcd for $C_{24}H_{23}NNaO_3^+$ 396.1570, found 396.1570.
HPLC (Chiralpak-IC column, Hexane/^{i}PrOH = 80/20, flow rate: 1.0 mL/min, wavelength = 250 nm) $t_{(major)}$ = 12.239 min; $t_{(minor)}$ = 11.472 min, indicated 99% ee.
$[\alpha]_D^{28}$ = +33 (c = 1.5 in $CHCl_3$).

benzhydryl (furan-2-carbonyl)-L-phenylalaninate **4ac**

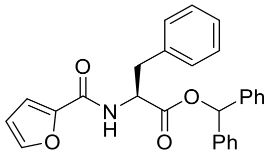

Following the **General Procedure A** using α-amino acid **1ac** (13 mg, 0.05 mmol, 1.0 equiv.) and diphenylmethanol (0.075 mmol, 1.5 equiv.), the crude product was purified by column chromatography petroleum ether/EtOAc (most use 4/1) as eluent to afford the desired product **4ac** as a yellow solid in 82% yield (17.5 mg), **m.p.** 81-82 °C.
TLC (4:1, PE: EtOAc): R_f = 0.4

^{1}H NMR (400 MHz, CDCl$_3$) δ 7.4 (s, 1H), 7.4 – 7.3 (m, 10H), 7.2 – 7.1 (m, 4H), 7.0 – 6.9 (m, 3H), 6.8 (d, *J* = 8.1 Hz, 1H), 6.5 (dd, *J* = 3.5, 1.8 Hz, 1H), 5.3 – 5.1 (m, 1H), 3.2 (dd, *J* = 5.8, 2.6 Hz, 2H).
^{13}C NMR (101 MHz, CDCl$_3$) δ 170.5, 157.8, 147.4, 144.3, 139.4, 139.3, 135.3, 129.4, 128.6, 128.6, 128.3, 128.1, 127.7, 127.1, 126.9, 114.8, 112.1, 78.3, 52.8, 38.0.
HRMS (ESI-TOF) *m/z:* $[M+Na]^+$ Calcd for $C_{27}H_{23}NNaO_4^+$ 448.1519, found 448.1512.
HPLC (Chiralpak-OD-3 column, Hexane/^{i}PrOH = 70/30, flow rate: 1.0 mL/min, wavelength = 254 nm) $t_{(major)}$ = 9.453 min; $t_{(minor)}$ = 7.810 min, indicated 99% ee.
$[\alpha]_D^{28}$ = +34.5 (c = 1 in $CHCl_3$).

benzhydryl cinnamoyl-L-phenylalaninate **4ad**

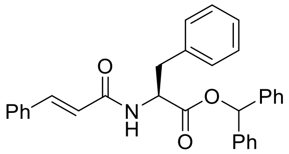

Following **General Procedure A** using α-amino acid **1ad** (15 mg, 0.05 mmol, 1.0 equiv.) and diphenylmethanol (0.075 mmol, 1.5 equiv.), the crude product was purified by column chromatography petroleum ether/EtOAc (most use 6/1) as eluent to afford the desired product **4ad** as a colorless oil in 74% yield (17.2 mg).
TLC (6:1, PE: EtOAc): R_f = 0.4
^{1}H NMR (400 MHz, CDCl$_3$) δ 7.6 (d, *J* = 15.6 Hz, 1H), 7.5 (dd, *J* = 6.6, 3.0 Hz, 2H), 7.4 – 7.3 (m, 13H), 7.2 (qd, *J* = 8.7, 7.8, 3.7 Hz, 3H), 7.0 – 6.8 (m, 3H), 6.4 (d, *J* = 15.6 Hz, 1H), 6.2 (d, *J* = 7.9 Hz, 1H), 5.2 (dt, *J* = 7.9, 5.6 Hz, 1H), 3.3 – 3.1 (m, 2H).
^{13}C NMR (101 MHz, CDCl$_3$) δ 170.8, 165.3, 141.8, 139.4, 135.5, 134.6, 129.9, 129.5, 128.8, 128.7, 128.6, 128.5, 128.4, 128.1, 127.9, 127.8, 127.0, 126.9, 126.6, 120.0, 78.3, 53.3, 37.8.
HRMS (ESI-TOF) *m/z:* $[M+H]^+$ Calcd for $C_{31}H_{28}NO_3^+$ 462.2064, found 462.2079.
HPLC (Chiralpak-OD-3 column, Hexane/^{i}PrOH = 70/30, flow rate: 1.0 mL/min, wavelength = 254 nm) $t_{(major)}$ = 15.163 min; $t_{(minor)}$ = 9.487 min, indicated 98% ee.
$[\alpha]_D^{28}$ = +14.7 (c = 1 in $CHCl_3$).

benzhydryl (cyclohexanecarbonyl)-L-phenylalaninate **4ae**

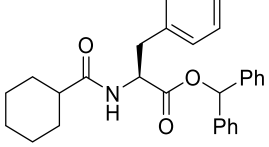

Following **General Procedure A** using α-amino acid **1ae** (14 mg, 0.05 mmol, 1.0 equiv.) and diphenylmethanol (0.075 mmol, 1.5 equiv.), the crude product was purified by column chromatography petroleum ether/DCM (most use 3/1) as eluent to afford the desired product **4ae** as a colorless oil in 96% yield (21.2 mg).

TLC (3:1, PE: DCM): R_f = 0.4

^{1}H NMR (400 MHz, CDCl$_3$) δ 7.4 – 7.3 (m, 10H), 7.2 (dtd, *J* = 12.2, 6.6, 5.9, 2.4 Hz, 3H), 6.9 (s, 1H), 6.9 – 6.8 (m, 2H), 5.9 – 5.8 (m, 1H), 5.0 (dt, *J* = 8.0, 5.7 Hz, 1H), 3.1 (qd, *J* = 13.9, 5.6 Hz, 2H), 2.1 (tt, *J* = 11.8, 3.4 Hz, 1H), 1.8 – 1.7 (m, 2H), 1.7 – 1.6 (m, 1H), 1.4 (ddd, *J* = 17.1, 8.8, 2.9 Hz, 2H), 1.3 – 1.1 (m, 4H).

^{13}C NMR (101 MHz, CDCl$_3$) δ 175.5, 170.9, 143.9, 139.4, 135.6, 129.5, 128.7, 128.5, 128.5, 128.4, 127.7, 126.9, 126.6, 78.2, 76.3, 52.6, 45.3, 37.7, 29.6, 29.3, 25.7, 25.7, 25.6.

HRMS (ESI-TOF) *m/z:* $[M+Na]^+$ Calcd for $C_{29}H_{31}NNaO_3^+$ 464.2196, found 464.2198.

HPLC (Chiralpak-IC column, Hexane/^{i}PrOH = 90/10, flow rate: 1.0 mL/min, wavelength = 223 nm) $t_{(major)}$ = 14.706 min; $t_{(minor)}$ = 13.004 min, indicated 88% ee.

$[\alpha]_D^{28}$ = +22 (c = 0.8 in $CHCl_3$).

benzhydryl (diphenylcarbamoyl)-L-phenylalaninate **4af**

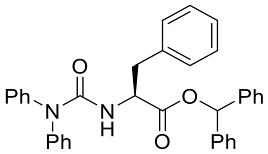

Following the **General Procedure A** using α-amino acid **1af** (18 mg, 0.05 mmol, 1.0 equiv.) and diphenylmethanol (0.075 mmol, 1.5 equiv.), the crude product was purified by column chromatography petroleum ether/EtOAc (most use 3/1) as eluent to afford the desired product **4af** as a white solid in 90% yield (23.7 mg). **m.p.** 111-113 °C.

TLC (4:1, PE: EtOAc): R_f = 0.2

^{1}H NMR (400 MHz, CDCl$_3$) δ 7.4 – 7.1 (m, 26H), 6.9 (s, 1H), 6.9 – 6.8 (m, 2H), 5.0 – 4.9 (m, 2H), 3.2 – 3.0 (m, 2H).

^{13}C NMR (101 MHz, CDCl$_3$) δ 171.1, 155.2, 142.3, 139.6, 139.5, 135.7, 129.3, 129.2, 128.6, 128.5, 128.2, 128.0, 127.6, 127.3, 127.0, 126.9, 126.3, 78.0, 54.3, 37.7.

HRMS (ESI-TOF) *m/z:* $[M+Na]^+$ Calcd for $C_{35}H_{30}N_2NaO_3^+$ 549.2149, found 549.2151.

HPLC (Chiralpak-IC column, Hexane/^{i}PrOH = 80/20, flow rate: 1.0 mL/min, wavelength = 239 nm) $t_{(major)}$ = 34.780 min; $t_{(minor)}$ = 24.218 min, indicated 97% ee.

$[\alpha]_D^{28}$ = +49.5 (c = 1 in $CHCl_3$).

methyl acetyl-L-phenylalaninate **4ag**

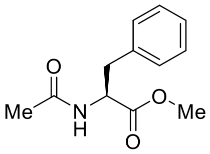

Following **General Procedure A** using α-amino acid **1ab** (11 mg, 0.05 mmol, 1.0 equiv.) and methanol (4 µL, 0.1 mmol, 2 equiv.), the crude product was purified by column chromatography petroleum ether/EtOAc (most use 2/1) as eluent to afford the desired product **4ag** as a white solid in 90% yield (10 mg).

TLC (2:1, PE: EtOAc): R_f = 0.2

^{1}H NMR (400 MHz, CDCl$_3$) δ 7.4 – 7.2 (m, 4H), 7.2 – 7.0 (m, 2H), 4.9 (dt, *J* = 7.9, 5.8 Hz, 1H), 3.7 (s, 3H), 3.2 – 3.0 (m, 2H), 2.0 (s, 3H).

^{13}C NMR (101 MHz, CDCl$_3$) δ 172.1, 169.7, 157.0, 135.8, 129.2, 128.6, 127.2, 53.1, 52.3, 42.1, 37.8.

HRMS (ESI-TOF) *m/z:* $[M+K]^+$ Calcd for $C_{12}H_{15}NKO_3^+$ 260.0684, found 260.0686.

HPLC (Chiralpak-IC column, Hexane/^{i}PrOH = 80/20, flow rate: 1.0 mL/min, wavelength = 250 nm) $t_{(major)}$ = 13.502 min; $t_{(minor)}$ = 15.348 min, indicated 88% ee.

$[\alpha]_D^{28}$ = +8.4 (c = 0.5 in $CHCl_3$).

2,4,6-trimethylbenzyl (4-methoxybenzoyl)-L-phenylalaninate **4ah**

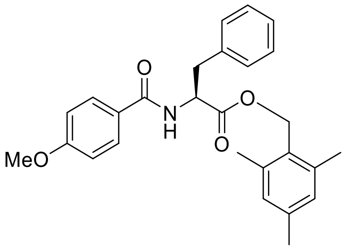

Following **General Procedure A** using α-amino acid **1a** (15 mg, 0.05 mmol, 1.0 equiv.) and mesityl methanol (0.075 mmol, 1.5 equiv.), the crude product was purified by column chromatography petroleum ether/EtOAc (most use 4/1) as eluent to afford the desired product **4ah** as a white solid in 94% yield (20.1 mg). **m.p.** 129-130 °C.

TLC (5:1, PE: EtOAc): R_f = 0.4

^{1}H NMR (400 MHz, CDCl$_3$) δ 7.7 (d, *J* = 8.9 Hz, 2H), 7.2 – 7.2 (m, 3H), 7.0 – 6.9 (m, 2H), 6.9 – 6.8 (m, 4H), 6.5 (d, *J* = 7.5 Hz, 1H), 5.3 – 5.2 (m, 2H), 5.1 (dt, *J* = 7.6, 5.4 Hz, 1H), 3.8 (s, 3H), 3.3 – 3.1 (m, 2H), 2.4 – 2.3 (m, 9H).

^{13}C NMR (101 MHz, CDCl$_3$) δ 171.8, 166.3, 162.4, 138.8, 138.4, 135.8, 129.4, 129.2, 128.9, 128.5, 128.4, 127.1, 126.2, 113.8, 62.1, 55.4, 53.5, 37.9, 21.1, 19.6.

HRMS (ESI-TOF) *m/z:* $[M+Na]^+$ Calcd for $C_{27}H_{39}NNaO_4^+$ 454.1989, found 454.1990.

HPLC (Chiralpak-IC column, Hexane/^{i}PrOH = 80/20, flow rate: 1.0 mL/min, wavelength = 260 nm) $t_{(major)}$ = 15.812 min; $t_{(minor)}$ = 16.942 min, indicated 97% ee.

$[\alpha]_D^{28}$ = +45.7 (c = 1 in $CHCl_3$).

but-3-en-1-yl benzoyl-L-phenylalaninate **4ai**

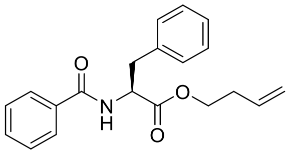

Following **General Procedure A** using α-amino acid **1aa** (14 mg, 0.05 mmol, 1.0 equiv.) and but-3-en-1-ol (0.075 mmol, 1.5 equiv.), the crude product was purified by column chromatography petroleum ether/DCM (most use 4/1) as eluent to afford the desired product **4ai** as a white solid in 91% yield (14.8 mg). **m.p.** 70-71℃.

TLC (3:1, PE: DCM): R_f = 0.5

^{1}H NMR (400 MHz, CDCl$_3$) δ 7.8 – 7.7 (m, 2H), 7.5 – 7.5 (m, 1H), 7.4 (dd, *J* = 8.3, 6.8 Hz, 2H), 7.3 (td, *J* = 10.7, 9.7, 6.0 Hz, 3H), 7.2 – 7.1 (m, 2H), 6.6 (d, *J* = 7.7 Hz, 1H), 5.7 (ddt, *J* = 17.0, 10.3, 6.7 Hz, 1H), 5.2 – 5.0 (m, 3H), 4.3 – 4.1 (m, 2H), 3.3 (qd, *J* = 13.9, 5.7 Hz, 2H), 2.5 – 2.3 (m, 2H).

^{13}C NMR (101 MHz, CDCl$_3$) δ 171.6, 166.8, 135.9, 133.9, 133.6, 131.8, 129.4, 128.6, 128.6, 127.2, 127.0, 117.7, 64.6, 53.6, 38.0, 32.9.

HRMS (ESI-TOF) *m/z:* $[M+Na]^+$ Calcd for $C_{20}H_{21}NNaO_3^+$ 346.1414, found 346.1413.

HPLC (Chiralpak-IB column, Hexane/^{i}PrOH = 95/5, flow rate: 1.0 mL/min, wavelength = 250 nm) $t_{(major)}$ = 10.838 min; $t_{(minor)}$ = 9.851 min, indicated 90% ee.

$[\alpha]_D^{28}$ = +23.5 (c = 0.5 in $CHCl_3$).

1-((9H-fluoren-9-yl)methyl) 2-methyl (2S,4R)-4-((benzoyl-L-phenylalanyl)oxy)pyrrolidine-1,2-dicarboxylate **4aj**

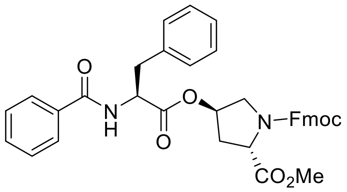

Following the **General Procedure A** using α-amino acid **1aa** (14 mg, 0.05 mmol, 1.0 equiv.) and 1-((9H-fluoren-9-yl)methyl) 2-methyl (2S,4R)-4-hydroxypyrrolidine-1,2-dicarboxylate (22 mg, 0.06 mmol, 1.2 equiv.), the crude product was purified by column chromatography DCM/Acetone (most use 20/1) as eluent to afford the desired product **4aj** as a white solid in 90% yield (28.1 mg). **m.p.** 136-137 ℃

TLC (20:1, DCM: Acetone): R_f = 0.4

^{1}H NMR (400 MHz, $CDCl_3$) δ 7.8 – 7.7 (m, 4H), 7.6 – 7.5 (m, 3H), 7.5 – 7.4 (m, 4H), 7.4 – 7.3 (m, 3H), 7.2 – 7.2 (m, 1H), 7.1 (dd, *J* = 7.2, 5.3 Hz, 2H), 6.6 (dd, *J* = 7.4, 3.3 Hz, 1H), 5.0 (q, *J* = 6.6 Hz, 1H), 4.5 (ddd, *J* = 10.2, 6.3, 3.4 Hz, 1H), 4.4 – 4.3 (m, 3H), 4.1 (dt, *J* = 13.9, 6.9 Hz, 1H), 3.8 – 3.6 (m, 4H), 3.3 – 3.1 (m, 2H), 2.5 – 2.4 (m, 1H), 2.2 (dtd, *J* = 14.0, 4.9, 2.4 Hz, 1H).

^{13}C NMR (101 MHz, $CDCl_3$) δ 172.4, 171.3, 167.1, 154.5, 141.3, 135.6, 133.6, 132.0, 129.2, 128.8, 128.8, 128.7, 127.8, 127.4, 127.0, 125.0, 120.0, 73.8, 73.1, 67.8, 57.8, 57.3, 53.7, 52.5, 47.2, 38.0, 36.7, 35.5.

HRMS (ESI-TOF) *m/z:* $[M+Na]^+$ Calcd for $C_{37}H_{34}N_2NaO_7^+$ 641.2258, found 641.2261.

HPLC (Chiralpak-IA column, Hexane/^{i}PrOH = 70/30, flow rate: 1.0 mL/min, wavelength = 214 nm) $t_{(major)}$ = 21.931 min; $t_{(minor)}$ = 28.063 min, indicated 88% de.

$[\alpha]_D^{28}$ = +47 (c = 1 in $CHCl_3$).

(S)-2-((((9H-fluoren-9-yl)methoxy)carbonyl)amino)-3-phenylpropyl acetyl-L-phenylalaninate **4ak**

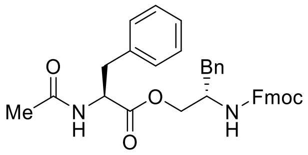

Following the **General Procedure A** using α-amino acid **1ab** (11 mg, 0.05 mmol, 1.0 equiv.), Fmoc-phenylalaninol (24 mg, 0.06 mmol, 1.2 equiv.) and NHC-**3c** (5 mg, 0.01 mmol, 20% mmol) the crude product was purified by column chromatography PE/EA (most use 1/2) as eluent to afford the desired product **4ak** as a white solid in 67% yield (18.8 mg). **m.p.** 173-174 ℃

TLC (1:1, PE: EA): R_f = 0.2

^{1}H NMR (400 MHz, Acetone-*d*$_6$) δ 7.9 – 7.8 (m, 2H), 7.6 (t, *J* = 7.2 Hz, 2H), 7.5 – 7.4 (m, 3H), 7.3 – 7.2 (m, 12H), 6.6 (dd, *J* = 35.1, 8.6 Hz, 1H), 4.7 (pd, *J* = 8.1, 6.9, 4.1 Hz, 1H), 4.3 – 4.1 (m, 4H), 4.1 – 4.0 (m, 1H), 3.2 – 2.9 (m, 4H), 1.9 (d, *J* = 3.6 Hz, 3H).

^{13}C NMR (101 MHz, Acetone-*d*$_6$) δ 205.5, 205.1, 171.5, 171.4, 156.9, 144.2, 141.2, 129.3, 129.2, 128.3, 127.6, 127.0, 126.6, 126.3, 125.3, 119.9, 66.0, 65.5, 54.1, 54.0, 51.8, 47.2, 37.2, 22.7, 21.8.

HRMS (ESI-TOF) *m/z:* $[M+Na]^+$ Calcd for $C_{35}H_{34}N_2NaO_5^+$ 585.2360, found 585.2364.

HPLC (Chiralpak-IA column, Hexane/^{i}PrOH = 80/20, flow rate: 1.0 mL/min, wavelength = 250 nm) $t_{(major)}$ = 9.422 min; $t_{(minor)}$ = 8.928 min, indicated 92% de.
$[\alpha]_D^{28}$ = +35.9 (c = 1.5 in $CHCl_3$).

benzhydryl ([1,1'-biphenyl]-4-carbonyl)-L-phenylalaninate **4al**

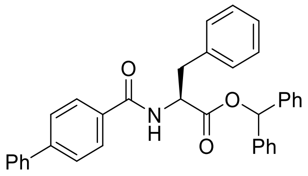

Following **General Procedure A** using α-amino acid **1al** (17.5 mg, 0.05 mmol, 1.0 equiv.) and diphenylmethanol (0.075 mmol, 1.5 equiv.), the crude product was purified by column chromatography petroleum ether/EtOAc (most use 3/1) as eluent to afford the desired product **4al** as a white solid in 90% yield (23.0 mg). **m.p.** 176-177 °C.
TLC (4:1, PE: EtOAc): R_f = 0.3
^{1}H NMR (400 MHz, $CDCl_3$) δ 7.8 (d, *J* = 8.4 Hz, 2H), 7.7 – 7.6 (m, 4H), 7.5 (t, *J* = 7.4 Hz, 2H), 7.4 – 7.3 (m, 10H), 7.2 – 7.1 (m, 3H), 7.0 (s, 1H), 6.9 – 6.9 (m, 2H), 6.6 (d, *J* = 7.6 Hz, 1H), 5.3 (ddd, *J* = 7.8, 6.0, 4.9 Hz, 1H), 3.3 (qd, *J* = 13.9, 5.5 Hz, 2H).
^{13}C NMR (101 MHz, $CDCl_3$).
^{13}C NMR (101 MHz, $CDCl_3$) δ 170.8, 166.5, 144.6, 140.0, 139.4, 139.4, 135.5, 132.5, 129.5, 128.9, 128.7, 128.6, 128.6, 128.4, 128.1, 128.1, 127.8, 127.6, 127.3, 127.2, 127.1, 126.9, 53.5, 37.8.
HRMS (ESI-TOF) *m/z:* $[M+Na]^+$ Calcd for $C_{35}H_{29}NNaO_3^+$ 534.2040, found 534.2042.
HPLC (Chiralpak-IC column, Hexane/^{i}PrOH = 70/30, flow rate: 1.0 mL/min, wavelength = 254 nm) $t_{(major)}$ = 12.127 min; $t_{(minor)}$ = 10.280 min, indicated 99% ee.
$[\alpha]_D^{28}$ = +44.7 (c = 1.02 in $CHCl_3$)

benzhydryl (4-(tert-butyl)benzoyl)-L-phenylalaninate **4am**

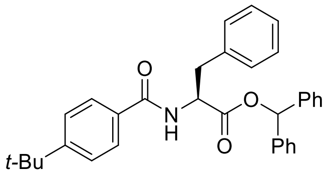

Following **General Procedure A** using α-amino acid **1am** (16.5 mg, 0.05 mmol, 1.0 equiv.) and diphenylmethanol (0.075 mmol, 1.5 equiv.), the crude product was purified by column chromatography petroleum ether/EtOAc (most use 6/1) as eluent to afford the desired product **4am** as a colorless oil in 93% yield (23 mg).

TLC (5:1, PE: EtOAc): R_f = 0.4.

^{1}H NMR (400 MHz, $CDCl_3$) δ 7.7 (d, *J* = 8.5 Hz, 2H), 7.4 (d, *J* = 8.5 Hz, 2H), 7.4 – 7.3 (m, 10H), 7.2 (ddd, *J* = 14.4, 8.6, 6.2, 2.3 Hz, 3H), 7.0 (s, 1H), 6.9 – 6.9 (m, 2H), 6.6 (d, *J* = 7.7 Hz, 1H), 5.3 – 5.2 (m, 1H), 3.3 (qd, *J* = 13.9, 5.5 Hz, 2H), 1.3 (s, 9H).

^{13}C NMR (101 MHz, $CDCl_3$) δ 170.9, 166.8, 155.3, 139.4, 135.6, 131.1, 129.5, 128.7, 128.6, 128.5, 128.4, 128.1, 127.8, 127.0, 126.9, 126.9, 125.6, 78.3, 53.4, 37.8, 35.0, 31.2.

HRMS (ESI-TOF) *m/z:* $[M+Na]^+$ Calcd for $C_{33}H_{33}NNaO_3^+$ 514.2353, found 514.2355.

HPLC (Chiralpak-IC column, Hexane/^{i}PrOH = 70/30, flow rate: 1.0 mL/min, wavelength = 254 nm) $t_{(major)}$ = 10.523 min; $t_{(minor)}$ = 7.750 min, indicated 95% ee.

$[\alpha]_D^{28}$ = +14.5 (c = 1 in $CHCl_3$).

benzhydryl (4-bromobenzoyl)-L-phenylalaninate **4an**

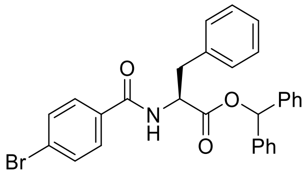

Following **General Procedure A** using α-amino acid **1an** (17.5 mg, 0.05 mmol, 1.0 equiv.) and diphenylmethanol (0.075 mmol, 1.5 equiv.), the crude product was purified by column chromatography petroleum ether/DCM (most use 3/1) as eluent to afford the desired product **4an** as a white solid in 97% yield (25 mg), **m.p.** 105-106 ℃.

TLC (3:1, PE: DCM): R_f = 0.4

^{1}H NMR (400 MHz, $CDCl_3$) δ 7.5 (d, *J* = 1.3 Hz, 4H), 7.4 – 7.3 (m, 10H), 7.2 – 7.1 (m, 3H), 6.9 (s, 1H), 6.9 – 6.8 (m, 2H), 6.5 (d, *J* = 7.6 Hz, 1H), 5.2 (dt, *J* = 7.9, 5.6 Hz, 1H), 3.3 (qd, *J* = 13.9, 5.5 Hz, 2H).

^{13}C NMR (101 MHz, $CDCl_3$) δ 170.7, 165.9, 139.3, 139.3, 135.3, 132.7, 131.9, 129.4, 128.7, 128.6, 128.6, 128.4, 128.1, 127.8, 127.1, 126.9, 126.6, 78.5, 53.5, 37.6.

HRMS (ESI-TOF) *m/z:* $[M+Na]^+$ Calcd for $C_{29}H_{24}BrNNaO_3^+$ 536.0832, found 536.0834.

HPLC (Chiralpak-IB column, Hexane/^{i}PrOH = 90/10, flow rate: 1.0 mL/min, wavelength = 254 nm) $t_{(major)}$ = 12.820 min; $t_{(minor)}$ = 10.820 min, indicated 97% ee.

$[\alpha]_D^{28}$ = +22 (c = 0.7 in $CHCl_3$).

benzhydryl (4-nitrobenzoyl)-L-phenylalaninate **4ao**

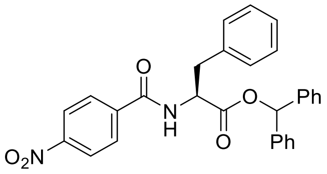

Following **General Procedure A** using α-amino acid **1ao** (15.7 mg, 0.05 mmol, 1.0 equiv.) and diphenylmethanol (0.075 mmol, 1.5 equiv.), the crude product was purified by column chromatography petroleum ether/EtOAc (most use 3/1) as eluent to afford the desired product **4ao** as a yellow solid in 97% yield (23.2 mg). **m.p.** 126-127 °C.

TLC (3:1, PE: EtOAc): R_f = 0.4

^{1}H NMR (400 MHz, CDCl$_3$) δ 8.3 – 8.2 (m, 2H), 7.8 (dd, *J* = 8.8, 3.4 Hz, 2H), 7.4 (qdd, *J* = 11.5, 6.0, 2.8 Hz, 10H), 7.2 – 7.1 (m, 3H), 7.0 (s, 1H), 6.9 – 6.8 (m, 2H), 6.7 (d, *J* = 7.9 Hz, 1H), 5.2 (dt, *J* = 7.7, 5.5 Hz, 1H), 3.4 – 3.2 (m, 2H).

^{13}C NMR (101 MHz, CDCl$_3$) δ 170.6, 164.9, 149.7, 139.4, 139.2, 139.1, 135.1, 129.4, 128.8, 128.7, 128.7, 128.6, 128.2, 128.2, 127.8, 127.3, 126.9, 123.9, 78.7, 53.7, 37.5.

HRMS (ESI-TOF) *m/z:* $[M+Na]^+$ Calcd for $C_{29}H_{24}N_2NaO_5^+$ 503.1577, found 503.1579.

HPLC (Chiralpak-IB column, Hexane/^{i}PrOH = 80/20, flow rate: 1.0 mL/min, wavelength = 254 nm) $t_{(major)}$ = 19.390 min; $t_{(minor)}$ = 13.367 min, indicated 86% ee.

$[\alpha]_D^{28}$ = +11 (c = 2 in $CHCl_3$).

benzhydryl (4-(trifluoromethyl)benzoyl)-L-phenylalaninate **4ap**

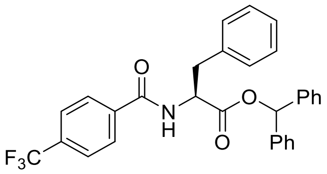

Following **General Procedure A** using α-amino acid **1ap** (17 mg, 0.05 mmol, 1.0 equiv.) and diphenylmethanol (0.075 mmol, 1.5 equiv.), the crude product was purified by column chromatography petroleum ether/DCM (most use 3/1) as eluent to afford the desired product **4ap** as a yellow solid in 81% yield (20.4 mg), **m.p.** 109-111 °C.

TLC (3:1, PE: DCM): R_f = 0.4

^{1}H NMR (400 MHz, CDCl$_3$) δ 7.8 (d, *J* = 8.1 Hz, 2H), 7.7 (d, *J* = 8.2 Hz, 2H), 7.4 – 7.3 (m, 10H), 7.2 – 7.1 (m, 3H), 7.0 (s, 1H), 6.9 – 6.8 (m, 2H), 6.6 (d, *J* = 7.6 Hz, 1H), 5.2 (ddd, *J* = 7.7, 6.0, 5.0 Hz, 1H), 3.4 – 3.2 (m, 2H).

^{13}C NMR (101 MHz, CDCl$_3$) δ 170.7, 165.6, 143.9, 139.2 (d, *J* = 1.7 Hz), 137.2, 135.3, 133.5 (d, *J* = 32.8 Hz), 129.4, 128.7, 128.6, 128.5, 128.2, 127.8, 127.5, 126.9, 126.6, 125.7 (q, *J* = 3.7 Hz), 123.6 (d, *J* = 272.8 Hz), 78.6, 53.6, 37.6.

^{19}F NMR (376 MHz, CDCl$_3$) δ -63.0.

HRMS (ESI-TOF) *m/z:* $[M+Na]^+$ Calcd for $C_{30}H_{24}F_3NNaO_3^+$ 526.1600, found 526.1603.

HPLC (Chiralpak-IB column, Hexane/^{i}PrOH = 90/10, flow rate: 1.0 mL/min, wavelength = 254 nm) $t_{(major)}$ = 12.230 min; $t_{(minor)}$ = 9.483 min, indicated 94% ee.

$[\alpha]_D^{28}$ = +24.5 (c = 1 in $CHCl_3$).

benzhydryl (3-methoxybenzoyl)-L-phenylalaninate **4aq**

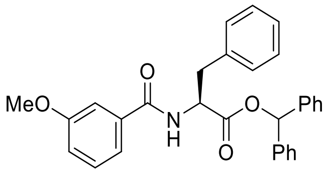

Following **General Procedure A** using α-amino acid **1aq** (15 mg, 0.05 mmol, 1.0 equiv.) and diphenylmethanol (0.075 mmol, 1.5 equiv.), the crude product was purified by column chromatography petroleum ether/EtOAc (most use 3/1) as eluent to afford the desired product **4aq** as a colorless oil in 91% yield (21.2 mg).

TLC (5:1, PE: EtOAc): R_f = 0.3

^{1}H NMR (400 MHz, CDCl$_3$) δ 7.4 – 7.3 (m, 12H), 7.2 – 7.1 (m, 4H), 7.0 (ddd, *J* = 8.3, 2.6, 1.0 Hz, 1H), 7.0 – 6.8 (m, 3H), 6.6 (d, *J* = 7.7 Hz, 1H), 5.2 (ddd, *J* = 7.7, 6.0, 4.9 Hz, 1H), 3.8 (s, 3H), 3.3 (qd, *J* = 13.9, 5.5 Hz, 2H).

^{13}C NMR (101 MHz, CDCl$_3$) δ 170.7, 166.8, 159.8, 139.3, 139.3, 135.4, 135.3, 129.7, 129.5, 128.7, 128.6, 128.6, 128.4, 128.1, 127.8, 127.1, 126.9, 118.8, 118.2, 112.1, 78.3, 55.4, 53.5, 37.7.

HRMS (ESI-TOF) *m/z:* $[M+Na]^+$ Calcd for $C_{30}H_{27}NNaO_4^+$ 488.1832, found 488.1833.

HPLC (Chiralpak-IC column, Hexane/^{i}PrOH = 90/10, flow rate: 1.0 mL/min, wavelength = 254 nm) $t_{(major)}$ = 41.303 min; $t_{(minor)}$ = 46.757 min, indicated 88% ee.

$[\alpha]_D^{28}$ = +29.5 (c = 2 in $CHCl_3$).

benzhydryl (3,5-dimethoxybenzoyl)-L-phenylalaninate **4ar**

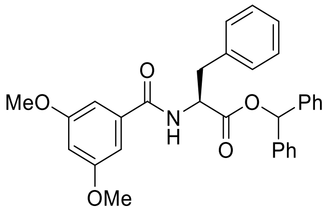

Following **General Procedure A** using α-amino acid **1ar** (16.5 mg, 0.05 mmol, 1.0 equiv.) and diphenylmethanol (0.075 mmol, 1.5 equiv.), the crude product was purified by column chromatography petroleum ether/EtOAc (most use 4/1) as eluent to afford the desired product **4ar** as a white solid in 92% yield (22.8 mg), **m.p.** 109-111 ℃.

TLC (5:1, PE: EtOAc): R_f = 0.22

^{1}H NMR (400 MHz, CDCl$_3$) δ 7.4 – 7.3 (m, 10H), 7.2 – 7.1 (m, 3H), 6.9 (s, 1H), 6.9 (d, *J* = 6.5 Hz, 2H), 6.8 (d, *J* = 2.3 Hz, 2H), 6.6 (t, *J* = 2.3 Hz, 1H), 6.5 (d, *J* = 7.7 Hz, 1H), 5.2 (ddd, *J* = 7.7, 6.0, 5.0 Hz, 1H), 3.8 (s, 6H), 3.3 (qd, *J* = 13.9, 5.6 Hz, 2H).

^{13}C NMR (101 MHz, CDCl$_3$) δ 170.7, 166.7, 160.9, 139.4, 136.2, 135.5, 129.5, 128.7, 128.6, 128.5, 128.4, 128.1, 127.8, 127.1, 126.9, 104.8, 104.1, 78.3, 55.6, 53.5, 37.7.

HRMS (ESI-TOF) *m/z:* $[M+Na]^+$ Calcd for $C_{31}H_{29}NNaO_5^+$ 518.1938, found 518.1939.

HPLC (Chiralpak-IC column, Hexane/^{i}PrOH = 80/20, flow rate: 1.0 mL/min, wavelength = 254 nm) $t_{(major)}$ = 21.860 min; $t_{(minor)}$ = 24.913 min, indicated 90% ee.

$[\alpha]_D^{28}$ = +34.6 (c = 1 in $CHCl_3$).

benzhydryl (1-naphthoyl)-L-phenylalaninate **4as**

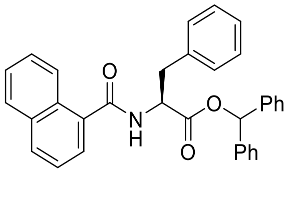

Following **General Procedure A** using α-amino acid **1as** (16 mg, 0.05 mmol, 1.0 equiv.) and diphenylmethanol (0.075 mmol, 1.5 equiv.), the crude product was purified by column chromatography petroleum ether/EtOAc (most use 5/1) as eluent to afford the desired product **4as** as a white solid in 88% yield (21.5 mg). **m.p.** 91-92 ℃.

TLC (5:1, PE: EtOAc): R_f = 0.2

^{1}H NMR (400 MHz, CDCl$_3$) δ 8.2 (d, *J* = 1.7 Hz, 1H), 7.9 – 7.7 (m, 4H), 7.7 – 7.5 (m, 2H), 7.5 – 7.3 (m, 10H), 7.2 – 7.1 (m, 3H), 7.1 – 6.9 (m, 3H), 6.8 (d, *J* = 7.6 Hz, 1H), 5.3 (dt, *J* = 7.8, 5.5 Hz, 1H), 3.4 – 3.2 (m, 2H).

^{13}**C NMR (101 MHz, CDCl$_3$)** δ 170.9, 167.0, 139.4, 135.6, 134.9, 132.6, 131.1, 129.6, 129.0, 128.7, 128.6, 128.6, 128.6, 128.4, 128.1, 127.8, 127.8, 127.8, 127.7, 127.1, 126.9, 126.8, 123.5, 78.4, 53.7, 37.8.

HRMS (ESI-TOF) *m/z:* [M+Na]$^+$ Calcd for $C_{33}H_{27}NNaO_3^+$ 508.1883, found 508.1886.

HPLC (Chiralpak-IA column, Hexane/^{i}PrOH = 70/30, flow rate: 1.0 mL/min, wavelength = 254 nm) t$_{(major)}$ = 15.773 min; t$_{(minor)}$ = 10.820 min, indicated 92% ee.

$[\alpha]_D^{28}$ = +47.5 (c = 1.2 in $CHCl_3$).

benzhydryl (cyclopropanecarbonyl)-L-phenylalaninate **4at**

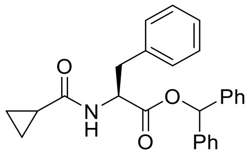

Following **General Procedure A** using α-amino acid **1at** (12 mg, 0.05 mmol, 1.0 equiv.) and diphenylmethanol (0.075 mmol, 1.5 equiv.), the crude product was purified by column chromatography petroleum ether/EtOAc (most use 6/1) as eluent to afford the desired product **4at** as a white solid in 69% yield (14 mg). **m.p.** 114-115 °C.

TLC (5:1, PE: EtOAc): R$_f$ = 0.4

^{1}**H NMR (400 MHz, CDCl$_3$)** δ 7.4 – 7.3 (m, 10H), 7.2 – 7.1 (m, 3H), 6.9 – 6.8 (m, 3H), 6.1 (d, *J* = 7.9 Hz, 1H), 5.1 (dt, *J* = 7.9, 5.6 Hz, 1H), 3.1 (d, *J* = 5.6 Hz, 2H), 1.3 (dt, *J* = 7.8, 4.5 Hz, 1H), 1.0 (dq, *J* = 4.6, 1.6 Hz, 2H), 0.8 – 0.7 (m, 2H).

^{13}**C NMR (101 MHz, CDCl$_3$)** δ 173.1, 170.9, 139.4, 139.4, 135.6, 129.5, 128.7, 128.6, 128.5, 128.3, 128.1, 127.7, 127.0, 126.9, 78.1, 53.1, 37.8, 14.7, 7.5.

HRMS (ESI-TOF) *m/z:* [M+Na]$^+$ Calcd for $C_{26}H_{25}NNaO_3^+$ 422.1727, found 422.1724.

HPLC (Chiralpak-IA column, Hexane/^{i}PrOH = 80/20, flow rate: 1.0 mL/min, wavelength = 221 nm) t$_{(major)}$ = 11.387 min; t$_{(minor)}$ = 6.756 min, indicated 96% ee.

$[\alpha]_D^{28}$ = +40 (c = 1.5 in $CHCl_3$).

benzhydryl (thiophene-2-carbonyl)-L-phenylalaninate **4au**

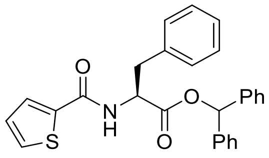

Following **General Procedure A** using α-amino acid **1au** (14 mg, 0.05 mmol, 1.0 equiv.) and diphenylmethanol (0.075 mmol, 1.5 equiv.), the crude product was purified by column chromatography petroleum ether/EtOAc (most use 4/1) as eluent to afford the desired product **4au** as a colorless oil in 81% yield (18 mg).

TLC (4:1, PE: EtOAc): R_f = 0.2

^{1}H NMR (400 MHz, CDCl$_3$) δ 7.5 (dd, *J* = 5.0, 1.2 Hz, 1H), 7.4 (dd, *J* = 3.7, 1.2 Hz, 1H), 7.4 – 7.3 (m, 9H), 7.2 (dddd, *J* = 14.4, 8.6, 6.2, 2.2 Hz, 3H), 7.0 (dd, *J* = 5.0, 3.7 Hz, 1H), 7.0 – 6.8 (m, 3H), 6.5 (d, *J* = 7.7 Hz, 1H), 5.2 (ddd, *J* = 7.8, 6.0, 5.0 Hz, 1H), 3.3 – 3.1 (m, 2H).

^{13}C NMR (101 MHz, CDCl$_3$) δ 170.6, 161.3, 139.3, 139.3, 138.2, 135.3, 130.5, 129.5, 128.7, 128.6, 128.6, 128.5, 128.4, 128.1, 127.8, 127.7, 127.1, 126.9, 78.4, 53.4, 37.8.

HRMS (ESI-TOF) *m/z:* $[M+Na]^+$ Calcd for $C_{27}H_{23}NNaO_3S^+$ 464.1291, found 464.1290.

HPLC (Chiralpak-IC column, Hexane/^{i}PrOH = 70/30, flow rate: 1.0 mL/min, wavelength = 254 nm) $t_{(major)}$ = 9.397 min; $t_{(minor)}$ = 10.623 min, indicated 96% ee.

$[\alpha]_D^{28}$ = +3.5 (c = 1 in CHCl$_3$).

benzyl (4-methoxybenzoyl)-L-phenylalaninate **4av**

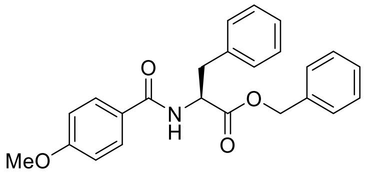

Following **General Procedure A** using α-amino acid **1a** (15 mg, 0.05 mmol, 1.0 equiv.) and phenylmethanol (10 μL, 0.1 mmol, 2 equiv.), the crude product was purified by column chromatography petroleum ether/EtOAc (most use 4/1) as eluent to afford the desired product **4av** as a white solid in 81% yield (15.8 mg). **m.p.** 113-114 °C.

TLC (4:1, PE: EtOAc): R_f = 0.4

^{1}H NMR (400 MHz, CDCl$_3$) δ 7.8 – 7.6 (m, 2H), 7.4 – 7.3 (m, 5H), 7.3 – 7.2 (m, 3H), 7.1 – 7.0 (m, 2H), 6.9 (d, *J* = 8.8 Hz, 2H), 6.6 (d, *J* = 7.6 Hz, 1H), 5.4 – 5.1 (m, 3H), 3.8 (s, 3H), 3.4 – 3.1 (m, 2H).

^{13}C NMR (101 MHz, CDCl$_3$) δ 171.7, 166.4, 162.4, 135.8, 135.1, 129.4, 128.9, 128.7, 128.6, 127.1, 127.0, 126.2, 113.8, 67.4, 55.4, 53.5, 37.9.

HRMS (ESI-TOF) *m/z:* $[M+H]^+$ Calcd for $C_{24}H_{24}NO_4^+$ 390.1700, found 390.1694.

HPLC (Chiralpak-IC column, Hexane/^{i}PrOH = 80/20, flow rate: 1.0 mL/min, wavelength = 250 nm) $t_{(major)}$ = 18.563 min; $t_{(minor)}$ = 22.229 min, indicated 90% ee.

$[\alpha]_D^{28}$ = +16 (c = 0.8 in CHCl$_3$)

methyl (4-methoxybenzoyl)-L-phenylalanyl-D-phenylalaninate **4aw**

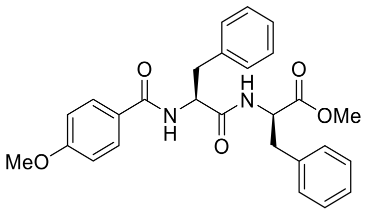

Following the **General Procedure A** using α-amino acid **1a** (15 mg, 0.05 mmol, 1.0 equiv.), H-D-Phe-OMe.HCl (13.0 mg, 0.06 mmol, 1.2 equiv.) NHC-**3e** (5 mg, 0.01 mmol, 20% mol) and KOAc were used as a base; the crude product was purified by column chromatography using petroleum ether/EtOAc (most commonly 3:1) as the eluent to afford the desired product **4aw** as a white solid in 91% yield (21.0 mg). **m.p.** 176-177 °C.

TLC (3:1, PE: EtOAc): R_f = 0.4

^{1}H NMR (400 MHz, Methanol-*d*$_4$) δ 7.68 (d, *J* = 8.8 Hz, 2H), 7.29 – 7.11 (m, 10H), 6.94 (d, *J* = 8.9 Hz, 2H), 4.83 – 4.79 (m, 1H), 4.73 – 4.65 (m, 1H), 3.83 (s, 3H), 3.69 (s, 3H), 3.17 – 2.84 (m, 4H).

^{13}C NMR (101 MHz, Methanol-*d*$_4$) δ 172.22, 171.73, 168.09, 162.71, 137.06, 136.49, 128.92, 128.77, 128.21, 128.09, 128.01, 126.61, 126.35, 125.74, 113.29, 54.90, 54.53, 53.68, 51.37, 37.42, 37.01.

HRMS (ESI-TOF) *m/z:* $[M+Na]^+$ Calcd for $C_{27}H_{28}N_2NaO_5^+$ 483.1896, found 483.1896.

HPLC (Chiralpak-IC column, Hexane/^{i}PrOH = 80/20, flow rate: 1.0 mL/min, wavelength = 250 nm) $t_{(major)}$ = 18.094 min; $t_{(minor)}$ = 9.850 min, indicated 72% de.

$[\alpha]_D^{28}$ = +6 (c = 1 in $CHCl_3$).

benzhydryl benzoylglycyl-L-phenylalaninate **6a**

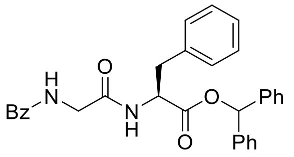

Following **General Procedure A** using peptide **5a** (16.5 mg, 0.05 mmol, 1.0 equiv.) and diphenylmethanol (0.075 mmol, 1.5 equiv.), the crude product was purified by column chromatography petroleum ether/DCM (most use 3/1) as eluent to afford the desired product **6a** as a white solid in 96% yield (23.6 mg). **m.p.** 77-79 °C.

TLC (2:1, PE: DCM): R_f = 0.5

^{1}H NMR (400 MHz, $CDCl_3$) δ 7.9 – 7.7 (m, 2H), 7.6 – 7.5 (m, 1H), 7.4 (dd, *J* = 8.4, 6.9 Hz, 2H), 7.4 – 7.3 (m, 10H), 7.2 – 7.0 (m, 3H), 7.0 (t, *J* = 5.2 Hz, 1H), 6.9 – 6.8 (m,

3H), 6.7 (d, *J* = 8.0 Hz, 1H), 5.0 (dt, *J* = 8.1, 5.9 Hz, 1H), 4.1 (d, *J* = 5.1 Hz, 2H), 3.1 (qd, *J* = 13.9, 5.9 Hz, 2H).

^{13}C NMR (101 MHz, $CDCl_3$) δ 170.3, 168.6, 167.7, 139.3, 135.2, 133.3, 132.0, 129.3, 128.7, 128.7, 128.6, 128.6, 128.4, 128.1, 127.6, 127.2, 126.9, 126.2, 115.7, 78.3, 53.3, 43.5, 37.7.

HRMS (ESI-TOF) *m/z:* $[M+Na]^+$ Calcd for $C_{31}H_{28}N_2NaO_4^+$ 515.1941, found 515.1945.

HPLC (Chiralpak-IB column, Hexane/^{i}PrOH = 80/20, flow rate: 1.0 mL/min, wavelength = 254 nm) $t_{(major)}$ = 7.967 min; $t_{(minor)}$ = 10.130 min, indicated 97% ee.

$[\alpha]_D^{28}$ = +25 (c = 1 in $CHCl_3$)

benzhydryl (tert-butoxycarbonyl)-L-alanyl-L-phenylalaninate **6b**

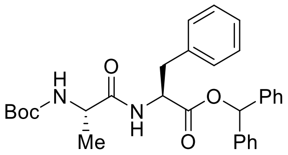

Following **General Procedure A** using peptide **5b** (17 mg, 0.05 mmol, 1.0 equiv.) and diphenylmethanol (0.075 mmol, 1.5 equiv.), the crude product was purified by column chromatography petroleum ether/EtOAc (most use 5/1) as eluent to afford the desired product **6b** as a white solid in 90% yield (22.6 mg). **m.p.** 114-115 °C.

TLC (4:1, PE: EA): R_f = 0.5

^{1}H NMR (400 MHz, $CDCl_3$) δ 7.3 – 7.3 (m, 10H), 7.2 (dd, *J* = 9.2, 6.9 Hz, 3H), 6.9 – 6.9 (m, 3H), 6.6 (s, 1H), 5.0 (dt, *J* = 8.0, 5.8 Hz, 1H), 4.8 (s, 1H), 4.1 (d, *J* = 10.7 Hz, 1H), 3.2 – 3.1 (m, 2H), 1.4 (s, 9H), 1.3 (d, *J* = 7.1 Hz, 3H).

^{13}C NMR (101 MHz, $CDCl_3$) δ 172.1, 170.4, 139.4, 135.3, 129.4, 128.7, 128.6, 128.3, 128.1, 127.7, 127.1, 126.9, 80.2, 78.2, 53.0, 50.1, 37.7, 29.7, 28.3.

HRMS (ESI-TOF) *m/z:* $[M+Na]^+$ Calcd for $C_{30}H_{34}N_2NaO_5^+$ 525.2360, found 525.2363.

HPLC (Chiralpak-IA column, Hexane/^{i}PrOH = 80/20, flow rate: 1.0 mL/min, wavelength = 214 nm) $t_{(major)}$ = 11.064 min; $t_{(minor)}$ = 7.202 min, indicated 96% de.

$[\alpha]_D^{28}$ = +62 (c = 0.8 in $CHCl_3$).

benzhydryl (tert-butoxycarbonyl)-L-phenylalanyl-L-phenylalaninate **6c**

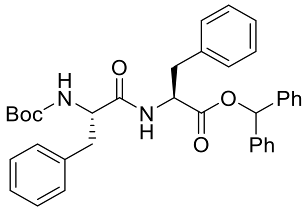

Following **General Procedure A** using peptide **5c** (20.6 mg, 0.05 mmol, 1.0 equiv.) and diphenylmethanol (0.075 mmol, 1.5 equiv.), the crude product was purified by column chromatography petroleum ether/EtOAc (most use 5/1) as eluent to afford the desired product **6c** as a white solid in 91% yield (26.2 mg). **m.p.** 133-134 ℃.

TLC (3:1, PE: DCM): R_f = 0.5

^{1}H NMR (400 MHz, CDCl$_3$) δ 7.4 – 7.2 (m, 10H), 7.2 – 7.0 (m, 8H), 6.9 (s, 1H), 6.8 (s, 2H), 6.3 (d, *J* = 7.7 Hz, 1H), 4.9 (s, 2H), 4.4 – 4.3 (m, 1H), 3.2 – 2.9 (m, 4H), 1.4 (s, 9H).

^{13}C NMR (101 MHz, CDCl$_3$) δ 170.8, 170.1, 139.4, 139.3, 136.5, 135.3, 129.3, 128.6, 128.6, 128.5, 128.3, 128.1, 127.6, 127.0, 78.1, 53.3, 37.8, 28.2.

HRMS (ESI-TOF) *m/z:* $[M+Na]^+$ Calcd for $C_{36}H_{38}N_2NaO_5^+$ 601.2673, found 601.2675.

HPLC (Chiralpak-IA column, Hexane/^{i}PrOH = 80/20, flow rate: 1.0 mL/min, wavelength = 250 nm) $t_{(major)}$ = 7.287 min; $t_{(minor)}$ = 11.108 min, indicated 95% de.

$[\alpha]_D^{28}$ = +47 (c = 1 in $CHCl_3$).

benzhydryl ((benzyloxy)carbonyl)-D-tryptophyl-L-tryptophanate **6d**

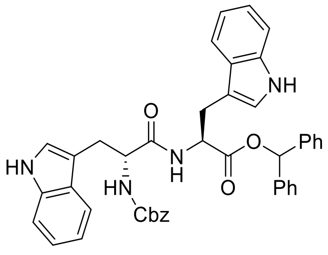

Following **General Procedure A** using peptide **5d** (26 mg, 0.05 mmol, 1.0 equiv.) and diphenylmethanol (0.075 mmol, 1.5 equiv.), the crude product was purified by column chromatography petroleum ether/EtOAc (most use 2/1) as eluent to afford the desired product **6d** as a yellow solid in 82% yield (28.3 mg). **m.p.** 144-145 ℃.

TLC (1:1, PE: EA): R_f = 0.3

^{1}H NMR (400 MHz, CDCl$_3$) δ 7.9 (s, 1H), 7.8 (d, *J* = 2.6 Hz, 1H), 7.6 (d, *J* = 7.8 Hz, 1H), 7.3 – 7.3 (m, 12H), 7.2 (d, *J* = 8.9 Hz, 3H), 7.1 (dt, *J* = 27.0, 8.1 Hz, 4H), 6.8 (d, *J* = 16.8 Hz, 2H), 6.7 (s, 1H), 6.4 (d, *J* = 8.1 Hz, 1H), 6.3 (s, 1H), 5.5 (d, *J* = 7.7 Hz,

1H), 5.0 (td, J = 11.8, 6.8 Hz, 3H), 4.5 (s, 1H), 3.3 – 3.2 (m, 1H), 3.2 (d, J = 5.7 Hz, 2H), 3.1 (dd, J = 14.6, 7.4 Hz, 1H).

^{13}C NMR (101 MHz, CDCl$_3$) δ 171.1, 170.6, 156.0, 139.8, 139.6, 136.2, 136.0, 128.7, 128.6, 128.6, 128.2, 128.2, 128.1, 128.1, 128.0, 127.4, 127.4, 127.3, 127.1, 123.6, 123.2, 122.2, 122.1, 119.7, 119.6, 118.9, 118.5, 111.3, 111.3, 110.0, 109.1, 78.1, 66.9, 55.4, 53.0, 28.5, 27.5.

HRMS (ESI-TOF) *m/z:* $[M+Na]^+$ Calcd for $C_{43}H_{38}N_4NaO_5^+$ 713.2734, found 713.2737.

HPLC (Chiralpak-IC column, Hexane/^{i}PrOH = 80/20, flow rate: 1.0 mL/min, wavelength = 263 nm) $t_{(major)}$ = 18.705 min; $t_{(minor)}$ = 26.107 min, indicated 97% de.

$[\alpha]_D^{28}$ = +43.5 (c = 1 in CHCl$_3$).

benzhydryl (tert-butoxycarbonyl)-L-phenylalanylglycyl-L-phenylalaninate **6e**

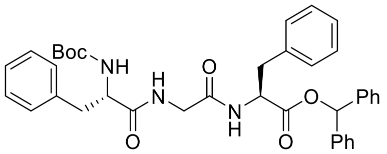

Following **General Procedure A** using peptide **5e** (23.5 mg, 0.05 mmol, 1.0 equiv.) and diphenylmethanol (0.075 mmol, 1.5 equiv.), the crude product was purified by column chromatography petroleum ether/EtOAc (most use 5/1) as eluent to afford the desired product **6e** as a colorless oil in 74% yield (23.5 mg).

TLC (3:1, PE: DCM): R_f = 0.5

^{1}H NMR (400 MHz, Methanol-*d*$_4$) δ 7.3 – 7.2 (m, 19H), 7.1 – 7.1 (m, 2H), 6.8 (s, 1H), 4.8 (dd, J = 8.0, 6.4 Hz, 1H), 4.3 – 4.2 (m, 1H), 3.8 (p, J = 6.5 Hz, 1H), 3.7 (d, J = 16.9 Hz, 1H), 3.2 – 3.0 (m, 3H), 2.8 (dd, J = 13.8, 9.4 Hz, 1H), 1.3 (s, 9H).

^{13}C NMR (101 MHz, Methanol-*d*$_4$) δ 173.4, 170.4, 169.9, 156.4, 139.9, 137.3, 136.4, 131.0, 128.9, 128.2, 128.0, 127.6, 127.6, 126.9, 126.7, 126.5, 126.3, 79.3, 78.0, 56.3, 54.2, 48.1, 47.9, 47.7, 41.3, 37.4, 36.9, 27.3.

HRMS (ESI-TOF) *m/z:* $[M+Na]^+$ Calcd for $C_{38}H_{41}N_3NaO_6^+$ 658.2893, found 658.2894.

HPLC (Chiralpak-IA column, Hexane/^{i}PrOH = 95/5, flow rate: 1.0 mL/min, wavelength = 215 nm) $t_{(major)}$ = 14.607 min; $t_{(minor)}$ = 12.784 min, indicated 97% de.

$[\alpha]_D^{28}$ = +17.5 (c = 1.2 in CHCl$_3$).

benzhydryl (tert-butoxycarbonyl)-L-phenylalanyl-L-alanyl-L-phenylalaninate **6f**

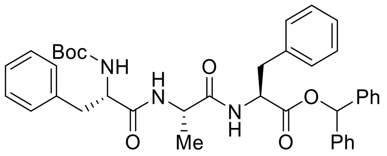

Following **General Procedure A** using peptide **5f** (24.5 mg, 0.05 mmol, 1.0 equiv.) and diphenylmethanol (0.075 mmol, 1.5 equiv.), the crude product was purified by column chromatography petroleum ether/EtOAc (most use 5/1) as eluent to afford the desired product **6f** as a white solid in 80% yield (26.0 mg). **m.p.** 102-103℃.

TLC (3:1, PE: DCM): R_f = 0.5

^{1}H NMR (400 MHz, Acetone-*d*$_6$) δ 7.8 (d, *J* = 7.9 Hz, 1H), 7.6 (d, *J* = 8.2 Hz, 1H), 7.4 – 7.2 (m, 21H), 7.0 (s, 1H), 6.9 (s, 1H), 5.4 (d, *J* = 4.1 Hz, 1H), 4.9 (td, *J* = 7.9, 5.9 Hz, 1H), 4.8 (t, *J* = 4.4 Hz, 1H), 3.2 – 3.0 (m, 4H), 1.3 (s, 9H), 1.2 (d, *J* = 7.0 Hz, 3H).

^{13}C NMR (101 MHz, Acetone-*d*$_6$) δ 171.9, 171.1, 170.1, 155.4, 141.3, 140.7, 136.8, 136.7, 129.4, 129.3, 128.7, 128.5, 128.3, 128.2, 127.9, 127.8, 127.1, 126.8, 126.6, 126.3, 125.7, 124.8, 78.1, 77.6, 60.7, 57.6, 53.9, 37.4, 37.1, 27.6, 17.1.

HRMS (ESI-TOF) *m/z:* $[M+Na]^+$ Calcd for $C_{39}H_{43}N_3NaO_6^+$ 672.3044, found 672.3046.

HPLC (Chiralpak-IC column, Hexane/^{i}PrOH = 80/20, flow rate: 1.0 mL/min, wavelength = 216 nm) $t_{(major)}$ = 8.441 min; $t_{(minor)}$ = 11.600 min, indicated 87% de.

$[\alpha]_D^{28}$ = +13 (c = 0.9 in $CHCl_3$).

benzhydryl (tert-butoxycarbonyl)-L-phenylalanylglycyl-L-tryptophanate **6g**

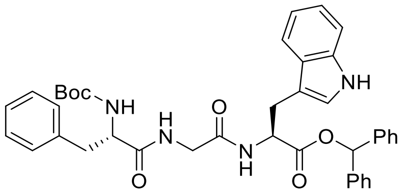

Following **General Procedure A** using peptide **5g** (25.5 mg, 0.05 mmol, 1.0 equiv.) and diphenylmethanol (0.075 mmol, 1.5 equiv.), the crude product was purified by column chromatography petroleum ether/EtOAc (most use 5/1) as eluent to afford the desired product **6g** as a white solid in 73% yield (24.6 mg). **m.p.** 145-146 ℃.

TLC (3:1, PE: DCM): R_f = 0.5

^{1}H NMR (400 MHz, Acetone-*d*$_6$) δ 10.1 (s, 1H), 7.8 – 7.6 (m, 2H), 7.5 (ddd, *J* = 6.9, 5.6, 2.8 Hz, 1H), 7.4 – 7.2 (m, 15H), 7.2 (ddd, *J* = 7.6, 4.1, 2.3 Hz, 1H), 7.1 (ddt, *J* = 11.2, 6.2, 2.0 Hz, 2H), 7.0 – 7.0 (m, 1H), 6.8 (d, *J* = 1.8 Hz, 1H), 6.2 (d, *J* = 7.9 Hz, 1H), 5.0 – 4.9 (m, 1H), 4.4 – 4.3 (m, 1H), 4.0 – 3.8 (m, 2H), 3.4 – 3.1 (m, 3H), 2.9 – 2.9 (m, 1H), 1.3 (d, *J* = 5.2 Hz, 9H).

^{13}C NMR (101 MHz, Acetone-d_6) δ 171.9, 170.6, 168.7, 140.6, 138.0, 136.7, 129.3, 128.4, 128.4, 128.2, 127.7, 127.6, 126.9, 126.9, 126.8, 126.3, 123.8, 123.8, 121.3, 118.8, 118.3, 111.4, 109.6, 109.5, 78.7, 77.5, 56.3, 53.4, 42.4, 37.7, 27.7, 24.9.

HRMS (ESI-TOF) *m/z:* $[M+Na]^+$ Calcd for $C_{40}H_{42}N_4NaO_4^+$ 697.2997, found 697.2999.

HPLC (Chiralpak-AS column, Hexane/^{i}PrOH = 80/20, flow rate: 1.0 mL/min, wavelength = 289 nm) $t_{(major)}$ = 8.347 min; $t_{(minor)}$ = 14.466 min, indicated 99% de.

$[\alpha]_D^{28}$ = +56.9 (c = 1 in $CHCl_3$).

benzhydryl (tert-butoxycarbonyl)-L-phenylalanylglycyl-L-tryptophanate **6h**

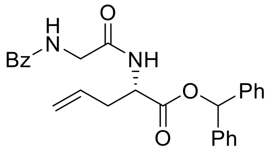

Following **General Procedure A** using peptide **5h** (14 mg, 0.05 mmol, 1.0 equiv.) and diphenylmethanol (0.075 mmol, 1.5 equiv.), the crude product was purified by column chromatography petroleum ether/EtOAc (most use 3/1) as eluent to afford the desired product **6h** as a white solid in 92% yield (20.1 mg). **m.p.** 99-100 ℃.

TLC (4:1, PE: EA): R_f = 0.5

^{1}H NMR (400 MHz, $CDCl_3$) δ 7.8 (d, *J* = 7.0 Hz, 2H), 7.5 – 7.5 (m, 1H), 7.4 (dd, *J* = 8.2, 6.8 Hz, 2H), 7.4 – 7.3 (m, 10H), 7.2 (d, *J* = 5.0 Hz, 1H), 6.9 (d, *J* = 24.2 Hz, 2H), 5.6 (ddt, *J* = 18.3, 9.1, 7.2 Hz, 1H), 5.1 – 4.9 (m, 2H), 4.8 (ddd, *J* = 7.9, 6.4, 5.2 Hz, 1H), 4.1 (dq, *J* = 5.2, 2.6 Hz, 2H), 2.7 – 2.5 (m, 2H).

^{13}C NMR (101 MHz, $CDCl_3$) δ 170.5, 168.8, 167.8, 157.1, 139.5, 139.3, 133.5, 131.8, 131.6, 128.6, 128.6, 128.2, 128.1, 127.3, 127.2, 127.0, 119.7, 78.3, 51.9, 43.6, 36.2.

HRMS (ESI-TOF) *m/z:* $[M+Na]^+$ Calcd for $C_{27}H_{26}N_2NaO_4^+$ 465.1785, found 465.1787.

HPLC (Chiralpak-IA column, Hexane/^{i}PrOH = 80/20, flow rate: 1.0 mL/min, wavelength = 229 nm) $t_{(major)}$ = 10.878 min; $t_{(minor)}$ = 9.336 min, indicated 90% ee.

$[\alpha]_D^{28}$ = +59.5 (c = 2 in $CHCl_3$).

benzhydryl (S,Z)-2-(2-acetamido-3-phenylacrylamido)-3-(4-nitrophenyl)propanoate **6i**

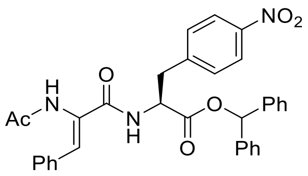

Following **General Procedure A** using peptide **5i** (20 mg, 0.05 mmol, 1.0 equiv.) and diphenylmethanol (0.075 mmol, 1.5 equiv.), the crude product was purified by column chromatography petroleum ether/EtOAc (most use 3/1) as eluent to afford the desired product **6i** as a yellow solid in 74% yield (20.9 mg). **m.p.** 104-105 °C

TLC (4:1, PE: EA): R_f = 0.5

^{1}H NMR (400 MHz, Acetone-d_6) δ 8.7 (s, 1H), 8.0 (d, *J* = 8.7 Hz, 2H), 7.8 (d, *J* = 8.0 Hz, 1H), 7.5 (d, *J* = 2.7 Hz, 3H), 7.4 – 7.3 (m, 14H), 7.1 (s, 1H), 6.9 (s, 1H), 5.0 – 5.0 (m, 1H), 3.4 – 3.3 (m, 2H), 2.1 (s, 3H).

^{13}C NMR (101 MHz, Acetone-d_6) δ 169.9, 169.4, 165.2, 146.9, 145.2, 140.4, 134.3, 130.7, 129.9, 129.4, 128.7, 128.5, 128.5, 128.4, 127.9, 127.7, 127.2, 126.8, 123.2, 77.8, 53.8, 36.7, 22.2.

HRMS (ESI-TOF) *m/z:* $[M+Na]^+$ Calcd for $C_{33}H_{29}N_3NaO_6^+$ 586.1949, found 586.1950.

HPLC (Chiralpak-IA column, Hexane/^{i}PrOH = 70/30, flow rate: 1.0 mL/min, wavelength = 250 nm) $t_{(major)}$ = 29.329 min; $t_{(minor)}$ = 20.597 min, indicated 93% ee.

$[\alpha]_D^{28}$ = +40 (c = 2 in $CHCl_3$).

benzhydryl (S)-2-((S)-2-((((9H-fluoren-9-yl)methoxy)carbonyl)amino)propanamido)-4-phenylbutanoate **6j**

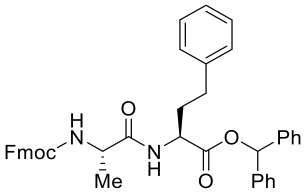

Following **General Procedure A** using peptide **5j** (27.5 mg, 0.05 mmol, 1.0 equiv.) and diphenylmethanol (0.075 mmol, 1.5 equiv.), the crude product was purified by column chromatography petroleum ether/EtOAc (most use 3/1) as eluent to afford the desired product **6j** as a white solid in 85% yield (28.7 mg). **m.p.** 161-162 °C

TLC (4:1, PE: EA): R_f = 0.5

^{1}H NMR (400 MHz, $CDCl_3$) δ 7.8 (d, *J* = 7.5 Hz, 2H), 7.6 (d, *J* = 6.4 Hz, 2H), 7.4 – 7.3 (m, 14H), 7.2 (dt, *J* = 14.1, 7.1 Hz, 3H), 7.0 – 7.0 (m, 2H), 6.9 (s, 1H), 6.5 (d, *J* = 7.9 Hz, 1H), 5.2 (d, *J* = 7.8 Hz, 1H), 4.8 (td, *J* = 7.3, 4.9 Hz, 1H), 4.4 (d, *J* = 6.8 Hz, 2H), 3.8 (p, *J* = 6.4 Hz, 1H), 2.5 (dtd, *J* = 13.8, 8.9, 8.4, 5.3 Hz, 2H), 2.3 – 2.2 (m, 1H), 2.1 (td, *J* = 9.6, 6.8 Hz, 1H), 1.3 (d, *J* = 7.0 Hz, 3H).

^{13}C NMR (101 MHz, CDCl$_3$) δ 171.7, 170.9, 143.7, 141.3, 140.7, 139.5, 139.2, 128.7, 128.6, 128.5, 128.4, 128.3, 128.2, 127.8, 127.3, 127.1, 127.0, 126.2, 125.0, 120.0, 78.3, 67.0, 52.3, 50.4, 47.1, 42.3, 33.6, 31.3.

HRMS (ESI-TOF) *m/z:* $[M+Na]^+$ Calcd for $C_{41}H_{38}N_2NaO_5^+$ 661.2673, found 661.2675.

HPLC (Chiralpak-IA column, Hexane/^{i}PrOH = 70/30, flow rate: 1.0 mL/min, wavelength = 250 nm) $t_{(major)}$ = 6.118 min; $t_{(minor)}$ = 7.607 min, indicated 96% de.

$[\alpha]_D^{28}$ = +47.9 (c = 1.3 in CHCl$_3$).

Benzhydryl (((9H-fluoren-9-yl)methoxy)carbonyl)-L-phenylalanyl-L-phenylalaninate **6k**

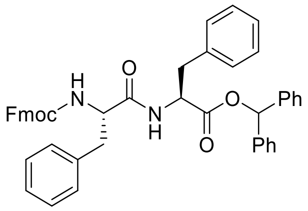

Following **General Procedure A** using peptide **5k** (27 mg, 0.05 mmol, 1.0 equiv.) and diphenylmethanol (0.075 mmol, 1.5 equiv.), the crude product was purified by column chromatography petroleum ether/EtOAc (most use 5/1) as eluent to afford the desired product **6k** as a white solid in 90% yield (31.5 mg). **m.p.** 172-173 °C.

TLC (3:1, PE: EA): R_f = 0.5

^{1}H NMR (400 MHz, CDCl$_3$) δ 7.8 (d, *J* = 7.6 Hz, 2H), 7.5 (d, *J* = 6.9 Hz, 2H), 7.5 – 7.3 (m, 14H), 7.2 (d, *J* = 6.8 Hz, 3H), 7.1 – 7.0 (m, 5H), 6.9 (s, 1H), 6.8 (d, *J* = 7.3 Hz, 2H), 6.3 (d, *J* = 7.3 Hz, 1H), 5.3 (d, *J* = 6.9 Hz, 1H), 4.9 (q, *J* = 6.4 Hz, 1H), 4.5 – 4.3 (m, 2H), 4.3 (d, *J* = 7.9 Hz, 1H), 4.2 (t, *J* = 7.8 Hz, 1H), 3.2 – 2.9 (m, 4H).

^{13}C NMR (101 MHz, CDCl$_3$) δ 170.2, 170.1, 143.7, 141.3, 139.4, 139.3, 135.2, 129.3, 129.3, 128.7, 128.6, 128.6, 128.5, 128.3, 128.1, 127.8, 127.6, 127.1, 127.1, 126.9, 120.0, 78.2, 67.1, 53.3, 47.1, 37.8, 23.5.

HRMS (ESI-TOF) *m/z:* $[M+Na]^+$ Calcd for $C_{46}H_{60}N_2NaO_5^+$ 723.2829, found 723.2831.

HPLC (Chiralpak-IA column, Hexane/^{i}PrOH = 70/30, flow rate: 1.0 mL/min, wavelength = 250 nm) $t_{(major)}$ = 7.057 min; $t_{(minor)}$ = 10.634 min, indicated 95% de.

$[\alpha]_D^{28}$ = +74.2 (c = 1 in CHCl$_3$).

Racemization-free peptide synthesis

Table S4 General procedure for the screening of different NHC-precursors and bases.

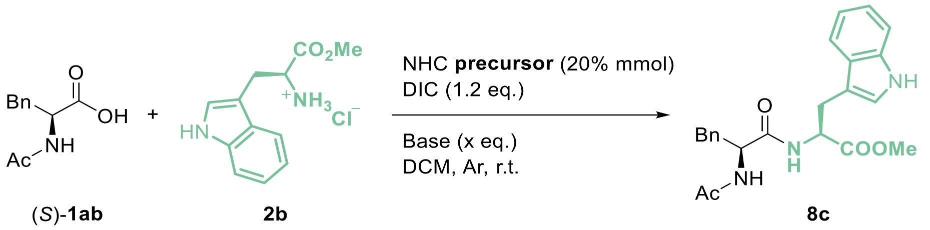

Entry	NHC	Base	Yield of **8c** (%) [a]	es (%) [b]
1	***NHC-3a***	Cs_2CO_3 1 eq.	97	27
2	--	Cs_2CO_3 1 eq.	94	8
3	--	--	42	64
2	***NHC-3a***	LiHMDs 1 eq.	trace	--
3	***NHC-3a***	NaOAc 1 eq.	61	53
4	***NHC-3k***	NaOAc 1 eq.	64	61
5	***NHC-3x***	NaOAc 1 eq.	64	90
6	***NHC-3y***	NaOAc 1 eq.	61	83
7	***NHC-3x***	NaOAc 0.5 eq.	80	94
7	***NHC-7a***	--	73	99

Reaction conditions: A mixture of (*S*)-**1ab** (0.05 mmol, 1.0 eq.), and **2b** (0.055 mmol, 1.1 eq.), NHC-precursor (20 mol%), base (x eq.) and DIC (0.06 mmol, 1.2 eq.) dissolved in DCM (0.05 M) was stirred at r.t. for 3-6 hours. [a]Isolated yields are presented. [b]Chiral HPLC determined es values.

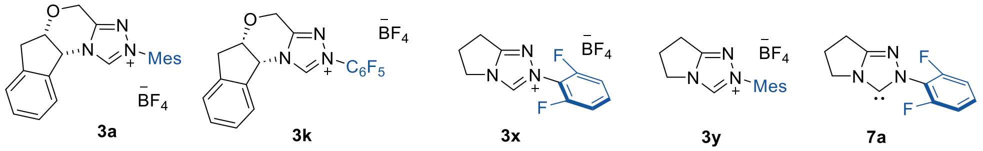

Figure S6 General process of synthesis of peptides without racemization by NHC.

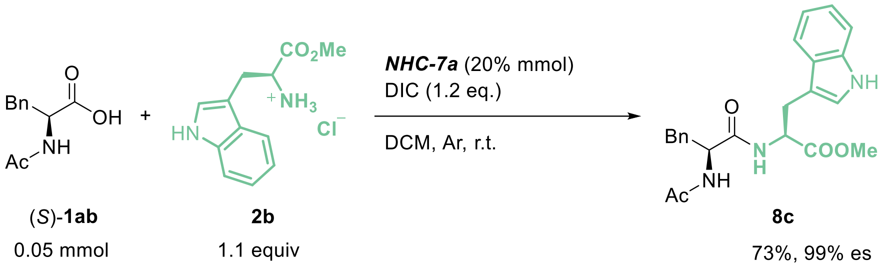

In a glove box under an argon atmosphere, the NHC precursor was pre-activated with NaH (1.2 equiv) for 1 h. After filtration, the resulting solution of free NHC-**7a** (0.01 mmol, 20 mol%) was added to a reaction mixture containing α-amino acid **1** (0.05 mmol) and amine **2b** (0.055 mmol, 1.1 equiv) in an oven-dried 5 mL Schlenk tube equipped with a magnetic stir bar. Dry dichloromethane (DCM, 1 mL) and DIC (0.06 mmol) were subsequently added inside the glove box. The tube was sealed with a screw cap, and the mixture was stirred vigorously at room temperature (25°C) for 6 hours. Upon complete consumption of α-amino acid (*S*)-**1ab** (monitored by TLC), the crude reaction mixture was purified by flash column chromatography (petroleum ether/ethyl acetate = 2:1 to 1:1) to afford the desired peptide product **8c**.

Figure S7 Gram-scale synthesis of **8c**.

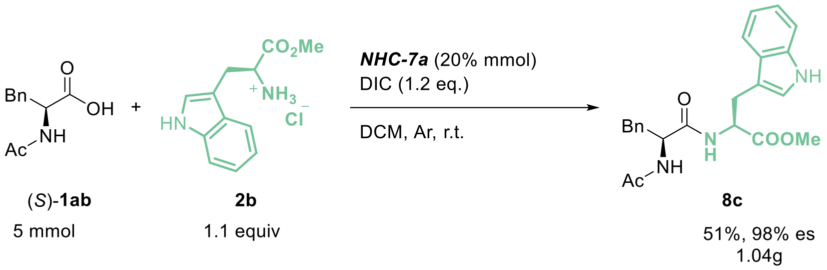

In a glove box under an argon atmosphere, the NHC precursor was pre-activated with NaH (1.2 equiv) for 1 h. After filtration, the resulting solution of free NHC-**7a** (1 mmol, 20 mol%) was added to a reaction mixture containing (*S*)-**1ab** (5 mmol) and amine **2b** (6 mmol, 1.2 equiv) in an oven-dried 100 mL Schlenk bottle equipped with a magnetic stir bar. Dry dichloromethane (DCM, 70 mL) and DIC (0.55 mmol, 1.1 equiv.) were subsequently added inside the glove box. The tube was sealed with a screw cap, and the mixture was stirred vigorously at room temperature (25°C) for 7 hours. Upon complete consumption of the α-amino acid (*S*)-**1ab** (as monitored by TLC), the crude reaction mixture was concentrated under reduced pressure. The reaction mixture was washed with H_2O, then extracted with EtOAc (3×20 mL), and the combined organic layer was dried over Na_2SO_4, filtered, and concentrated under reduced pressure. The residue was subjected to column chromatography directly using petroleum ether /EtOAc/ DCM (most commonly 1:1:0.5) as the eluent to afford the desired product **8c** (1.04g, 51%, 98% *es*).

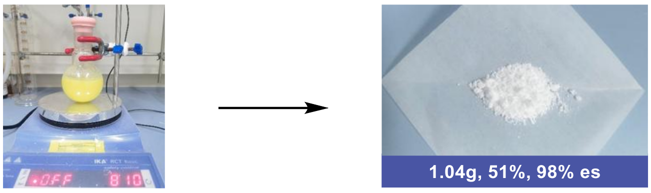

Figure S8 General application of the synthesis of peptides without racemization by NHC.[a]

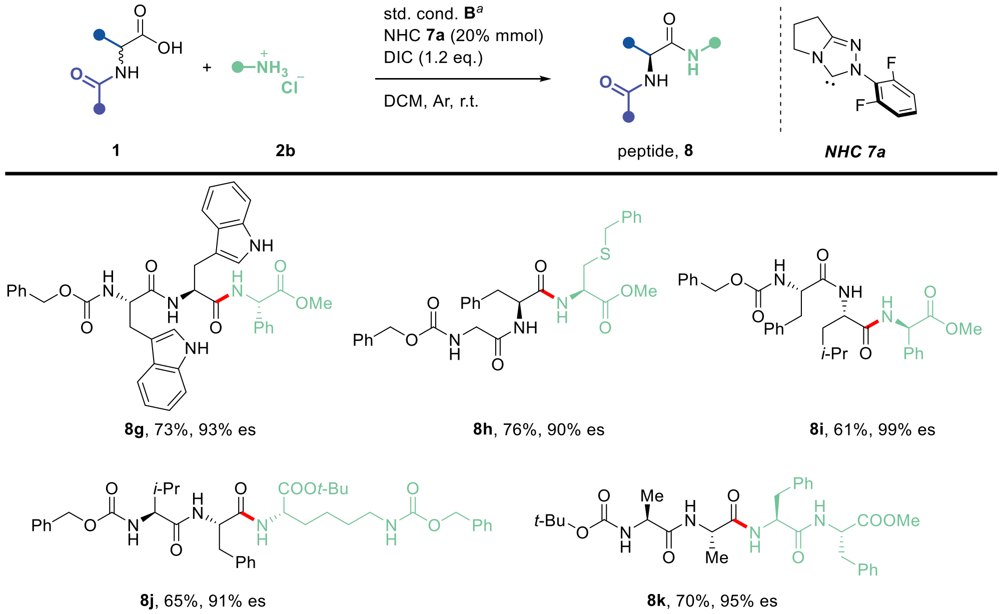

[a] Reaction conditions: A mixture of chiral AA **1** (0.05 mmol), amine **2b** (0.055 mmol), NHC-**7a** (20 mol%) and DIC (0.06 mmol) dissolved in DCM (0.05 M) was stirred at r.t. for 3-6 hours. Isolated yields are presented. Chiral HPLC determined es values.

Figure S9 The control experiment of the influence of NHC-7a on resistant racemization.

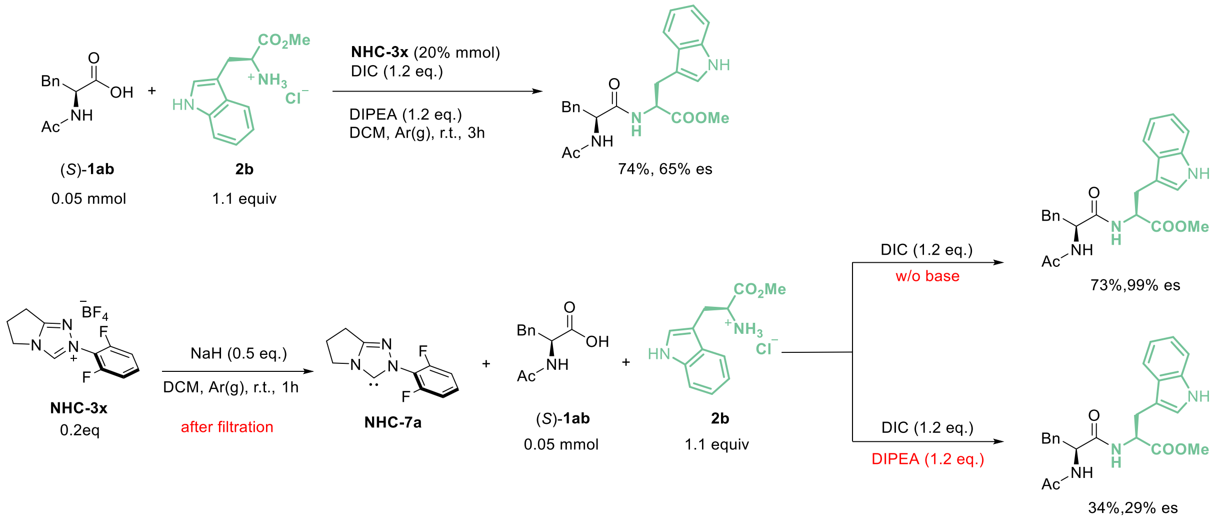

Three experiments were conducted to illustrate the importance of NHC-**7a** in racemization-free peptide synthesis, where DIC was used as an activated reagent for these processes.

To the solution, added (*S*)-**1ab** (0.05 mmol), amine **2b** (0.055 mmol, 1.1 equiv.), **NHC-3x** (0.01 mmol), DIPEA (0.025 mmol), DIC (0.06 mmol) in the glove box. DCM (1 mL) was then added, and the reaction mixture was stirred for 3 hours at room temperature (23-26 °C) under Ar(g). After completion of the reaction, monitored by TLC, the reaction mixture was concentrated under reduced pressure, and the residue was subjected to column chromatography directly using a petroleum ether/ethyl acetate (2:1 to 1:1) eluent to afford the desired product **8c**.

In a glove box under an argon atmosphere, the NHC precursor was pre-activated with NaH (1.2 equiv.) for 1 h. After filtration, the resulting solution of free NHC-**7a** (0.01 mmol, 20 mol%) was added to a reaction mixture containing α-amino acid **1ab** (0.05 mmol) and amine **2b** (0.055 mmol, 1.1 equiv.) in an oven-dried 5 mL Schlenk tube equipped with a magnetic stir bar. Dry dichloromethane (DCM, 1 mL) and DIC (0.06 mmol) were subsequently added inside the glove box. Under the above two conditions: without base and with added DIPEA (1.2 eq.), respectively, stirring for 12 hours, and then the yield and es of **8c** were detected.

Figure S10 General process of synthesis of peptides directly via DIC.

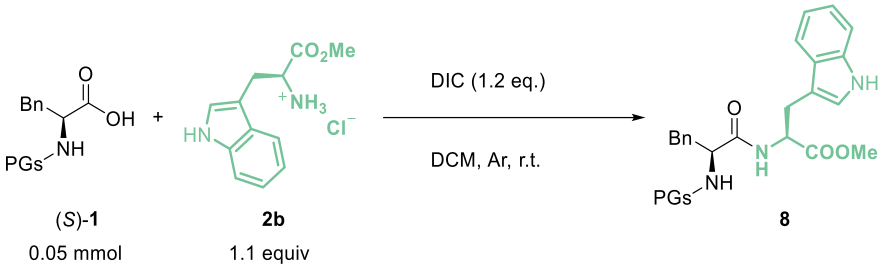

In an oven-dried 5 mL Schlenk tube equipped with a magnetic stir bar, α-amino acids **1** (0.05 mmol) and amine **2b** (0.055 mmol, 1.1 equiv.) were added, followed by 1 mL of dry dichloromethane (DCM). The resulting mixture was degassed and backfilled with argon (3 cycles), after which DIC (0.06 mmol) was added. The tube was sealed with a screw cap, and the reaction mixture was stirred vigorously at room temperature (25°C) for 10 hours. Upon completion (as monitored by TLC), the crude mixture was purified by flash column chromatography (petroleum ether/ethyl acetate = 2:1 to 1:1) to afford the corresponding ester products **8**.

methyl benzoyl-L-phenylalanyl-L-tryptophanate **8b**

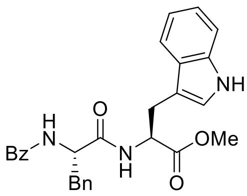

Following **General Procedure B** using α-amino acid **1aa** (14 mg, 0.05 mmol, 1.0 equiv.) amine **2b** (13 mg, 0.055 mmol, 1.1 equiv.) and NaOAc (50% mmol), the crude product was purified by column chromatography petroleum ether/EtOAc (most use 3/1) as eluent to afford the desired product **8b** as a white solid in 74% yield (17.5 mg). **m.p.** 157-158 °C.

TLC (2:1, PE: EtOAc): R_f = 0.5

^{1}H NMR (400 MHz, Acetone-*d*$_6$) δ 10.1 (d, *J* = 12.5 Hz, 1H), 7.5 (t, *J* = 8.6 Hz, 3H), 7.4 – 7.3 (m, 1H), 7.2 – 7.0 (m, 11H), 6.6 (s, 1H), 4.6 (ddt, *J* = 11.5, 7.6, 6.2 Hz, 1H), 4.3 – 4.1 (m, 1H), 3.6 (d, *J* = 13.7 Hz, 3H), 3.1 – 2.9 (m, 3H), 2.8 (ddd, *J* = 25.3, 13.9, 8.5 Hz, 1H).

^{13}C NMR (101 MHz, Acetone-*d*$_6$) δ 171.7, 170.3, 169.9, 142.8, 138.0, 136.9, 136.8, 129.5, 129.3, 128.1, 126.8, 126.4, 123.9, 123.8, 121.3, 118.8, 118.3, 111.4, 109.3, 58.0, 53.2, 51.4, 38.9, 27.7.

HRMS (ESI-TOF) *m/z:* $[M+Na]^+$ Calcd for $C_{28}H_{27}N_3NaO_4^+$ 492.1899, found 492.1901.

HPLC (Chiralpak-IA column, Hexane/^{i}PrOH = 70/30, flow rate: 1.0 mL/min, wavelength = 250 nm) $t_{(major)}$ = 8.052 min; $t_{(minor)}$ = 6.464 min, indicated 81% es.

$[\alpha]_D^{28}$ = +14 (c = 2 in $CHCl_3$).

methyl acetyl-L-phenylalanyl-L-tryptophanate **8c**

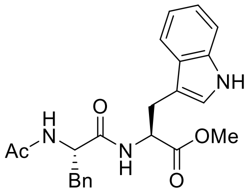

Following **General Procedure B** using α-amino acid **1ab** (10 mg, 0.05 mmol, 1.0 equiv.) and amine **2b** (13 mg, 0.055 mmol, 1.1 equiv.), the crude product was purified by column chromatography petroleum ether/EtOAc (most use 2/1) as eluent to afford the desired product **8c** as a white solid in 73% yield (14.9 mg). **m.p.** 182-183 °C.

TLC (1:1, PE: EtOAc): R_f = 0.3

^{1}H NMR (400 MHz, Methanol-*d*$_4$) δ 7.1 (d, *J* = 7.8 Hz, 1H), 7.0 (d, *J* = 8.1 Hz, 1H),

6.8 – 6.7 (m, 4H), 6.7 – 6.6 (m, 3H), 6.6 (s, 1H), 4.5 (s, 6H), 4.4 (dd, *J* = 7.8, 5.6 Hz, 1H), 4.3 (dd, *J* = 8.5, 5.9 Hz, 1H), 2.9 – 2.7 (m, 2H), 2.6 (dd, *J* = 13.8, 5.9 Hz, 1H), 2.4 (dd, *J* = 13.8, 8.5 Hz, 1H), 1.5 (s, 3H).

^{13}C NMR (101 MHz, Methanol-*d*$_4$) δ 172.3, 171.9, 171.6, 136.9, 136.7, 128.9, 127.9, 127.2, 126.3, 123.2, 121.1, 118.5, 117.7, 111.0, 108.9, 54.4, 53.2, 51.3, 37.5, 27.0, 21.0.

HRMS (ESI-TOF) *m/z:* $[M+Na]^+$ Calcd for $C_{23}H_{25}N_3NaO_4^+$ 430.1743, found 430.1743.

HPLC (Chiralpak-IA column, Hexane/^{i}PrOH = 70/30, flow rate: 1.0 mL/min, wavelength = 250 nm) $t_{(major)}$ = 5.947 min; $t_{(minor)}$ = 4.972 min, indicated 99% es.

$[\alpha]_D^{28}$ = +31.5 (c = 0.9 in $CHCl_3$).

methyl tosyl-L-phenylalanyl-L-tryptophanate **8d**

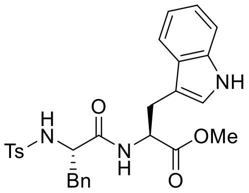

Following **General Procedure B** using α-amino acid **1av** (17.5 mg, 0.05 mmol, 1.0 equiv.) and amine **2b** (13 mg, 0.055 mmol, 1.1 equiv.), the crude product was purified by column chromatography petroleum ether/EtOAc (most use 3/1) as eluent to afford the desired product **8d** as a white solid in 90% yield (23.4 mg). **m.p.** 69-70 °C.

TLC (3:1, PE: EtOAc): R_f = 0.4

^{1}H NMR (400 MHz, Acetone-*d*$_6$) δ 10.1 (s, 1H), 7.8 – 7.7 (m, 4H), 7.6 – 7.5 (m, 2H), 7.4 – 7.3 (m, 4H), 7.3 – 7.0 (m, 7H), 7.0 – 7.0 (m, 1H), 5.0 – 4.9 (m, 1H), 4.8 (dt, *J* = 13.2, 6.9 Hz, 1H), 3.6 (d, *J* = 7.6 Hz, 3H), 3.3 – 3.2 (m, 3H), 3.1 – 3.0 (m, 1H).

^{13}C NMR (101 MHz, Acetone-*d*$_6$) δ 172.0, 170.9, 166.5, 137.8, 136.7, 134.5, 131.3, 129.3, 128.3, 128.1, 127.2, 126.3, 123.8, 121.3, 118.8, 118.2, 111.4, 109.6, 54.7, 53.1, 51.4, 37.5, 27.5, 21.5.

HRMS (ESI-TOF) *m/z:* $[M+Na]^+$ Calcd for $C_{28}H_{29}N_3NaO_5S^+$ 542.1726, found 542.1726.

HPLC (Chiralpak-IA column, Hexane/^{i}PrOH = 80/20, flow rate: 1.0 mL/min, wavelength = 250 nm) $t_{(major)}$ = 14.173 min; $t_{(minor)}$ = 15.120 min, indicated 99% es.

$[\alpha]_D^{28}$ = +61.7 (c = 1.5 in $CHCl_3$).

methyl ((benzyloxy)carbonyl)-L-phenylalanyl-L-tryptophanate **8e**

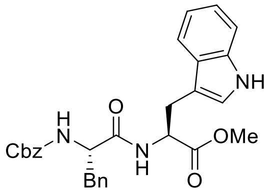

Following **General Procedure B** using α-amino acid **1aw** (16 mg, 0.05 mmol, 1.0 equiv.) and amine **2b** (13 mg, 0.055 mmol, 1.1 equiv.), the crude product was purified by column chromatography petroleum ether/EtOAc (most use 5/1) as eluent to afford the desired product **8e** as a white solid in 98% yield (24.5 mg). **m.p.** 162-163 °C.

TLC (4:1, PE: EtOAc): R_f = 0.5

^{1}H NMR (400 MHz, Acetone-*d*$_6$) δ 10.1 (s, 1H), 7.6 (t, *J* = 6.8 Hz, 2H), 7.4 (d, *J* = 8.0 Hz, 1H), 7.3 – 7.2 (m, 5H), 7.2 – 7.1 (m, 6H), 7.1 – 7.0 (m, 2H), 6.4 (d, *J* = 8.5 Hz, 1H), 5.1 – 4.9 (m, 2H), 4.8 – 4.7 (m, 1H), 4.5 (td, *J* = 8.2, 4.6 Hz, 1H), 3.6 (s, 3H), 3.3 – 3.2 (m, 2H), 2.9 – 2.8 (m, 2H).

^{13}C NMR (101 MHz, Acetone-*d*$_6$) δ 172.0, 170.9, 155.9, 137.6, 137.3, 136.7, 129.4, 128.3, 128.1, 127.7, 127.6, 127.6, 126.3, 123.8, 121.4, 118.8, 118.3, 111.4, 109.6, 65.8, 56.2, 53.0, 51.5, 38.0, 27.5.

HRMS (ESI-TOF) *m/z:* $[M+Na]^+$ Calcd for $C_{29}H_{29}N_3NaO_5^+$ 522.2005, found 522.2005.

HPLC (Chiralpak-IA column, Hexane/^{i}PrOH = 90/10, flow rate: 1.0 mL/min, wavelength = 250 nm) $t_{(major)}$ = 42.324 min; $t_{(minor)}$ = 39.870 min, indicated 99% es.

$[\alpha]_D^{28}$ = +54 (c = 1.5 in $CHCl_3$).

methyl (tert-butoxycarbonyl)-L-phenylalanyl-L-tryptophanate **8f**

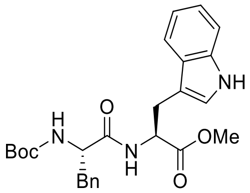

Following **General Procedure B** using α-amino acid **1ax** (15 mg, 0.05 mmol, 1.0 equiv.) and amine **2b** (13 mg, 0.055 mmol, 1.1 equiv.), the crude product was purified by column chromatography petroleum ether/EtOAc (most use 5/1) as eluent to afford the desired product **8f** as a white solid in 97% yield (22.6 mg). **m.p.** 159-160 °C.

TLC (5:1, PE: EtOAc): R_f = 0.5

^{1}H NMR (400 MHz, Acetone-*d*$_6$) δ 10.1 (s, 1H), 7.5 (d, *J* = 7.9 Hz, 1H), 7.5 (d, *J* = 7.1 Hz, 1H), 7.4 (d, *J* = 8.0 Hz, 1H), 7.2 (d, *J* = 4.3 Hz, 4H), 7.2 – 7.2 (m, 2H), 7.1 (ddd, *J* = 8.2, 7.0, 1.3 Hz, 1H), 7.0 (td, *J* = 7.5, 7.0, 1.1 Hz, 1H), 6.0 (d, *J* = 8.6 Hz, 1H), 4.9 – 4.8 (m, 1H), 4.4 (td, *J* = 8.8, 4.9 Hz, 1H), 3.6 (s, 3H),

3.3 (t, J = 5.5 Hz, 2H), 2.9 (d, J = 5.7 Hz, 2H), 1.3 (s, 9H).
^{13}C NMR (101 MHz, Acetone-d_6) δ 171.9, 171.2, 155.3, 137.9, 136.6, 129.4, 128.1, 127.7, 126.3, 123.7, 121.3, 118.8, 118.3, 111.3, 109.5, 78.5, 55.7, 53.1, 51.4, 37.8, 27.6.

HRMS (ESI-TOF) *m/z:* $[M+Na]^+$ Calcd for $C_{26}H_{31}N_3NaO_5^+$ 488.2161, found 488.2163.

HPLC (Chiralpak-IA column, Hexane/^{i}PrOH = 90/10, flow rate: 1.0 mL/min, wavelength = 250 nm) $t_{(major)}$ = 25.107 min; $t_{(minor)}$ = 27.647 min, indicated 99% es.

$[\alpha]_D^{28}$ = +54.1 (c = 1 in $CHCl_3$).

methyl (5S,8S,11S)-5,8-bis((1H-indol-3-yl)methyl)-3,6,9-trioxo-1,11-diphenyl-2-oxa-4,7,10-triazadodecan-12-oate **8g**

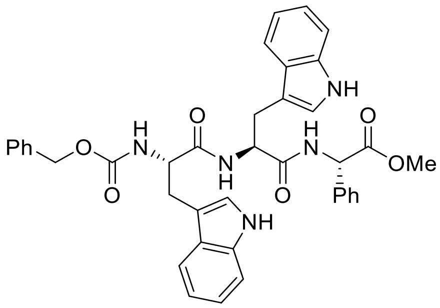

Following **General Procedure B** using Z-Trp-Trp-OH (26 mg, 0.05 mmol, 1.0 equiv.) and H-L-Phg-OMe·HCl (0.055 mmol, 1.1 equiv.), the crude product was purified by column chromatography DCM/Acetone (most use 30/1) as eluent to afford the desired product **8g** as a white solid in 73% yield (24.5 mg). **m.p.** 163-164 ℃.

TLC (2:0.1, DCM: Acetone): R_f = 0.4

^{1}H NMR (400 MHz, Acetone-d_6) δ 10.1 (d, J = 9.0 Hz, 2H), 7.9 (d, J = 7.1 Hz, 1H), 7.6 (d, J = 7.9 Hz, 1H), 7.5 (dd, J = 24.2, 7.9 Hz, 2H), 7.4 – 7.2 (m, 12H), 7.2 – 7.1 (m, 4H), 7.0 (dt, J = 11.6, 7.3 Hz, 2H), 6.3 (d, J = 7.5 Hz, 1H), 5.5 (d, J = 7.1 Hz, 1H), 4.9 – 4.8 (m, 3H), 4.5 (td, J = 7.8, 5.0 Hz, 1H), 3.7 (s, 3H), 3.2 (dtd, J = 25.1, 14.7, 5.8 Hz, 4H).

^{13}C NMR (101 MHz, Acetone-d_6) δ 171.5, 171.0, 170.7, 156.1, 137.0, 136.7, 136.6, 128.7, 128.3, 128.2, 127.8, 127.7, 127.6, 123.8, 123.8, 121.3, 121.2, 118.7, 118.5, 118.5, 111.3, 111.2, 110.3, 109.9, 65.9, 56.7, 55.9, 54.1, 53.5, 51.8, 31.4, 27.7, 27.5, 22.4, 13.5.
HPLC (IA-30%) 7.518, 11.430

HRMS (ESI-TOF) *m/z:* $[M+Na]^+$ Calcd for $C_{39}H_{37}N_5NaO_6^+$ 694.2636, found 694.2637.

HPLC (Chiralpak-IA column, Hexane/^{i}PrOH = 70/30, flow rate: 1.0 mL/min, wavelength = 228 nm) $t_{(major)}$ = 7.518 min; $t_{(minor)}$ = 11.480 min, indicated 93% es.

$[\alpha]_D^{28}$ = +72.6 (c = 0.4 in $CHCl_3$).

methyl S-benzyl-N-((benzyloxy)carbonyl)glycyl-L-phenylalanyl-L-cysteinate **8h**

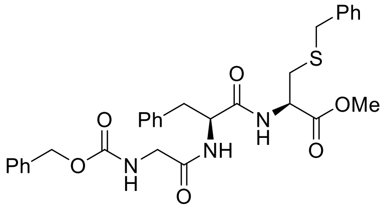

Following **General Procedure B** using Cbz-Gly-Phe-OH (18 mg, 0.05 mmol, 1.0 equiv.) and H-Cys(Bzl)-OMe·HCl (0.055 mmol, 1.1 equiv.), the crude product was purified by column chromatography, petroleum ether / EtOAc / DCM (most use 20:10:15) as eluent to afford the desired product **8h** as a yellow oil in 76% yield (21.3 mg).

TLC (2:1:1, PE : EA : DCM) : R_f = 0.5

^{1}H NMR (400 MHz, Acetone-*d*$_6$) δ 7.9 (d, *J* = 8.0 Hz, 1H), 7.6 (d, *J* = 8.2 Hz, 1H), 7.4 – 7.2 (m, 15H), 6.7 (t, *J* = 5.7 Hz, 1H), 5.1 (s, 2H), 4.8 (td, *J* = 8.1, 5.3 Hz, 1H), 4.7 (q, *J* = 7.1 Hz, 1H), 3.8 – 3.8 (m, 5H), 3.7 (s, 2H), 3.0 (dd, *J* = 13.9, 8.1 Hz, 2H), 2.9 – 2.7 (m, 2H).

^{13}C NMR (101 MHz, Acetone-*d*$_6$) δ 170.9, 170.8, 169.1, 156.7, 138.3, 137.4, 137.2, 129.5, 129.1, 128.4, 128.4, 128.2, 127.8, 126.9, 126.4, 66.1, 59.7, 54.0, 52.1, 51.8, 44.1, 37.8, 35.8, 32.5.

HRMS (ESI-TOF) *m/z:* $[M+Na]^+$ Calcd for $C_{30}H_{33}N_3NaO_6^+$ 586.1982, found 586.1982.

HPLC (Chiralpak-IA column, Hexane/^{i}PrOH = 70/30, flow rate: 1.0 mL/min, wavelength = 250 nm) $t_{(major)}$ = 11.078 min; $t_{(minor)}$ = 5.405 min, indicated 90% es.

$[\alpha]_D^{28}$ = +19.8 (c = 0.8 in $CHCl_3$).

methyl (5S,8S,11R)-5-benzyl-8-isobutyl-3,6,9-trioxo-1,11-diphenyl-2-oxa-4,7,10-triazadodecan-12-oate **8i**

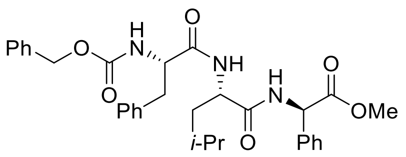

Following **General Procedure B** using Z-Phe-Leu-OH (20 mg, 0.05 mmol, 1.0 equiv.) and H-D-Phg-OMe·HCl (0.055 mmol, 1.1 equiv.), the crude product was purified by

column chromatography petroleum ether / EtOAc / DCM (most use 20:10:15) as eluent to afford the desired product **8i** as a colorless oil in 61% yield (17 mg).

TLC (2:1:1, PE : EA:DCM) : R_f = 0.5

^{1}H NMR (400 MHz, Acetone-*d*$_6$) δ 8.1 (d, *J* = 7.1 Hz, 1H), 7.6 (dd, *J* = 12.4, 8.3 Hz, 1H), 7.5 – 7.1 (m, 16H), 6.6 (dd, *J* = 11.6, 8.4 Hz, 1H), 5.5 (dd, *J* = 10.1, 7.2 Hz, 1H), 5.1 – 4.9 (m, 2H), 4.7 – 4.4 (m, 2H), 3.7 (d, *J* = 7.4 Hz, 3H), 1.7 (dtd, *J* = 21.8, 8.7, 7.6, 5.1 Hz, 2H), 1.6 (qd, *J* = 6.0, 5.5, 3.6 Hz, 1H), 0.9 (ddd, *J* = 16.9, 10.4, 6.0 Hz, 6H).

^{13}C NMR (101 MHz, Acetone-*d*$_6$) δ 171.6, 171.2, 170.8, 156.0, 137.7, 137.2, 136.8, 136.6, 129.3, 128.7, 128.3, 128.2, 127.7, 127.5, 126.4, 65.8, 56.6, 56.3, 51.8, 51.2, 41.1, 37.8, 24.3, 22.6, 21.3.

HRMS (ESI-TOF) *m/z:* $[M+Na]^+$ Calcd for $C_{32}H_{37}N_3NaO_6^+$ 582.2575, found 582.2575.

HPLC (Chiralpak-IA column, Hexane/^{i}PrOH = 70/30, flow rate: 1.0 mL/min, wavelength = 250 nm) $t_{(major)}$ = 5.369 min; $t_{(minor)}$ = 6.538 min, indicated 99% es.

$[\alpha]_D^{28}$ = +30.2 (c = 0.2 in $CHCl_3$).

tert-butyl N6-((benzyloxy)carbonyl)-N2-((benzyloxy)carbonyl)-L-valyl-L-phenylalanyl-L-lysinate **8j**

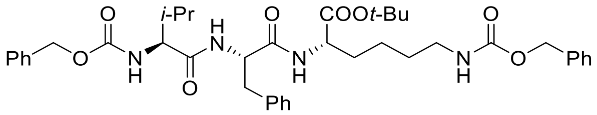

Following **General Procedure B** using Cbz-Val-Phe-OH (18 mg, 0.05 mmol, 1.0 equiv.) and H-Cys(Bzl)-OMe·HCl (0.055 mmol, 1.1 equiv.), the crude product was purified by column chromatography, petroleum ether / EtOAc / DCM (most use 20:10:15) as eluent to afford the desired product **8j** as a white solid in 65% yield (35.7 mg). **m.p.** 97-98 ℃.

TLC (2:1:1, PE : EA:DCM) : R_f = 0.5

^{1}H NMR (400 MHz, Acetone-*d*$_6$) δ 7.6 (d, *J* = 8.1 Hz, 1H), 7.5 (d, *J* = 7.8 Hz, 1H), 7.4 – 7.1 (m, 16H), 6.4 (dd, *J* = 22.6, 7.1 Hz, 2H), 5.2 – 5.0 (m, 5H), 4.8 (td, *J* = 8.5, 5.2 Hz, 1H), 4.3 (h, *J* = 4.9 Hz, 1H), 4.1 (dd, *J* = 8.5, 6.2 Hz, 1H), 3.9 – 3.7 (m, 1H), 3.2 (dtt, *J* = 16.2, 6.5, 4.1 Hz, 3H), 1.9 – 1.8 (m, 1H), 1.7 (td, *J* = 14.1, 7.1 Hz, 1H), 1.5 (d, *J* = 9.0 Hz, 13H), 0.9 (dd, *J* = 18.3, 6.9 Hz, 6H).

^{13}C NMR (101 MHz, Acetone-*d*$_6$) δ 171.3, 171.0, 170.7, 156.4, 137.7, 137.6, 137.2, 129.4, 129.3, 128.4, 128.3, 128.3, 128.2, 127.8, 127.7, 126.4, 80.8, 66.0, 65.5, 60.5,

54.1, 52.8, 41.2, 40.3, 37.6, 31.5, 30.8, 27.3, 22.8, 22.5, 18.8, 17.3.

HRMS (ESI-TOF) *m/z:* $[M+Na]^+$ Calcd for $C_{40}H_{52}N_4NaO_8^+$ 739.3677, found 739.3677.

HPLC (Chiralpak-IA column, Hexane/^{i}PrOH = 70/30, flow rate: 1.0 mL/min, wavelength = 250 nm) $t_{(major)}$ = 5.064 min; $t_{(minor)}$ = 16.396 min, indicated 91% es.

$[\alpha]_D^{28}$ = +35.6 (c = 0.8 in $CHCl_3$).

methyl (tert-butoxycarbonyl)-L-alanyl-L-alanyl-L-phenylalanyl-L-phenylalaninate--methane **8k**

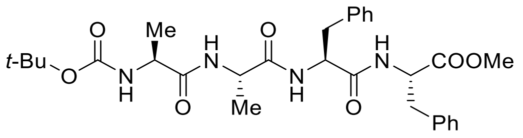

Following **General Procedure B** using Boc-Ala-Ala-OH (13 mg, 0.05 mmol, 1.0 equiv.) and H-Phe-Phe-OMe.HCl (0.055 mmol, 1.1 equiv.), the crude product was purified by column chromatography using DCM/Acetone (most commonly 30/1) as the eluent to afford the desired product **8k** as a white solid in 70% yield (21 mg). **m.p.** 166-167 °C.

TLC (2:0.1, DCM : Acetone) : R_f = 0.5

^{1}H NMR (400 MHz, Acetone-*d*$_6$) δ 7.8 – 7.4 (m, 3H), 7.2 (qq, *J* = 15.3, 7.1, 6.6 Hz, 10H), 6.3 (dd, *J* = 27.8, 6.4 Hz, 1H), 4.7 (dd, *J* = 8.5, 4.2 Hz, 2H), 4.3 (s, 1H), 4.1 (s, 1H), 3.6 (s, 3H), 3.3 – 2.8 (m, 8H), 1.5 – 1.1 (m, 17H), 0.9 (s, 1H).

^{13}C NMR (101 MHz, Acetone-*d*$_6$) δ 173.2, 172.7, 171.9, 171.5, 170.7, 155.9, 137.7, 137.1, 129.3, 129.2, 128.3, 128.3, 128.1, 126.6, 126.3, 78.8, 54.1, 53.9, 53.8, 51.4, 50.7, 49.4, 49.0, 37.4, 27.7, 17.4.

HRMS (ESI-TOF) *m/z:* $[M+Na]^+$ Calcd for $C_{32}H_{48}N_4NaO_7^+$ 623.3415, found 623.3415.

HPLC (Chiralpak-IA column, Hexane/^{i}PrOH = 70/30, flow rate: 1.0 mL/min, wavelength = 250 nm) $t_{(major)}$ = 5.640 min; $t_{(minor)}$ = 6.064 min, indicated 95% es.

$[\alpha]_D^{28}$ = +78.5 (c = 0.6 in $CHCl_3$).

Figure S11 Application of the general process in the synthesis of short peptide **8a**

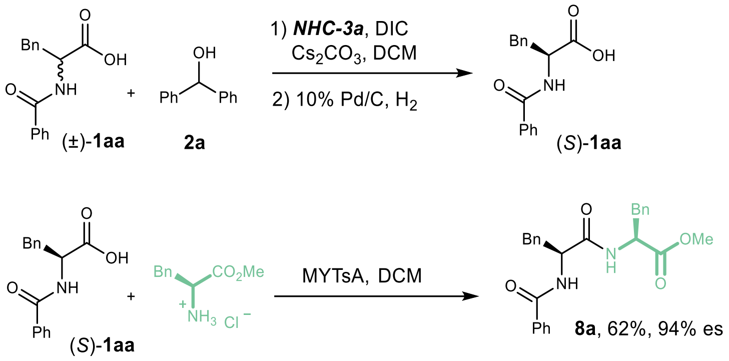

Step 1:

To a 10 mL flame-dry Schlenk reaction tube equipped with a magnetic stir bar, was added Ac-Phe-OH **1aa** (0.1 mmol), alcohol **2a** (0.24 mmol, 22 mg), **NHC-3a** (0.02 mmol, 10.5 mg), Cs_2CO_3 (0.1 mmol, 32.0 mg), DIC (0.12 mmol, 20.0 μL) at the glove box. DCM (3 mL) was then added, and the reaction mixture was stirred for 12 hours at room temperature (23-26 °C) under N_2. After completion of the reaction, monitored by TLC, the reaction mixture was concentrated under reduced pressure, and the residue was subjected to column chromatography directly using petroleum ether /EtOAc (most commonly 5/1) as the eluent to afford the desired product **4aa** with a yield of 91% and an enantiomeric excess (ee) of 99%.

Then, the desired product was dissolved in EtOAc (3 mL). To this solution, 10% Pd/C was added, cooled to 0 ºC, and stirred overnight under H_2(g). After the reaction was completed, the Pd/C catalyst was removed by filtration. The reaction mixture was concentrated under reduced pressure and purified by C18 column chromatography using MeCN/H_2O (most commonly 6:1) as the eluent to afford the desired product (*S*)-**1aa**.

Step 2:[5]

To a 10 mL flame-dry Schlenk reaction tube equipped with a magnetic stir bar, (*S*)-**1aa**, N-methylnaphthalenesulfonamide (MYTsA) (0.12 mmol, 16 mg) was added. DCM (2 mL) was then added, and the reaction mixture was stirred at 30°C until the MYTsA was consumed. The solvent was removed under vacuum, and 1 mL DMF was added to the reaction mixture. H-L-Phe-OMe.HCl (0.11 mmol, 24 mg) was added to the reaction mixture, which was stirred at room temperature until the α-acyloxyenamide active ester was fully consumed. After the reaction, the mixture was concentrated and purified by silica gel chromatography directly using petroleum ether /EtOAc (most commonly 4:1) to afford the dipeptide **8a** (m.p. 169-170 °C).

^{1}H NMR (400 MHz, Methanol-*d*$_4$) δ 7.7 (d, *J* = 7.4 Hz, 2H), 7.5 (t, *J* = 7.4 Hz, 1H), 7.4 (t, *J* = 7.6 Hz, 2H), 7.3 – 7.1 (m, 10H), 4.9 – 4.8 (m, 2H), 4.7 (dd, *J* = 8.3, 5.7 Hz, 1H), 3.7 (s, 3H), 3.2 (ddd, *J* = 17.6, 13.9, 5.7 Hz, 2H), 3.0 (ddd, *J* = 13.9, 8.7, 2.9 Hz, 2H).

^{13}C NMR (101 MHz, Methanol-*d*$_4$) δ 172.1, 171.7, 168.6, 137.1, 136.5, 133.8, 131.4, 128.9, 128.9, 128.1, 128.1, 128.0, 127.1, 126.5, 126.3, 54.9, 53.8, 51.3, 37.2, 37.1.

HRMS (ESI-TOF) *m/z:* $[M+Na]^+$ Calcd for $C_{26}H_{26}N_2NaO_4^+$ 453.1790, found 453.1790.

HPLC (Chiralpak-IC column, Hexane/^{i}PrOH = 80/20, flow rate: 1.0 mL/min, wavelength = 250 nm) $t_{(major)}$ = 6.569 min; $t_{(minor)}$ = 11.099 min, indicated 94% de.

methyl benzoylphenylalanylphenylalaninate

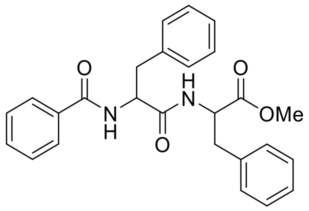

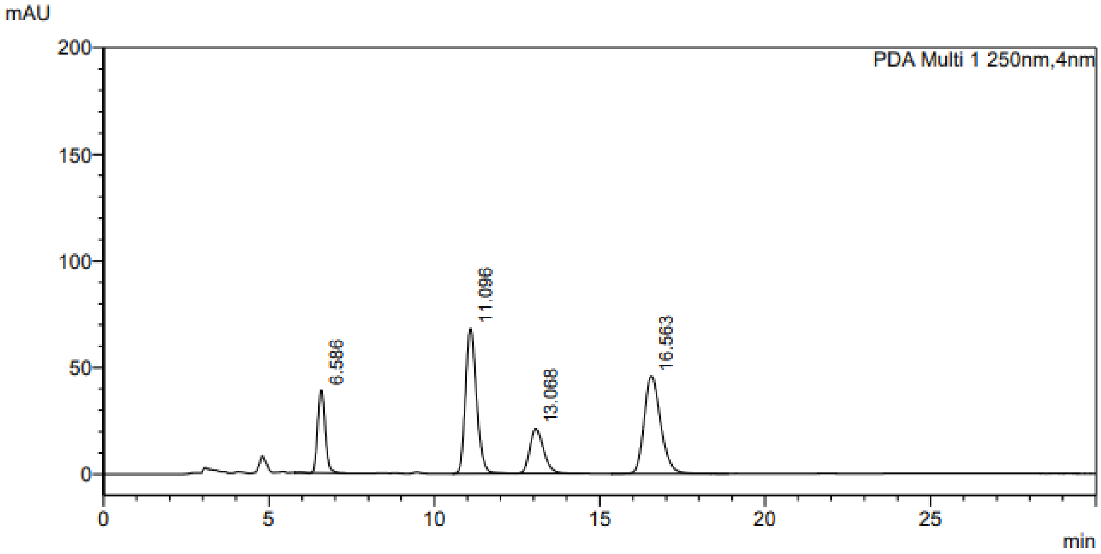

<Peak Table>

PDA Ch1 250nm

Peak#	Ret. Time	Area	Height	Area%	Height%
1	6.586	612763	38780	13.920	22.318
2	11.096	1587097	68214	36.053	39.258
3	13.068	614074	21025	13.949	12.100
4	16.563	1588224	45741	36.078	26.324
Total		4402158	173759	100.000	100.000

Figure S12. HPLC spectrum of racemic **8a**

methyl benzoylphenylalanyl-L-phenylalaninate

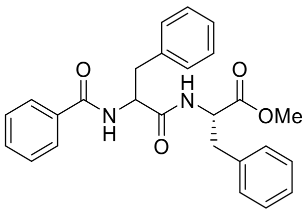

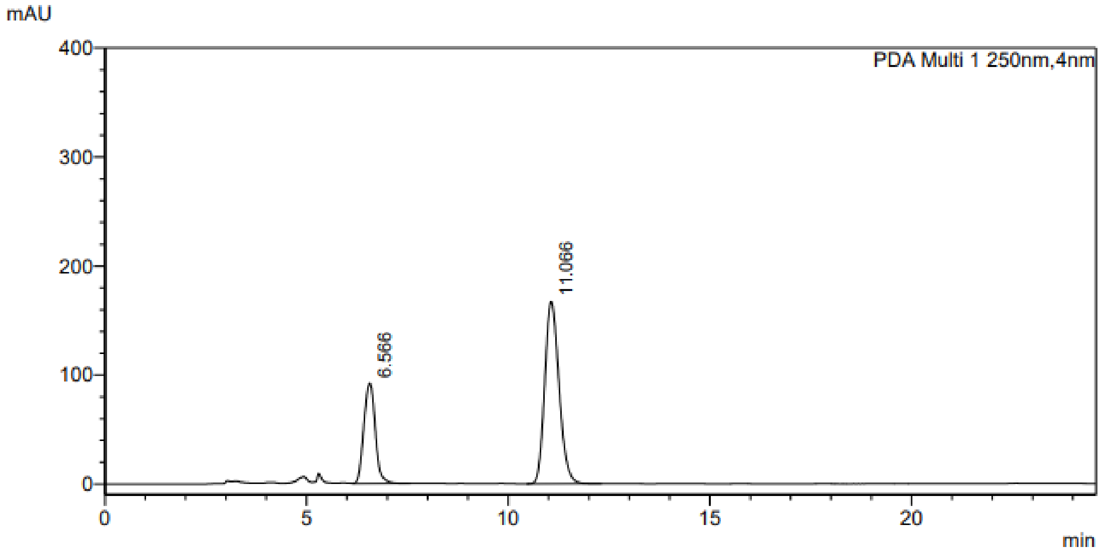

<Peak Table>

PDA Ch1 250nm

Peak#	Ret. Time	Area	Height	Area%	Height%
1	6.566	1823191	91946	30.676	35.522
2	11.066	4120135	166894	69.324	64.478
Total		5943326	258840	100.000	100.000

Figure S13. HPLC spectrum of (*RS*,2*S*)-**8a**

methyl benzoyl-L-phenylalanyl-L-phenylalaninate

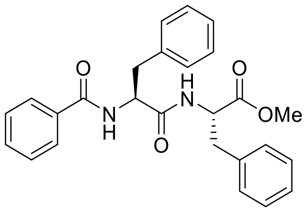

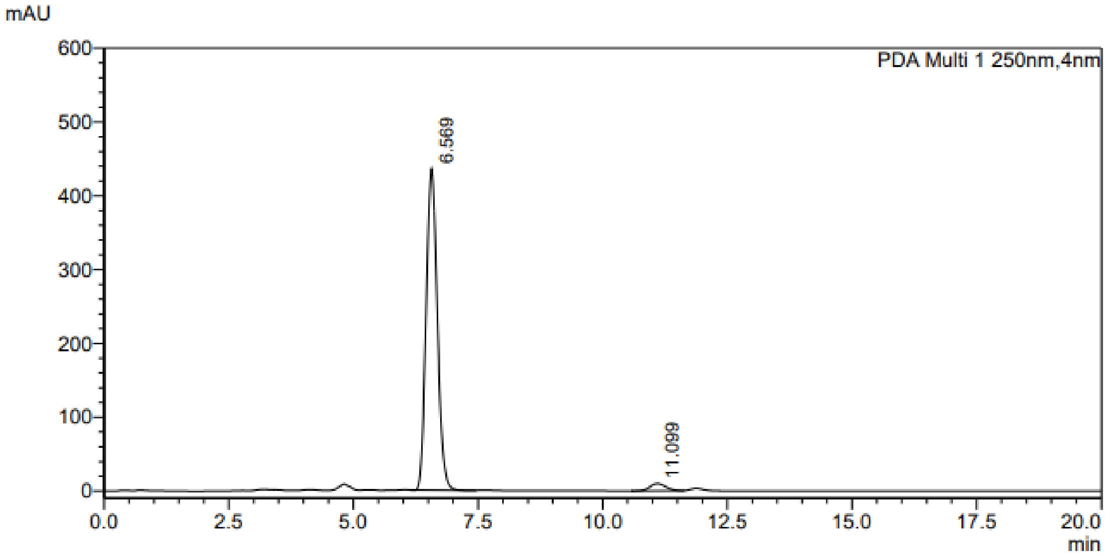

<Peak Table>

PDA Ch1 250nm

Peak#	Ret. Time	Area	Height	Area%	Height%
1	6.569	6804904	434968	96.967	97.841
2	11.099	212875	9600	3.033	2.159
Total		7017779	444568	100.000	100.000

Figure S14. HPLC spectrum of (*S*,*S*)**-8a**

Figure S15 Solid phase peptide synthesis applied in the synthesis of **8c**

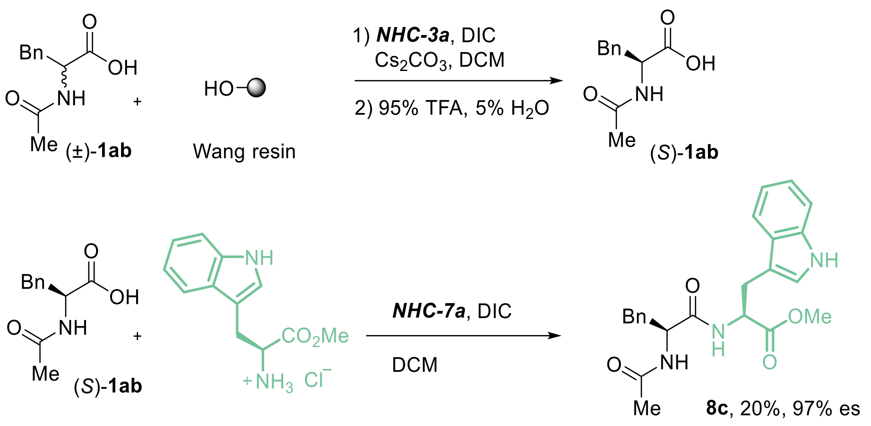

Step 1:

600 mg of Wang resin (0.58 mmol/g, 2 equiv) was placed in a 10 mL polypropylene syringe containing a porous polypropylene disc (Torviq). After swelling in DCM for 30 min, the resin was washed with DCM (3×). 2-Acetamido-3-phenylpropanoic acid (*Rac*)-**1ab** (0.300 mmol, 1 equiv) was dissolved in 0.025 M NHC-**3a** solution in DCM (5 mL, 0.15 mmol, 0.5 equiv), activated with DIC (0.09 mL, 0.6 mmol, 2 equiv) and Cs_2CO_3 (117 mg, 0.36 mmol, 1.2 equiv). The mixture was sonicated briefly, transferred to a fritted syringe containing the resin, and stirred for 6 hours in a glovebox under argon. The coupling solution was drained upon completion, and the resin was washed with DCM (2×) and DMF (2×). Then, the piperidine solution was drained, and the resin was washed with DCM (3×). The peptide was cleaved from the resin by treating the resin with a cleavage cocktail (3 mL) containing 98% TFA and 2% water twice at room temperature under air, each lasting 1 hour. The resin was removed by filtration, and the resulting cocktail solution was collected in a Falcon tube. Next, the solution was put under a gentle stream of nitrogen gas to evaporate the volatile solvents until a thin film was reached. The resulting crude peptide was precipitated by adding Et_2O to the Falcon tube at room temperature. The tube was then centrifuged at 3000 rpm for 5 min. The supernatant was removed, and the precipitated peptide was triturated three times with Et_2O. The resulting crude material was purified by C18 column chromatography using $MeCN/H_2O$ (most commonly 6:1) as the eluent to afford the desired product (*S*)-**1ab**.

Step 2:

To a 10 mL flame-dry Schlenk reaction tube equipped with a magnetic stir bar, was added (*S*)-**1ab**, amine (0.12 mmol, 11 mg), **NHC-7a** (0.01 mmol, 4.8 mg), DIC (0.06 mmol, 10.0 µL). DCM (1.5 mL) was added, and the reaction mixture was stirred for 2 hours at room temperature (23-26 °C). After completion of the reaction, monitored by

TLC, the reaction mixture was concentrated under reduced pressure, and the residue was subjected to column chromatography directly using petroleum ether/EtOAc (most commonly 2:1) as the eluent to afford the desired product **8c**.

HPLC (Chiralpak-IA column, Hexane/^{i}PrOH = 70/30, flow rate: 1.0 mL/min, wavelength = 250 nm) $t_{(major)}$ = 5.977 min; $t_{(minor)}$ = 4.993 min, indicated 97% de.

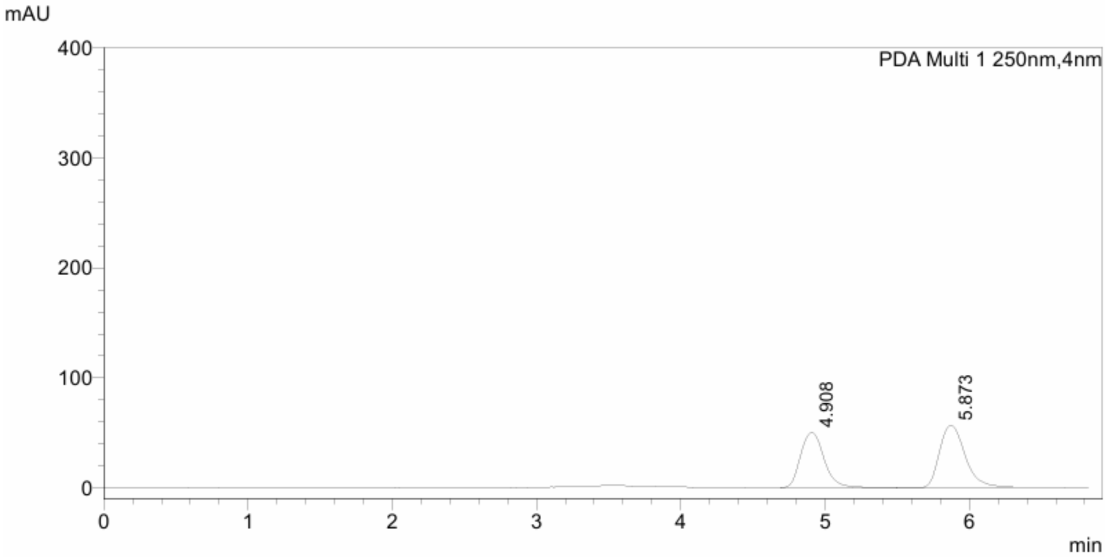

<Peak Table>

PDA Ch1 250nm

Peak#	Ret. Time	Area	Height	Area%	Height%
1	4.908	578417	50233	44.856	47.065
2	5.873	711076	56498	55.144	52.935
Total		1289493	106731	100.000	100.000

Figure S16. HPLC spectrum of racemic (*RS*,2*S*)-**8c**

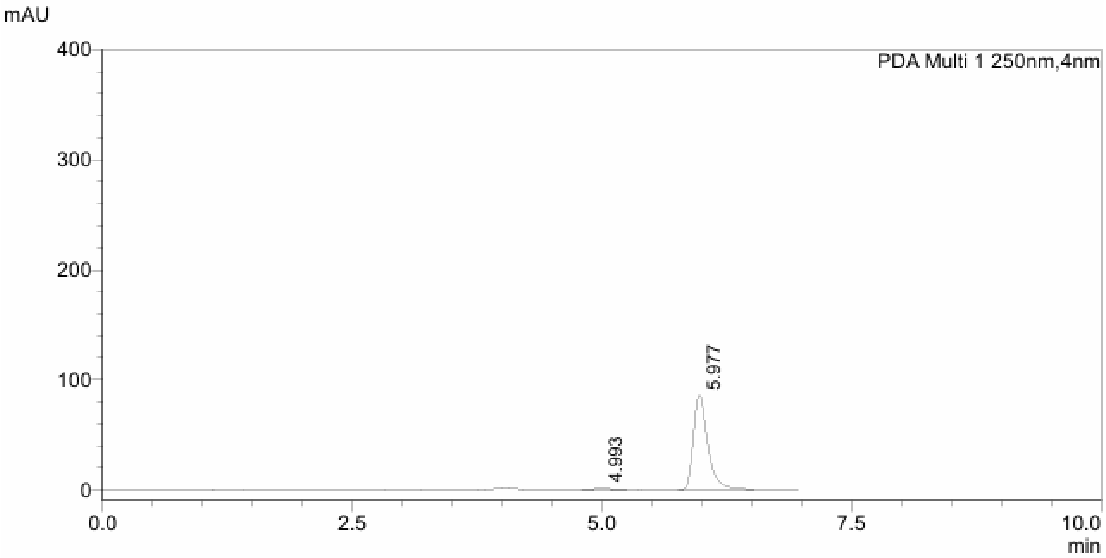

<Peak Table>

PDA Ch1 250nm

Peak#	Ret. Time	Area	Height	Area%	Height%
1	4.993	13775	1614	1.573	1.846
2	5.977	862052	85803	98.427	98.154
Total		875827	87417	100.000	100.000

Figure S17. HPLC spectrum of (*S*,*S*)-**8c**

Mechanism study

Figure S18 Deuteration experiments of the reaction

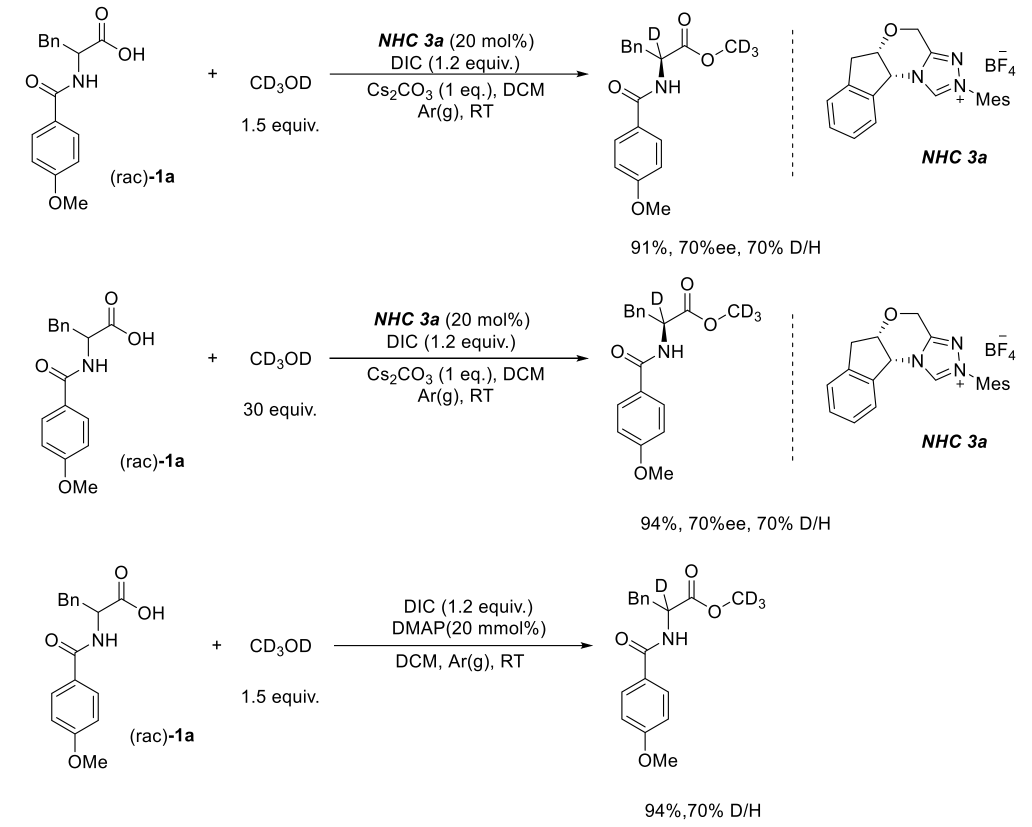

1a (0.05 mmol) and CD3OD were the initial substrates under these conditions. When NHC-**3a** was used as the catalyst, a different amount of CD3OD was added, and the deuteration result was the same, at only 70%. Then, **1a** and CD3OD, under the standard conditions of using DIC and DMAP, have the same result regarding deuteration. The experiments show that H-shuttling may not determine the chirality, but it appears in the reaction.

Determination of absolute configuration

Crystals suitable for analysis were obtained by vapor diffusion of cyclohexane into a methanol solution of the compound at room temperature over 4-6 days.

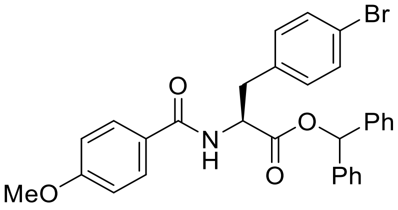

(**4i**)

The absolute configuration of **4i** was determined to be the (*S*)-configuration.

X-ray Crystal Structure for 4i (CCDC 2469893)

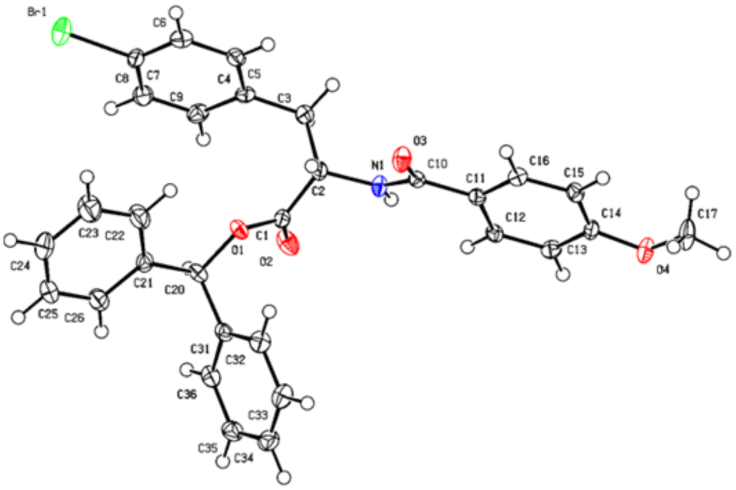

Crystal data and structure refinement for 4i.

Identification code	**4i** (CCDC 2469893)
Empirical formula	$C_{30}H_{26}BrNO_4$
Formula weight	544.43
Temperature/K	173.15
Crystal system	monoclinic
Space group	$P2_1$
a/Å	13.5267(11)
b/Å	5.2663(5)
c/Å	17.9386(16)
α/°	90
β/°	104.040(3)
γ/°	90
Volume/Å^3	1239.69(19)
Z	2

ρ_{calc} g/cm^3	1.458
μ/mm^{-1}	0.915
F (000)	560.0
Crystal size/mm^3	0.396 × 0.186 × 0.108
Radiation	Ag Kα (λ = 0.56086)
2Θ range for data collection/°	3.694 to 40.994
Index ranges	-16 ≤ h ≤ 16, -6 ≤ k ≤ 6, -20 ≤ l ≤ 22
Reflections collected	10264
Independent reflections	4993 [R_{int} = 0. 0334, R_{sigma} = 0. 0425]
Data/restraints/parameters	4993/1/326
Goodness-of-fit on F^2	1.058
Final R indexes [I>=2σ (I)]	R_1 = 0.0327, wR_2 = 0.0703
Final R indexes [all data]	R_1 = 0.0365, wR_2 = 0.0723
Largest diff. peak/hole / e Å^{-3}	0.31/-0.30
Flack parameter	0.002(6)

Table S5 Fractional Atomic Coordinates (×10^4) and Equivalent Isotropic Displacement Parameters (Å^2×10^3) for **4i**. Ueq is defined as 1/3 of the trace of the orthogonalised U_{IJ} tensor.

**Atom**	***x***	***y***	***z***	**U(eq)**
Br(1)	7510.4(3)	6637.7(9)	1977.8(2)	41.15(13)
O(1)	3089.1(16)	4862(4)	2334.2(11)	19.4(5)
O(2)	2716.8(19)	1147(5)	2831.2(13)	29.1(6)
O(3)	2980.6(19)	8205(5)	4584.8(14)	25.3(6)
O(4)	-18.6(17)	4398(5)	6560.6(13)	27.2(5)
N(1)	2887.9(19)	4081(5)	4213.8(15)	18.7(6)
C(1)	3050(2)	3272(7)	2916.1(18)	19.3(7)
C(2)	3528(2)	4527(6)	3679.6(16)	17.8(6)
C(3)	4612(2)	3465(7)	3995.2(18)	22.9(7)
C(4)	5319(2)	4211(7)	3500.9(18)	19.8(7)
C(5)	5894(2)	6409(8)	3657.2(16)	21.8(7)
C(6)	6544(2)	7151(7)	3203.7(17)	24.0(7)
C(7)	6599(2)	5676(7)	2583.0(17)	22.1(7)
C(8)	6039(2)	3467(7)	2407.8(19)	24.8(7)
C(9)	5398(2)	2759(7)	2868.7(19)	22.7(7)
C(10)	2652(2)	6041(6)	4630.9(16)	15.9(7)
C(11)	1973(2)	5462(6)	5152.6(16)	16.1(6)
C(12)	1351(2)	3312(6)	5091.4(17)	16.6(6)
C(13)	708(2)	3017(6)	5578.8(18)	19.7(7)
C(14)	674(2)	4830(6)	6131.6(17)	18.4(7)

C(15)	1302(2)	6960(7)	6214.3(16)	18.2(6)
C(16)	1943(2)	7246(6)	5715.3(17)	17.3(7)
**Atom**	***x***	***y***	***z***	**U(eq)**
C(17)	-131(3)	6349(9)	7094(2)	37.3(9)
C(20)	2694(2)	3904(6)	1559.2(17)	18.9(7)
C(21)	3163(2)	5540(6)	1036.4(17)	19.5(6)
C(22)	3865(3)	7401(7)	1306(2)	33.0(9)
C(23)	4275(3)	8845(8)	808(2)	38.5(10)
C(24)	3985(3)	8410(8)	23(2)	32.3(9)
C(25)	3285(2)	6534(10)	-250.5(17)	33.7(8)
C(26)	2875(3)	5098(8)	244.6(19)	29.6(8)
C(31)	1533(2)	3995(6)	1358.5(17)	18.3(6)
C(32)	1031(2)	5980(6)	1627.7(18)	24.2(7)
C(33)	-30(3)	6059(7)	1428.2(19)	27.8(8)
C(34)	-579(3)	4202(8)	961.1(19)	28.5(8)
C(35)	-82(2)	2250(7)	691.2(18)	26.9(8)
C(36)	975(2)	2136(7)	897.6(16)	23.2(7)

Table S6 Anisotropic Displacement Parameters ($Å^2 \times 10^3$) for **4i**. The Anisotropic displacement factor exponent takes the form: $-2\pi^2[h^2a^{*2}U_{11}+2hka^*b^*U_{12}+\ldots]$.

**Atom**	**U_{11}**	**U_{22}**	**U_{33}**	**U_{23}**	**U_{13}**	**U_{12}**
Br(1)	36.84(19)	58.0(3)	35.28(19)	4.1(2)	21.60(14)	-6.6(2)
O(1)	25.3(11)	19.7(12)	12.1(10)	2.2(9)	2.4(8)	-0.6(10)
O(2)	44.7(14)	21.8(15)	20.0(11)	0.7(10)	6.5(10)	-8.1(12)
O(3)	34.3(14)	18.3(13)	27.9(13)	-1.5(10)	16.7(11)	-6.4(11)
O(4)	29.1(13)	29.4(13)	29.4(13)	-1.2(11)	19.1(10)	-1.1(11)
N(1)	22.5(13)	17.9(14)	18.7(13)	0.3(11)	10.7(11)	-1.9(12)
C(1)	16.9(15)	24.9(19)	17.3(16)	1.7(14)	6.7(12)	1.9(14)
C(2)	18.6(15)	20.1(17)	16.2(15)	0.3(13)	7.2(12)	-1.8(13)
C(3)	20.3(15)	30.3(19)	18.0(16)	4.1(14)	4.6(12)	0.6(14)
C(4)	13.8(14)	24.0(17)	20.2(16)	2.6(14)	1.3(11)	3.6(13)
C(5)	19.4(13)	27.2(18)	17.8(13)	-3.6(16)	2.8(11)	3.6(16)
C(6)	19.7(14)	24(2)	26.5(16)	-1.8(15)	1.9(12)	-2.1(14)
C(7)	16.6(14)	32.1(19)	18.8(15)	6.6(14)	6.4(12)	3.0(13)
C(8)	23.4(16)	27.8(19)	23.7(17)	-7.2(15)	6.9(13)	1.0(15)
C(9)	19.7(15)	20.4(17)	27.8(17)	-4.2(14)	5.3(13)	-3.3(14)
C(10)	16.2(14)	17.1(19)	13.8(14)	0.7(12)	2.6(11)	0.1(12)
C(11)	15.9(14)	16.8(15)	14.9(14)	2.8(12)	2.4(11)	3.4(13)
C(12)	19.2(14)	14.7(16)	15.9(14)	-4.3(12)	4.2(11)	0.0(13)
C(13)	19.1(14)	16.4(16)	23.2(16)	0.1(14)	4.2(12)	-3.6(13)

C(14)	17.4(15)	22.6(18)	17.3(15)	4.1(13)	8.7(12)	3.5(13)
C(15)	21.4(13)	17.0(17)	16.6(13)	-2.6(14)	5.4(10)	3.4(14)
**Atom**	**U_{11}**	**U_{22}**	**U_{33}**	**U_{23}**	**U_{13}**	**U_{12}**
C(16)	18.1(13)	12.8(17)	21.0(14)	-0.4(12)	4.4(11)	0.2(12)
C(17)	43.7(19)	44(2)	33.5(18)	-2(2)	27.8(15)	7(2)
C(20)	23.2(16)	19.0(16)	13.0(14)	-2.1(13)	1.4(12)	0.4(13)
C(21)	18.9(15)	22.3(16)	18.9(15)	0.5(13)	7.5(12)	4.9(14)
C(22)	41(2)	37(2)	21.9(17)	-6.3(15)	8.9(15)	-12.9(17)
C(23)	48(2)	38(2)	32(2)	-2.7(18)	13.4(17)	-16(2)
C(24)	37(2)	34(2)	31.4(19)	6.2(17)	18.5(16)	-1.7(17)
C(25)	34.1(17)	51(2)	17.4(14)	-2(2)	8.2(12)	-4(2)
C(26)	31.3(18)	37(2)	20.5(17)	-3.2(16)	6.2(14)	-8.6(17)
C(31)	21.4(15)	18.6(16)	13.6(15)	5.2(12)	2.1(12)	-1.0(13)
C(32)	26.6(15)	21(2)	26.1(16)	-1.8(13)	7.8(13)	-1.0(13)
C(33)	28.2(16)	26(2)	32.8(18)	-0.9(15)	13.8(14)	1.1(15)
C(34)	22.2(16)	37(2)	26.9(18)	8.5(16)	6.4(14)	-1.4(16)
C(35)	28.4(16)	30(2)	21.2(15)	-0.6(15)	3.3(12)	-10.1(15)
C(36)	28.7(15)	23(2)	18.6(14)	-0.3(14)	6.3(12)	-2.0(14)

Table S7 Bond Lengths for **4i**.

Atom	Atom	Length/Å	Atom	Atom	Length/Å
Br(1)	C(7)	1.899(3)	C(11)	C(16)	1.387(4)
O(1)	C(1)	1.349(4)	C(12)	C(13)	1.383(4)
O(1)	C(20)	1.453(3)	C(13)	C(14)	1.385(4)
O(2)	C(1)	1.202(4)	C(14)	C(15)	1.393(5)
O(3)	C(10)	1.233(4)	C(15)	C(16)	1.397(4)
O(4)	C(14)	1.368(3)	C(20)	C(21)	1.520(4)
O(4)	C(17)	1.437(5)	C(20)	C(31)	1.525(4)
N(1)	C(2)	1.458(4)	C(21)	C(22)	1.369(5)
N(1)	C(10)	1.358(4)	C(21)	C(26)	1.398(4)
C(1)	C(2)	1.517(4)	C(22)	C(23)	1.388(5)
C(2)	C(3)	1.543(4)	C(23)	C(24)	1.385(5)
C(3)	C(4)	1.506(4)	C(24)	C(25)	1.374(6)
C(4)	C(5)	1.385(5)	C(25)	C(26)	1.382(5)
C(4)	C(9)	1.393(5)	C(31)	C(32)	1.395(4)
C(5)	C(6)	1.390(4)	C(31)	C(36)	1.382(5)
C(6)	C(7)	1.375(5)	C(32)	C(33)	1.392(5)
C(7)	C(8)	1.382(5)	C(33)	C(34)	1.382(5)
C(8)	C(9)	1.386(5)	C(34)	C(35)	1.379(5)
C(10)	C(11)	1.492(4)	C(35)	C(36)	1.388(5)

C(11)	C(12)	1.399(4)

Table S8 Bond Angles for **4i**.

**Atom**	**Atom**	**Atom**	**Angle/°**	**Atom**	**Atom**	**Atom**	**Angle/°**
C(1)	O(1)	C(20)	116.8(3)	C(13)	C(12)	C(11)	120.0(3)
C(14)	O(4)	C(17)	116.9(3)	C(12)	C(13)	C(14)	120.7(3)
C(10)	N(1)	C(2)	119.8(3)	O(4)	C(14)	C(13)	115.7(3)
O(1)	C(1)	C(2)	109.9(3)	O(4)	C(14)	C(15)	123.9(3)
O(2)	C(1)	O(1)	124.3(3)	C(13)	C(14)	C(15)	120.4(3)
O(2)	C(1)	C(2)	125.8(3)	C(14)	C(15)	C(16)	118.4(3)
N(1)	C(2)	C(1)	109.4(2)	C(11)	C(16)	C(15)	121.7(3)
N(1)	C(2)	C(3)	111.3(2)	O(1)	C(20)	C(21)	106.4(2)
C(1)	C(2)	C(3)	109.4(2)	O(1)	C(20)	C(31)	109.5(2)
C(4)	C(3)	C(2)	111.8(3)	C(21)	C(20)	C(31)	113.3(3)
C(5)	C(4)	C(3)	120.3(3)	C(22)	C(21)	C(20)	123.0(3)
C(5)	C(4)	C(9)	118.3(3)	C(22)	C(21)	C(26)	118.5(3)
C(9)	C(4)	C(3)	121.4(3)	C(26)	C(21)	C(20)	118.5(3)
C(4)	C(5)	C(6)	121.3(3)	C(21)	C(22)	C(23)	121.0(3)
C(7)	C(6)	C(5)	118.8(3)	C(24)	C(23)	C(22)	120.4(4)
C(6)	C(7)	Br(1)	118.9(3)	C(25)	C(24)	C(23)	118.9(3)
C(6)	C(7)	C(8)	121.7(3)	C(24)	C(25)	C(26)	120.9(3)
C(8)	C(7)	Br(1)	119.4(2)	C(25)	C(26)	C(21)	120.4(3)
C(7)	C(8)	C(9)	118.6(3)	C(32)	C(31)	C(20)	120.1(3)
C(8)	C(9)	C(4)	121.4(3)	C(36)	C(31)	C(20)	120.1(3)
O(3)	C(10)	N(1)	121.9(3)	C(36)	C(31)	C(32)	119.8(3)
O(3)	C(10)	C(11)	121.2(3)	C(33)	C(32)	C(31)	119.4(3)
N(1)	C(10)	C(11)	116.9(3)	C(34)	C(33)	C(32)	120.3(3)
C(12)	C(11)	C(10)	124.5(3)	C(35)	C(34)	C(33)	120.3(3)
C(16)	C(11)	C(10)	116.7(3)	C(34)	C(35)	C(36)	119.8(3)
C(16)	C(11)	C(12)	118.8(3)	C(31)	C(36)	C(35)	120.4(3)

Table S9 Torsion Angles for **4i**.

**A**	**B**	**C**	**D**	**Angle/°**	**A**	**B**	**C**	**D**	**Angle/°**
Br(1)	C(7)	C(8)	C(9)	178.3(3)	C(10)	C(11)	C(12)	C(13)	-176.5(3)
O(1)	C(1)	C(2)	N(1)	135.9(3)	C(10)	C(11)	C(16)	C(15)	177.2(3)
O(1)	C(1)	C(2)	C(3)	-102.0(3)	C(11)	C(12)	C(13)	C(14)	-0.2(5)
O(1)	C(20)	C(21)	C(22)	3.8(4)	C(12)	C(11)	C(16)	C(15)	-0.6(4)
O(1)	C(20)	C(21)	C(26)	-177.2(3)	C(12)	C(13)	C(14)	O(4)	177.4(3)
O(1)	C(20)	C(31)	C(32)	35.7(4)	C(12)	C(13)	C(14)	C(15)	-1.2(5)

O(1)	C(20)	C(31)	C(36)	-145.4(3)	C(13)	C(14)	C(15)	C(16)	1.6(4)
O(2)	C(1)	C(2)	N(1)	-46.7(4)	C(14)	C(15)	C(16)	C(11)	-0.7(4)
O(2)	C(1)	C(2)	C(3)	75.5(4)	C(16)	C(11)	C(12)	C(13)	1.1(4)
**A**	**B**	**C**	**D**	**Angle/°**	**A**	**B**	**C**	**D**	**Angle/°**
O(3)	C(10)	C(11)	C(12)	161.6(3)	C(17)	O(4)	C(14)	C(13)	-174.9(3)
O(3)	C(10)	C(11)	C(16)	-16.1(4)	C(17)	O(4)	C(14)	C(15)	3.6(4)
O(4)	C(14)	C(15)	C(16)	-176.9(3)	C(20)	O(1)	C(1)	O(2)	0.3(4)
N(1)	C(2)	C(3)	C(4)	-172.5(3)	C(20)	O(1)	C(1)	C(2)	177.7(2)
N(1)	C(10)	C(11)	C(12)	-18.5(4)	C(20)	C(21)	C(22)	C(23)	179.8(4)
N(1)	C(10)	C(11)	C(16)	163.9(3)	C(20)	C(21)	C(26)	C(25)	-179.7(3)
C(1)	O(1)	C(20)	C(21)	-159.5(3)	C(20)	C(31)	C(32)	C(33)	178.9(3)
C(1)	O(1)	C(20)	C(31)	77.7(3)	C(20)	C(31)	C(36)	C(35)	-177.9(3)
C(1)	C(2)	C(3)	C(4)	66.5(4)	C(21)	C(20)	C(31)	C(32)	-82.8(3)
C(2)	N(1)	C(10)	O(3)	-1.5(5)	C(21)	C(20)	C(31)	C(36)	96.0(3)
C(2)	N(1)	C(10)	C(11)	178.6(3)	C(21)	C(22)	C(23)	C(24)	-0.6(6)
C(2)	C(3)	C(4)	C(5)	91.5(4)	C(22)	C(21)	C(26)	C(25)	-0.7(5)
C(2)	C(3)	C(4)	C(9)	-87.1(4)	C(22)	C(23)	C(24)	C(25)	0.1(6)
C(3)	C(4)	C(5)	C(6)	-179.3(3)	C(23)	C(24)	C(25)	C(26)	0.0(6)
C(3)	C(4)	C(9)	C(8)	179.1(3)	C(24)	C(25)	C(26)	C(21)	0.2(6)
C(4)	C(5)	C(6)	C(7)	0.9(5)	C(26)	C(21)	C(22)	C(23)	0.8(5)
C(5)	C(4)	C(9)	C(8)	0.5(5)	C(31)	C(20)	C(21)	C(22)	124.1(3)
C(5)	C(6)	C(7)	Br(1)	-178.4(2)	C(31)	C(20)	C(21)	C(26)	-56.9(4)
C(5)	C(6)	C(7)	C(8)	-1.0(5)	C(31)	C(32)	C(33)	C(34)	-0.7(5)
C(6)	C(7)	C(8)	C(9)	0.8(5)	C(32)	C(31)	C(36)	C(35)	1.0(5)
C(7)	C(8)	C(9)	C(4)	-0.6(5)	C(32)	C(33)	C(34)	C(35)	0.2(5)
Br(1)	C(7)	C(8)	C(9)	178.3(3)	C(10)	C(11)	C(12)	C(13)	-176.5(3)
O(1)	C(1)	C(2)	N(1)	135.9(3)	C(10)	C(11)	C(16)	C(15)	177.2(3)
O(1)	C(1)	C(2)	C(3)	-102.0(3)	C(11)	C(12)	C(13)	C(14)	-0.2(5)
O(1)	C(20)	C(21)	C(22)	3.8(4)	C(12)	C(11)	C(16)	C(15)	-0.6(4)
C(9)	C(4)	C(5)	C(6)	-0.7(5)	C(33)	C(34)	C(35)	C(36)	0.8(5)
C(10)	N(1)	C(2)	C(1)	-133.0(3)	C(34)	C(35)	C(36)	C(31)	-1.4(5)
C(10)	N(1)	C(2)	C(3)	106.0(3)	C(36)	C(31)	C(32)	C(33)	0.1(5)

Table S10 Hydrogen Atom Coordinates (Å×10^4) and Isotropic Displacement Parameters (Å^2×10^3) for **4i**.

**Atom**	***x***	***y***	***z***	**U(eq)**
H(1)	2655.29	2547.14	4265.64	22
H(2)	3574.29	6397.07	3597.22	21
H(3A)	4886.99	4108.85	4523.39	27
H(3B)	4579.62	1589.82	4020.86	27

H(5)	5844.12	7431.09	4082.96	26
H(6)	6941.9	8650.19	3320.55	29
H(8)	6091.37	2455.24	1980.7	30
H(9)	5004.41	1253.4	2751.12	27
**Atom**	***x***	***y***	***z***	**U(eq)**
H(12)	1370.41	2054.78	4715.36	20
H(13)	285.83	1556.42	5533.96	24
H(15)	1295.12	8187.81	6600.77	22
H(16)	2368.79	8700.56	5762.6	21
H(17A)	-317.69	7950.04	6816.75	56
H(17B)	513.49	6566.03	7479.76	56
H(17C)	-667.23	5862.63	7348.59	56
H(20)	2921	2105.84	1532.56	23
H(22)	4075.2	7708.46	1843.56	40
H(23)	4756.55	10138.3	1005.31	46
H(24)	4264.73	9393.02	-319.49	39
H(25)	3081.4	6219.52	-787.51	40
H(26)	2393.72	3803.62	46.02	36
H(32)	1408.89	7266.36	1944.33	29
H(33)	-376.83	7395.23	1613.92	33
H(34)	-1302.75	4269.45	825.41	34
H(35)	-461.3	987.31	364.92	32
H(36)	1316.78	770.26	720.71	28

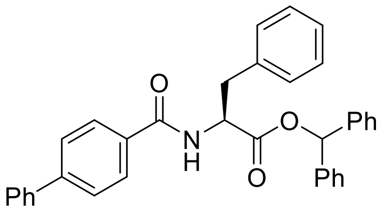

(**4al**)

The absolute configuration of **4al** was determined to be the (*S*)-configuration.

X-ray Crystal Structure for 4al (CCDC 2469924)

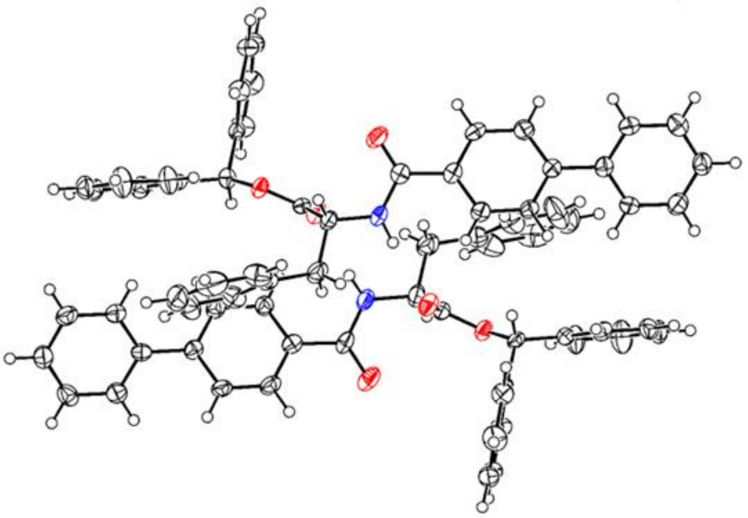

Crystal data and structure refinement for 4al.

Identification code	**4al** (CCDC 2469924)
Empirical formula	$C_{35}H_{29}NO_3$
Formula weight	511.59
Temperature/K	173.15
Crystal system	orthorhombic
Space group	$Pca2_1$
a/Å	18.2598(9)
b/Å	15.3959(9)
c/Å	18.9373(12)
α/°	90
β/°	90
γ/°	90
Volume/Å^3	5323.8(5)
Z	8
ρ_{calc} g/cm^3	1.277
μ/mm^{-1}	0.052
F (000)	2160.0
Crystal size/mm^3	0.146 × 0.134 × 0.134
Radiation	Ag Kα (λ = 0.56086)
2Θ range for data collection/°	3.908 to 40.998
Index ranges	$-22 \leq h \leq 22$, $-19 \leq k \leq 16$, $-22 \leq l \leq 23$
Reflections collected	30376
Independent reflections	10216 [R_{int} = 0.0557, R_{sigma} = 0.0654]
Data/restraints/parameters	10216/2/712
Goodness-of-fit on F^2	1.040
Final R indexes [I>=2σ (I)]	R_1 = 0.0498, wR_2 = 0.1119

Final R indexes [all data]	R_1 = 0.0785, wR_2 = 0.1274
Largest diff. peak/hole / e Å^{-3}	0.33/-0.23

Table S11 Fractional Atomic Coordinates (×10^4) and Equivalent Isotropic Displacement Parameters (Å^2×10^3) for **4al**. Ueq is defined as 1/3 of the trace of the orthogonalised U_{IJ} tensor.

**Atom**	***x***	***y***	***z***	**U(eq)**
O(1)	5373.3(16)	8024(3)	3079(2)	44.6(10)
O(2)	4474.2(15)	8699(2)	4664(2)	37.3(9)
O(3)	3631.3(14)	8769(2)	3803.5(19)	29.5(8)
N(1)	5025.4(18)	7176(3)	3984(3)	34.6(10)
C(1)	4161(2)	8369(3)	4173(3)	29.5(12)
C(2)	4265(2)	7450(3)	3913(3)	36.6(13)
**Atom**	***x***	***y***	***z***	**U(eq)**
C(3)	3791(2)	6821(4)	4324(3)	46.7(15)
C(4)	2982(2)	6871(4)	4163(3)	36.5(13)
C(5)	2527(3)	7331(4)	4588(4)	47.1(15)
C(6)	1791(3)	7384(5)	4475(5)	73(2)
C(7)	1491(3)	6971(5)	3915(5)	68(2)
C(8)	1921(3)	6500(5)	3463(3)	66(2)
C(9)	2675(3)	6441(4)	3591(4)	59.0(19)
C(10)	5532(2)	7481(3)	3534(3)	30.7(12)
C(11)	6290(2)	7113(3)	3597(3)	29.2(11)
C(12)	6869(2)	7589(4)	3323(3)	33.7(12)
C(13)	7580(2)	7281(3)	3368(3)	31.5(11)
C(14)	7727(2)	6483(3)	3694(3)	27.7(11)
C(15)	7139(2)	5999(3)	3954(3)	31.1(12)
C(16)	6432(2)	6313(3)	3907(3)	30.9(12)
C(17)	8488(2)	6155(3)	3760(3)	27.9(11)
C(18)	9002(2)	6272(3)	3228(3)	34.4(12)
C(19)	9714(2)	5965(4)	3294(3)	38.7(14)
C(20)	9925(2)	5558(3)	3902(3)	40.3(13)
C(21)	9429(2)	5444(4)	4453(3)	38.9(14)
C(22)	8712(2)	5738(3)	4378(3)	35.0(12)
C(30)	3376.8(19)	9612(3)	4057(3)	25.5(11)
C(31)	2583(2)	9697(3)	3837(3)	26.9(11)
C(32)	2271(2)	9206(4)	3300(3)	41.6(15)
C(33)	1544(3)	9328(5)	3116(4)	55.1(19)
C(34)	1125(2)	9955(4)	3454(3)	39.1(14)
C(35)	1432(2)	10447(3)	3982(3)	36.7(14)
C(36)	2159(2)	10320(3)	4175(3)	29.8(12)

C(41)	3854.6(19)	10330(3)	3770(3)	25.4(11)
C(42)	4015(2)	10367(3)	3053(3)	34.0(13)
C(43)	4449(2)	11040(4)	2788(3)	39.4(14)
C(44)	4708(2)	11672(4)	3225(4)	39.7(14)
C(45)	4536(2)	11658(4)	3930(4)	45.1(15)
C(46)	4116(2)	10977(4)	4202(3)	36.4(12)
O(1A)	4483.7(15)	6975(3)	6959(2)	40.6(10)
O(2A)	5405.3(15)	6287(2)	5361(2)	36.8(9)
O(3A)	6267.2(13)	6231(2)	6214.8(19)	27.8(8)
N(1A)	4847.0(19)	7791(3)	6051(2)	34.0(10)
C(1A)	5730(2)	6610(3)	5854(3)	27.1(11)
C(2A)	5603(2)	7534(3)	6121(3)	29.4(11)
C(3A)	6080(2)	8170(4)	5692(3)	42.1(14)
**Atom**	***x***	***y***	***z***	**U(eq)**
C(4A)	6893(2)	8129(3)	5844(3)	32.2(12)
C(5A)	7191(3)	8573(4)	6402(4)	49.2(16)
C(6A)	7944(3)	8530(5)	6533(4)	68(2)
C(7A)	8378(3)	8030(5)	6097(5)	68(2)
C(8A)	8087(3)	7616(4)	5535(4)	61(2)
C(9A)	7347(3)	7655(4)	5403(3)	44.5(14)
C(10A)	4333(2)	7510(3)	6501(3)	28.2(11)
C(11A)	3582(2)	7874(3)	6423(3)	27.1(11)
C(12A)	2991(2)	7408(4)	6700(3)	31.2(11)
C(13A)	2284(2)	7719(3)	6632(3)	33.7(12)
C(14A)	2139(2)	8517(3)	6311(3)	27.7(11)
C(15A)	2736(2)	8995(3)	6056(3)	30.8(12)
C(16A)	3442(2)	8676(3)	6104(3)	29.7(11)
C(17A)	1383(2)	8843(3)	6225(3)	28.3(11)
C(18A)	1164(2)	9259(3)	5609(3)	31.8(12)
C(19A)	457(2)	9553(4)	5521(3)	41.4(14)
C(20A)	-46(2)	9452(3)	6056(3)	37.2(12)
C(21A)	162(2)	9054(3)	6683(3)	35.8(13)
C(22A)	864(2)	8744(3)	6769(3)	30.0(11)
C(30A)	6531(2)	5390(3)	5957(3)	27.4(11)
C(31A)	7322(2)	5307(3)	6176(3)	25.5(11)
C(32A)	7750(2)	4689(4)	5844(3)	35.5(13)
C(33A)	8478(2)	4577(3)	6037(3)	35.7(13)
C(34A)	8784(2)	5070(4)	6559(3)	40.3(15)
C(35A)	8362(2)	5678(4)	6887(3)	48.4(17)
C(36A)	7632(2)	5813(4)	6695(3)	43.4(15)
C(41A)	6058.7(19)	4662(3)	6233(3)	26.0(11)

C(42A)	5771(2)	4048(4)	5780(3)	38.8(13)
C(43A)	5350(3)	3373(4)	6042(4)	47.0(15)
C(44A)	5206(2)	3312(4)	6749(4)	41.7(15)
C(45A)	5497(2)	3921(4)	7205(3)	38.5(14)
C(46A)	5925(2)	4584(3)	6950(3)	33.7(13)

Table S12 Anisotropic Displacement Parameters (Å^2×10^3) for **4al**. The Anisotropic displacement factor exponent takes the form: $-2\pi^2[h^2a^{*2}U_{11}+2hka^*b^*U_{12}+\ldots]$.

**Atom**	**U_{11}**	**U_{22}**	**U_{33}**	**U_{23}**	**U_{13}**	**U_{12}**
O(1)	47.0(18)	40(3)	46(2)	9(2)	-20.2(16)	1.9(16)
O(2)	38.1(16)	40(2)	34(2)	3.5(19)	-8.6(14)	7.0(14)
O(3)	29.1(14)	22(2)	38(2)	1.9(17)	-5.5(13)	2.7(12)
**Atom**	**U_{11}**	**U_{22}**	**U_{33}**	**U_{23}**	**U_{13}**	**U_{12}**
N(1)	29.8(19)	30(3)	44(3)	8(2)	-6.0(19)	5.6(16)
C(1)	23.7(19)	28(3)	37(3)	8(2)	-1.8(19)	0.6(17)
C(2)	27(2)	29(3)	53(3)	6(3)	-7(2)	4.6(17)
C(3)	43(3)	35(4)	62(4)	13(3)	-6(3)	-2(2)
C(4)	36(2)	24(3)	50(4)	1(3)	1(2)	-7.2(19)
C(5)	43(3)	40(4)	58(4)	-8(3)	0(3)	-14(2)
C(6)	40(3)	55(5)	123(7)	-14(5)	20(3)	-8(3)
C(7)	40(3)	59(5)	106(7)	26(5)	-13(3)	-10(3)
C(8)	83(4)	77(6)	38(4)	10(4)	-22(3)	-43(4)
C(9)	65(4)	61(5)	51(4)	-16(4)	15(3)	-26(3)
C(10)	31(2)	22(3)	38(3)	0(2)	-14(2)	1.9(18)
C(11)	36(2)	23(3)	28(3)	-2(2)	-1.9(19)	0.1(18)
C(12)	43(2)	23(3)	35(3)	5(2)	1(2)	5.0(19)
C(13)	38(2)	24(3)	32(3)	6(2)	4(2)	0.6(19)
C(14)	33(2)	21(3)	29(3)	-3(2)	0.4(18)	2.7(16)
C(15)	33(2)	22(3)	39(3)	1(2)	-2(2)	1.2(17)
C(16)	28(2)	27(3)	38(3)	2(2)	0(2)	0.7(17)
C(17)	33(2)	23(3)	28(3)	0(2)	1.0(18)	-4.7(17)
C(18)	36(2)	28(3)	39(3)	0(2)	1(2)	-1.9(19)
C(19)	34(2)	29(3)	54(4)	-2(3)	11(2)	-2.0(19)
C(20)	30(2)	27(3)	64(4)	3(3)	0(2)	-1.0(18)
C(21)	39(2)	32(3)	46(4)	11(3)	-5(2)	-4(2)
C(22)	34(2)	31(3)	39(3)	8(3)	1(2)	-1.6(19)
C(30)	31(2)	19(3)	26(3)	4(2)	-0.1(18)	4.5(16)
C(31)	26.5(19)	24(3)	31(3)	3(2)	2.5(18)	1.9(17)
C(32)	33(2)	45(4)	47(4)	-18(3)	-6(2)	10(2)
C(33)	39(3)	62(5)	65(5)	-24(4)	-17(3)	4(2)

C(34)	25(2)	37(3)	55(4)	3(3)	-3(2)	1.8(19)
C(35)	33(2)	31(3)	47(4)	2(3)	10(2)	4.6(19)
C(36)	37(2)	20(3)	32(3)	-1(2)	0(2)	2.6(18)
C(41)	22.6(18)	23(3)	31(3)	3(2)	-5.1(18)	4.9(16)
C(42)	42(2)	28(3)	32(3)	2(2)	1(2)	-1(2)
C(43)	39(2)	36(4)	43(4)	10(3)	5(2)	2(2)
C(44)	33(2)	27(3)	59(4)	7(3)	4(2)	-2.3(19)
C(45)	43(2)	30(3)	62(4)	-10(3)	-2(3)	-10(2)
C(46)	37(2)	33(3)	39(3)	-2(3)	-4(2)	-5(2)
O(1A)	39.4(17)	36(2)	46(2)	14(2)	-12.7(15)	-0.1(14)
O(2A)	35.2(16)	32(2)	43(2)	0.4(19)	-11.5(15)	3.6(13)
O(3A)	28.2(13)	20.3(19)	35(2)	-0.2(16)	-5.3(13)	4.5(12)
N(1A)	30.0(18)	37(3)	35(3)	10(2)	-5.6(17)	9.0(16)
**Atom**	**U_{11}**	**U_{22}**	**U_{33}**	**U_{23}**	**U_{13}**	**U_{12}**
C(1A)	25.6(19)	25(3)	31(3)	9(2)	0.9(18)	-0.7(17)
C(2A)	28.4(19)	26(3)	34(3)	4(2)	-4.9(18)	3.8(16)
C(3A)	39(2)	28(3)	58(4)	11(3)	-7(2)	-1(2)
C(4A)	35(2)	22(3)	40(3)	8(2)	-2(2)	-3.3(18)
C(5A)	56(3)	50(4)	42(4)	-2(3)	0(3)	-16(3)
C(6A)	67(4)	69(6)	66(5)	18(4)	-26(4)	-34(4)
C(7A)	34(3)	55(5)	116(7)	33(5)	-8(3)	-8(3)
C(8A)	42(3)	43(4)	98(6)	-7(4)	17(3)	-8(2)
C(9A)	48(3)	36(3)	49(4)	-4(3)	8(2)	-9(2)
C(10A)	30(2)	24(3)	30(3)	-5(2)	-5.4(18)	-2.7(18)
C(11A)	32(2)	22(3)	28(3)	-5(2)	-5.9(18)	1.6(17)
C(12A)	41(2)	25(3)	28(3)	6(2)	-2(2)	3.3(19)
C(13A)	34(2)	29(3)	38(3)	0(2)	2(2)	-0.9(18)
C(14A)	30(2)	27(3)	26(3)	-1(2)	-0.7(18)	0.8(17)
C(15A)	32(2)	21(3)	39(3)	4(2)	0(2)	0.6(17)
C(16A)	30.1(19)	23(3)	36(3)	-1(2)	-4.3(19)	-3.3(17)
C(17A)	30(2)	18(3)	37(3)	-5(2)	-1.1(19)	0.8(17)
C(18A)	30(2)	32(3)	33(3)	5(2)	2.9(19)	-3.3(19)
C(19A)	36(2)	36(4)	52(4)	9(3)	-6(2)	0(2)
C(20A)	31(2)	27(3)	54(3)	0(3)	-1(2)	0.6(18)
C(21A)	36(2)	32(3)	40(3)	-9(3)	5(2)	-1.5(19)
C(22A)	39(2)	25(3)	26(3)	-1(2)	1.0(19)	-0.1(19)
C(30A)	27.0(19)	28(3)	27(3)	-6(2)	-1.5(18)	3.0(17)
C(31A)	27.1(19)	23(3)	27(3)	5(2)	0.1(17)	-1.8(16)
C(32A)	35(2)	29(3)	43(4)	0(3)	-3(2)	2.5(19)
C(33A)	33(2)	27(3)	47(4)	4(3)	0(2)	8.0(19)
C(34A)	26(2)	40(4)	54(4)	3(3)	-3(2)	1(2)

C(35A)	36(2)	54(5)	55(4)	-22(3)	-10(2)	4(2)
C(36A)	32(2)	48(4)	50(4)	-16(3)	-3(2)	7(2)
C(41A)	24.3(18)	22(3)	32(3)	1(2)	-1.1(18)	3.0(16)
C(42A)	45(2)	38(3)	34(3)	-4(3)	-1(2)	-7(2)
C(43A)	50(3)	35(4)	56(4)	-5(3)	-6(3)	-12(2)
C(44A)	35(2)	30(3)	60(4)	9(3)	6(2)	-2(2)
C(45A)	40(3)	39(4)	36(3)	10(3)	7(2)	2(2)
C(46A)	36(2)	30(3)	35(3)	-4(3)	-1(2)	-3.8(19)

Table S13 Bond Lengths for **4al**.

**Atom**	**Atom**	**Length/Å**	**Atom**	**Atom**	**Length/Å**
O(1)	C(10)	1.234(6)	O(1A)	C(10A)	1.227(6)
**Atom**	**Atom**	**Length/Å**	**Atom**	**Atom**	**Length/Å**
O(2)	C(1)	1.204(6)	O(2A)	C(1A)	1.213(6)
O(3)	C(1)	1.343(5)	O(3A)	C(1A)	1.330(5)
O(3)	C(30)	1.459(6)	O(3A)	C(30A)	1.465(6)
N(1)	C(2)	1.458(5)	N(1A)	C(2A)	1.442(5)
N(1)	C(10)	1.343(7)	N(1A)	C(10A)	1.340(6)
C(1)	C(2)	1.511(7)	C(1A)	C(2A)	1.529(7)
C(2)	C(3)	1.513(7)	C(2A)	C(3A)	1.542(7)
C(3)	C(4)	1.511(6)	C(3A)	C(4A)	1.515(6)
C(4)	C(5)	1.356(8)	C(4A)	C(5A)	1.371(8)
C(4)	C(9)	1.387(9)	C(4A)	C(9A)	1.384(8)
C(5)	C(6)	1.363(7)	C(5A)	C(6A)	1.398(7)
C(6)	C(7)	1.353(11)	C(6A)	C(7A)	1.379(11)
C(7)	C(8)	1.369(11)	C(7A)	C(8A)	1.350(11)
C(8)	C(9)	1.403(8)	C(8A)	C(9A)	1.377(7)
C(10)	C(11)	1.502(6)	C(10A)	C(11A)	1.489(6)
C(11)	C(12)	1.386(7)	C(11A)	C(12A)	1.399(6)
C(11)	C(16)	1.389(7)	C(11A)	C(16A)	1.398(7)
C(12)	C(13)	1.384(6)	C(12A)	C(13A)	1.383(6)
C(13)	C(14)	1.400(7)	C(13A)	C(14A)	1.396(7)
C(14)	C(15)	1.397(6)	C(14A)	C(15A)	1.402(6)
C(14)	C(17)	1.484(5)	C(14A)	C(17A)	1.477(5)
C(15)	C(16)	1.381(6)	C(15A)	C(16A)	1.382(6)
C(17)	C(18)	1.387(7)	C(17A)	C(18A)	1.390(7)
C(17)	C(22)	1.397(7)	C(17A)	C(22A)	1.408(7)
C(18)	C(19)	1.389(6)	C(18A)	C(19A)	1.380(6)
C(19)	C(20)	1.366(8)	C(19A)	C(20A)	1.377(8)
C(20)	C(21)	1.392(8)	C(20A)	C(21A)	1.388(8)

C(21)	C(22)	1.393(6)	C(21A)	C(22A)	1.377(6)
C(30)	C(31)	1.513(5)	C(30A)	C(31A)	1.508(5)
C(30)	C(41)	1.509(6)	C(30A)	C(41A)	1.508(6)
C(31)	C(32)	1.390(7)	C(31A)	C(32A)	1.384(7)
C(31)	C(36)	1.389(6)	C(31A)	C(36A)	1.376(7)
C(32)	C(33)	1.386(6)	C(32A)	C(33A)	1.389(6)
C(33)	C(34)	1.388(8)	C(33A)	C(34A)	1.365(8)
C(34)	C(35)	1.372(8)	C(34A)	C(35A)	1.362(8)
C(35)	C(36)	1.390(6)	C(35A)	C(36A)	1.398(6)
C(41)	C(42)	1.391(7)	C(41A)	C(42A)	1.380(7)
C(41)	C(46)	1.375(7)	C(41A)	C(46A)	1.384(7)
C(42)	C(43)	1.397(7)	C(42A)	C(43A)	1.384(8)
C(43)	C(44)	1.362(8)	C(43A)	C(44A)	1.368(9)
**Atom**	**Atom**	**Length/Å**	**Atom**	**Atom**	**Length/Å**
C(44)	C(45)	1.372(9)	C(44A)	C(45A)	1.380(8)
C(45)	C(46)	1.396(8)	C(45A)	C(46A)	1.374(7)

Table S14 Bond Angles for **4al**.

**Atom**	**Atom**	**Atom**	**Angle/˚**	**Atom**	**Atom**	**Atom**	**Angle/˚**
C(1)	O(3)	C(30)	117.8(4)	C(1A)	O(3A)	C(30A)	117.3(4)
C(10)	N(1)	C(2)	119.8(4)	C(10A)	N(1A)	C(2A)	121.5(4)
O(2)	C(1)	O(3)	123.4(5)	O(2A)	C(1A)	O(3A)	125.2(5)
O(2)	C(1)	C(2)	126.0(4)	O(2A)	C(1A)	C(2A)	124.3(4)
O(3)	C(1)	C(2)	110.5(4)	O(3A)	C(1A)	C(2A)	110.4(4)
N(1)	C(2)	C(1)	111.1(4)	N(1A)	C(2A)	C(1A)	111.7(4)
N(1)	C(2)	C(3)	108.2(4)	N(1A)	C(2A)	C(3A)	108.5(4)
C(1)	C(2)	C(3)	111.1(5)	C(1A)	C(2A)	C(3A)	109.3(4)
C(4)	C(3)	C(2)	115.0(4)	C(4A)	C(3A)	C(2A)	115.2(4)
C(5)	C(4)	C(3)	120.4(5)	C(5A)	C(4A)	C(3A)	121.0(5)
C(5)	C(4)	C(9)	117.8(5)	C(5A)	C(4A)	C(9A)	119.4(5)
C(9)	C(4)	C(3)	121.8(5)	C(9A)	C(4A)	C(3A)	119.6(5)
C(4)	C(5)	C(6)	122.9(6)	C(4A)	C(5A)	C(6A)	120.2(6)
C(7)	C(6)	C(5)	119.6(6)	C(7A)	C(6A)	C(5A)	119.0(7)
C(6)	C(7)	C(8)	120.6(5)	C(8A)	C(7A)	C(6A)	120.6(5)
C(7)	C(8)	C(9)	119.2(6)	C(7A)	C(8A)	C(9A)	120.6(6)
C(4)	C(9)	C(8)	120.1(6)	C(8A)	C(9A)	C(4A)	120.1(6)
O(1)	C(10)	N(1)	121.2(4)	O(1A)	C(10A)	N(1A)	120.6(4)
O(1)	C(10)	C(11)	121.8(4)	O(1A)	C(10A)	C(11A)	121.9(4)
N(1)	C(10)	C(11)	116.9(4)	N(1A)	C(10A)	C(11A)	117.4(4)
C(12)	C(11)	C(10)	118.3(5)	C(12A)	C(11A)	C(10A)	118.7(5)

C(12)	C(11)	C(16)	119.0(4)	C(16A)	C(11A)	C(10A)	123.0(4)
C(16)	C(11)	C(10)	122.7(4)	C(16A)	C(11A)	C(12A)	118.3(4)
C(13)	C(12)	C(11)	120.7(5)	C(13A)	C(12A)	C(11A)	120.6(5)
C(12)	C(13)	C(14)	120.5(4)	C(12A)	C(13A)	C(14A)	121.5(4)
C(13)	C(14)	C(17)	121.0(4)	C(13A)	C(14A)	C(15A)	117.7(4)
C(15)	C(14)	C(13)	118.4(4)	C(13A)	C(14A)	C(17A)	121.6(4)
C(15)	C(14)	C(17)	120.6(4)	C(15A)	C(14A)	C(17A)	120.7(5)
C(16)	C(15)	C(14)	120.6(5)	C(16A)	C(15A)	C(14A)	121.1(5)
C(15)	C(16)	C(11)	120.7(4)	C(15A)	C(16A)	C(11A)	120.9(4)
C(18)	C(17)	C(14)	121.9(5)	C(18A)	C(17A)	C(14A)	121.1(4)
C(18)	C(17)	C(22)	118.1(4)	C(18A)	C(17A)	C(22A)	118.1(4)
C(22)	C(17)	C(14)	120.0(4)	C(22A)	C(17A)	C(14A)	120.8(5)
C(17)	C(18)	C(19)	121.5(5)	C(19A)	C(18A)	C(17A)	121.5(4)
**Atom**	**Atom**	**Atom**	**Angle/°**	**Atom**	**Atom**	**Atom**	**Angle/°**
C(20)	C(19)	C(18)	119.7(5)	C(20A)	C(19A)	C(18A)	119.8(6)
C(19)	C(20)	C(21)	120.4(4)	C(19A)	C(20A)	C(21A)	119.8(4)
C(20)	C(21)	C(22)	119.7(5)	C(22A)	C(21A)	C(20A)	120.6(5)
C(21)	C(22)	C(17)	120.6(5)	C(21A)	C(22A)	C(17A)	120.2(5)
O(3)	C(30)	C(31)	107.0(3)	O(3A)	C(30A)	C(31A)	107.4(4)
O(3)	C(30)	C(41)	110.4(3)	O(3A)	C(30A)	C(41A)	110.8(3)
C(41)	C(30)	C(31)	113.0(4)	C(41A)	C(30A)	C(31A)	112.9(4)
C(32)	C(31)	C(30)	123.2(4)	C(32A)	C(31A)	C(30A)	118.4(4)
C(36)	C(31)	C(30)	117.9(4)	C(36A)	C(31A)	C(30A)	122.8(4)
C(36)	C(31)	C(32)	118.9(4)	C(36A)	C(31A)	C(32A)	118.8(4)
C(33)	C(32)	C(31)	120.2(5)	C(31A)	C(32A)	C(33A)	120.4(5)
C(32)	C(33)	C(34)	120.4(5)	C(34A)	C(33A)	C(32A)	120.9(5)
C(35)	C(34)	C(33)	119.6(4)	C(35A)	C(34A)	C(33A)	118.7(4)
C(34)	C(35)	C(36)	120.3(5)	C(34A)	C(35A)	C(36A)	121.5(5)
C(31)	C(36)	C(35)	120.6(5)	C(31A)	C(36A)	C(35A)	119.6(5)
C(42)	C(41)	C(30)	120.3(4)	C(42A)	C(41A)	C(30A)	120.8(5)
C(46)	C(41)	C(30)	121.2(5)	C(42A)	C(41A)	C(46A)	118.9(5)
C(46)	C(41)	C(42)	118.5(5)	C(46A)	C(41A)	C(30A)	120.3(4)
C(41)	C(42)	C(43)	120.0(5)	C(41A)	C(42A)	C(43A)	120.1(5)
C(44)	C(43)	C(42)	120.6(6)	C(44A)	C(43A)	C(42A)	120.6(6)
C(43)	C(44)	C(45)	119.9(5)	C(43A)	C(44A)	C(45A)	119.5(5)
C(44)	C(45)	C(46)	119.8(5)	C(46A)	C(45A)	C(44A)	120.2(5)
C(41)	C(46)	C(45)	121.0(5)	C(45A)	C(46A)	C(41A)	120.7(5)

Table S15 Torsion Angles for **4al**.

**A**	**B**	**C**	**D**	**Angle/°**	**A**	**B**	**C**	**D**	**Angle/°**

O(1)	C(10)	C(11)	C(12)	22.8(8)	O(1A)	C(10A)	C(11A)	C(12A)	-22.0(7)
O(1)	C(10)	C(11)	C(16)	-156.3(5)	O(1A)	C(10A)	C(11A)	C(16A)	156.2(5)
O(2)	C(1)	C(2)	N(1)	-35.6(7)	O(2A)	C(1A)	C(2A)	N(1A)	34.3(6)
O(2)	C(1)	C(2)	C(3)	84.9(5)	O(2A)	C(1A)	C(2A)	C(3A)	-85.8(5)
O(3)	C(1)	C(2)	N(1)	147.9(4)	O(3A)	C(1A)	C(2A)	N(1A)	-148.8(4)
O(3)	C(1)	C(2)	C(3)	-91.6(5)	O(3A)	C(1A)	C(2A)	C(3A)	91.1(5)
O(3)	C(30)	C(31)	C(32)	-19.9(6)	O(3A)	C(30A)	C(31A)	C(32A)	-163.1(4)
O(3)	C(30)	C(31)	C(36)	162.5(4)	O(3A)	C(30A)	C(31A)	C(36A)	17.5(7)
O(3)	C(30)	C(41)	C(42)	49.4(5)	O(3A)	C(30A)	C(41A)	C(42A)	129.0(4)
O(3)	C(30)	C(41)	C(46)	-133.2(4)	O(3A)	C(30A)	C(41A)	C(46A)	-52.9(5)
N(1)	C(2)	C(3)	C(4)	-165.4(5)	N(1A)	C(2A)	C(3A)	C(4A)	166.1(4)
N(1)	C(10)	C(11)	C(12)	-158.9(5)	N(1A)	C(10A)	C(11A)	C(12A)	158.4(5)
**A**	**B**	**C**	**D**	**Angle/°**	**A**	**B**	**C**	**D**	**Angle/°**
N(1)	C(10)	C(11)	C(16)	22.0(7)	N(1A)	C(10A)	C(11A)	C(16A)	-23.5(7)
C(1)	O(3)	C(30)	C(31)	-150.5(4)	C(1A)	O(3A)	C(30A)	C(31A)	151.9(4)
C(1)	O(3)	C(30)	C(41)	86.2(5)	C(1A)	O(3A)	C(30A)	C(41A)	-84.5(5)
C(1)	C(2)	C(3)	C(4)	72.3(6)	C(1A)	C(2A)	C(3A)	C(4A)	-71.9(6)
C(2)	N(1)	C(10)	O(1)	3.5(8)	C(2A)	N(1A)	C(10A)	O(1A)	-5.1(7)
C(2)	N(1)	C(10)	C(11)	-174.8(4)	C(2A)	N(1A)	C(10A)	C(11A)	174.6(4)
C(2)	C(3)	C(4)	C(5)	-97.9(7)	C(2A)	C(3A)	C(4A)	C(5A)	-83.7(7)
C(2)	C(3)	C(4)	C(9)	83.4(7)	C(2A)	C(3A)	C(4A)	C(9A)	98.1(6)
C(3)	C(4)	C(5)	C(6)	-178.7(6)	C(3A)	C(4A)	C(5A)	C(6A)	-179.7(5)
C(3)	C(4)	C(9)	C(8)	179.6(6)	C(3A)	C(4A)	C(9A)	C(8A)	179.7(5)
C(4)	C(5)	C(6)	C(7)	-0.5(11)	C(4A)	C(5A)	C(6A)	C(7A)	-0.6(10)
C(5)	C(4)	C(9)	C(8)	0.9(10)	C(5A)	C(4A)	C(9A)	C(8A)	1.5(9)
C(5)	C(6)	C(7)	C(8)	0.1(11)	C(5A)	C(6A)	C(7A)	C(8A)	2.7(11)
C(6)	C(7)	C(8)	C(9)	0.8(11)	C(6A)	C(7A)	C(8A)	C(9A)	-2.7(11)
C(7)	C(8)	C(9)	C(4)	-1.3(10)	C(7A)	C(8A)	C(9A)	C(4A)	0.6(10)
C(9)	C(4)	C(5)	C(6)	0.0(10)	C(9A)	C(4A)	C(5A)	C(6A)	-1.4(9)
C(10)	N(1)	C(2)	C(1)	-74.7(6)	C(10A)	N(1A)	C(2A)	C(1A)	77.0(6)
C(10)	N(1)	C(2)	C(3)	163.1(5)	C(10A)	N(1A)	C(2A)	C(3A)	-162.4(5)
C(10)	C(11)	C(12)	C(13)	179.9(5)	C(10A)	C(11A)	C(12A)	C(13A)	-179.3(5)
C(10)	C(11)	C(16)	C(15)	180.0(5)	C(10A)	C(11A)	C(16A)	C(15A)	-178.8(5)
C(11)	C(12)	C(13)	C(14)	-0.2(8)	C(11A)	C(12A)	C(13A)	C(14A)	-2.2(8)
C(12)	C(11)	C(16)	C(15)	0.9(8)	C(12A)	C(11A)	C(16A)	C(15A)	-0.6(8)
C(12)	C(13)	C(14)	C(15)	1.6(8)	C(12A)	C(13A)	C(14A)	C(15A)	0.0(8)
C(12)	C(13)	C(14)	C(17)	-178.6(5)	C(12A)	C(13A)	C(14A)	C(17A)	178.8(5)

C(13)	C(14)	C(15)	C(16)	-1.7(8)	C(13A)	C(14A)	C(15A)	C(16A)	1.9(8)
C(13)	C(14)	C(17)	C(18)	-37.8(8)	C(13A)	C(14A)	C(17A)	C(18A)	-139.6(5)
C(13)	C(14)	C(17)	C(22)	140.3(5)	C(13A)	C(14A)	C(17A)	C(22A)	40.7(7)
C(14)	C(15)	C(16)	C(11)	0.5(8)	C(14A)	C(15A)	C(16A)	C(11A)	-1.6(8)
C(14)	C(17)	C(18)	C(19)	179.9(5)	C(14A)	C(17A)	C(18A)	C(19A)	178.9(5)
C(14)	C(17)	C(22)	C(21)	-178.6(5)	C(14A)	C(17A)	C(22A)	C(21A)	179.8(5)
C(15)	C(14)	C(17)	C(18)	142.0(5)	C(15A)	C(14A)	C(17A)	C(18A)	39.1(7)
C(15)	C(14)	C(17)	C(22)	-39.9(7)	C(15A)	C(14A)	C(17A)	C(22A)	-140.5(5)
C(16)	C(11)	C(12)	C(13)	-1.0(8)	C(16A)	C(11A)	C(12A)	C(13A)	2.5(7)
C(17)	C(14)	C(15)	C(16)	178.5(5)	C(17A)	C(14A)	C(15A)	C(16A)	-176.9(5)
C(17)	C(18)	C(19)	C(20)	-1.8(8)	C(17A)	C(18A)	C(19A)	C(20A)	1.3(8)
C(18)	C(17)	C(22)	C(21)	-0.4(8)	C(18A)	C(17A)	C(22A)	C(21A)	0.1(7)
**A**	**B**	**C**	**D**	**Angle/˚**	**A**	**B**	**C**	**D**	**Angle/˚**
C(18)	C(19)	C(20)	C(21)	0.5(8)	C(18A)	C(19A)	C(20A)	C(21A)	-0.1(8)
C(19)	C(20)	C(21)	C(22)	0.8(8)	C(19A)	C(20A)	C(21A)	C(22A)	-1.2(8)
C(20)	C(21)	C(22)	C(17)	-0.8(8)	C(20A)	C(21A)	C(22A)	C(17A)	1.1(8)
C(22)	C(17)	C(18)	C(19)	1.8(8)	C(22A)	C(17A)	C(18A)	C(19A)	-1.4(8)
C(30)	O(3)	C(1)	O(2)	-6.2(7)	C(30A)	O(3A)	C(1A)	O(2A)	5.6(7)
C(30)	O(3)	C(1)	C(2)	170.3(4)	C(30A)	O(3A)	C(1A)	C(2A)	-171.3(4)
C(30)	C(31)	C(32)	C(33)	-178.8(5)	C(30A)	C(31A)	C(32A)	C(33A)	-178.8(5)
C(30)	C(31)	C(36)	C(35)	178.1(5)	C(30A)	C(31A)	C(36A)	C(35A)	177.7(5)
C(30)	C(41)	C(42)	C(43)	179.1(4)	C(30A)	C(41A)	C(42A)	C(43A)	178.7(4)
C(30)	C(41)	C(46)	C(45)	-177.6(4)	C(30A)	C(41A)	C(46A)	C(45A)	-179.9(4)
C(31)	C(30)	C(41)	C(42)	-70.4(5)	C(31A)	C(30A)	C(41A)	C(42A)	-110.5(5)
C(31)	C(30)	C(41)	C(46)	107.1(5)	C(31A)	C(30A)	C(41A)	C(46A)	67.5(5)
C(31)	C(32)	C(33)	C(34)	1.5(10)	C(31A)	C(32A)	C(33A)	C(34A)	0.4(8)
C(32)	C(31)	C(36)	C(35)	0.3(8)	C(32A)	C(31A)	C(36A)	C(35A)	-1.6(8)
C(32)	C(33)	C(34)	C(35)	-1.0(10)	C(32A)	C(33A)	C(34A)	C(35A)	-0.3(9)
C(33)	C(34)	C(35)	C(36)	0.1(9)	C(33A)	C(34A)	C(35A)	C(36A)	-0.8(9)
C(34)	C(35)	C(36)	C(31)	0.2(8)	C(34A)	C(35A)	C(36A)	C(31A)	1.8(9)
C(36)	C(31)	C(32)	C(33)	-1.2(9)	C(36A)	C(31A)	C(32A)	C(33A)	0.6(8)
C(41)	C(30)	C(31)	C(32)	101.8(6)	C(41A)	C(30A)	C(31A)	C(32A)	74.5(6)
C(41)	C(30)	C(31)	C(36)	-75.8(6)	C(41A)	C(30A)	C(31A)	C(36A)	-104.8(6)
C(41)	C(42)	C(43)	C(44)	-1.2(7)	C(41A)	C(42A)	C(43A)	C(44A)	0.9(8)
C(42)	C(41)	C(46)	C(45)	-0.2(7)	C(42A)	C(41A)	C(46A)	C(45A)	-1.8(7)
C(42)	C(43)	C(44)	C(45)	-0.8(8)	C(42A)	C(43A)	C(44A)	C(45A)	-1.3(8)
C(43)	C(44)	C(45)	C(46)	2.2(8)	C(43A)	C(44A)	C(45A)	C(46A)	0.2(8)

C(44)	C(45)	C(46)	C(41)	-1.8(8)	C(44A)	C(45A)	C(46A)	C(41A)	1.4(7)
C(46)	C(41)	C(42)	C(43)	1.6(7)	C(46A)	C(41A)	C(42A)	C(43A)	0.6(7)

Table S16 Hydrogen Atom Coordinates (Å×10^4) and Isotropic Displacement Parameters (Å^2×10^3) for **4al**.

**Atom**	***x***	***y***	***z***	**U(eq)**
H(1)	5170(20)	6920(30)	4380(20)	41(15)
H(2)	4123.42	7422.24	3403.44	44
H(3A)	3961.95	6223.24	4225.16	56
H(3B)	3862.73	6930.74	4834.38	56
H(5)	2730	7628.52	4981.36	57
H(6)	1490.97	7709.82	4788.05	87
H(7)	978.77	7007.91	3835.19	82
**Atom**	***x***	***y***	***z***	**U(eq)**
H(8)	1709.56	6215.81	3066.33	79
H(9)	2977.11	6105.55	3286.65	71
H(12)	6776.95	8131.85	3102.87	40
H(13)	7970.64	7613.3	3176.86	38
H(15)	7226.14	5448.61	4164.7	37
H(16)	6037.88	5977.72	4090.04	37
H(18)	8863.44	6569.4	2809.07	41
H(19)	10052.75	6036.88	2918.07	46
H(20)	10412.6	5351.49	3949.79	48
H(21)	9578.79	5167.49	4877.02	47
H(22)	8371.09	5653.39	4751	42
H(30)	3404.43	9619.21	4584.08	31
H(32)	2557.47	8785.74	3057.88	50
H(26)	2393.72	3803.62	46.02	36
H(32)	1408.89	7266.36	1944.33	29
H(33)	-376.83	7395.23	1613.92	33
H(34)	629.01	10043.36	3322.09	47
H(35)	1147.06	10875.24	4215.38	44
H(36)	2366.44	10661.95	4541.31	36
H(42)	3829.64	9935.25	2742.27	41
H(43)	4564.63	11056.46	2299.41	47
H(44)	5007.92	12123.43	3041.22	48
H(45)	4701.25	12109.04	4232.21	54
H(46)	4010.14	10961.15	4692.58	44
H(1A)	4760(20)	8100(30)	5670(20)	33(14)
H(2A)	5747.12	7567.84	6630.21	35
H(3AA)	6002.7	8052.39	5183.26	50
H(3AB)	5907.13	8769.02	5785.74	50
H(5A)	6885.04	8910.18	6701.11	59

H(6A)	8153.01	8841.45	6916.35	81
H(7A)	8886.44	7977.04	6193.91	82
H(8A)	8396.5	7294.1	5227.95	74
H(9A)	7146.33	7356.6	5008.86	53
H(12A)	3075.27	6872.86	6935.75	37
H(13A)	1887.74	7382.71	6808.68	40
H(15A)	2655.07	9548.27	5848	37
H(16A)	3836.85	9005.56	5917.51	36
H(18A)	1510.09	9342.58	5240.54	38
H(19A)	316.35	9824.6	5090.97	50
H(20A)	-533.67	9654.06	5997.56	45
H(21A)	-183.38	8994.16	7055.05	43
H(22A)	997.91	8463.58	7196.82	36
H(30A)	6505.07	5389.62	5429.81	33
**Atom**	***x***	***y***	***z***	**U(eq)**
H(32A)	7545.56	4337.87	5481.13	43
H(33A)	8767.1	4153.07	5802.19	43
H(34A)	9280.87	4989.77	6691.25	48
H(35A)	8568.81	6017.83	7254.75	58
H(36A)	7350.98	6251.57	6921.18	52
H(42A)	5861.56	4088.91	5287.33	47
H(43A)	5159.97	2948.24	5728.41	56
H(44A)	4908.91	2854.35	6925.29	50
H(45A)	5400.37	3881.32	7697.13	46
H(46A)	6131.95	4992.5	7267.95	40

Supplementary information for computational studies

DFT calculation

Computational Methods

All the calculations were performed using the Gaussian 16 program package [6]. Geometry optimizations were carried out at B3LYP [7a-b] functional with dispersion energy corrections by Grimme's dispersion correction D3BJ [7c] and def2-SVP [8] basis set for all atoms. Gibbs free energies were calculated under 298.15K and 1 atm pressure. Single-point energies were computed at the same function with the def2-TZVP basis set to obtain more accurate energetic results. The solvent effects of dichloromethane were considered using the SMD [9] model. Frequency calculations were conducted to characterize all the stationary points (no imaginary frequency) and transition state structures (only one imaginary frequency). Intrinsic reaction coordinate (IRC) [10] calculations were performed to confirm that each transition state connects to the corresponding reactants and products. To further investigate the potential interaction between the substrate and the catalyst, we conducted an independent gradient model based on Hirshfeld partition (IGMH) analysis [11] using the software Multiwfn 3.8 [12]. The wave functions for the analysis were generated at the B3LYP-D3(BJ)/ def2-SVP level of theory. The isosurface was visualized using VMD [13] with the isovalue of 0.008 (IGMH). All 3D molecular structures were visualized using the CYLview program [14].

Computational Results

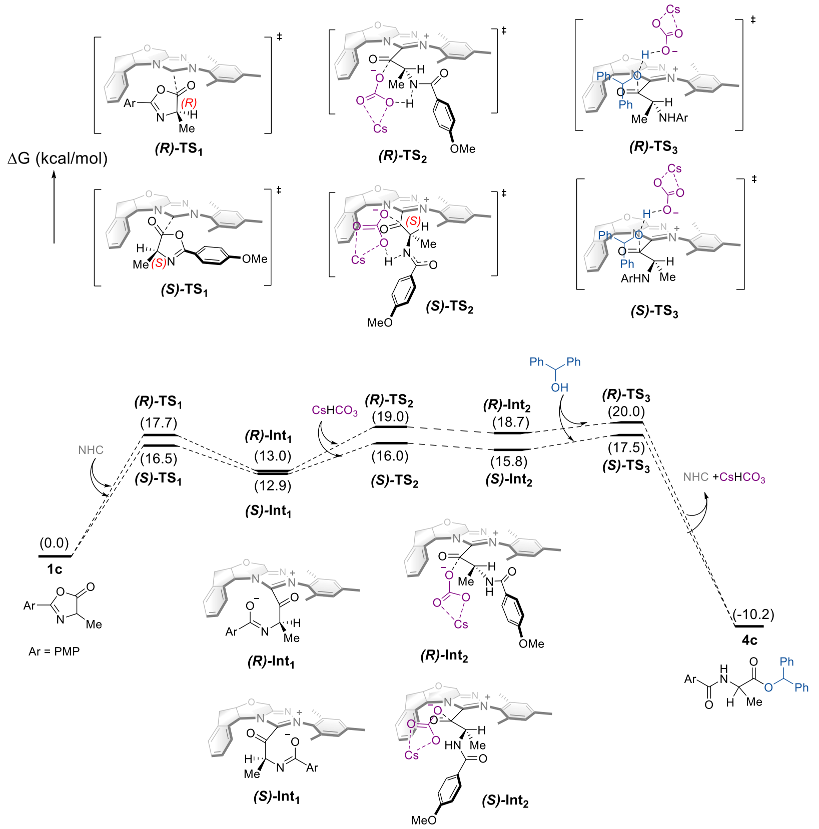

Figure S19. Free energy profile for the NHC-catalyzed alcoholysis of axazolone reaction calculated at the SMD(DCM)-B3LYP-D3(BJ)/def2tzvp // B3LYP-D3(BJ)/def2svp level of theory.

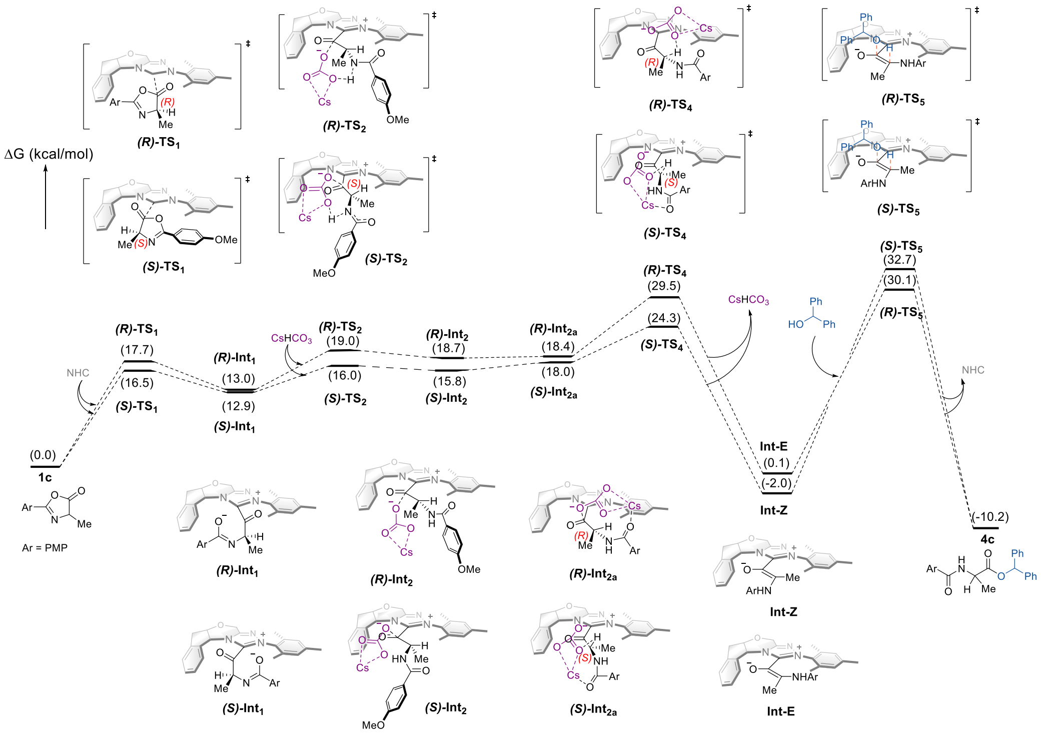

Figure S20. Free energy profile for an alternative pathway of the NHC-catalyzed alcoholysis of azlactone reaction calculated at the SMD(DCM)-B3LYP-D3(BJ)/def2-TZVP//B3LYP-D3(BJ)/def2-SVP level of theory.

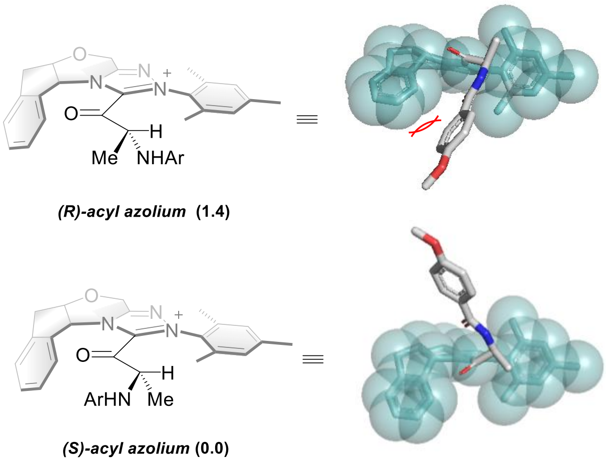

Figure S21. *R*-configured acyl azolium intermediate exhibits 1.4 kcal/mol energy higher than its *S* counterpart due to increased steric hindrance.

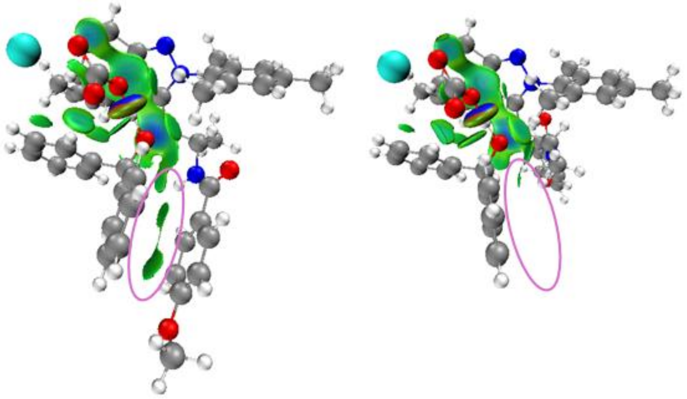

(S)-$\mathbf{TS_3}$ (0.0 kcal/mol) (R)-$\mathbf{TS_3}$ (2.5 kcal/mol)

Figure S22. IGMH analysis (isovalue=0.008) of non-covalent interactions between acyl azolium, alcohol and base fragments in both (R)-$\mathbf{TS_3}$ and (S)-$\mathbf{TS_3}$.

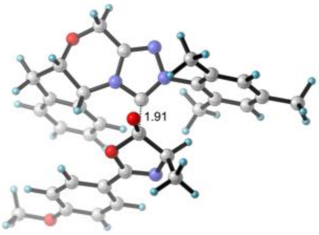

TS1R

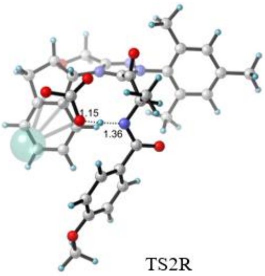

TS2R

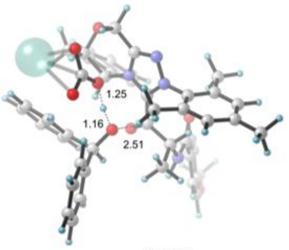

TS3R

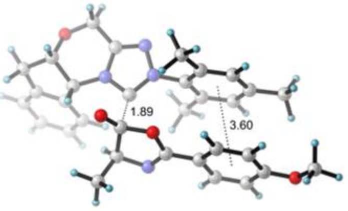

TS1S

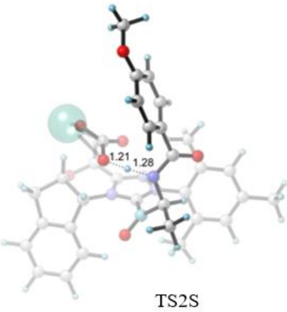

TS2S

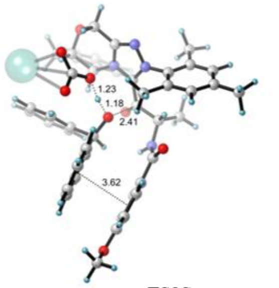

TS3S

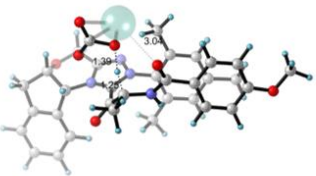

TS4R

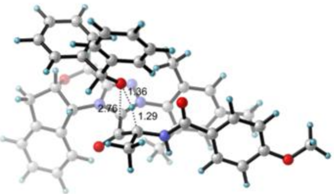

TS5R

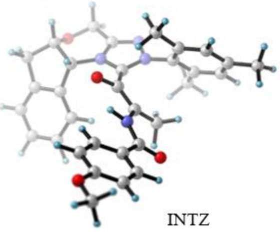
INTZ

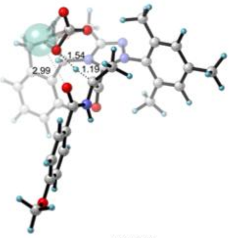

TS4S

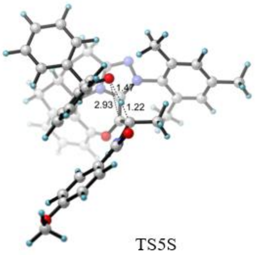

TS5S

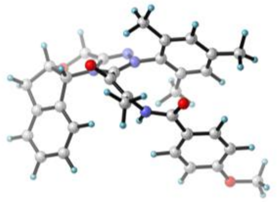
INT1E

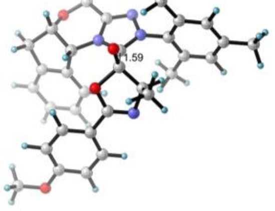

INT1R

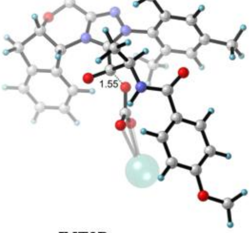

INT2R

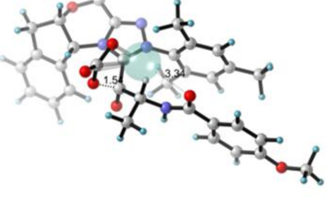
INT2aR

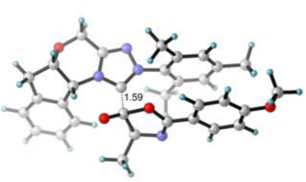

INT1S

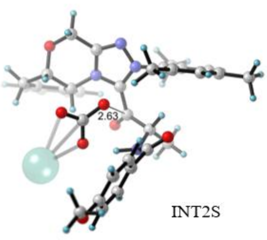

INT2S

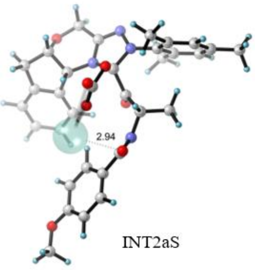

INT2aS

Figure S23. Optimized geometries with partial structural parameters (distances in Å) for the species involved in the energy profiles.

Chiral separation results

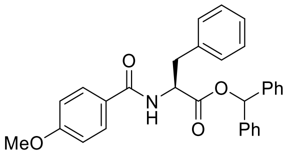

(**4a**)

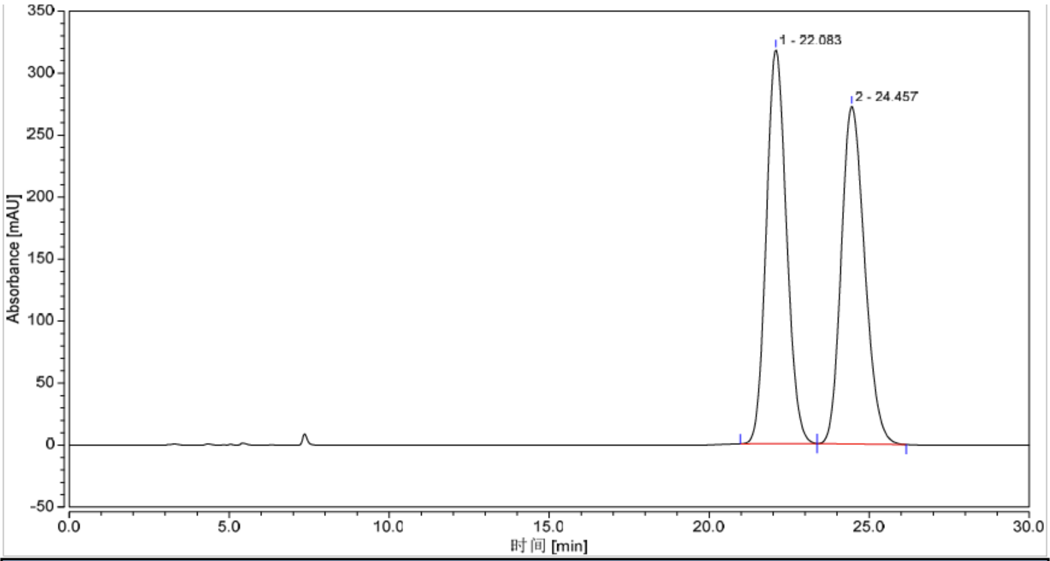

Integration Results							
No.	Peak Name	Retention Time min	Area mAu*min	Height mAU	Relative Area %	Relative Height %	Amount n.a.
1		22.083	232.166	317.917	50.12	53.84	n.a.
2		24.457	231.055	272.542	49.88	46.16	n.a.
**Total:**			**463.221**	**590.458**	**100.00**	**100.00**	

Figure S24. HPLC spectrum of racemic **4a**

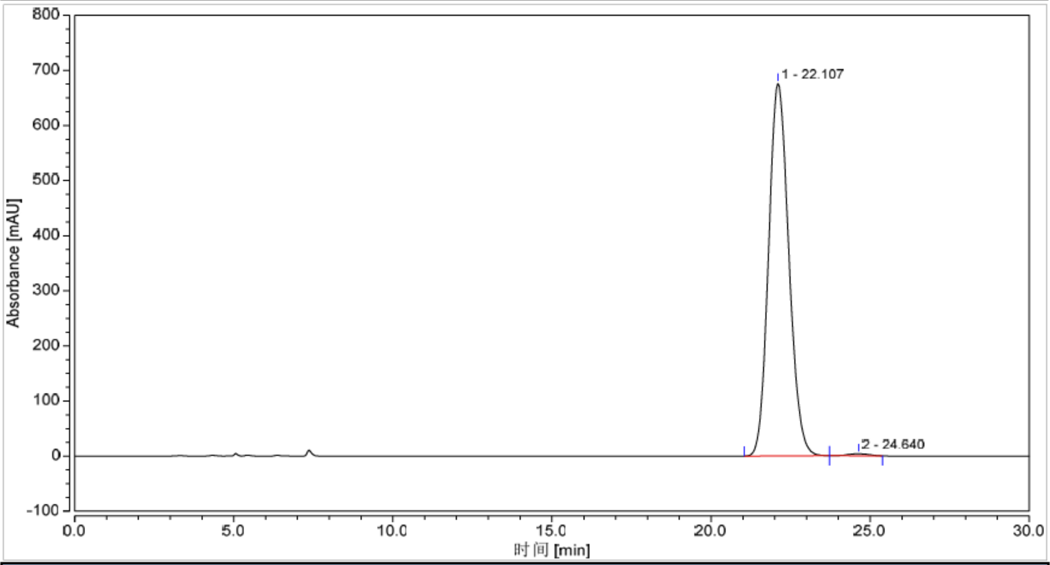

Integration Results							
No.	Peak Name	Retention Time min	Area mAu*min	Height mAU	Relative Area %	Relative Height %	Amount n.a.
1		22.107	497.221	676.104	99.44	99.45	n.a.
2		24.640	2.809	3.721	0.56	0.55	n.a.
**Total:**			**500.030**	**679.825**	**100.00**	**100.00**	

Figure S25. HPLC spectrum of racemic **4a**

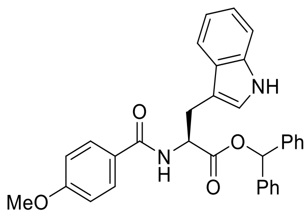

(4b)

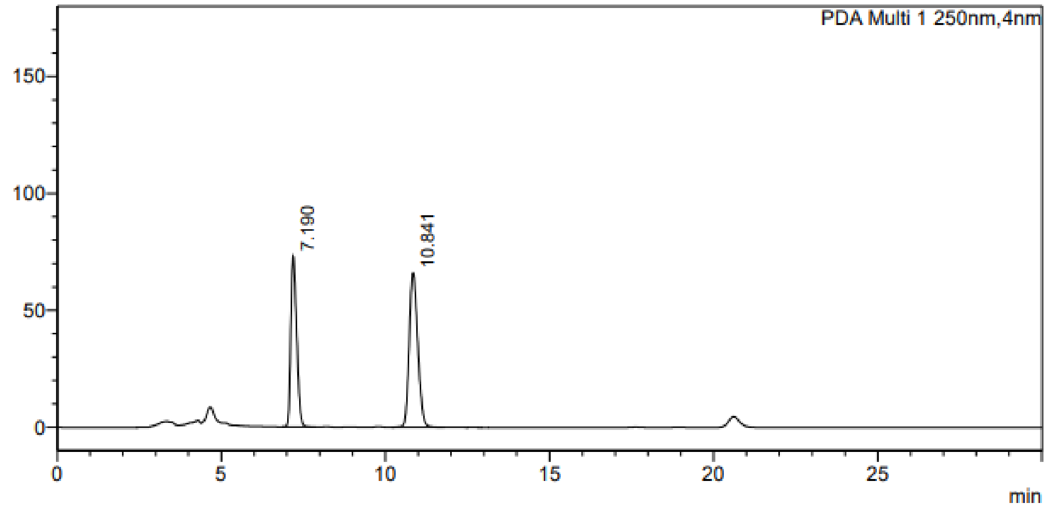

<Peak Table>

PDA Ch1 250nm

Peak#	Ret. Time	Area	Height	Area%	Height%
1	7.190	914048	73043	43.205	52.526
2	10.841	1201575	66017	56.795	47.474
Total		2115623	139060	100.000	100.000

Figure S26. HPLC spectrum of racemic **4b**

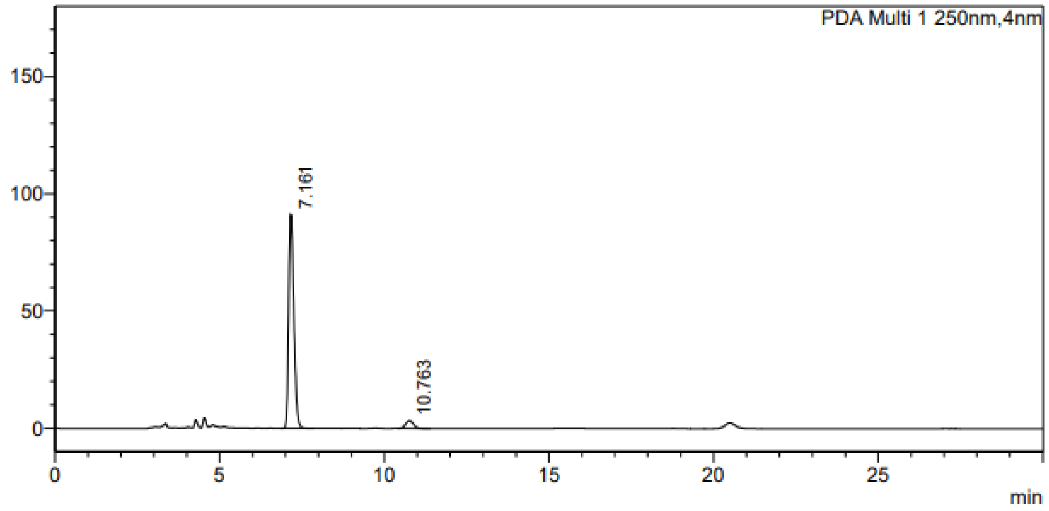

<Peak Table>

PDA Ch1 250nm

Peak#	Ret. Time	Area	Height	Area%	Height%
1	7.161	987816	91094	95.011	96.558
2	10.763	51869	3247	4.989	3.442
Total		1039685	94341	100.000	100.000

Figure S27. HPLC spectrum of racemic **4b**

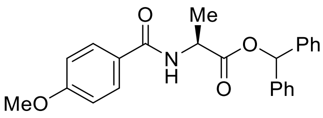

(4c)

Chromatogram

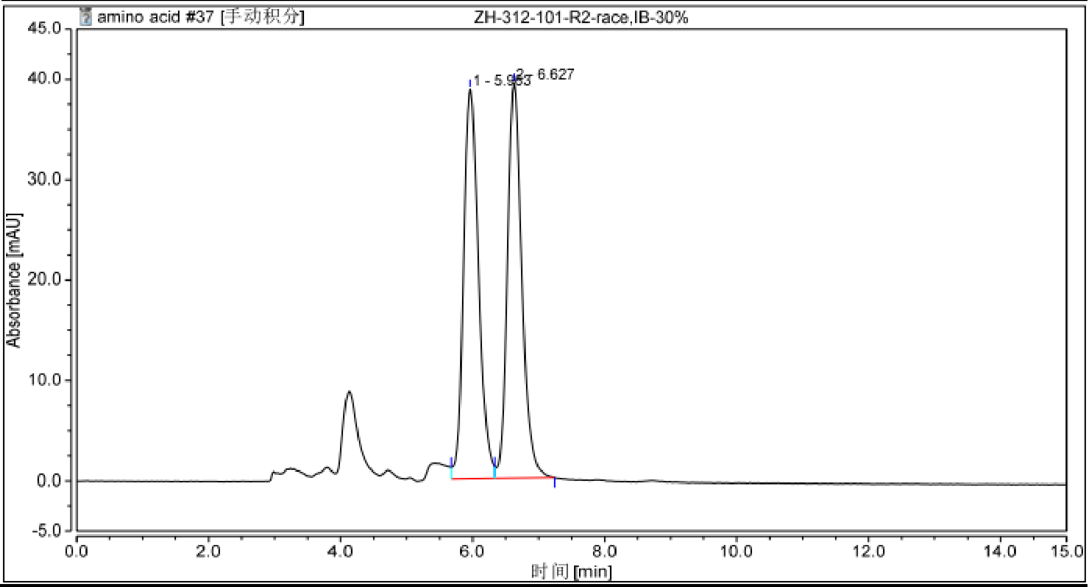

Integration Results

No.	Peak Name	Retention Time min	Area mAu*min	Height mAU	Relative Area %	Relative Height %	Amount n.a.
1		5.963	10.200	38.753	50.56	49.66	n.a.
2		6.627	9.974	39.278	49.44	50.34	n.a.
**Total:**			**20.174**	**78.031**	**100.00**	**100.00**	

Figure S28. HPLC spectrum of racemic **4c**

Chromatogram

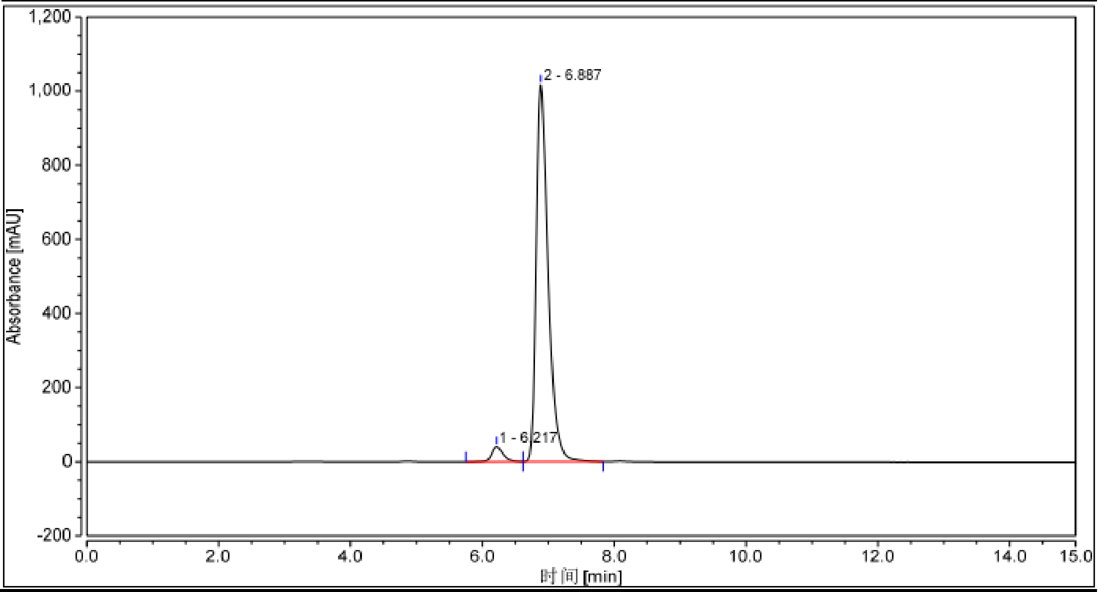

Integration Results

No.	Peak Name	Retention Time min	Area mAu*min	Height mAU	Relative Area %	Relative Height %	Amount n.a.
1		6.217	8.134	40.837	3.67	3.86	n.a.
2		6.887	213.621	1017.132	96.33	96.14	n.a.
**Total:**			**221.755**	**1057.969**	**100.00**	**100.00**	

Figure S29. HPLC spectrum of racemic **4c**

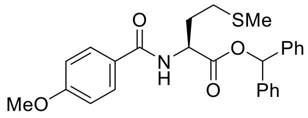

(4d)

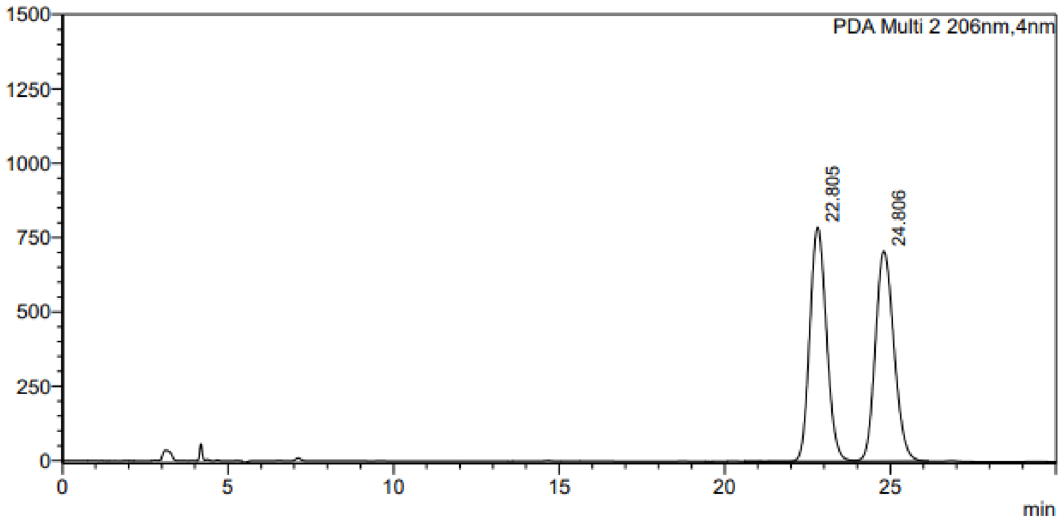

<Peak Table>

PDA Ch2 206nm

Peak#	Ret. Time	Area	Height	Area%	Height%
1	22.805	27544673	787303	49.792	52.700
2	24.806	27774249	706623	50.208	47.300
Total		55318922	1493926	100.000	100.000

Figure S30. HPLC spectrum of racemic **4d**

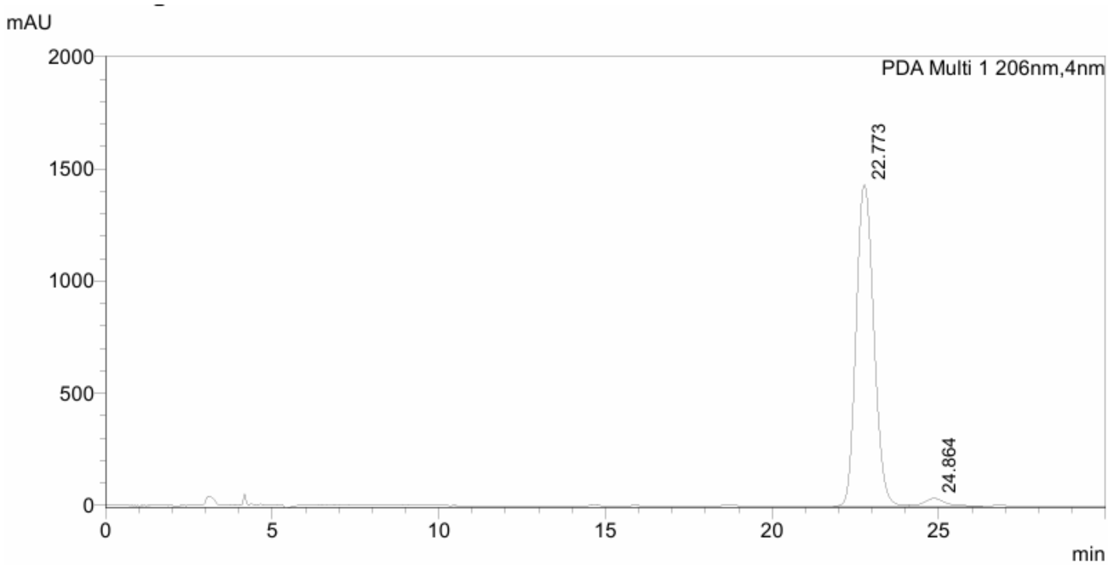

<Peak Table>

PDA Ch1 206nm

Peak#	Ret. Time	Area	Height	Area%	Height%
1	22.773	52664574	1429236	97.567	97.782
2	24.864	1313107	32413	2.433	2.218
Total		53977681	1461649	100.000	100.000

Figure S31. HPLC spectrum of racemic **4d**

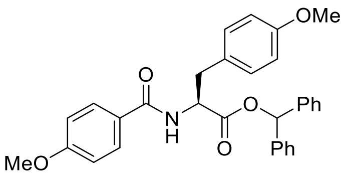

(4e)

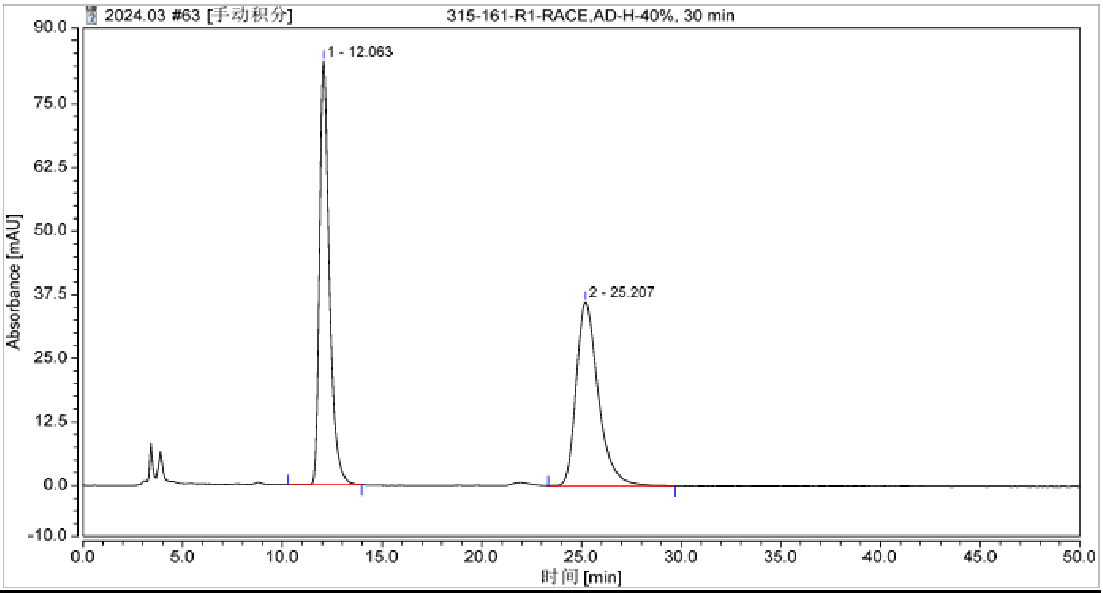

Integration Results

No.	Peak Name	Retention Time min	Area mAu*min	Height mAU	Relative Area %	Relative Height %	Amount n.a.
1		12.063	47.864	83.358	50.76	69.72	n.a.
2		25.207	46.429	36.203	49.24	30.28	n.a.
Total:			94.293	119.561	100.00	100.00	

Figure S32. HPLC spectrum of racemic **4e**

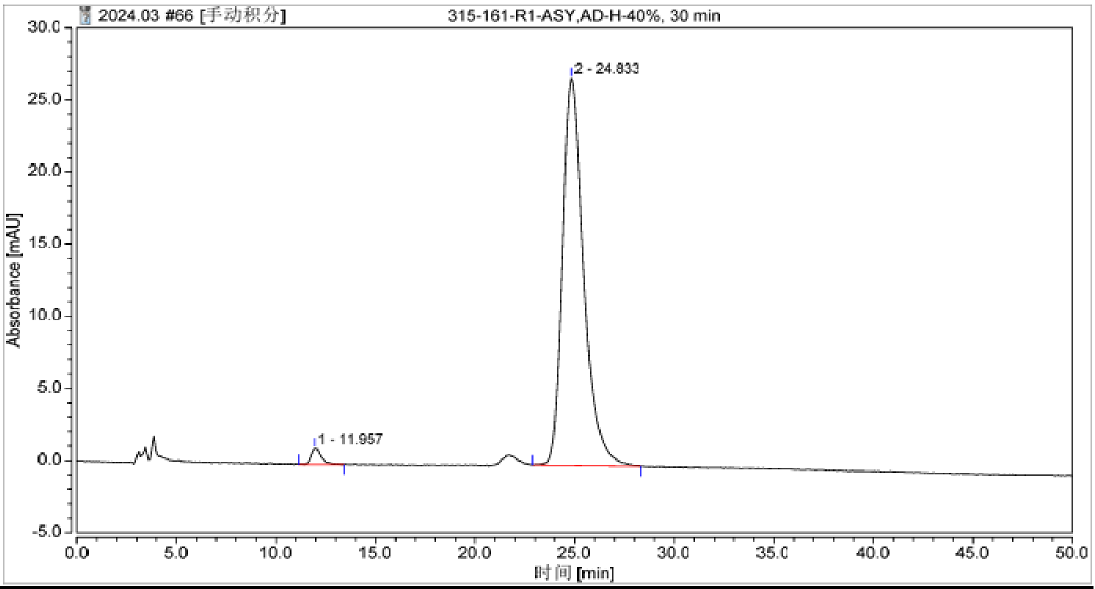

Integration Results

No.	Peak Name	Retention Time min	Area mAu*min	Height mAU	Relative Area %	Relative Height %	Amount n.a.
1		11.957	0.660	1.111	1.95	3.97	n.a.
2		24.833	33.242	26.865	98.05	96.03	n.a.
Total:			33.902	27.976	100.00	100.00	

Figure S33. HPLC spectrum of **4e**

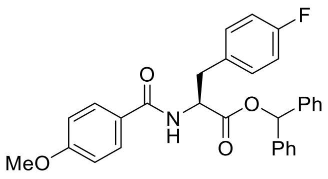

(4f)

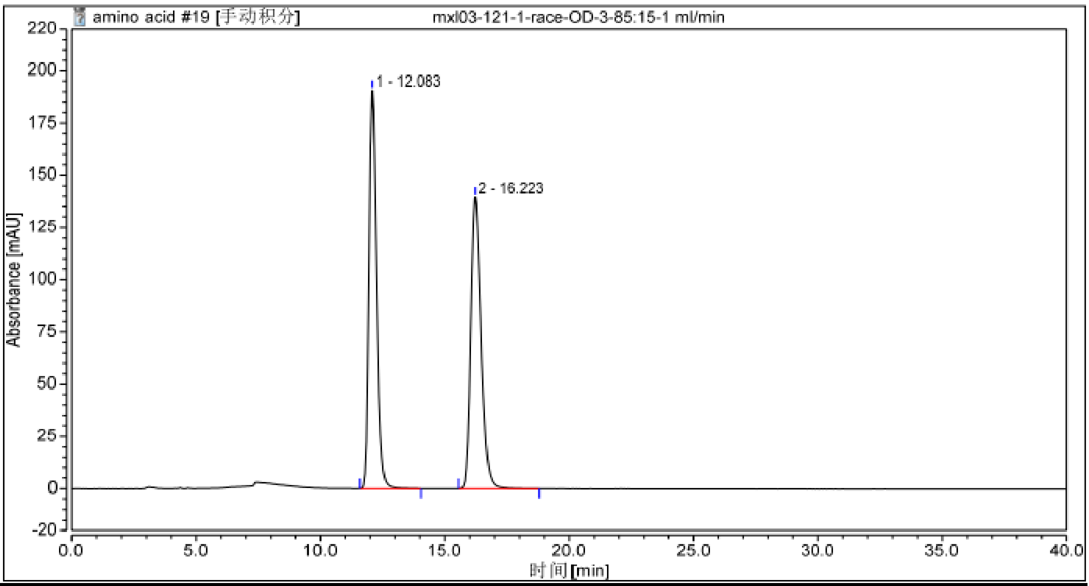

Integration Results							
No.	Peak Name	Retention Time min	Area mAu*min	Height mAU	Relative Area %	Relative Height %	Amount n.a.
1		12.083	66.807	190.540	49.97	57.71	n.a.
2		16.223	66.879	139.613	50.03	42.29	n.a.
**Total:**			**133.686**	**330.153**	**100.00**	**100.00**	

Figure S34. HPLC spectrum of racemic **4f**

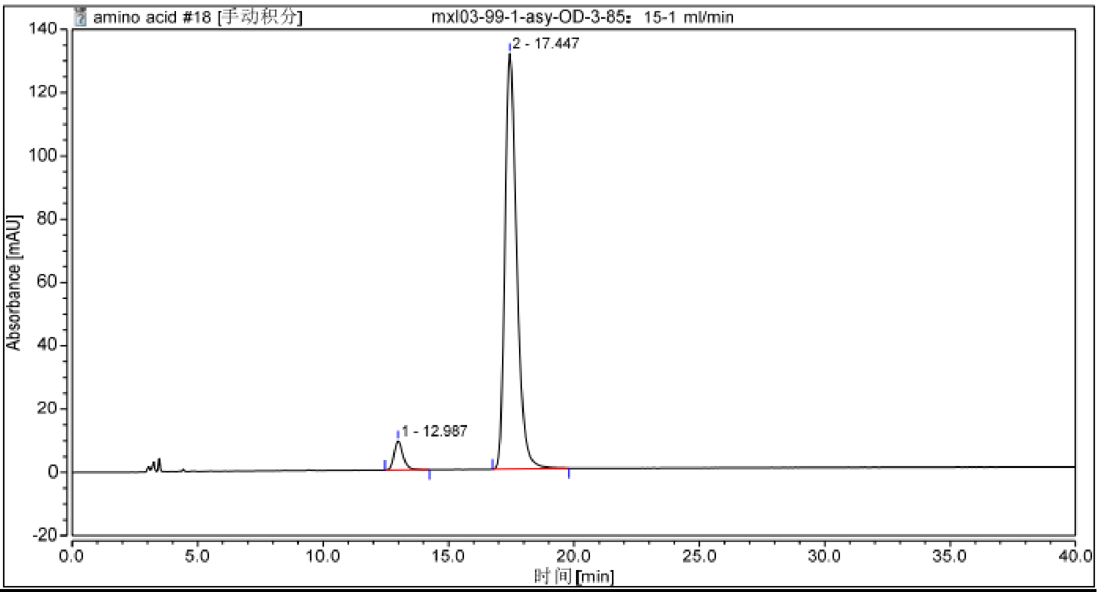

Integration Results							
No.	Peak Name	Retention Time min	Area mAu*min	Height mAU	Relative Area %	Relative Height %	Amount n.a.
1		12.987	3.736	9.156	4.96	6.52	n.a.
2		17.447	71.565	131.370	95.04	93.48	n.a.
**Total:**			**75.301**	**140.525**	**100.00**	**100.00**	

Figure S35. HPLC spectrum of **4f**

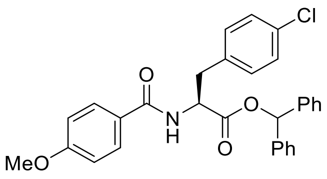

(4g)

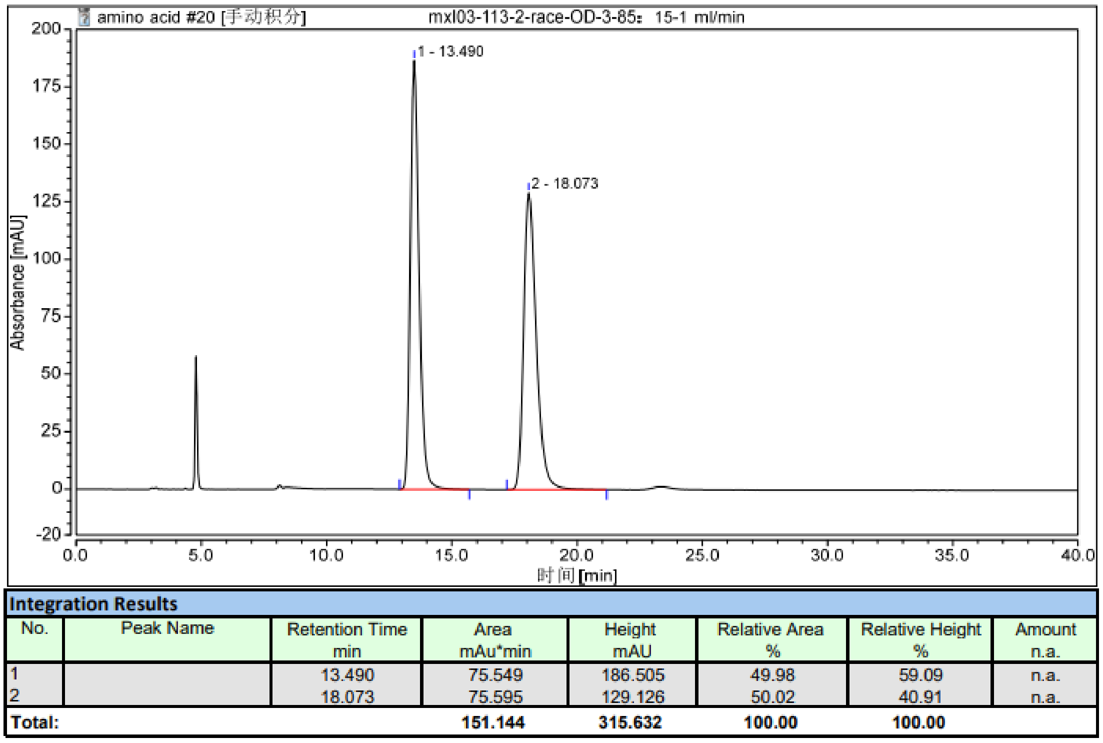

Integration Results							
No.	Peak Name	Retention Time<br>min	Area<br>mAu*min	Height<br>mAU	Relative Area<br>%	Relative Height<br>%	Amount<br>n.a.
1		13.490	75.549	186.505	49.98	59.09	n.a.
2		18.073	75.595	129.126	50.02	40.91	n.a.
**Total:**			**151.144**	**315.632**	**100.00**	**100.00**	

Figure S36. HPLC spectrum of racemic **4g**

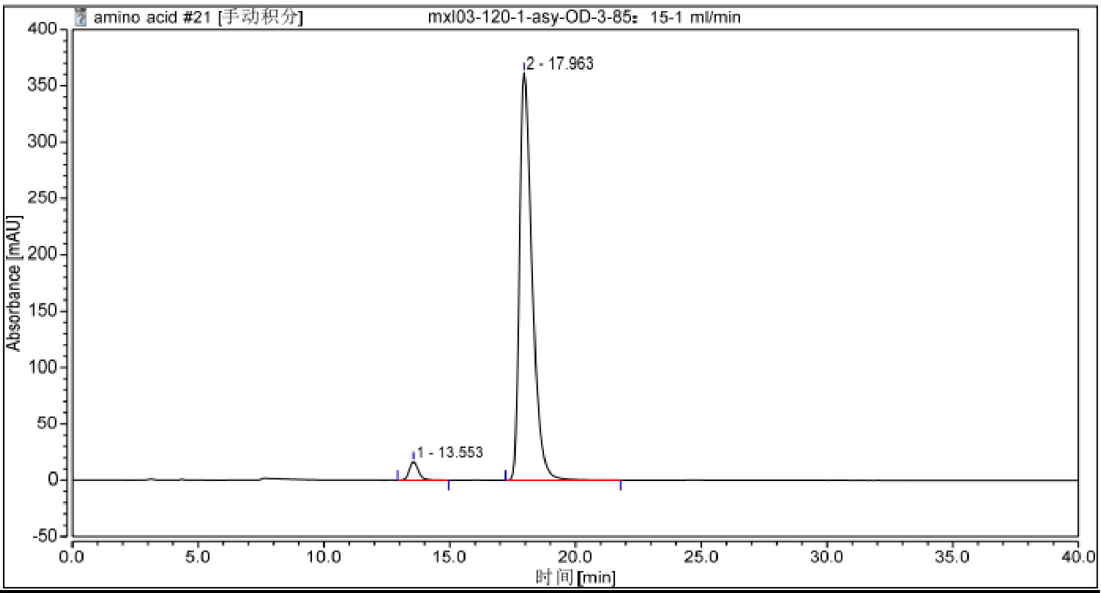

Integration Results							
No.	Peak Name	Retention Time<br>min	Area<br>mAu*min	Height<br>mAU	Relative Area<br>%	Relative Height<br>%	Amount<br>n.a.
1		13.553	6.975	16.229	3.20	4.29	n.a.
2		17.963	211.003	361.868	96.80	95.71	n.a.
**Total:**			**217.978**	**378.098**	**100.00**	**100.00**	

Figure S37. HPLC spectrum of **4g**

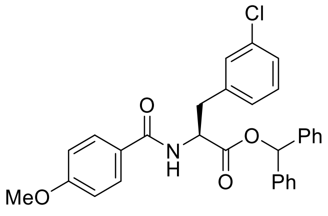

(**4h**)

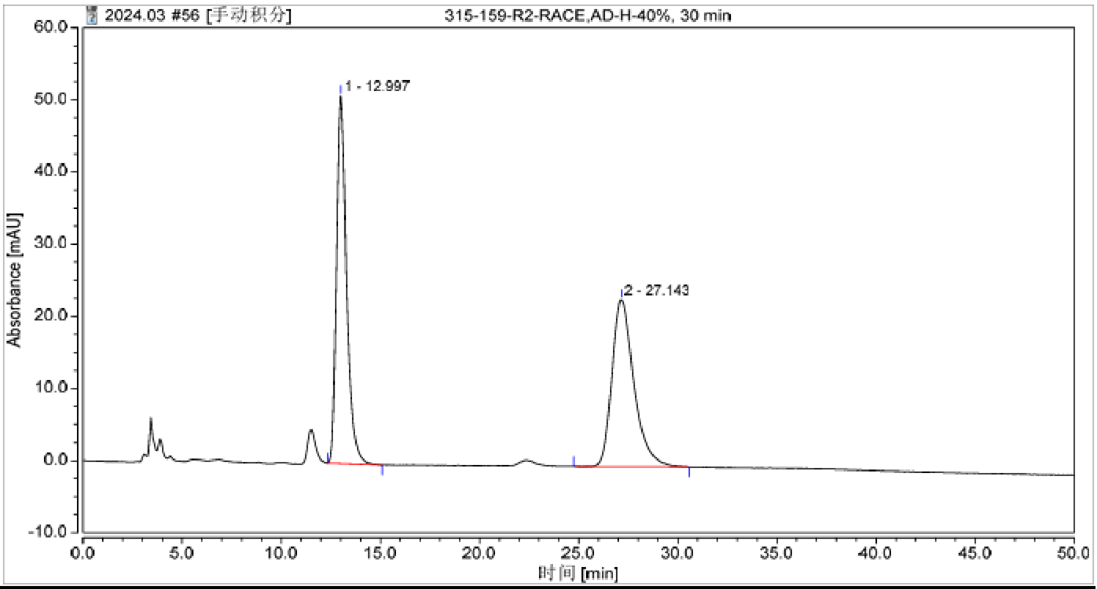

Integration Results							
No.	Peak Name	Retention Time min	Area mAu*min	Height mAU	Relative Area %	Relative Height %	Amount n.a.
1		12.997	29.871	50.966	50.11	68.75	n.a.
2		27.143	29.743	23.164	49.89	31.25	n.a.
**Total:**			**59.613**	**74.131**	**100.00**	**100.00**	

Figure S38. HPLC spectrum of racemic **4h**

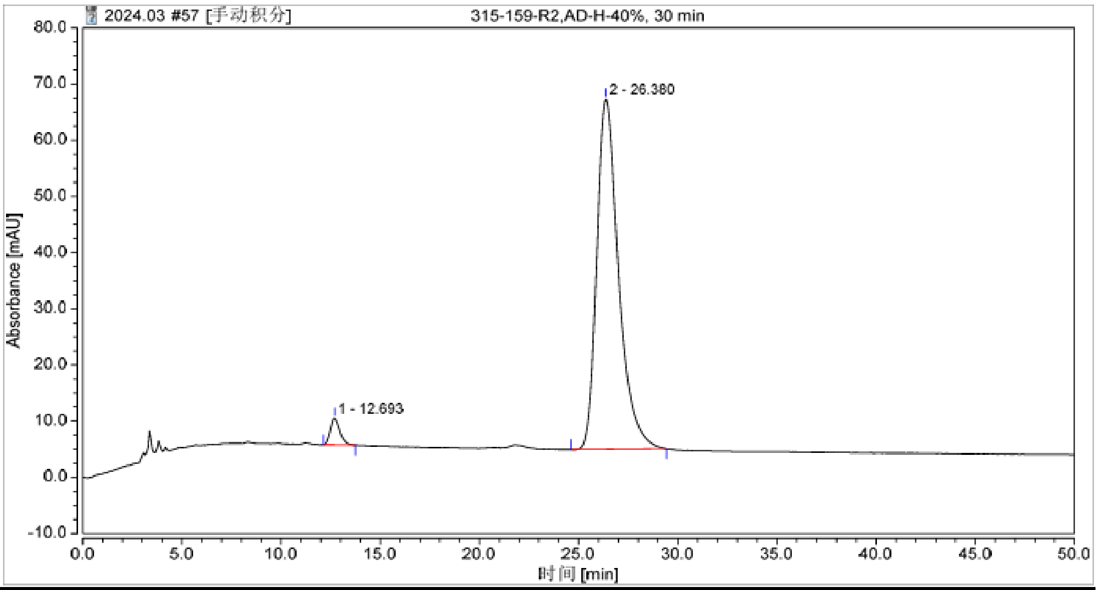

Integration Results							
No.	Peak Name	Retention Time min	Area mAu*min	Height mAU	Relative Area %	Relative Height %	Amount n.a.
1		12.693	2.624	4.778	3.21	7.12	n.a.
2		26.380	79.257	62.376	96.79	92.88	n.a.
**Total:**			**81.881**	**67.155**	**100.00**	**100.00**	

Figure S39. HPLC spectrum of **4h**

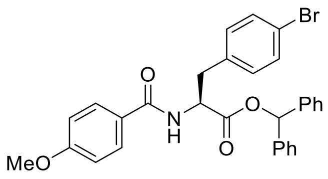

(4i)

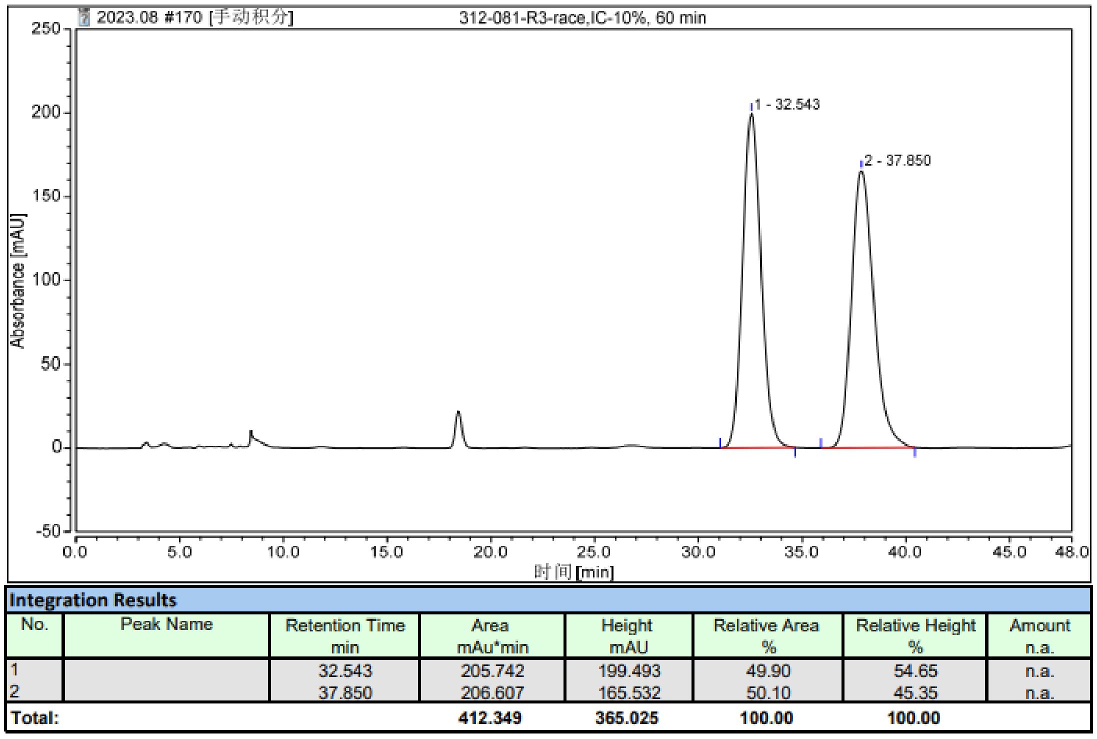

Integration Results							
No.	Peak Name	Retention Time min	Area mAu*min	Height mAU	Relative Area %	Relative Height %	Amount n.a.
1		32.543	205.742	199.493	49.90	54.65	n.a.
2		37.850	206.607	165.532	50.10	45.35	n.a.
Total:			412.349	365.025	100.00	100.00	

Figure S40. HPLC spectrum of racemic **4i**

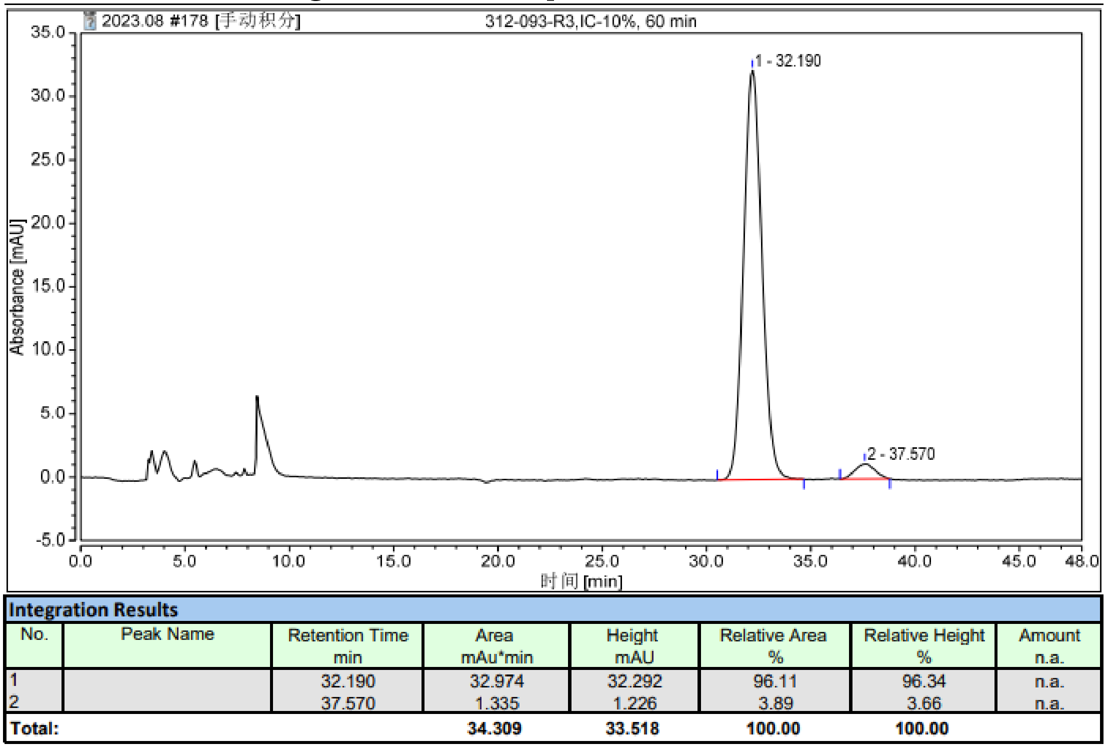

Integration Results							
No.	Peak Name	Retention Time min	Area mAu*min	Height mAU	Relative Area %	Relative Height %	Amount n.a.
1		32.190	32.974	32.292	96.11	96.34	n.a.
2		37.570	1.335	1.226	3.89	3.66	n.a.
Total:			34.309	33.518	100.00	100.00	

Figure S41. HPLC spectrum of **4i**

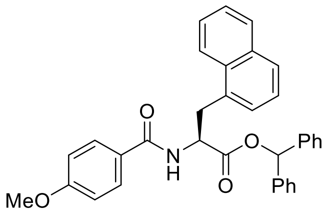

(4j)

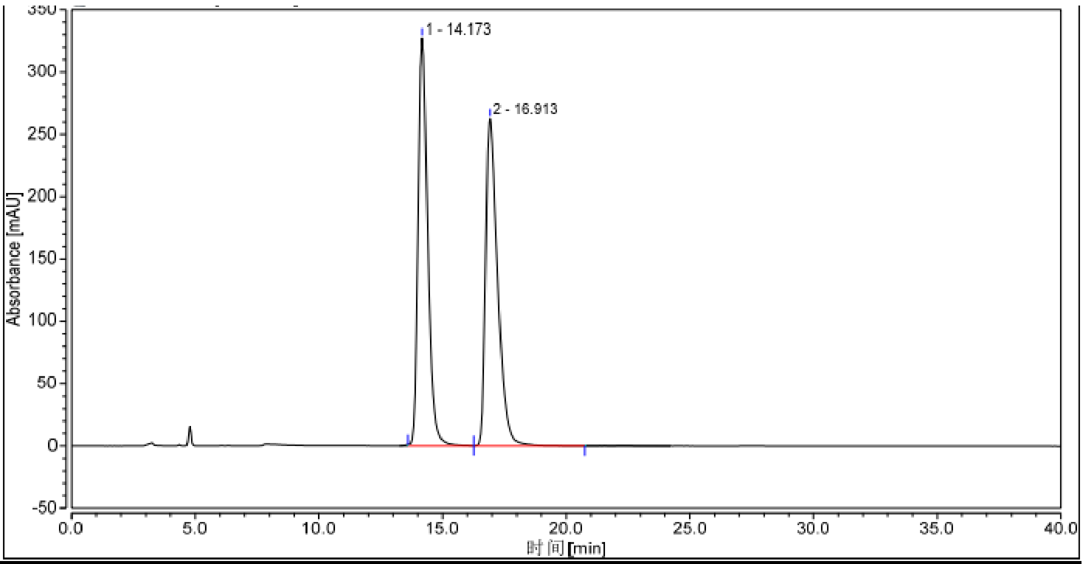

Integration Results							
No.	Peak Name	Retention Time min	Area mAu*min	Height mAU	Relative Area %	Relative Height %	Amount n.a.
1		14.173	147.690	327.103	49.85	55.47	n.a.
2		16.913	148.558	262.549	50.15	44.53	n.a.
Total:			296.248	589.652	100.00	100.00	

Figure S42. HPLC spectrum of racemic **4j**

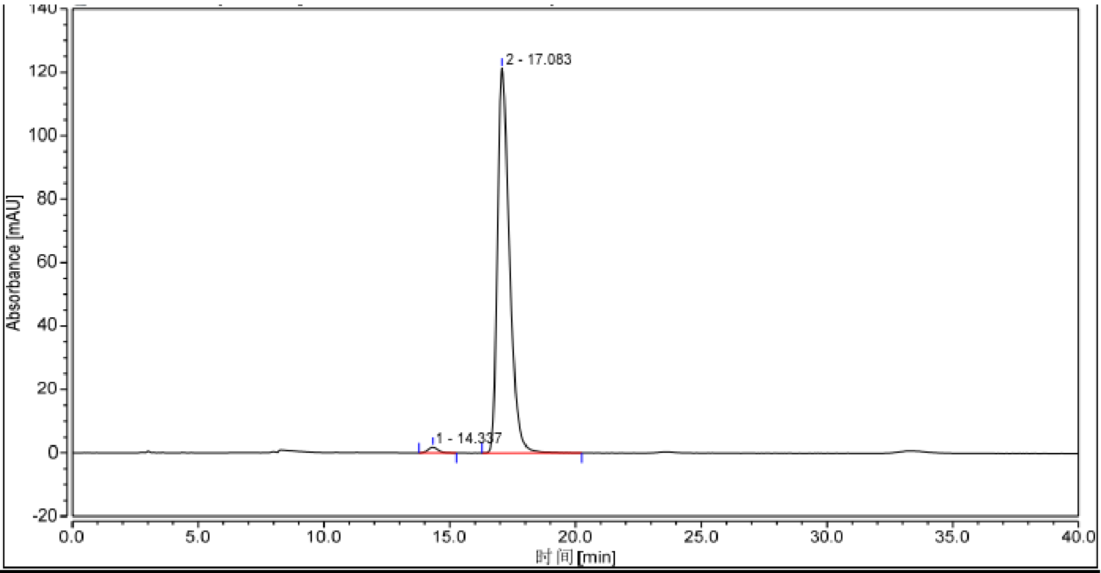

Integration Results							
No.	Peak Name	Retention Time min	Area mAu*min	Height mAU	Relative Area %	Relative Height %	Amount n.a.
1		14.337	0.878	1.871	1.28	1.52	n.a.
2		17.083	67.726	121.281	98.72	98.48	n.a.
Total:			68.604	123.152	100.00	100.00	

Figure S43. HPLC spectrum of **4j**

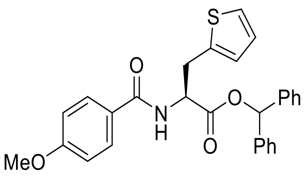

(4k)

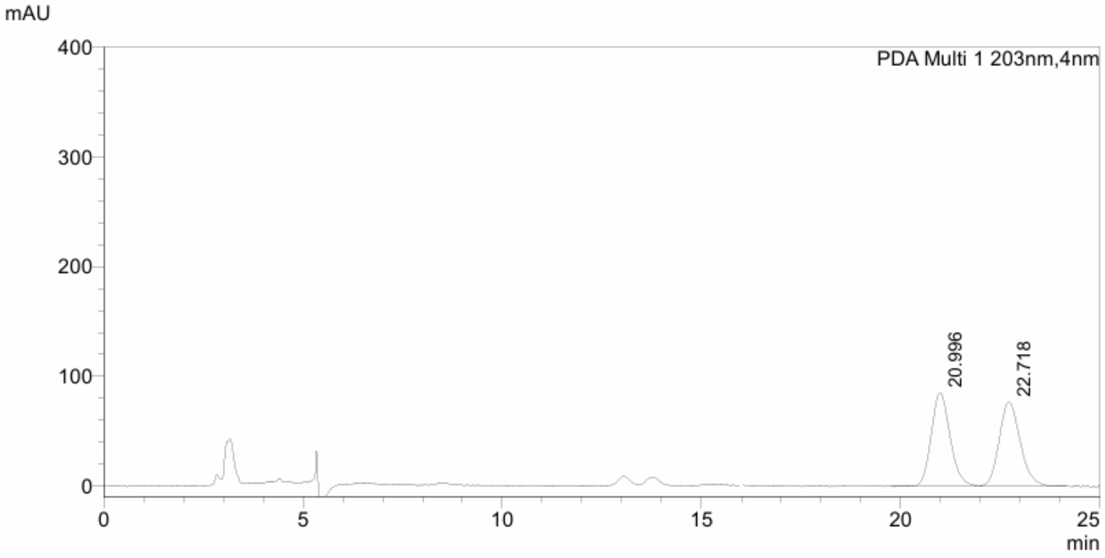

<Peak Table>

PDA Ch1 203nm

Peak#	Ret. Time	Area	Height	Area%	Height%
1	20.996	2839975	85277	49.855	52.634
2	22.718	2856484	76742	50.145	47.366
Total		5696460	162018	100.000	100.000

Figure S44. HPLC spectrum of racemic **4k**

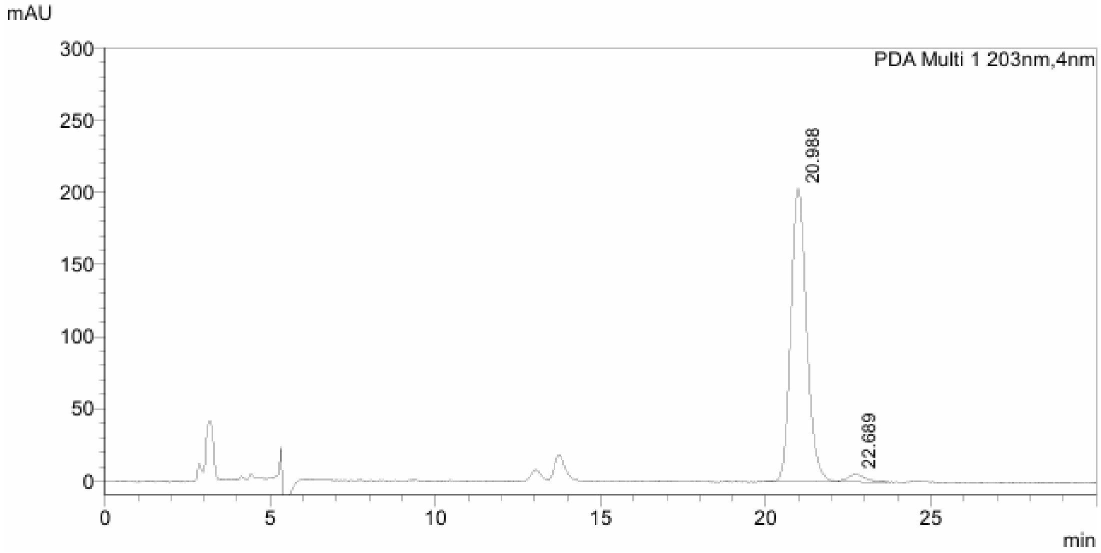

<Peak Table>

PDA Ch1 203nm

Peak#	Ret. Time	Area	Height	Area%	Height%
1	20.988	6746656	202780	97.074	97.321
2	22.689	203325	5582	2.926	2.679
Total		6949981	208361	100.000	100.000

Figure S45. HPLC spectrum of **4k**

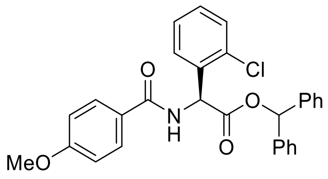

(4l)

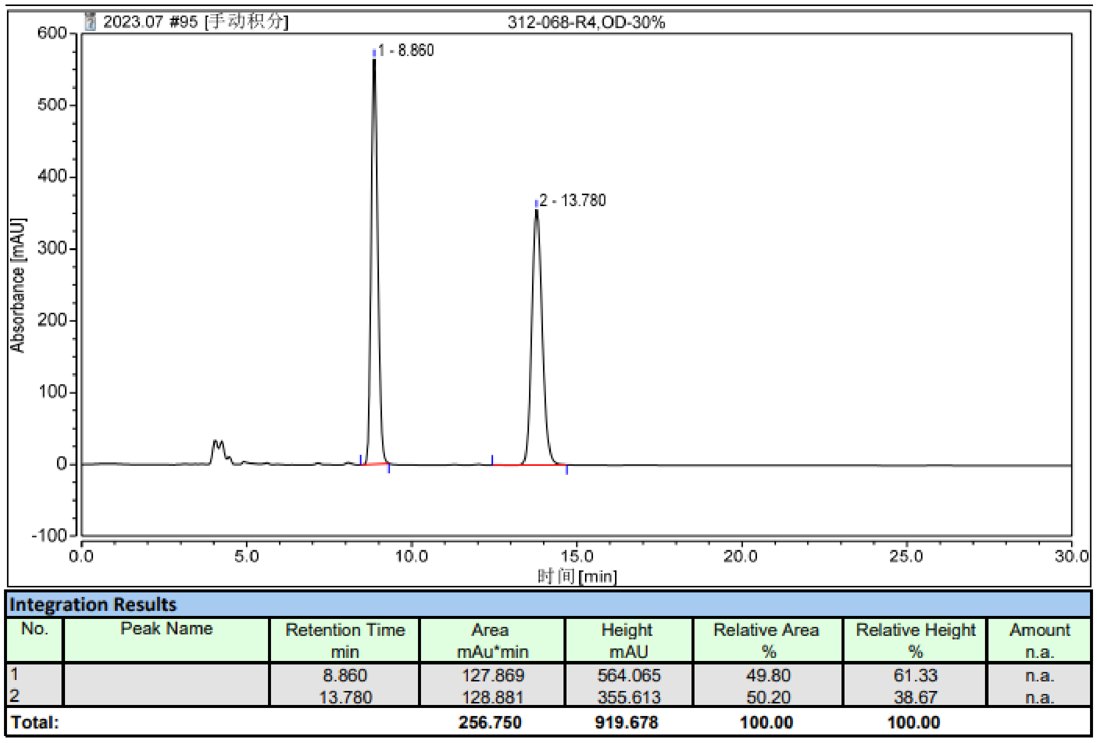

Integration Results							
No.	Peak Name	Retention Time min	Area mAu*min	Height mAU	Relative Area %	Relative Height %	Amount n.a.
1		8.860	127.869	564.065	49.80	61.33	n.a.
2		13.780	128.881	355.613	50.20	38.67	n.a.
**Total:**			**256.750**	**919.678**	**100.00**	**100.00**	

Figure S46. HPLC spectrum of racemic **4l**

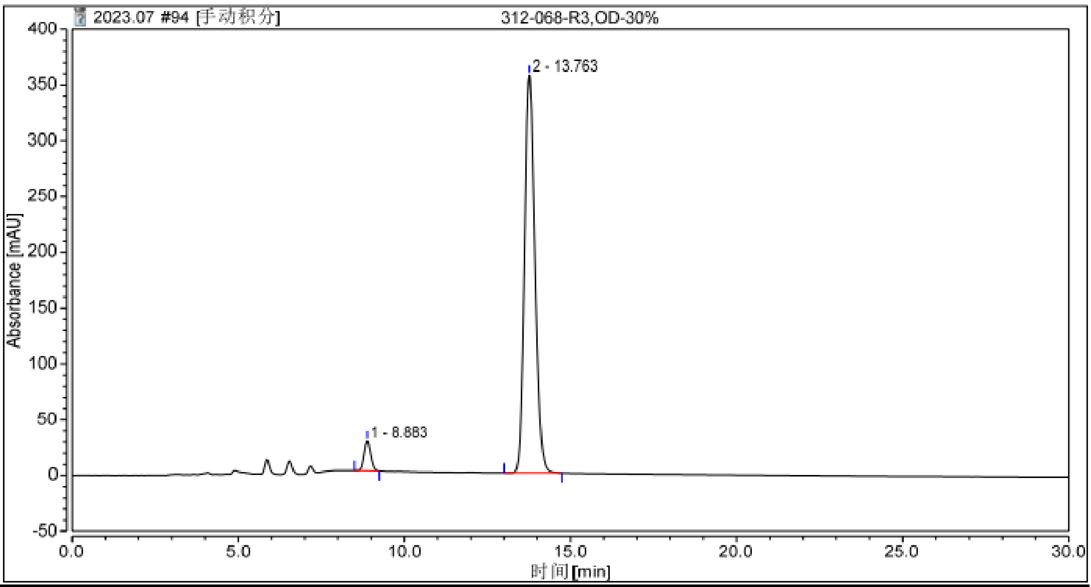

Integration Results							
No.	Peak Name	Retention Time min	Area mAu*min	Height mAU	Relative Area %	Relative Height %	Amount n.a.
1		8.883	6.065	26.635	4.50	6.95	n.a.
2		13.763	128.633	356.625	95.50	93.05	n.a.
**Total:**			**134.698**	**383.260**	**100.00**	**100.00**	

Figure S47. HPLC spectrum of **4l**

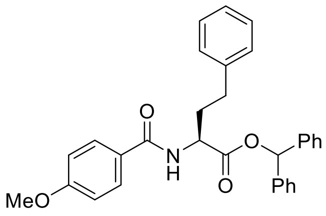

(4m)

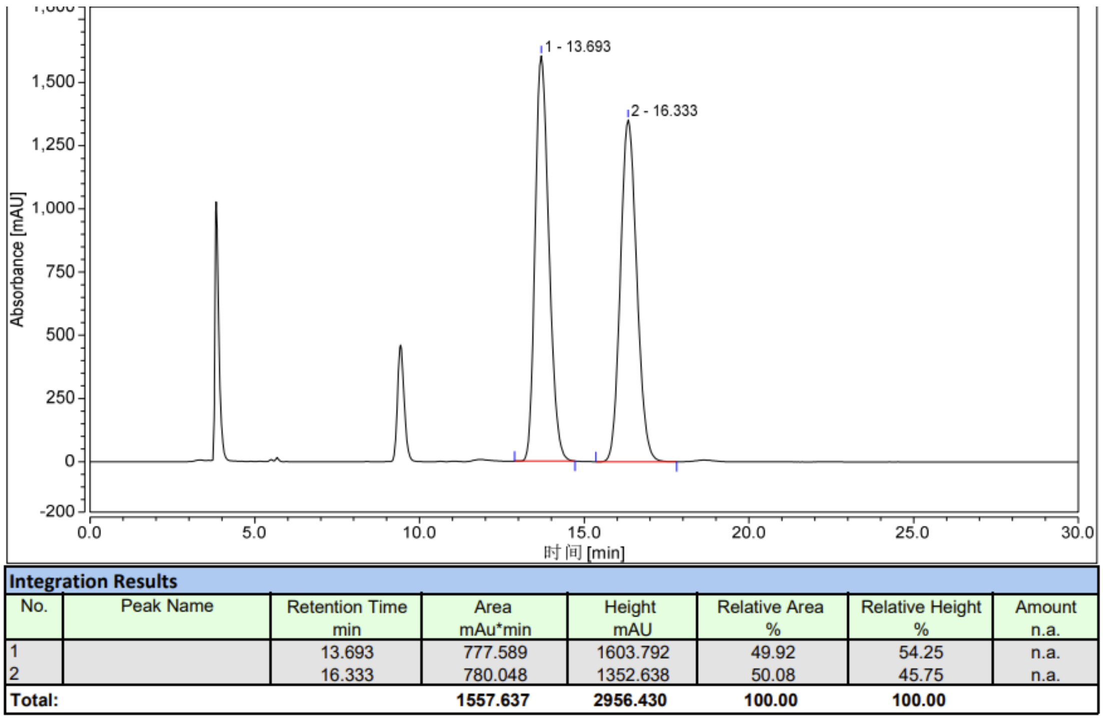

Integration Results

No.	Peak Name	Retention Time min	Area mAu*min	Height mAU	Relative Area %	Relative Height %	Amount n.a.
1		13.693	777.589	1603.792	49.92	54.25	n.a.
2		16.333	780.048	1352.638	50.08	45.75	n.a.
**Total:**			**1557.637**	**2956.430**	**100.00**	**100.00**	

Figure S48. HPLC spectrum of racemic **4m**

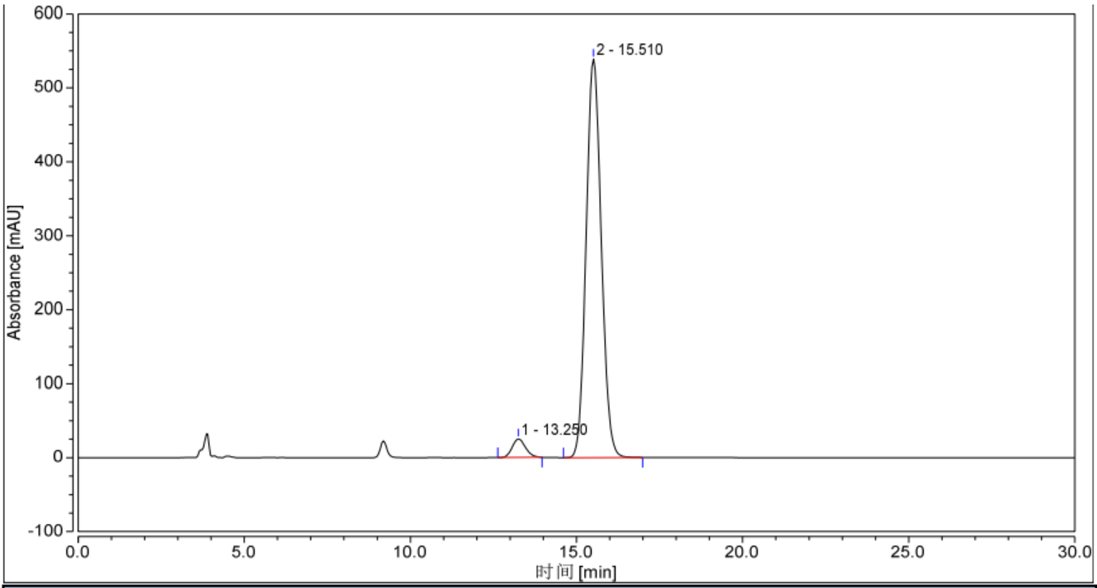

Integration Results

No.	Peak Name	Retention Time min	Area mAu*min	Height mAU	Relative Area %	Relative Height %	Amount n.a.
1		13.250	11.561	24.943	3.91	4.42	n.a.
2		15.510	284.325	538.755	96.09	95.58	n.a.
**Total:**			**295.886**	**563.698**	**100.00**	**100.00**	

Figure S49. HPLC spectrum of **4m**

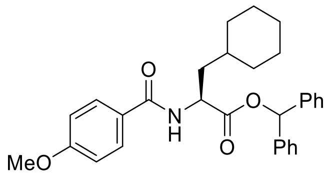

(4n)

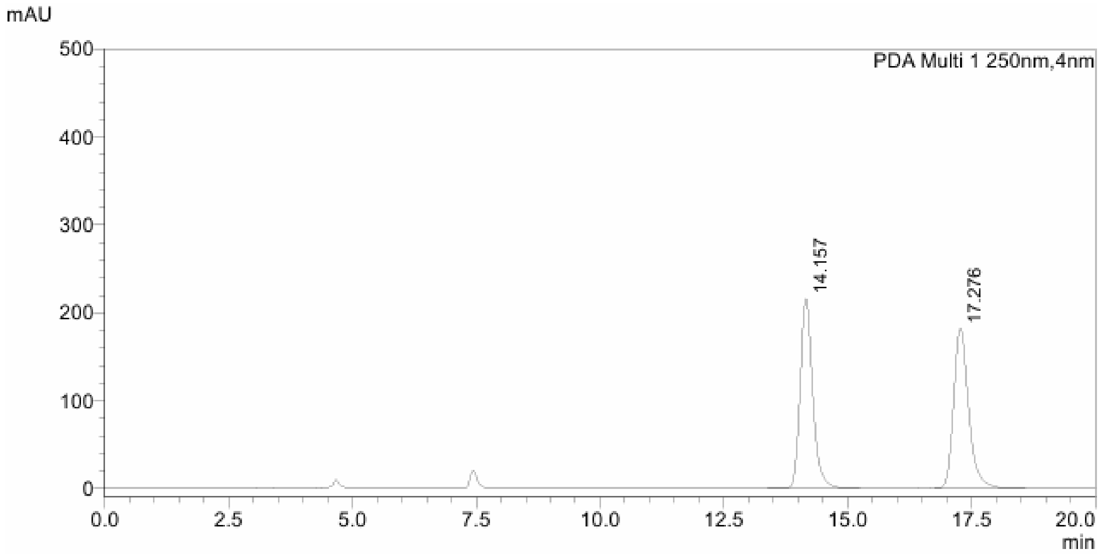

<Peak Table>

PDA Ch1 250nm

Peak#	Ret. Time	Area	Height	Area%	Height%
1	14.157	3805838	215651	50.026	54.296
2	17.276	3801921	181526	49.974	45.704
Total		7607758	397177	100.000	100.000

Figure S50. HPLC spectrum of racemic **4n**

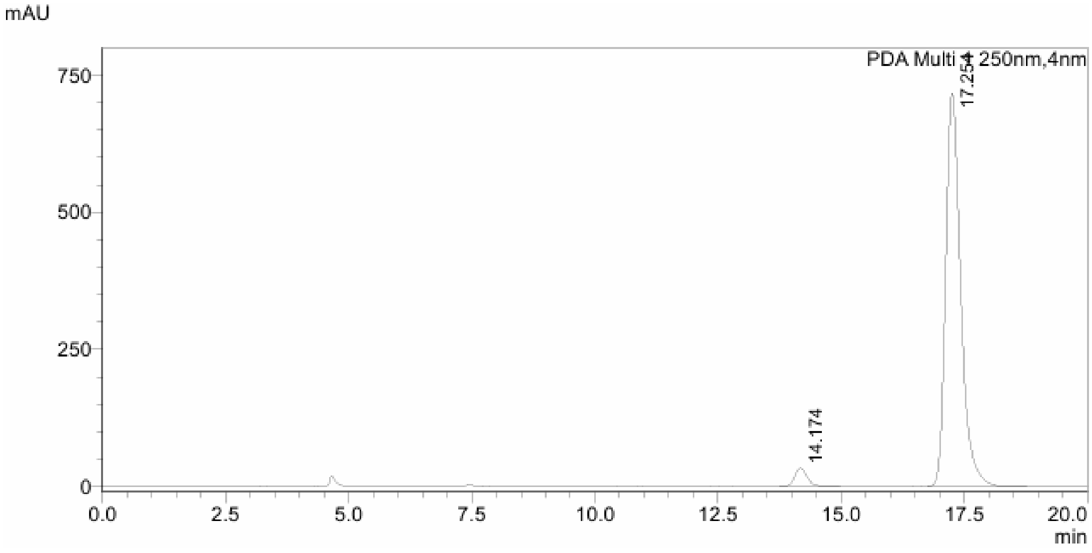

<Peak Table>

PDA Ch1 250nm

Peak#	Ret. Time	Area	Height	Area%	Height%
1	14.174	583603	33032	3.692	4.405
2	17.254	15225507	716869	96.308	95.595
Total		15809110	749901	100.000	100.000

Figure S51. HPLC spectrum of **4n**

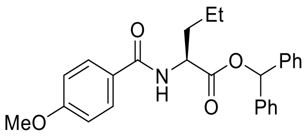

(4o)

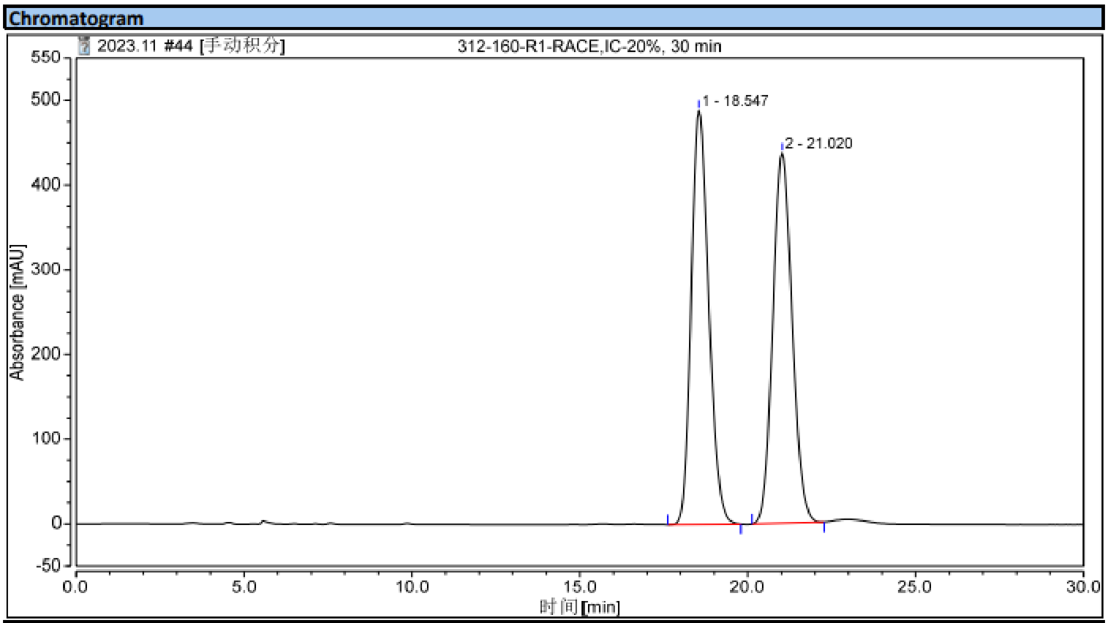

Integration Results

No.	Peak Name	Retention Time<br>min	Area<br>mAu*min	Height<br>mAU	Relative Area<br>%	Relative Height<br>%	Amount<br>n.a.
1		18.547	294.274	488.756	50.24	52.80	n.a.
2		21.020	291.487	436.983	49.76	47.20	n.a.
**Total:**			**585.760**	**925.739**	**100.00**	**100.00**	

Figure S52. HPLC spectrum of racemic **4o**

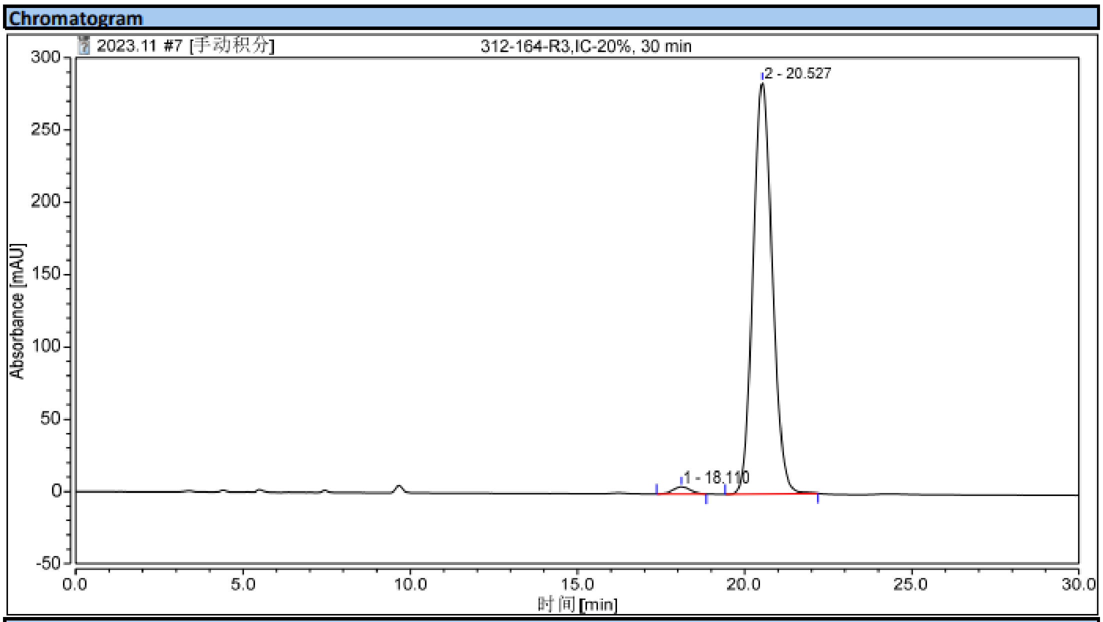

Integration Results

No.	Peak Name	Retention Time<br>min	Area<br>mAu*min	Height<br>mAU	Relative Area<br>%	Relative Height<br>%	Amount<br>n.a.
1		18.110	2.833	4.861	1.48	1.68	n.a.
2		20.527	189.181	284.500	98.52	98.32	n.a.
**Total:**			**192.014**	**289.361**	**100.00**	**100.00**	

Figure S53. HPLC spectrum of **4o**

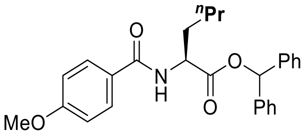

(4p)

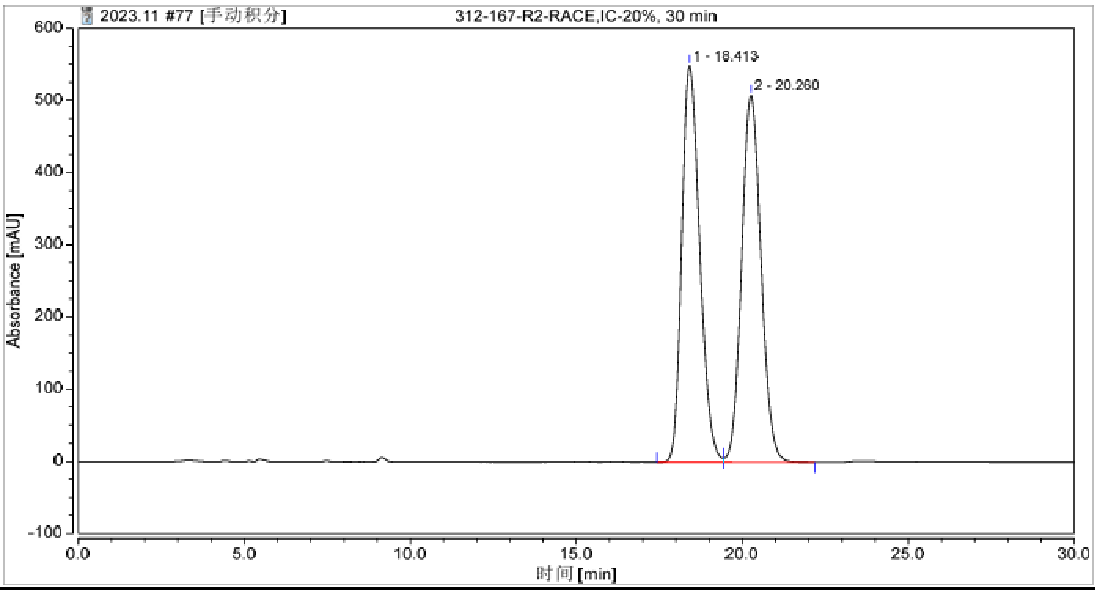

Integration Results							
No.	Peak Name	Retention Time min	Area mAu*min	Height mAU	Relative Area %	Relative Height %	Amount n.a.
1		18.413	343.759	550.235	49.94	51.94	n.a.
2		20.260	344.618	509.126	50.06	48.06	n.a.
**Total:**			**688.378**	**1059.361**	**100.00**	**100.00**	

Figure S54. HPLC spectrum of racemic **4p**

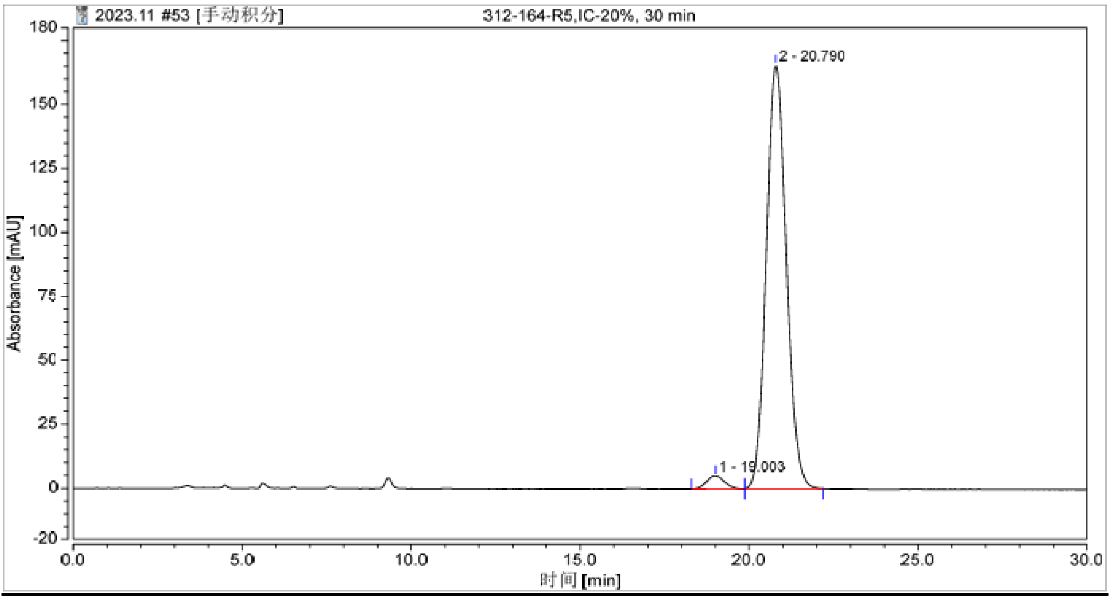

Integration Results							
No.	Peak Name	Retention Time min	Area mAu*min	Height mAU	Relative Area %	Relative Height %	Amount n.a.
1		19.003	3.062	5.058	2.62	2.96	n.a.
2		20.790	113.741	165.559	97.38	97.04	n.a.
**Total:**			**116.803**	**170.616**	**100.00**	**100.00**	

Figure S55. HPLC spectrum of **4p**

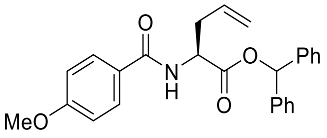

(4q)

Chromatogram

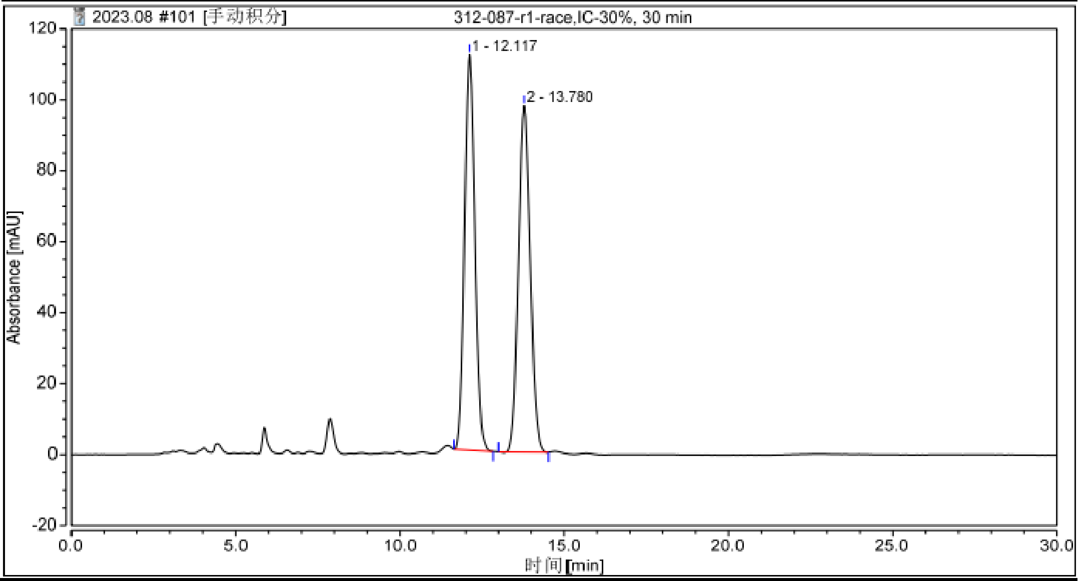

Integration Results

No.	Peak Name	Retention Time<br>min	Area<br>mAu*min	Height<br>mAU	Relative Area<br>%	Relative Height<br>%	Amount<br>n.a.
1		12.117	40.621	111.532	49.80	53.31	n.a.
2		13.780	40.941	97.693	50.20	46.69	n.a.
**Total:**			**81.562**	**209.225**	**100.00**	**100.00**	

Figure S56. HPLC spectrum of racemic **4q**

Chromatogram

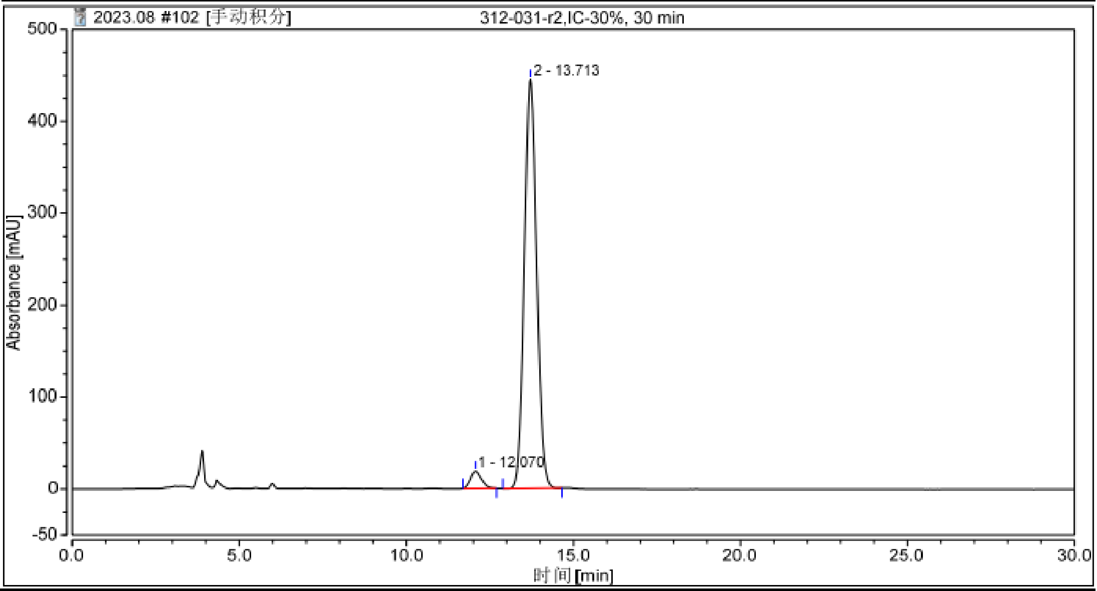

Integration Results

No.	Peak Name	Retention Time<br>min	Area<br>mAu*min	Height<br>mAU	Relative Area<br>%	Relative Height<br>%	Amount<br>n.a.
1		12.070	7.085	18.699	3.60	4.04	n.a.
2		13.713	189.846	444.483	96.40	95.96	n.a.
**Total:**			**196.931**	**463.182**	**100.00**	**100.00**	

Figure S57. HPLC spectrum of **4q**

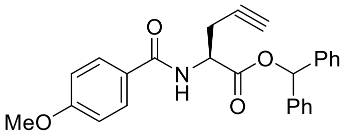

(4r)

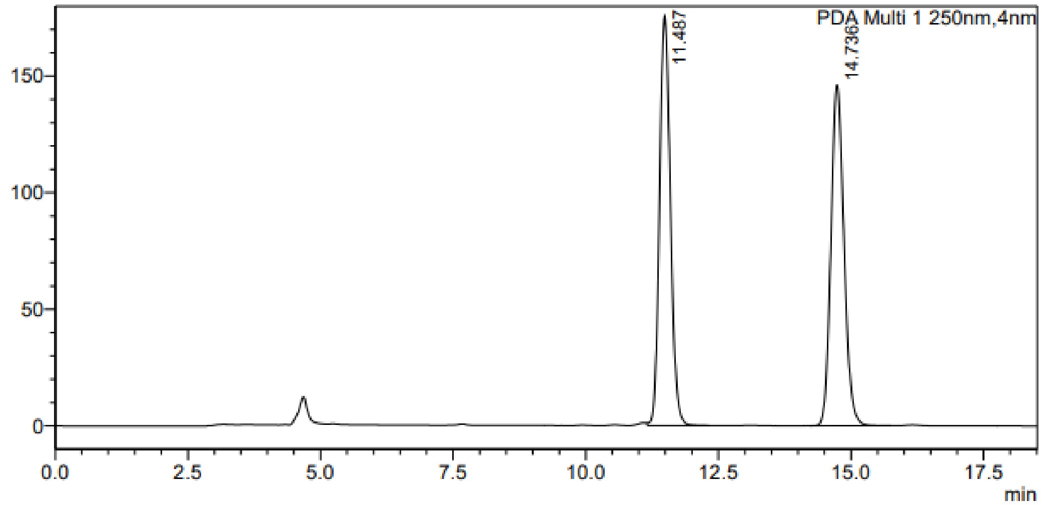

<Peak Table>

PDA Ch1 250nm

Peak#	Ret. Time	Area	Height	Area%	Height%
1	11.487	2514285	175302	50.061	54.555
2	14.736	2508148	146027	49.939	45.445
Total		5022433	321329	100.000	100.000

Figure S58. HPLC spectrum of racemic **4r**

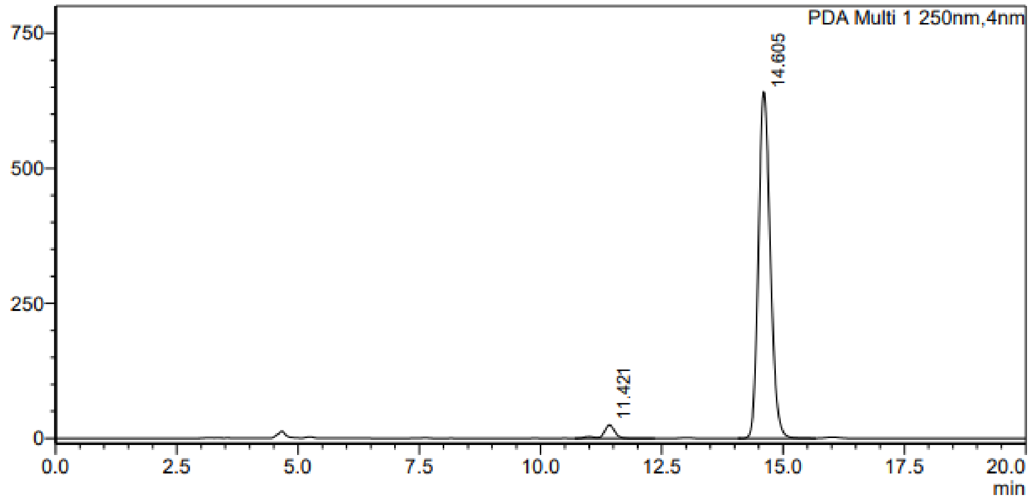

<Peak Table>

PDA Ch1 250nm

Peak#	Ret. Time	Area	Height	Area%	Height%
1	11.421	394414	24378	3.473	3.666
2	14.605	10961145	640506	96.527	96.334
Total		11355559	664884	100.000	100.000

Figure S59. HPLC spectrum of **4r**

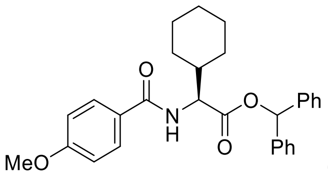

(4s)

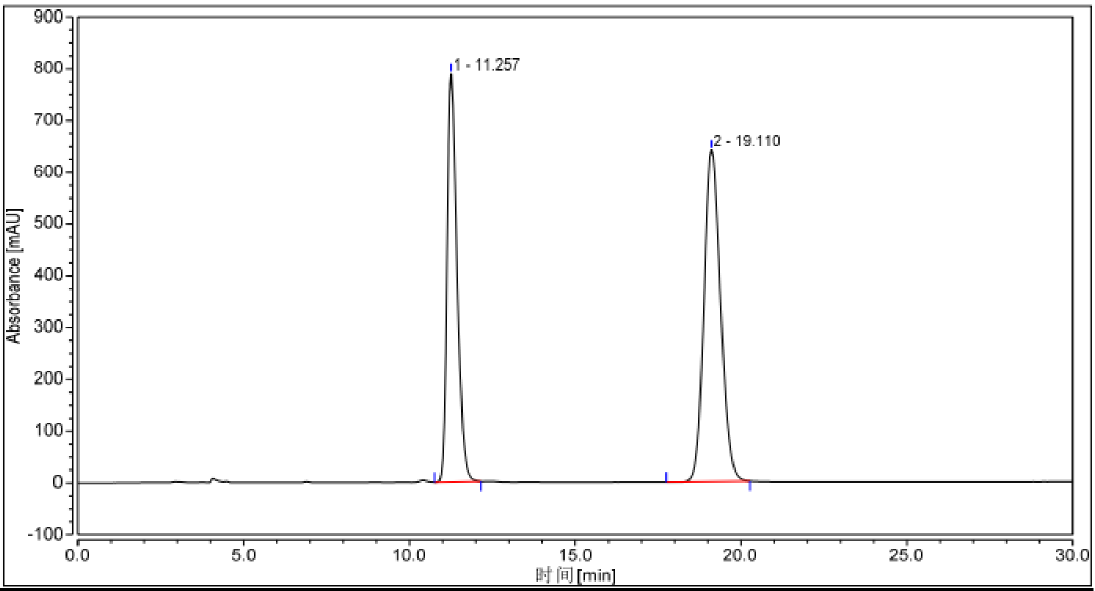

Integration Results							
No.	Peak Name	Retention Time min	Area mAu*min	Height mAU	Relative Area %	Relative Height %	Amount n.a.
1		11.257	271.158	788.407	42.21	55.20	n.a.
2		19.110	371.280	639.965	57.79	44.80	n.a.
Total:			642.438	1428.371	100.00	100.00	

Figure S60. HPLC spectrum of racemic **4s**

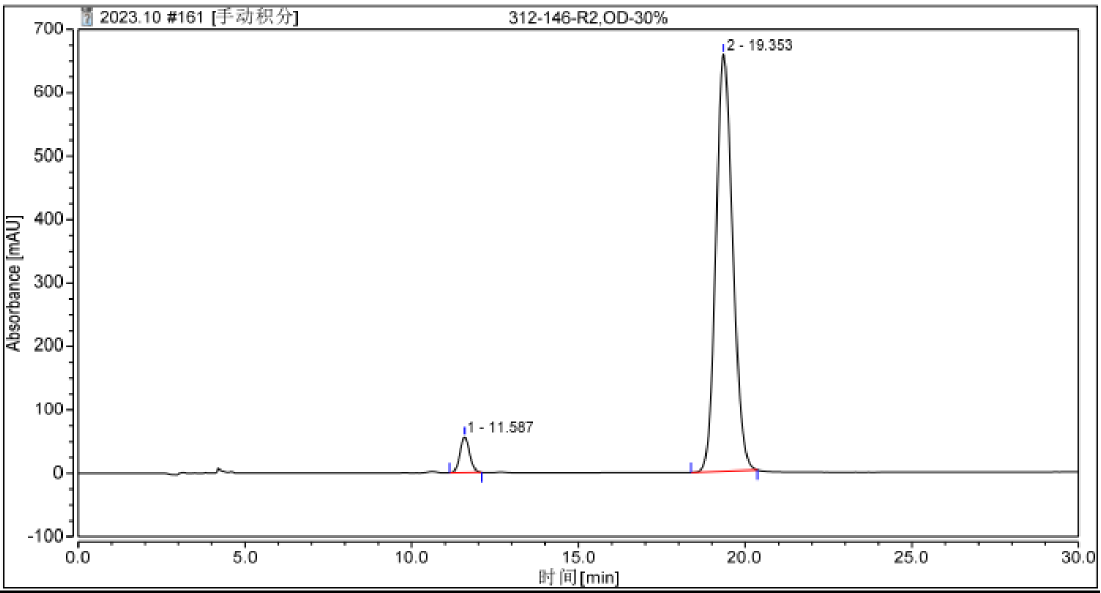

Integration Results							
No.	Peak Name	Retention Time min	Area mAu*min	Height mAU	Relative Area %	Relative Height %	Amount n.a.
1		11.587	18.592	55.859	4.62	7.83	n.a.
2		19.353	383.877	657.755	95.38	92.17	n.a.
Total:			402.468	713.614	100.00	100.00	

Figure S61. HPLC spectrum of **4s**

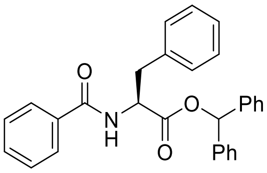

(**4aa**)

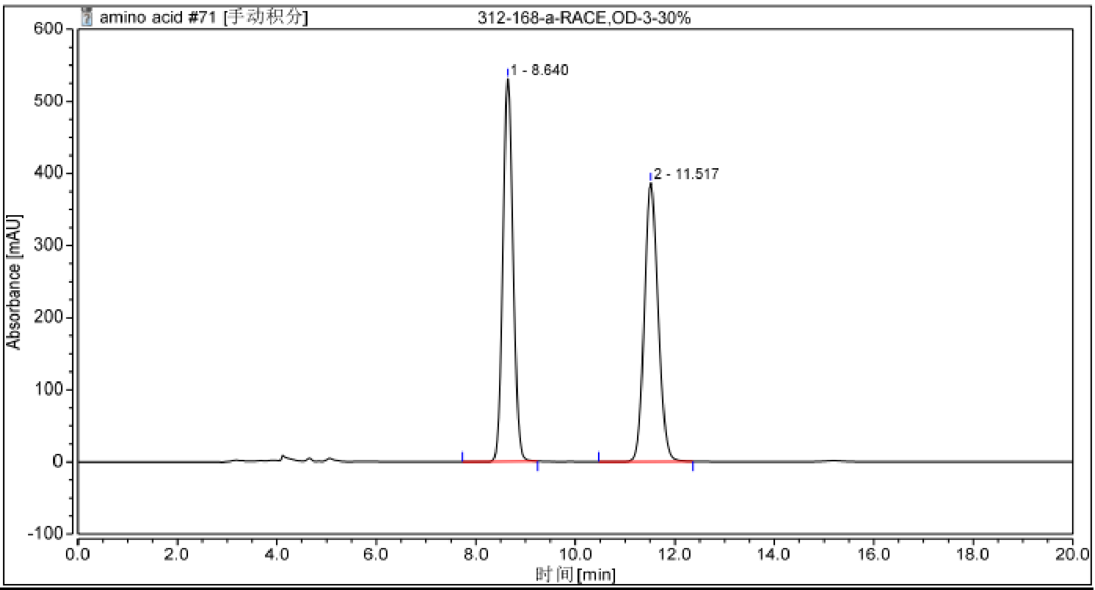

Integration Results							
No.	Peak Name	Retention Time min	Area mAu*min	Height mAU	Relative Area %	Relative Height %	Amount n.a.
1		8.640	123.969	530.974	49.94	57.86	n.a.
2		11.517	124.255	386.688	50.06	42.14	n.a.
**Total:**			**248.224**	**917.661**	**100.00**	**100.00**	

Figure S62. HPLC spectrum of racemic **4aa**

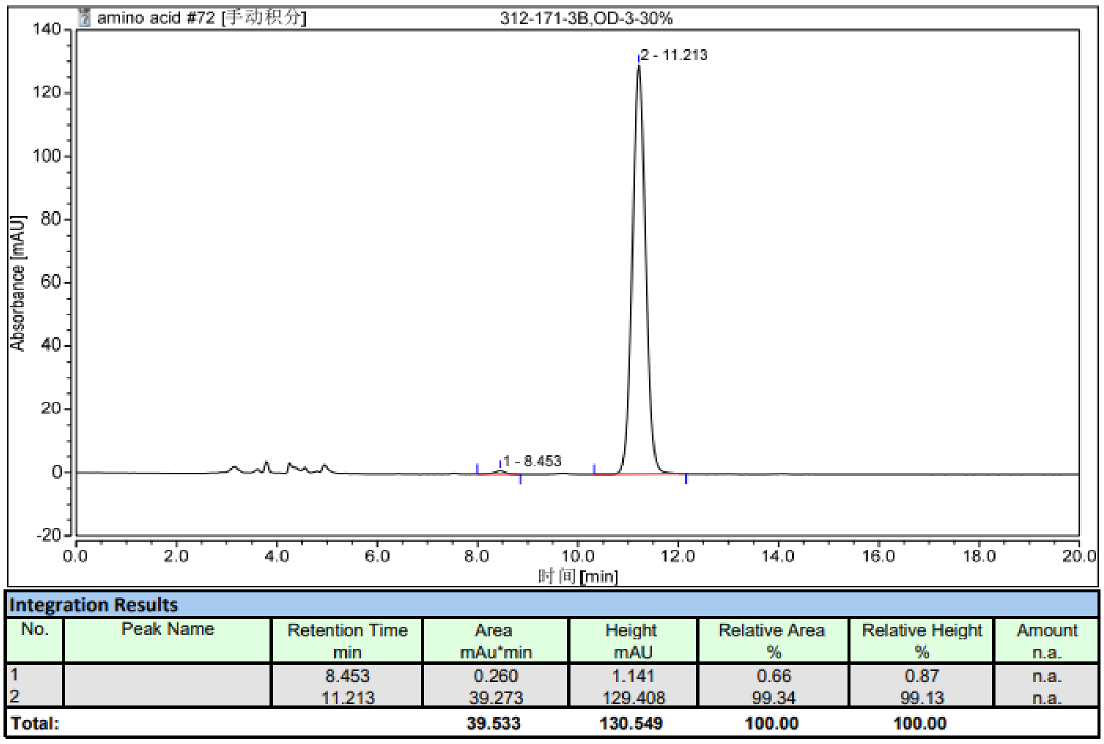

Integration Results							
No.	Peak Name	Retention Time min	Area mAu*min	Height mAU	Relative Area %	Relative Height %	Amount n.a.
1		8.453	0.260	1.141	0.66	0.87	n.a.
2		11.213	39.273	129.408	99.34	99.13	n.a.
**Total:**			**39.533**	**130.549**	**100.00**	**100.00**	

Figure S63. HPLC spectrum of **4aa**

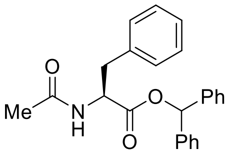

(*S*-**4ab**)

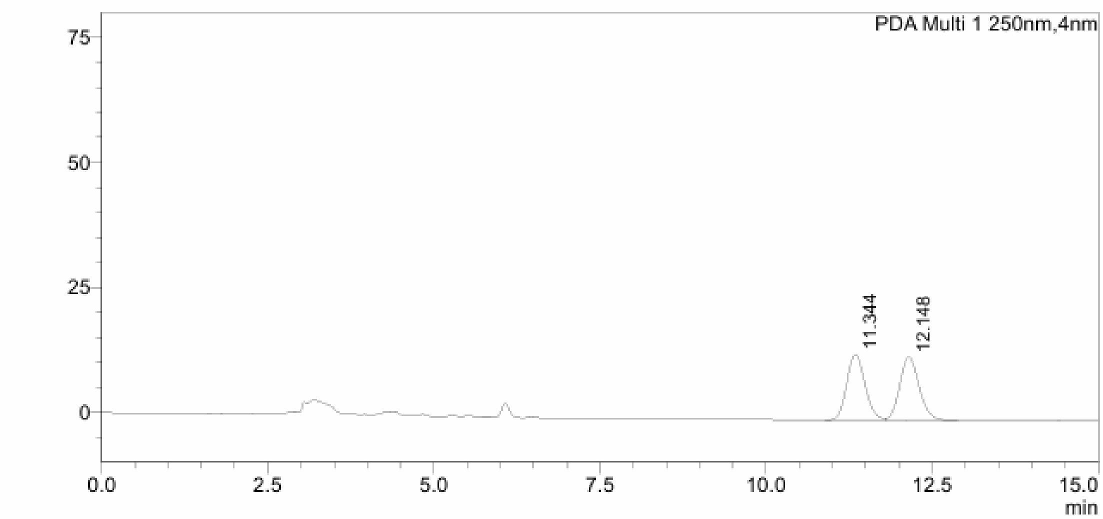

<Peak Table>

PDA Ch1 250nm

Peak#	Ret. Time	Area	Height	Area%	Height%
1	11.344	249062	13070	49.443	50.911
2	12.148	254668	12603	50.557	49.089
Total		503730	25673	100.000	100.000

Figure S64. HPLC spectrum of racemic **4ab**

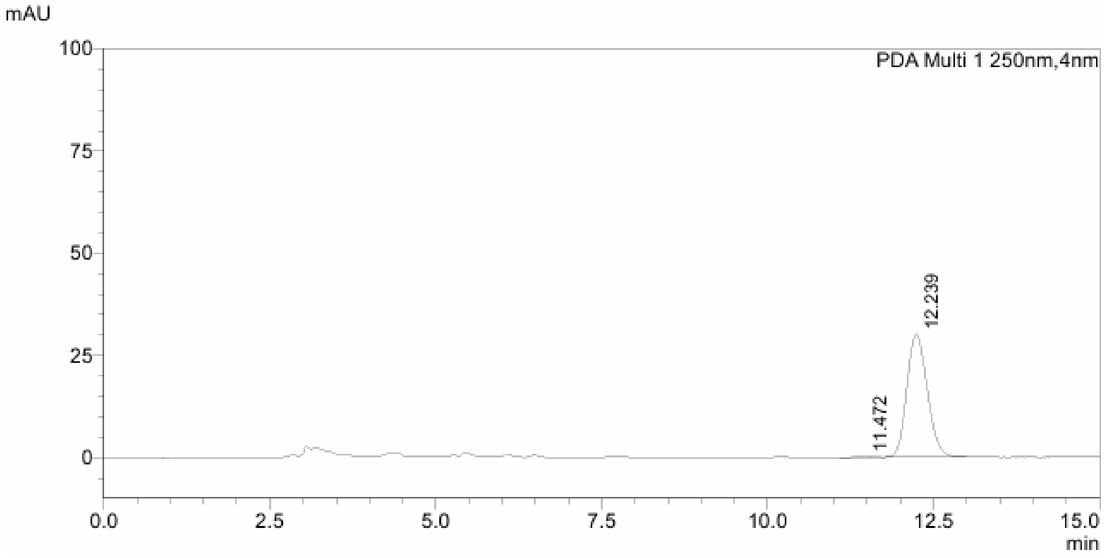

<Peak Table>

PDA Ch1 250nm

Peak#	Ret. Time	Area	Height	Area%	Height%
1	11.472	1534	93	0.239	0.308
2	12.239	639929	29986	99.761	99.692
Total		641463	30079	100.000	100.000

Figure S65. HPLC spectrum of (*S*)-**4ab**

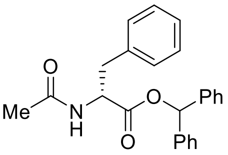

(*R*-**4ab**)

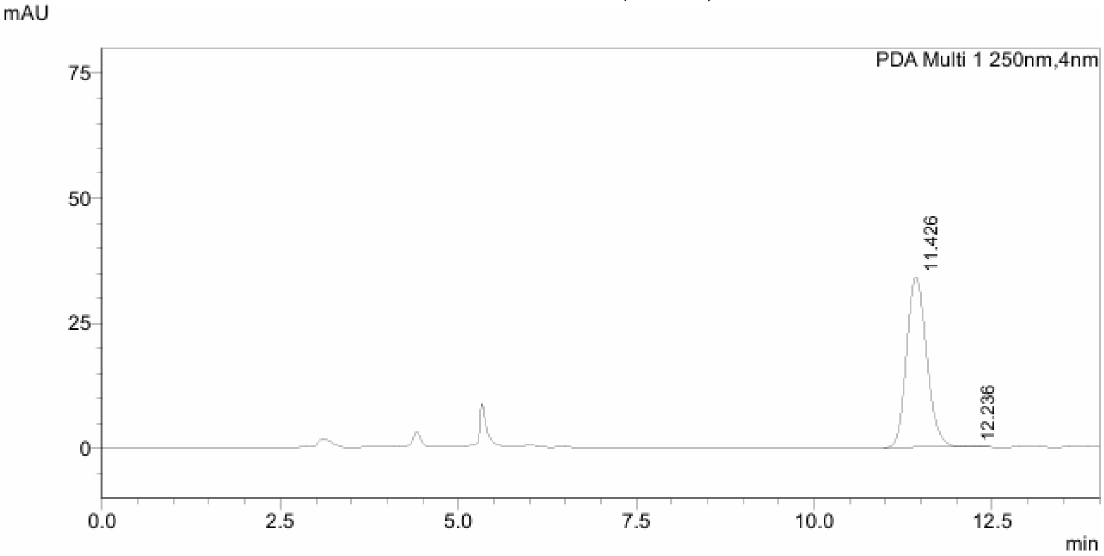

<Peak Table>

PDA Ch1 250nm

Peak#	Ret. Time	Area	Height	Area%	Height%
1	11.426	662332	34018	99.715	99.582
2	12.236	1890	143	0.285	0.418
Total		664222	34161	100.000	100.000

Figure S66. HPLC spectrum of (*R*)-**4ab**

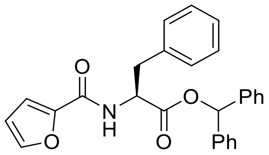

(4ac)

Chromatogram

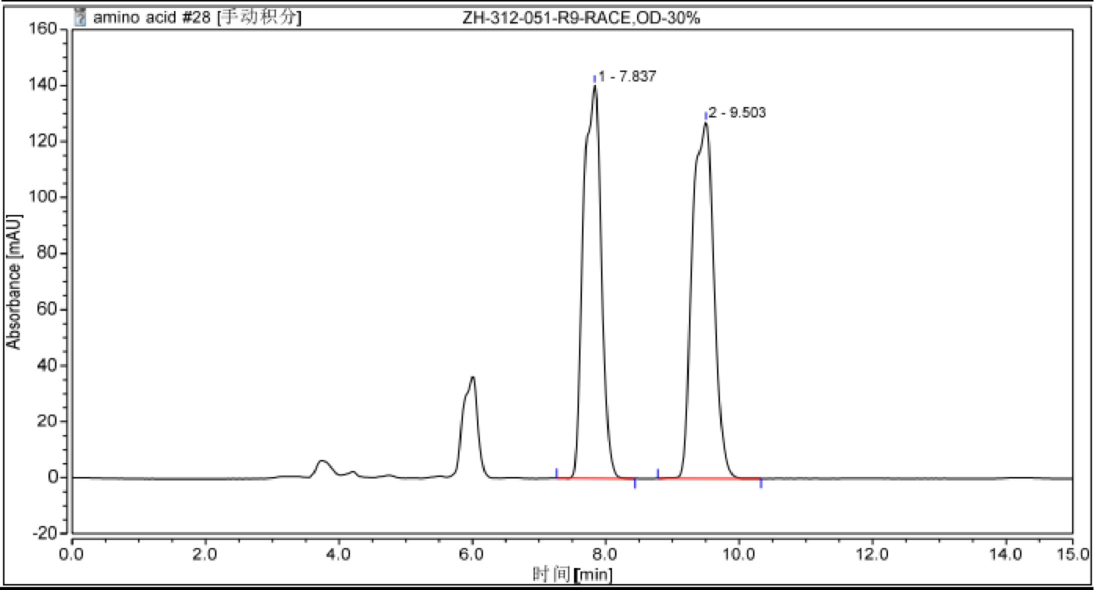

Integration Results

No.	Peak Name	Retention Time min	Area mAu*min	Height mAU	Relative Area %	Relative Height %	Amount n.a.
1		7.837	43.382	140.348	47.63	52.46	n.a.
2		9.503	47.703	127.196	52.37	47.54	n.a.
**Total:**			**91.084**	**267.545**	**100.00**	**100.00**	

Figure S67. HPLC spectrum of racemic **4ac**

Chromatogram

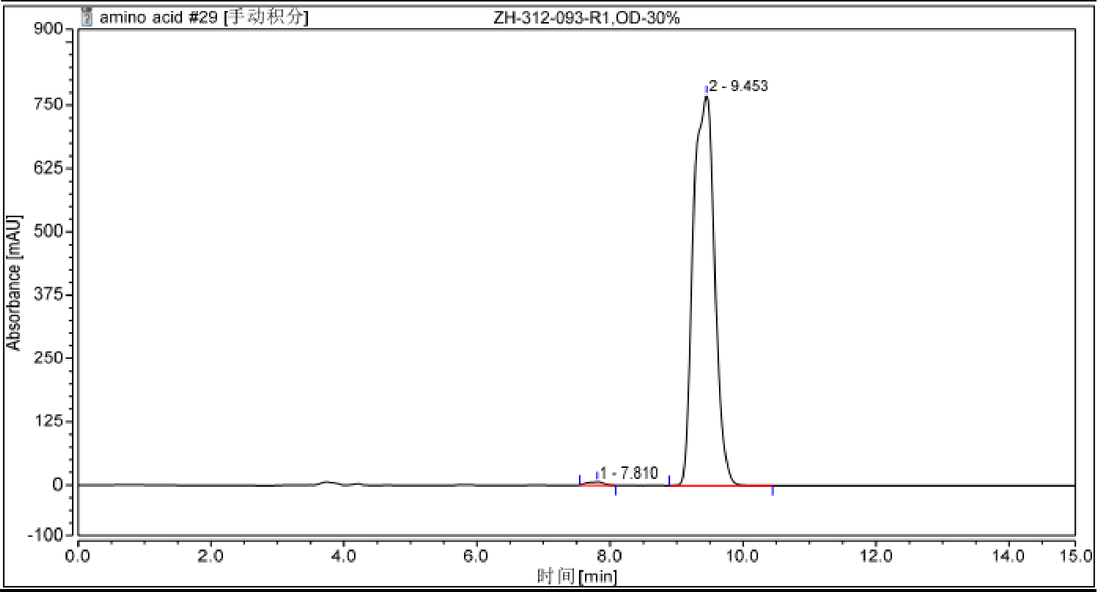

Integration Results

No.	Peak Name	Retention Time min	Area mAu*min	Height mAU	Relative Area %	Relative Height %	Amount n.a.
1		7.810	1.883	6.595	0.65	0.85	n.a.
2		9.453	285.757	770.042	99.35	99.15	n.a.
**Total:**			**287.640**	**776.637**	**100.00**	**100.00**	

Figure S68. HPLC spectrum of **4ac**

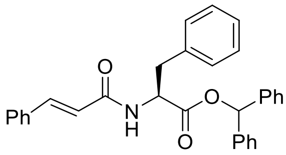

(4ad)

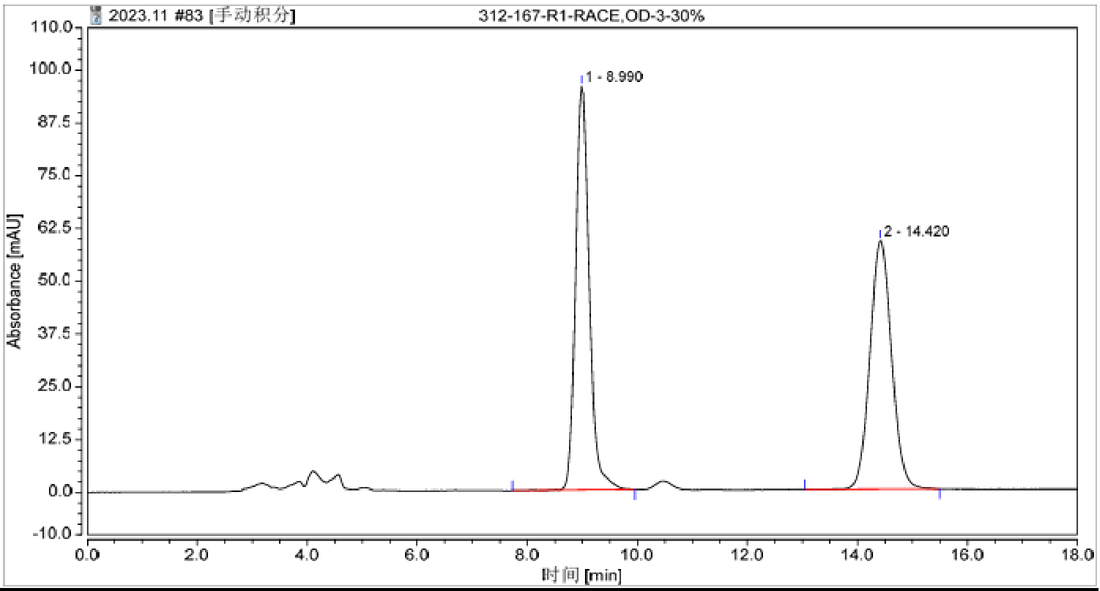

Integration Results							
No.	Peak Name	Retention Time min	Area mAu*min	Height mAU	Relative Area %	Relative Height %	Amount n.a.
1		8.990	27.573	95.711	50.68	61.91	n.a.
2		14.420	26.838	58.892	49.32	38.09	n.a.
**Total:**			**54.410**	**154.603**	**100.00**	**100.00**	

Figure S69. HPLC spectrum of racemic **4ad**

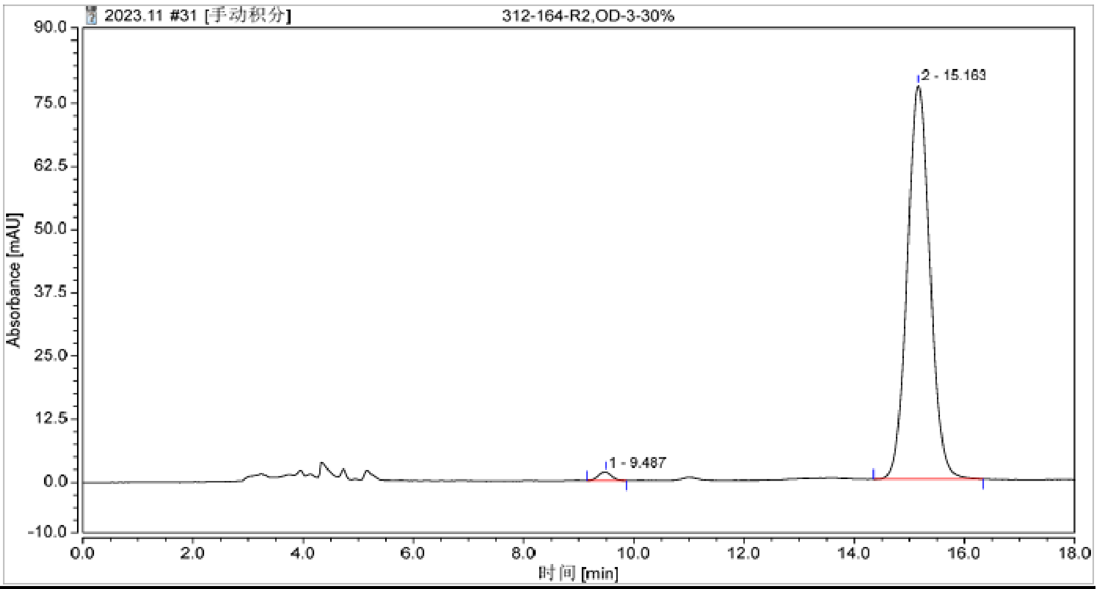

Integration Results							
No.	Peak Name	Retention Time min	Area mAu*min	Height mAU	Relative Area %	Relative Height %	Amount n.a.
1		9.487	0.466	1.720	1.24	2.16	n.a.
2		15.163	37.165	78.046	98.76	97.84	n.a.
**Total:**			**37.631**	**79.765**	**100.00**	**100.00**	

Figure S70. HPLC spectrum of **4ad**

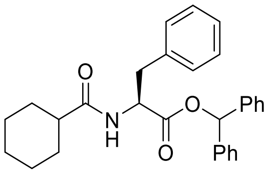

(**4ae**)

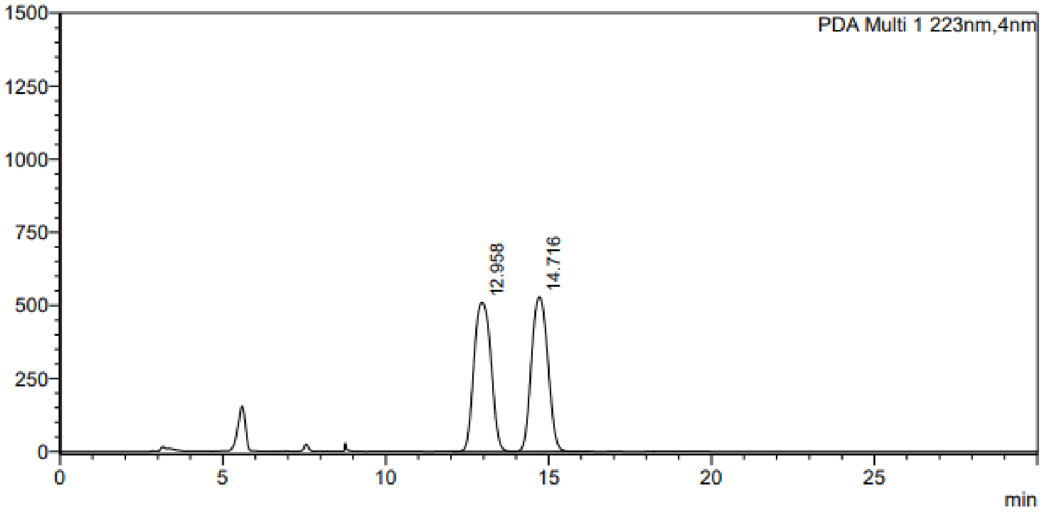

<Peak Table>

PDA Ch1 223nm

Peak#	Ret. Time	Area	Height	Area%	Height%
1	12.958	18594433	509537	50.064	49.080
2	14.716	18546553	528639	49.936	50.920
Total		37140985	1038176	100.000	100.000

Figure S71. HPLC spectrum of racemic **4ae**

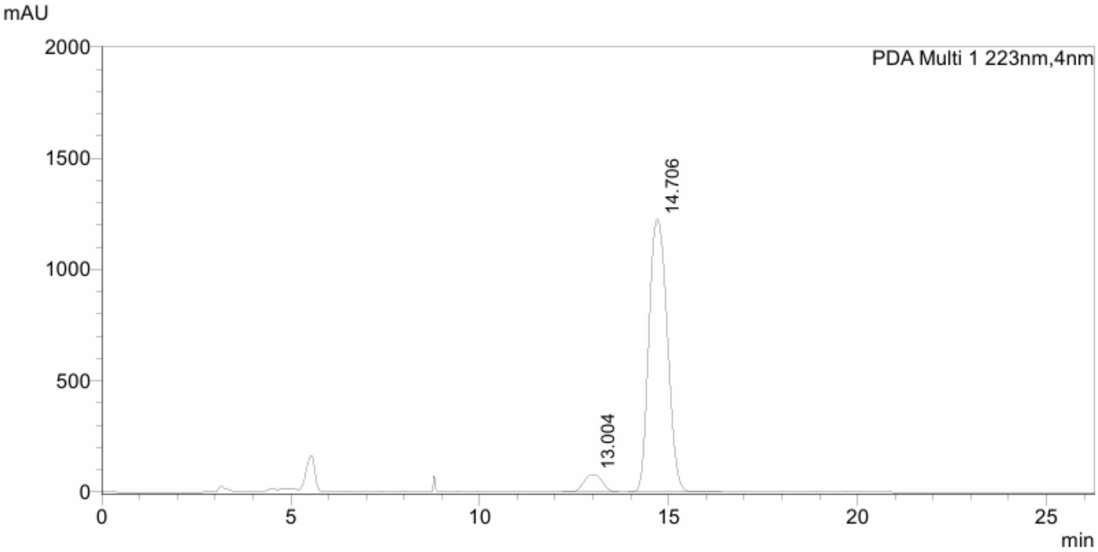

<Peak Table>

PDA Ch1 223nm

Peak#	Ret. Time	Area	Height	Area%	Height%
1	13.004	2584495	77510	5.880	5.949
2	14.706	41371688	1225330	94.120	94.051
Total		43956183	1302839	100.000	100.000

Figure S72. HPLC spectrum of **4ae**

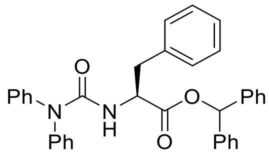

(4af)

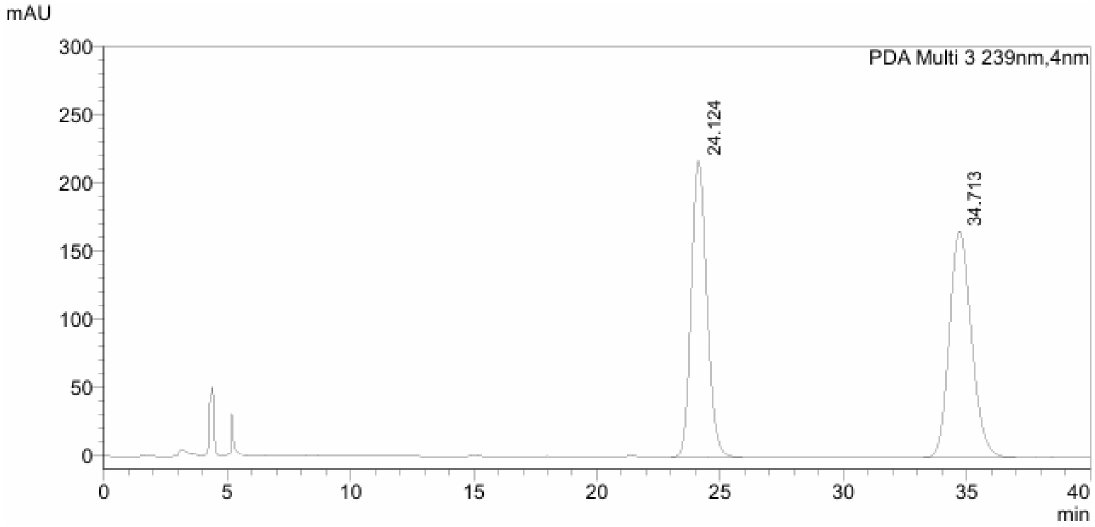

<Peak Table>

PDA Ch3 239nm

Peak#	Ret. Time	Area	Height	Area%	Height%
1	24.124	9512347	216905	47.968	56.724
2	34.713	10318358	165482	52.032	43.276
Total		19830705	382388	100.000	100.000

Figure S73. HPLC spectrum of racemic **4af**

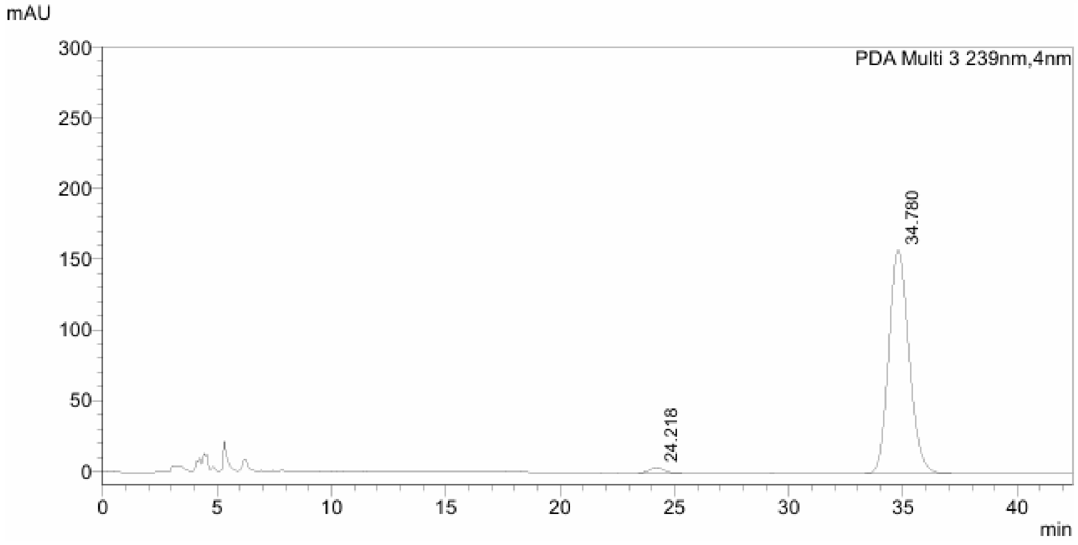

<Peak Table>

PDA Ch3 239nm

Peak#	Ret. Time	Area	Height	Area%	Height%
1	24.218	160057	3802	1.619	2.354
2	34.780	9727921	157725	98.381	97.646
Total		9887978	161528	100.000	100.000

Figure S74. HPLC spectrum of **4af**

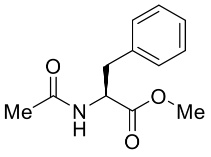

(4ag)

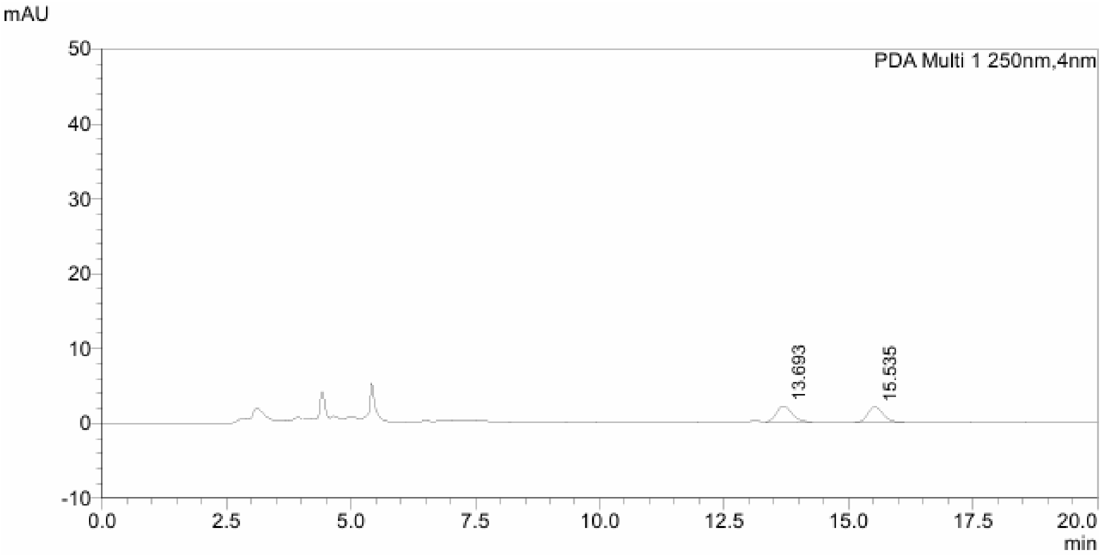

<Peak Table>

PDA Ch1 250nm

Peak#	Ret. Time	Area	Height	Area%	Height%
1	13.693	45240	2121	51.015	51.185
2	15.535	43440	2022	48.985	48.815
Total		88680	4143	100.000	100.000

Figure S75. HPLC spectrum of racemic **4ag**

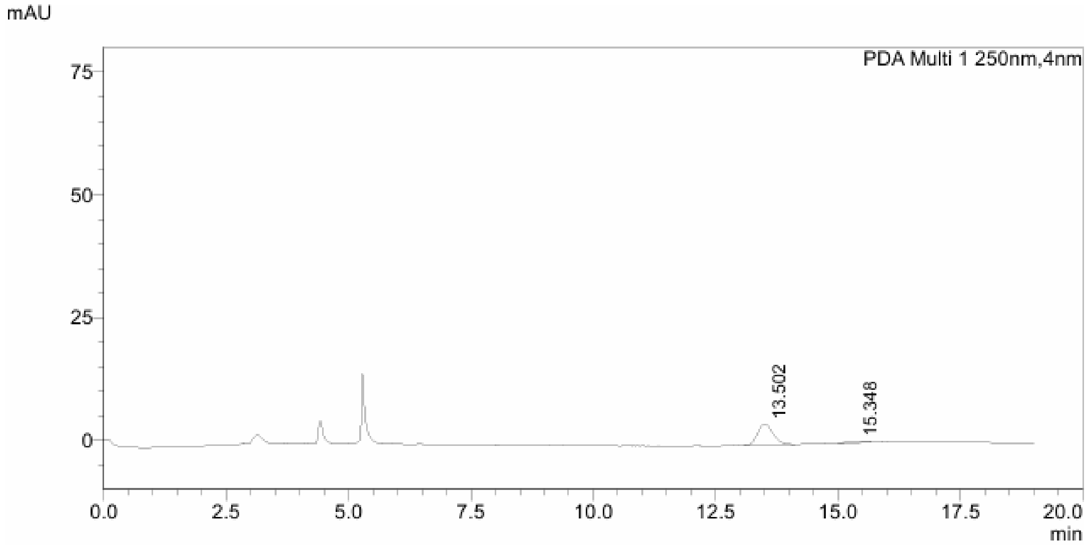

<Peak Table>

PDA Ch1 250nm

Peak#	Ret. Time	Area	Height	Area%	Height%
1	13.502	92454	4258	94.159	93.810
2	15.348	5735	281	5.841	6.190
Total		98190	4539	100.000	100.000

Figure S76. HPLC spectrum of **4ag**

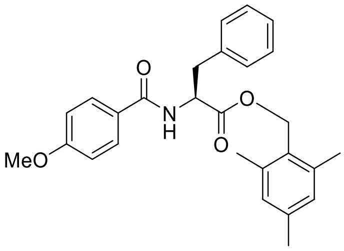

(4ah)

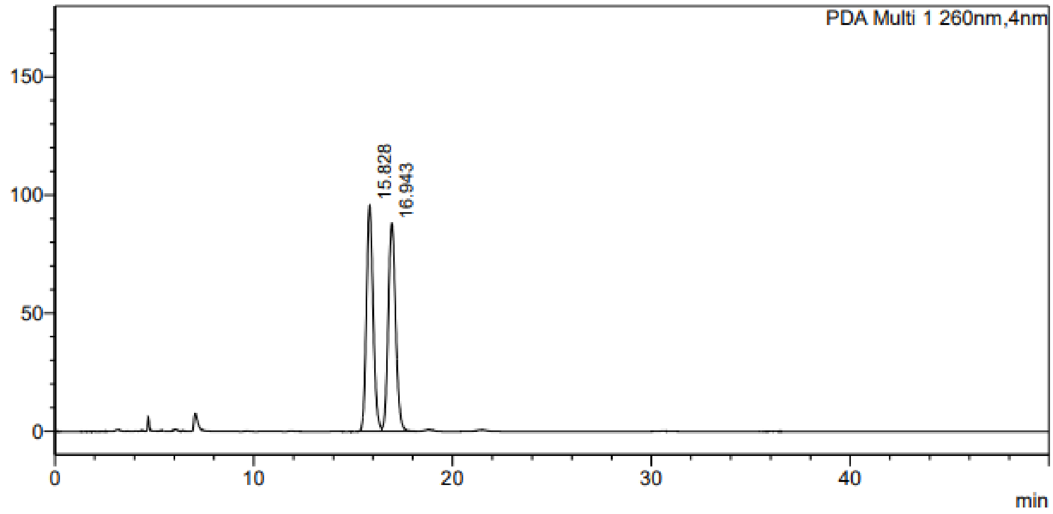

<Peak Table>

PDA Ch1 260nm

Peak#	Ret. Time	Area	Height	Area%	Height%
1	15.828	2274877	95699	49.933	52.111
2	16.943	2280993	87947	50.067	47.889
Total		4555870	183646	100.000	100.000

Figure S77. HPLC spectrum of racemic **4ah**

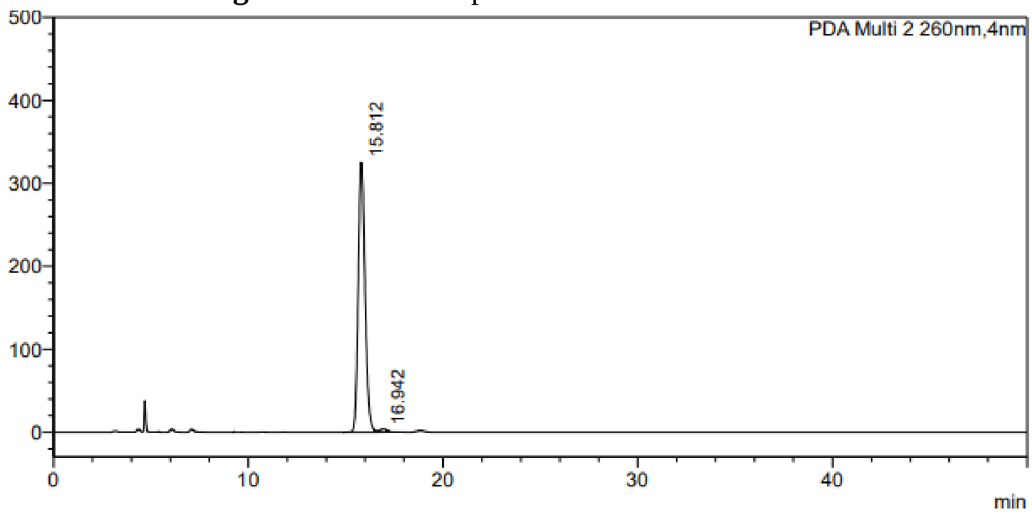

<Peak Table>

PDA Ch2 260nm

Peak#	Ret. Time	Area	Height	Area%	Height%
1	15.812	7815106	325802	98.712	98.853
2	16.942	101959	3780	1.288	1.147
Total		7917065	329582	100.000	100.000

Figure S78. HPLC spectrum of **4ah**

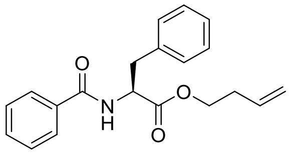

(**4ai**)

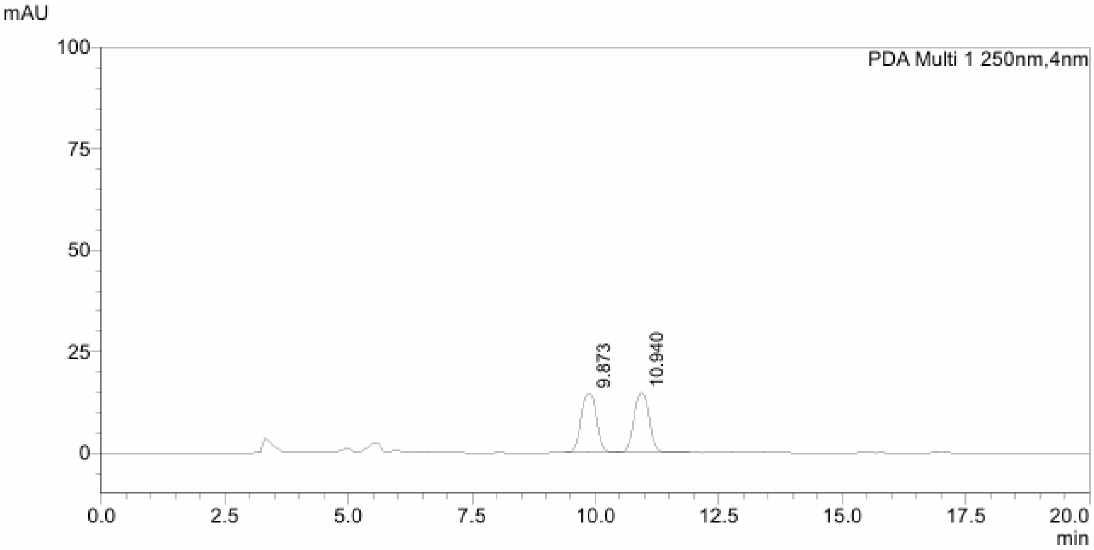

<Peak Table>

PDA Ch1 250nm

Peak#	Ret. Time	Area	Height	Area%	Height%
1	9.873	312976	14539	49.901	49.666
2	10.940	314218	14735	50.099	50.334
Total		627193	29275	100.000	100.000

Figure S79. HPLC spectrum of racemic **4ai**

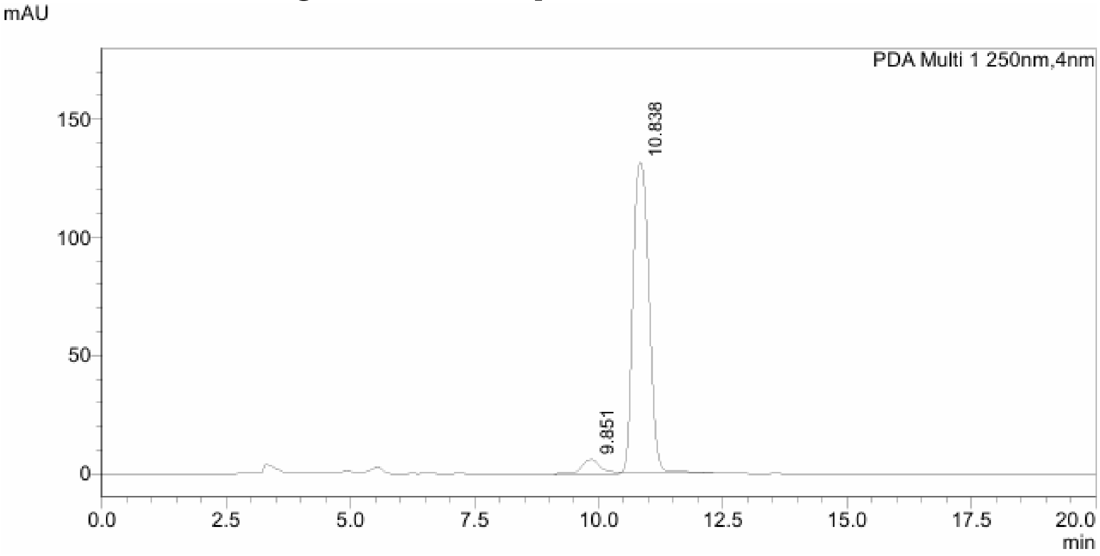

<Peak Table>

PDA Ch1 250nm

Peak#	Ret. Time	Area	Height	Area%	Height%
1	9.851	153944	5859	4.960	4.271
2	10.838	2949841	131324	95.040	95.729
Total		3103785	137183	100.000	100.000

Figure S80. HPLC spectrum of **4ai**

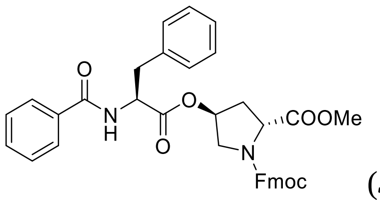

(*S*-**4aj**)

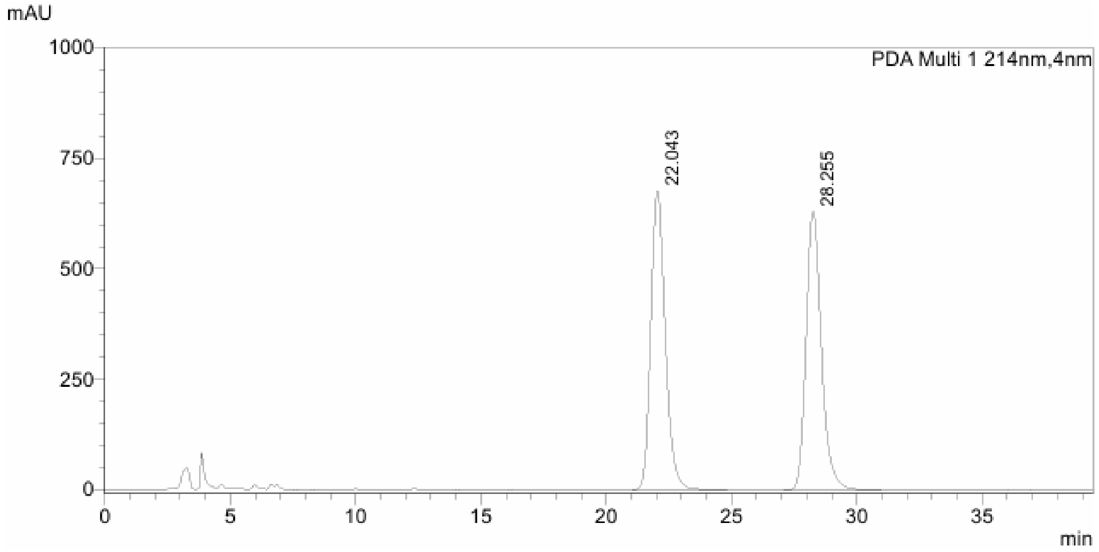

<Peak Table>

PDA Ch1 214nm

Peak#	Ret. Time	Area	Height	Area%	Height%
1	22.043	27176166	676689	50.350	51.809
2	28.255	26798611	629440	49.650	48.191
Total		53974777	1306128	100.000	100.000

Figure S81. HPLC spectrum of racemic **4aj**

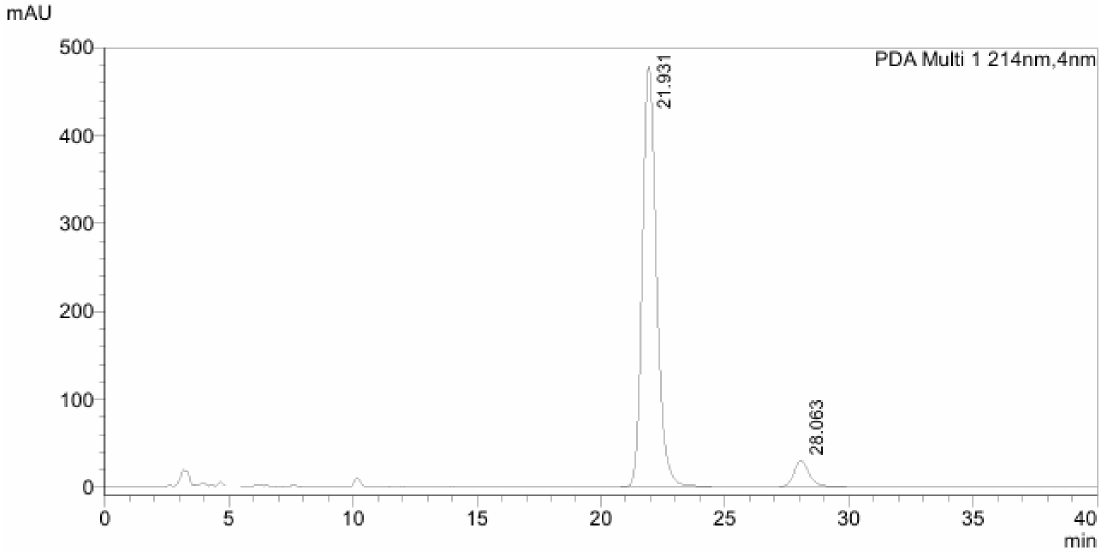

<Peak Table>

PDA Ch1 214nm

Peak#	Ret. Time	Area	Height	Area%	Height%
1	21.931	18874387	477423	93.992	94.205
2	28.063	1206463	29367	6.008	5.795
Total		20080850	506790	100.000	100.000

Figure S82. HPLC spectrum of (*S*)-**4aj**

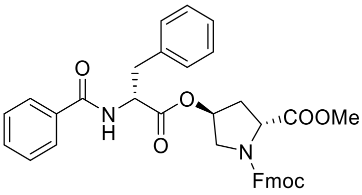

(*R*-**4aj**)

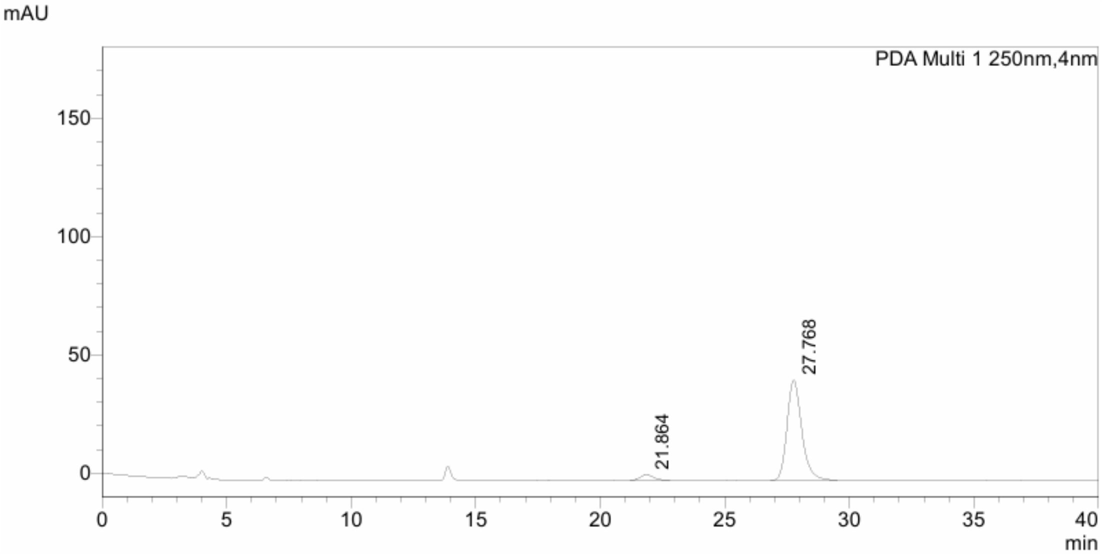

<Peak Table>

PDA Ch1 250nm

Peak#	Ret. Time	Area	Height	Area%	Height%
1	21.864	88727	2350	4.864	5.270
2	27.768	1735366	42230	95.136	94.730
Total		1824093	44580	100.000	100.000

Figure S83. HPLC spectrum of (*R*)-**4aj**

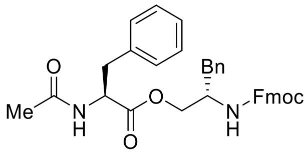

(4ak)

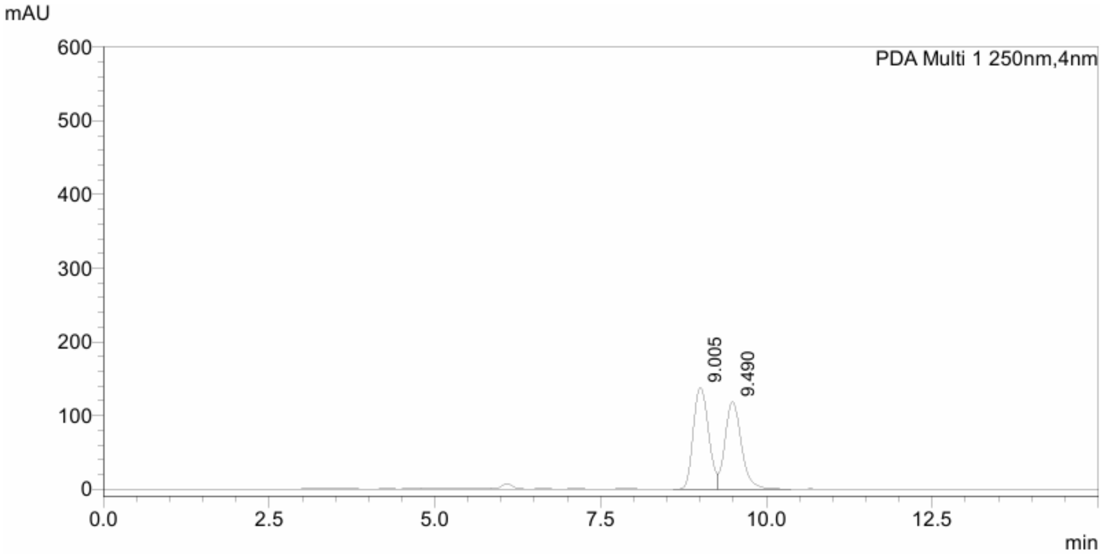

<Peak Table>

PDA Ch1 250nm

Peak#	Ret. Time	Area	Height	Area%	Height%
1	9.005	2206252	137605	52.164	53.780
2	9.490	2023233	118260	47.836	46.220
Total		4229485	255865	100.000	100.000

Figure S84. HPLC spectrum of racemic **4ak**

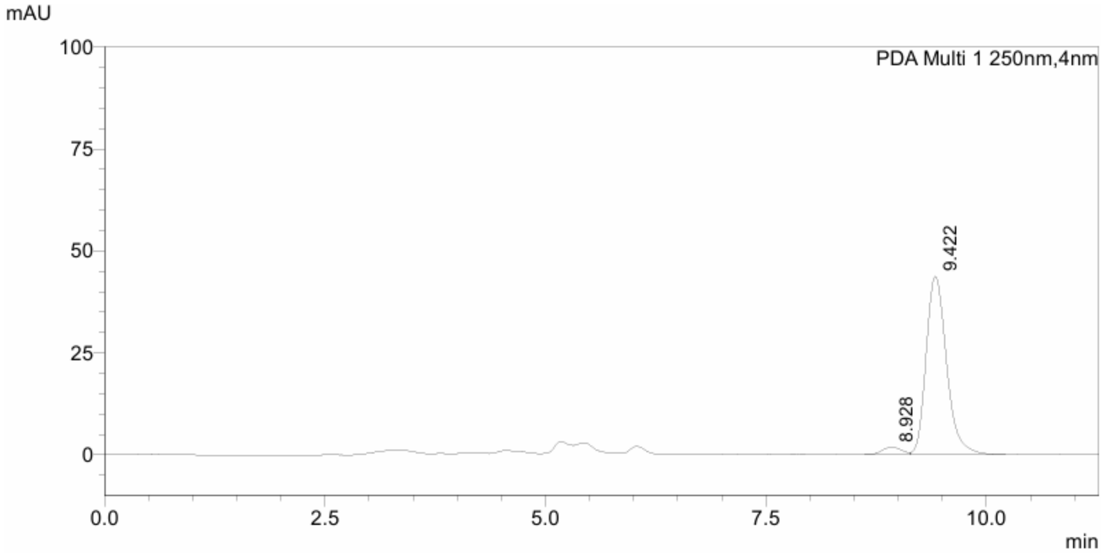

<Peak Table>

PDA Ch1 250nm

Peak#	Ret. Time	Area	Height	Area%	Height%
1	8.928	27036	1778	3.706	3.913
2	9.422	702497	43671	96.294	96.087
Total		729533	45449	100.000	100.000

Figure S85. HPLC spectrum of **4ak**

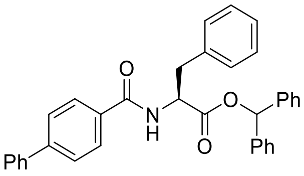

(**4al**)

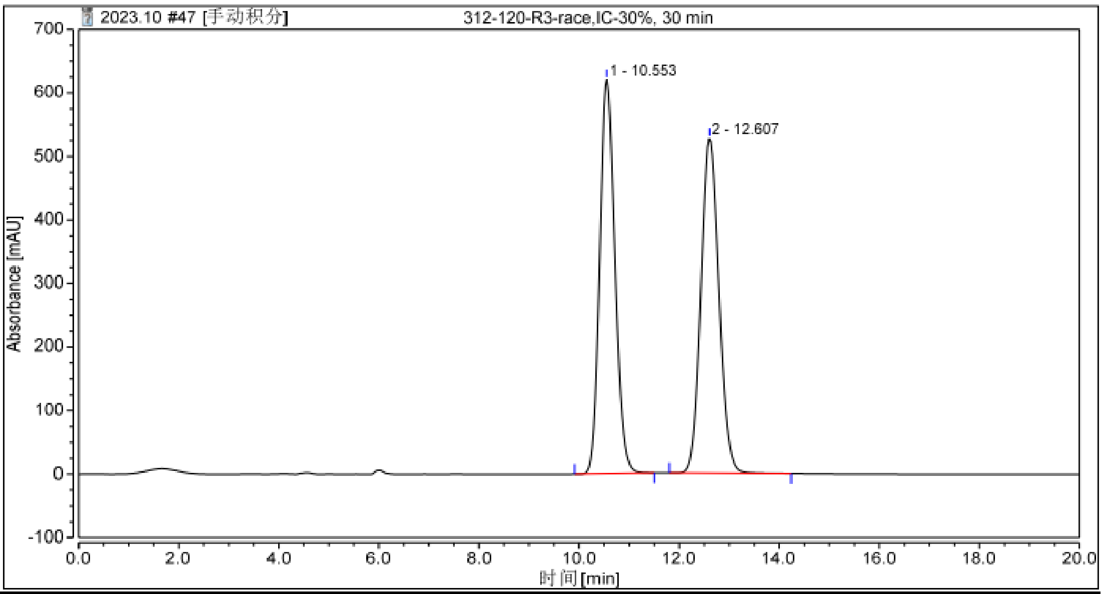

Integration Results							
No.	Peak Name	Retention Time min	Area mAu*min	Height mAU	Relative Area %	Relative Height %	Amount n.a.
1		10.553	219.183	620.338	49.86	54.08	n.a.
2		12.607	220.391	526.829	50.14	45.92	n.a.
Total:			439.575	1147.167	100.00	100.00	

Figure S86. HPLC spectrum of racemic **4al**

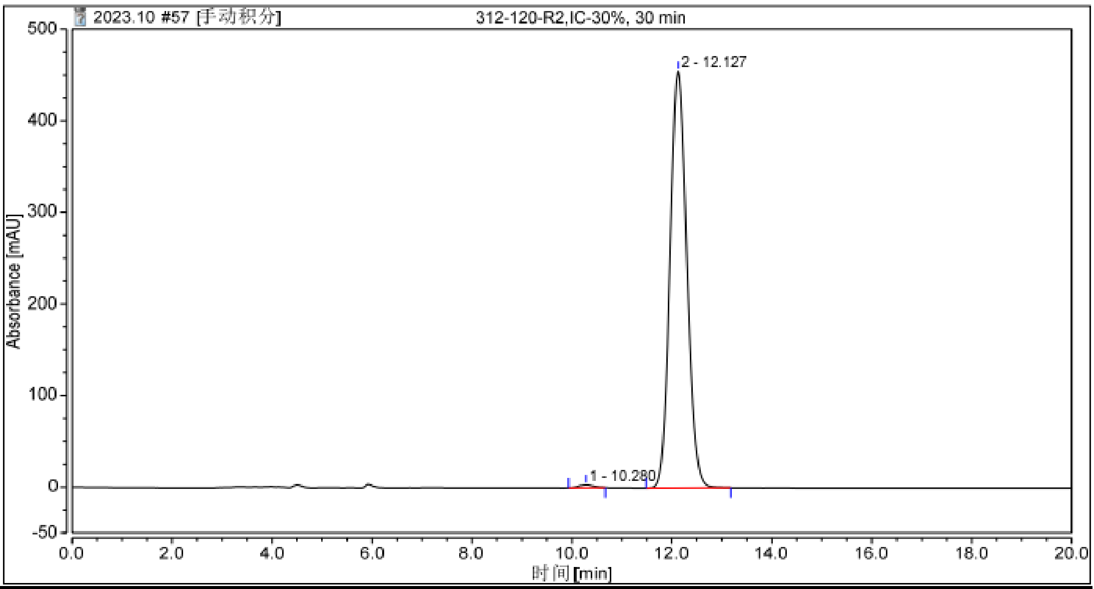

Integration Results							
No.	Peak Name	Retention Time min	Area mAu*min	Height mAU	Relative Area %	Relative Height %	Amount n.a.
1		10.280	1.074	3.285	0.60	0.72	n.a.
2		12.127	177.019	454.920	99.40	99.28	n.a.
Total:			178.093	458.205	100.00	100.00	

Figure S87. HPLC spectrum of **4al**

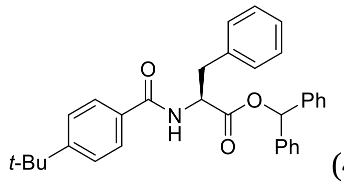

(4am)

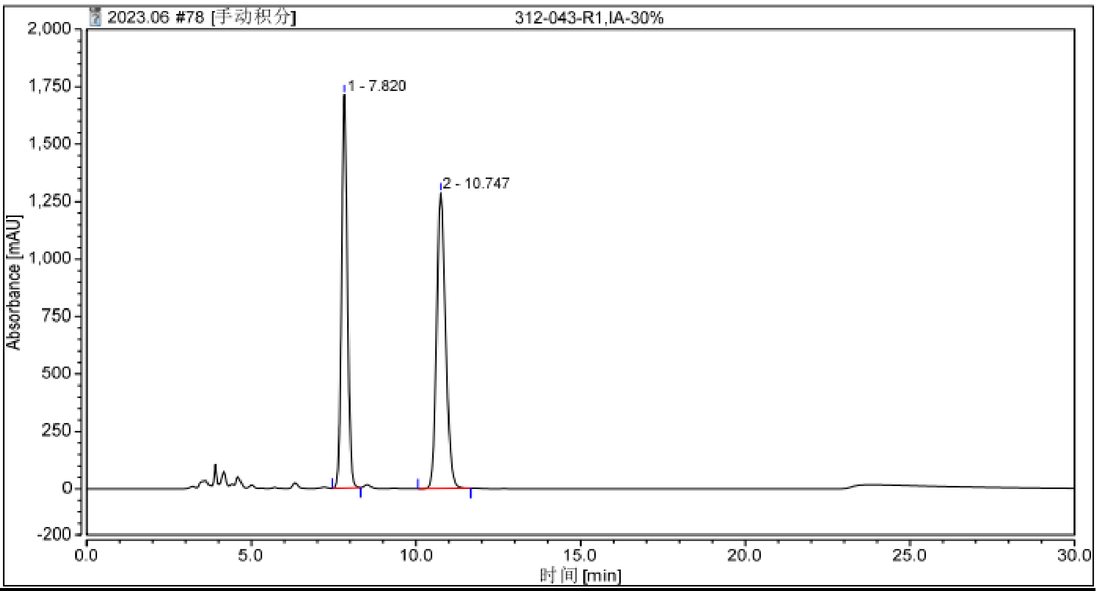

Integration Results							
No.	Peak Name	Retention Time min	Area mAu*min	Height mAU	Relative Area %	Relative Height %	Amount n.a.
1		7.820	359.824	1711.223	46.86	57.08	n.a.
2		10.747	408.057	1286.730	53.14	42.92	n.a.
**Total:**			**767.881**	**2997.953**	**100.00**	**100.00**	

Figure S88. HPLC spectrum of racemic **4am**

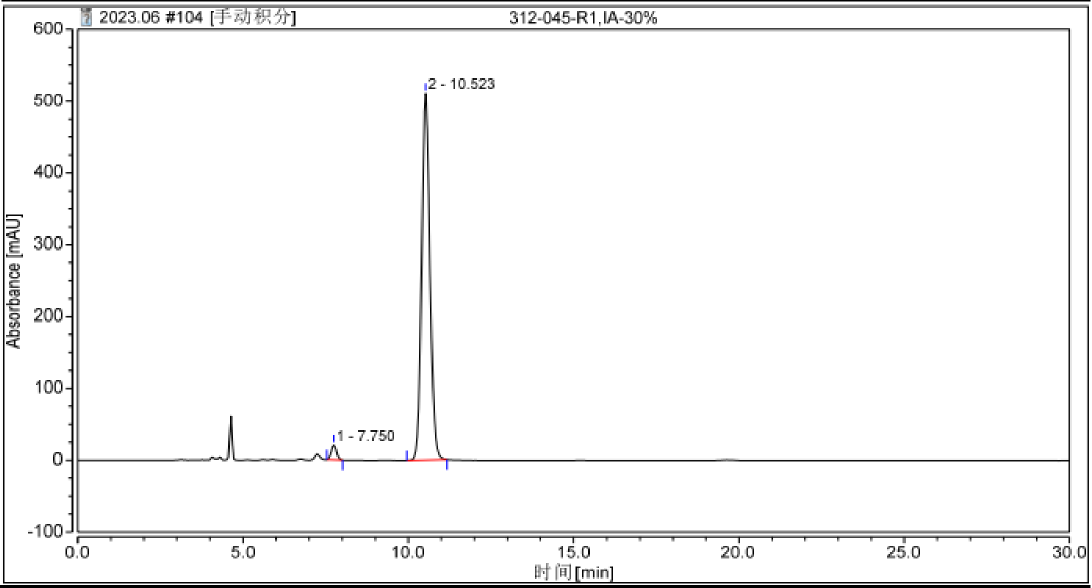

Integration Results							
No.	Peak Name	Retention Time min	Area mAu*min	Height mAU	Relative Area %	Relative Height %	Amount n.a.
1		7.750	3.916	20.856	2.59	3.93	n.a.
2		10.523	147.374	510.427	97.41	96.07	n.a.
**Total:**			**151.290**	**531.282**	**100.00**	**100.00**	

Figure S89. HPLC spectrum of **4am**

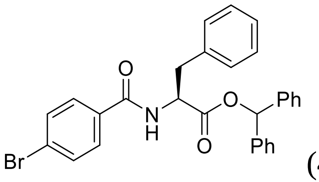

(**4an**)

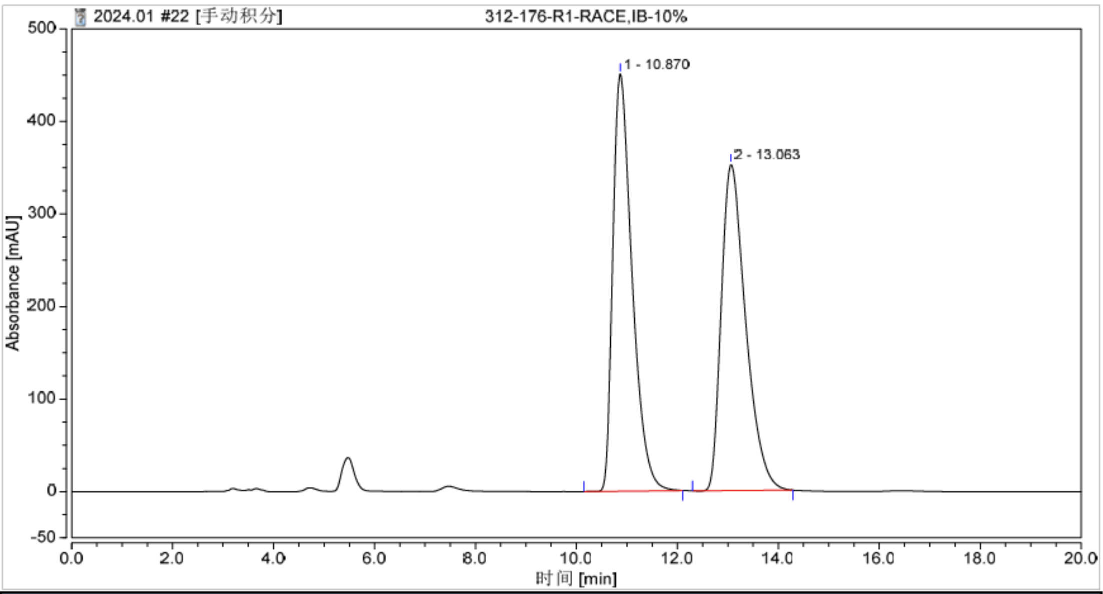

Integration Results

No.	Peak Name	Retention Time min	Area mAu*min	Height mAU	Relative Area %	Relative Height %	Amount n.a.
1		10.870	198.778	450.922	50.13	56.12	n.a.
2		13.063	197.713	352.587	49.87	43.88	n.a.
**Total:**			**396.492**	**803.509**	**100.00**	**100.00**	

Figure S90. HPLC spectrum of racemic **4an**

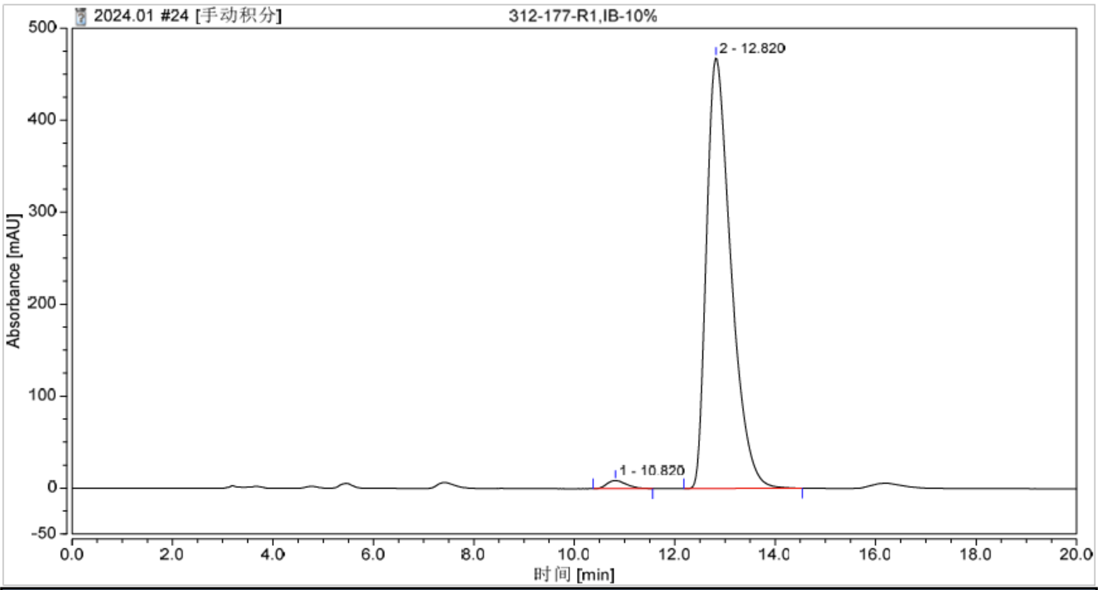

Integration Results

No.	Peak Name	Retention Time min	Area mAu*min	Height mAU	Relative Area %	Relative Height %	Amount n.a.
1		10.820	3.972	8.875	1.48	1.86	n.a.
2		12.820	263.880	468.675	98.52	98.14	n.a.
**Total:**			**267.852**	**477.549**	**100.00**	**100.00**	

Figure S91. HPLC spectrum of **4an**

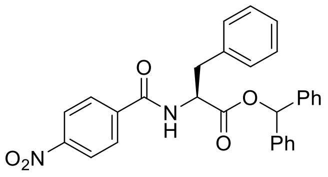

(**4ao**)

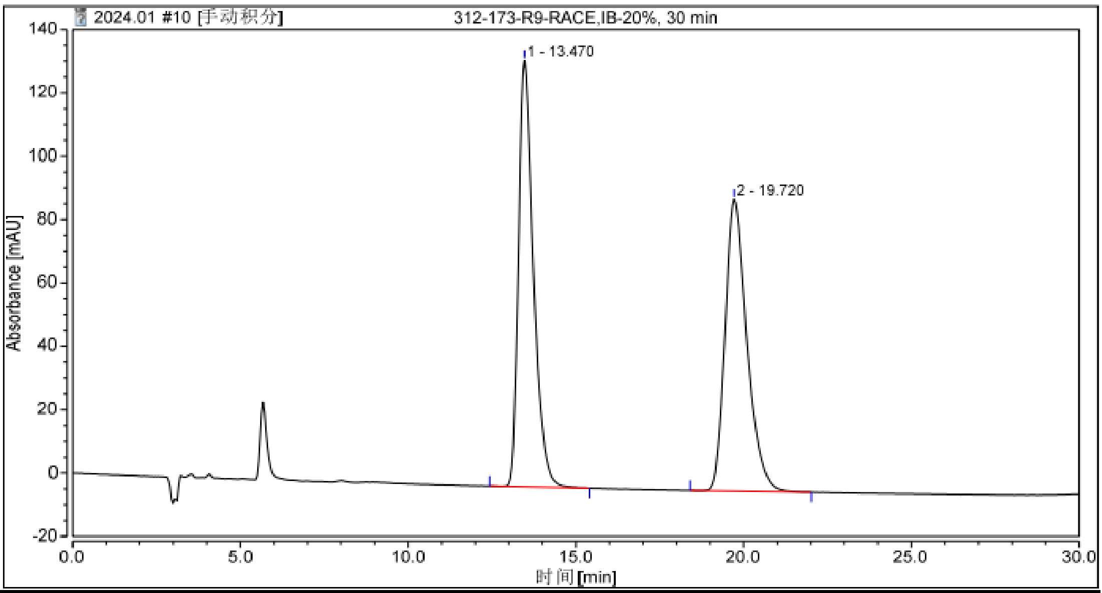

Integration Results							
No.	Peak Name	Retention Time min	Area mAu*min	Height mAU	Relative Area %	Relative Height %	Amount n.a.
1		13.470	69.275	134.624	49.99	59.40	n.a.
2		19.720	69.316	92.010	50.01	40.60	n.a.
Total:			138.590	226.634	100.00	100.00	

Figure S92. HPLC spectrum of racemic **4ao**

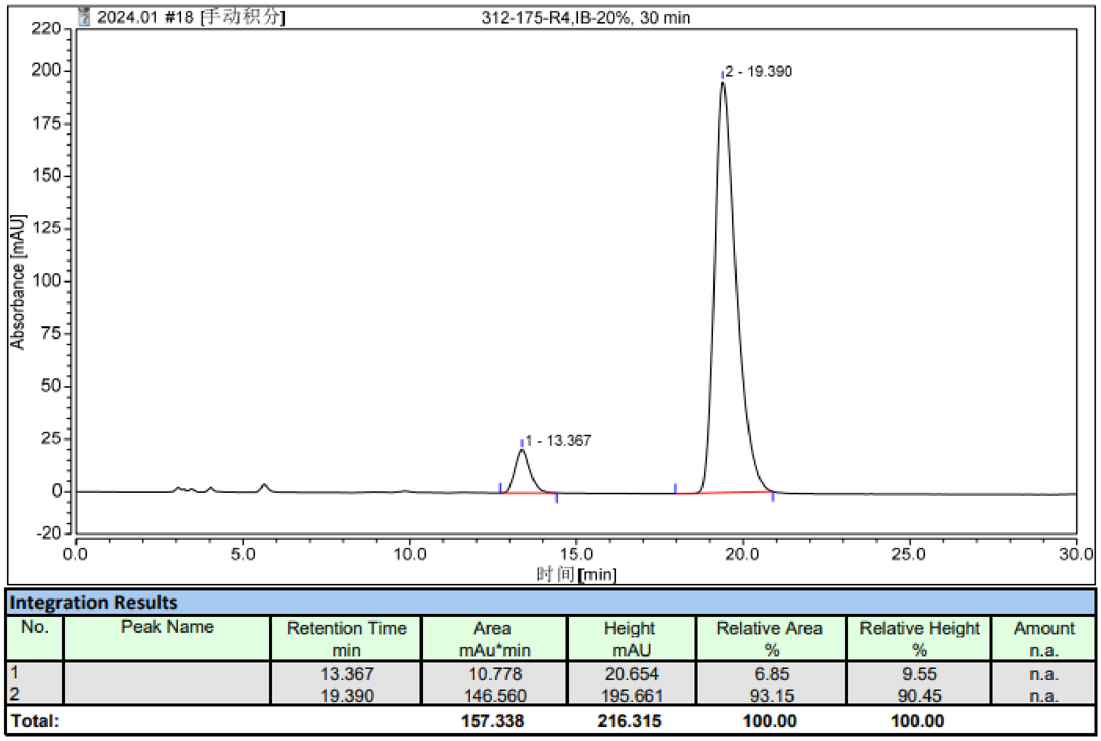

Integration Results							
No.	Peak Name	Retention Time min	Area mAu*min	Height mAU	Relative Area %	Relative Height %	Amount n.a.
1		13.367	10.778	20.654	6.85	9.55	n.a.
2		19.390	146.560	195.661	93.15	90.45	n.a.
Total:			157.338	216.315	100.00	100.00	

Figure S93. HPLC spectrum of **4ao**

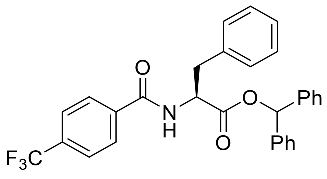

(4ap)

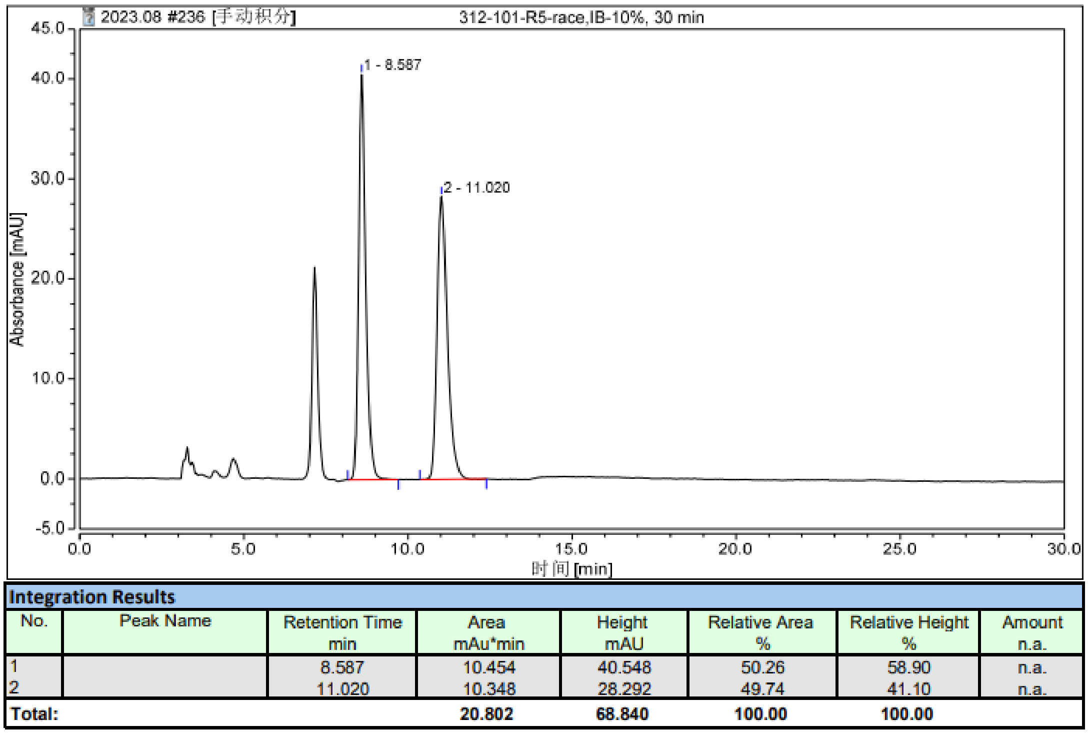

Integration Results							
No.	Peak Name	Retention Time min	Area mAu*min	Height mAU	Relative Area %	Relative Height %	Amount n.a.
1		8.587	10.454	40.548	50.26	58.90	n.a.
2		11.020	10.348	28.292	49.74	41.10	n.a.
Total:			20.802	68.840	100.00	100.00	

Figure S94. HPLC spectrum of racemic **4ap**

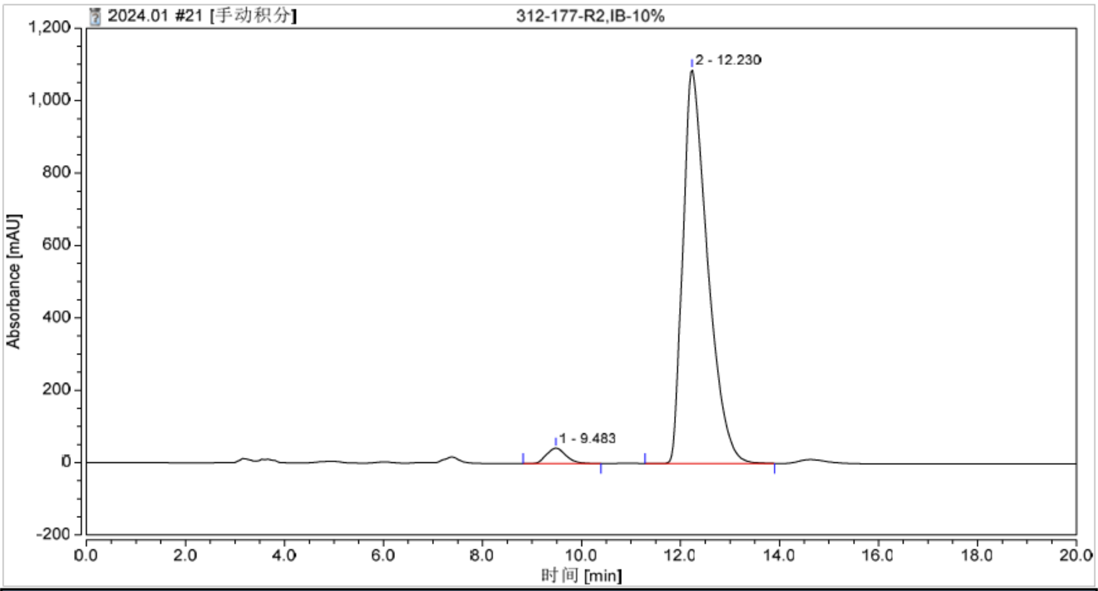

Integration Results							
No.	Peak Name	Retention Time min	Area mAu*min	Height mAU	Relative Area %	Relative Height %	Amount n.a.
1		9.483	19.169	42.639	2.98	3.77	n.a.
2		12.230	624.405	1088.318	97.02	96.23	n.a.
Total:			643.574	1130.957	100.00	100.00	

Figure S95. HPLC spectrum of **4ap**

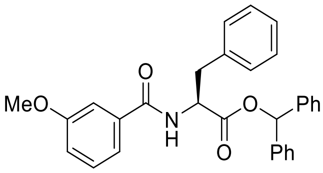

(4aq)

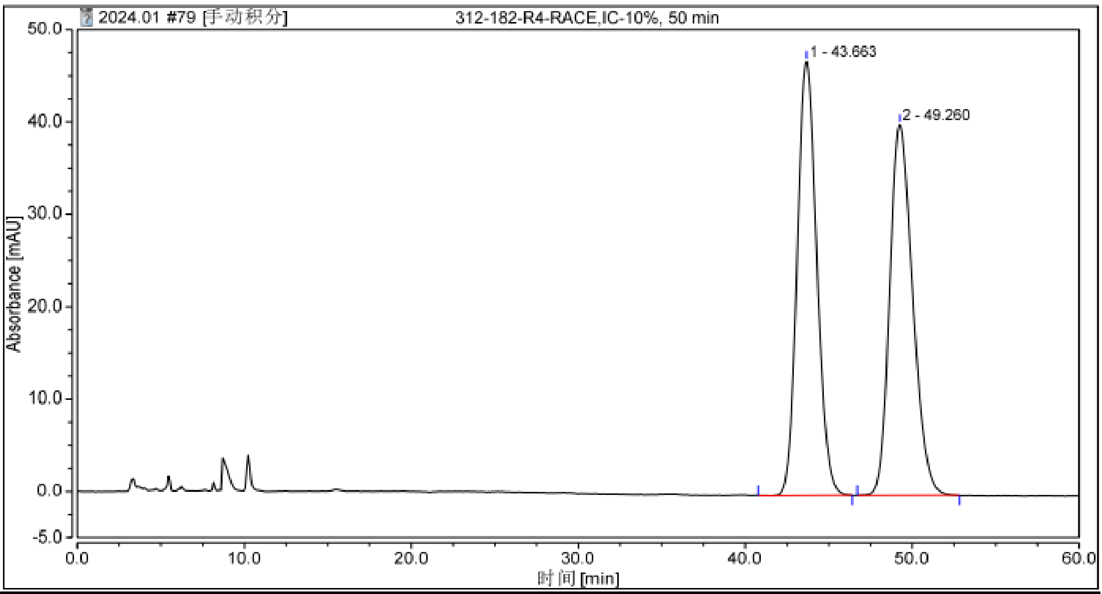

Integration Results

No.	Peak Name	Retention Time min	Area mAu*min	Height mAU	Relative Area %	Relative Height %	Amount n.a.
1		43.663	63.892	46.991	49.95	53.94	n.a.
2		49.260	64.030	40.132	50.05	46.06	n.a.
**Total:**			**127.923**	**87.123**	**100.00**	**100.00**	

Figure S96. HPLC spectrum of racemic **4aq**

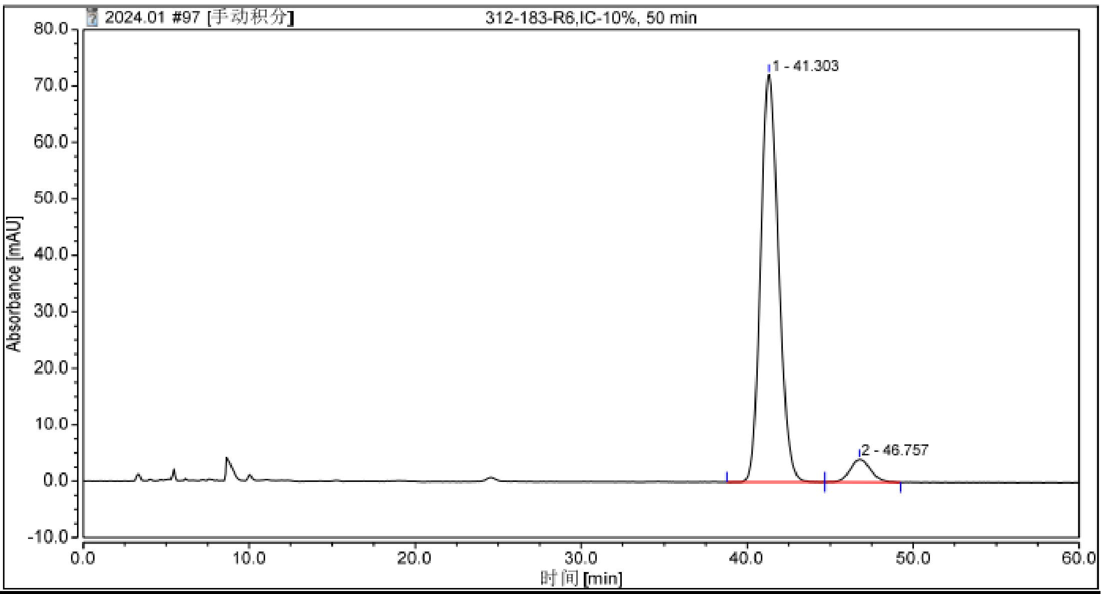

Integration Results

No.	Peak Name	Retention Time min	Area mAu*min	Height mAU	Relative Area %	Relative Height %	Amount n.a.
1		41.303	90.118	72.167	93.94	94.71	n.a.
2		46.757	5.813	4.028	6.06	5.29	n.a.
**Total:**			**95.931**	**76.195**	**100.00**	**100.00**	

Figure S97. HPLC spectrum of **4aq**

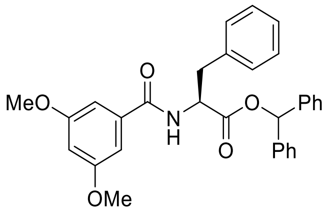

(4ar)

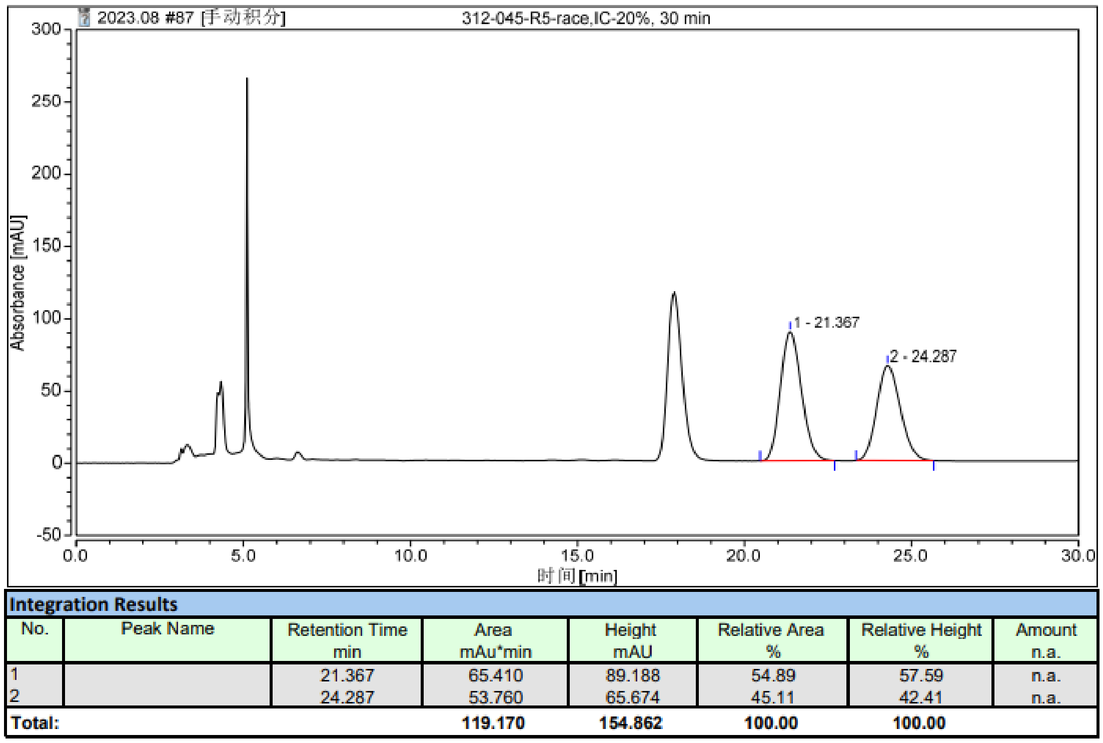

Integration Results

No.	Peak Name	Retention Time min	Area mAu*min	Height mAU	Relative Area %	Relative Height %	Amount n.a.
1		21.367	65.410	89.188	54.89	57.59	n.a.
2		24.287	53.760	65.674	45.11	42.41	n.a.
**Total:**			**119.170**	**154.862**	**100.00**	**100.00**	

Figure S98. HPLC spectrum of racemic **4ar**

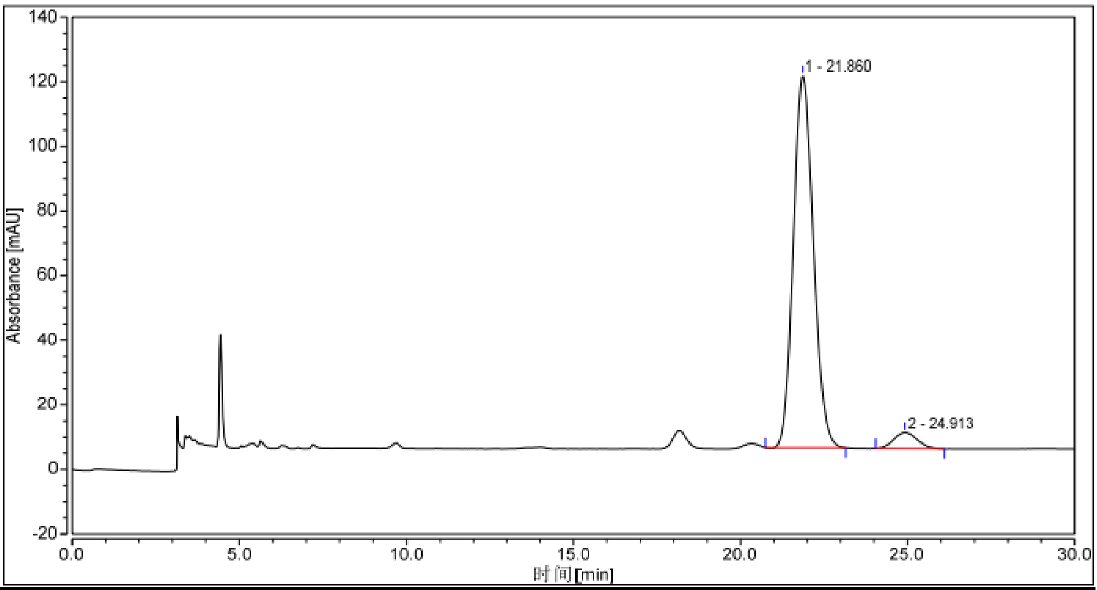

Integration Results

No.	Peak Name	Retention Time min	Area mAu*min	Height mAU	Relative Area %	Relative Height %	Amount n.a.
1		21.860	79.987	115.210	95.20	95.83	n.a.
2		24.913	4.029	5.009	4.80	4.17	n.a.
**Total:**			**84.017**	**120.219**	**100.00**	**100.00**	

Figure S99. HPLC spectrum of **4ar**

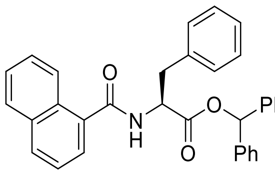

(4as)

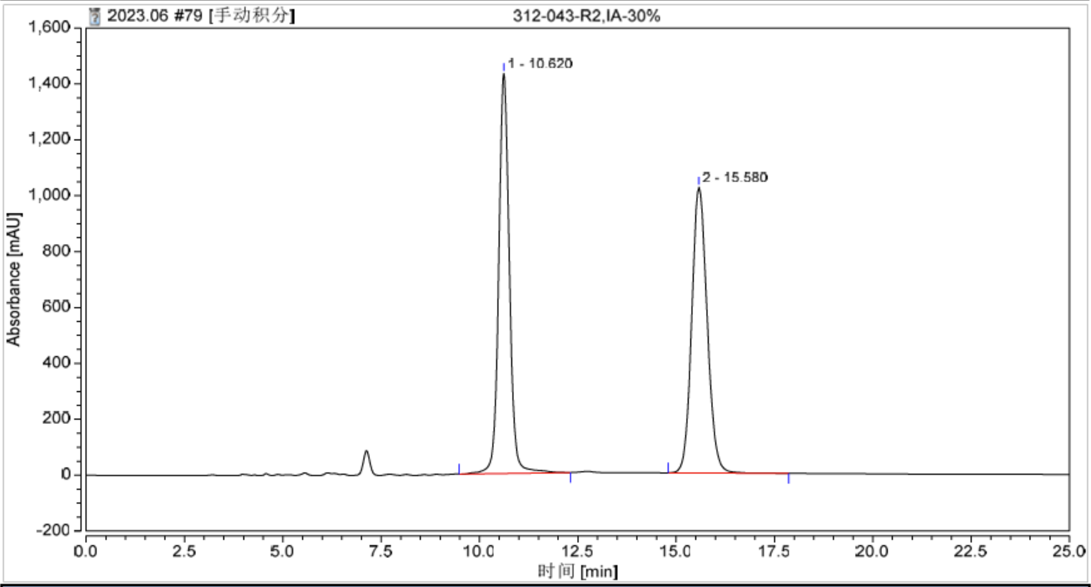

Integration Results

No.	Peak Name	Retention Time min	Area mAu*min	Height mAU	Relative Area %	Relative Height %	Amount n.a.
1		10.620	448.449	1431.802	49.09	58.33	n.a.
2		15.580	464.982	1022.676	50.91	41.67	n.a.
**Total:**			**913.430**	**2454.478**	**100.00**	**100.00**	

Figure S100. HPLC spectrum of racemic **4as**

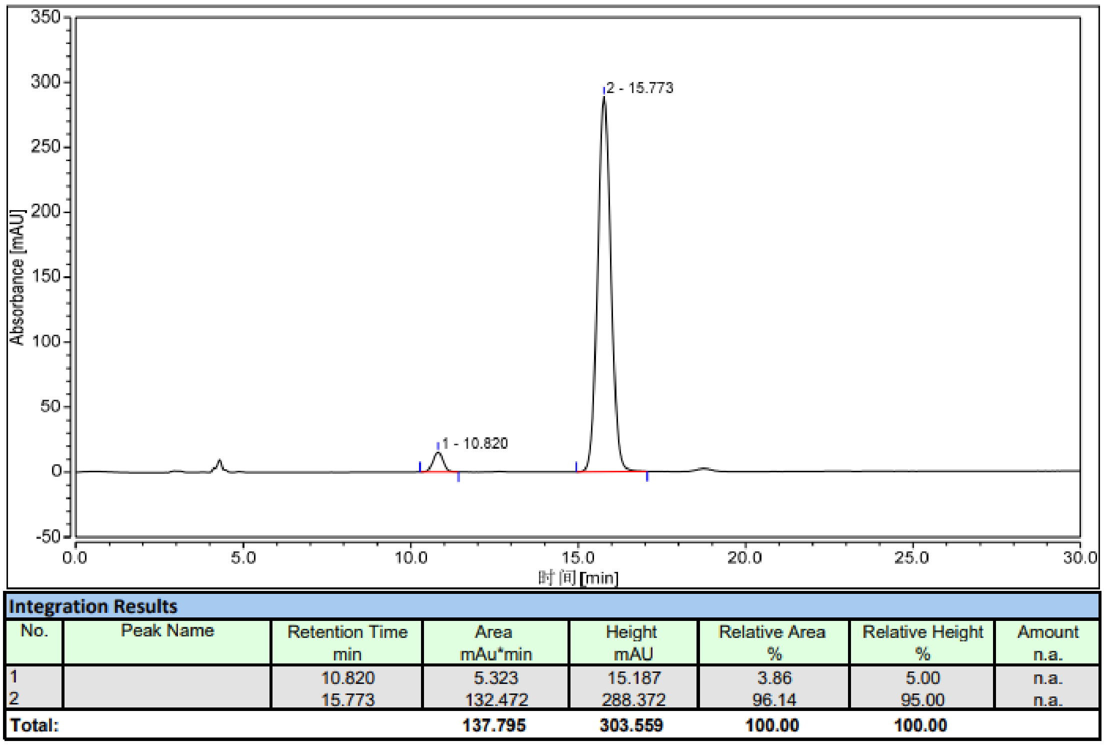

Integration Results

No.	Peak Name	Retention Time min	Area mAu*min	Height mAU	Relative Area %	Relative Height %	Amount n.a.
1		10.820	5.323	15.187	3.86	5.00	n.a.
2		15.773	132.472	288.372	96.14	95.00	n.a.
**Total:**			**137.795**	**303.559**	**100.00**	**100.00**	

Figure S101. HPLC spectrum of **4as**

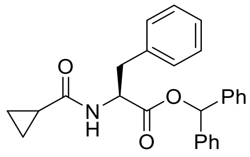

(4at)

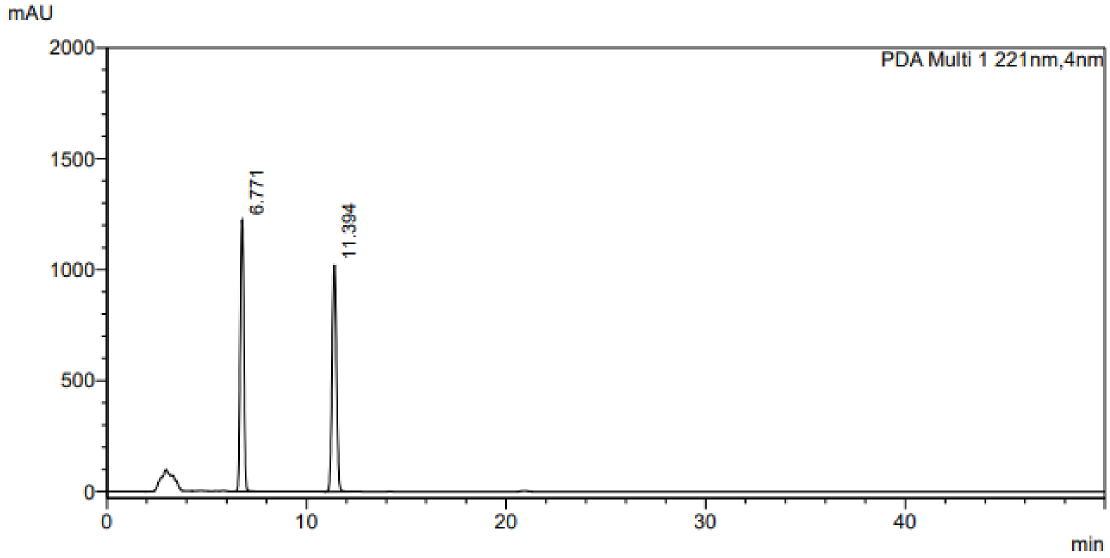

<Peak Table>

PDA Ch1 221nm

Peak#	Ret. Time	Area	Height	Area%	Height%
1	6.771	15392049	1222432	49.891	54.440
2	11.394	15459045	1023028	50.109	45.560
Total		30851094	2245460	100.000	100.000

Figure S102. HPLC spectrum of racemic **4at**

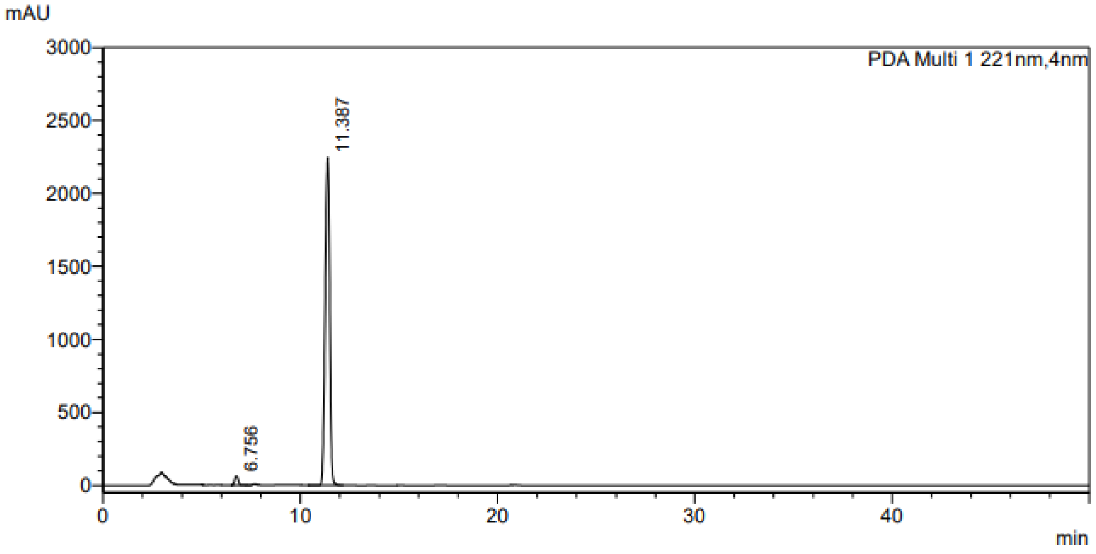

<Peak Table>

PDA Ch1 221nm

Peak#	Ret. Time	Area	Height	Area%	Height%
1	6.756	814560	63602	2.155	2.757
2	11.387	36979918	2243038	97.845	97.243
Total		37794478	2306639	100.000	100.000

Figure S103. HPLC spectrum of **4at**

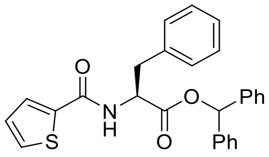

(**4au**)

Chromatogram

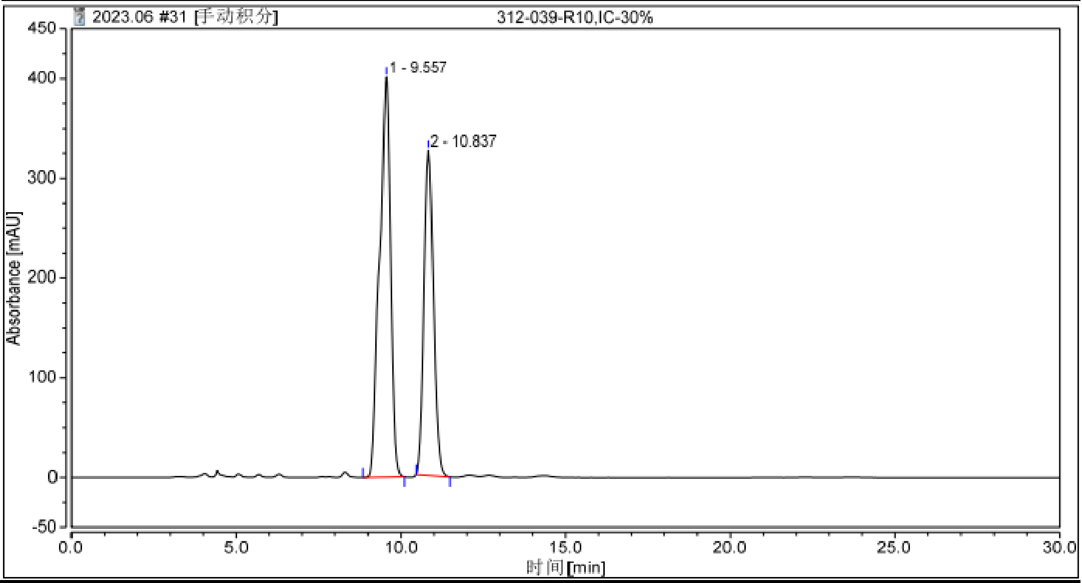

Integration Results

No.	Peak Name	Retention Time min	Area mAu*min	Height mAU	Relative Area %	Relative Height %	Amount n.a.
1		9.557	155.537	401.313	58.62	55.17	n.a.
2		10.837	109.810	326.044	41.38	44.83	n.a.
**Total:**			**265.348**	**727.358**	**100.00**	**100.00**	

Figure S104. HPLC spectrum of racemic **4au**

Chromatogram

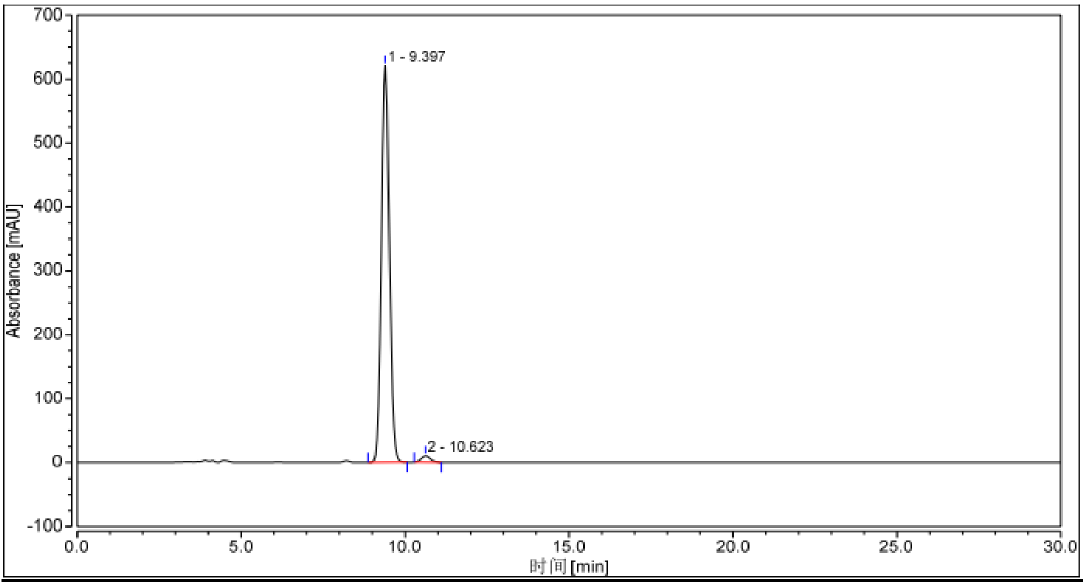

Integration Results

No.	Peak Name	Retention Time min	Area mAu*min	Height mAU	Relative Area %	Relative Height %	Amount n.a.
1		9.397	175.105	621.198	98.18	98.40	n.a.
2		10.623	3.249	10.101	1.82	1.60	n.a.
**Total:**			**178.354**	**631.299**	**100.00**	**100.00**	

Figure S105. HPLC spectrum of **4au**

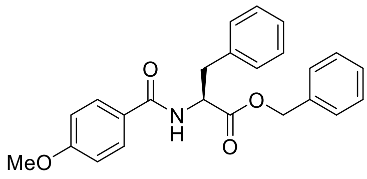

(4av)

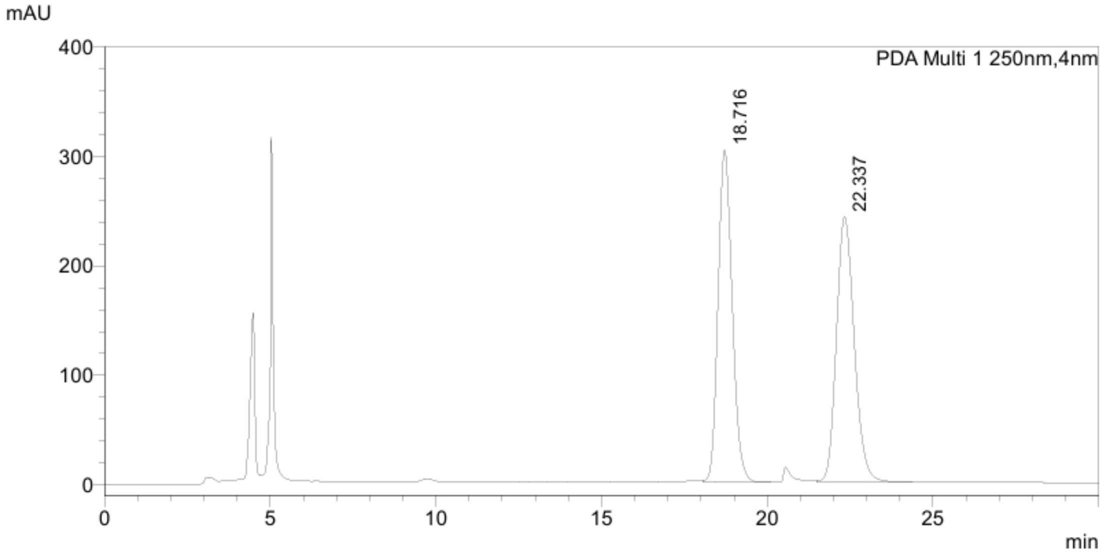

<Peak Table>

PDA Ch1 250nm

Peak#	Ret. Time	Area	Height	Area%	Height%
1	18.716	8991920	303447	49.707	55.582
2	22.337	9098088	242502	50.293	44.418
Total		18090007	545949	100.000	100.000

Figure S106. HPLC spectrum of racemic **4av**

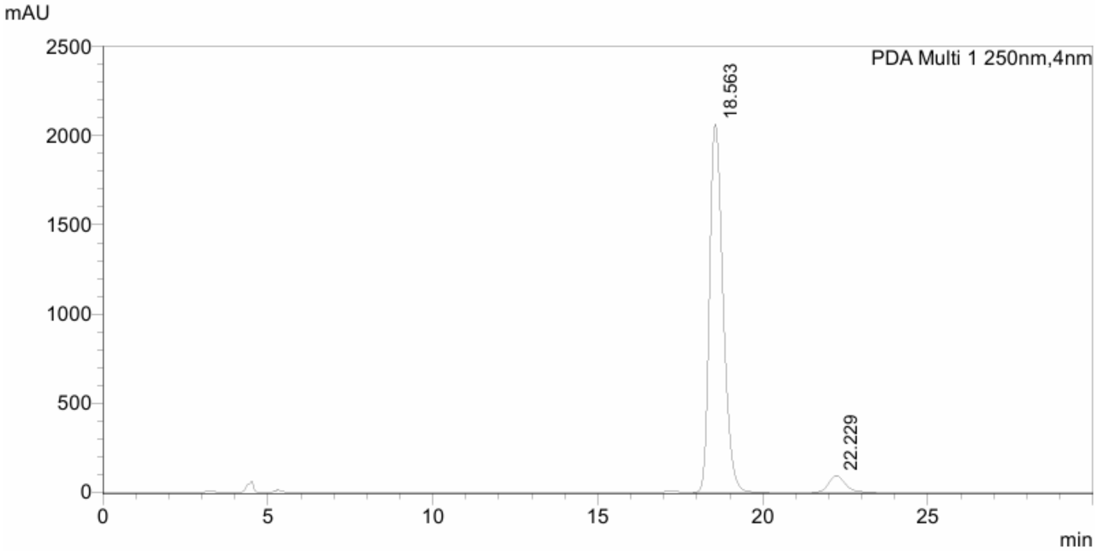

<Peak Table>

PDA Ch1 250nm

Peak#	Ret. Time	Area	Height	Area%	Height%
1	18.563	58698521	2063908	94.993	95.744
2	22.229	3094144	91754	5.007	4.256
Total		61792665	2155662	100.000	100.000

Figure S107. HPLC spectrum of **4av**

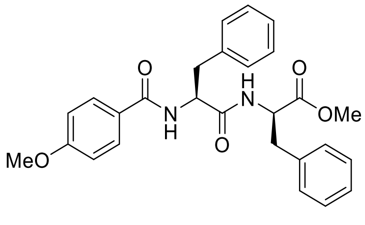

(4aw)

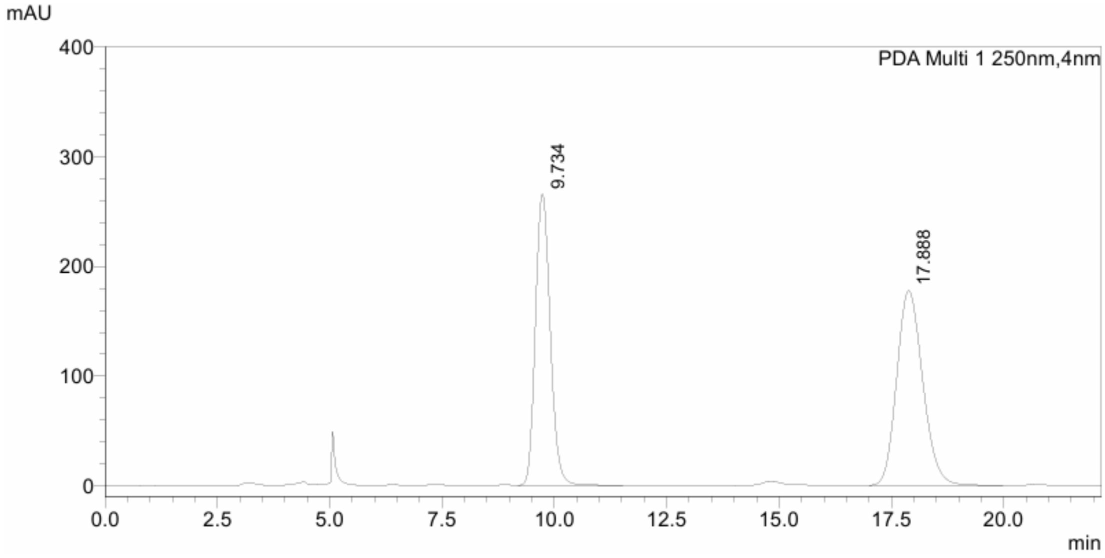

<Peak Table>

PDA Ch1 250nm

Peak#	Ret. Time	Area	Height	Area%	Height%
1	9.734	6073845	265212	45.891	59.906
2	17.888	7161502	177501	54.109	40.094
Total		13235347	442713	100.000	100.000

Figure S108. HPLC spectrum of racemic **4aw**

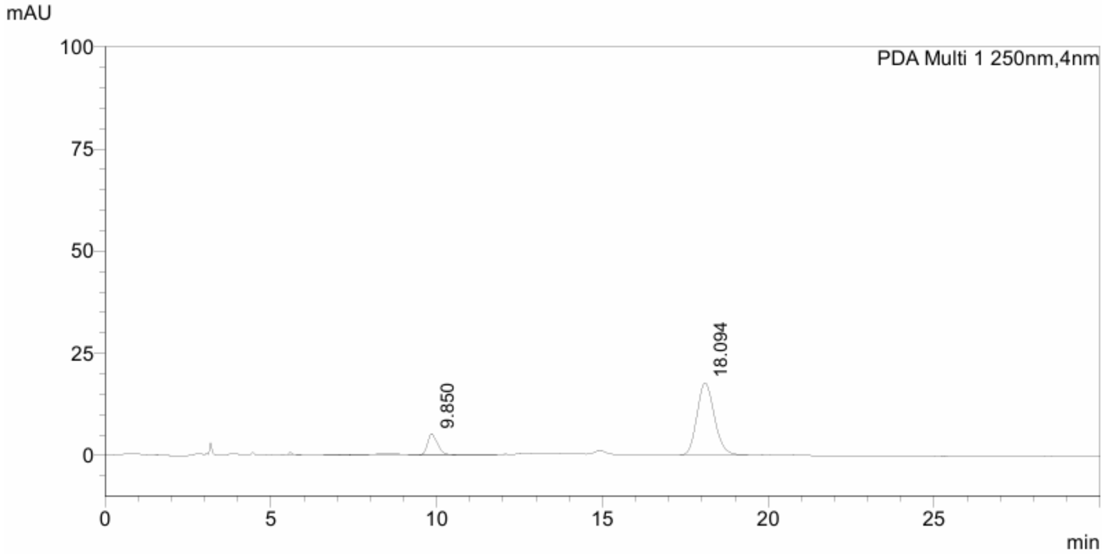

<Peak Table>

PDA Ch1 250nm

Peak#	Ret. Time	Area	Height	Area%	Height%
1	9.850	104584	5158	13.955	22.600
2	18.094	644843	17666	86.045	77.400
Total		749427	22825	100.000	100.000

Figure S109. HPLC spectrum of **4aw**

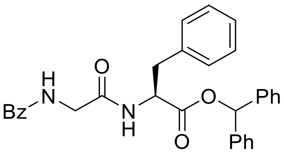

(6a)

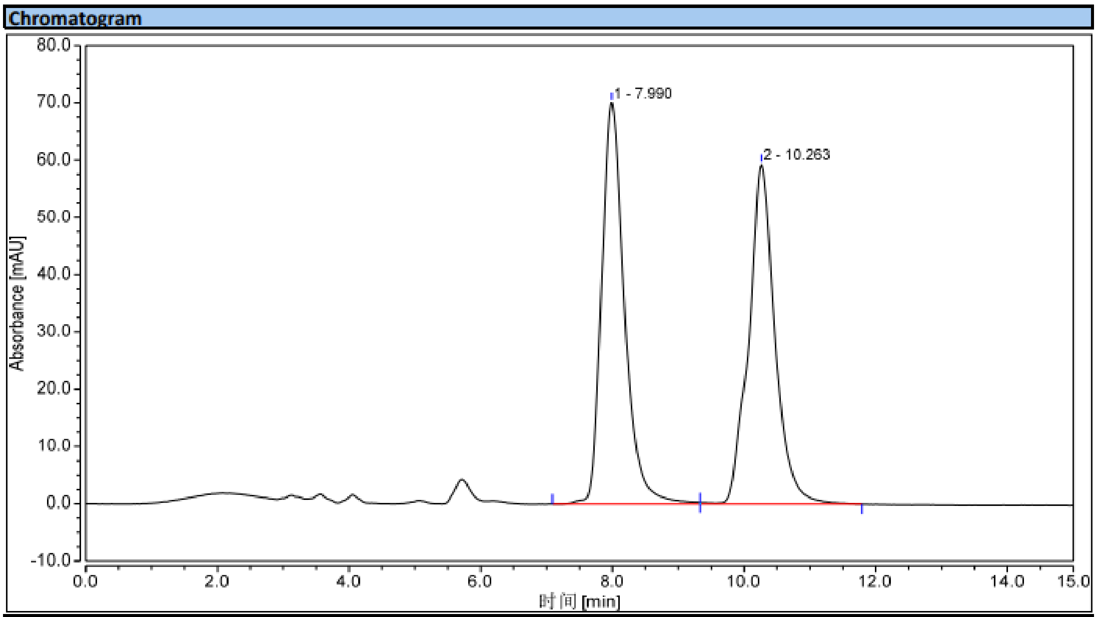

Integration Results

No.	Peak Name	Retention Time min	Area mAu*min	Height mAU	Relative Area %	Relative Height %	Amount n.a.
1		7.990	28.283	70.104	50.81	54.18	n.a.
2		10.263	27.381	59.286	49.19	45.82	n.a.
**Total:**			**55.664**	**129.390**	**100.00**	**100.00**	

Figure S110. HPLC spectrum of racemic **6a**

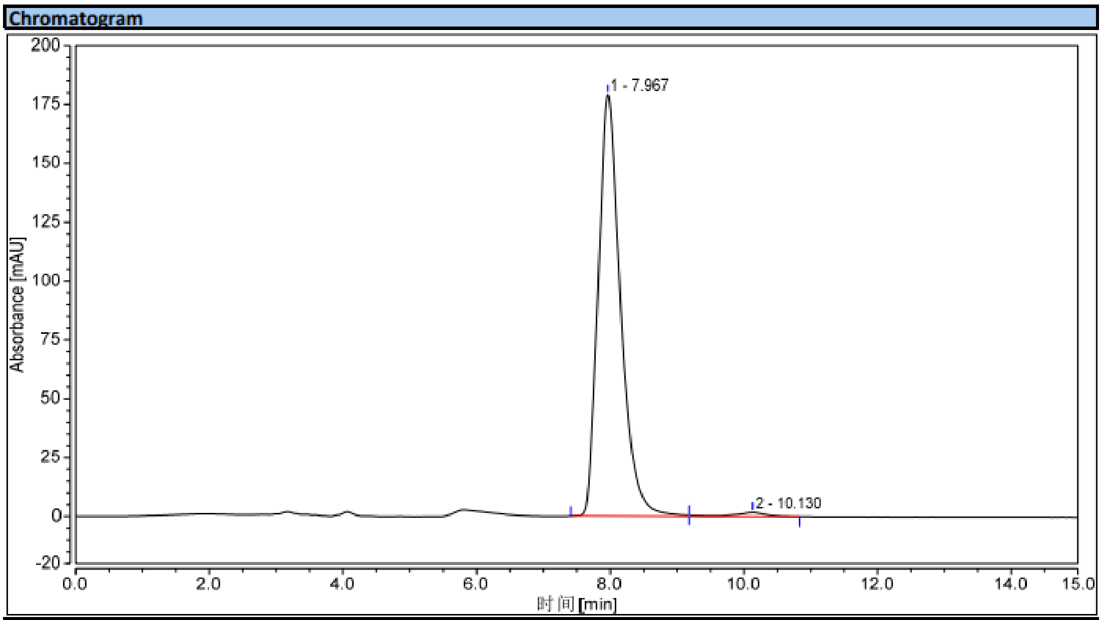

Integration Results

No.	Peak Name	Retention Time min	Area mAu*min	Height mAU	Relative Area %	Relative Height %	Amount n.a.
1		7.967	69.643	179.066	98.67	99.06	n.a.
2		10.130	0.942	1.704	1.33	0.94	n.a.
**Total:**			**70.584**	**180.770**	**100.00**	**100.00**	

Figure S111. HPLC spectrum of **6a**

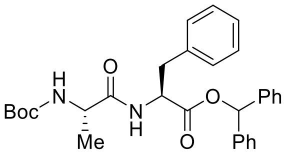

(**6b**)

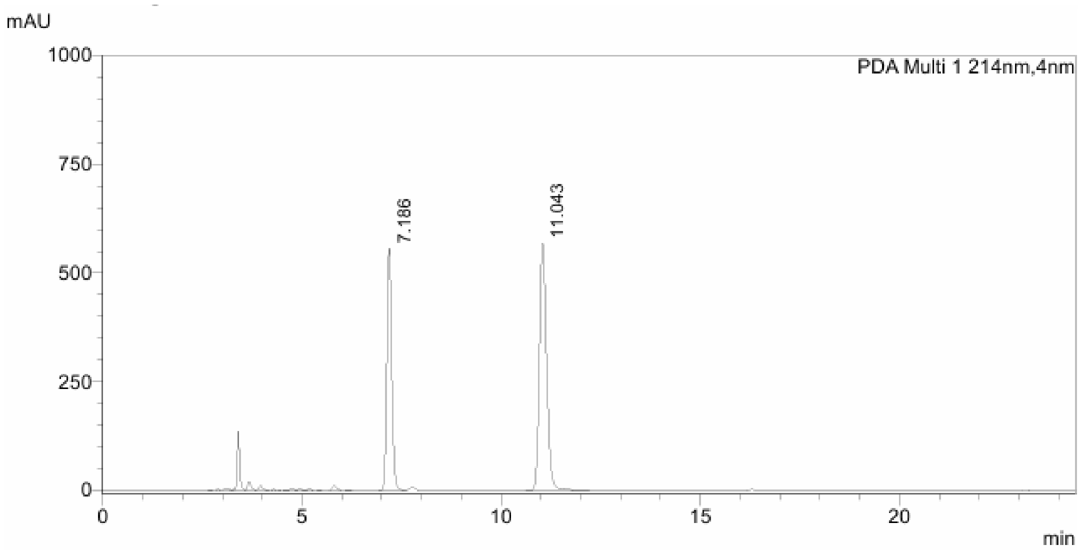

<Peak Table>

PDA Ch1 214nm

Peak#	Ret. Time	Area	Height	Area%	Height%
1	7.186	4658179	556599	39.441	49.472
2	11.043	7152420	568475	60.559	50.528
Total		11810599	1125074	100.000	100.000

Figure S112. HPLC spectrum of racemic **6b**

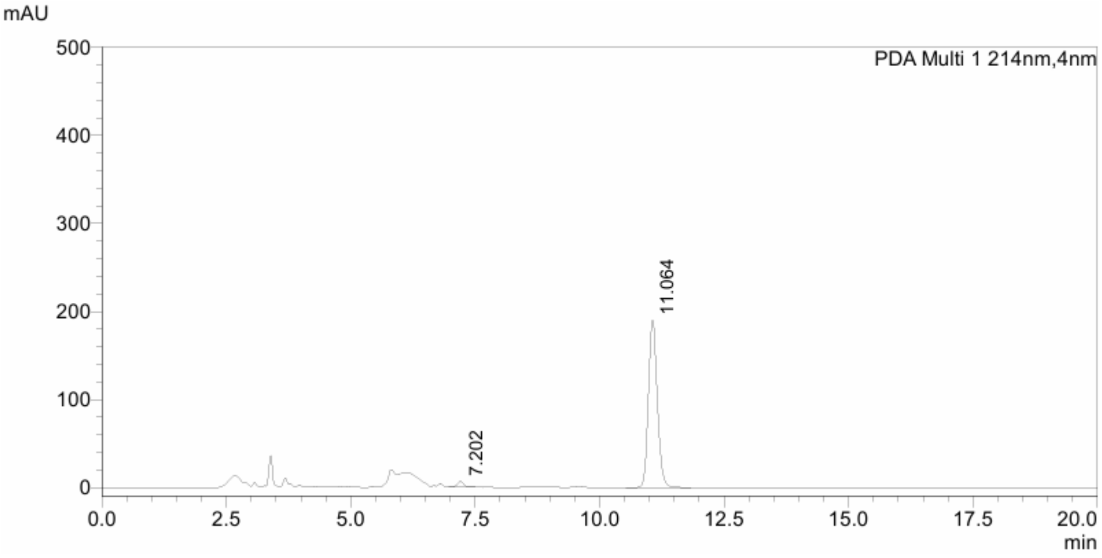

<Peak Table>

PDA Ch1 214nm

Peak#	Ret. Time	Area	Height	Area%	Height%
1	7.202	53825	6166	2.227	3.142
2	11.064	2363405	190052	97.773	96.858
Total		2417230	196218	100.000	100.000

Figure S113. HPLC spectrum of **6b**

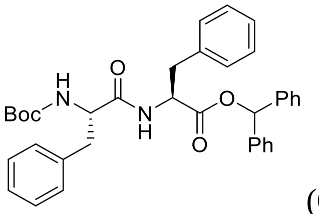

(**6c**)

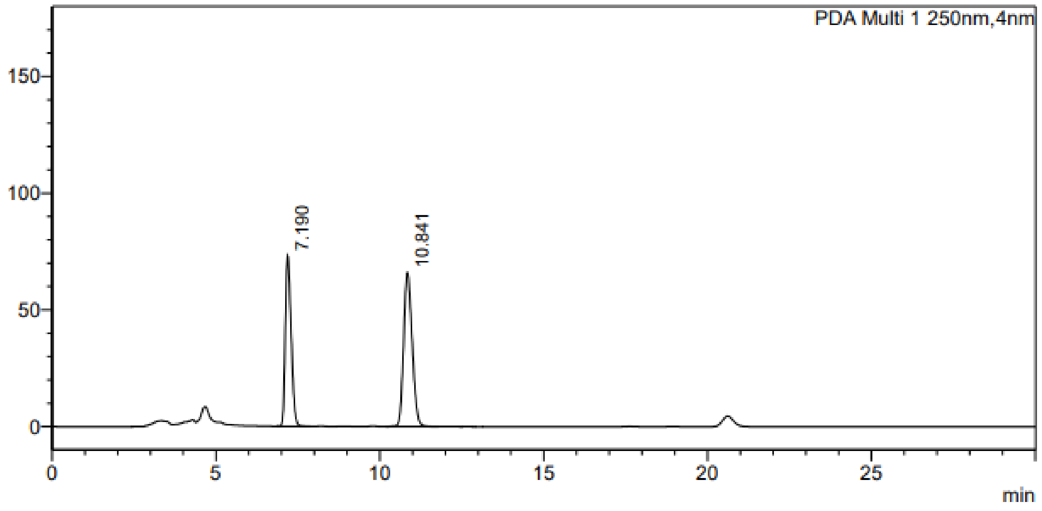

<Peak Table>

PDA Ch1 250nm

Peak#	Ret. Time	Area	Height	Area%	Height%
1	7.190	914048	73043	43.205	52.526
2	10.841	1201575	66017	56.795	47.474
Total		2115623	139060	100.000	100.000

Figure S114. HPLC spectrum of racemic **6c**

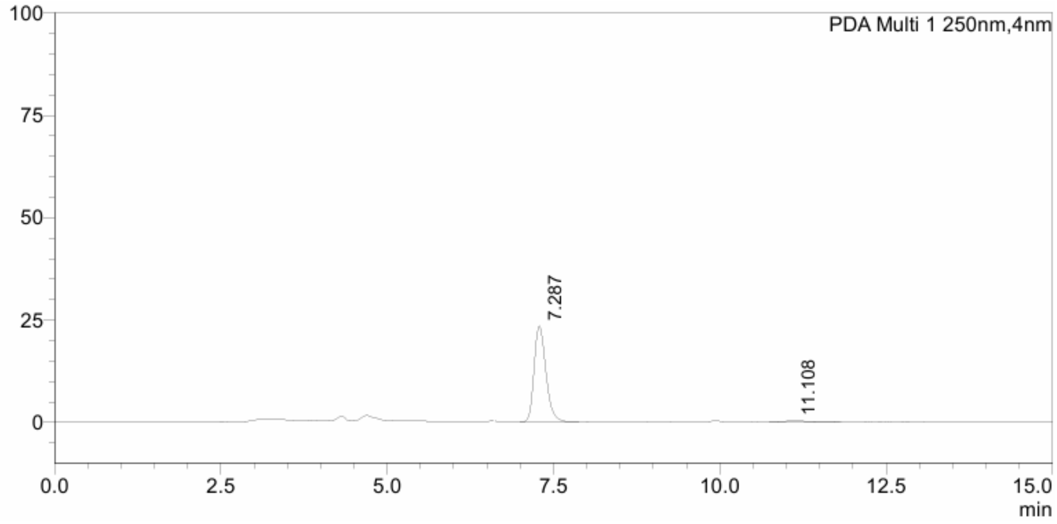

<Peak Table>

PDA Ch1 250nm

Peak#	Ret. Time	Area	Height	Area%	Height%
1	7.287	287395	23430	97.730	98.480
2	11.108	6675	362	2.270	1.520
Total		294070	23791	100.000	100.000

Figure S115. HPLC spectrum of **6c**

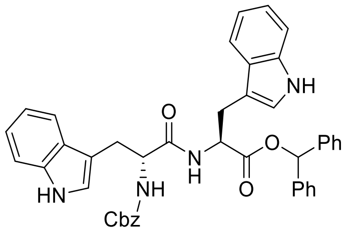

(**6d**)

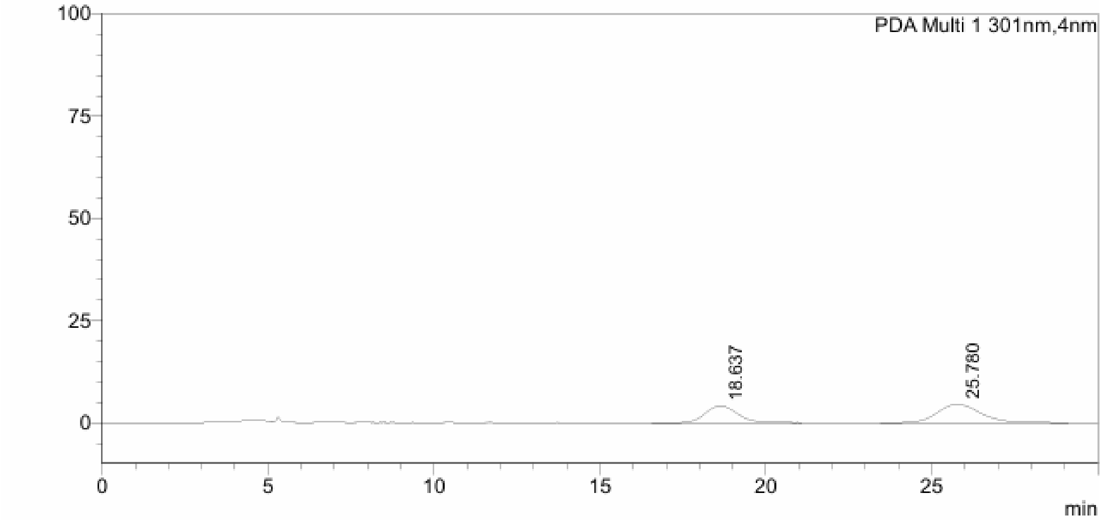

<Peak Table>

PDA Ch1 301nm

Peak#	Ret. Time	Area	Height	Area%	Height%
1	18.637	269018	4081	40.162	47.709
2	25.780	400816	4473	59.838	52.291
Total		669834	8555	100.000	100.000

Figure S116. HPLC spectrum of racemic **6d**

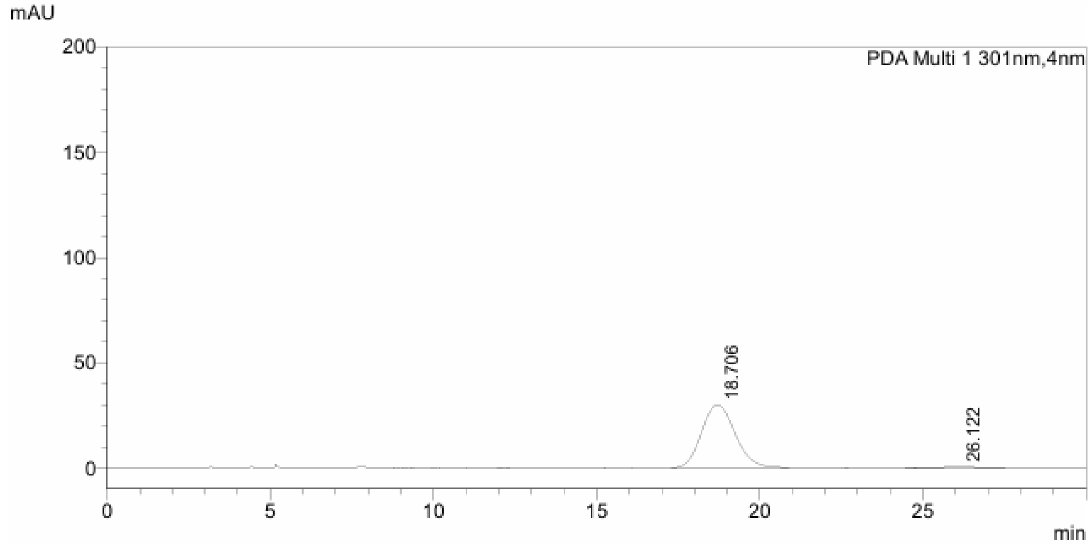

<Peak Table>

PDA Ch1 301nm

Peak#	Ret. Time	Area	Height	Area%	Height%
1	18.706	2118144	29646	98.581	98.821
2	26.122	30479	354	1.419	1.179
Total		2148623	29999	100.000	100.000

Figure S117. HPLC spectrum of **6d**

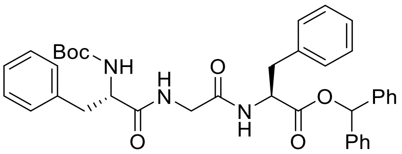

(**6e**)

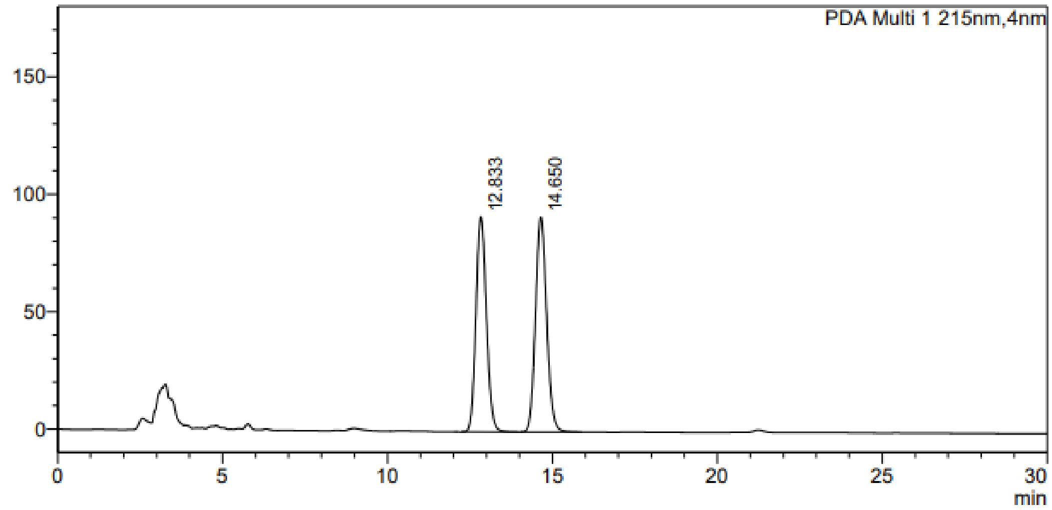

<Peak Table>

PDA Ch1 215nm

Peak#	Ret. Time	Area	Height	Area%	Height%
1	12.833	2011880	91425	48.199	49.996
2	14.650	2162228	91441	51.801	50.004
Total		4174108	182866	100.000	100.000

Figure S118. HPLC spectrum of racemic **6e**

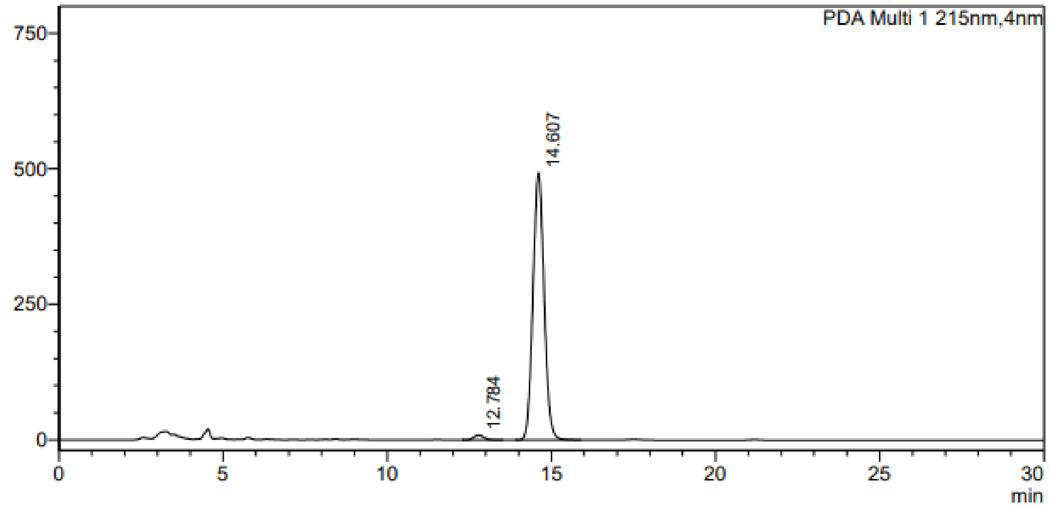

<Peak Table>

PDA Ch1 215nm

Peak#	Ret. Time	Area	Height	Area%	Height%
1	12.784	196888	8906	1.664	1.773
2	14.607	11632475	493371	98.336	98.227
Total		11829364	502278	100.000	100.000

Figure S119. HPLC spectrum of **6e**

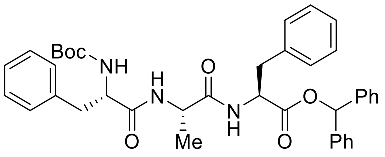

(6f)

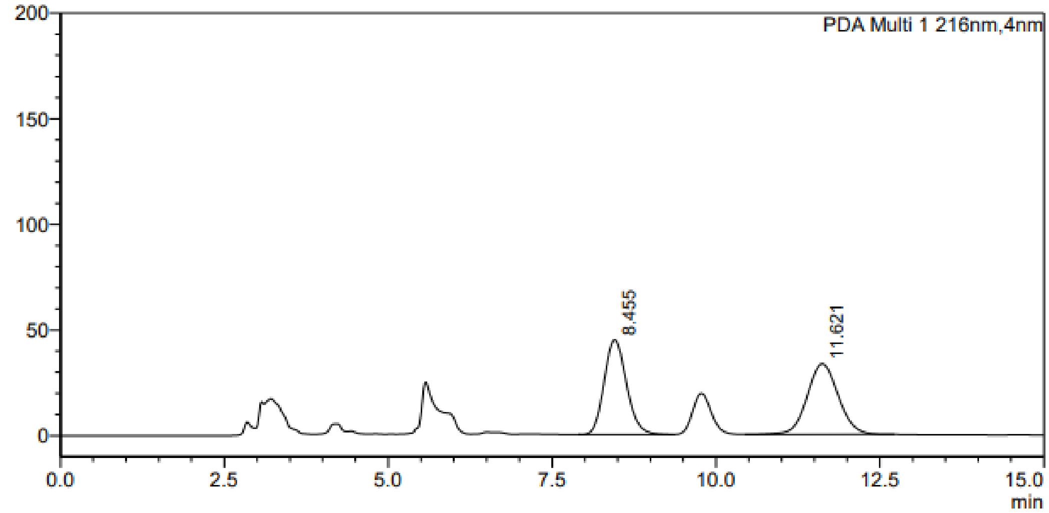

<Peak Table>

PDA Ch1 216nm

Peak#	Ret. Time	Area	Height	Area%	Height%
1	8.455	1071087	44928	48.964	57.336
2	11.621	1116420	33432	51.036	42.664
Total		2187507	78360	100.000	100.000

Figure S120. HPLC spectrum of racemic **4f**

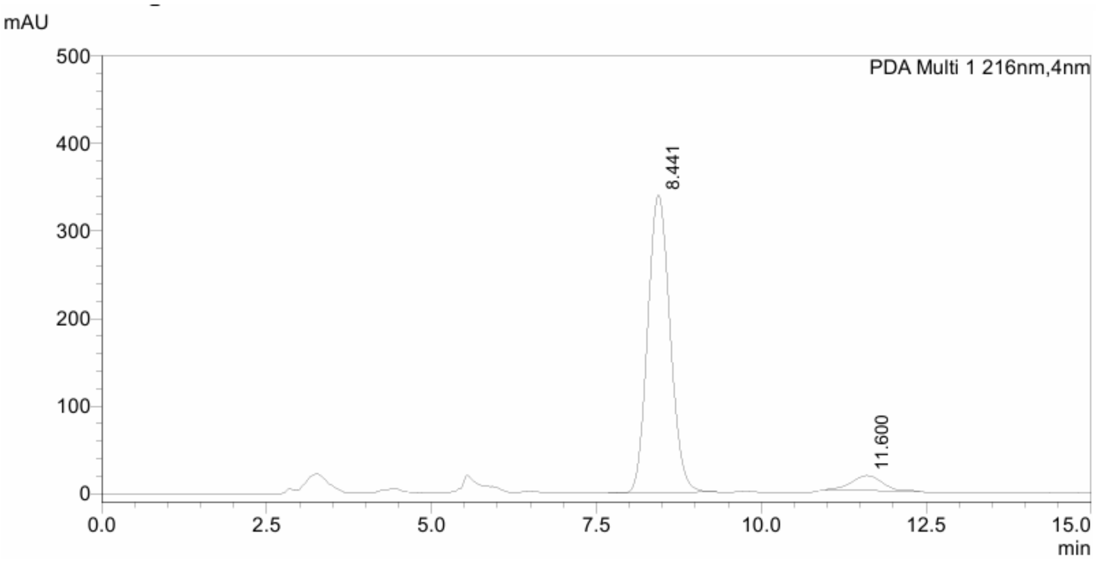

<Peak Table>

PDA Ch1 216nm

Peak#	Ret. Time	Area	Height	Area%	Height%
1	8.441	8033597	340034	93.614	95.249
2	11.600	548062	16962	6.386	4.751
Total		8581660	356996	100.000	100.000

Figure S121. HPLC spectrum of **6f**

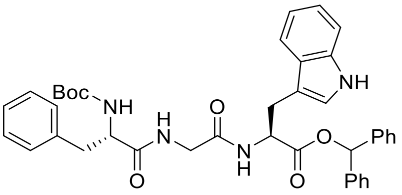

(6g)

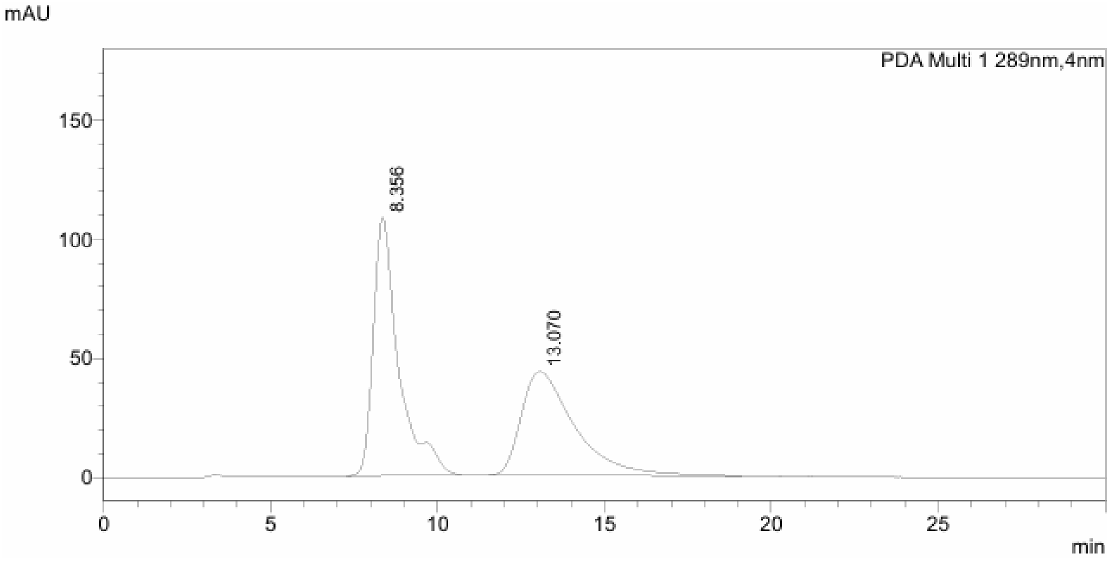

<Peak Table>

PDA Ch1 289nm

Peak#	Ret. Time	Area	Height	Area%	Height%
1	8.356	5749264	108400	54.247	71.417
2	13.070	4849072	43385	45.753	28.583
Total		10598336	151785	100.000	100.000

Figure S122. HPLC spectrum of racemic **6g**

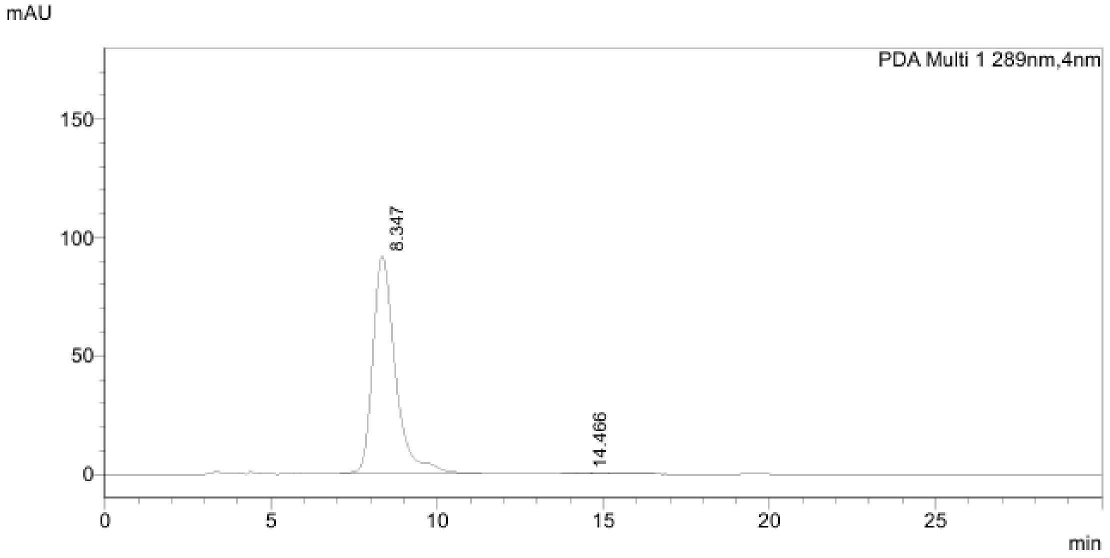

<Peak Table>

PDA Ch1 289nm

Peak#	Ret. Time	Area	Height	Area%	Height%
1	8.347	4363153	91656	99.629	99.744
2	14.466	16241	235	0.371	0.256
Total		4379393	91891	100.000	100.000

Figure S123. HPLC spectrum of **6g**

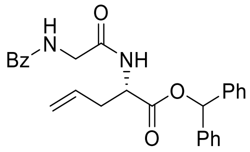

(**6h**)

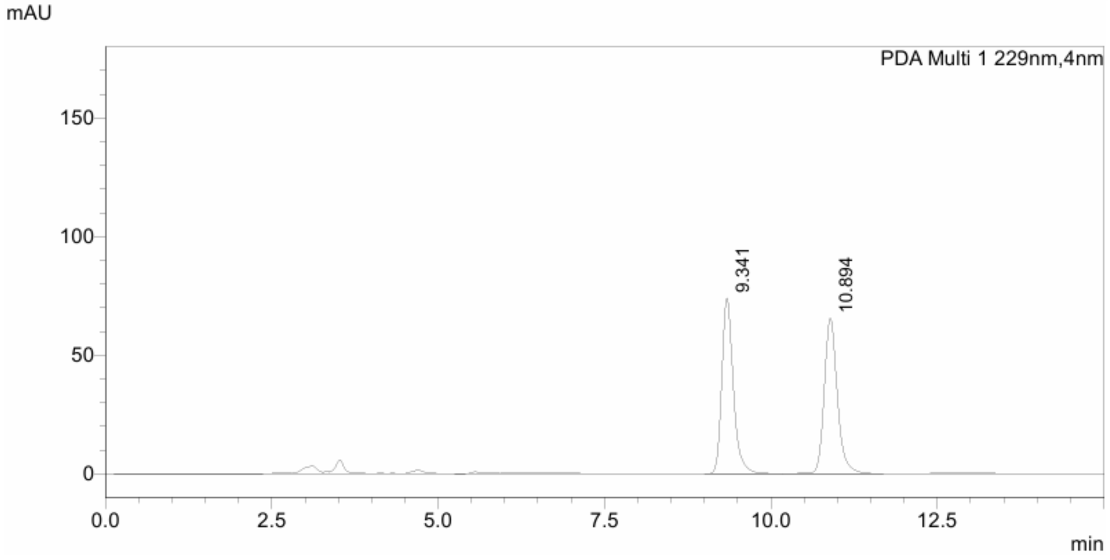

<Peak Table>

PDA Ch1 229nm

Peak#	Ret. Time	Area	Height	Area%	Height%
1	9.341	891211	74010	49.827	53.020
2	10.894	897400	65578	50.173	46.980
Total		1788611	139588	100.000	100.000

Figure S124. HPLC spectrum of racemic **6h**

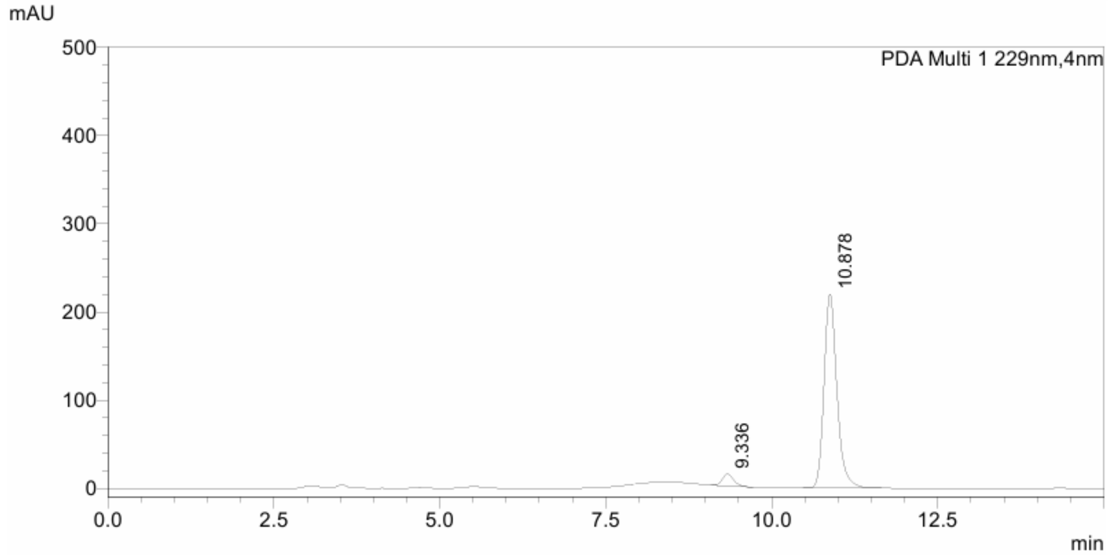

<Peak Table>

PDA Ch1 229nm

Peak#	Ret. Time	Area	Height	Area%	Height%
1	9.336	154734	13537	4.963	5.800
2	10.878	2962859	219849	95.037	94.200
Total		3117593	233387	100.000	100.000

Figure S125. HPLC spectrum of **6h**

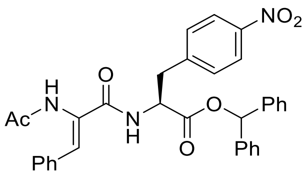

(6i)

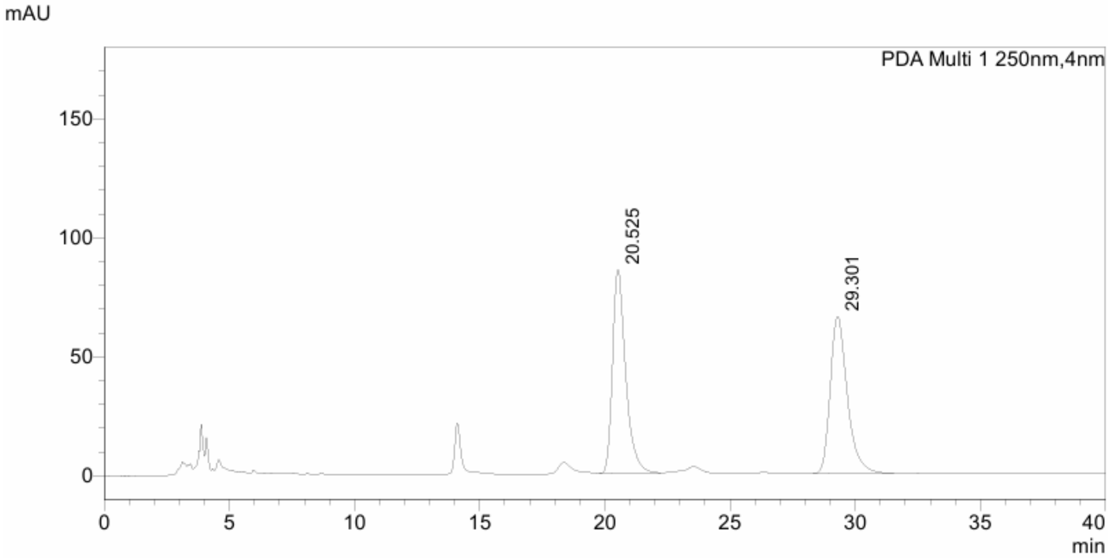

<Peak Table>

PDA Ch1 250nm

Peak#	Ret. Time	Area	Height	Area%	Height%
1	20.525	3118792	85592	49.794	56.448
2	29.301	3144562	66037	50.206	43.552
Total		6263354	151629	100.000	100.000

Figure S126. HPLC spectrum of racemic **6i**

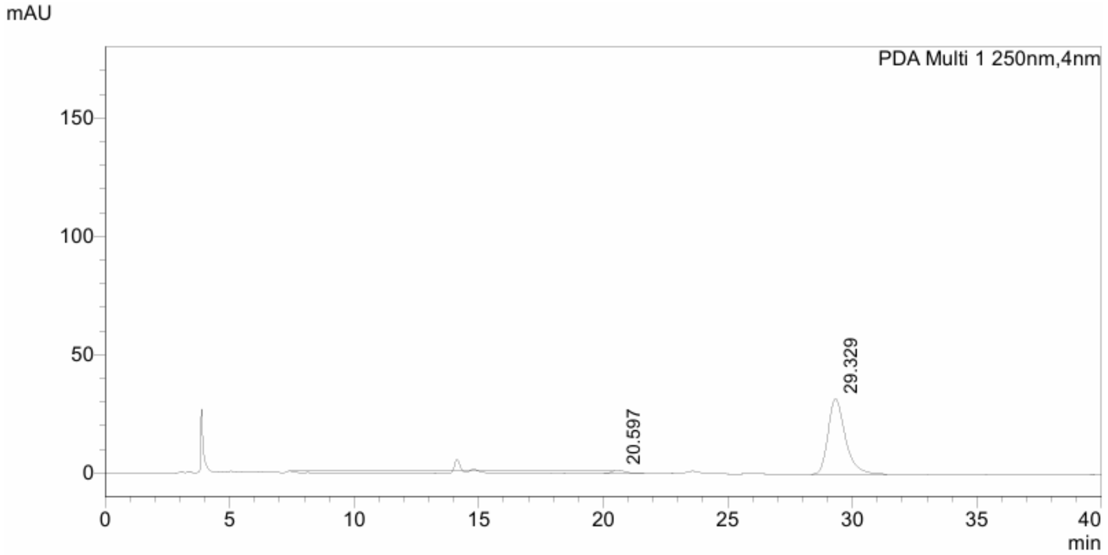

<Peak Table>

PDA Ch1 250nm

Peak#	Ret. Time	Area	Height	Area%	Height%
1	20.597	54714	1464	3.456	4.402
2	29.329	1528269	31790	96.544	95.598
Total		1582983	33254	100.000	100.000

Figure S127. HPLC spectrum of **6i**

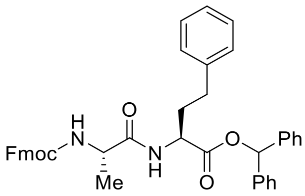

(6j)

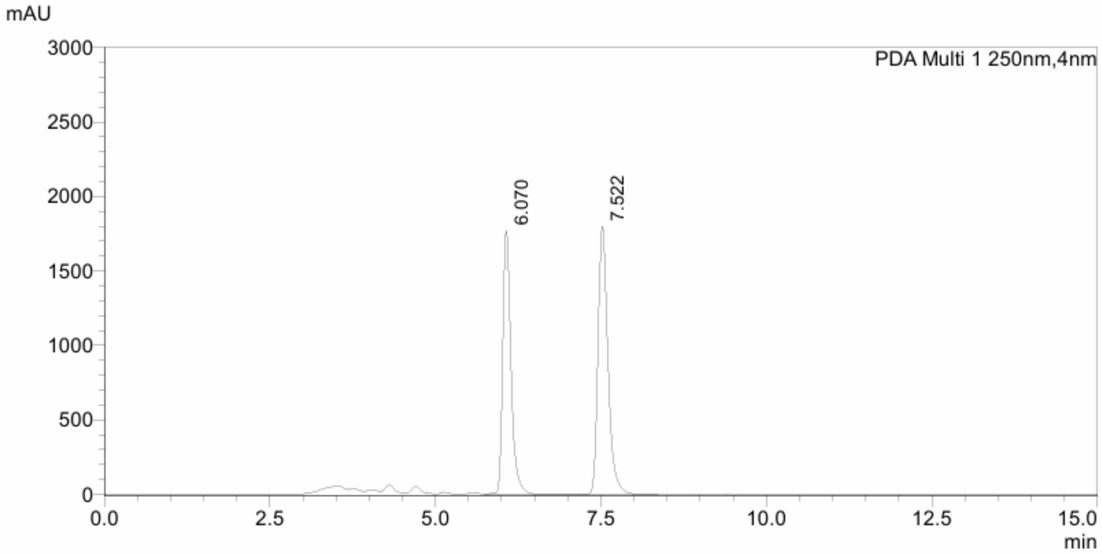

<Peak Table>

PDA Ch1 250nm

Peak#	Ret. Time	Area	Height	Area%	Height%
1	6.070	15418950	1773769	45.279	49.632
2	7.522	18634609	1800100	54.721	50.368
Total		34053560	3573869	100.000	100.000

Figure S128. HPLC spectrum of racemic **6j**

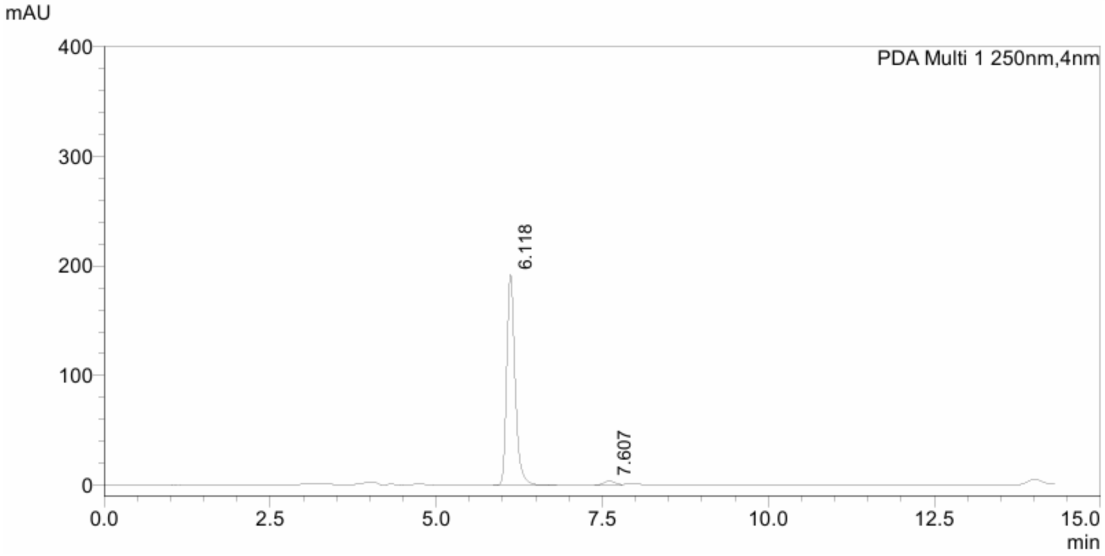

<Peak Table>

PDA Ch1 250nm

Peak#	Ret. Time	Area	Height	Area%	Height%
1	6.118	1672600	191605	97.837	98.186
2	7.607	36973	3540	2.163	1.814
Total		1709573	195145	100.000	100.000

Figure S129. HPLC spectrum of **6j**

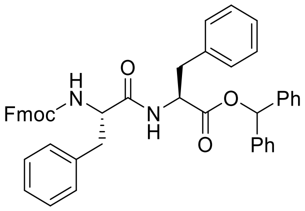

(**6k**)

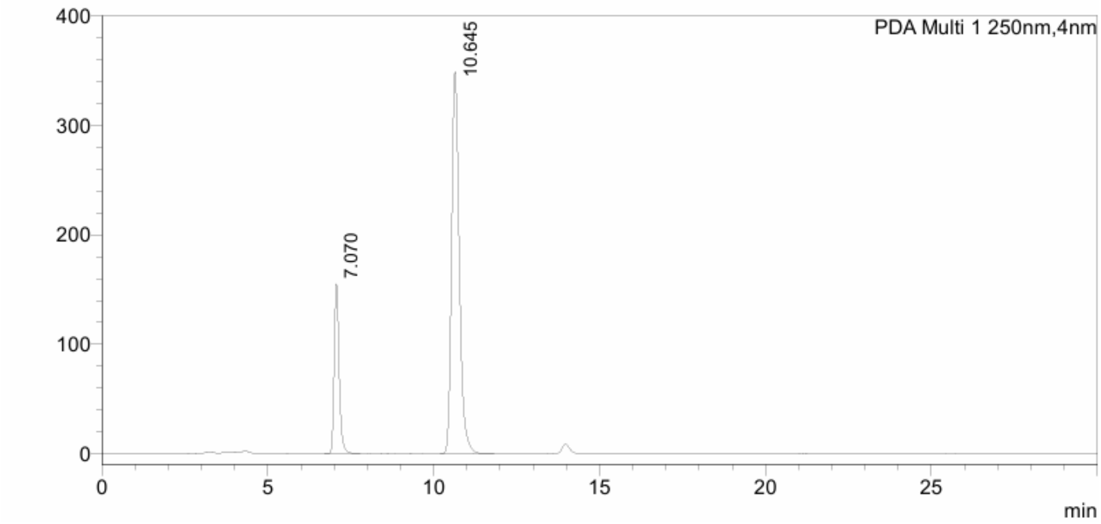

<Peak Table>

PDA Ch1 250nm

Peak#	Ret. Time	Area	Height	Area%	Height%
1	7.070	1613217	155301	22.278	30.807
2	10.645	5628221	348807	77.722	69.193
Total		7241438	504108	100.000	100.000

Figure S130. HPLC spectrum of racemic **6k**

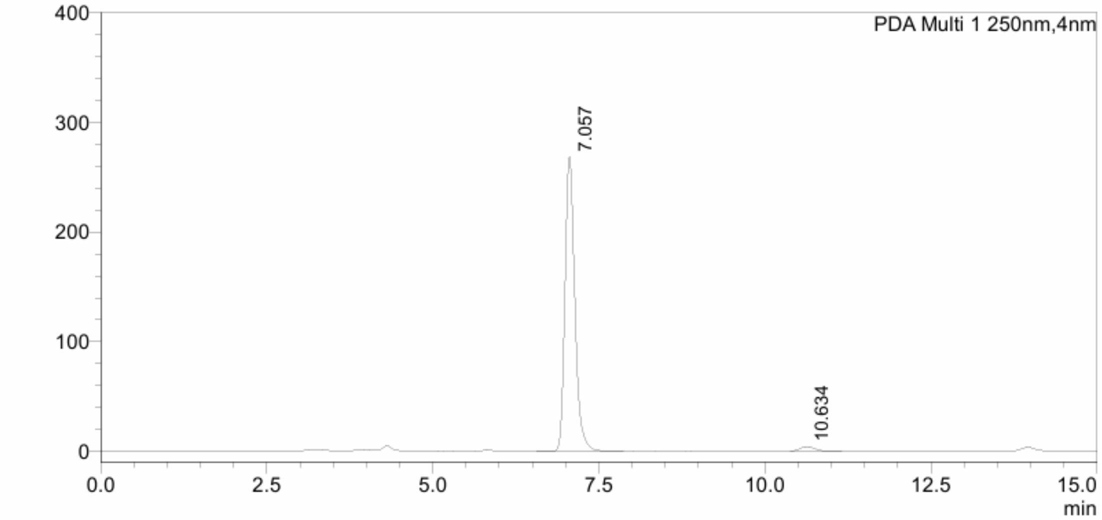

<Peak Table>

PDA Ch1 250nm

Peak#	Ret. Time	Area	Height	Area%	Height%
1	7.057	2776951	268553	97.690	98.439
2	10.634	65678	4259	2.310	1.561
Total		2842629	272812	100.000	100.000

Figure S131. HPLC spectrum of **6k**

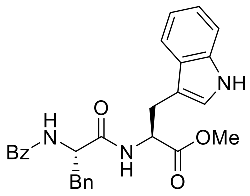

(**8b**)

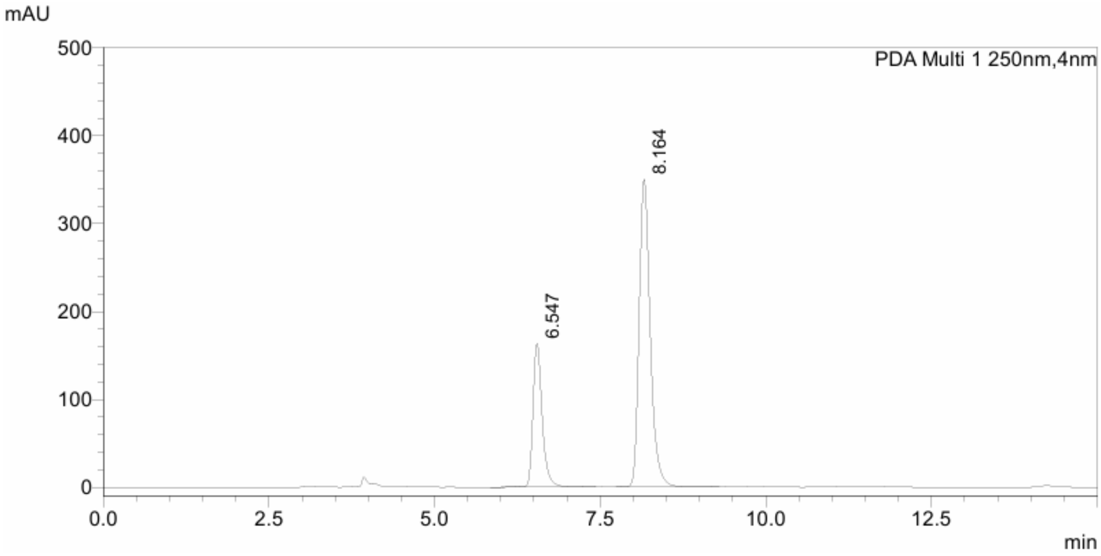

<Peak Table>

PDA Ch1 250nm

Peak#	Ret. Time	Area	Height	Area%	Height%
1	6.547	1572444	162928	27.834	31.789
2	8.164	4076876	349602	72.166	68.211
Total		5649320	512529	100.000	100.000

Figure S132. HPLC spectrum of racemic **8b**

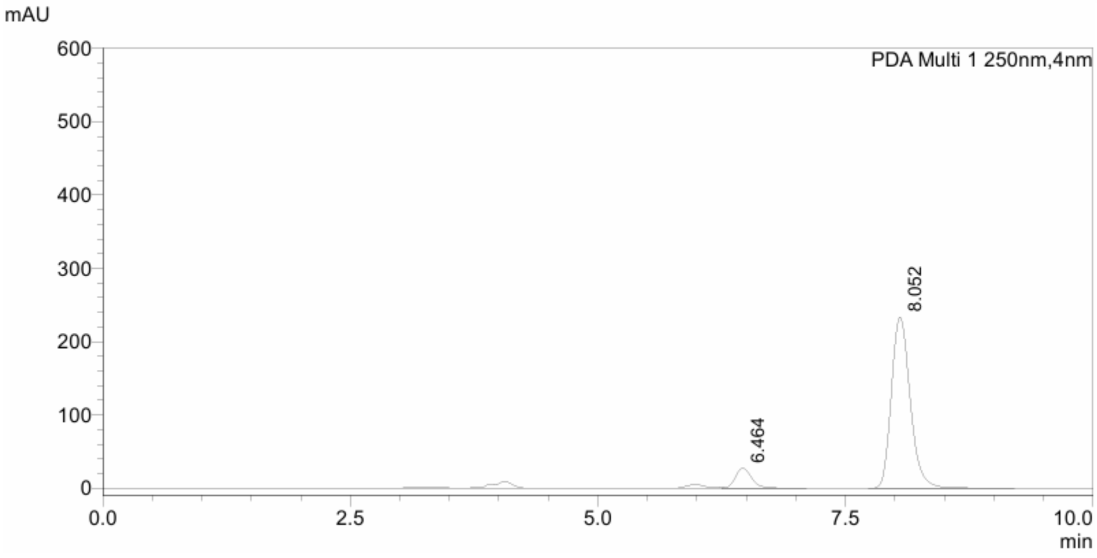

<Peak Table>

PDA Ch1 250nm

Peak#	Ret. Time	Area	Height	Area%	Height%
1	6.464	305237	26966	9.015	10.357
2	8.052	3080790	233394	90.985	89.643
Total		3386027	260360	100.000	100.000

Figure S133. HPLC spectrum of **8b**

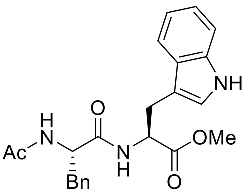

(**8c**)

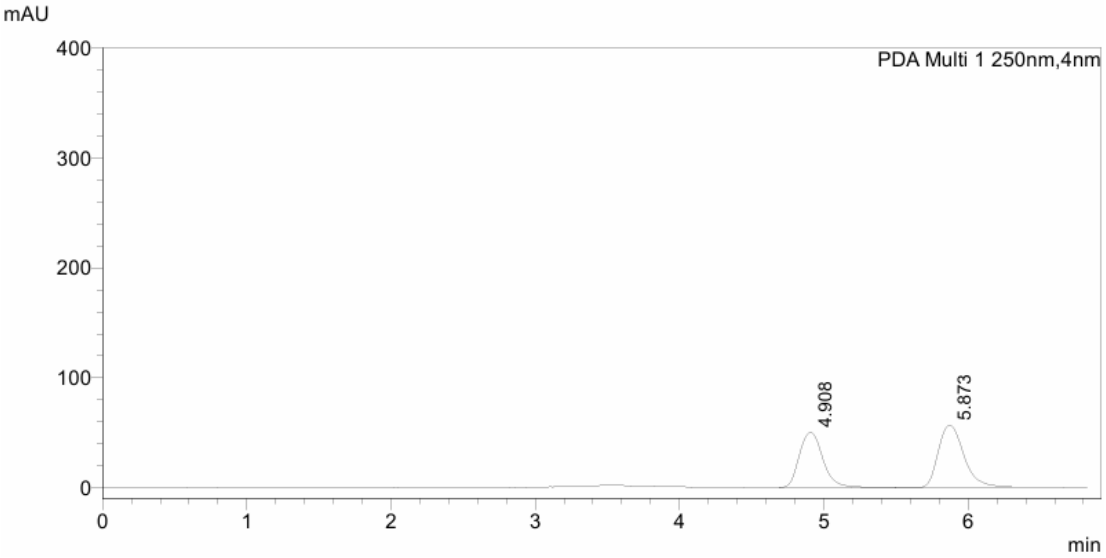

<Peak Table>

PDA Ch1 250nm

Peak#	Ret. Time	Area	Height	Area%	Height%
1	4.908	578417	50233	44.856	47.065
2	5.873	711076	56498	55.144	52.935
Total		1289493	106731	100.000	100.000

Figure S134. HPLC spectrum of racemic **8c**

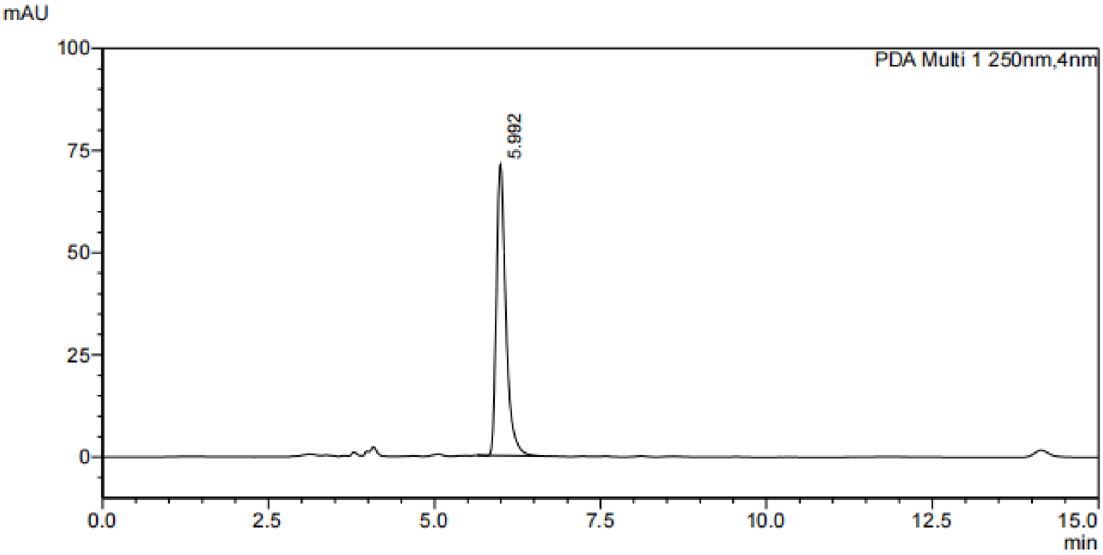

<Peak Table>

PDA Ch1 250nm

Peak#	Ret. Time	Area	Height	Area%	Height%
1	5.992	712240	71219	100.000	100.000
Total		712240	71219	100.000	100.000

Figure S135. HPLC spectrum of **8c**

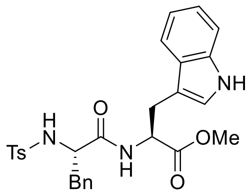

(**8d**)

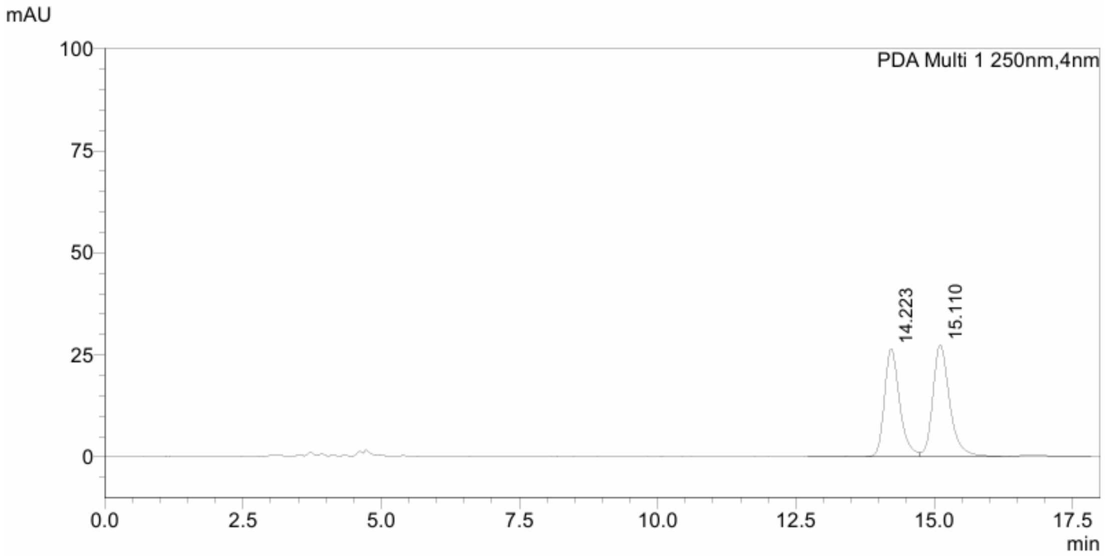

<Peak Table>

PDA Ch1 250nm

Peak#	Ret. Time	Area	Height	Area%	Height%
1	14.223	498952	26319	46.560	49.104
2	15.110	572687	27280	53.440	50.896
Total		1071639	53599	100.000	100.000

Figure S136. HPLC spectrum of racemic **8d**

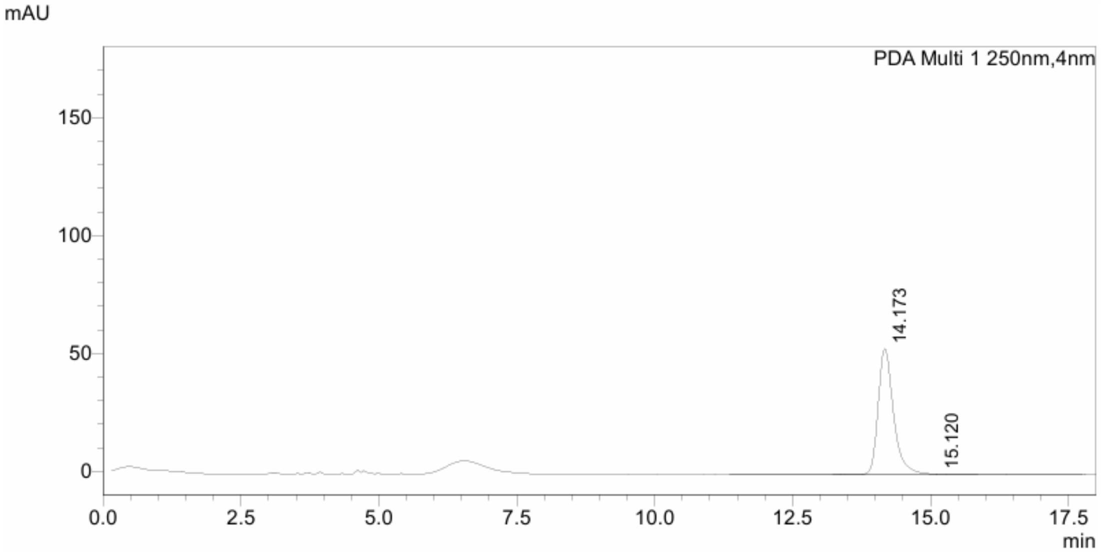

<Peak Table>

PDA Ch1 250nm

Peak#	Ret. Time	Area	Height	Area%	Height%
1	14.173	1019828	53198	99.817	99.735
2	15.120	1875	141	0.183	0.265
Total		1021703	53340	100.000	100.000

Figure S137. HPLC spectrum of **8d**

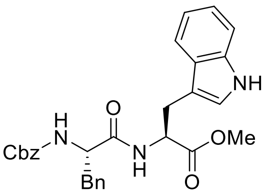

(**8e**)

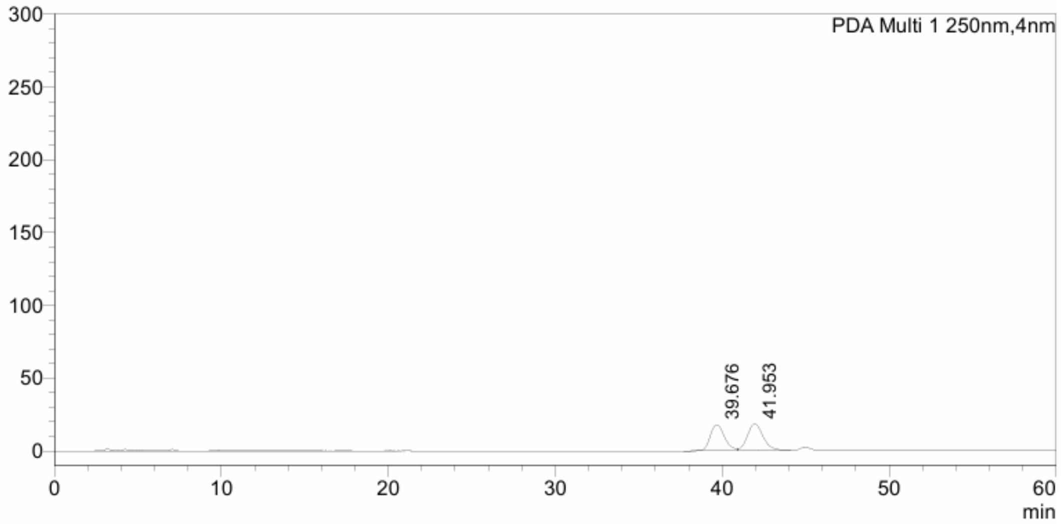

<Peak Table>

PDA Ch1 250nm

Peak#	Ret. Time	Area	Height	Area%	Height%
1	39.676	1064516	17798	46.735	49.449
2	41.953	1213233	18194	53.265	50.551
Total		2277748	35992	100.000	100.000

Figure S138. HPLC spectrum of racemic **8e**

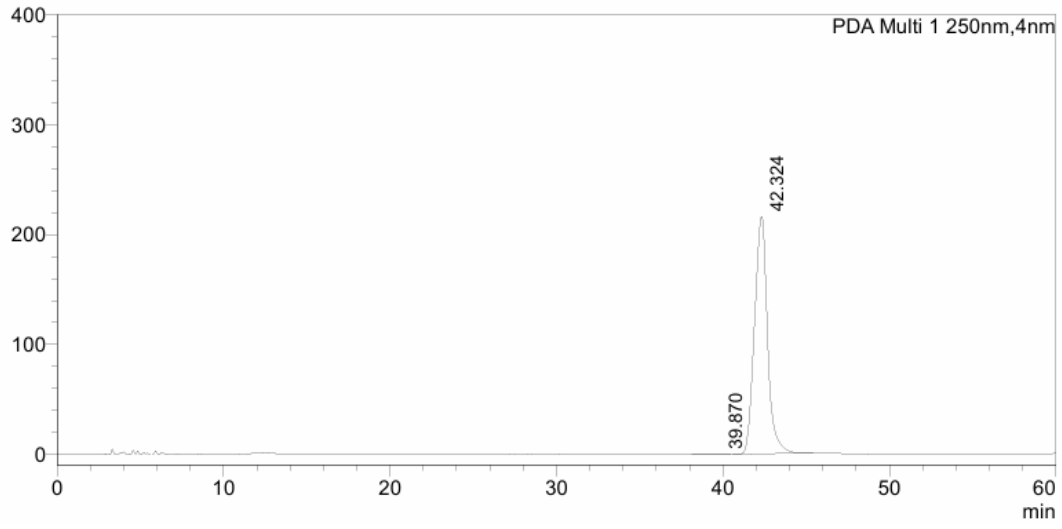

<Peak Table>

PDA Ch1 250nm

Peak#	Ret. Time	Area	Height	Area%	Height%
1	39.870	-1329	41	-0.011	0.019
2	42.324	11710384	216039	100.011	99.981
Total		11709056	216080	100.000	100.000

Figure S139. HPLC spectrum of **8e**

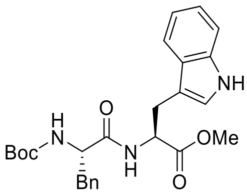

(**8f**)

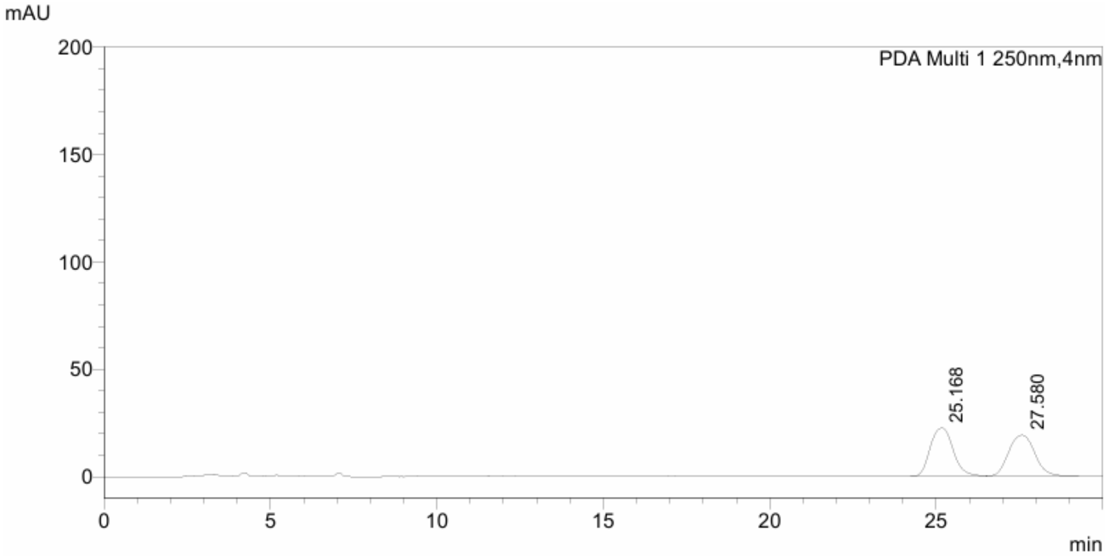

<Peak Table>

PDA Ch1 250nm

Peak#	Ret. Time	Area	Height	Area%	Height%
1	25.168	1087464	22475	50.618	53.960
2	27.580	1060895	19177	49.382	46.040
Total		2148359	41652	100.000	100.000

Figure S140. HPLC spectrum of racemic **8f**

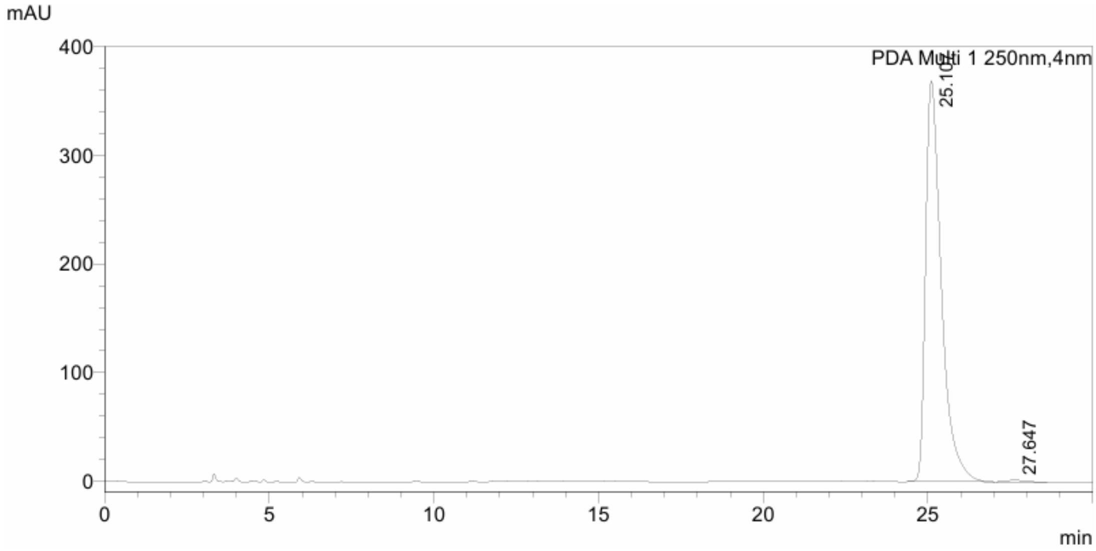

<Peak Table>

PDA Ch1 250nm

Peak#	Ret. Time	Area	Height	Area%	Height%
1	25.107	12241191	369021	99.549	99.577
2	27.647	55505	1567	0.451	0.423
Total		12296697	370588	100.000	100.000

Figure S141. HPLC spectrum of **8f**

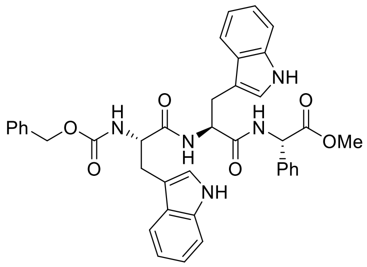

(8g)

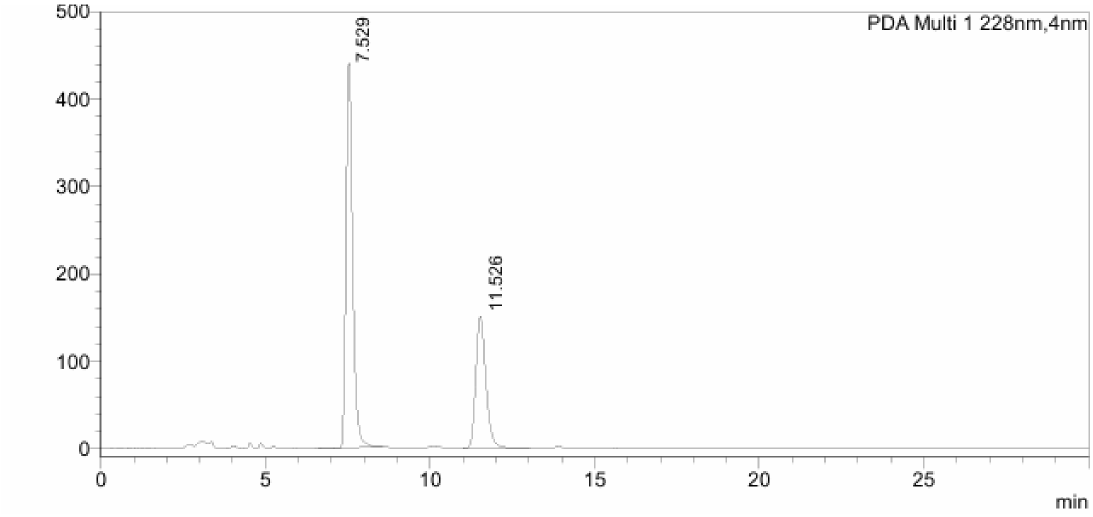

<Peak Table>

PDA Ch1 228nm

Peak#	Ret. Time	Area	Height	Area%	Height%
1	7.529	6104056	439729	65.363	74.413
2	11.526	3234622	151200	34.637	25.587
Total		9338678	590928	100.000	100.000

Figure S142. HPLC spectrum of racemic **8g**

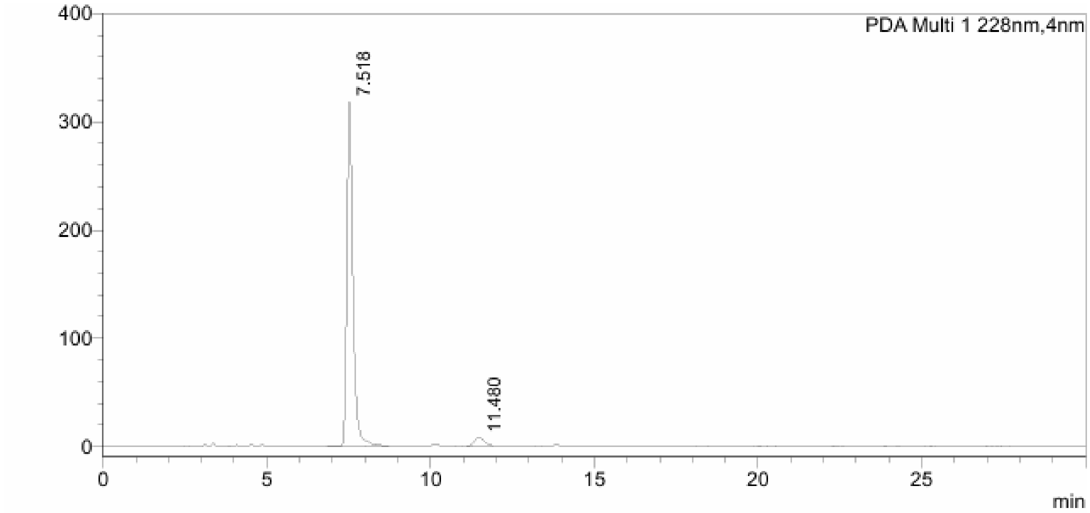

<Peak Table>

PDA Ch1 228nm

Peak#	Ret. Time	Area	Height	Area%	Height%
1	7.518	4122623	318908	96.453	97.574
2	11.480	151594	7929	3.547	2.426
Total		4274217	326837	100.000	100.000

Figure S143. HPLC spectrum of **8g**

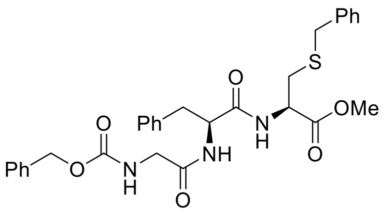

(**8h**)

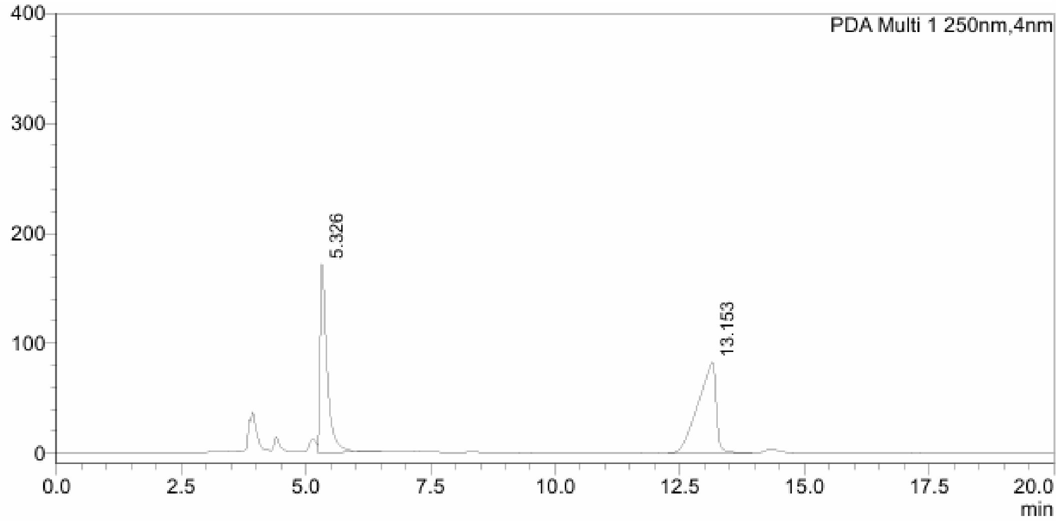

<Peak Table>

PDA Ch1 250nm

Peak#	Ret. Time	Area	Height	Area%	Height%
1	5.326	1646542	171194	44.476	67.554
2	13.153	2055566	82223	55.524	32.446
Total		3702108	253418	100.000	100.000

Figure S144. HPLC spectrum of racemic **8h**

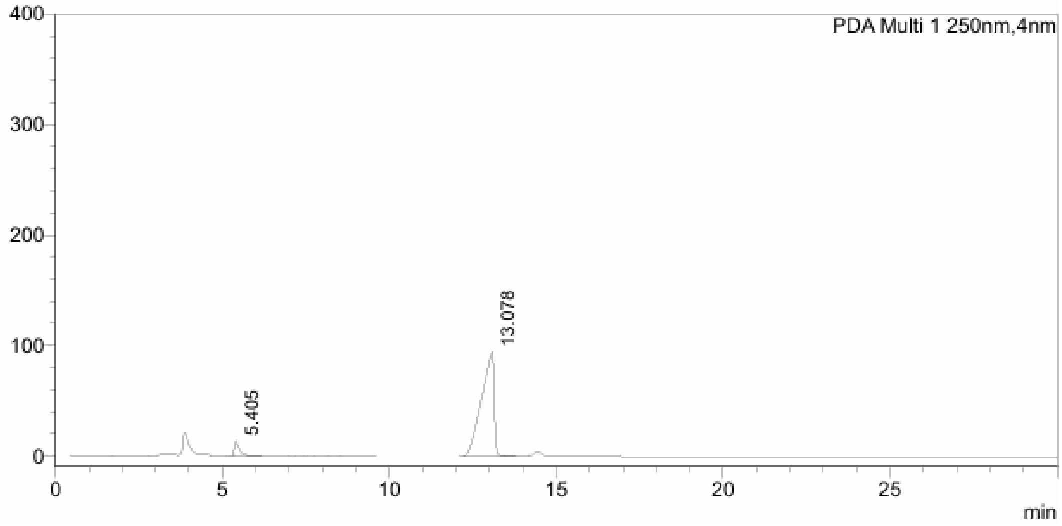

<Peak Table>

PDA Ch1 250nm

Peak#	Ret. Time	Area	Height	Area%	Height%
1	5.405	137014	13378	5.198	12.369
2	13.078	2498991	94778	94.802	87.631
Total		2636005	108157	100.000	100.000

Figure S145. HPLC spectrum of **8h**

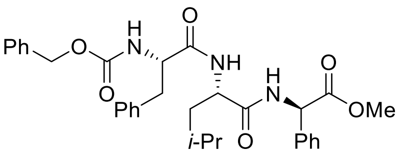

(8i)

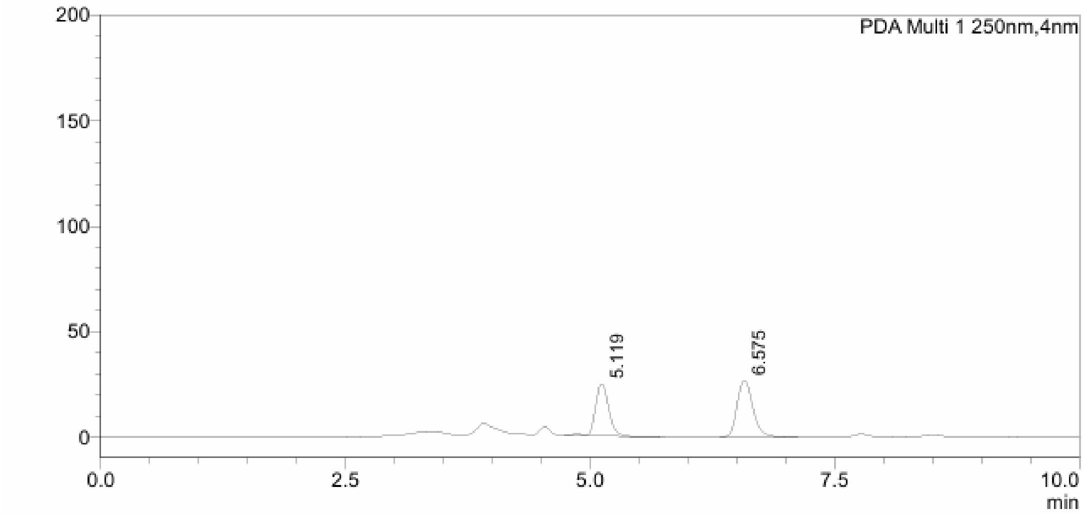

<Peak Table>

PDA Ch1 250nm

Peak#	Ret. Time	Area	Height	Area%	Height%
1	5.119	233054	24687	44.060	47.945
2	6.575	295891	26803	55.940	52.055
Total		528946	51490	100.000	100.000

Figure S146. HPLC spectrum of racemic **8i**

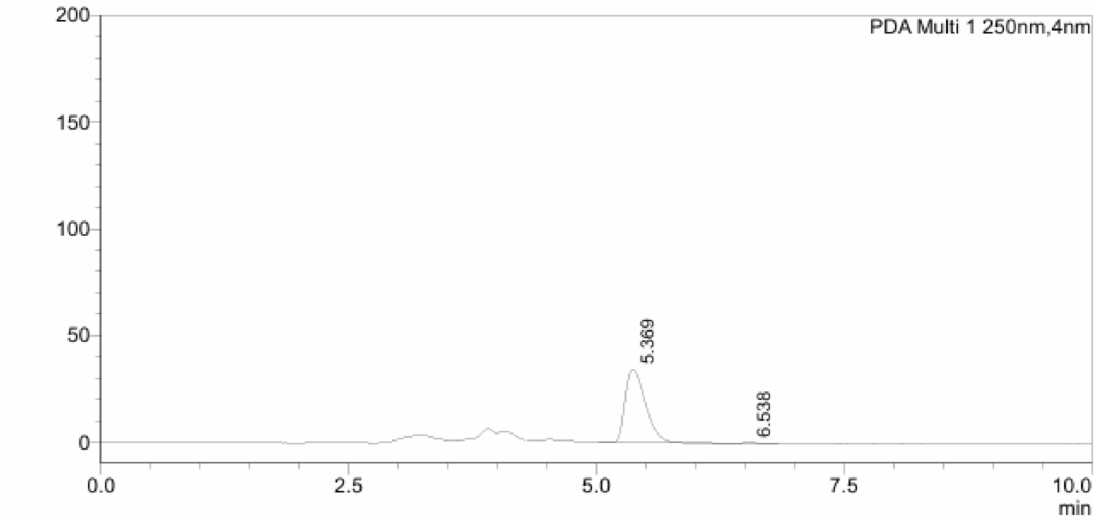

<Peak Table>

PDA Ch1 250nm

Peak#	Ret. Time	Area	Height	Area%	Height%
1	5.369	478799	34096	99.541	99.013
2	6.538	2209	340	0.459	0.987
Total		481009	34436	100.000	100.000

Figure S147. HPLC spectrum of **8i**

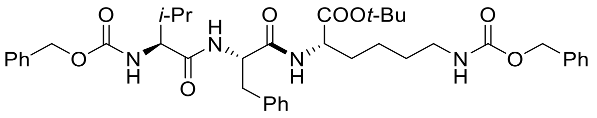

(8j)

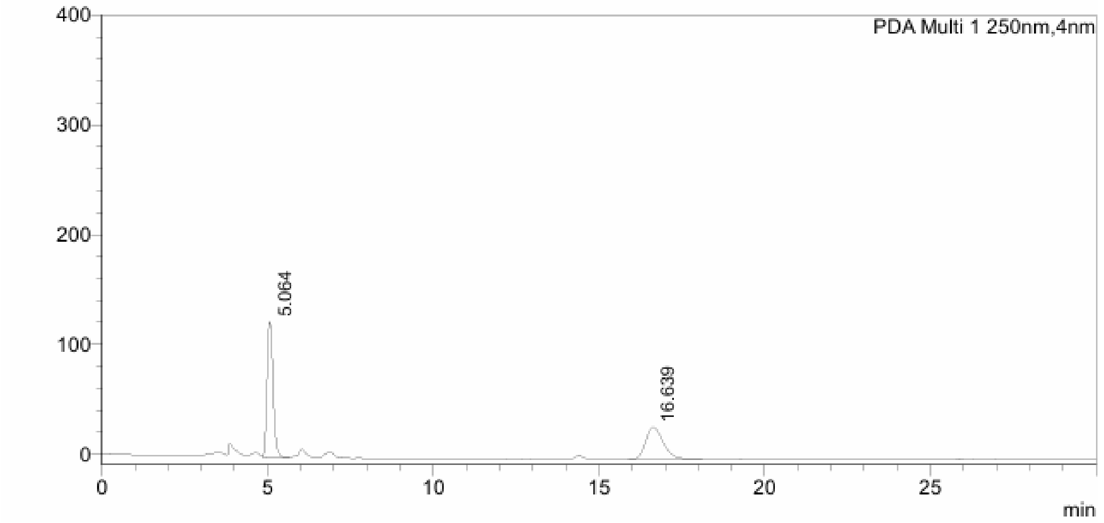

<Peak Table>

PDA Ch1 250nm

Peak#	Ret. Time	Area	Height	Area%	Height%
1	5.064	1546529	123261	59.411	80.979
2	16.639	1056590	28952	40.589	19.021
Total		2603119	152212	100.000	100.000

Figure S148. HPLC spectrum of racemic **8j**

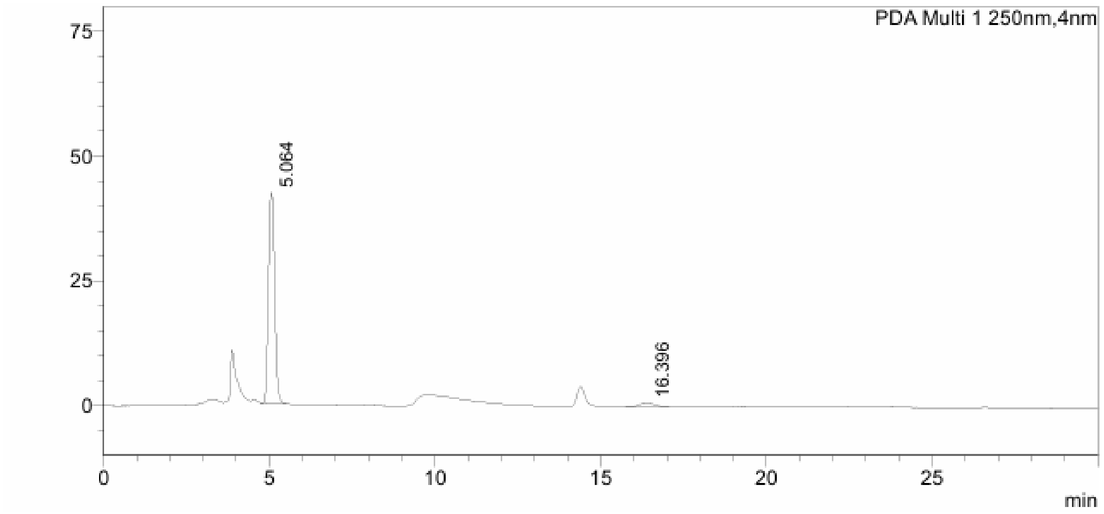

<Peak Table>

PDA Ch1 250nm

Peak#	Ret. Time	Area	Height	Area%	Height%
1	5.064	544701	42250	95.560	98.184
2	16.396	25310	781	4.440	1.816
Total		570011	43032	100.000	100.000

Figure S149. HPLC spectrum of **8j**

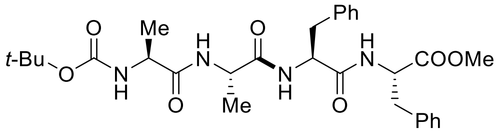

(8k)

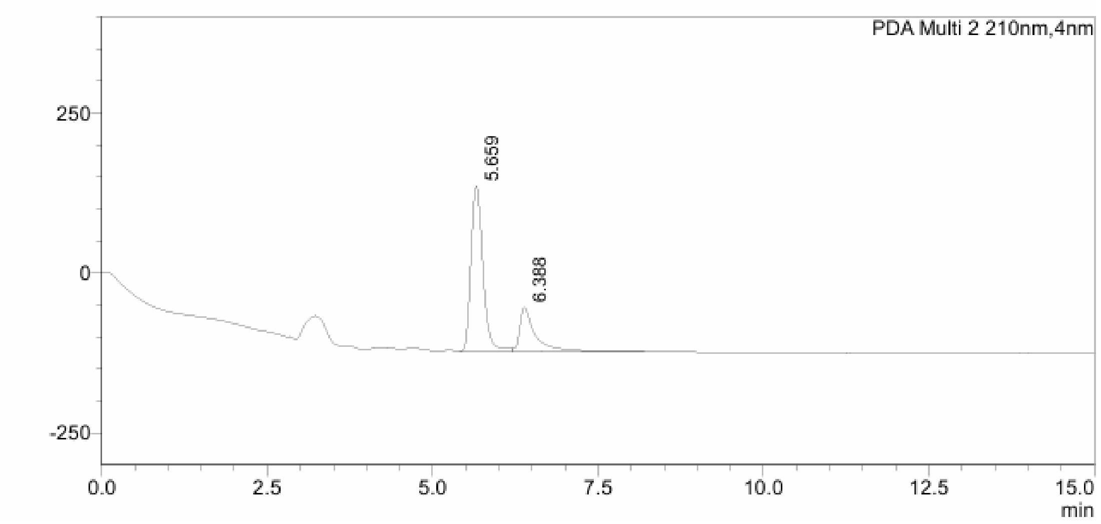

<Peak Table>

PDA Ch2 210nm

Peak#	Ret. Time	Area	Height	Area%	Height%
1	5.659	3259308	256781	74.546	78.989
2	6.388	1112908	68306	25.454	21.011
Total		4372216	325087	100.000	100.000

Figure S150. HPLC spectrum of racemic **8k**

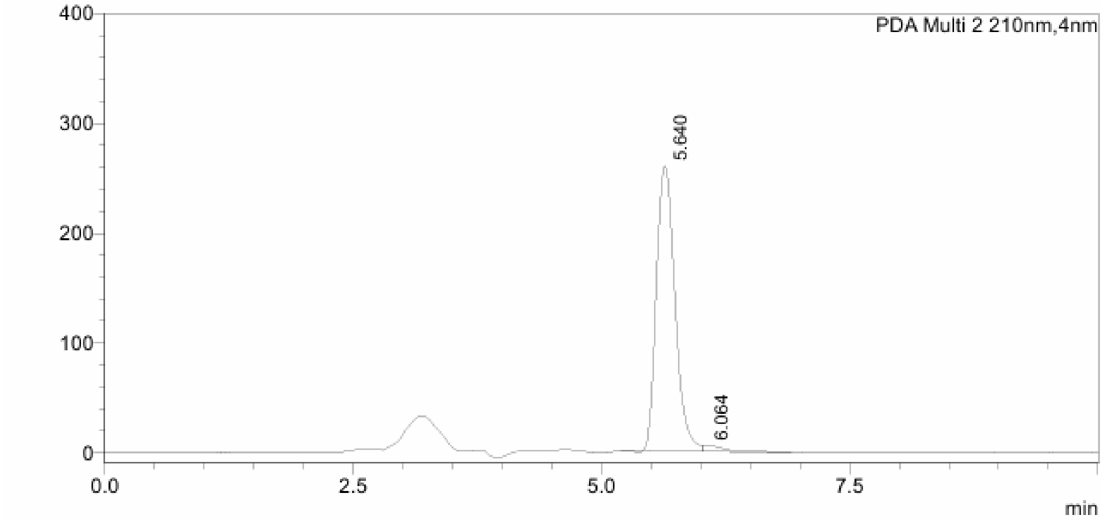

<Peak Table>

PDA Ch2 210nm

Peak#	Ret. Time	Area	Height	Area%	Height%
1	5.640	3382346	259930	97.667	97.936
2	6.064	80810	5479	2.333	2.064
Total		3463156	265409	100.000	100.000

Figure S151. HPLC spectrum of **8k**

NMR spectra

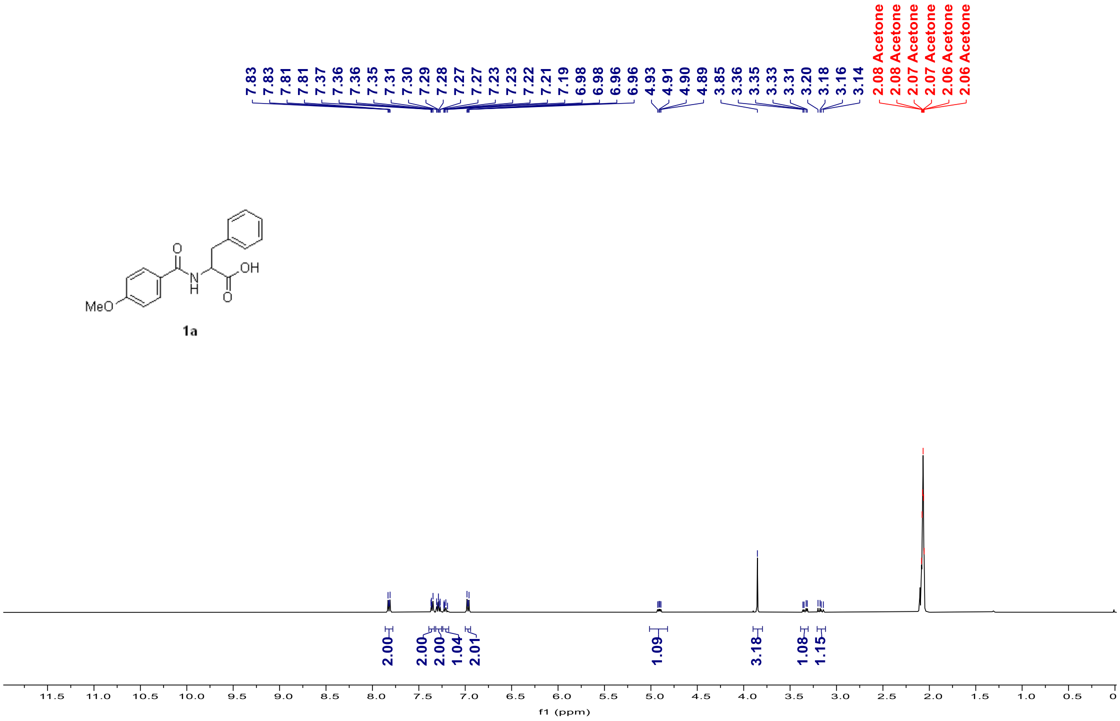

Figure S152. **^{1}H NMR** of the **1a** (400 MHz, Acetone-d_6)

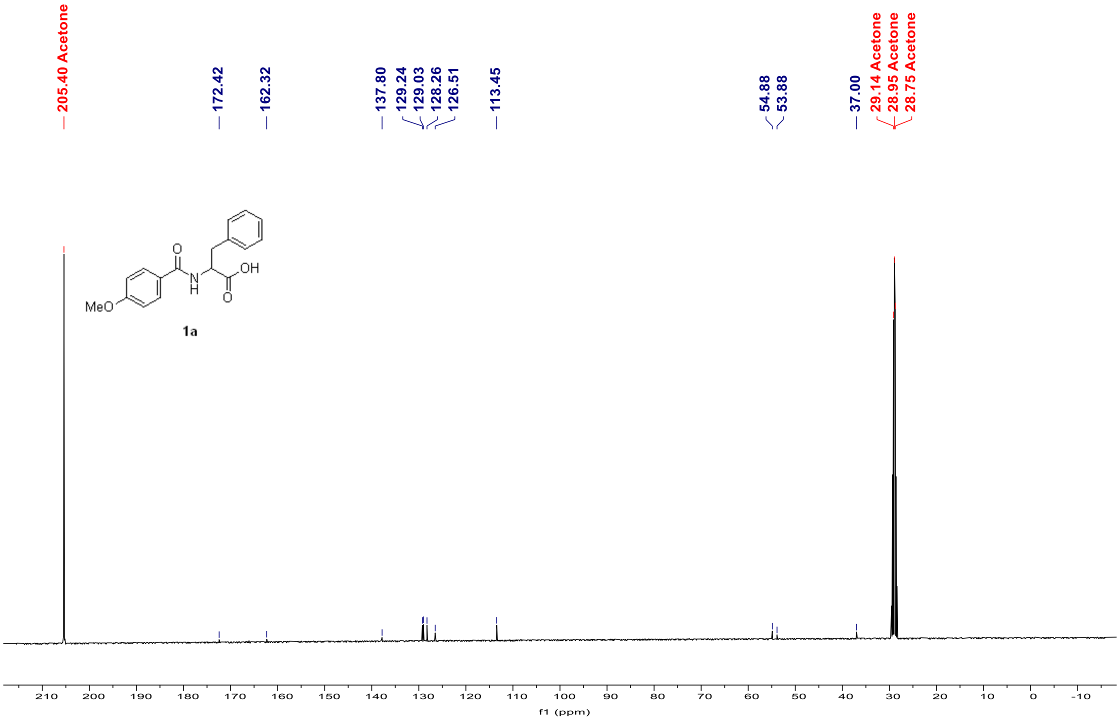

Figure S153. **^{13}C NMR** of the **1a** (101 MHz, Acetone-d_6)

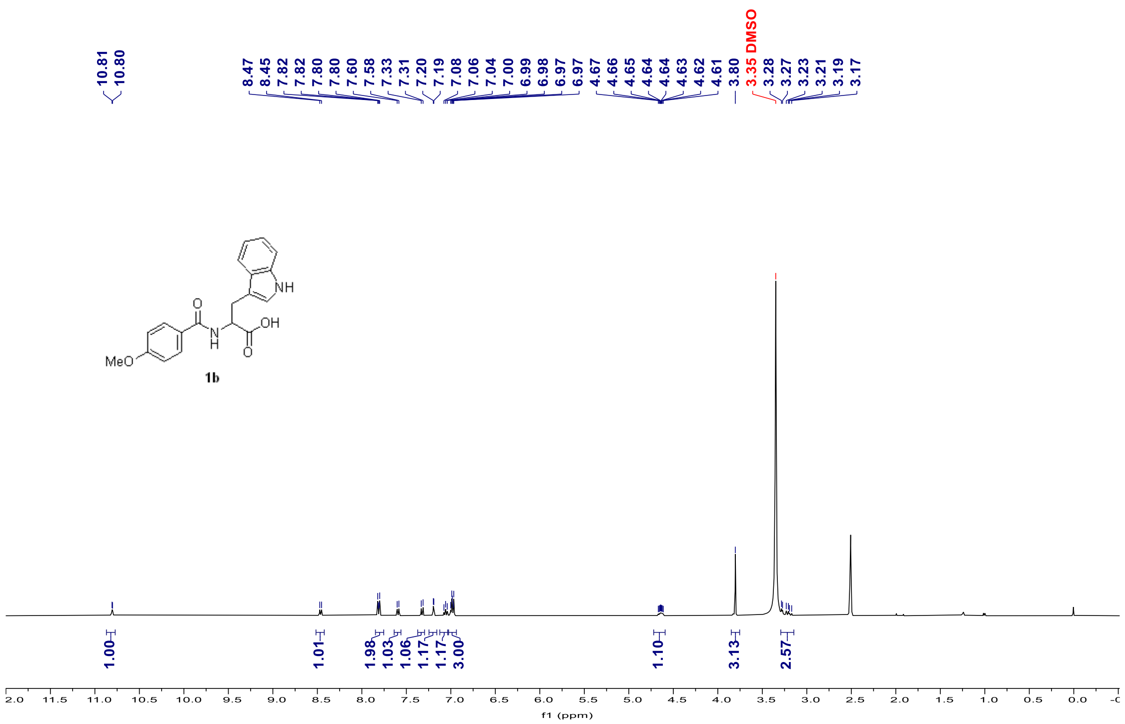

Figure S154. **^{1}H NMR** of the **1b** (400 MHz, DMSO-d_6)

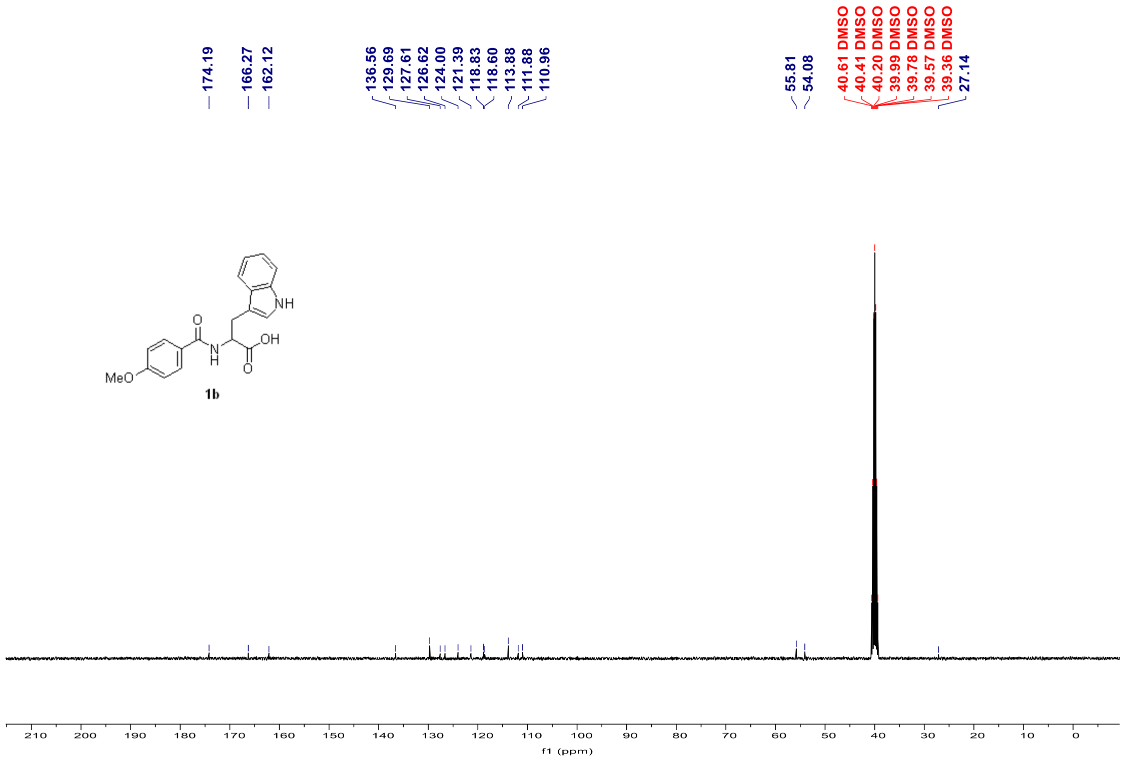

Figure S155. **^{13}C NMR** of the **1b** (101 MHz, DMSO-d_6)

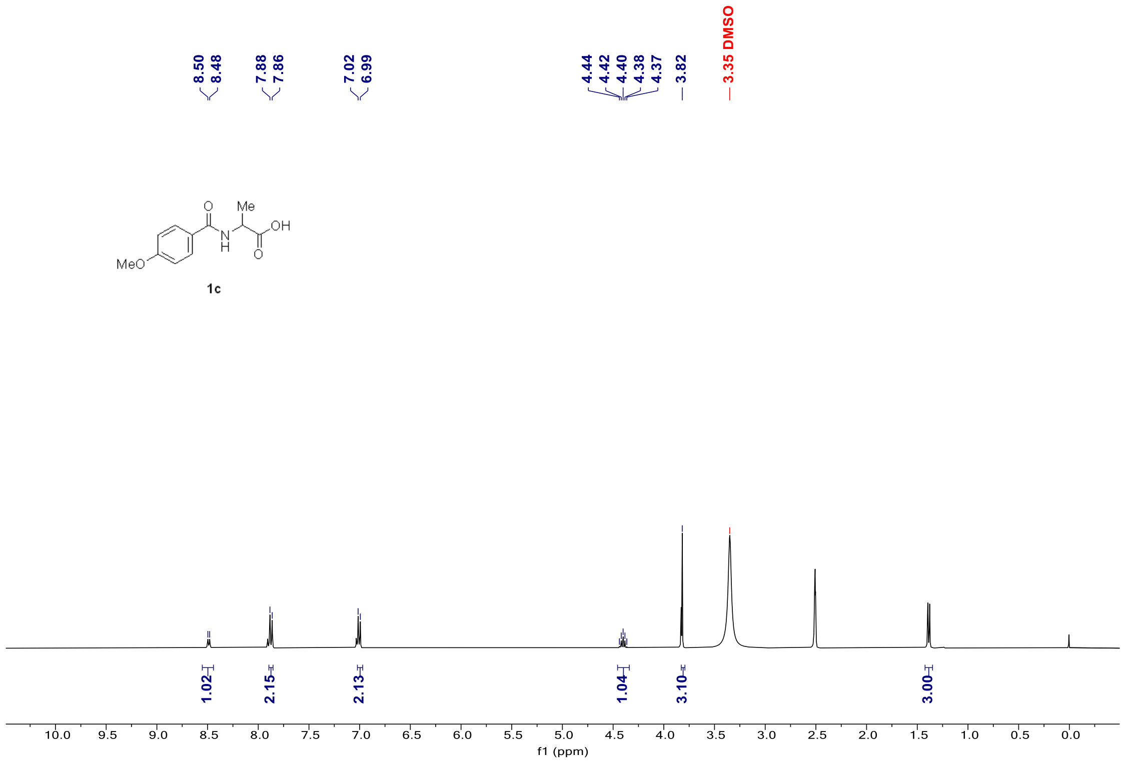

Figure S156. ¹H NMR of the **1c** (400 MHz, DMSO-d_6)

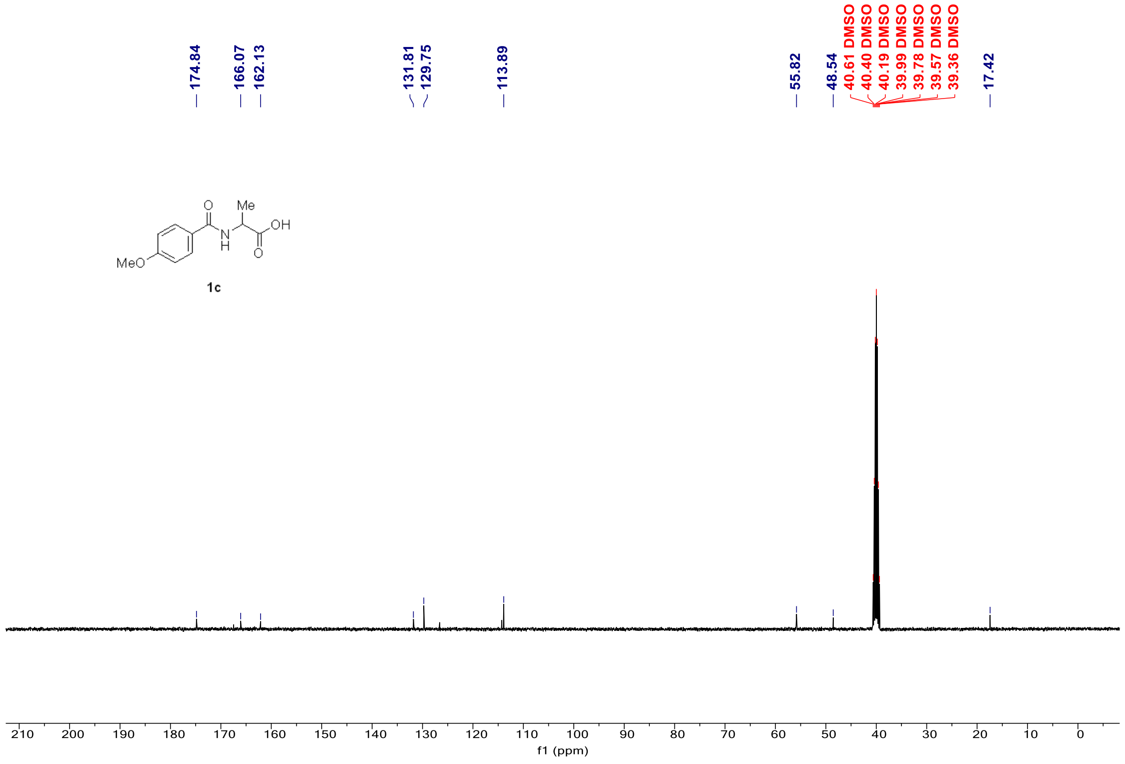

Figure S157. ¹³C NMR of the **1c** (101 MHz, DMSO-d_6)

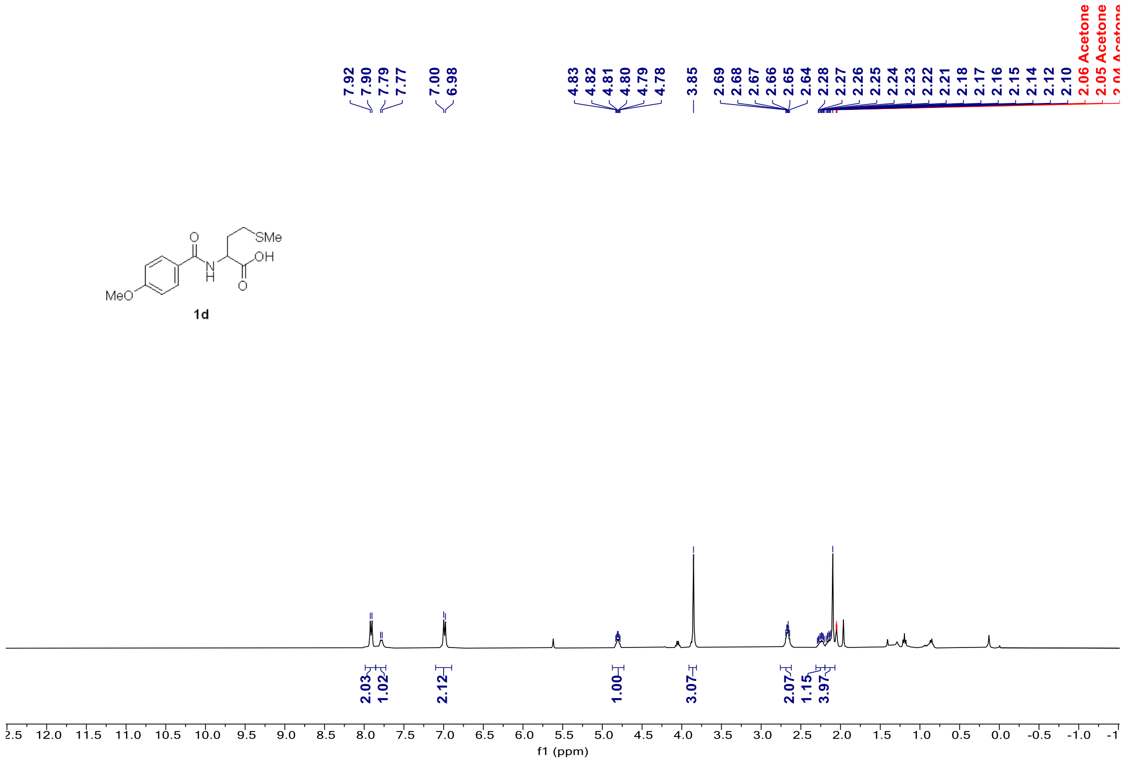

Figure S158. ^{1}H NMR of the 1d (400 MHz, Acetone-d_6)

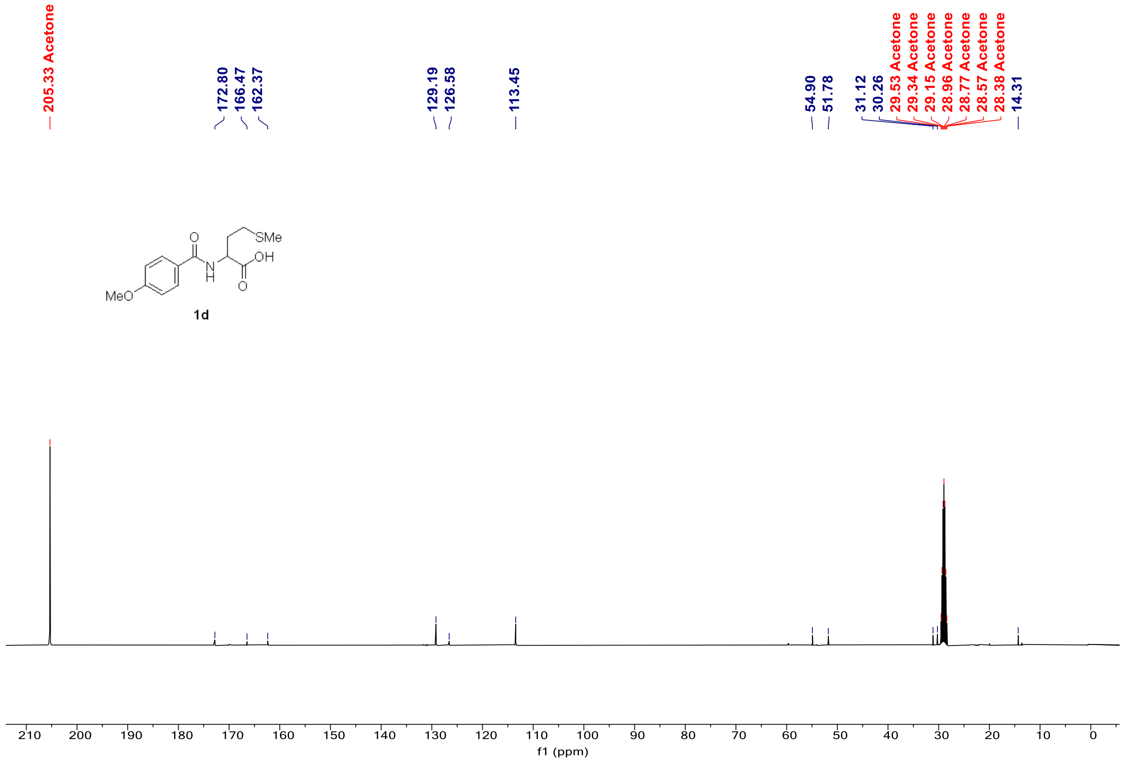

Figure S159. ^{13}C NMR of the 1d (101 MHz, Acetone-d_6)

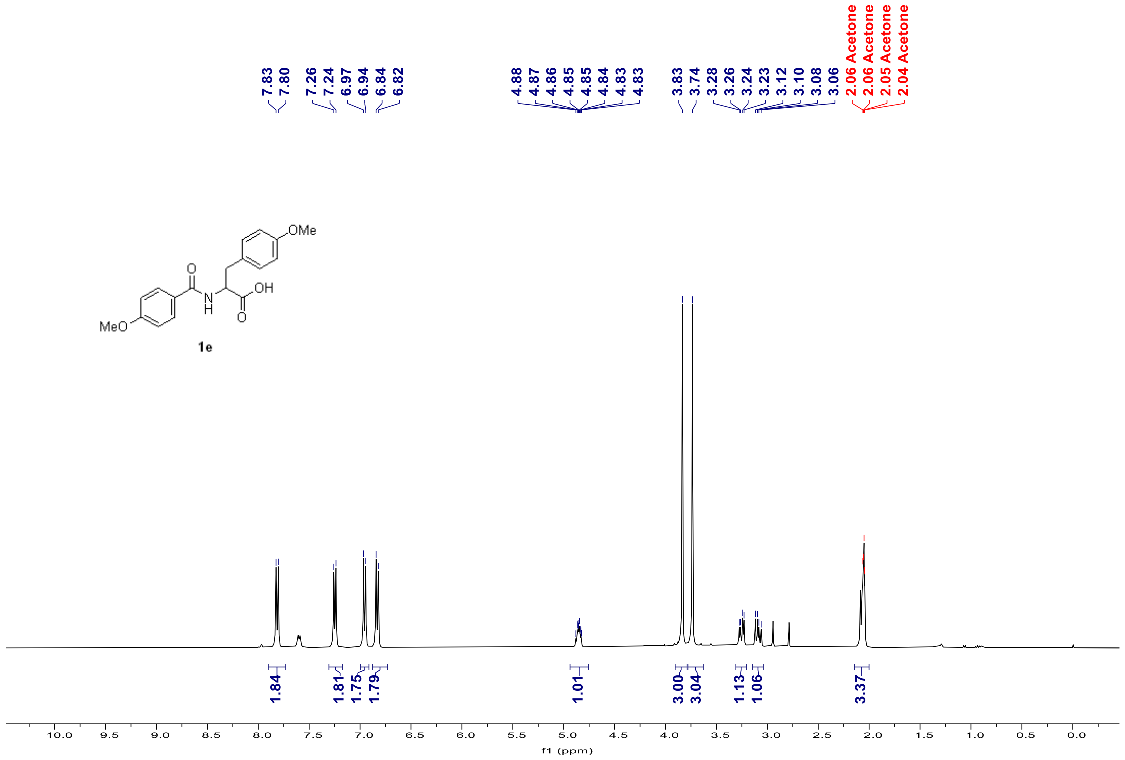

Figure S160. **^{1}H NMR** of the **1e** (400 MHz, Acetone-d_6)

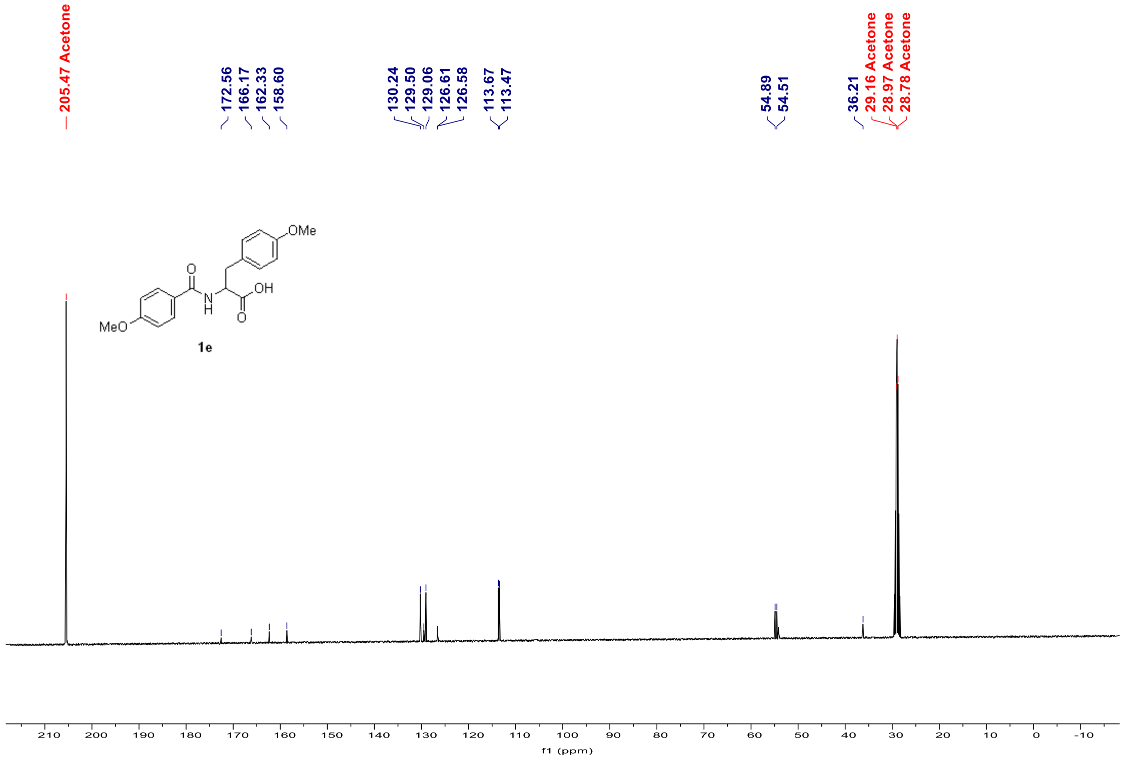

Figure S161. **^{13}C NMR** of the **1e** (101 MHz, Acetone-d_6)

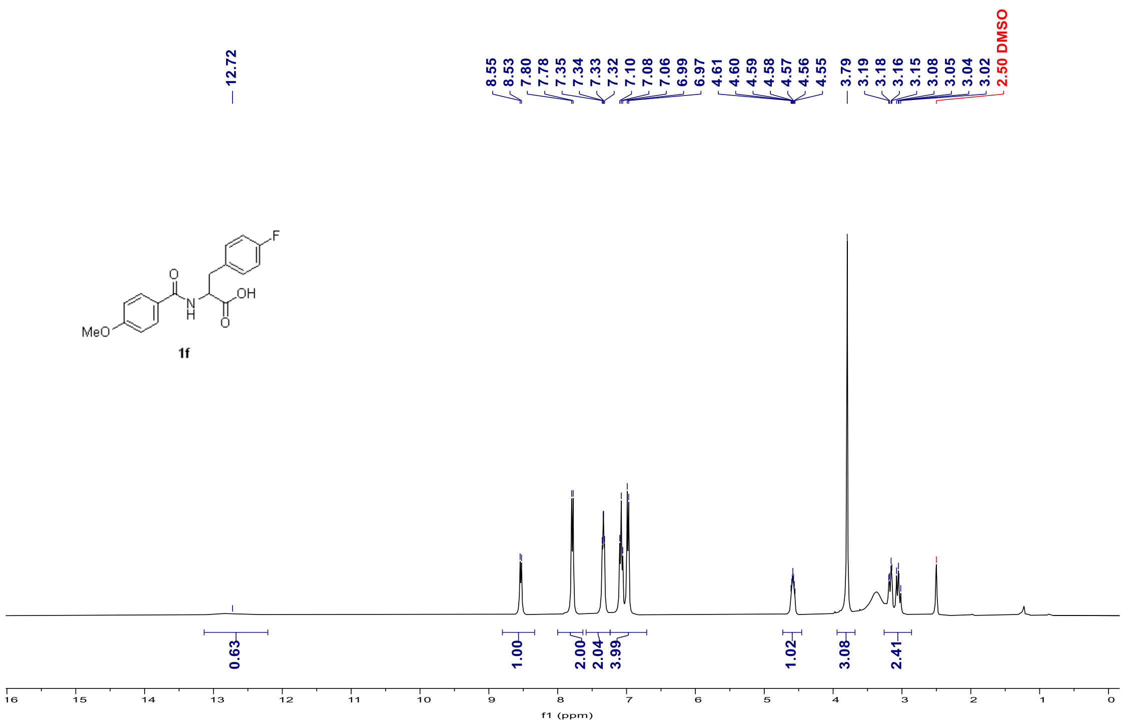

Figure S162. ^{1}**H NMR** of the **1f** (400 MHz, DMSO-d_6)

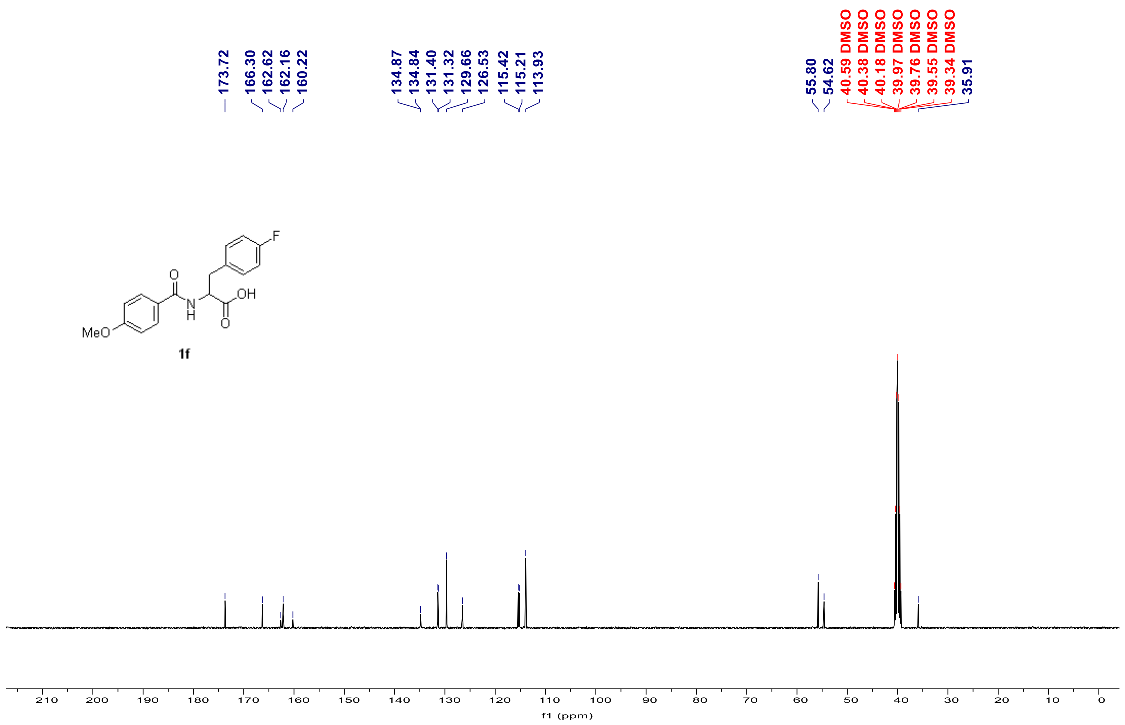

Figure S163. ^{13}**C NMR** of the **1f** (101 MHz, DMSO-d_6)

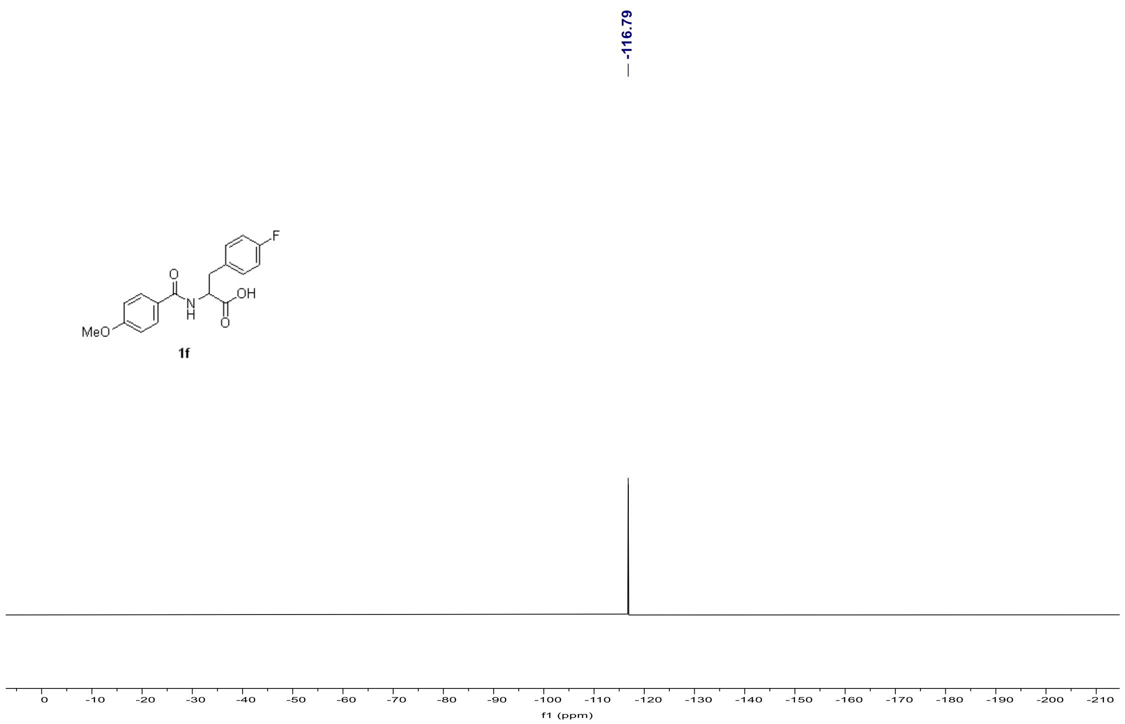

Figure S164. ^{19}F NMR of the **1f** (376 MHz, DMSO-d_6)

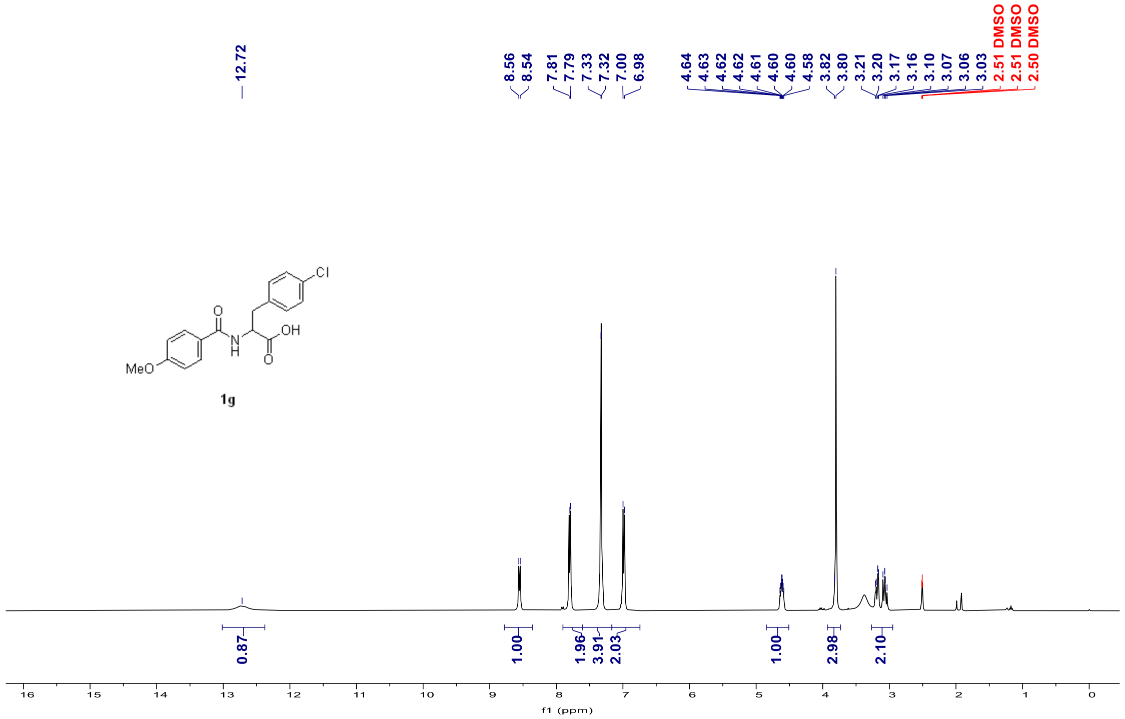

Figure S165. **^{1}H NMR** of the **1g** (400 MHz, DMSO-d_6)

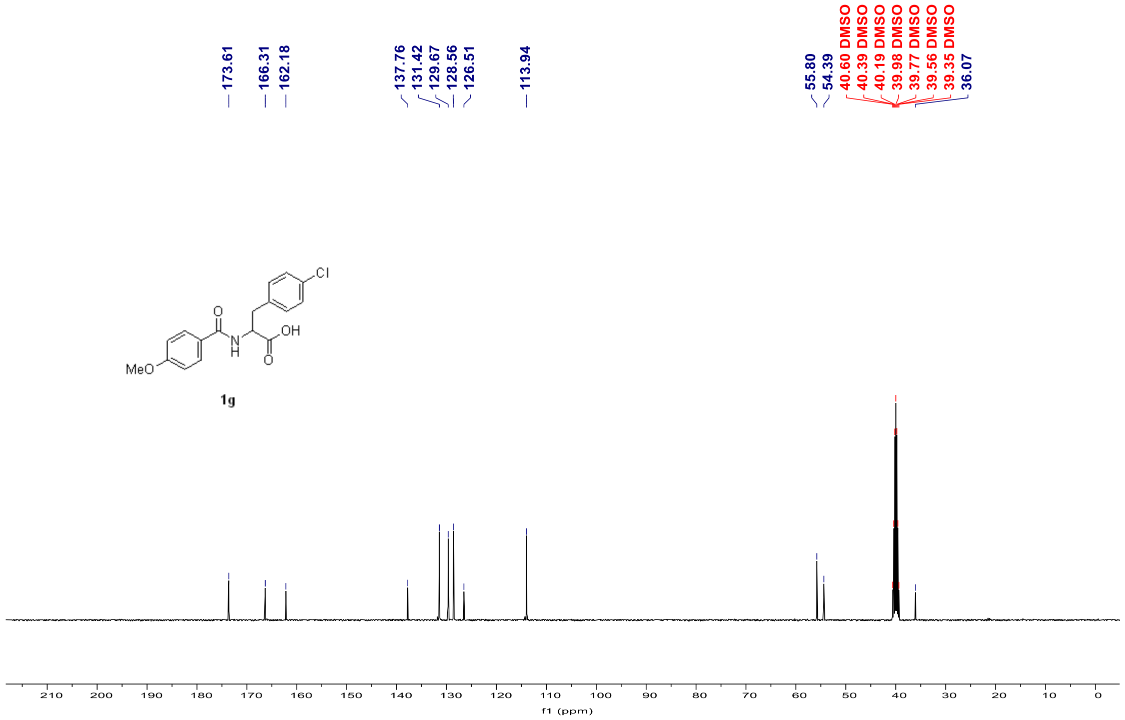

Figure S166. **^{13}C NMR** of the **1g** (101 MHz, DMSO-d_6)

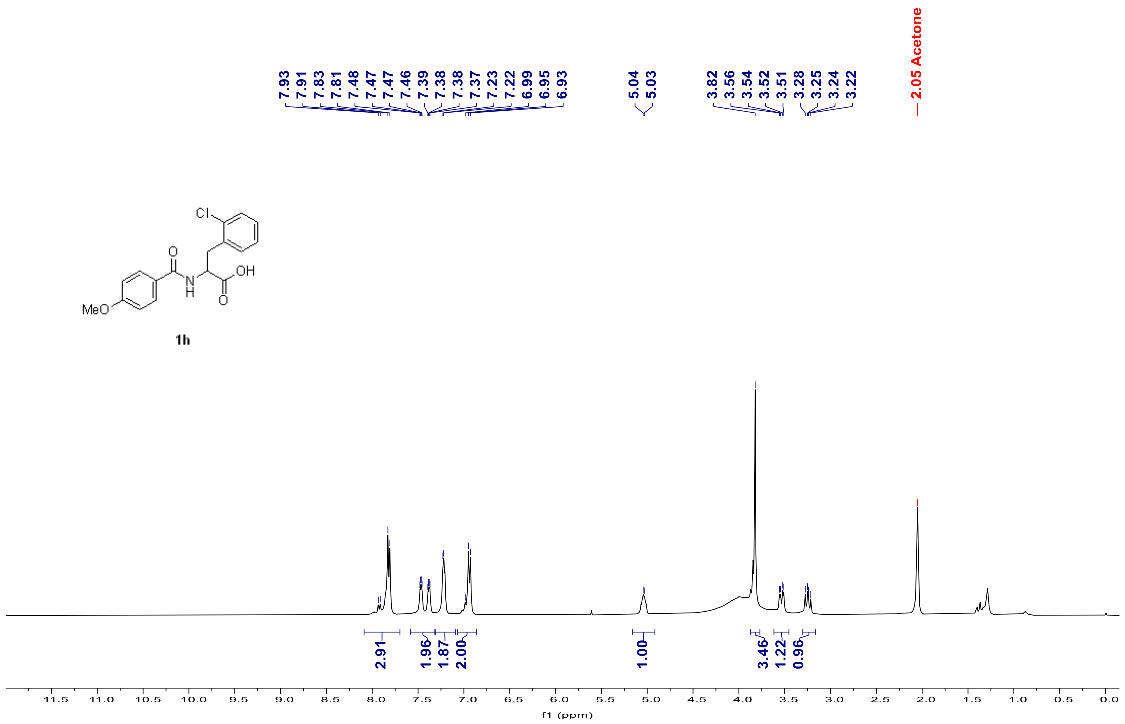

Figure S167. **^{1}H NMR** of the **1h** (400 MHz, DMSO-d_6)

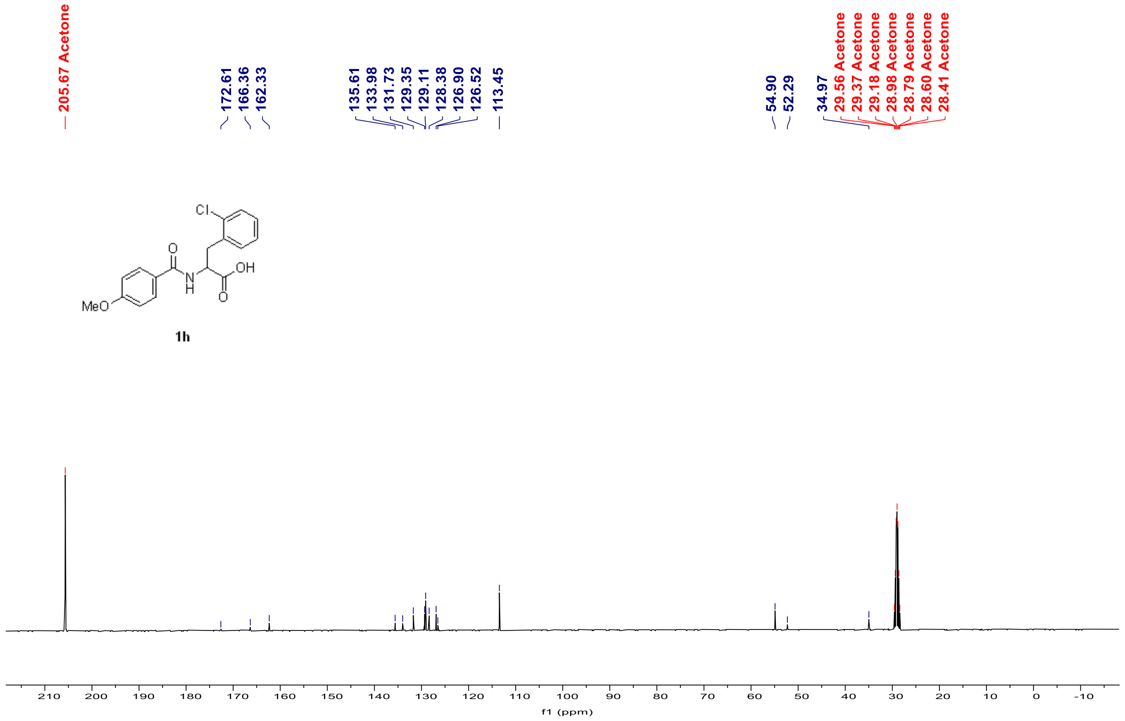

Figure S168. **^{13}C NMR** of the **1h** (101 MHz, DMSO-d_6)

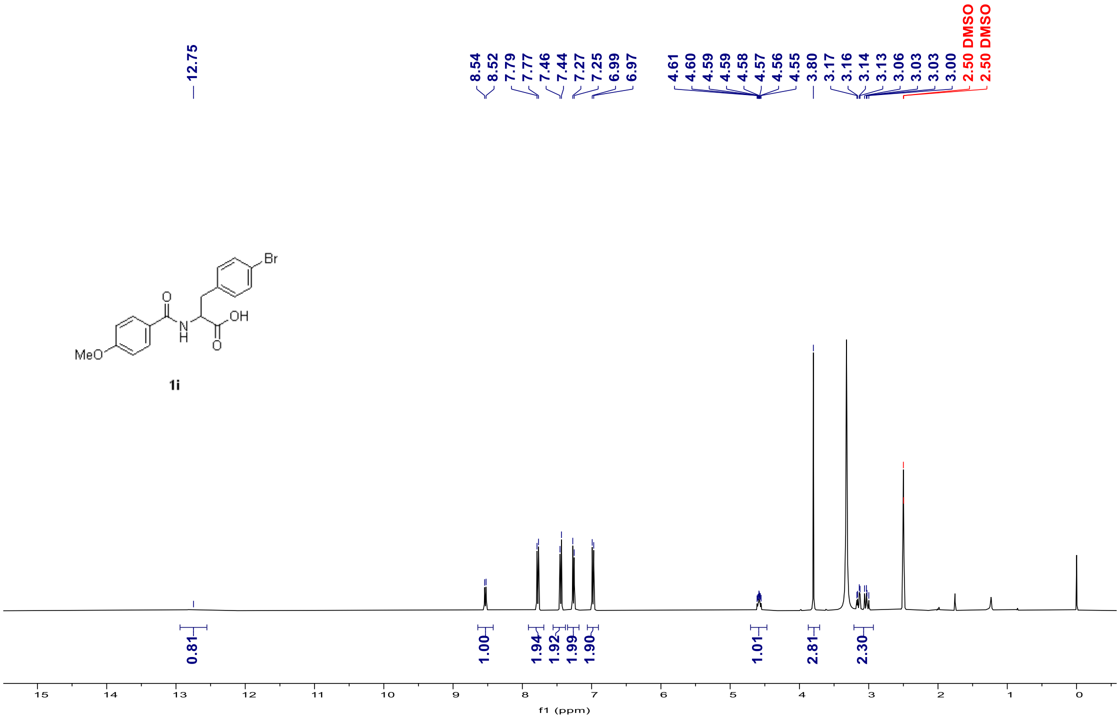

Figure S169. ^{1}**H NMR** of the **1i** (400 MHz, DMSO-d_6)

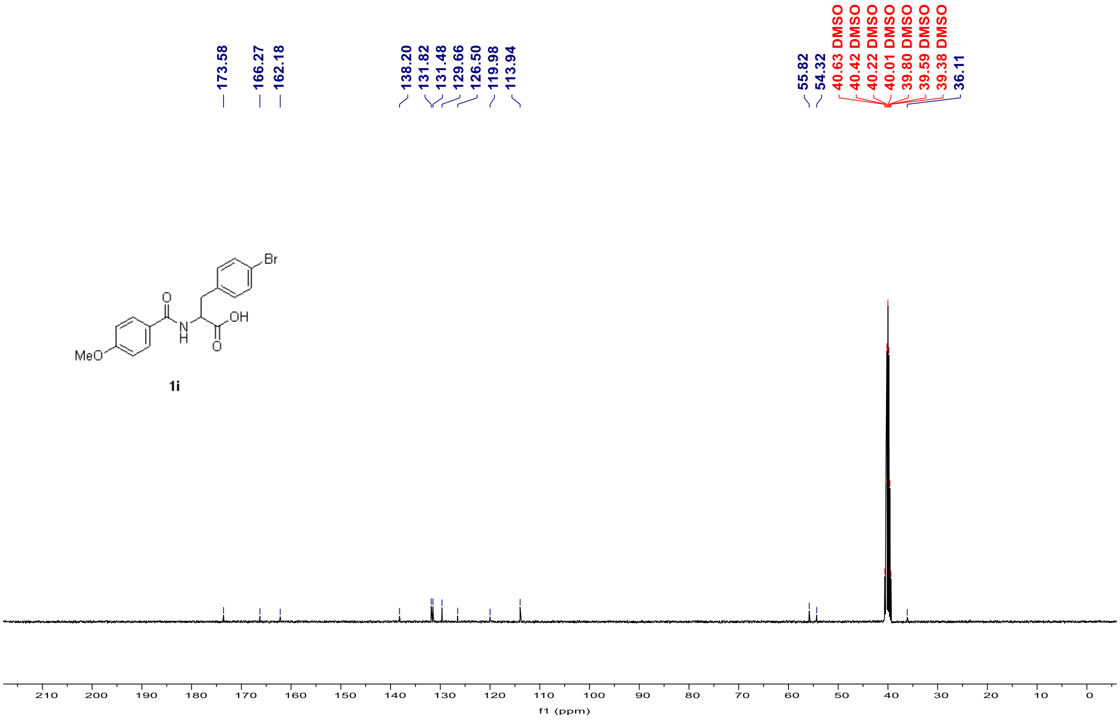

Figure S170. ^{13}**C NMR** of the **1i** (101 MHz, DMSO-d_6)

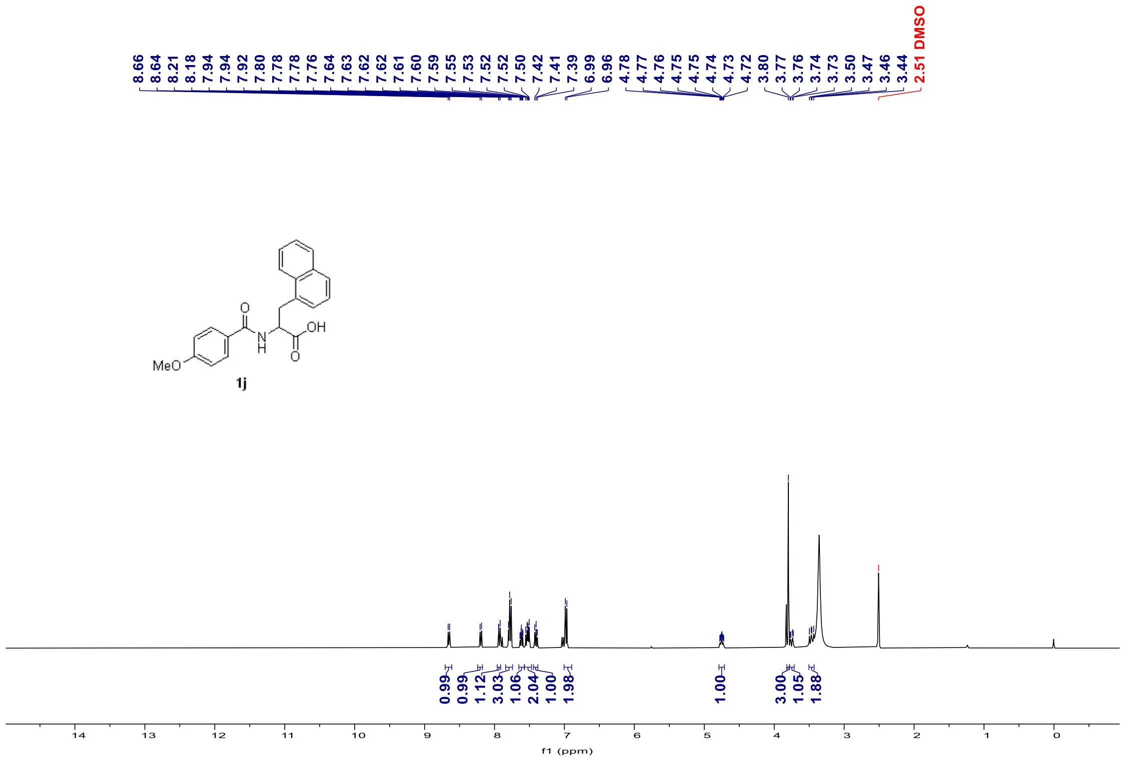

Figure S171. ^{1}**H NMR** of the **1j** (400 MHz, DMSO-d_6**)**

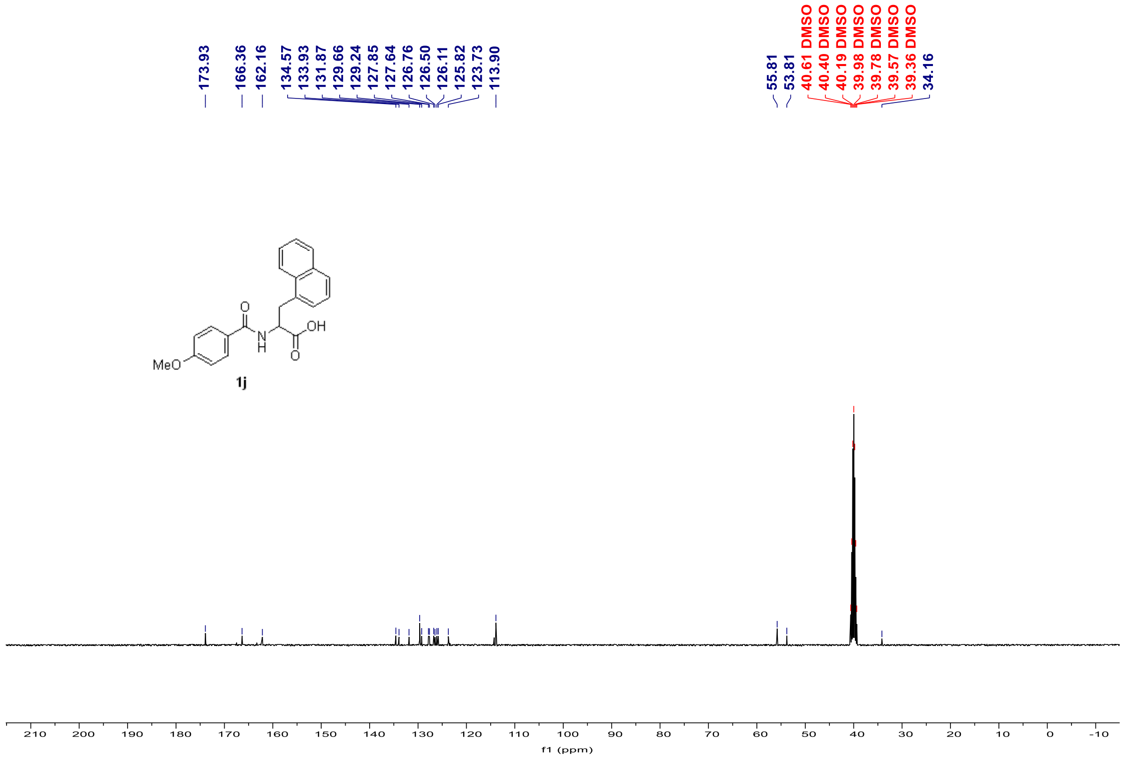

Figure S172. ^{13}**C NMR** of the **1j** (101 MHz, DMSO-d_6**)**

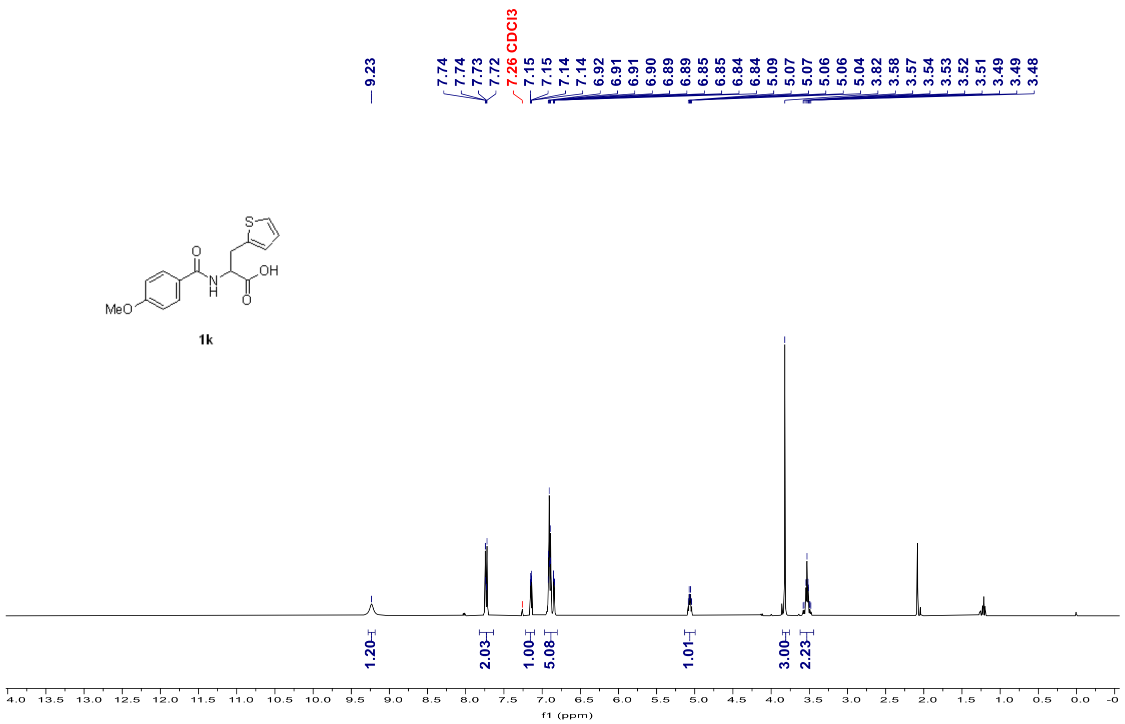

Figure S173. ¹H NMR of the **1k** (400 MHz, $CDCl_3$**)**

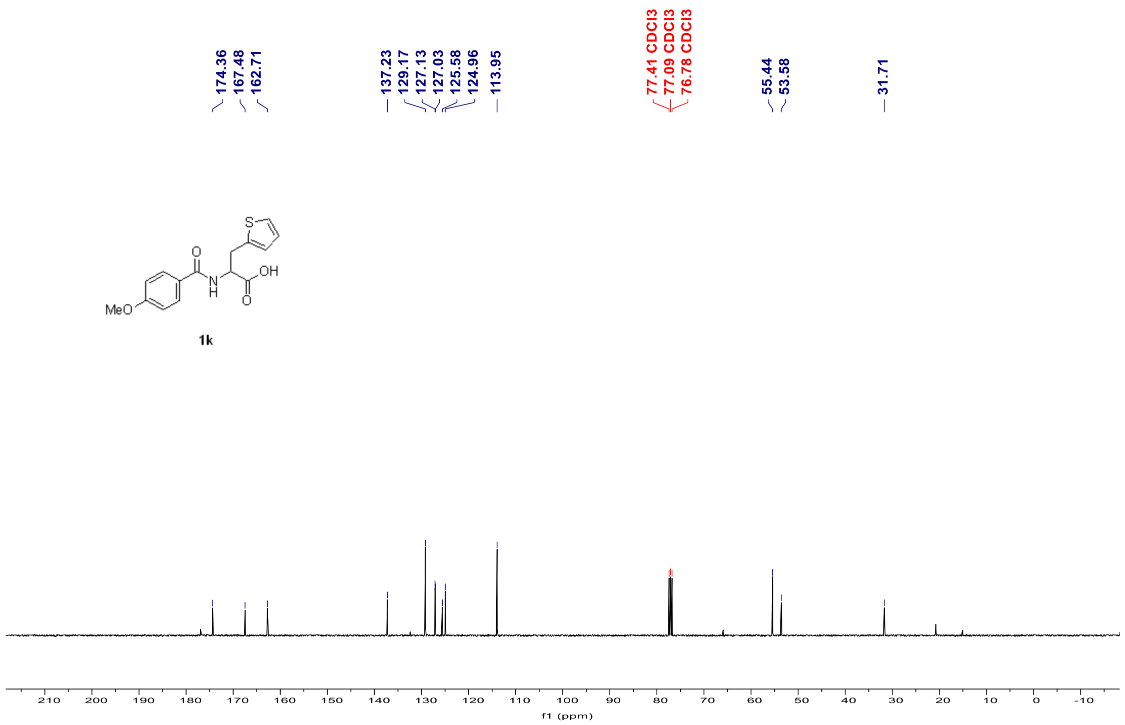

Figure S174. ¹³C NMR of the **1k** (101 MHz, $CDCl_3$**)**

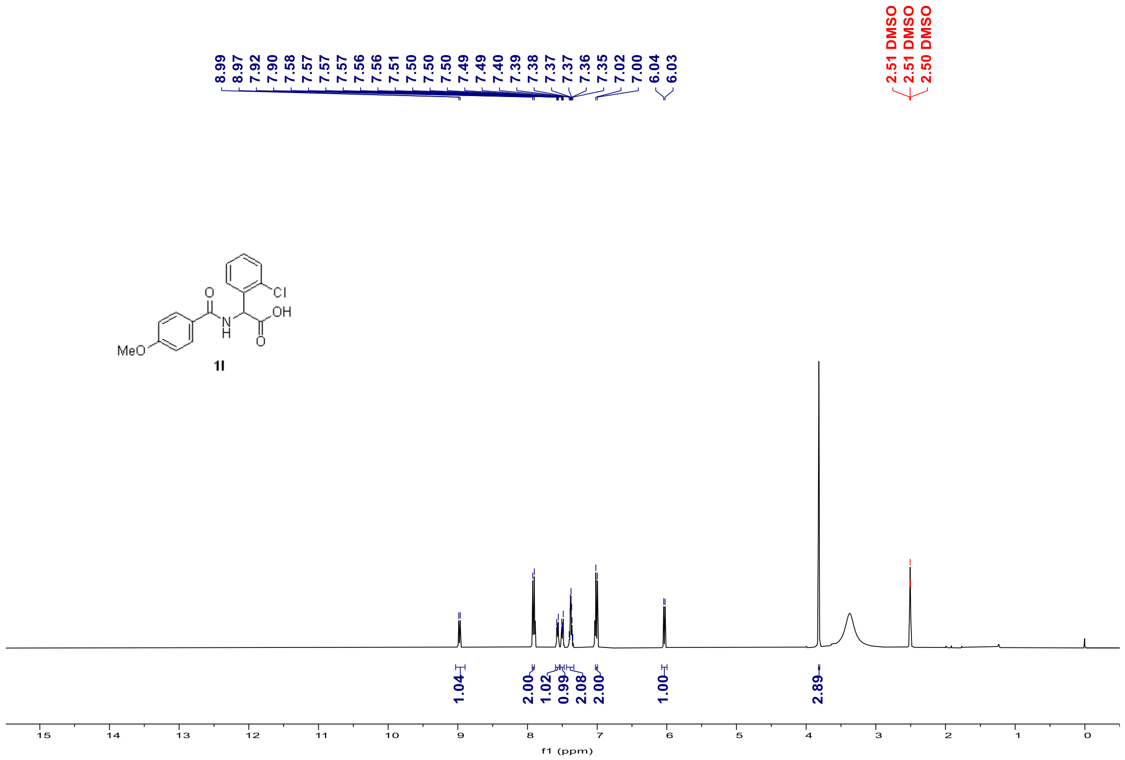

Figure S175. **^{1}H NMR** of the **1l** (400 MHz, DMSO-*d*6)

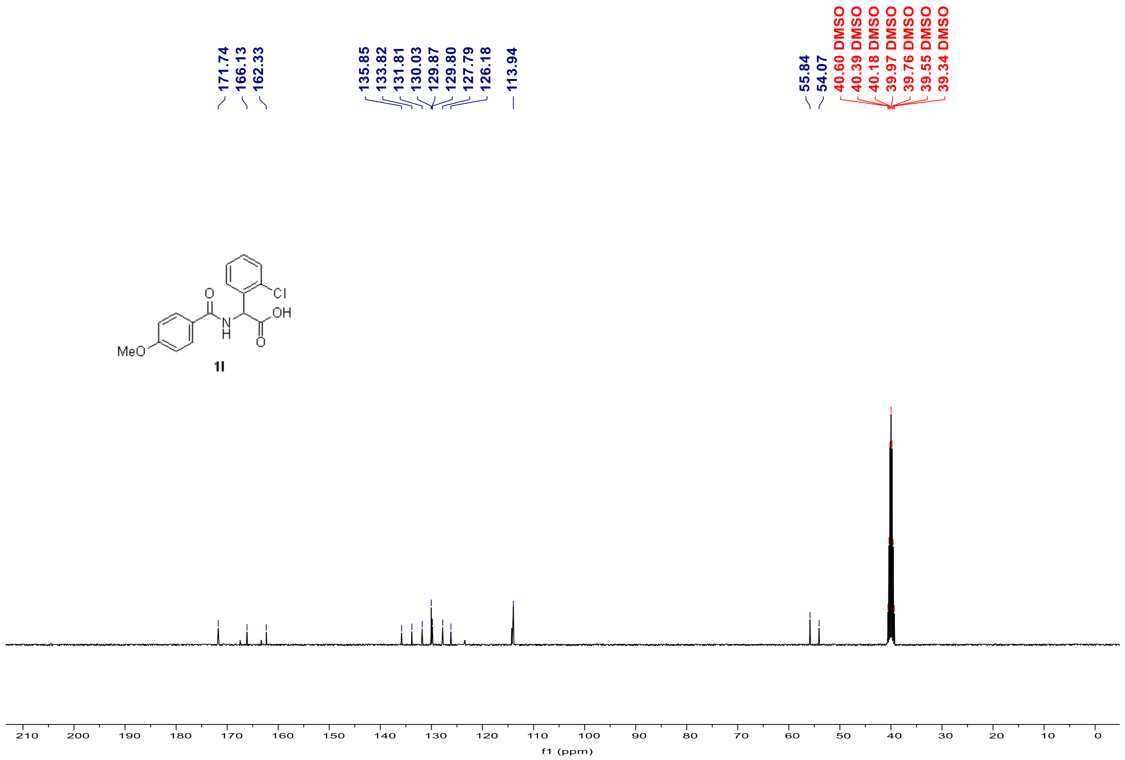

Figure S176. **^{13}C NMR** of the **1l** (101 MHz, DMSO-*d*6)

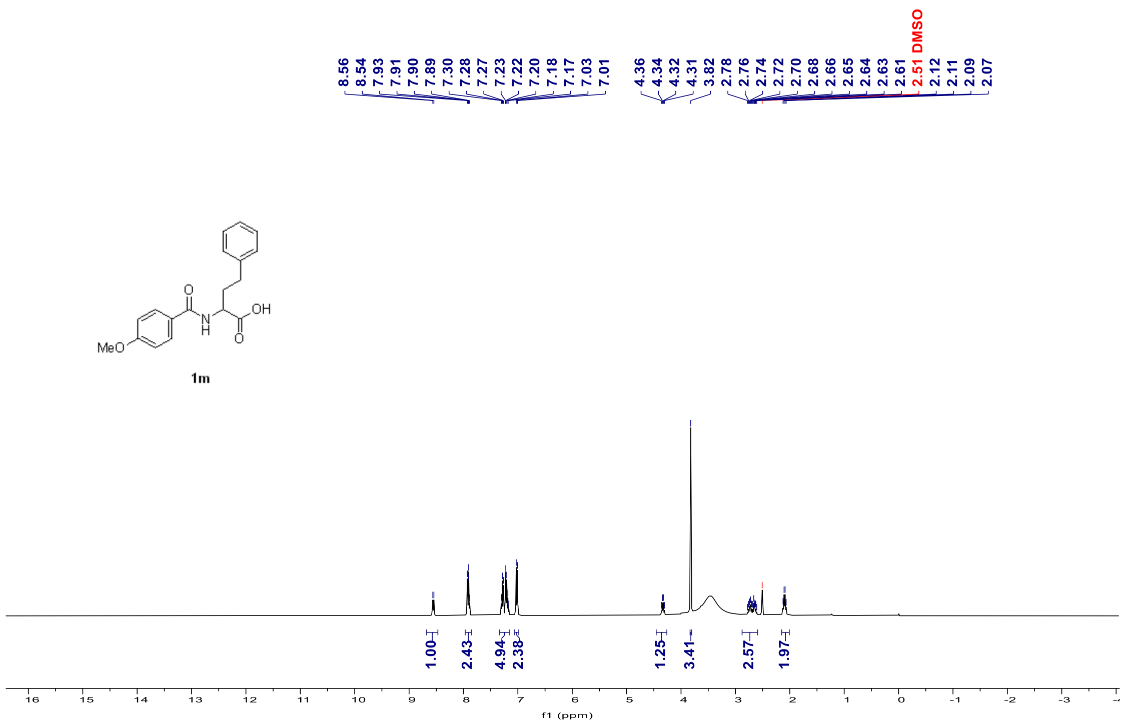

Figure S177. ^{1}**H NMR** of the **1m** (400 MHz, DMSO-d_6)

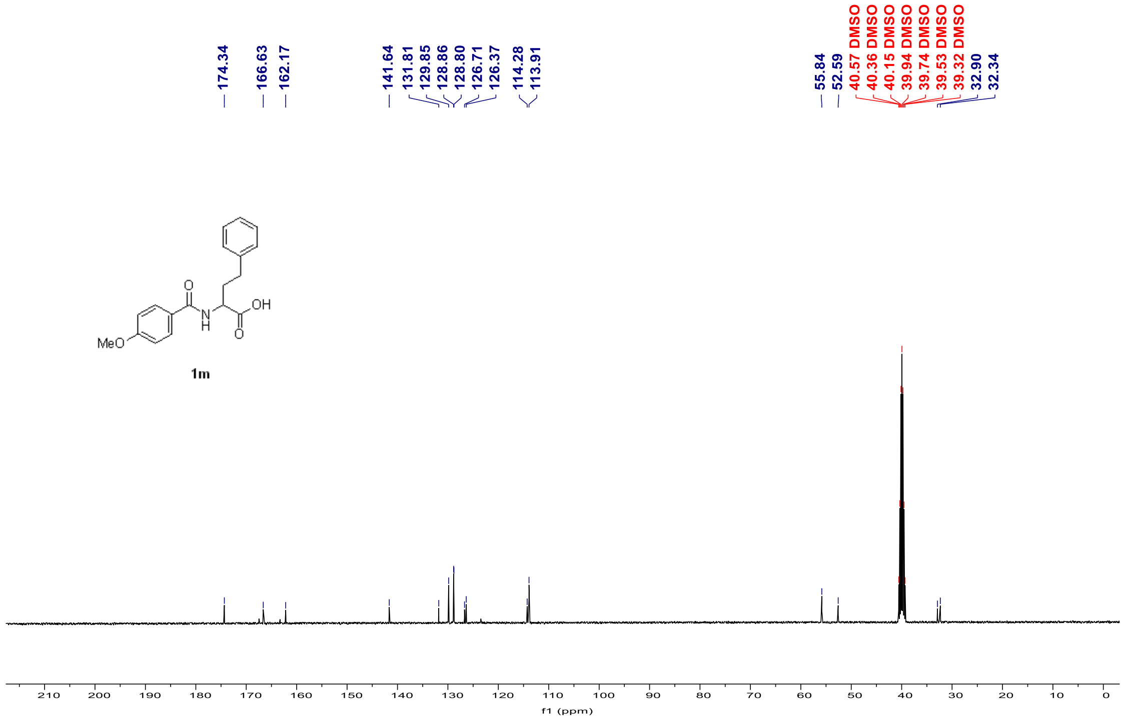

Figure S178. ^{13}**C NMR** of the **1m** (101 MHz, DMSO-d_6)

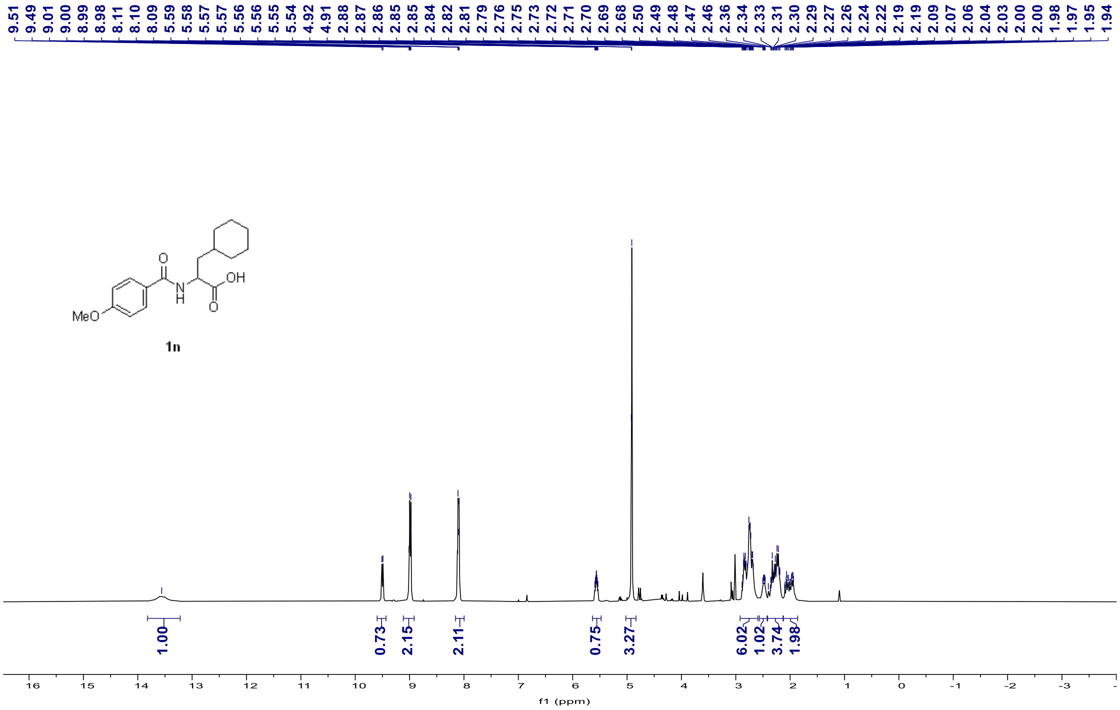

Figure S179. ^{1}H NMR of the **1n** (400 MHz, DMSO-d_6**)**

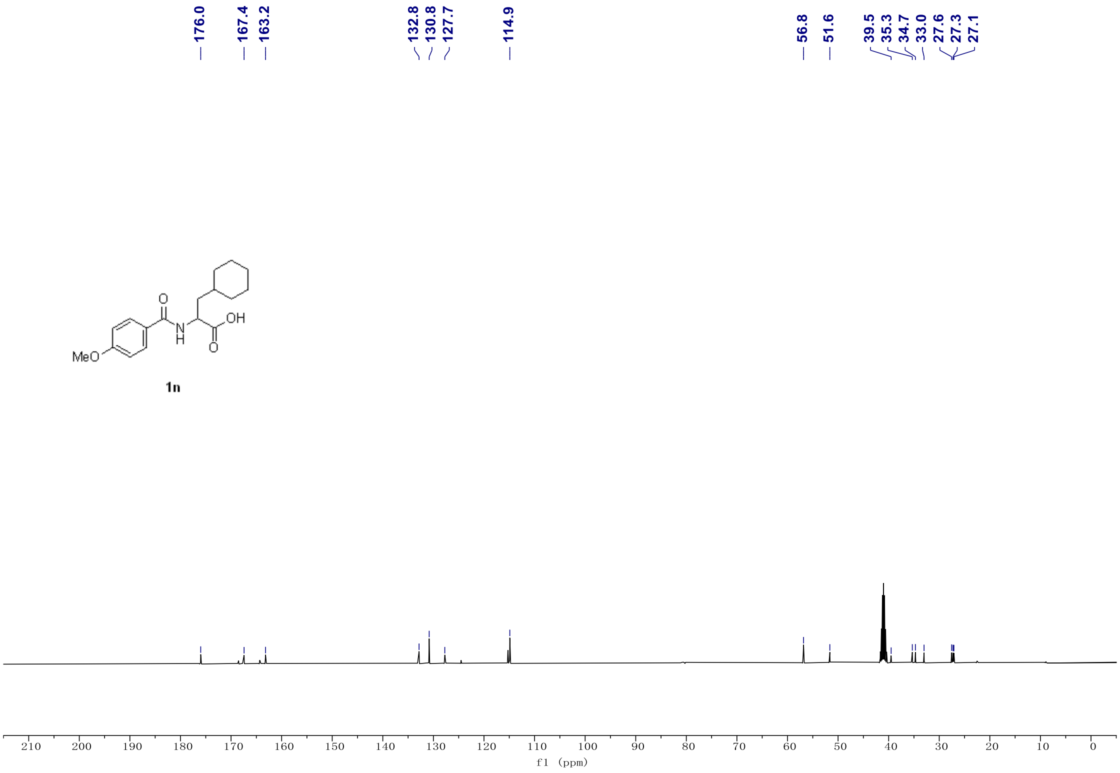

Figure S180. ^{13}C NMR of the **1n** (101 MHz, DMSO-d_6**)**

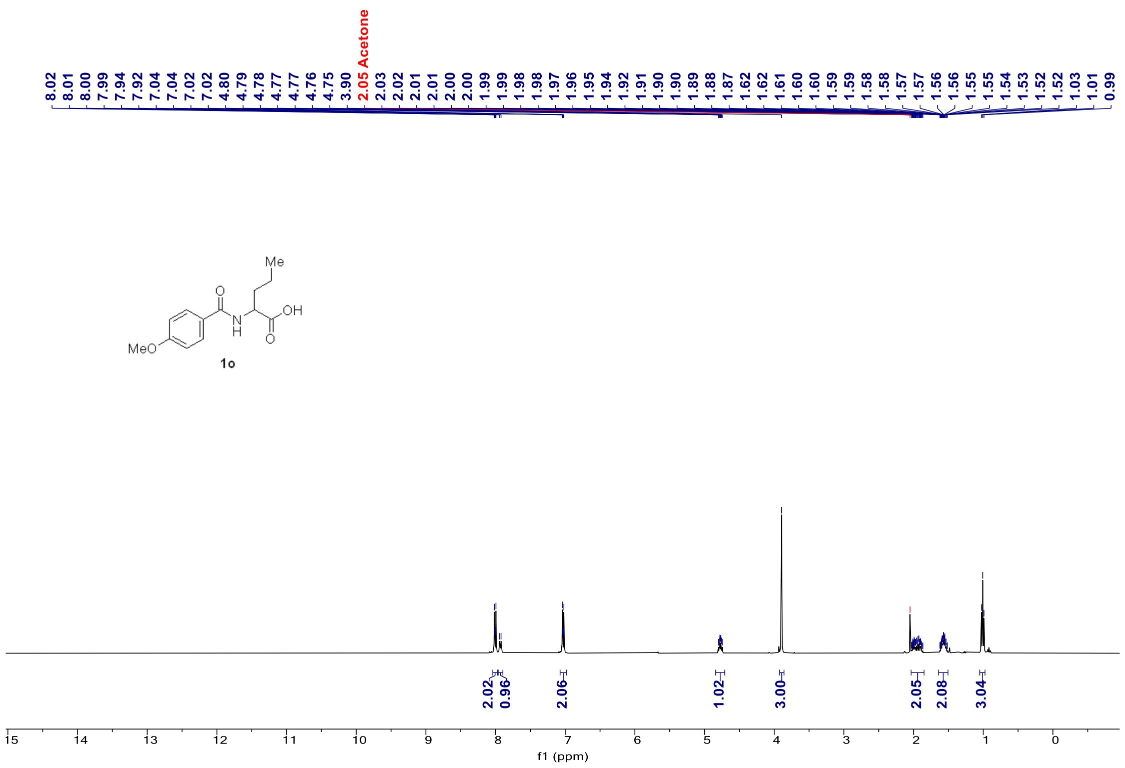

Figure S181. ^{1}**H NMR** of the **1o** (400 MHz, Acetone-d_6)

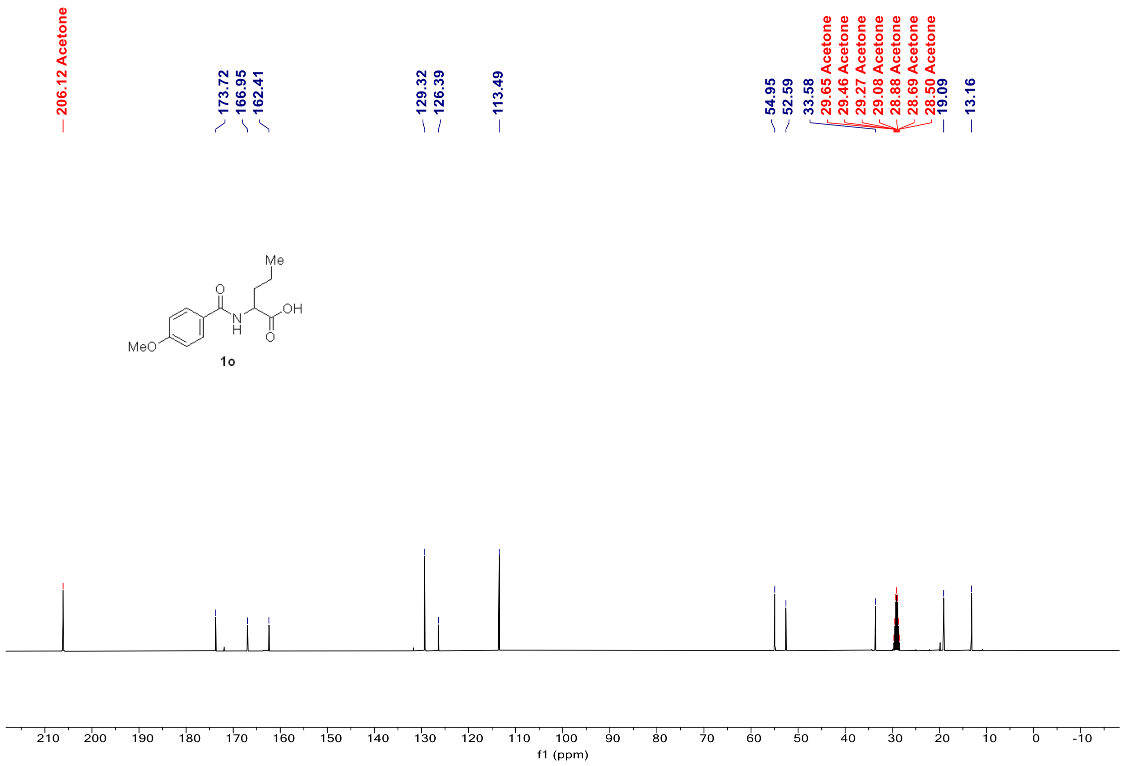

Figure S182. ^{13}**C NMR** of the **1o** (101 MHz, Acetone-d_6)

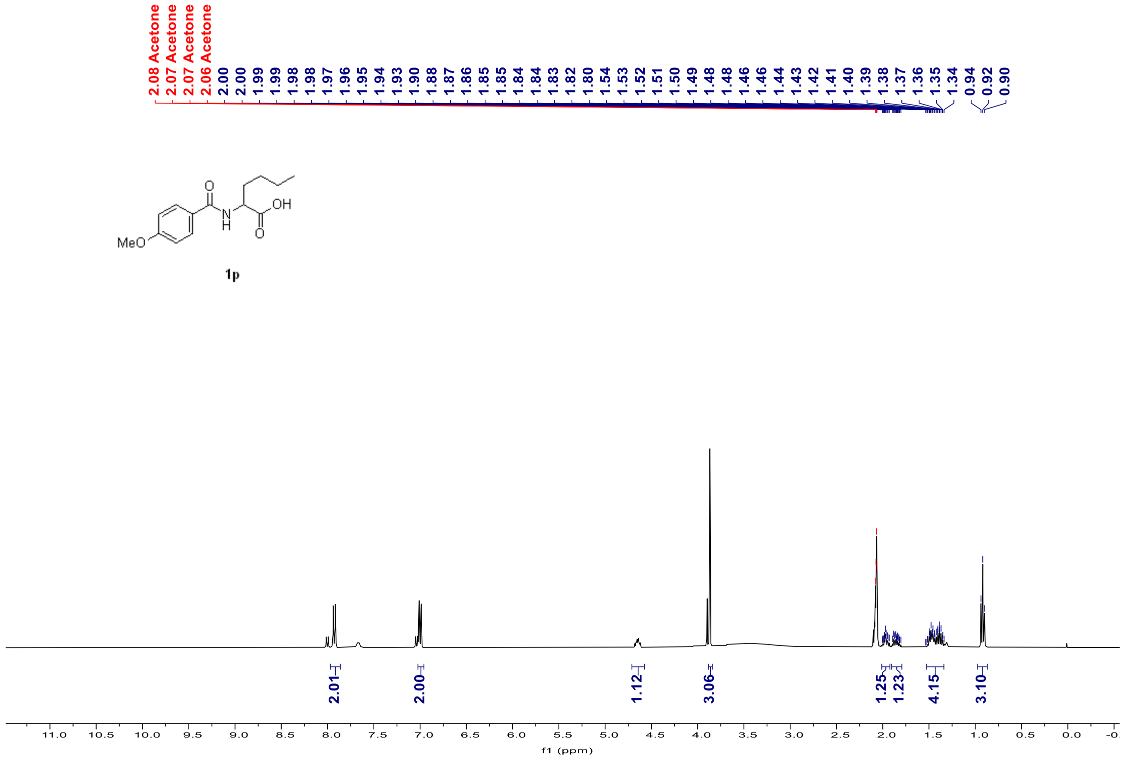

Figure S183. **^{1}H NMR** of the **1p** (400 MHz, Acetone-d_6)

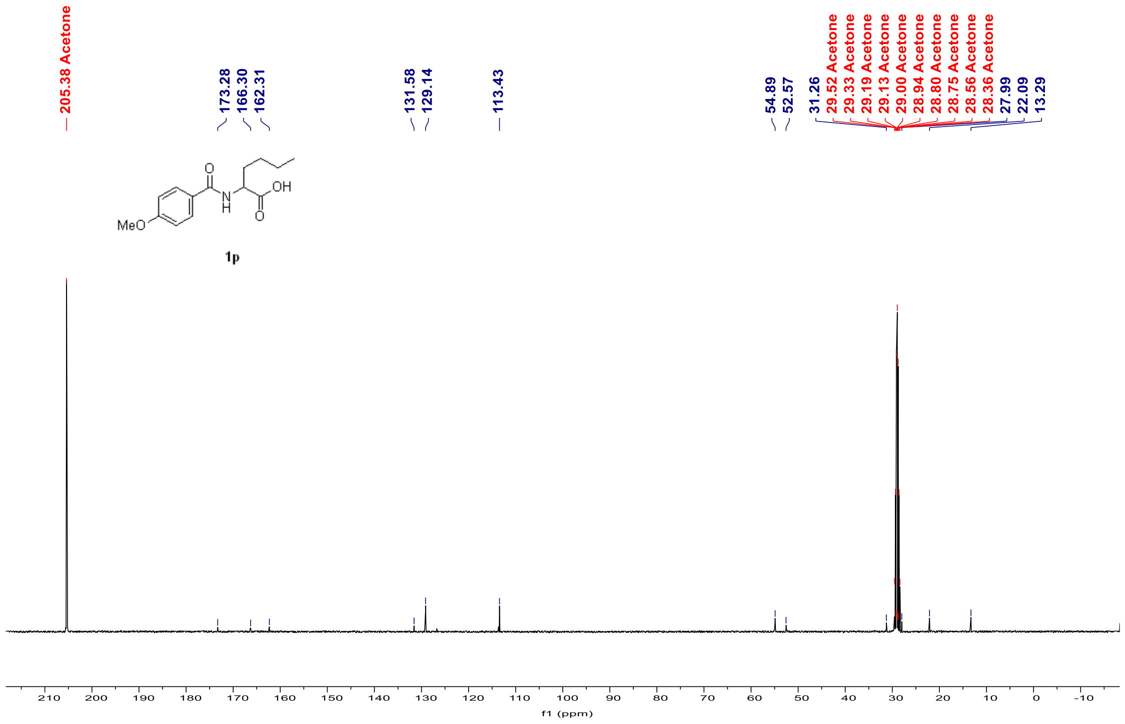

Figure S184. **^{13}C NMR** of the **1p** (101 MHz, Acetone-d_6)

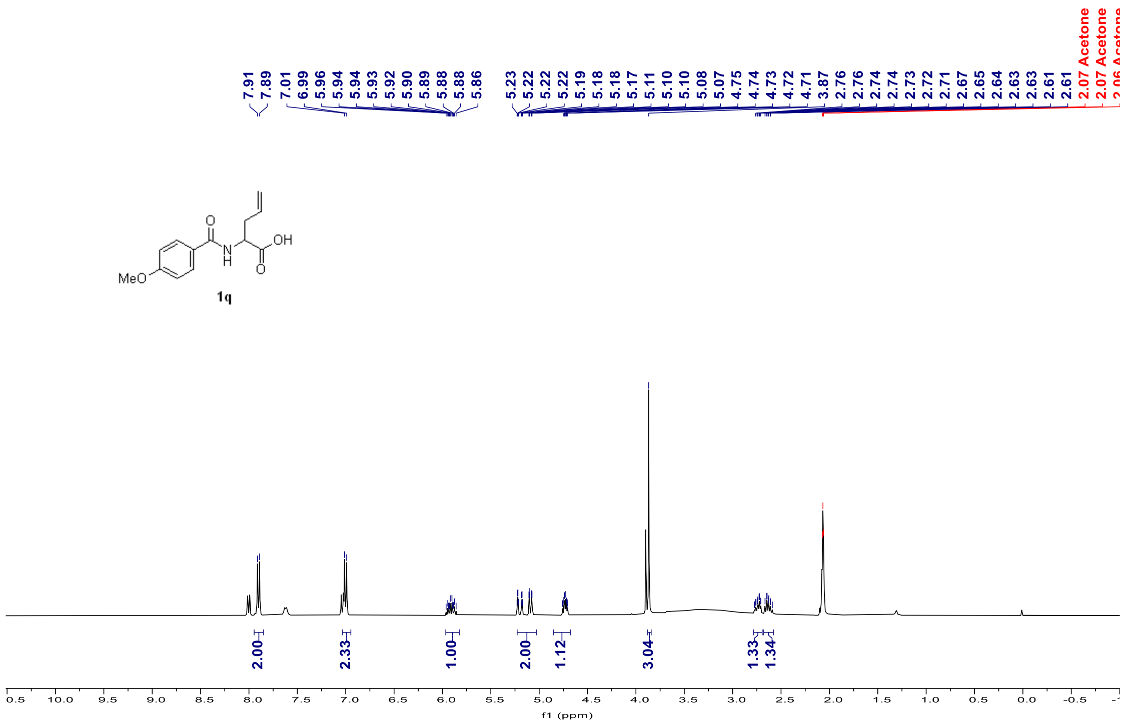

Figure S185. ^{1}**H NMR** of the **1q** (400 MHz, Acetone-d_6)

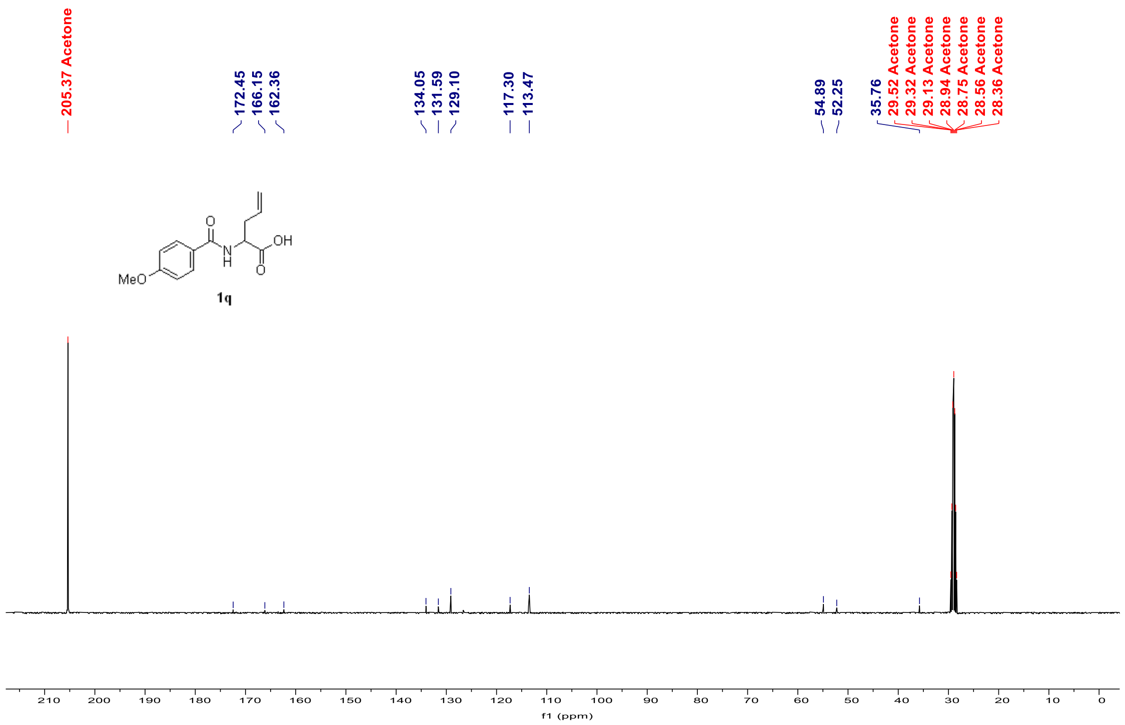

Figure S186. ^{13}**C NMR** of the **1q** (101 MHz, Acetone-d_6)

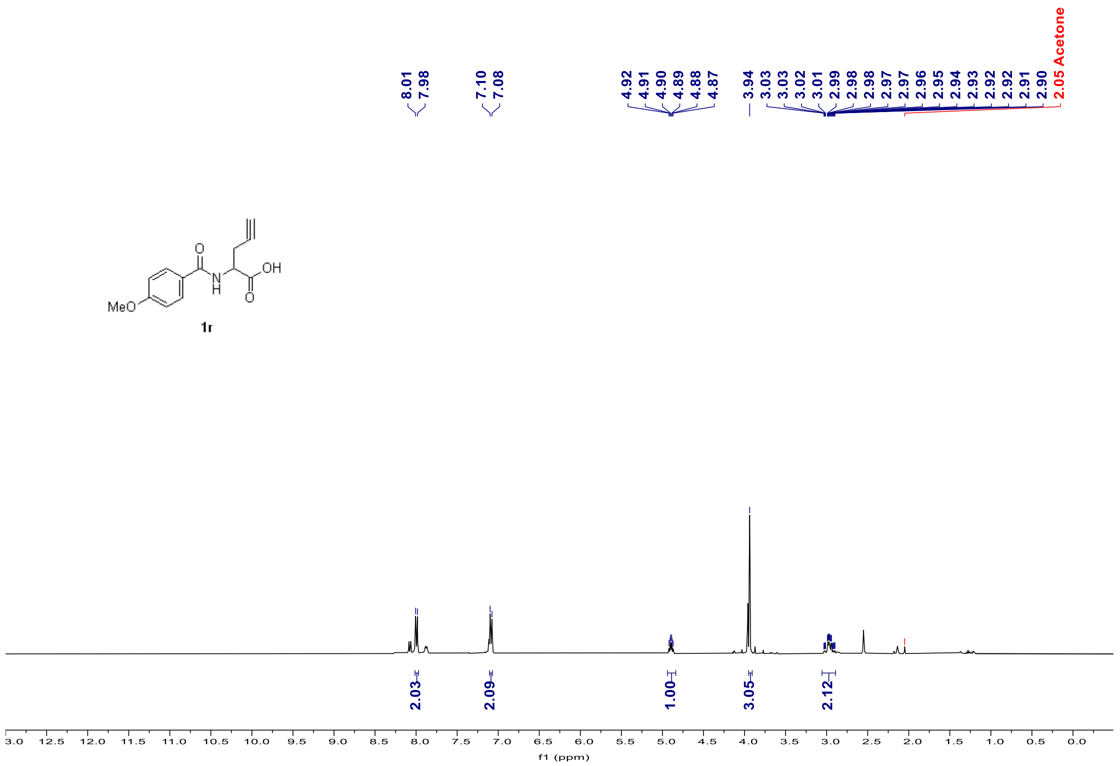

Figure S187. ^{1}**H NMR** of the **1r** (400 MHz, Acetone-d_6)

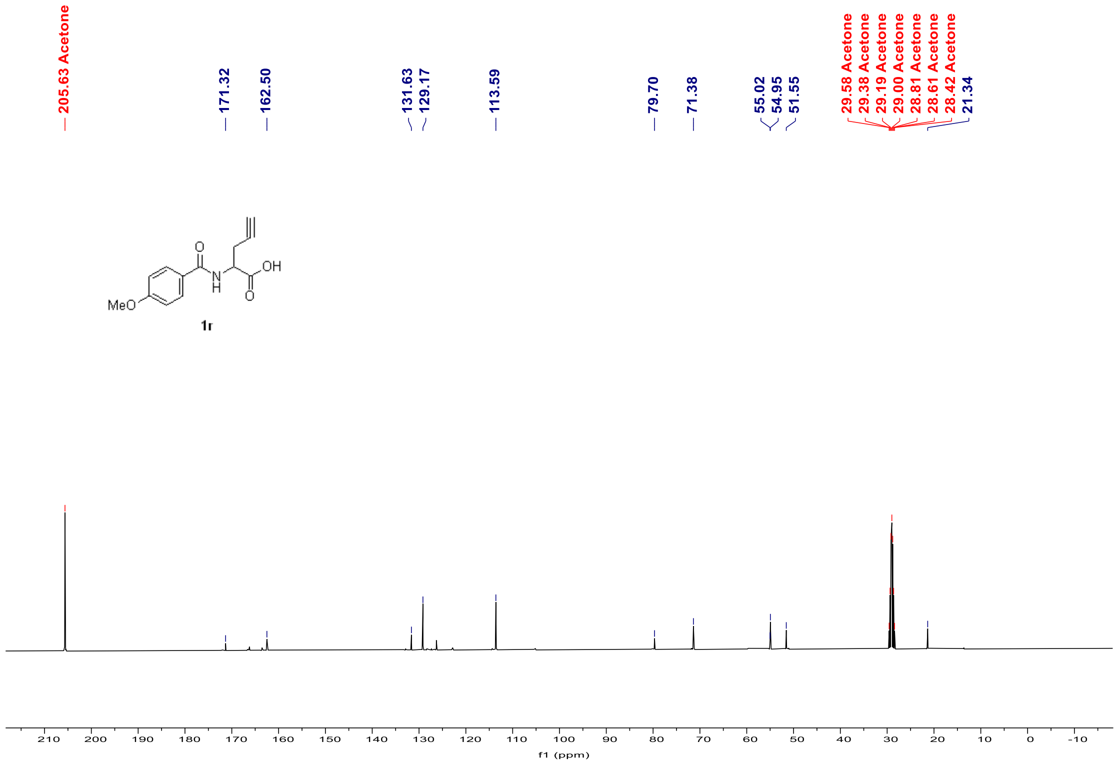

Figure S188. ^{13}**C NMR** of the **1r** (101 MHz, Acetone-d_6)

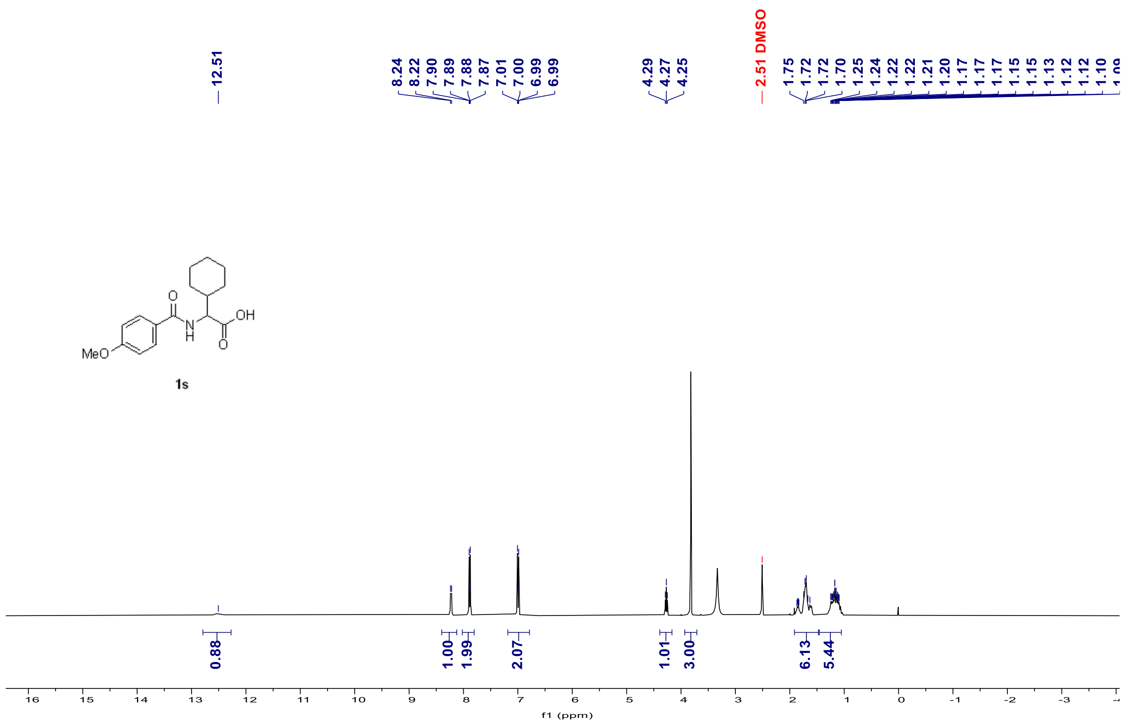

Figure S189. **^{1}H NMR** of the **1s** (400 MHz, DMSO-d_6)

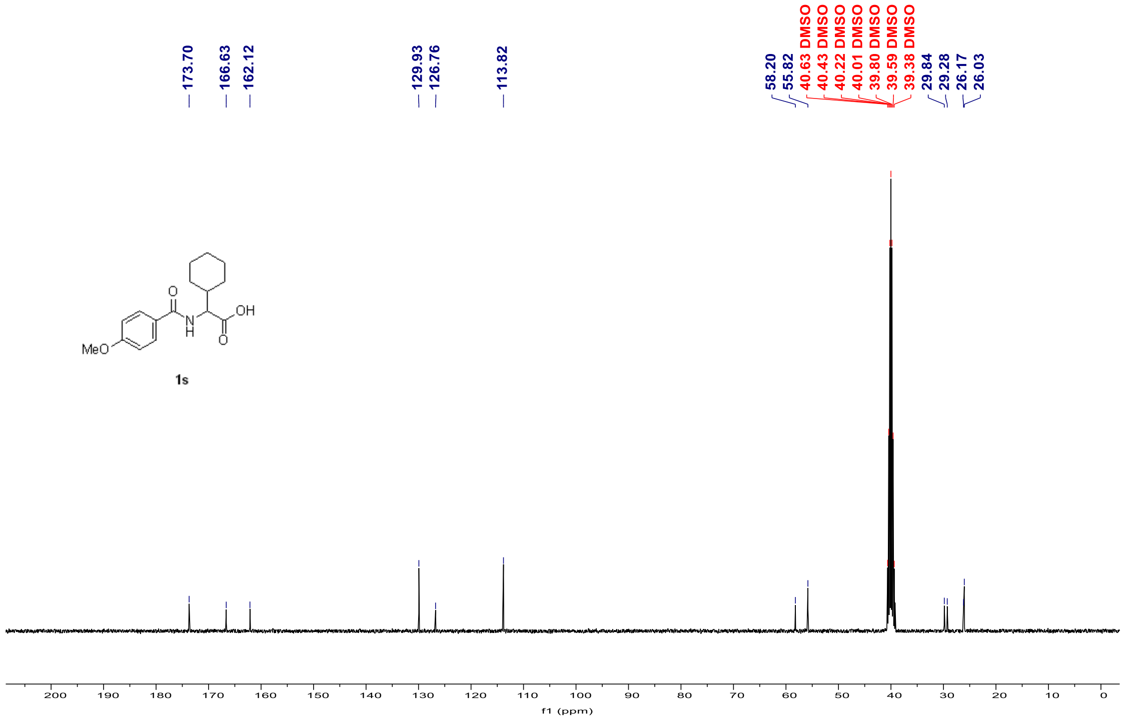

Figure S190. **^{13}C NMR** of the **1s** (101 MHz, DMSO-d_6)

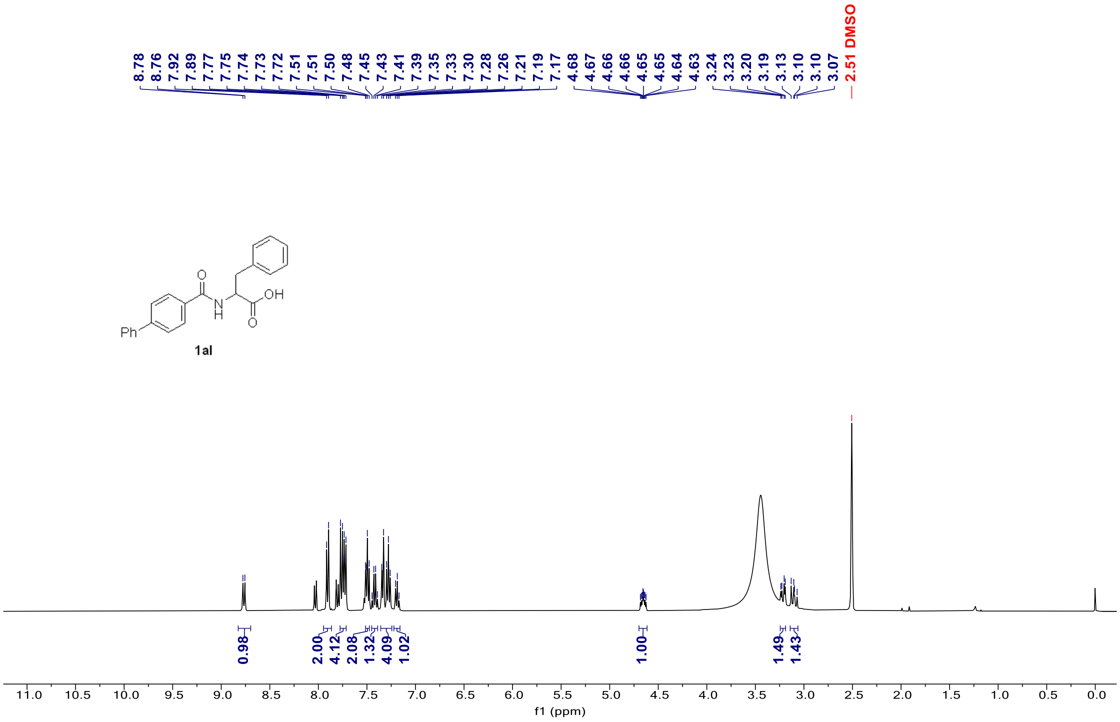

Figure S191. ^{1}**H NMR** of the **1al** (400 MHz, DMSO-d_6)

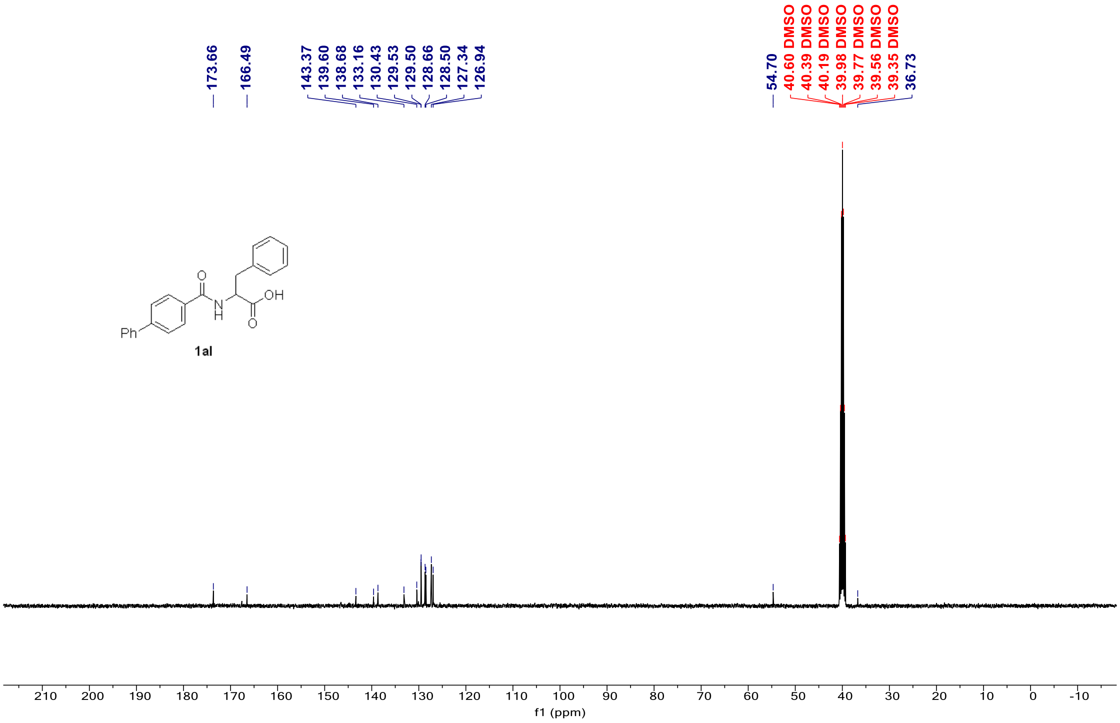

Figure S192. ^{13}**C NMR** of the **1al** (101 MHz, DMSO-d_6)

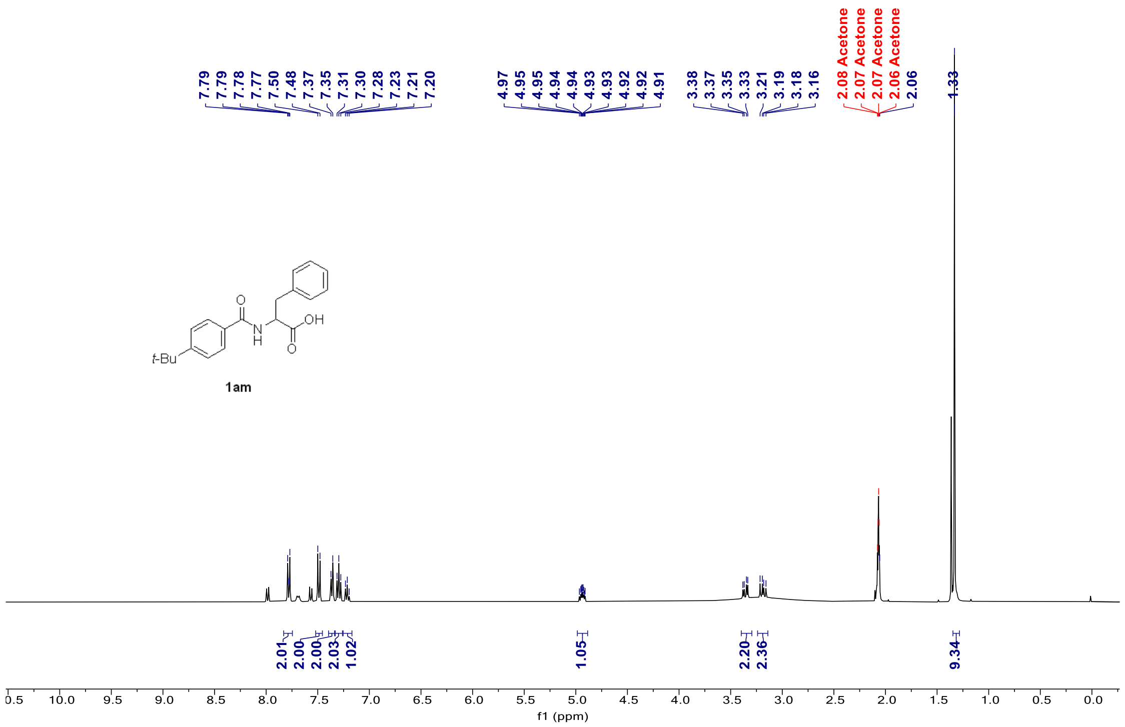

Figure S193. ^{1}**H NMR** of the **1am** (400 MHz, Acetone-d_6)

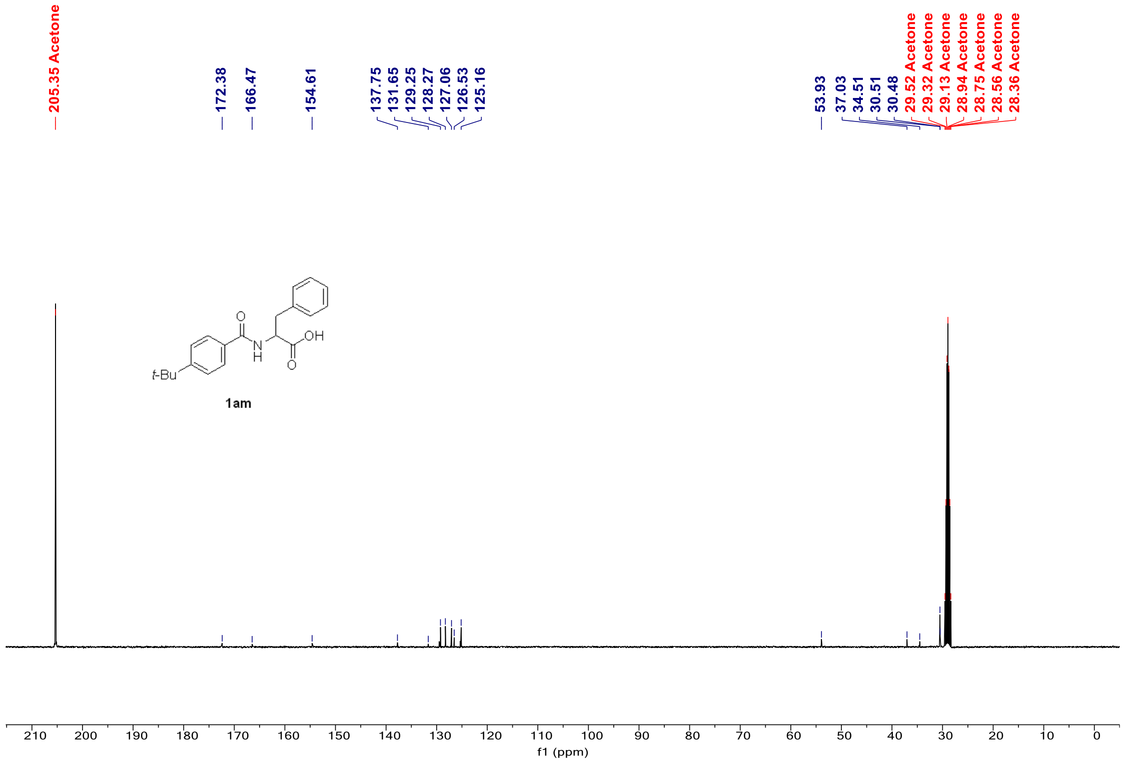

Figure S194. ^{13}**C NMR** of the **1am** (101 MHz, Acetone-d_6)

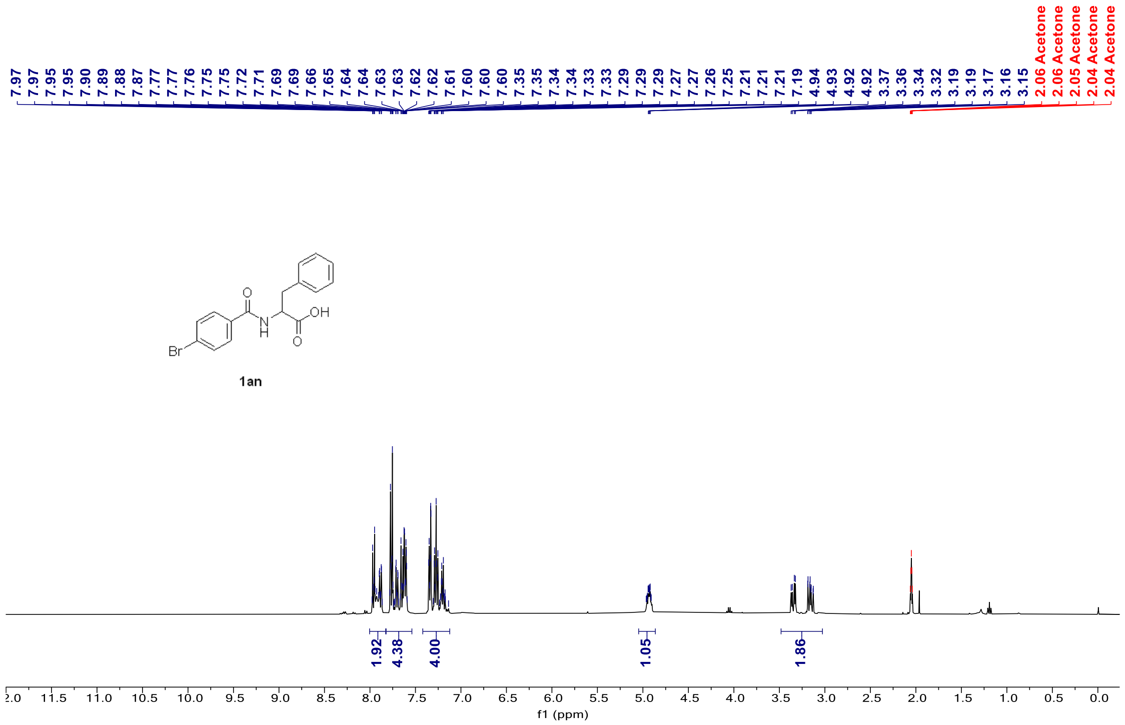

Figure S195. ^{1}**H NMR** of the **1an** (400 MHz, Acetone-d_6)

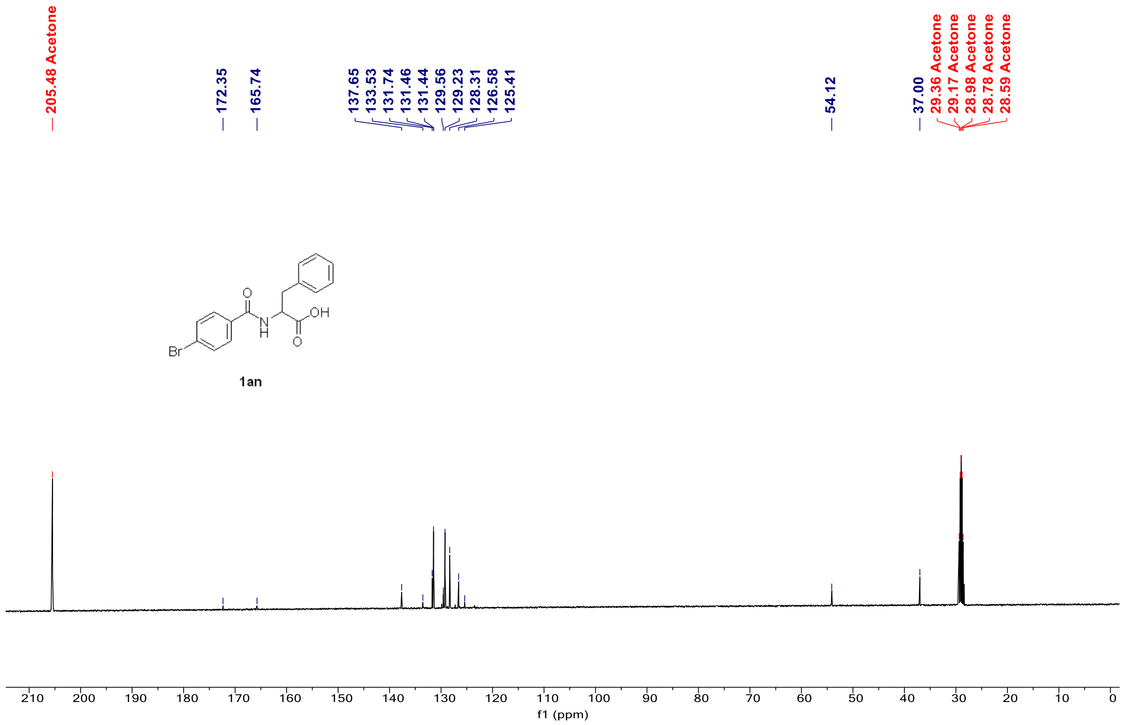

Figure S196. ^{13}**C NMR** of the **1an** (101 MHz, Acetone-d_6)

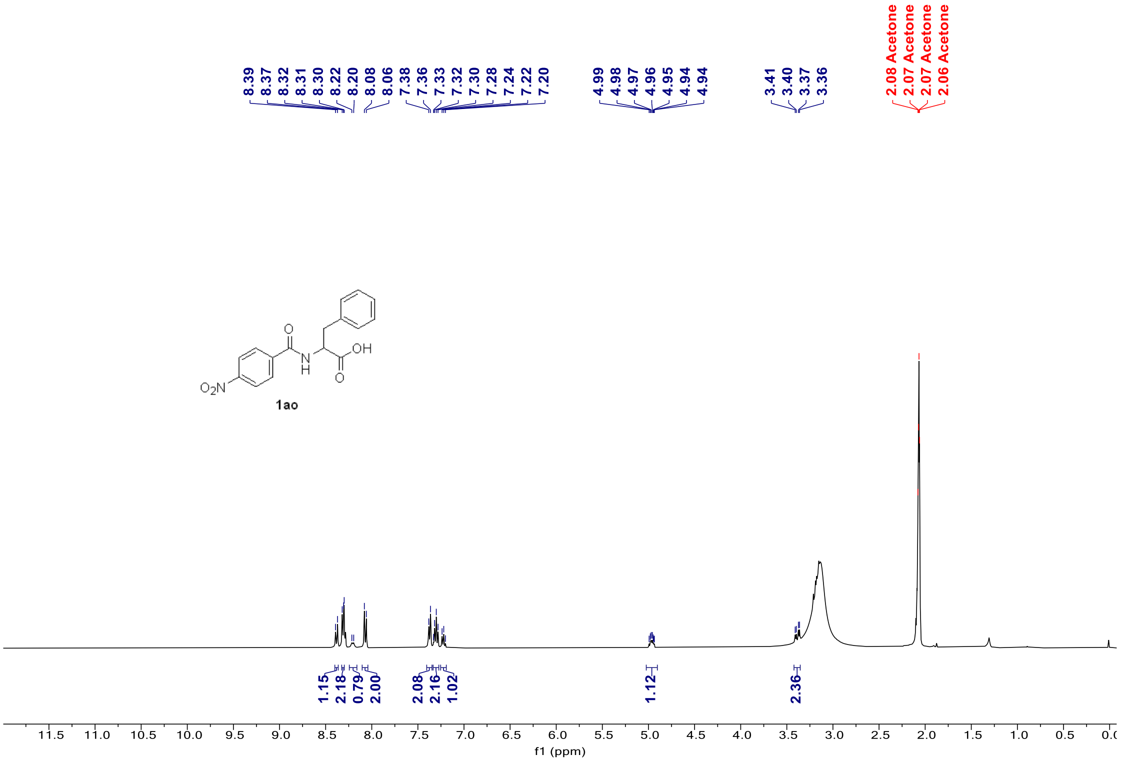

Figure S197. ^{1}**H NMR** of the **1ao** (400 MHz, Acetone-d_6)

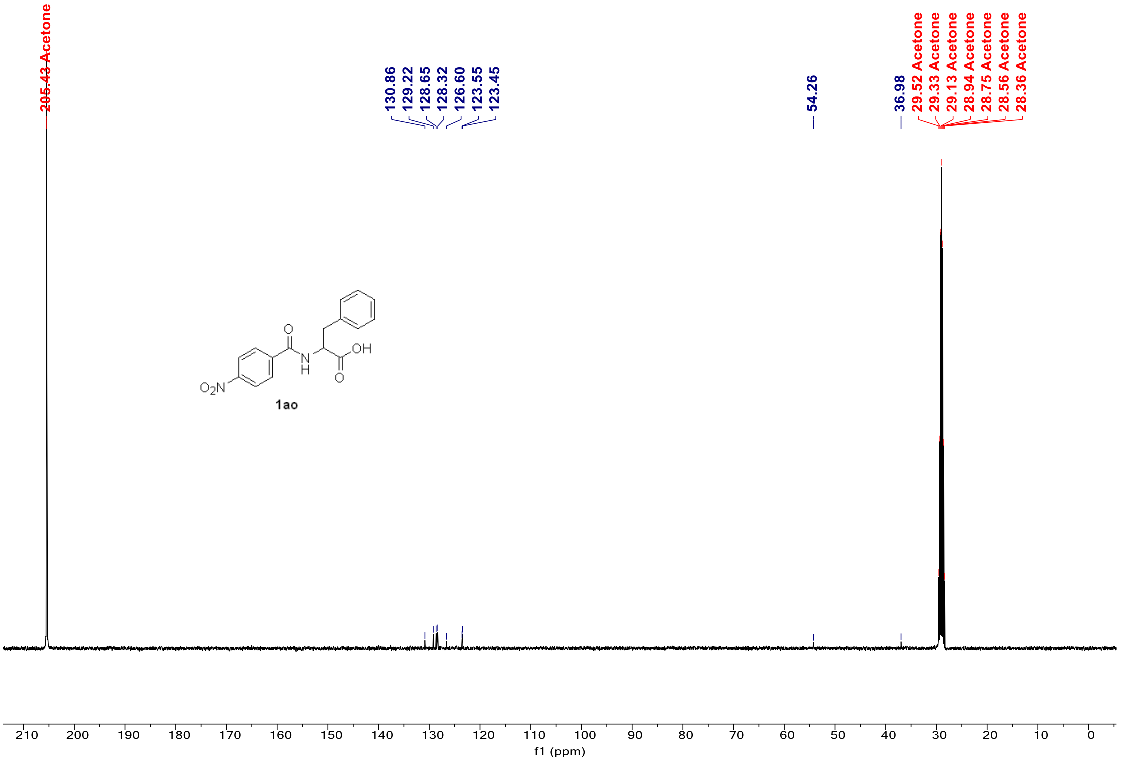

Figure S198. ^{13}**C NMR** of the **1ao** (101 MHz, Acetone-d_6)

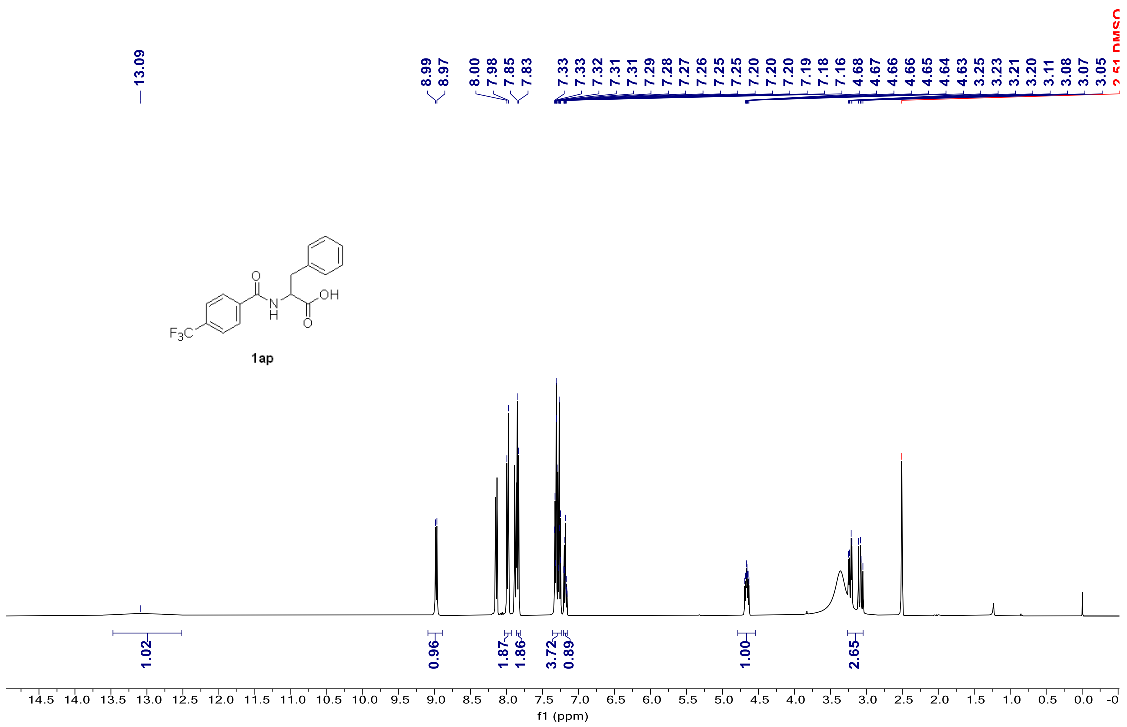

Figure S199. ^{1}**H NMR** of the **1ap** (400 MHz, DMSO-d_6)

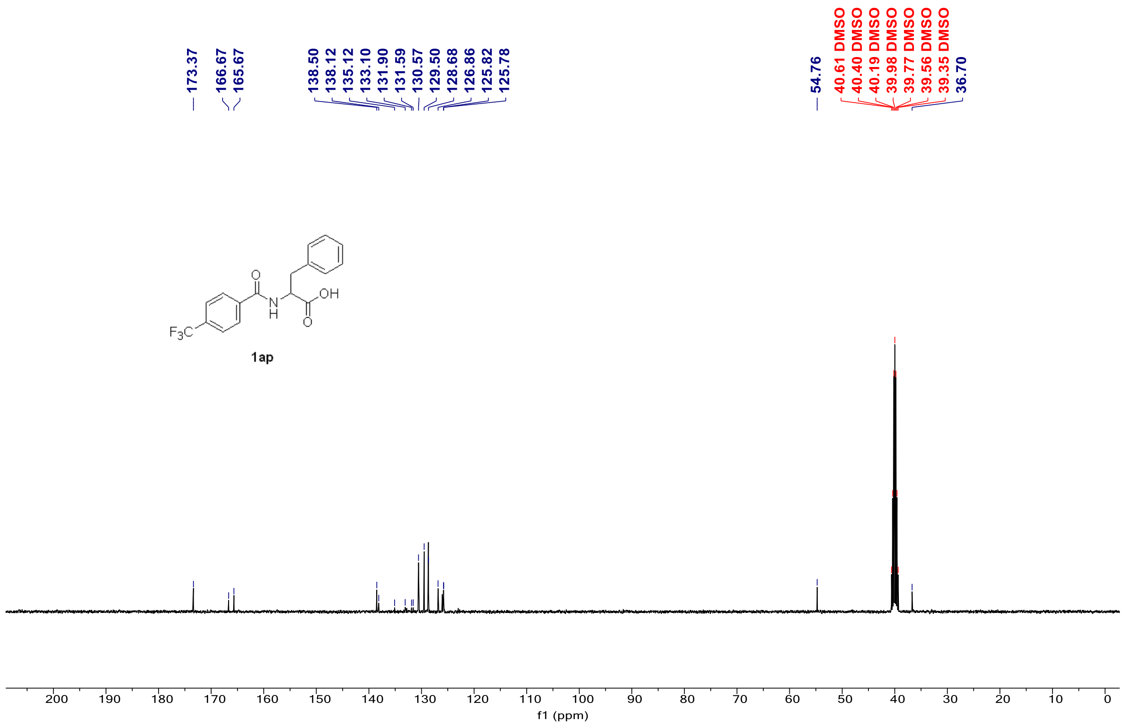

Figure S200. ^{13}**C NMR** of the **1ap** (101 MHz, DMSO-d_6)

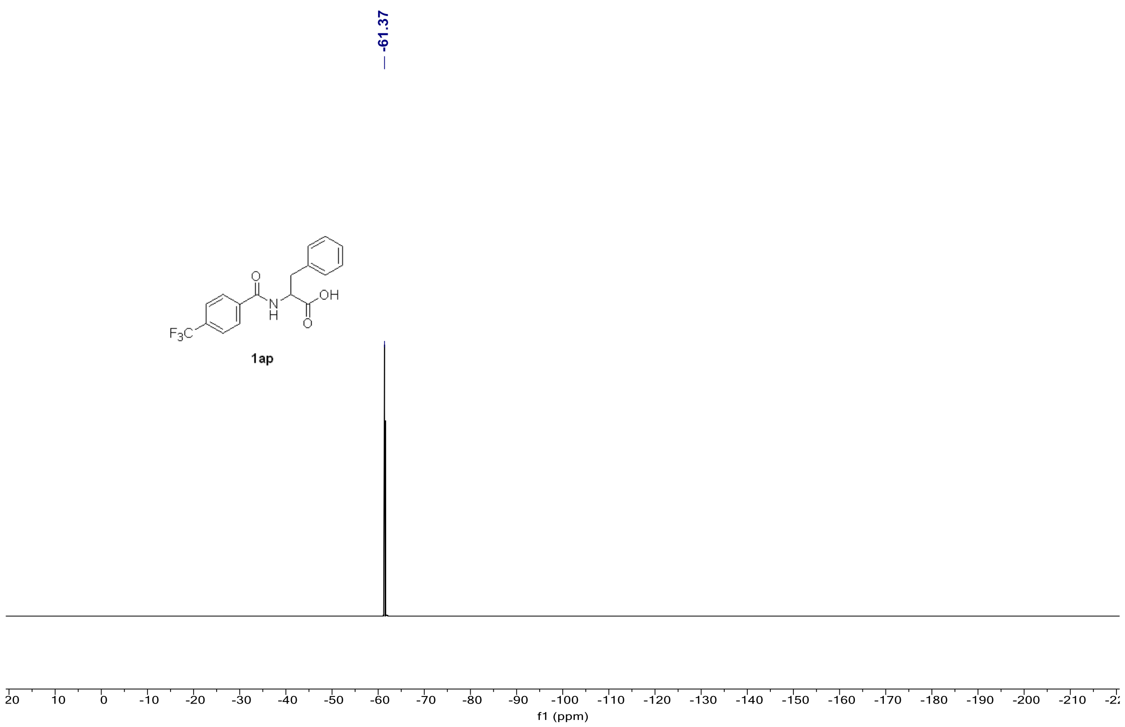

Figure S201. **^{19}F NMR** of the **1ap** (376 MHz, DMSO-d_6)

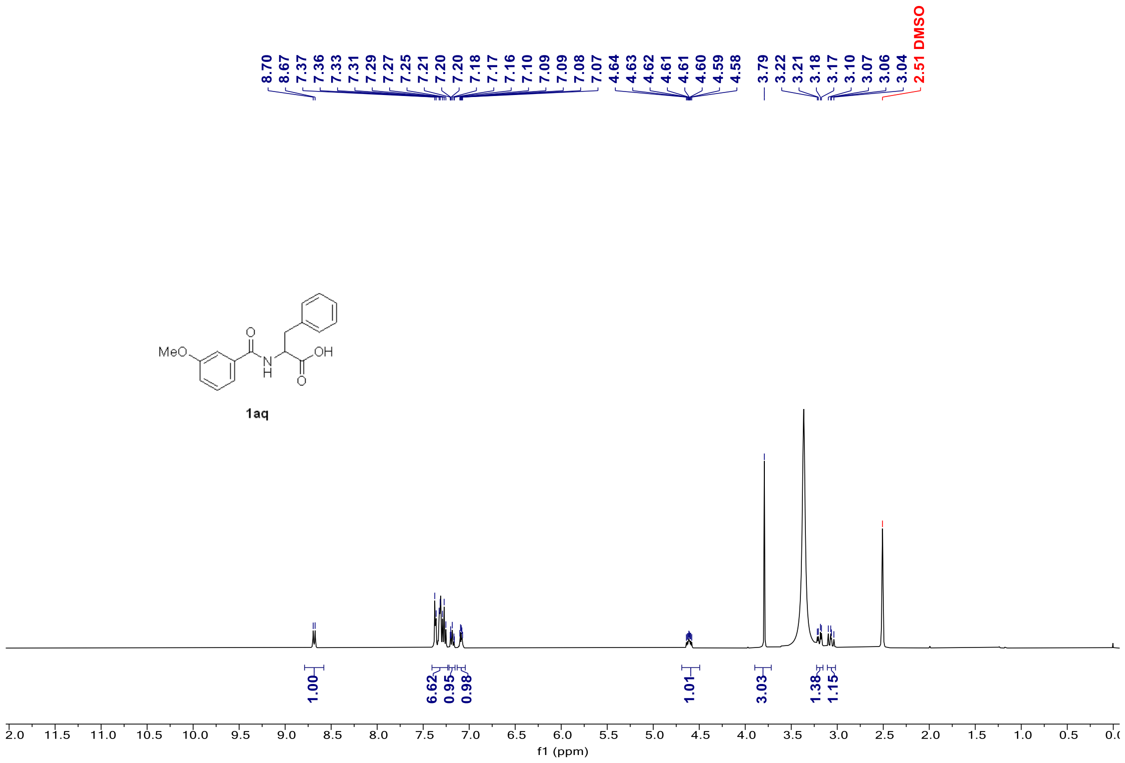

Figure S202. **^{1}H NMR** of the **1aq** (400 MHz, DMSO-d_6)

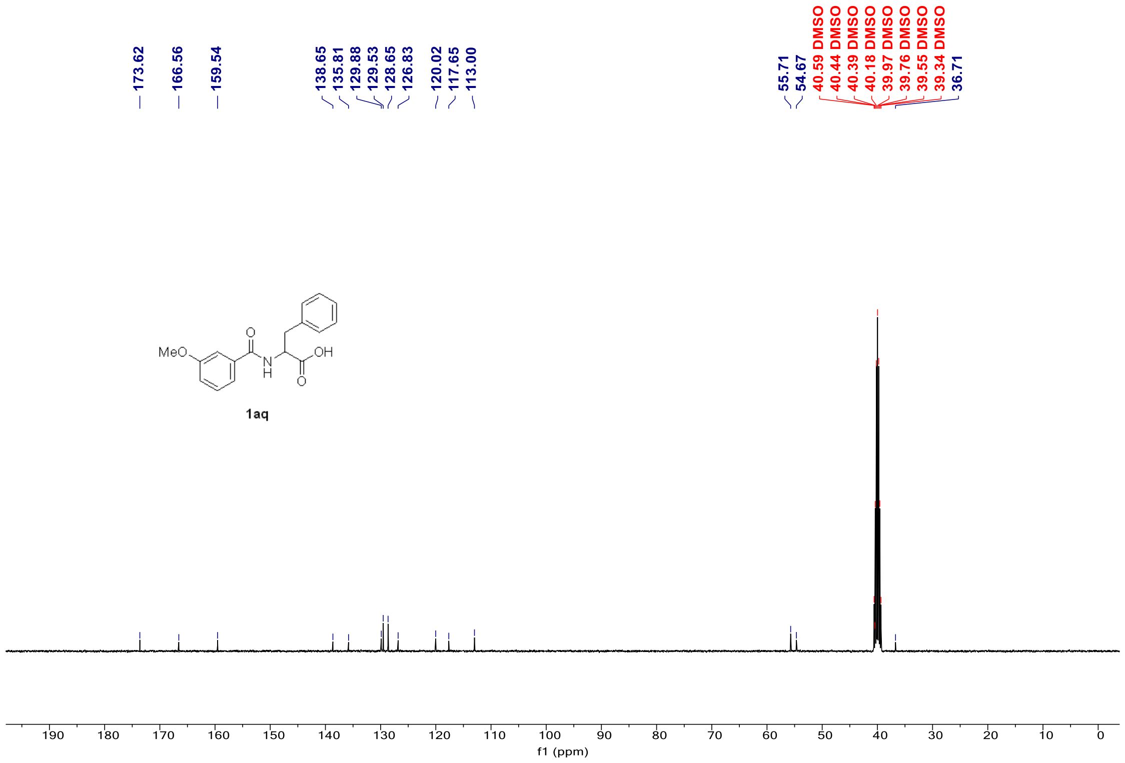

Figure S203. **^{13}C NMR** of the **1aq** (101 MHz, DMSO-d_6)

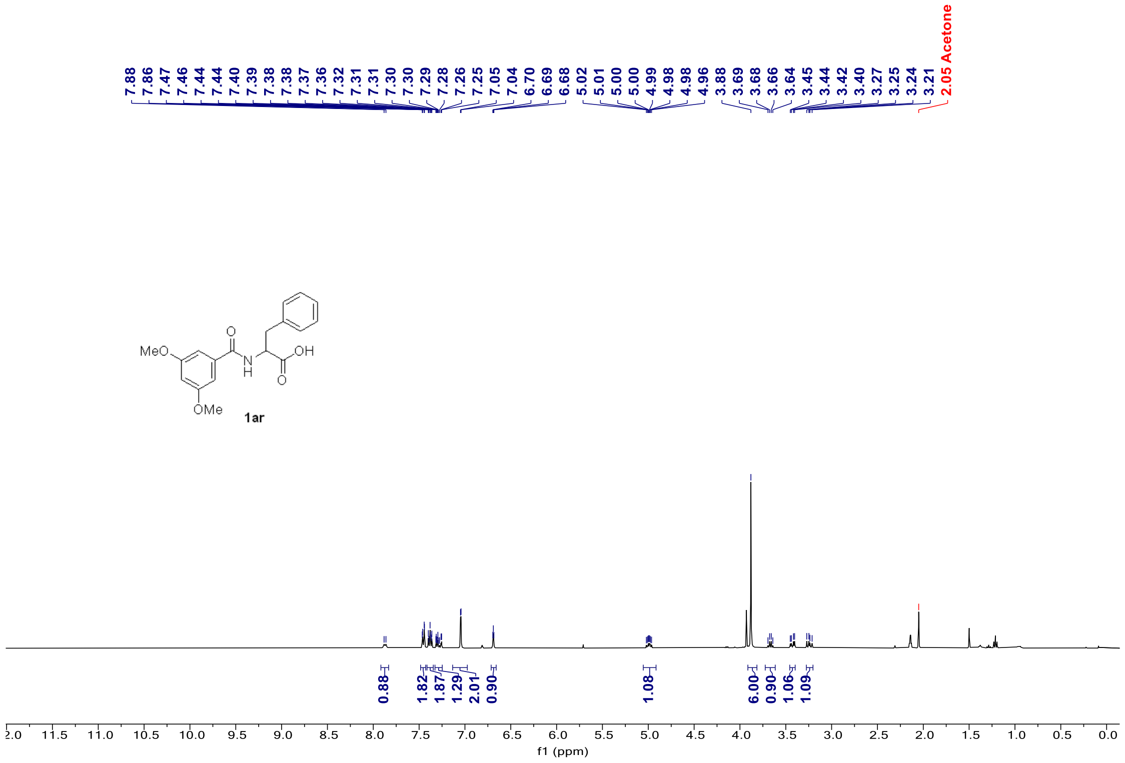

Figure S204. ^{1}**H NMR** of the **1ar** (400 MHz, Acetone-d_6)

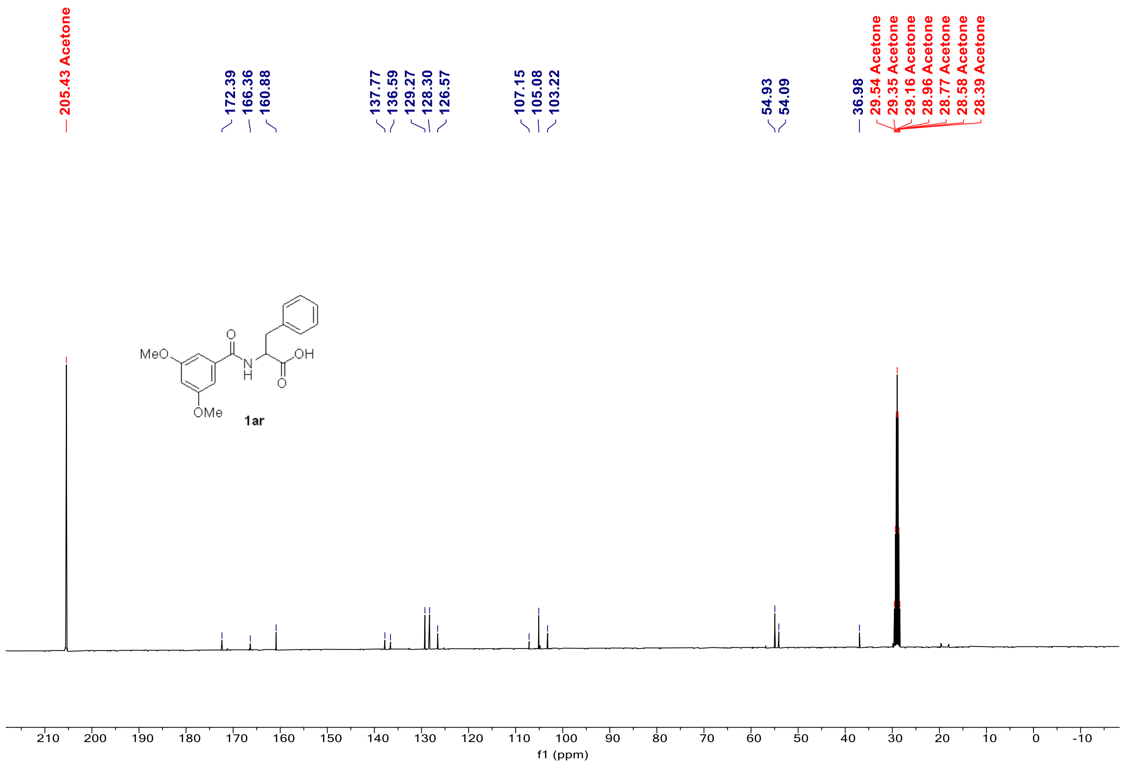

Figure S205. ^{13}**C NMR** of the **1ar** (101 MHz, Acetone-d_6)

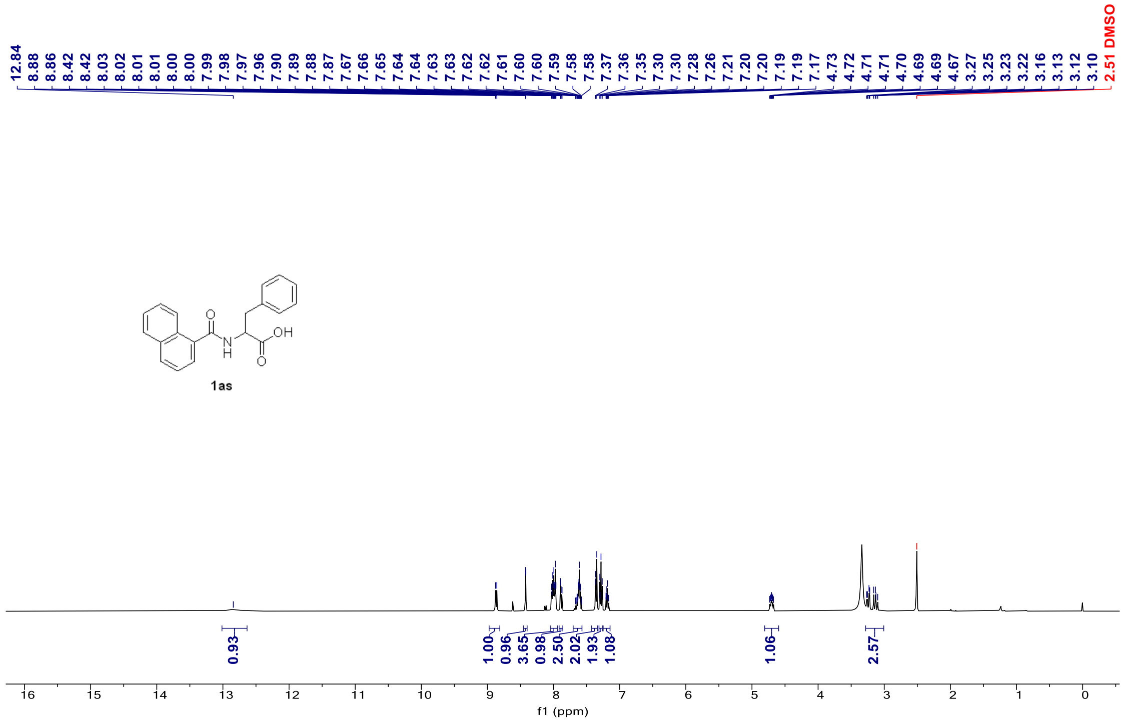

Figure S206. ^{1}**H NMR** of the **1as** (400 MHz, DMSO-d_6)

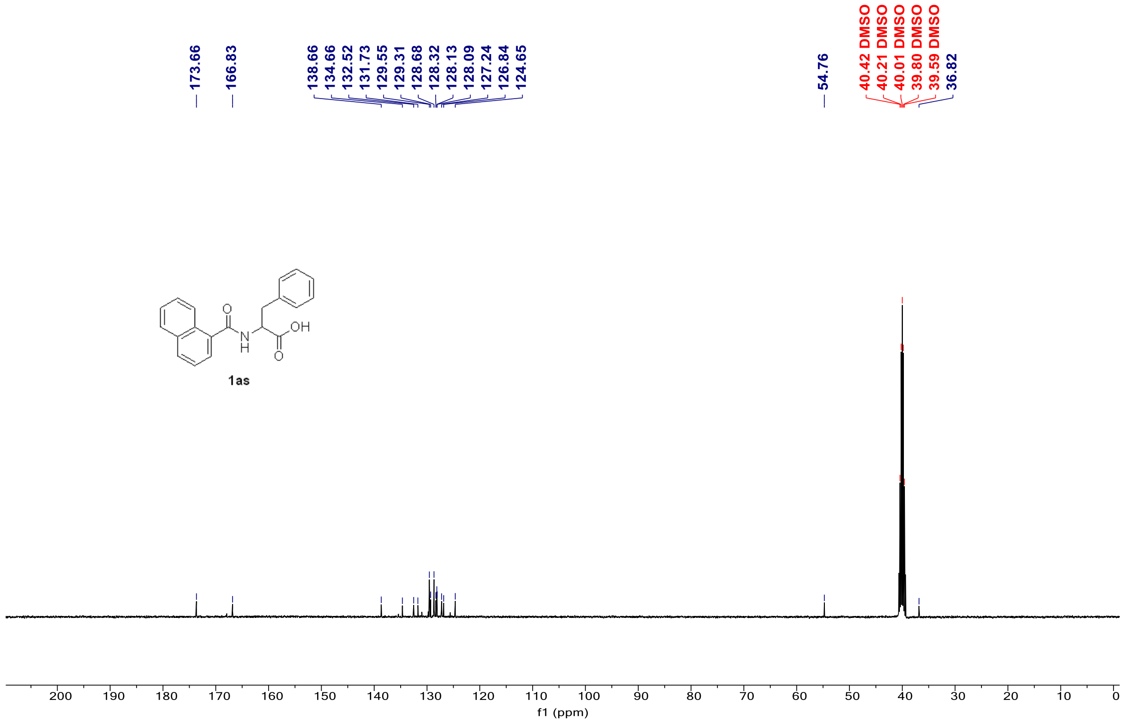

Figure S207. ^{13}**C NMR** of the **1as** (101 MHz, DMSO-d_6)

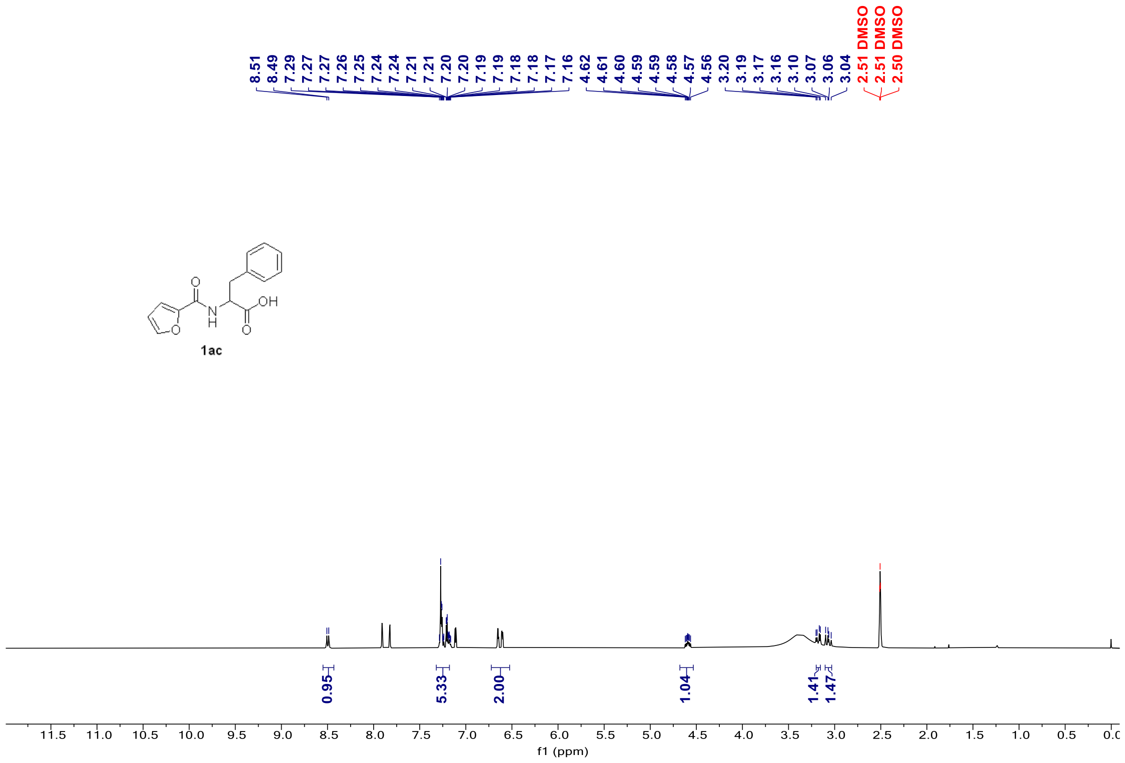

Figure S208. ^{1}**H NMR** of the **1ac** (400 MHz, DMSO-d_6)

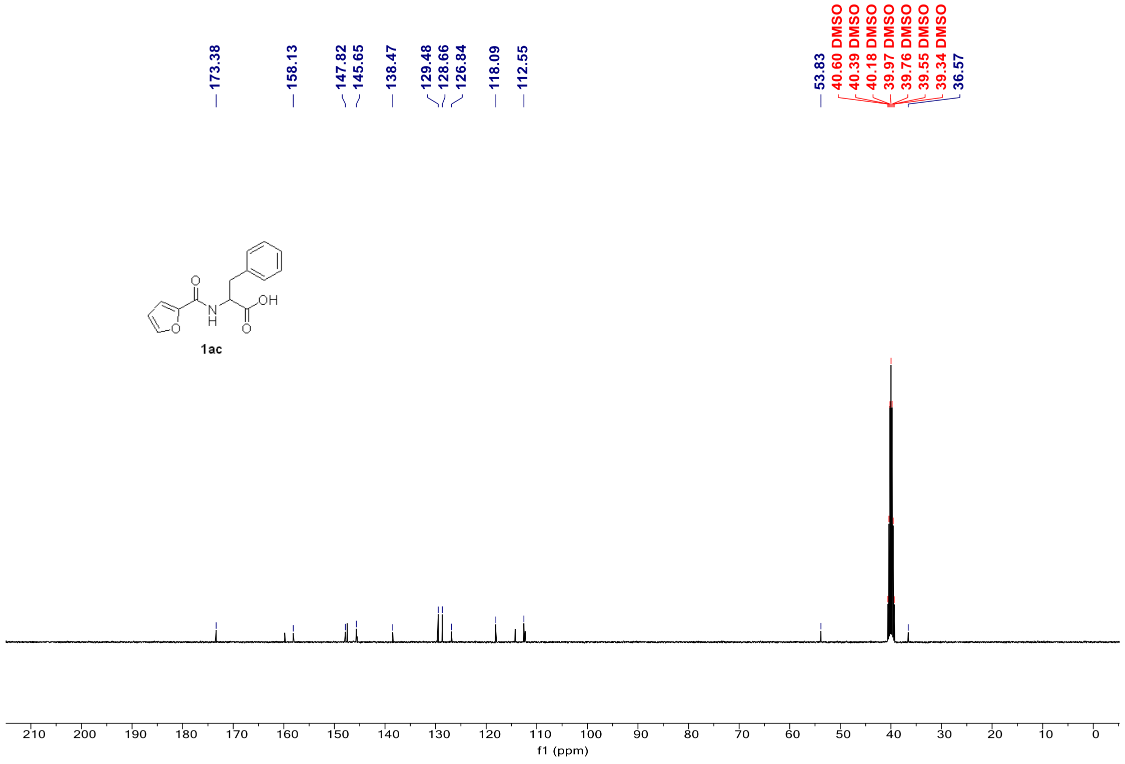

Figure S209. ^{13}**C NMR** of the **1ac** (101 MHz, DMSO-d_6)

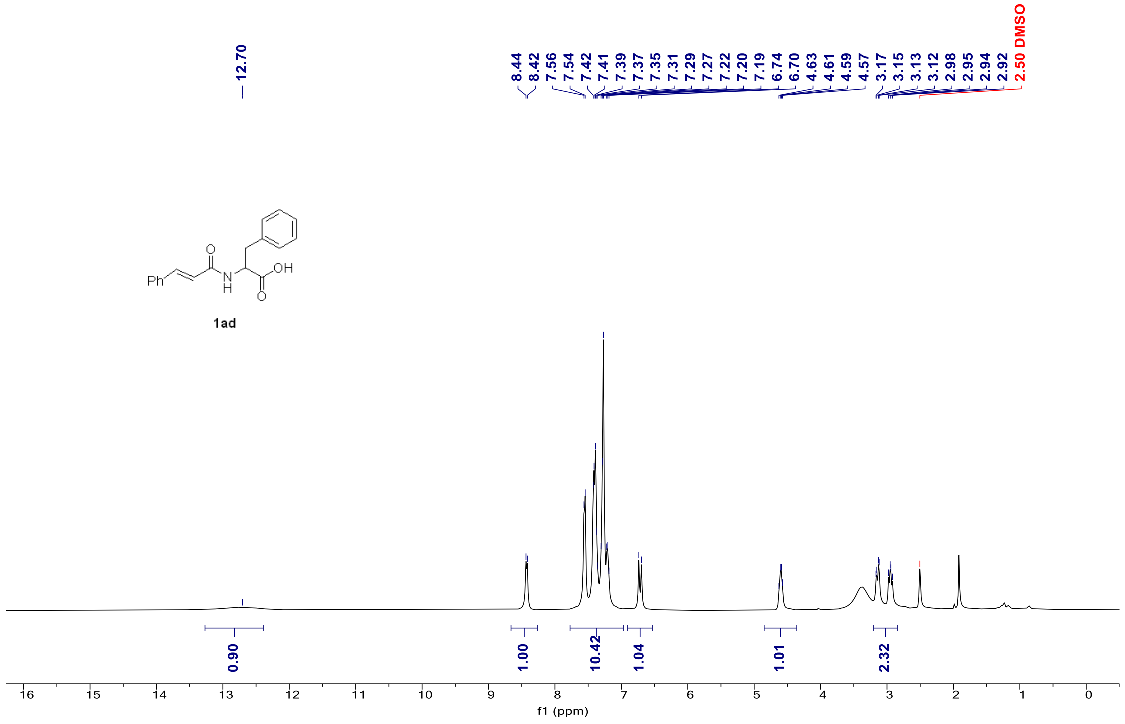

Figure S210. **^{1}H NMR** of the **1ad** (400 MHz, DMSO-*d*$_6$)

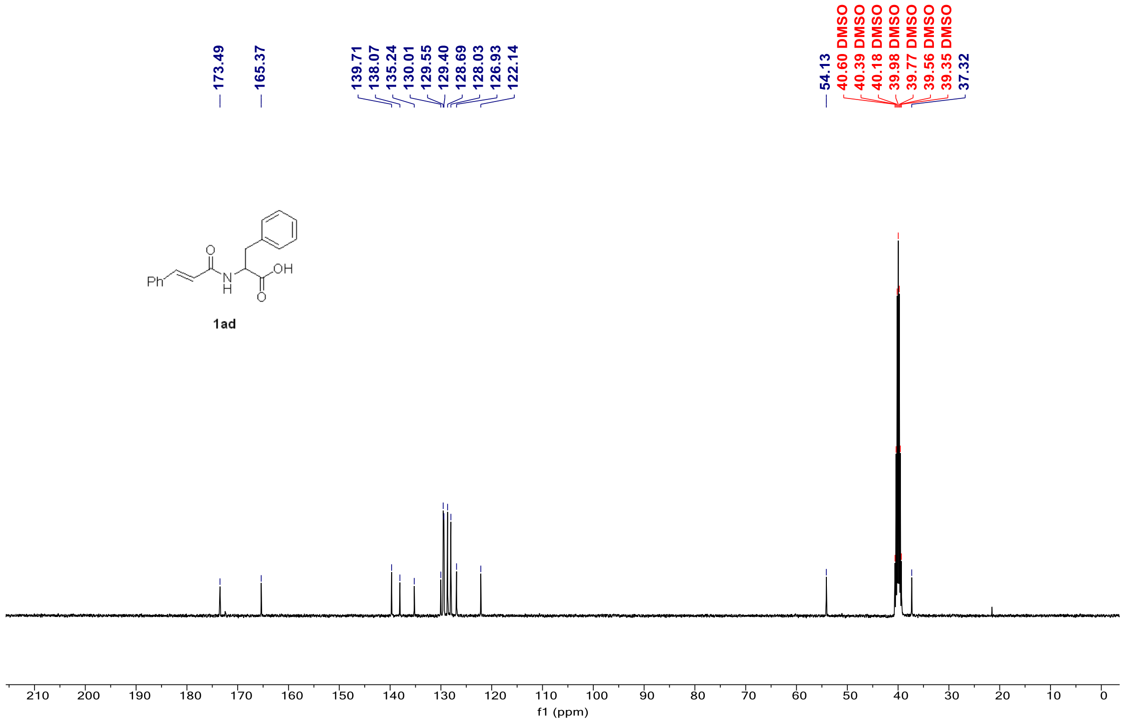

Figure S211. **^{13}C NMR** of the **1ad** (101 MHz, DMSO-*d*$_6$)

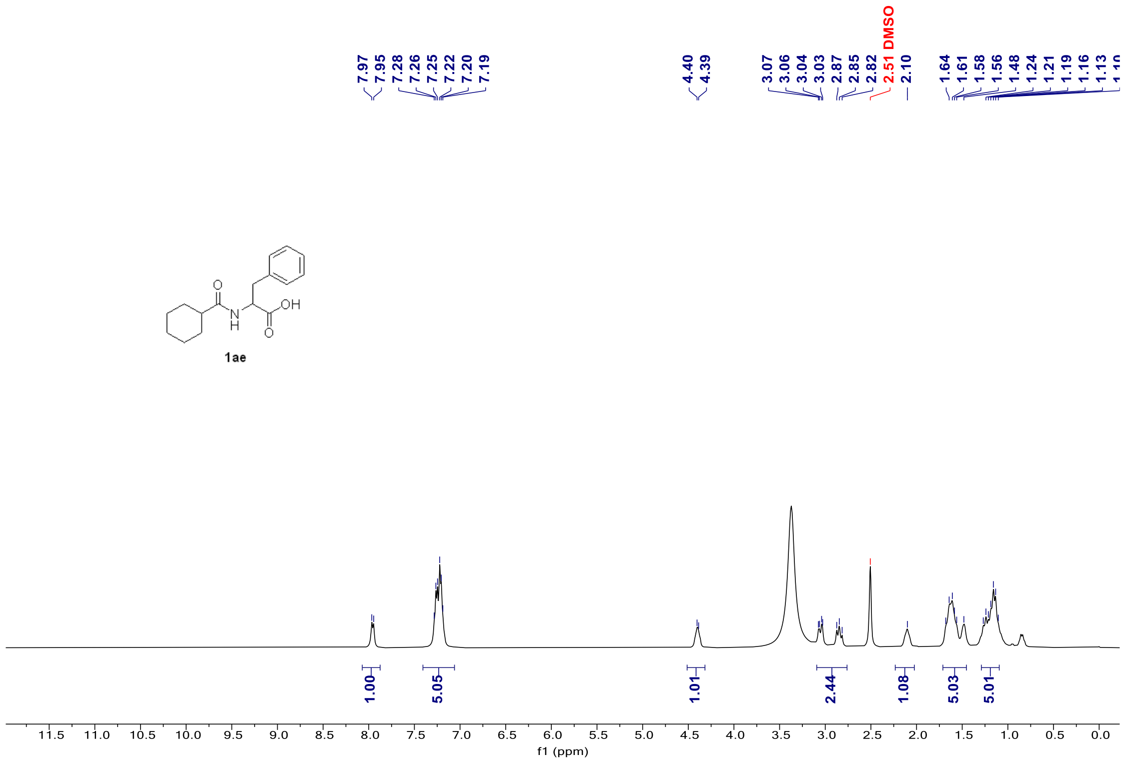

Figure S212. ^{1}H NMR of the **1ae** (400 MHz, DMSO-d_6**)**

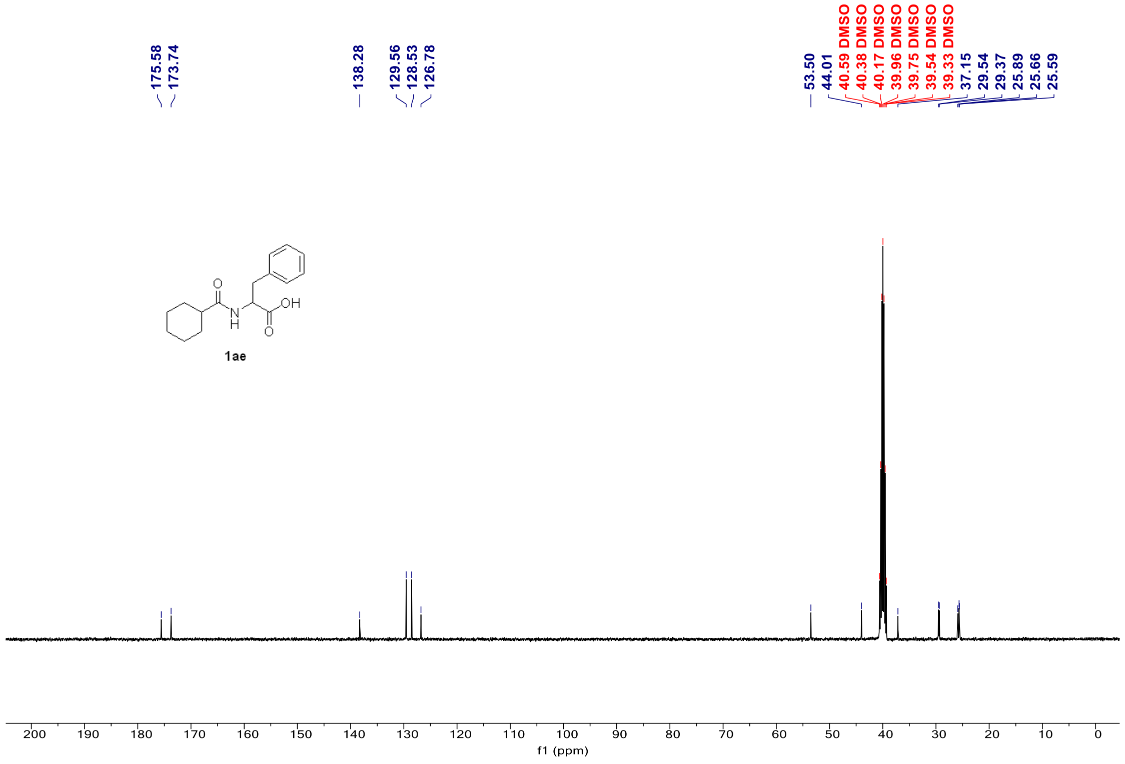

Figure S213. ^{13}C NMR of the **1ae** (101 MHz, DMSO-d_6**)**

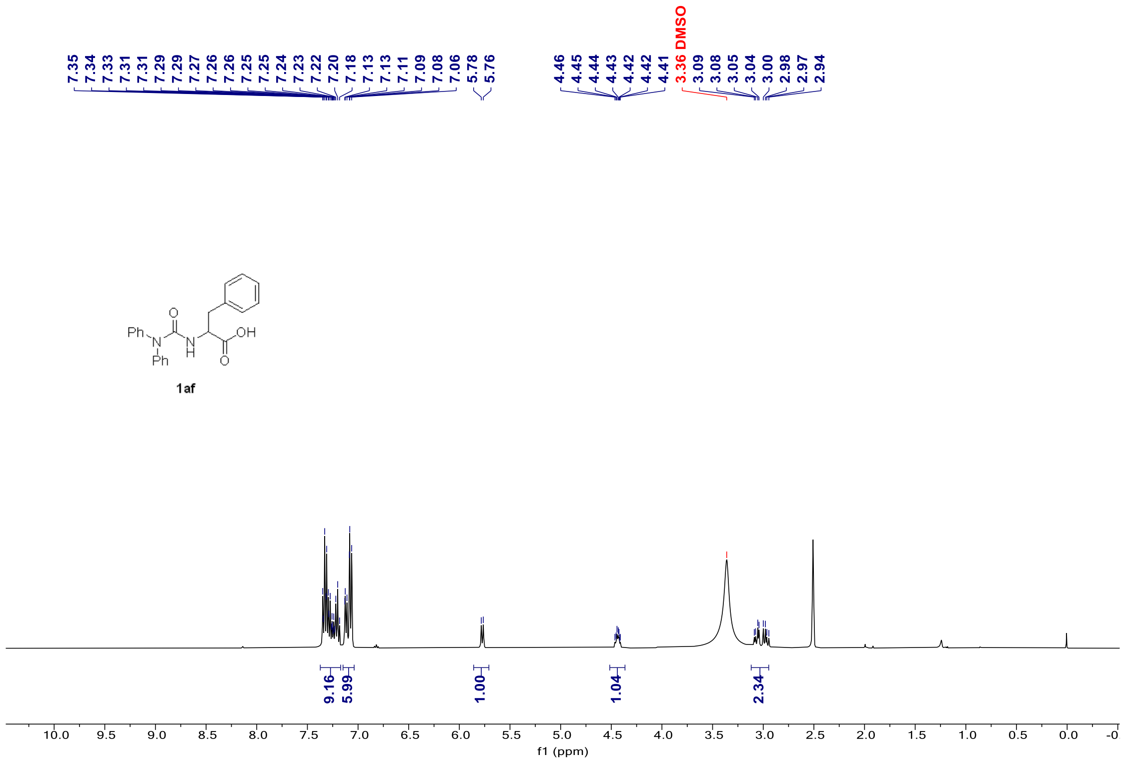

Figure S214. **^{1}H NMR** of the **1af** (400 MHz, DMSO-d_6)

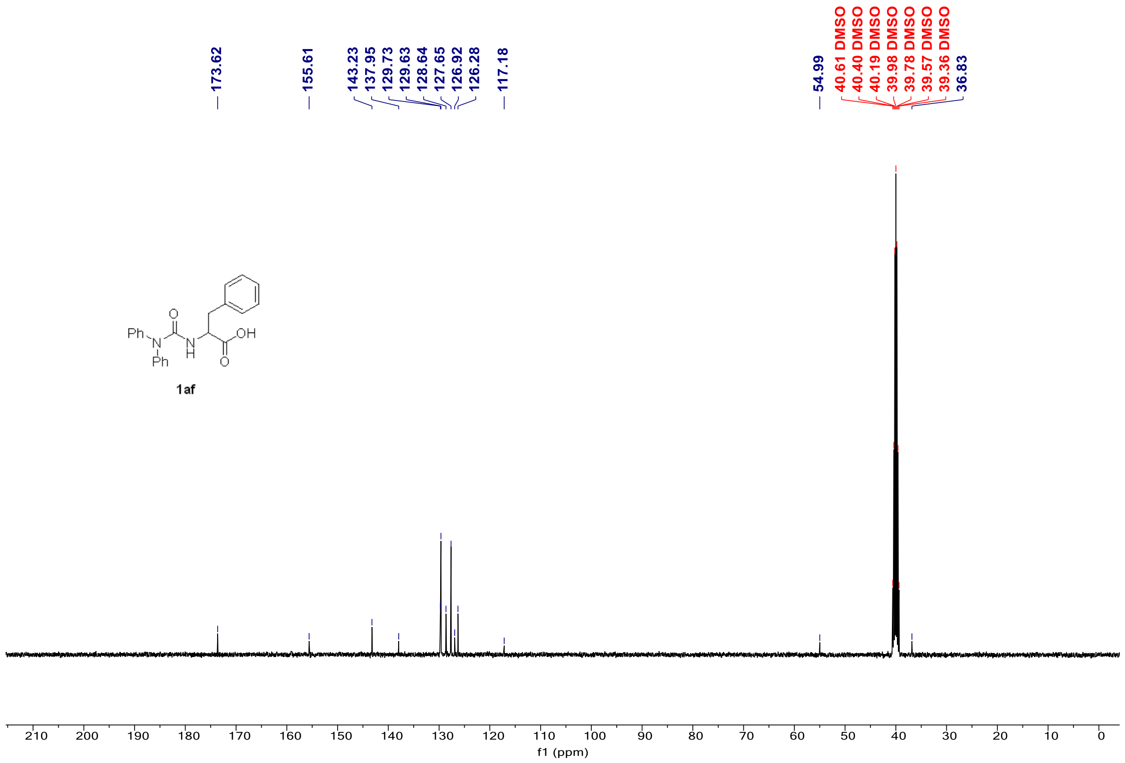

Figure S215. **^{13}C NMR** of the **1af** (101 MHz, DMSO-d_6)

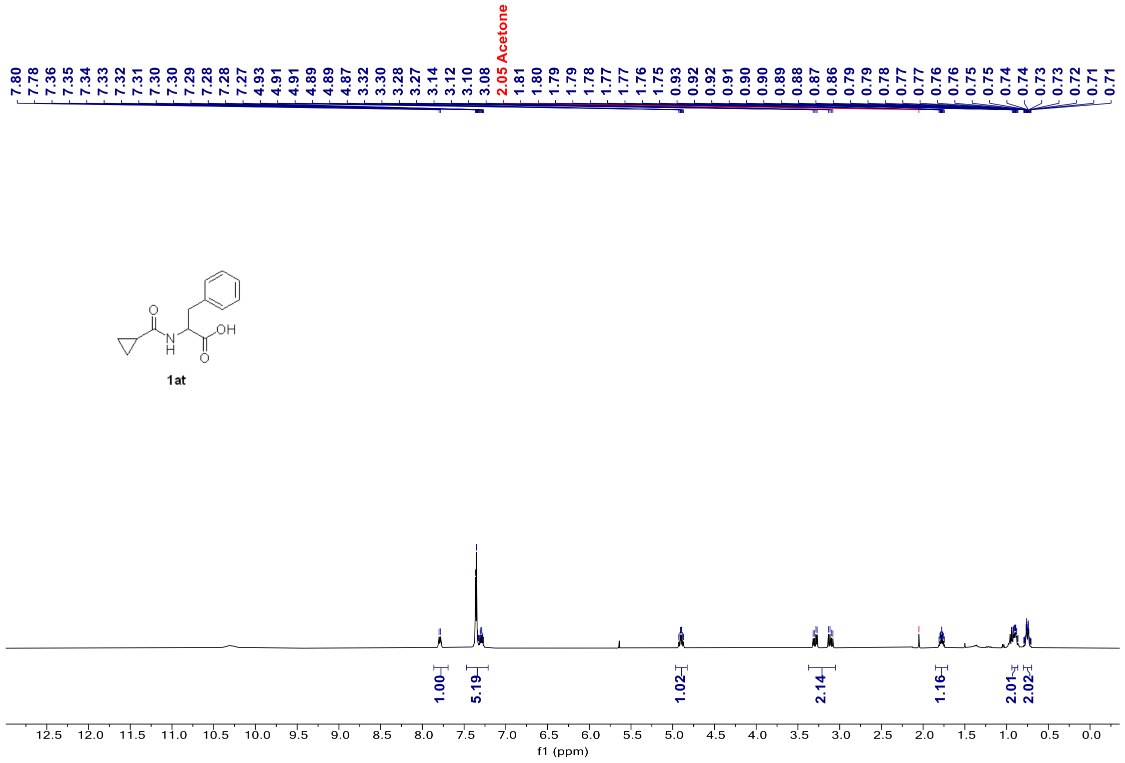

Figure S216. **^{1}H NMR** of the **1at** (400 MHz, Acetone-*d*$_6$)

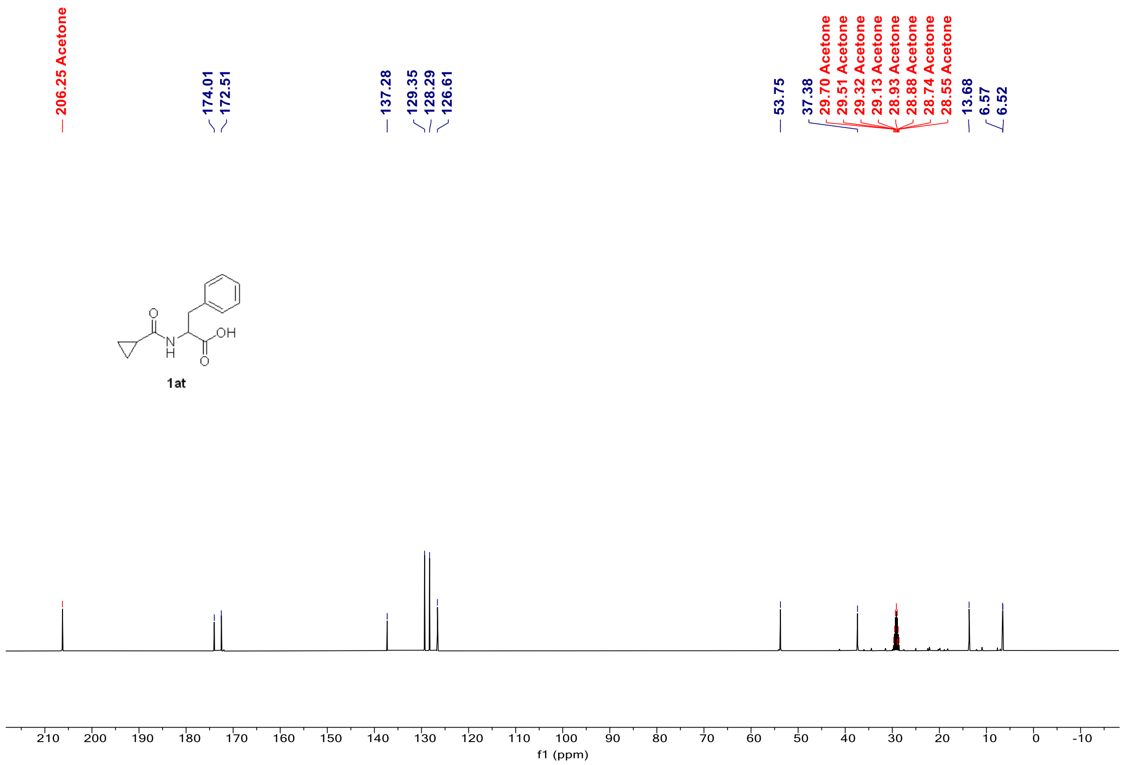

Figure S217. **^{13}C NMR** of the **1at** (101 MHz, Acetone-*d*$_6$)

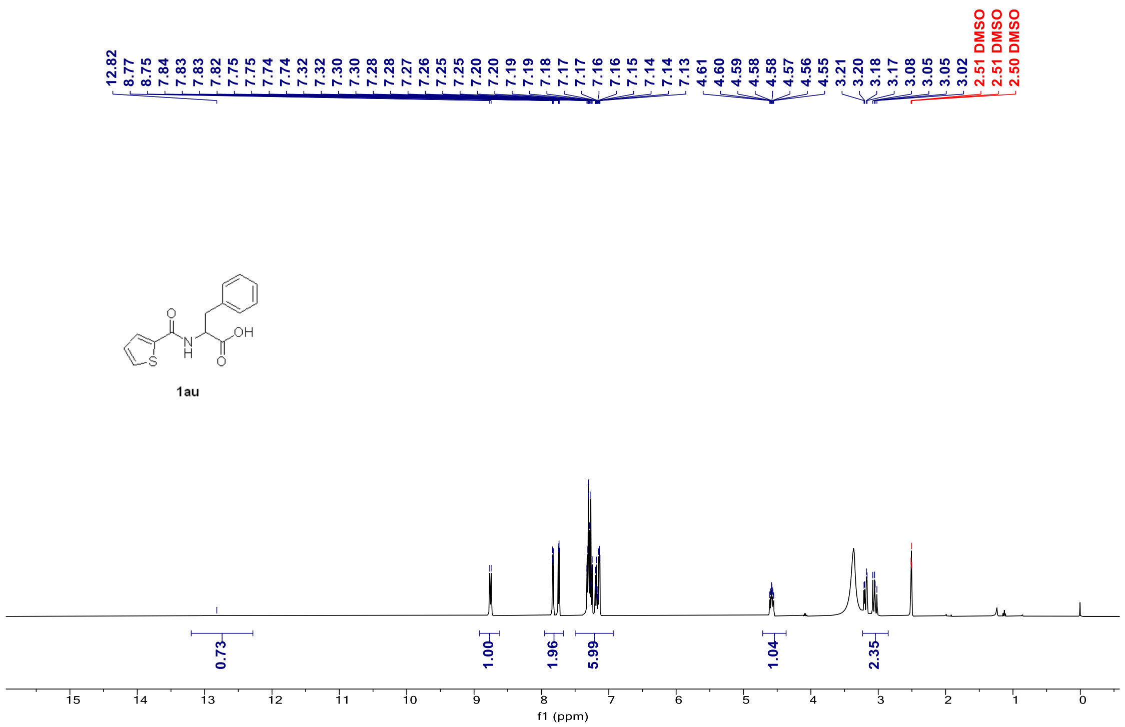

Figure S218. ^{1}H NMR of the **1au** (400 MHz, DMSO-d_6**)**

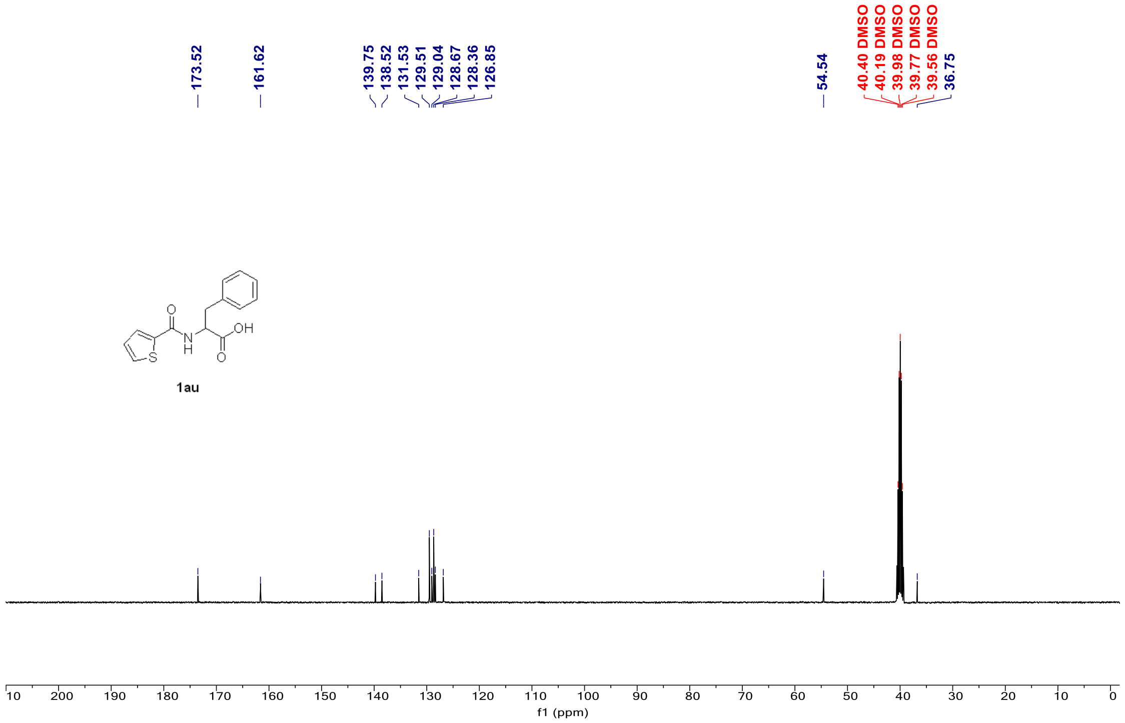

Figure S219. ^{13}C NMR of the **1au** (101 MHz, DMSO-d_6**)**

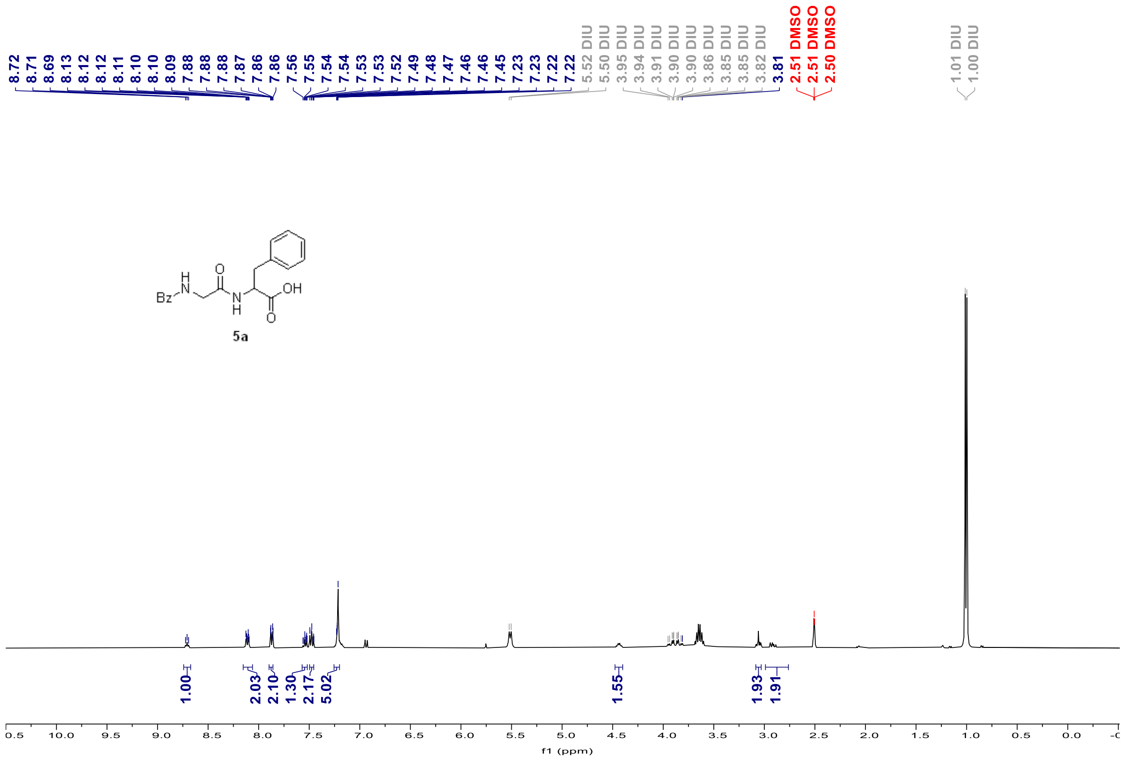

Figure S220. ^{1}**H NMR** of the **5a** (400 MHz, DMSO-d_6)

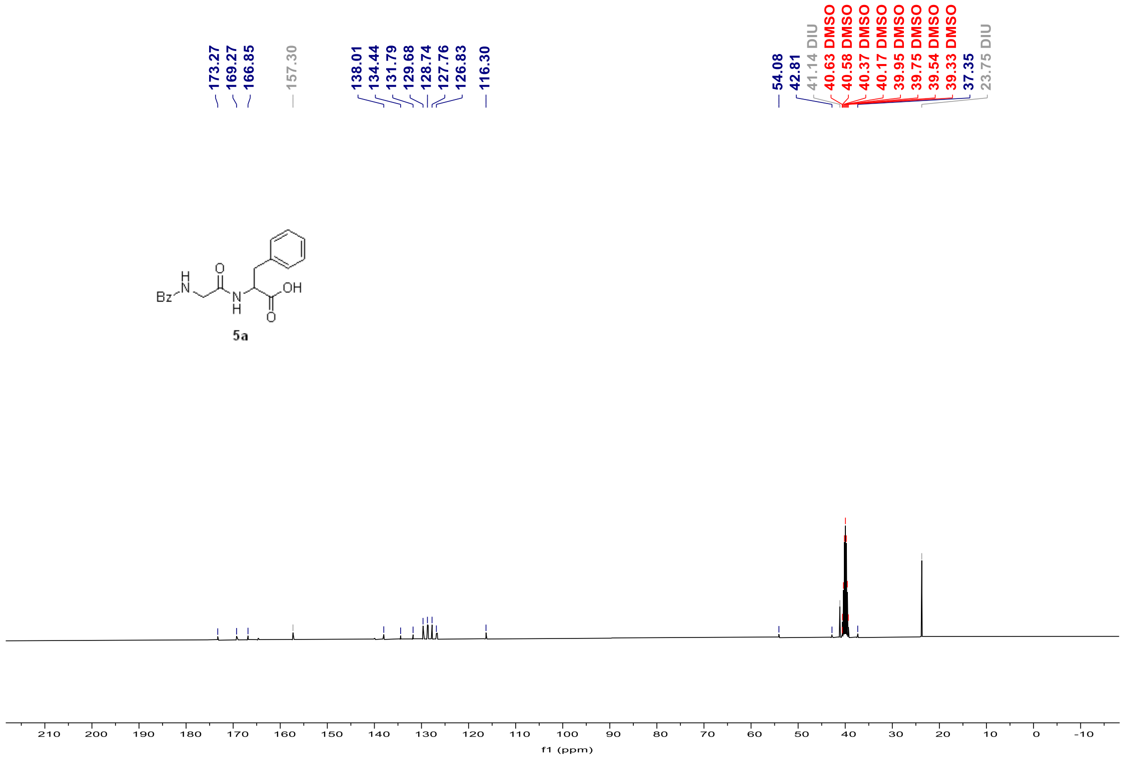

Figure S221. ^{13}**C NMR** of the **5a** (101 MHz, DMSO-d_6)

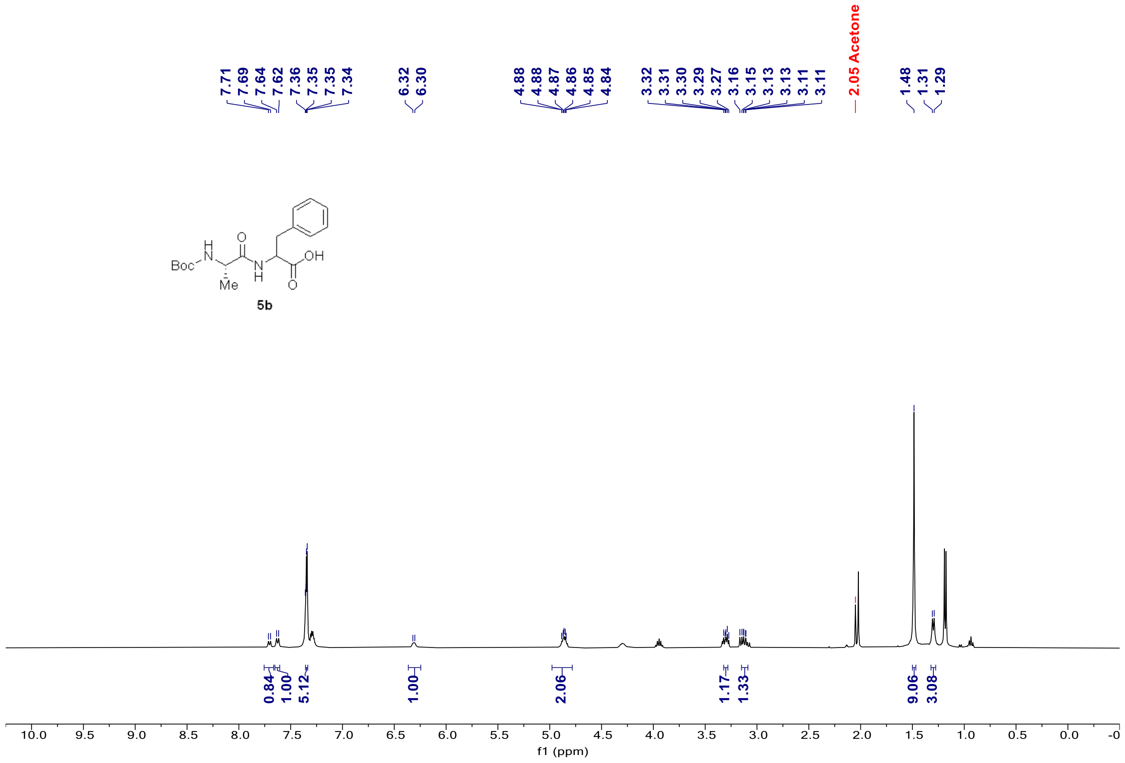

Figure S222. **^{1}H NMR** of the **5b** (400 MHz, Acetone-d_6)

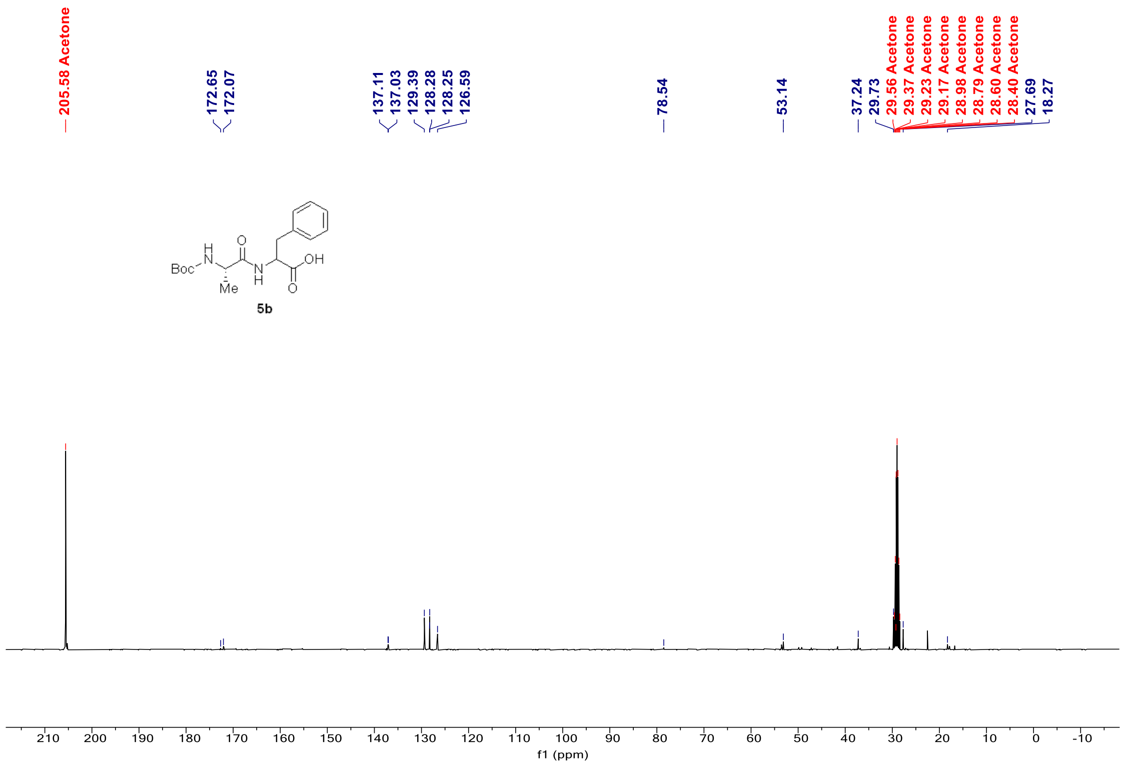

Figure S223. **^{13}C NMR** of the **5b** (101 MHz, Acetone-d_6)

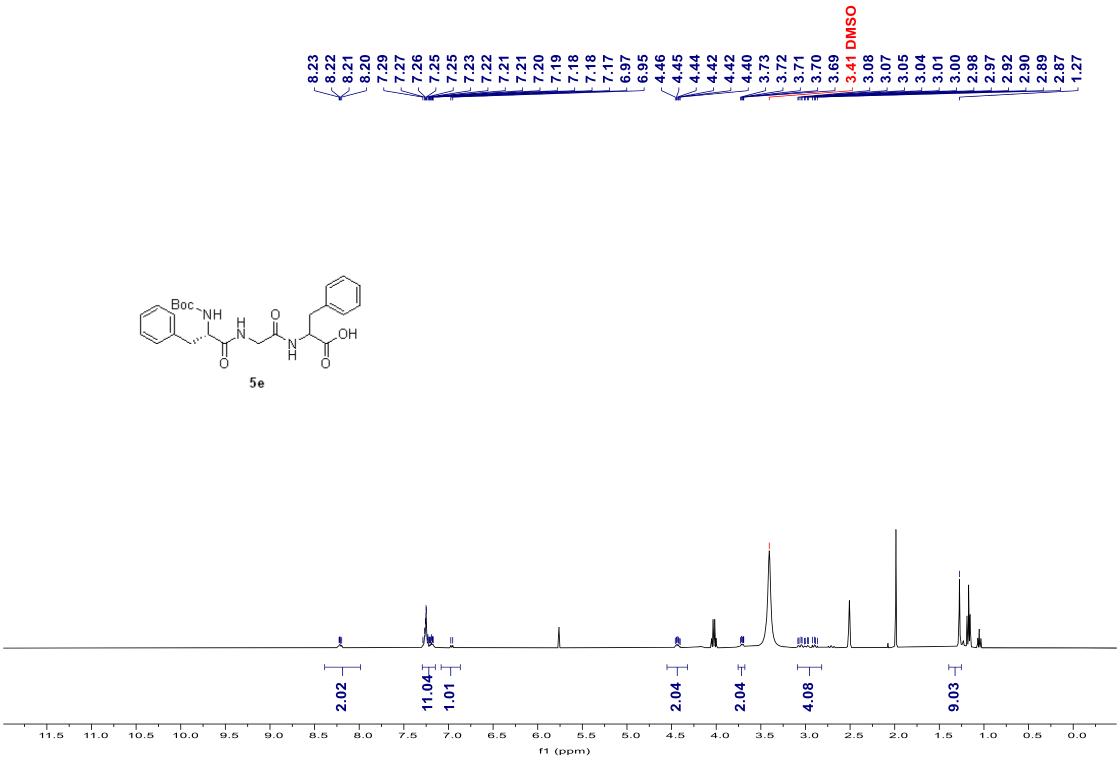

Figure S224. **^{1}H NMR** of the **5e** (400 MHz, DMSO-d_6)

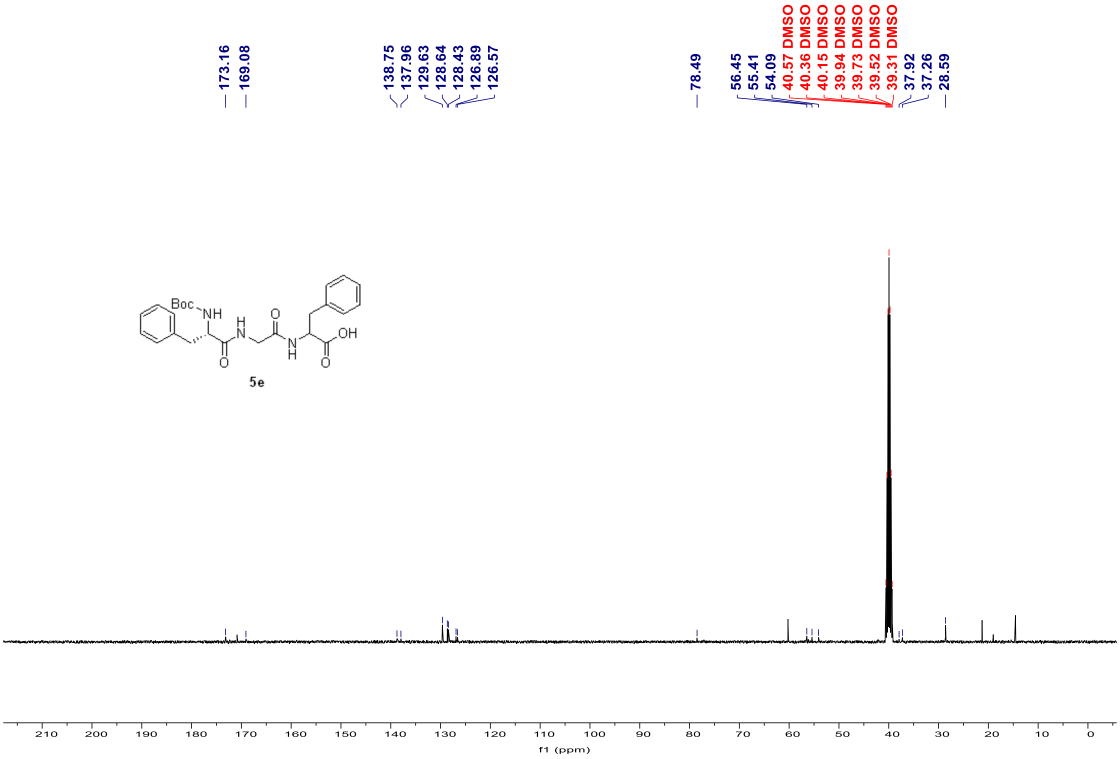

Figure S225. **^{13}C NMR** of the **5e** (101 MHz, DMSO-d_6)

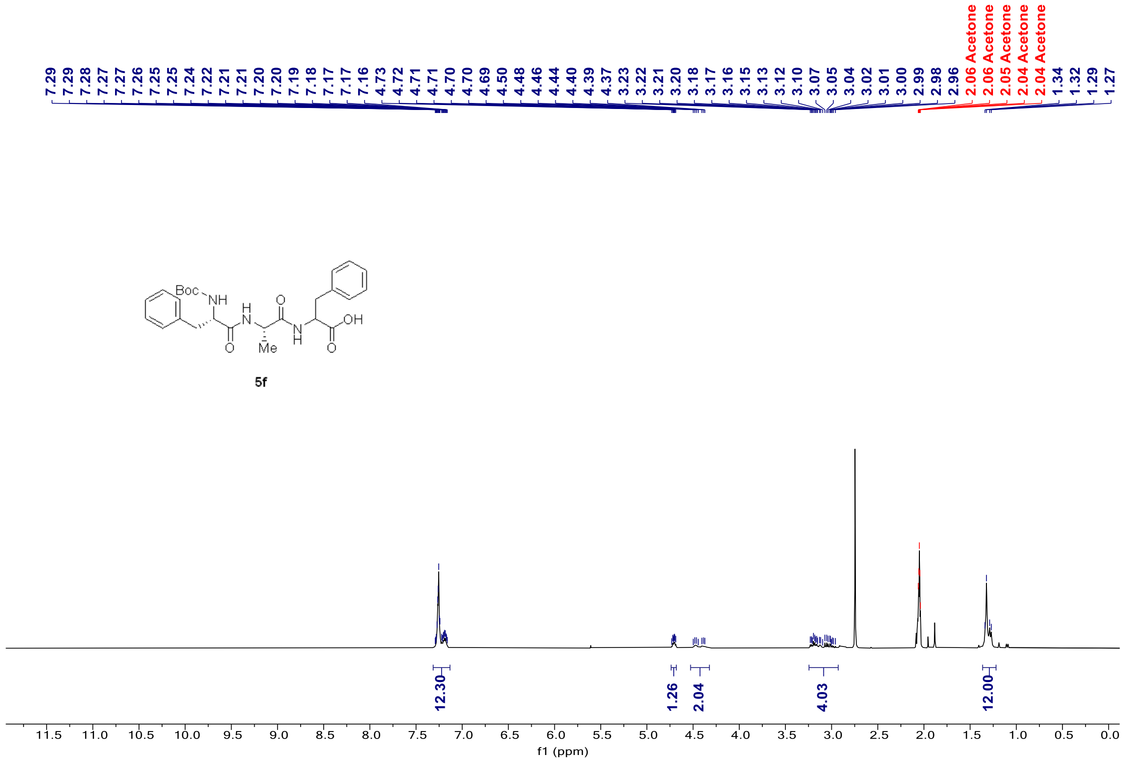

Figure S226. ^{1}H NMR of the **5f** (400 MHz, Acetone-d_6)

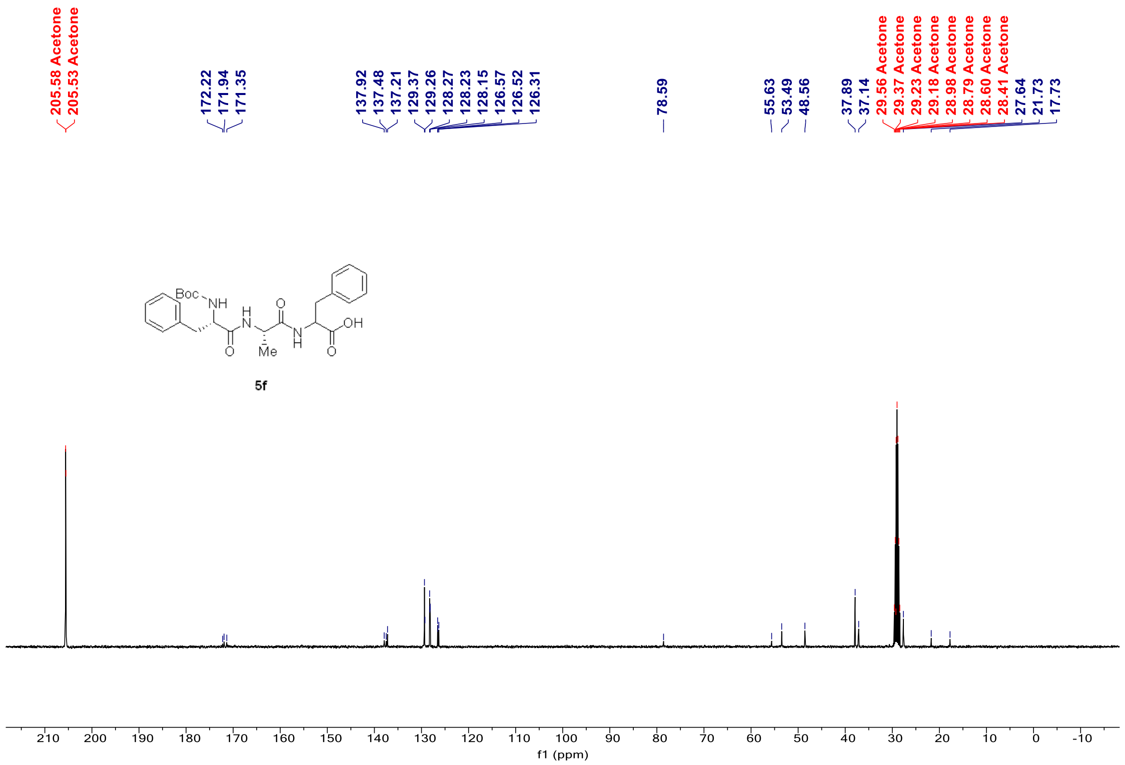

Figure S227. ^{13}C NMR of the **5f** (101 MHz, Acetone-d_6)

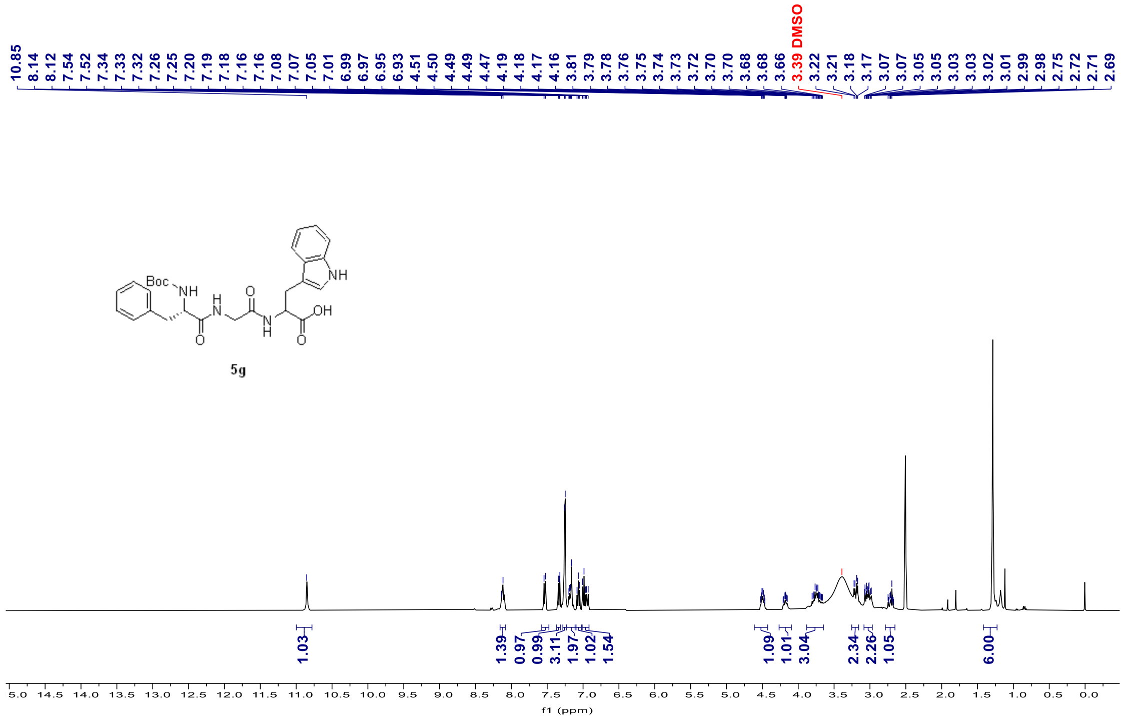

Figure S228. ^{1}**H NMR** of the **5g** (400 MHz, DMSO-d_6)

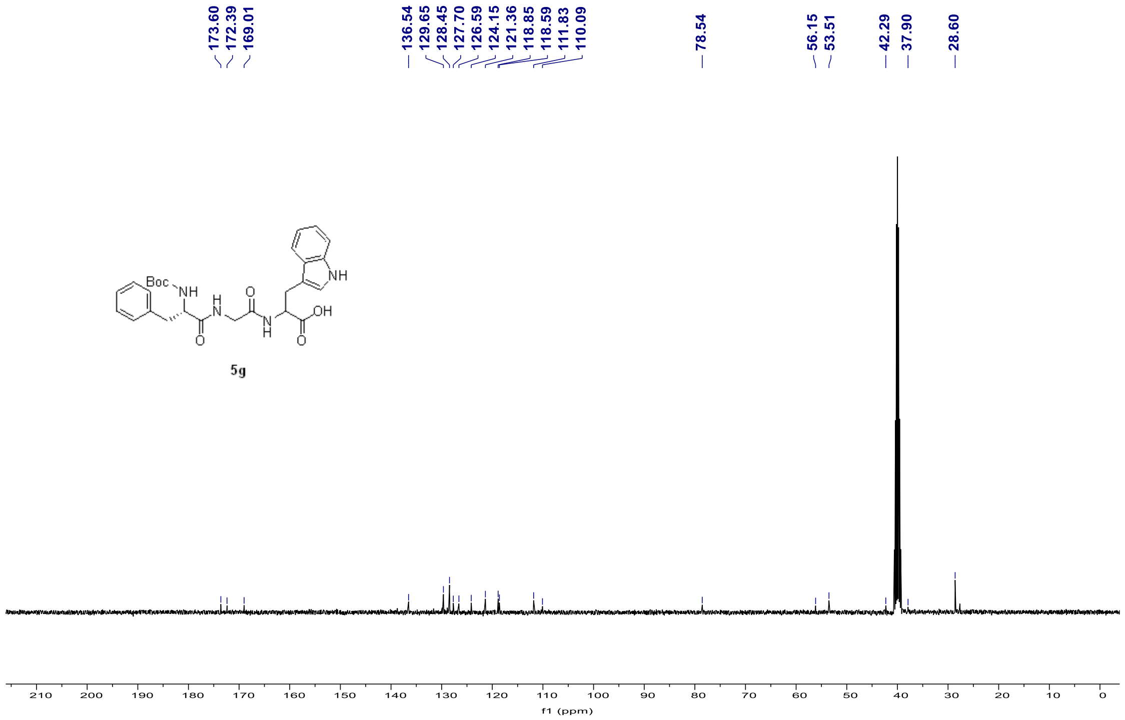

Figure S229. ^{13}**C NMR** of the **5g** (101 MHz, DMSO-d_6)

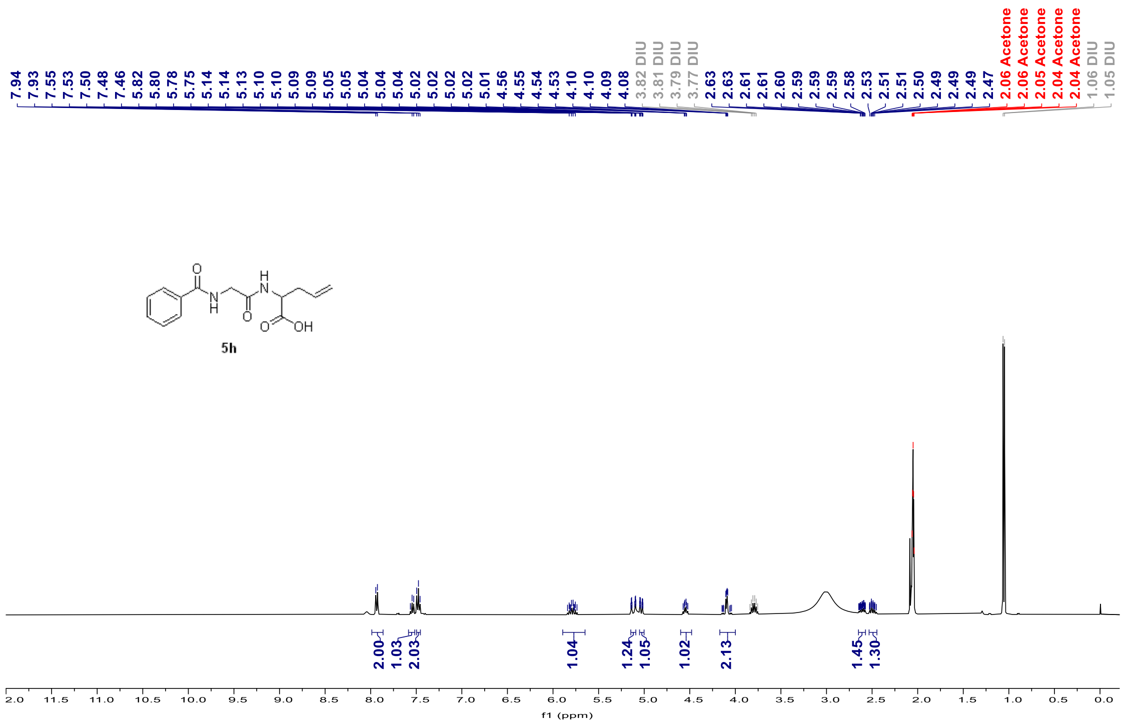

Figure S230. ^{1}H NMR of the **5h** (400 MHz, Acetone-d_6)

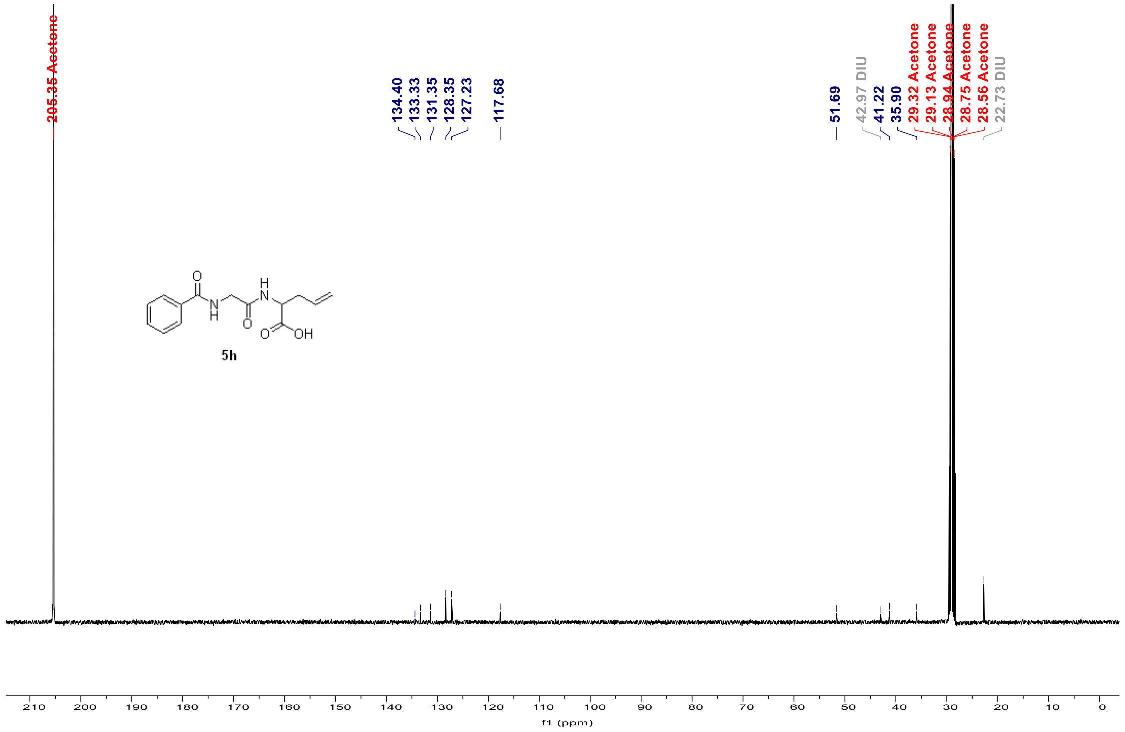

Figure S231. ^{13}C NMR of the **5h** (101 MHz, Acetone-d_6)

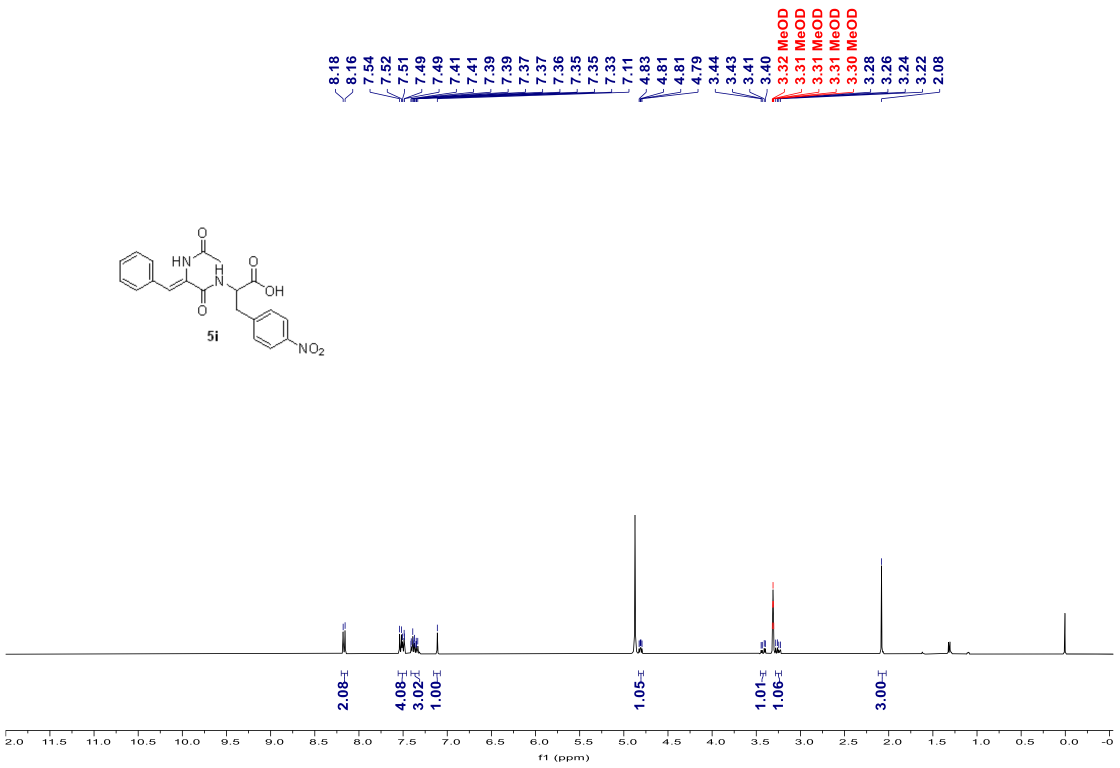

Figure S232. **^{1}H NMR** of the **5i** (400 MHz, Methanol-d_4)

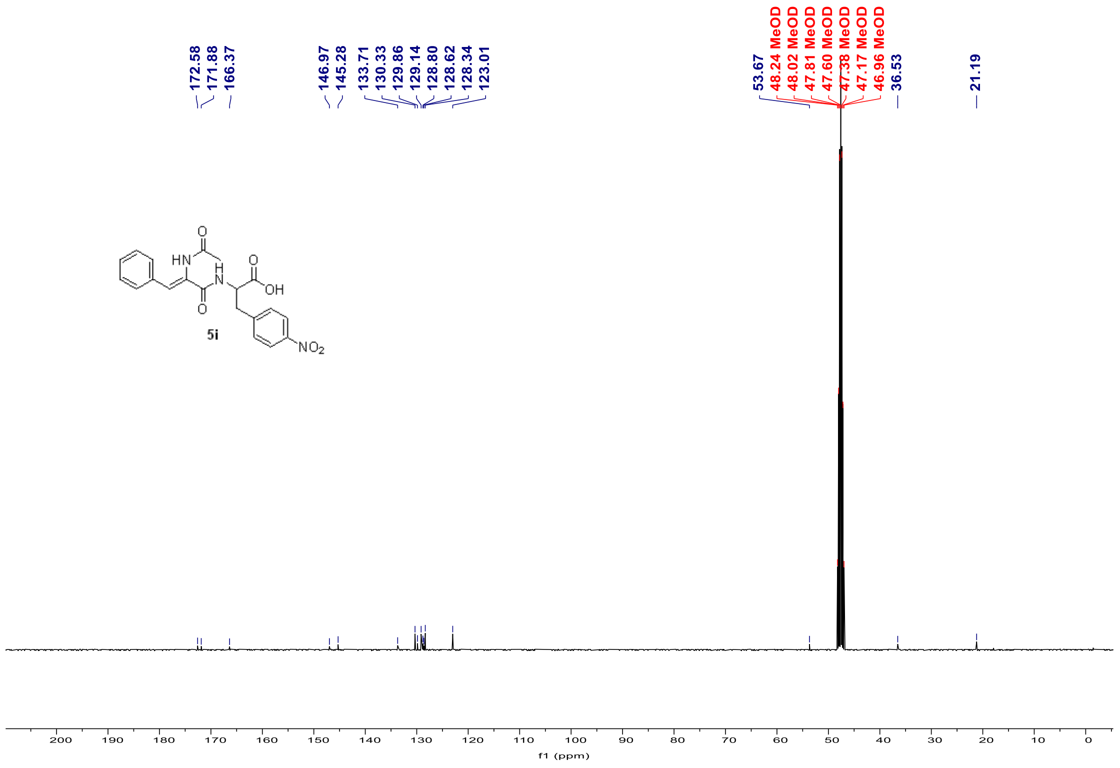

Figure S233. **^{13}C NMR** of the **5i** (101 MHz, Methanol-d_4)

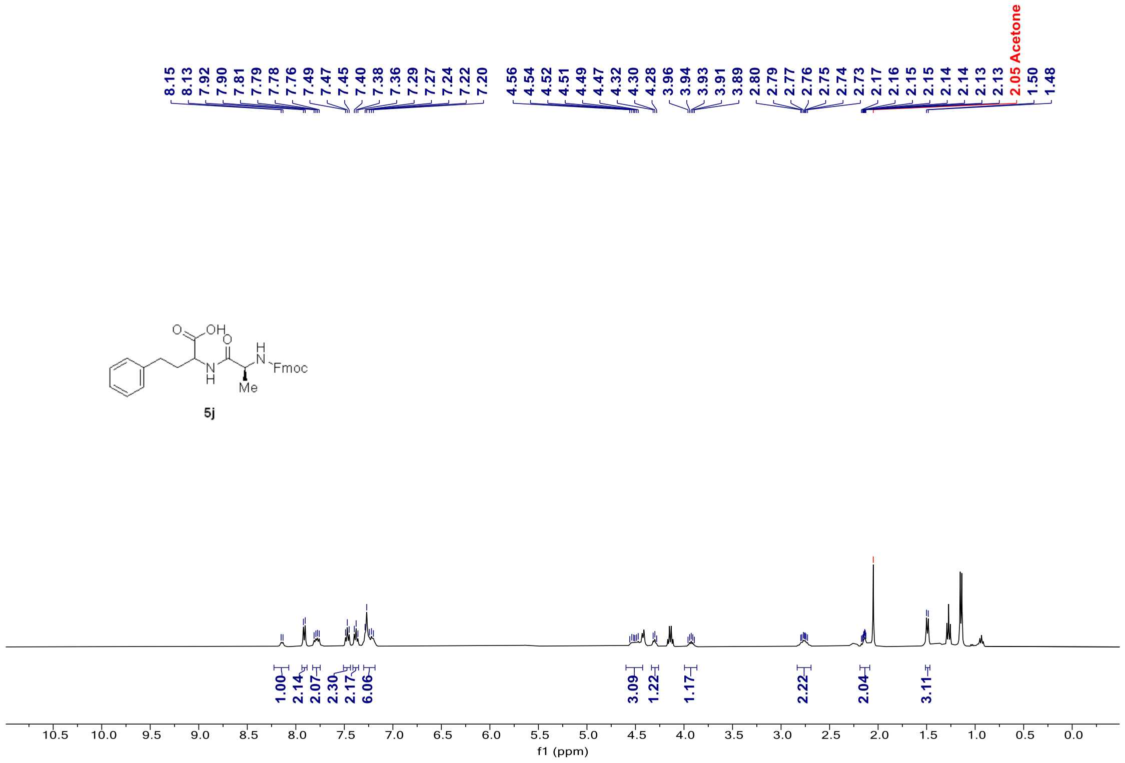

Figure S234. ^{1}H NMR of the **5j** (400 MHz, Acetone-*d*$_6$)

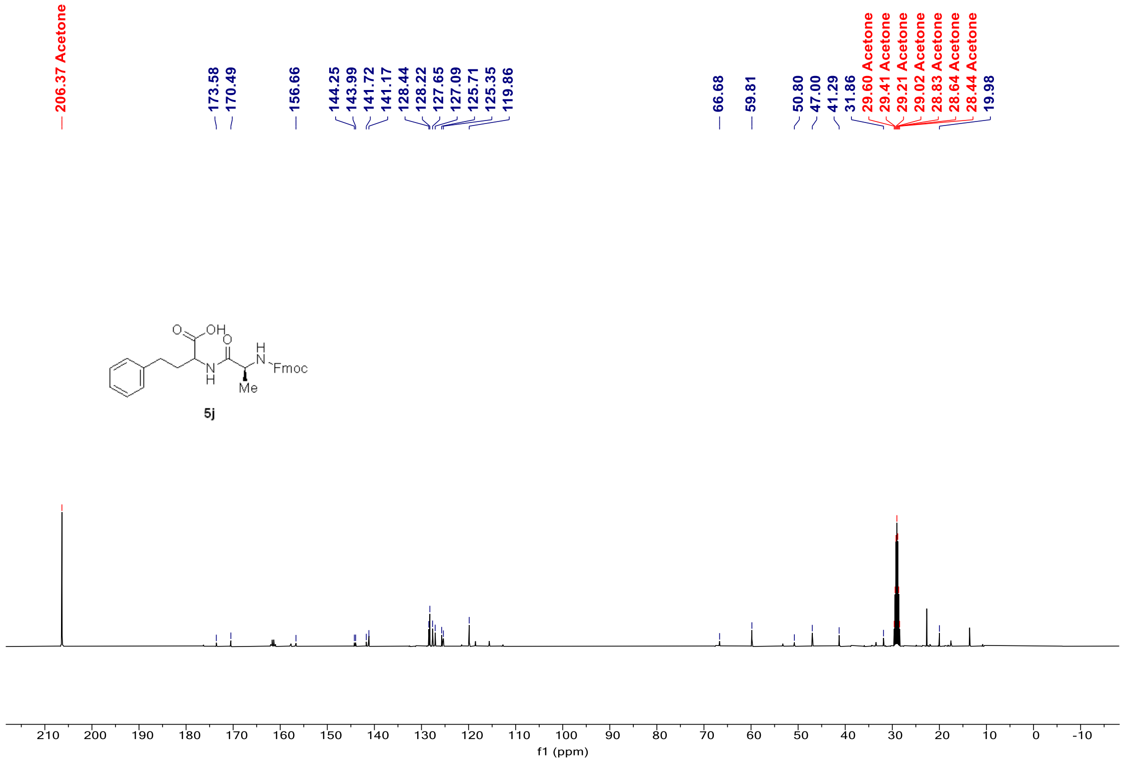

Figure S235. ^{13}C NMR of the **5j** (101 MHz, Acetone-*d*$_6$)

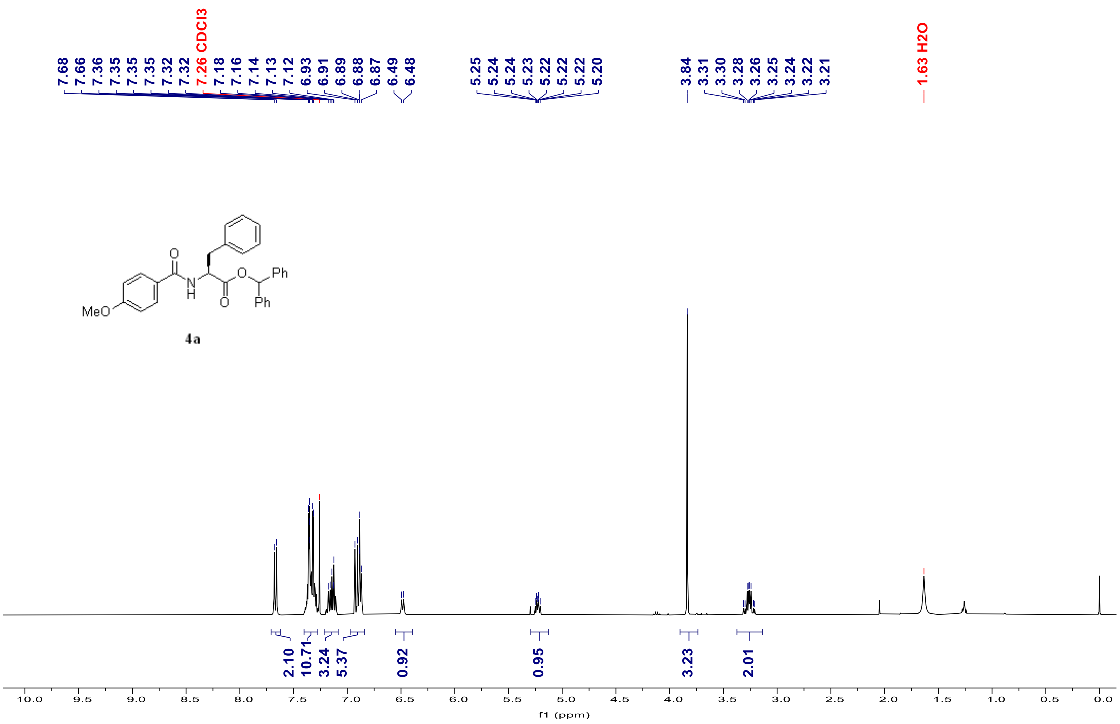

Figure S236. ^{1}H NMR of the **4a** (400 MHz, $CDCl_3$**)**

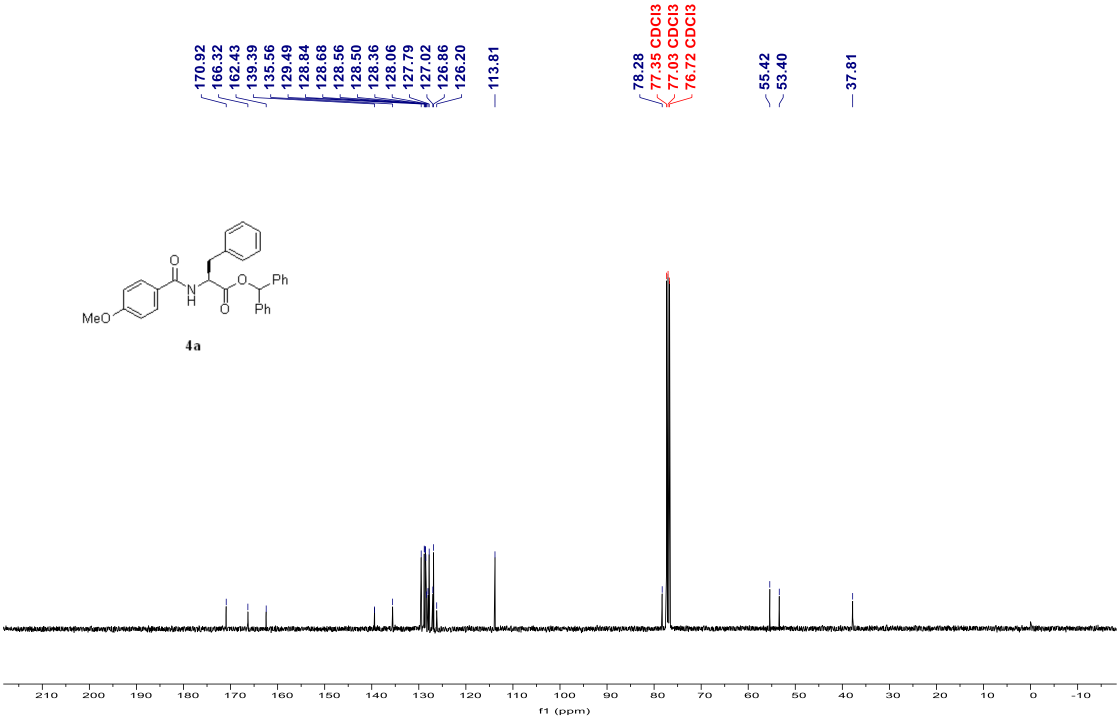

Figure S237. ^{13}C NMR of the **4a** (101 MHz, $CDCl_3$**)**

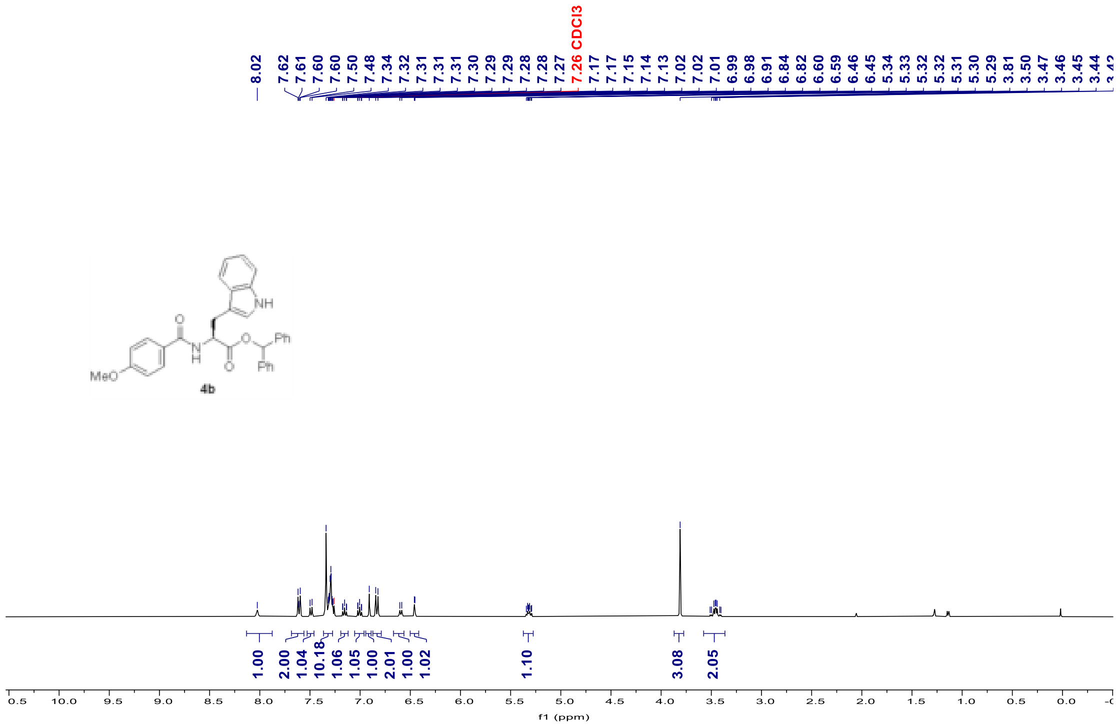

Figure S238. ^{1}H NMR of the **4b** (400 MHz, $CDCl_3$**)**

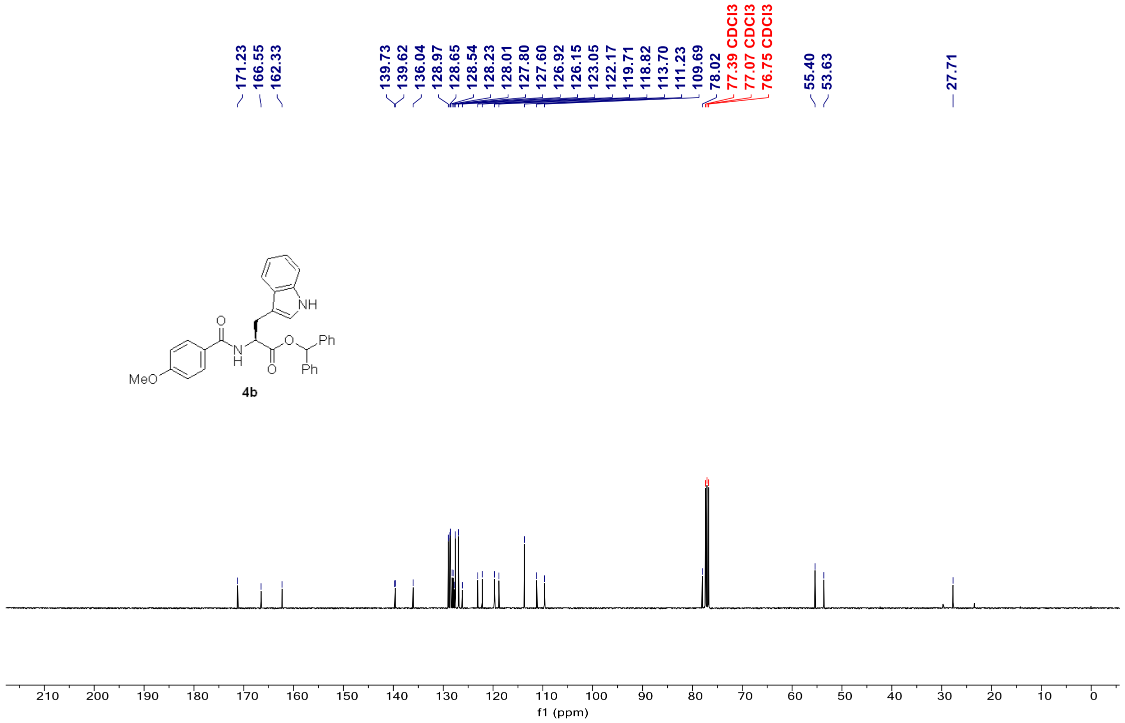

Figure S239. ^{13}C NMR of the **4b** (101 MHz, $CDCl_3$**)**

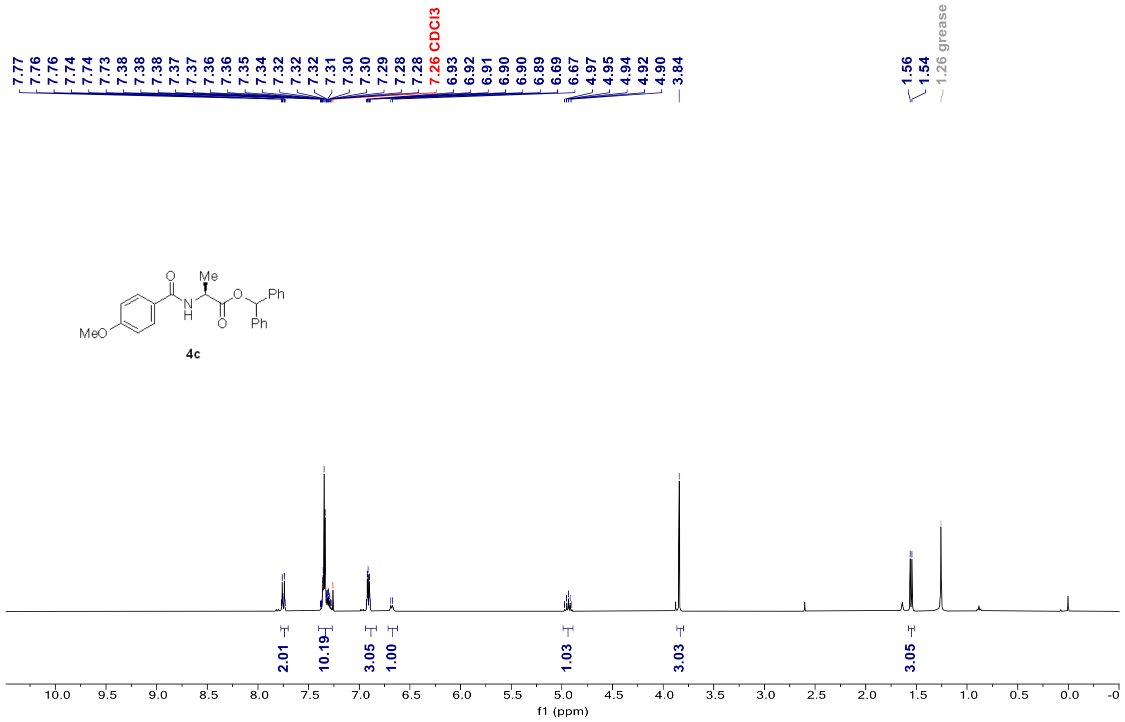

Figure S240. ^{1}**H NMR** of the **4c** (400 MHz, $CDCl_3$)

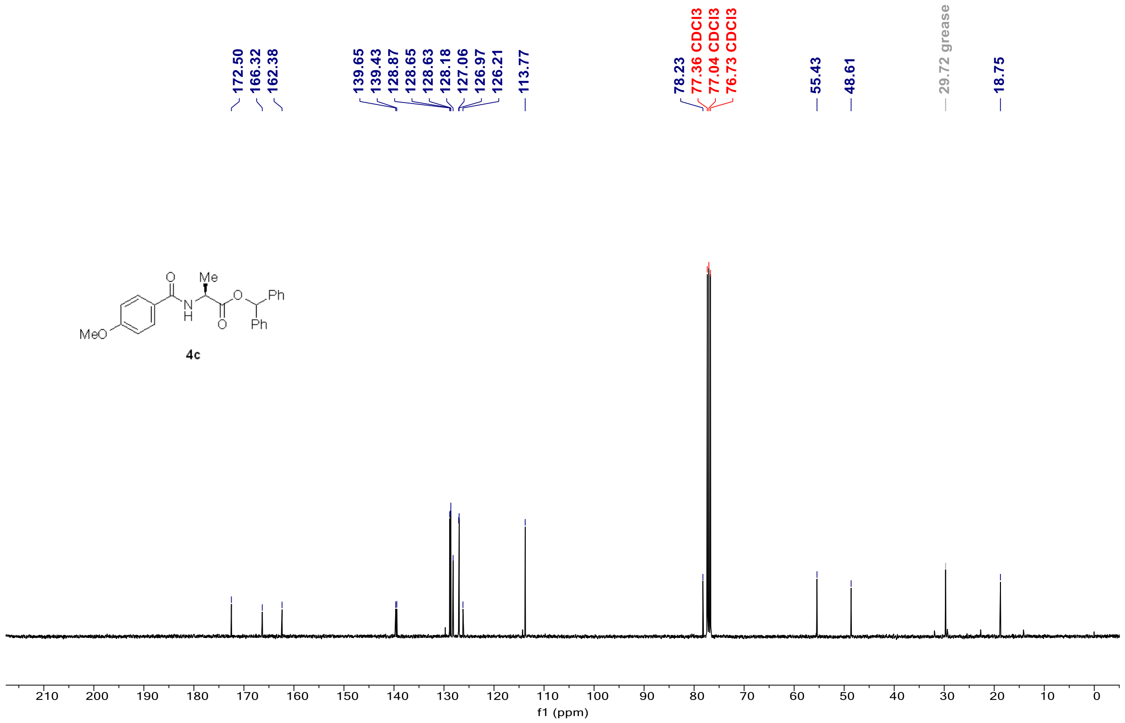

Figure S241. ^{13}**C NMR** of the **4c** (101 MHz, $CDCl_3$)

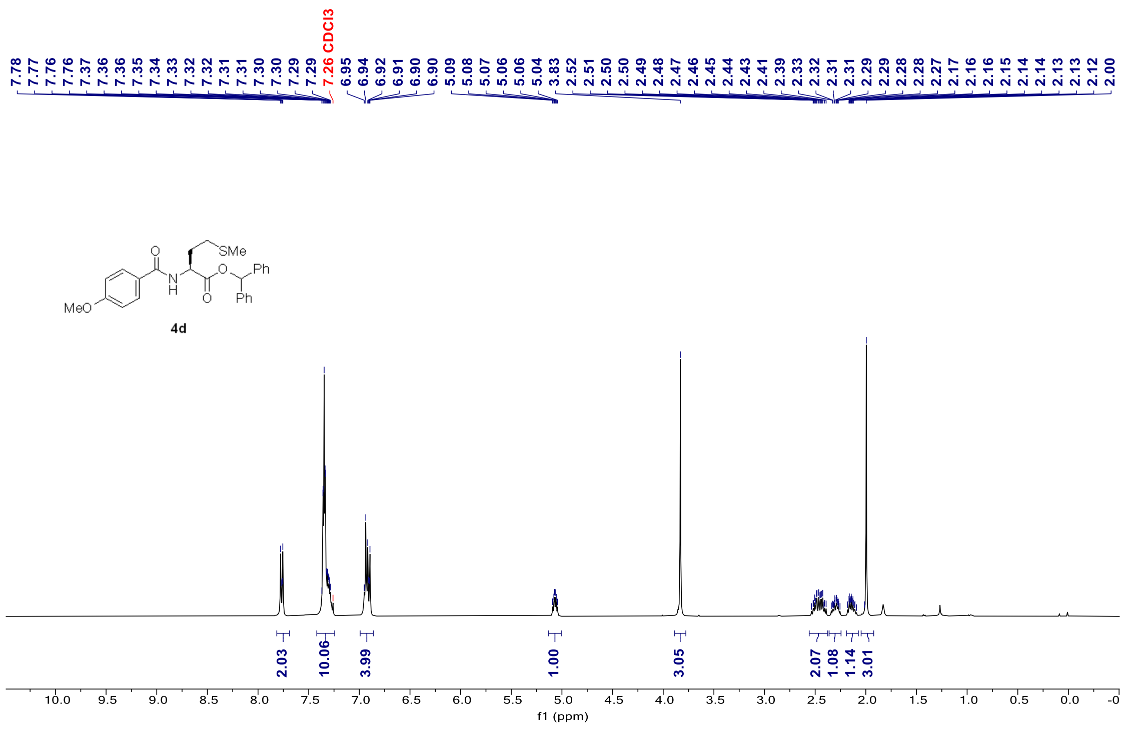

Figure S242. **^{1}H NMR** of the **4d** (400 MHz, $CDCl_3$)

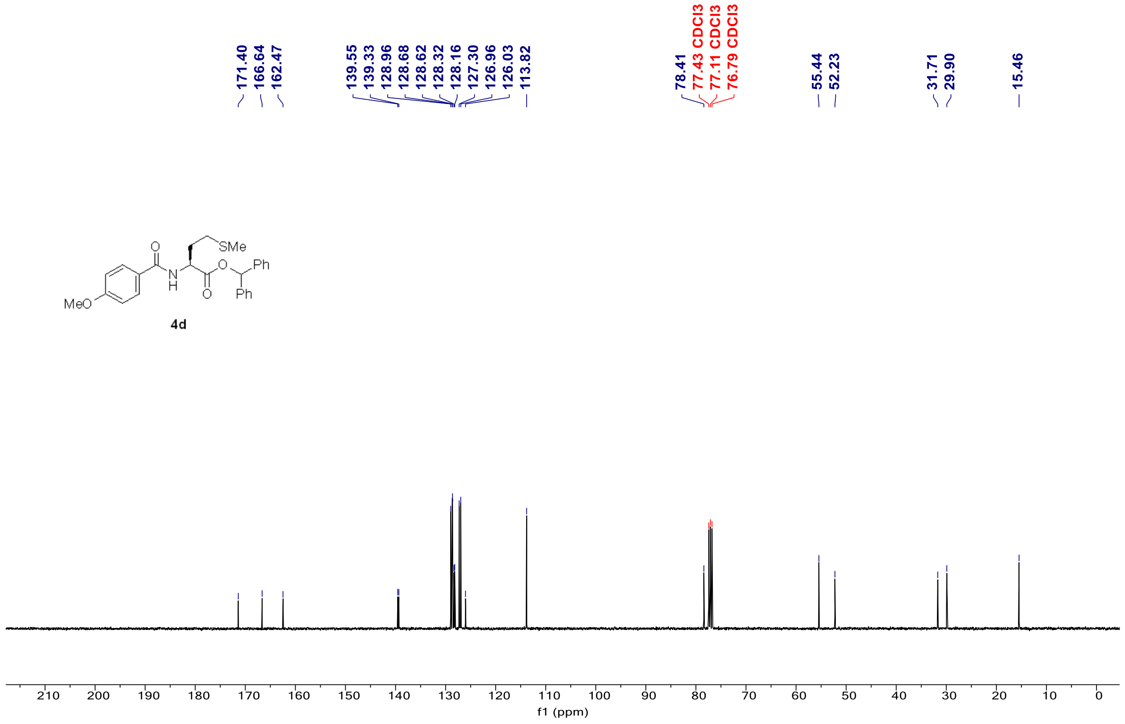

Figure S243. **^{13}C NMR** of the **4d** (101 MHz, $CDCl_3$)

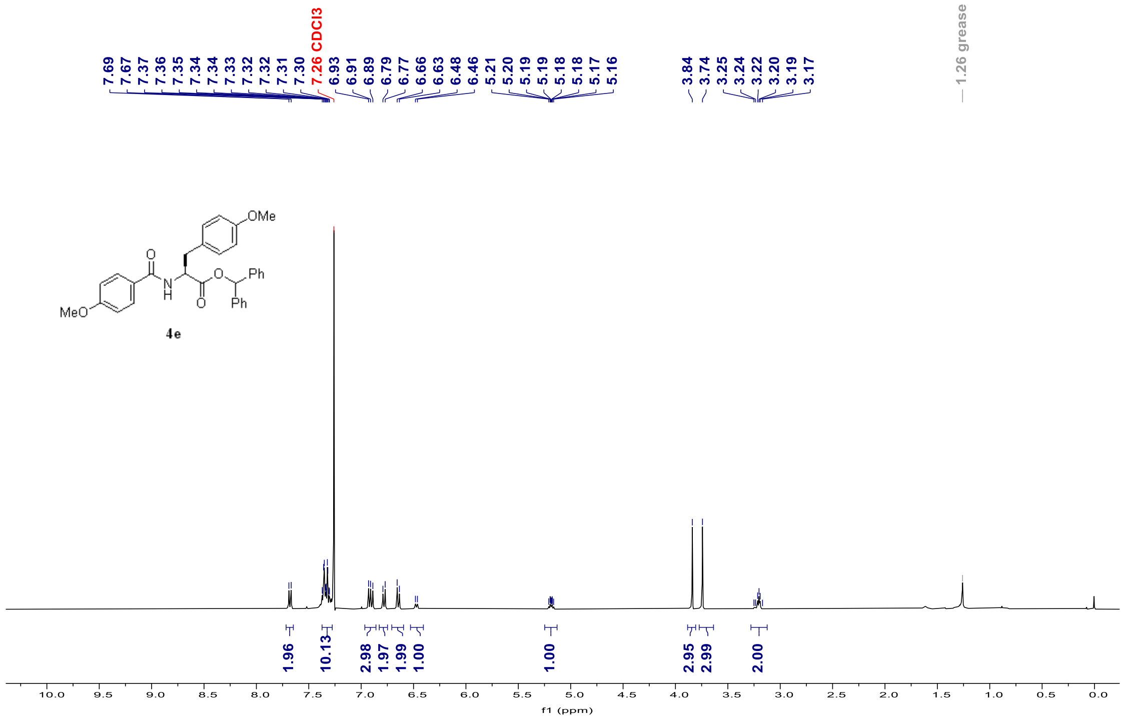

Figure S244. **^{1}H NMR** of the **4e** (400 MHz, $CDCl_3$)

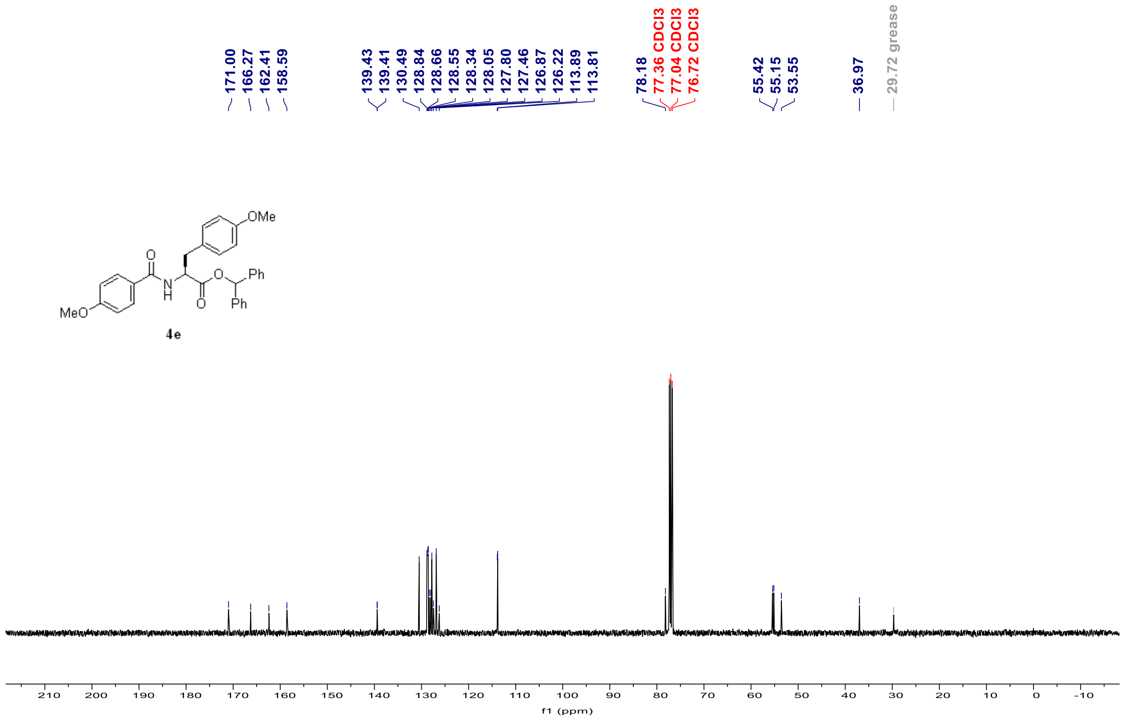

Figure S245. **^{13}C NMR** of the **4e** (101 MHz, $CDCl_3$)

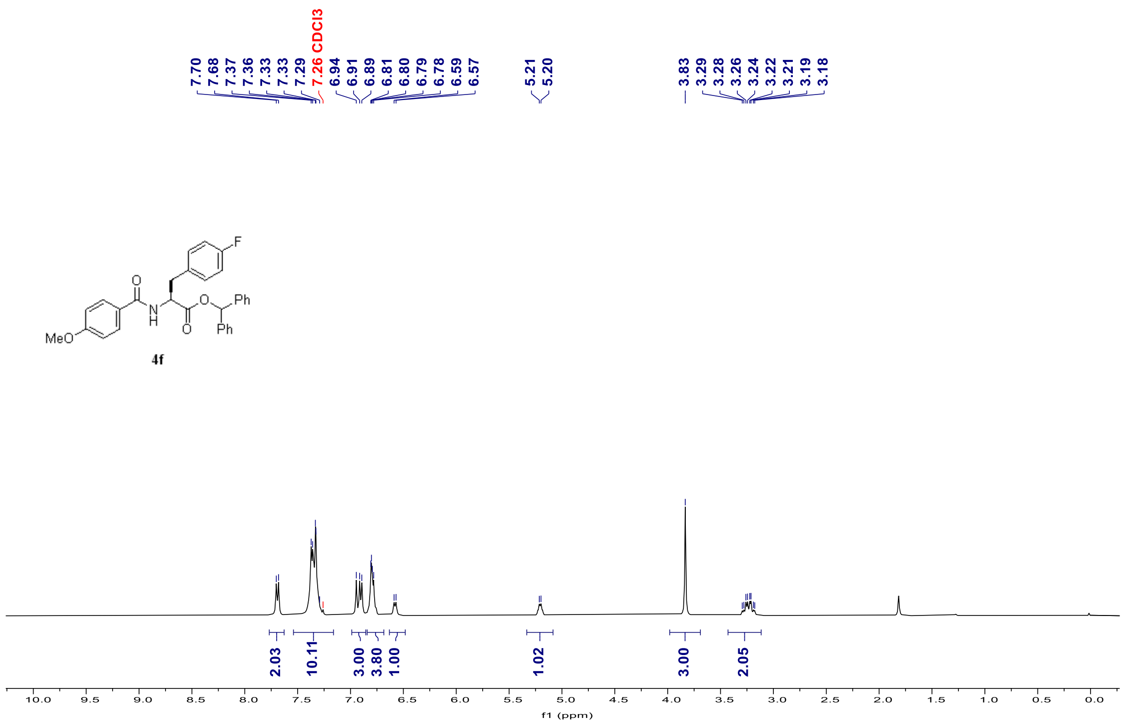

Figure S246. **^{1}H NMR** of the **4f** (400 MHz, $CDCl_3$)

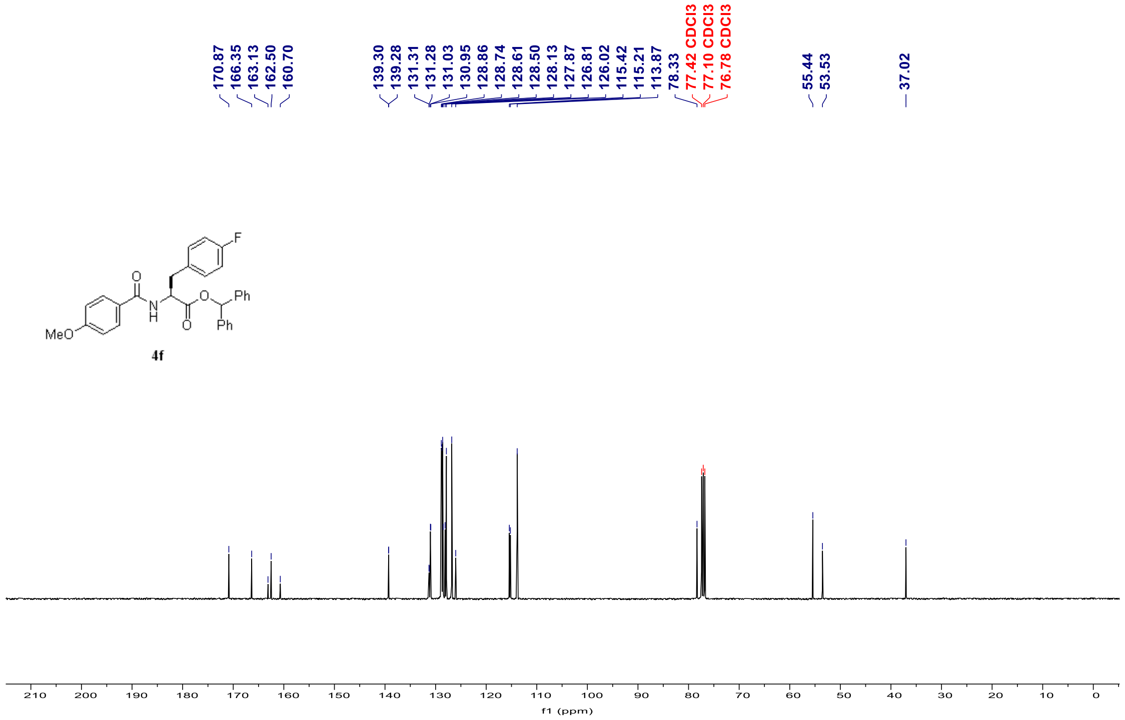

Figure S247. **^{13}C NMR** of the **4f** (101 MHz, $CDCl_3$)

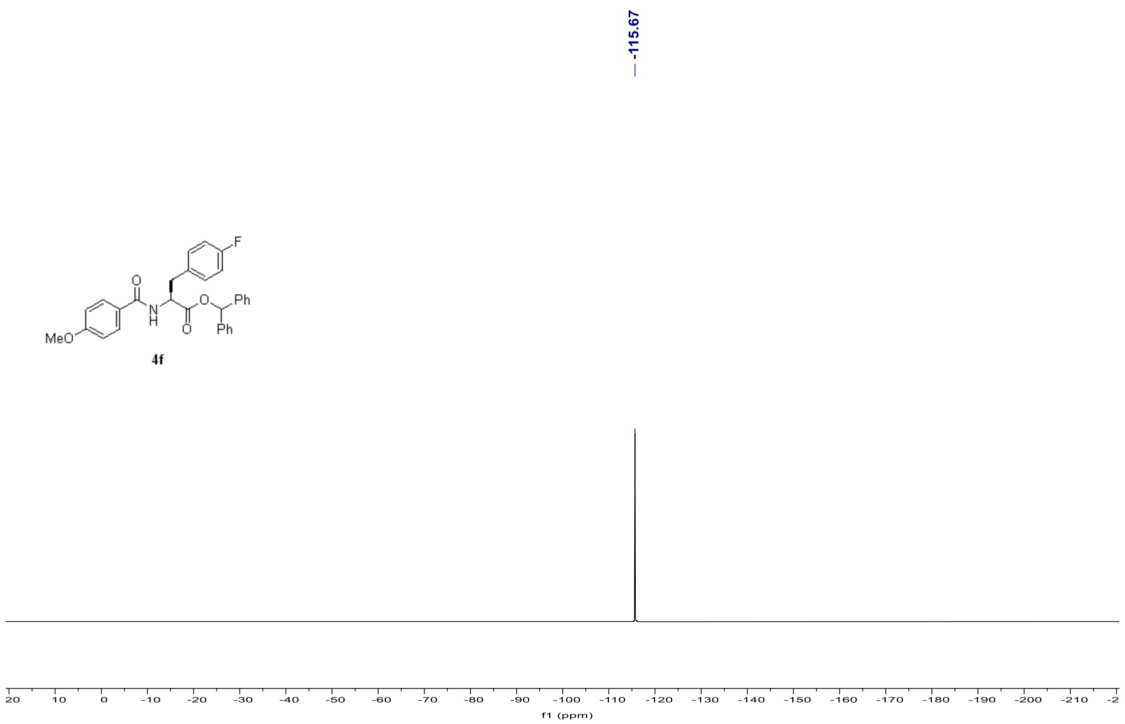

Figure S248. ^{19}F NMR of the **4f** (376 MHz, $CDCl_3$)

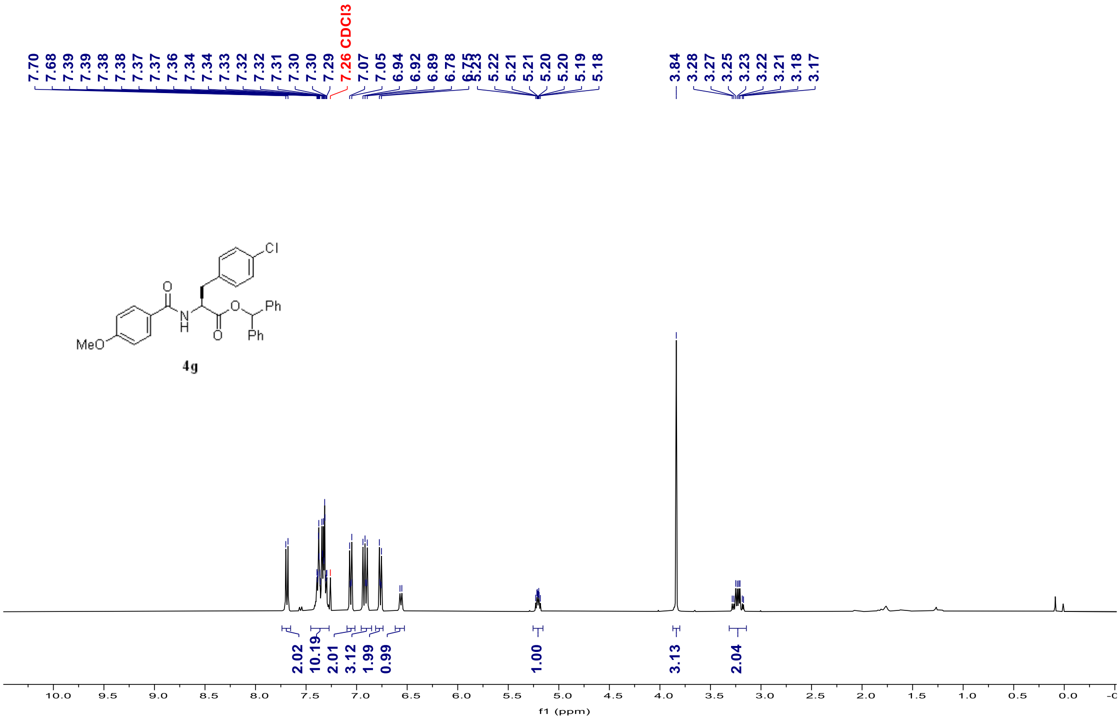

Figure S249. ^{1}H NMR of the **4g** (400 MHz, $CDCl_3$**)**

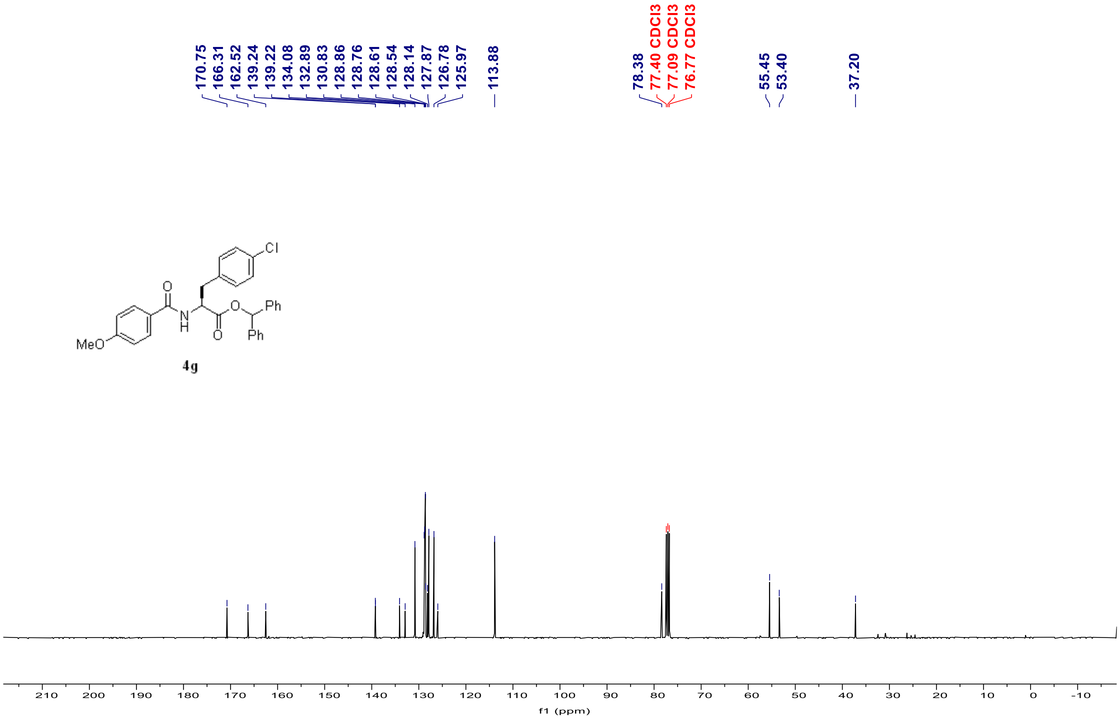

Figure S250. ^{13}C NMR of the **4g** (101 MHz, $CDCl_3$**)**

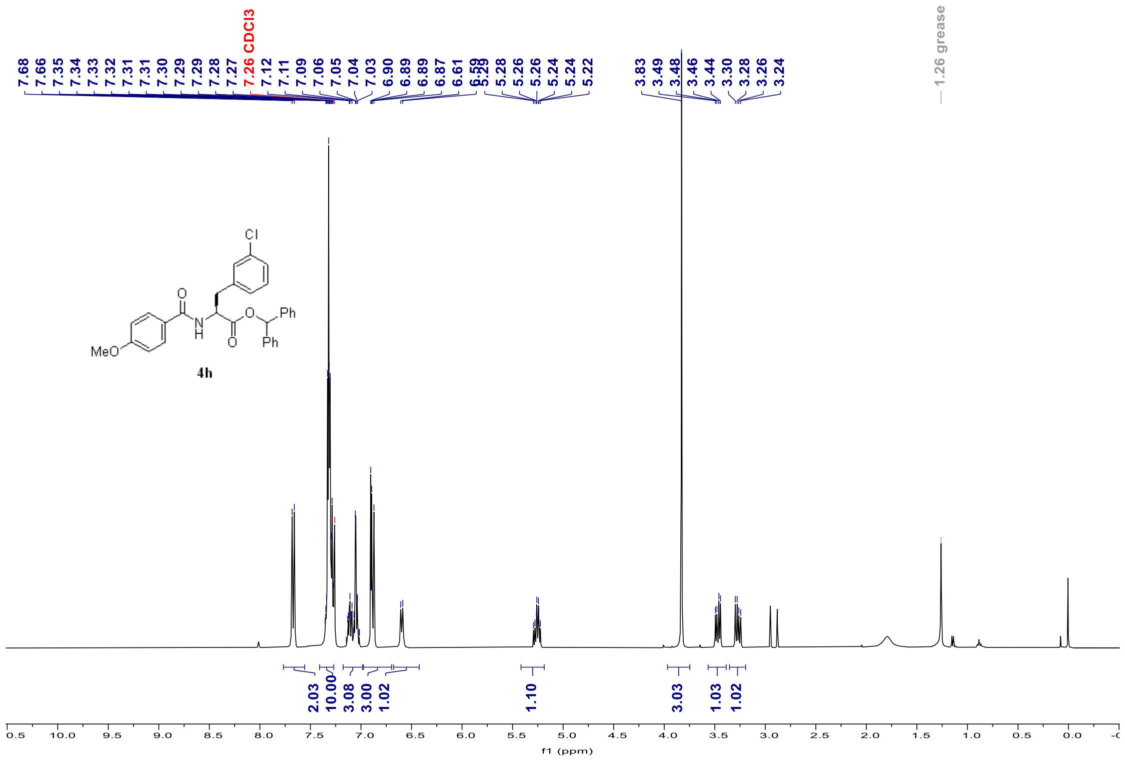

Figure S251. ^{1}H NMR of the **4h** (400 MHz, $CDCl_3$)

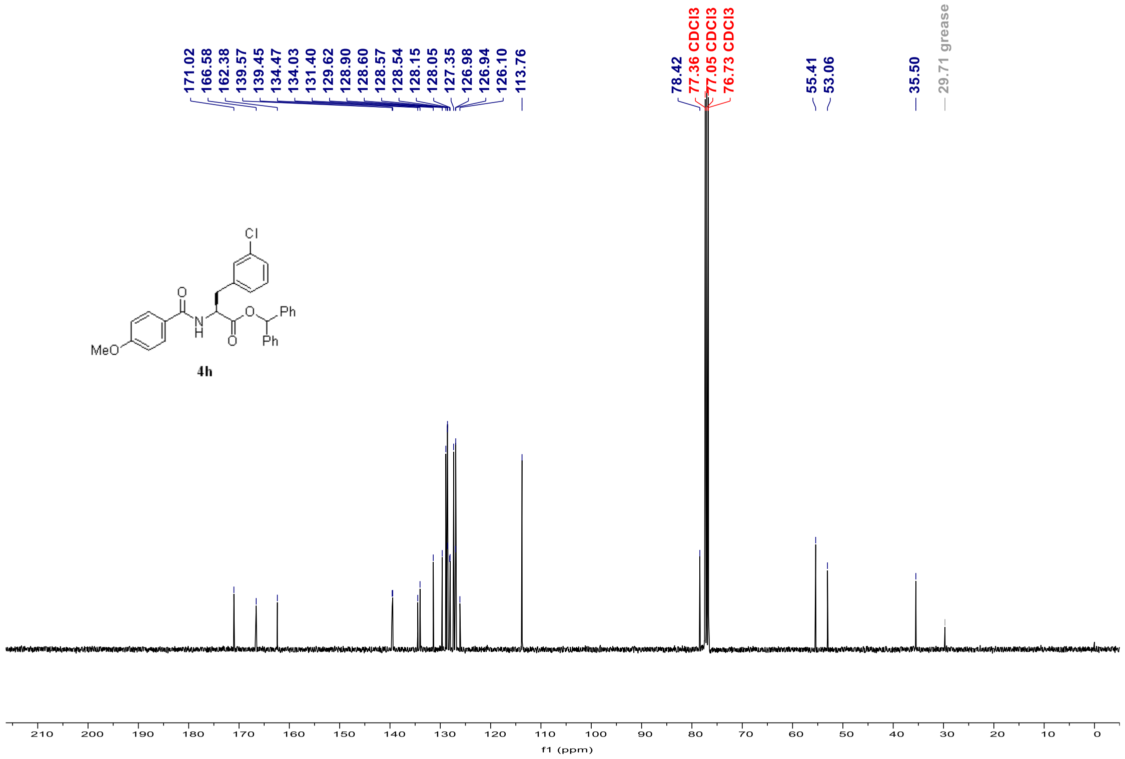

Figure S252. ^{13}C NMR of the **4h** (101 MHz, $CDCl_3$)

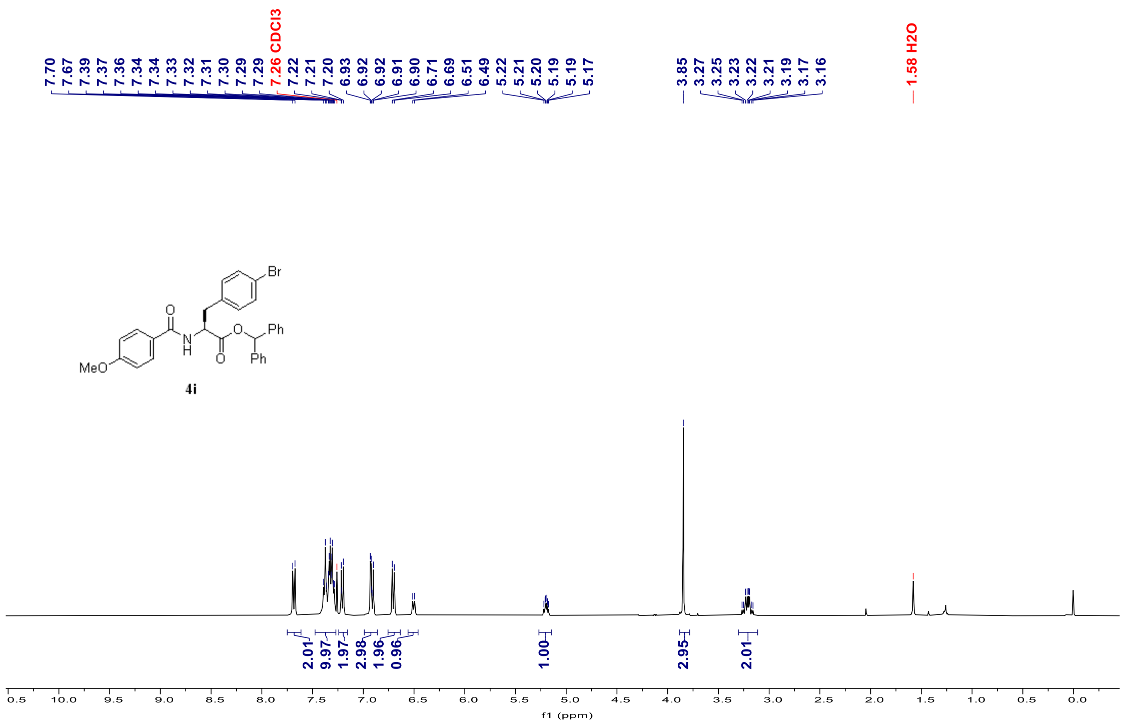

Figure S253. ^{1}**H NMR** of the **4i** (400 MHz, $CDCl_3$)

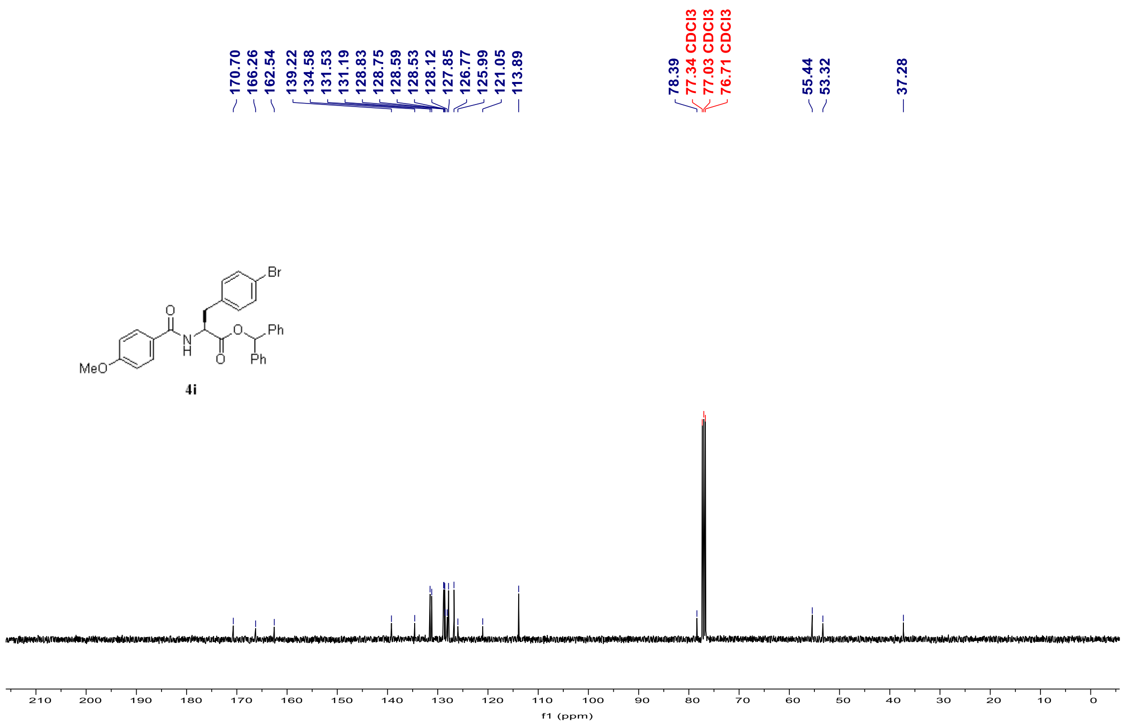

Figure S254. ^{13}**C NMR** of the **4i** (101 MHz, $CDCl_3$)

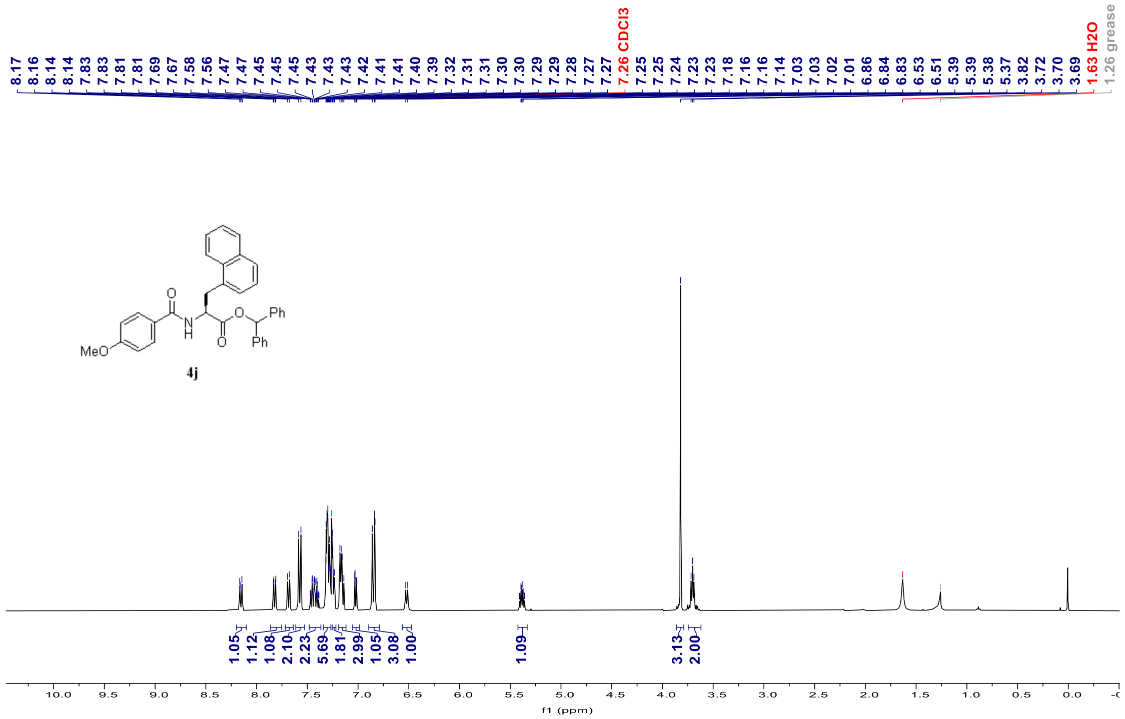

Figure S255. **^{1}H NMR** of the **4j** (400 MHz, $CDCl_3$)

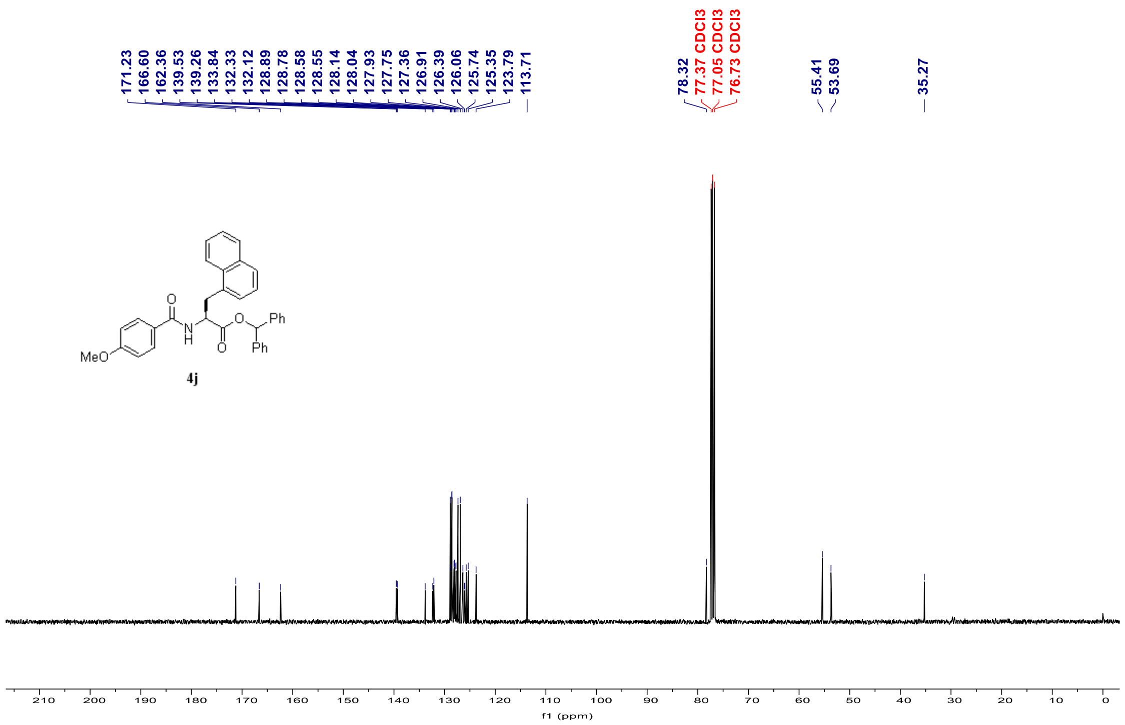

Figure S256. **^{13}C NMR** of the **4j** (101 MHz, $CDCl_3$)

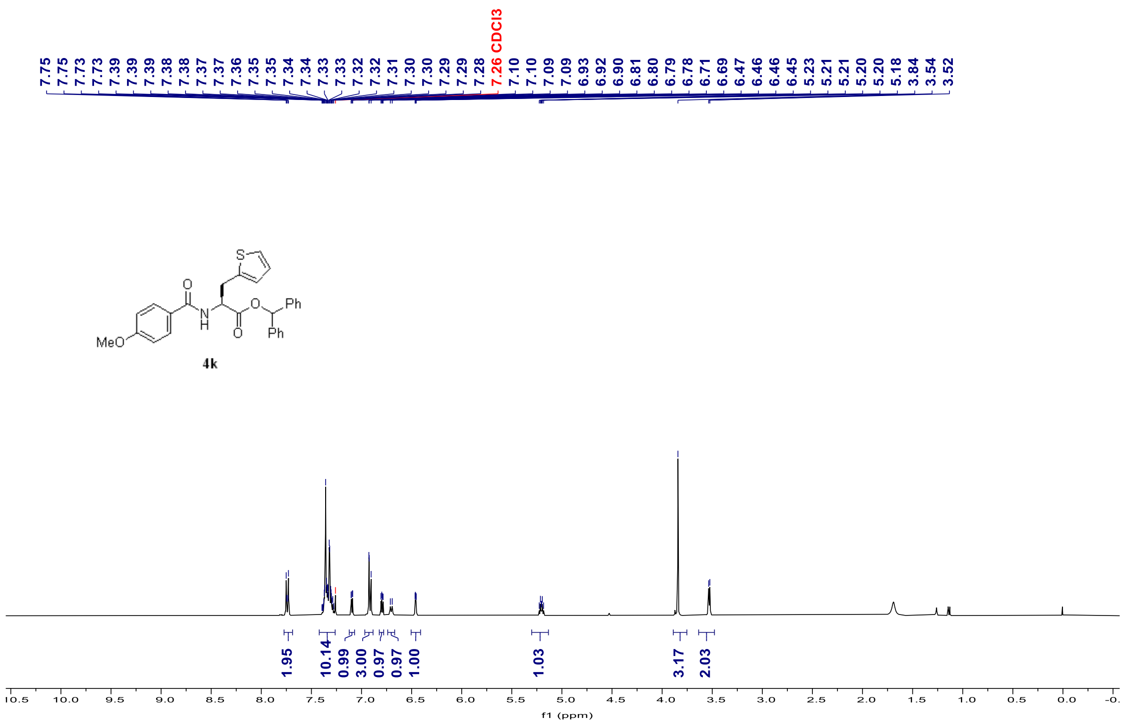

Figure S257. ^{1}**H NMR** of the **4k** (400 MHz, $CDCl_3$)

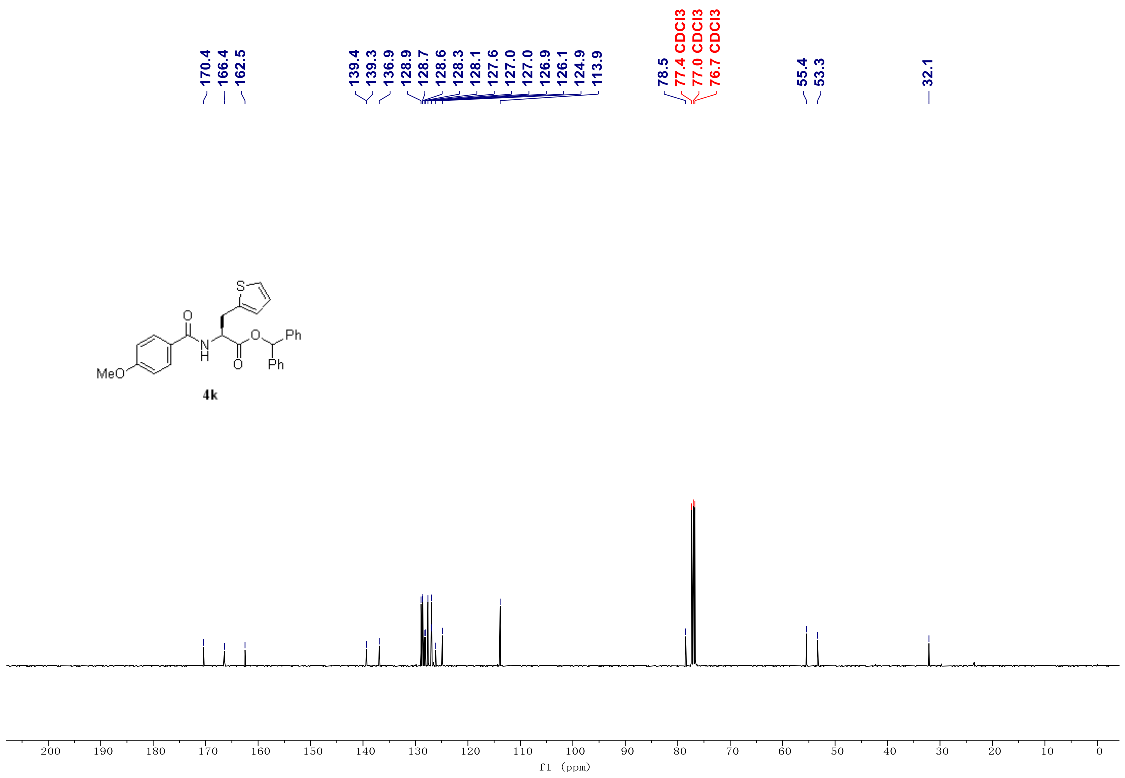

Figure S258. ^{13}**C NMR** of the **4k** (101 MHz, $CDCl_3$)

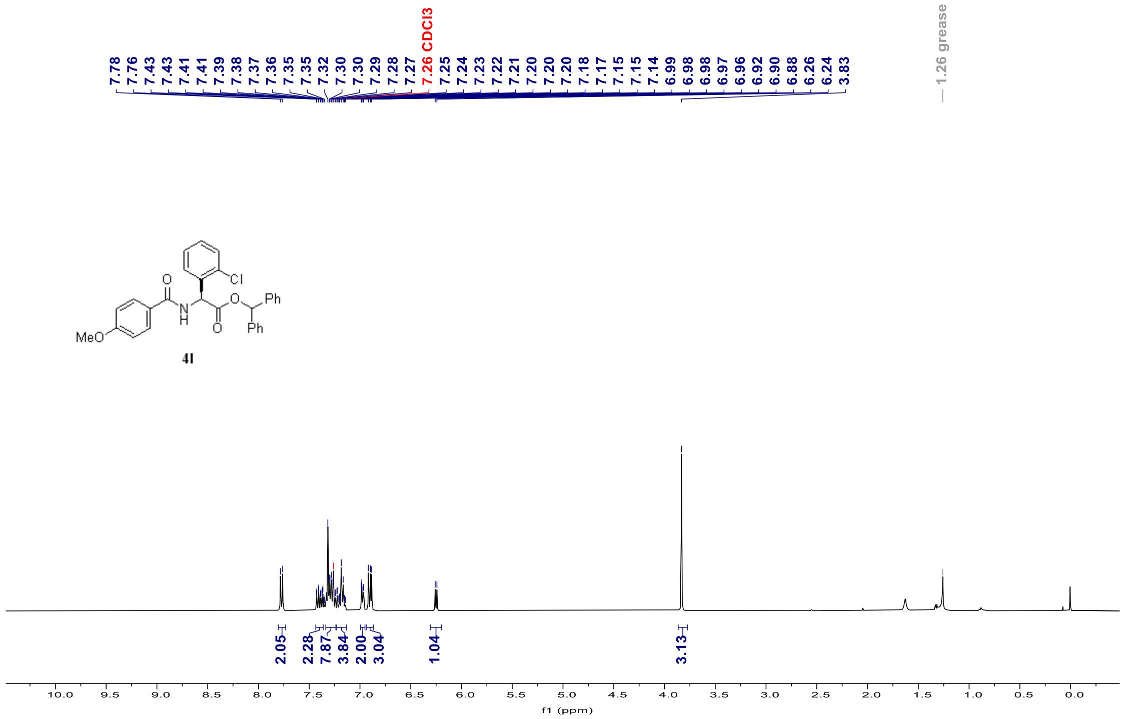

Figure S259. ^{1}**H NMR** of the **4l** (400 MHz, $CDCl_3$)

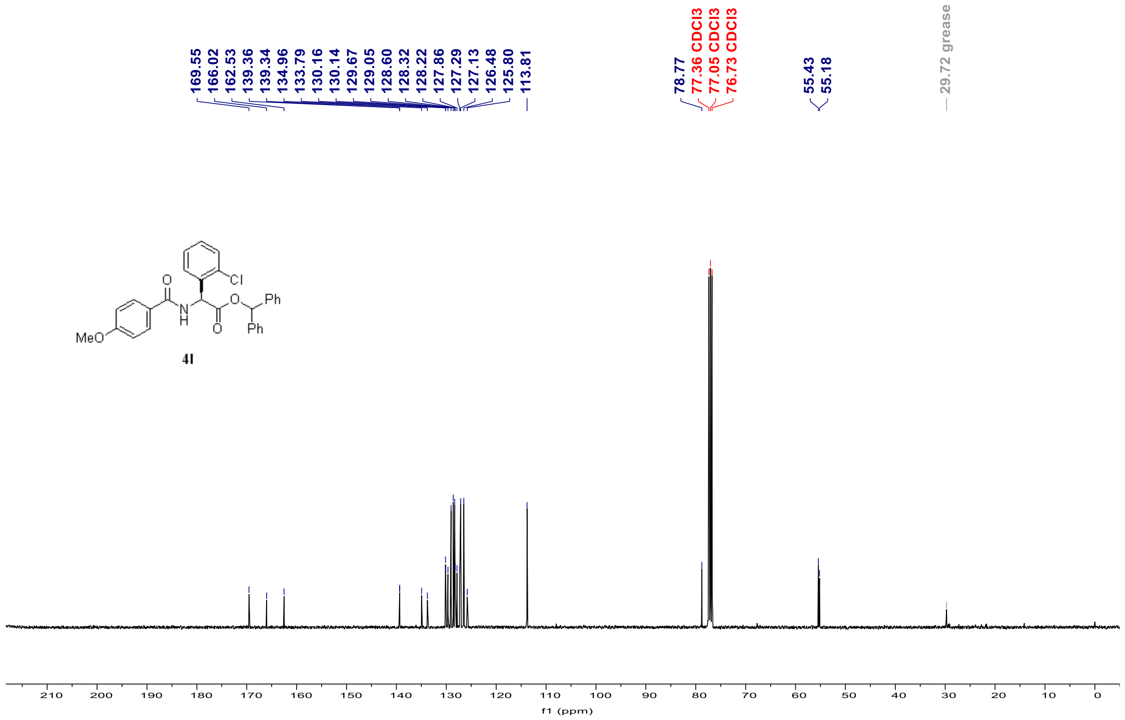

Figure S260. ^{13}**C NMR** of the **4l** (101 MHz, $CDCl_3$)

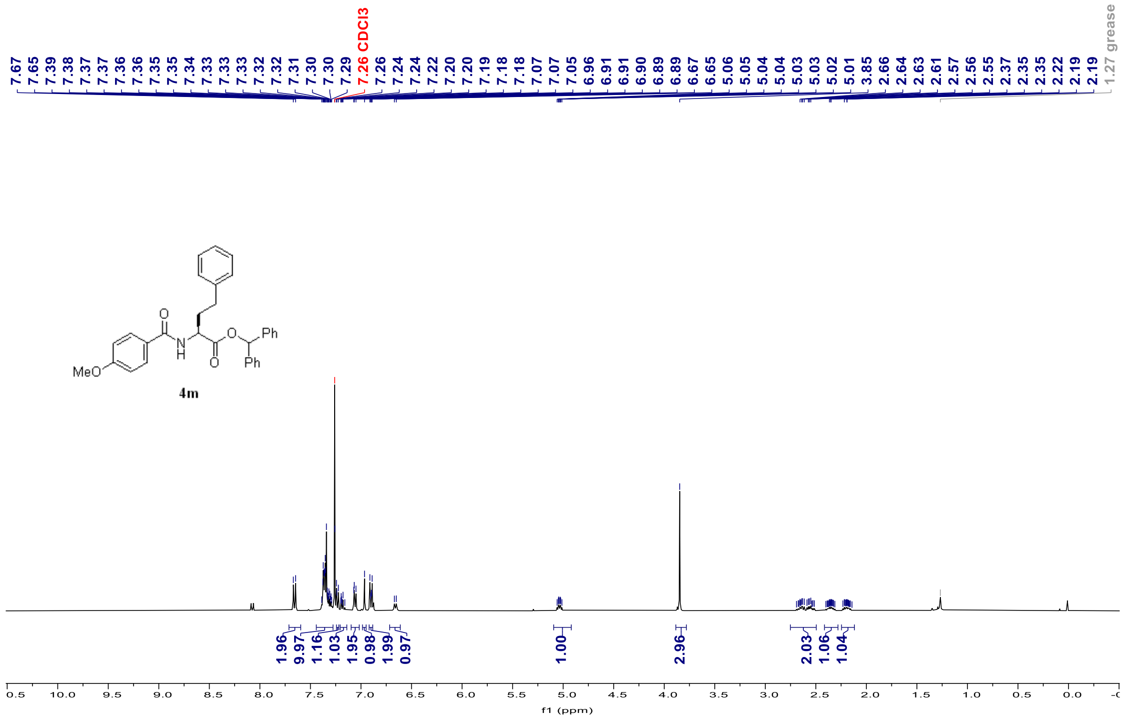

Figure S261. ^{1}**H NMR** of the **4m** (400 MHz, $CDCl_3$)

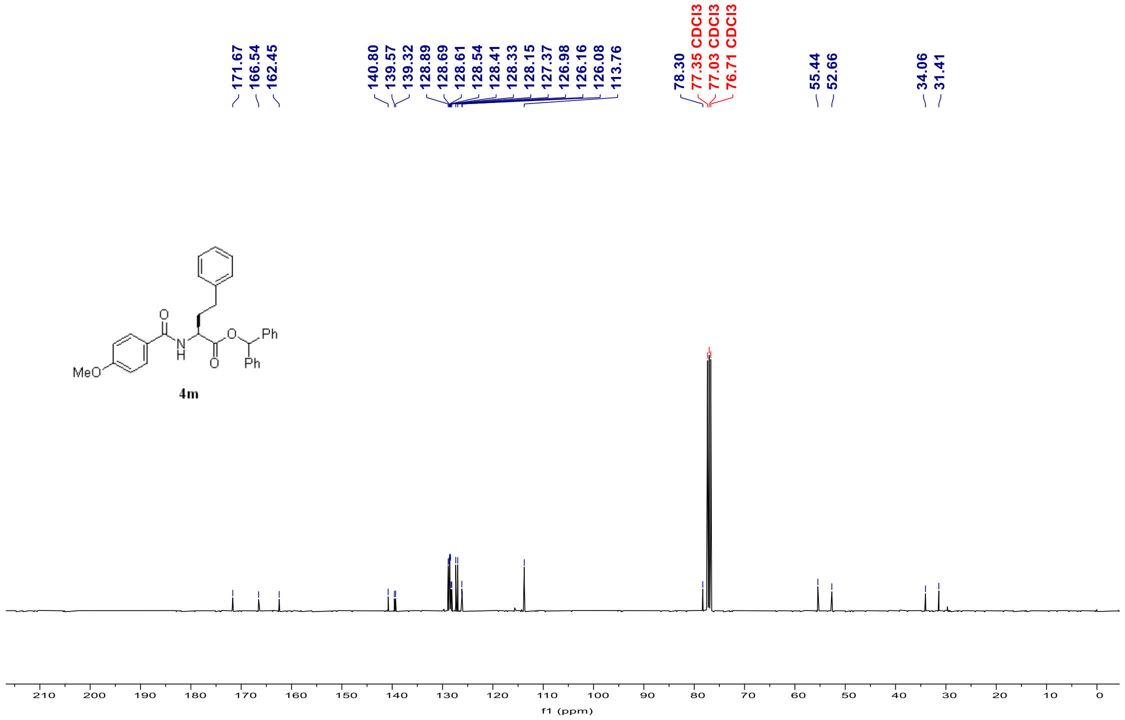

Figure S262. ^{13}**C NMR** of the **4m** (101 MHz, $CDCl_3$)

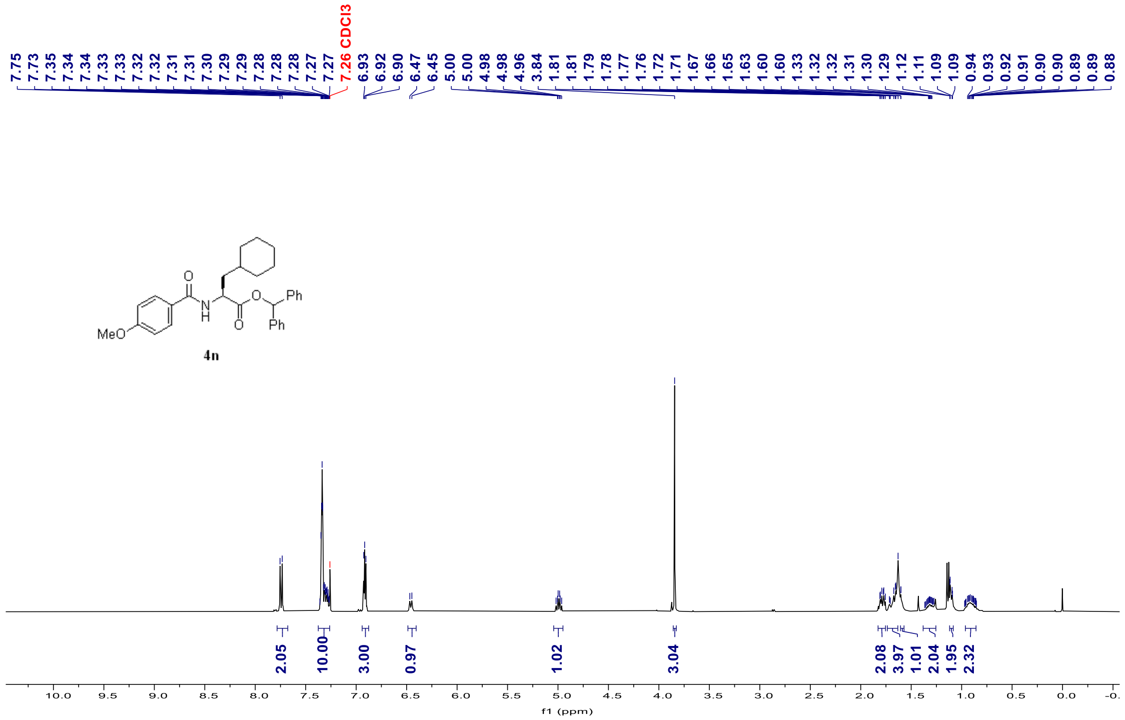

Figure S263. ^{1}H NMR of the **4n** (400 MHz, CDCl$_3$)

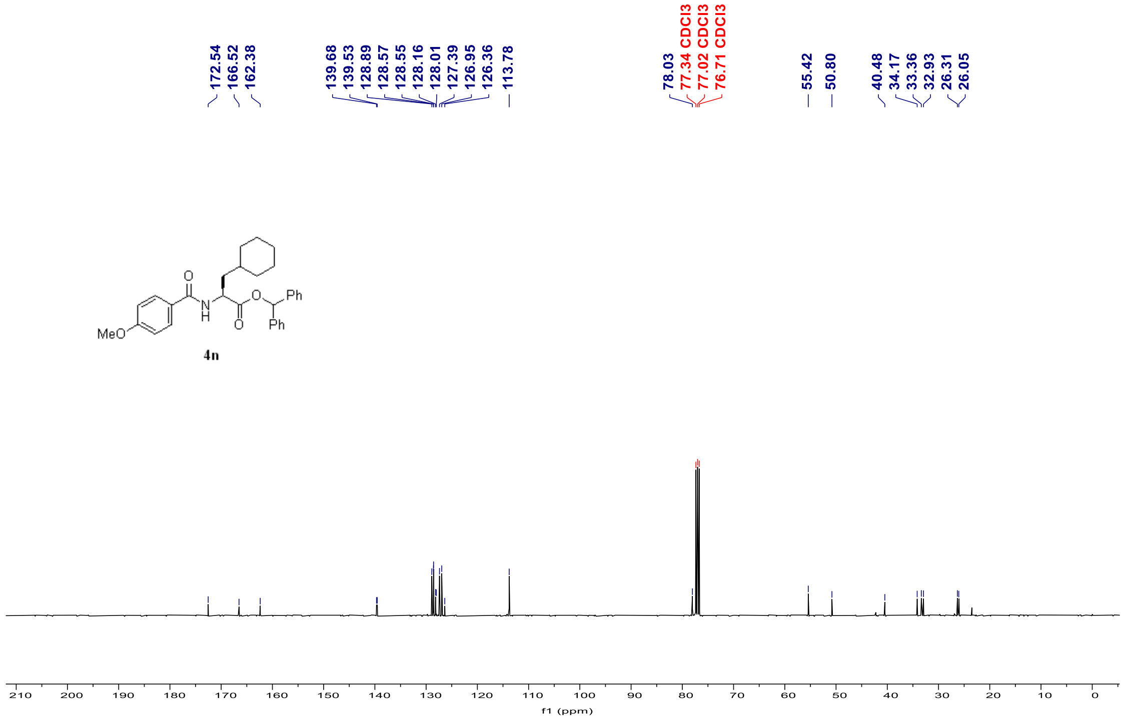

Figure S264. ^{13}C NMR of the **4n** (101 MHz, CDCl$_3$)

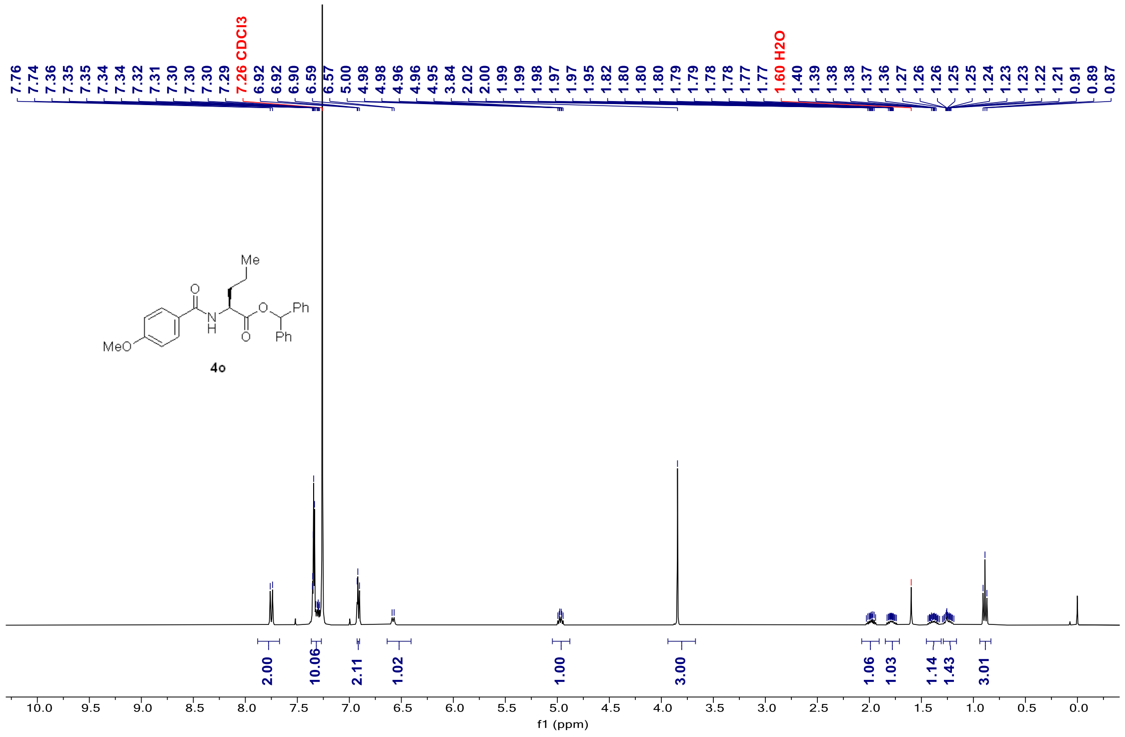

Figure S265. **^{1}H NMR** of the **4o** (400 MHz, $CDCl_3$)

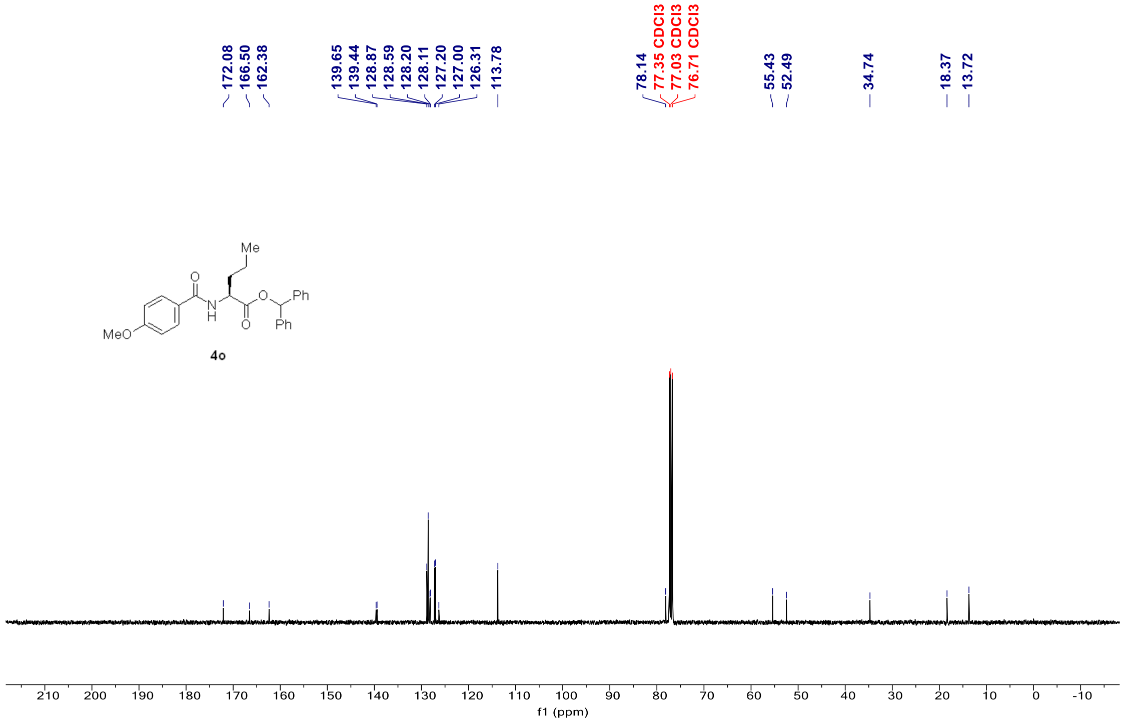

Figure S266. **^{13}C NMR** of the **4o** (101 MHz, $CDCl_3$)

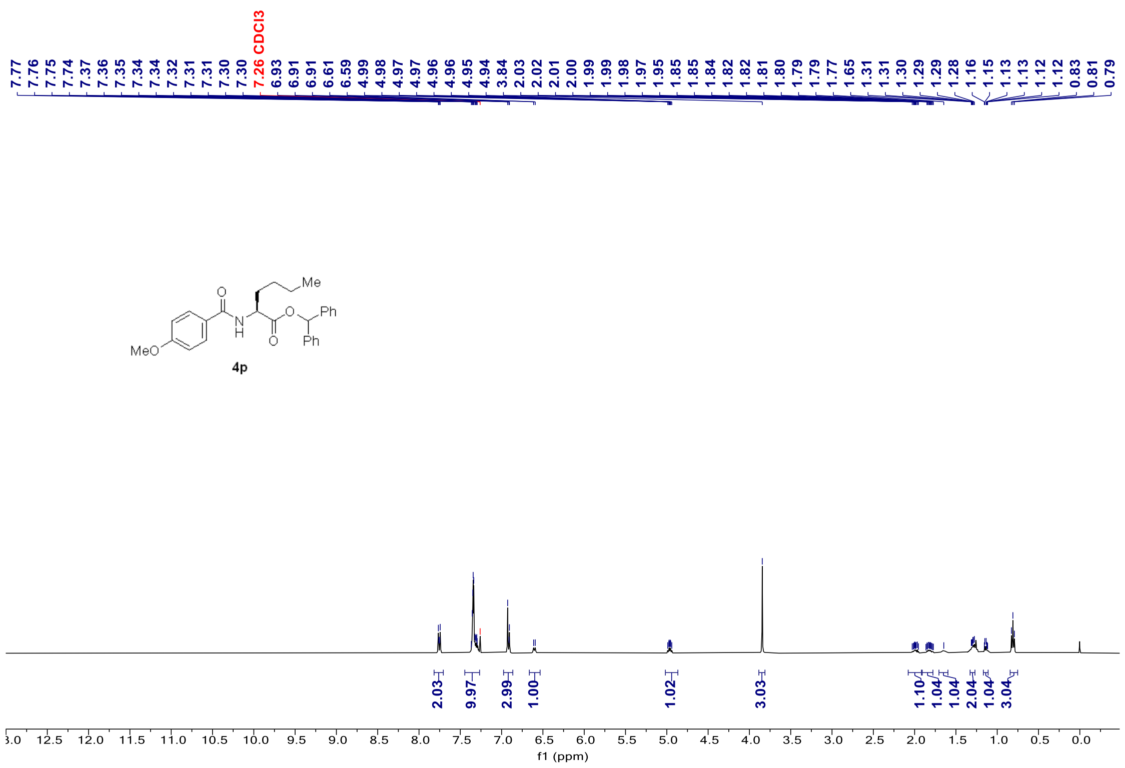

Figure S267. ^{1}**H NMR** of the **4p** (400 MHz, $CDCl_3$)

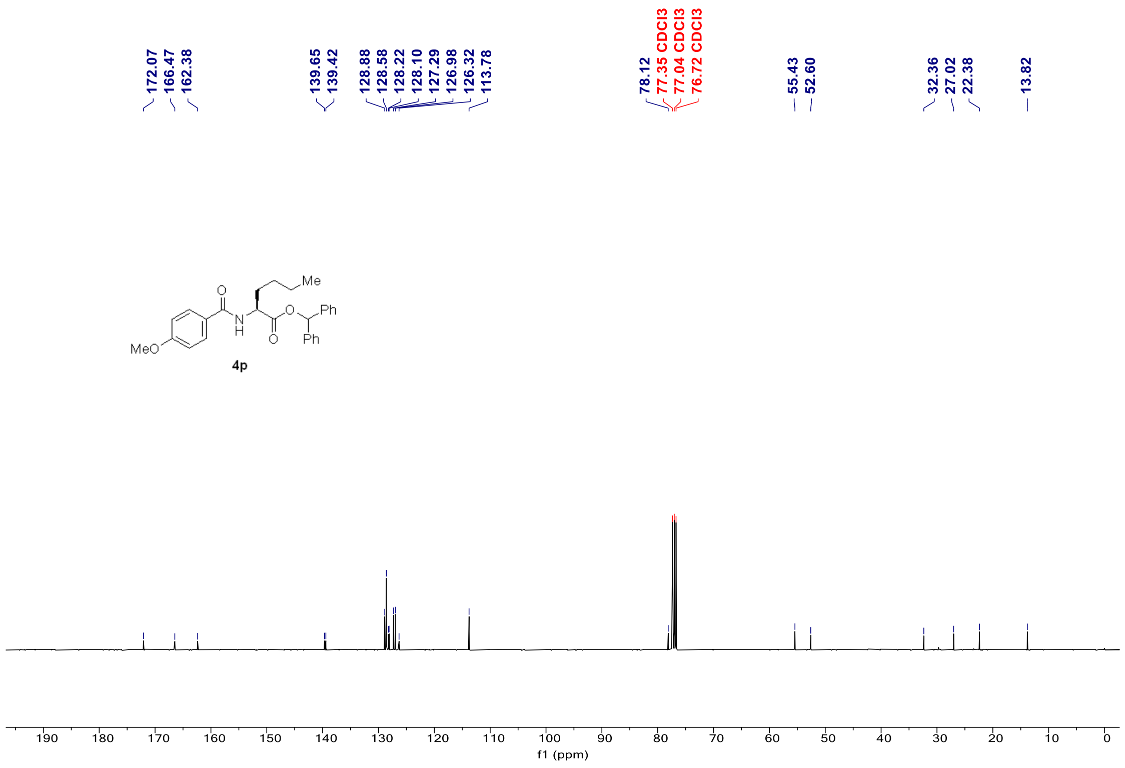

Figure S268. ^{13}**C NMR** of the**4p** (101 MHz, $CDCl_3$)

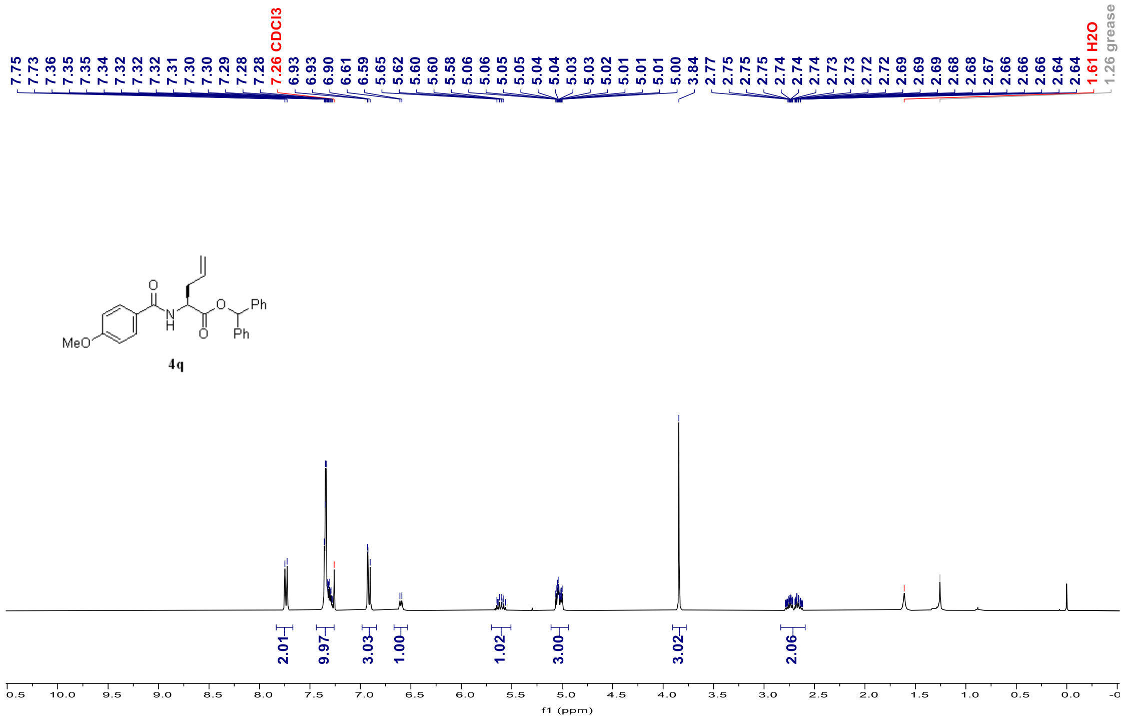

Figure S269. ¹H NMR of the **4q** (400 MHz, $CDCl_3$)

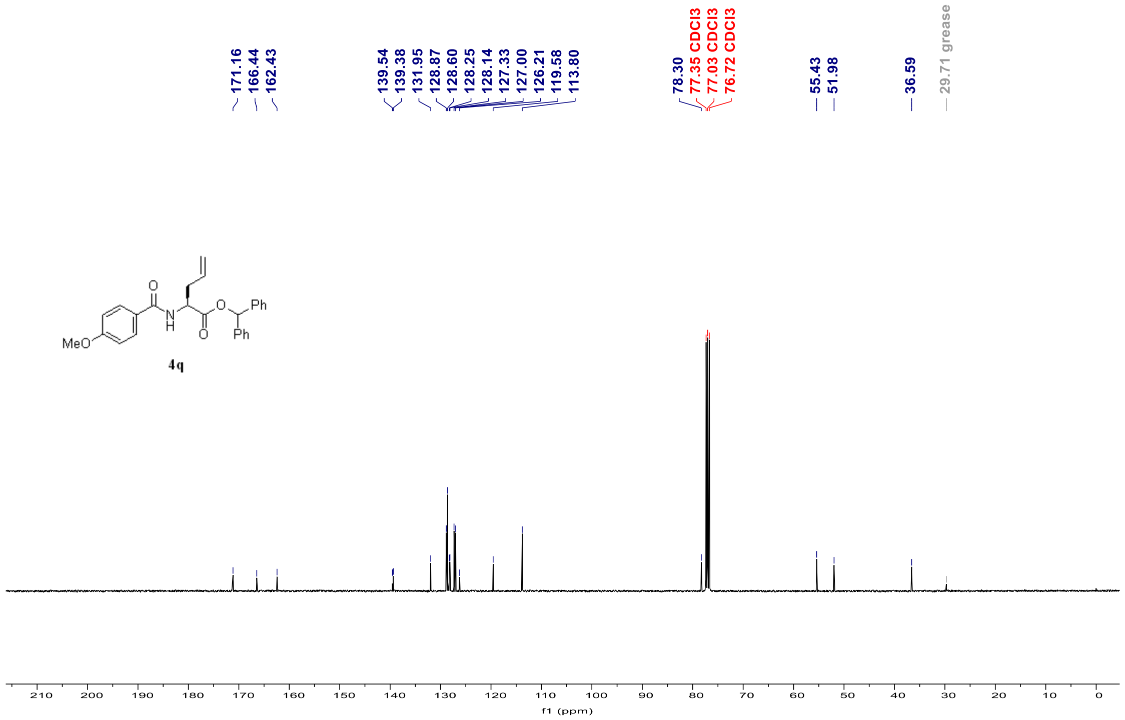

Figure S270. ¹³C NMR of the **4q** (101 MHz, $CDCl_3$)

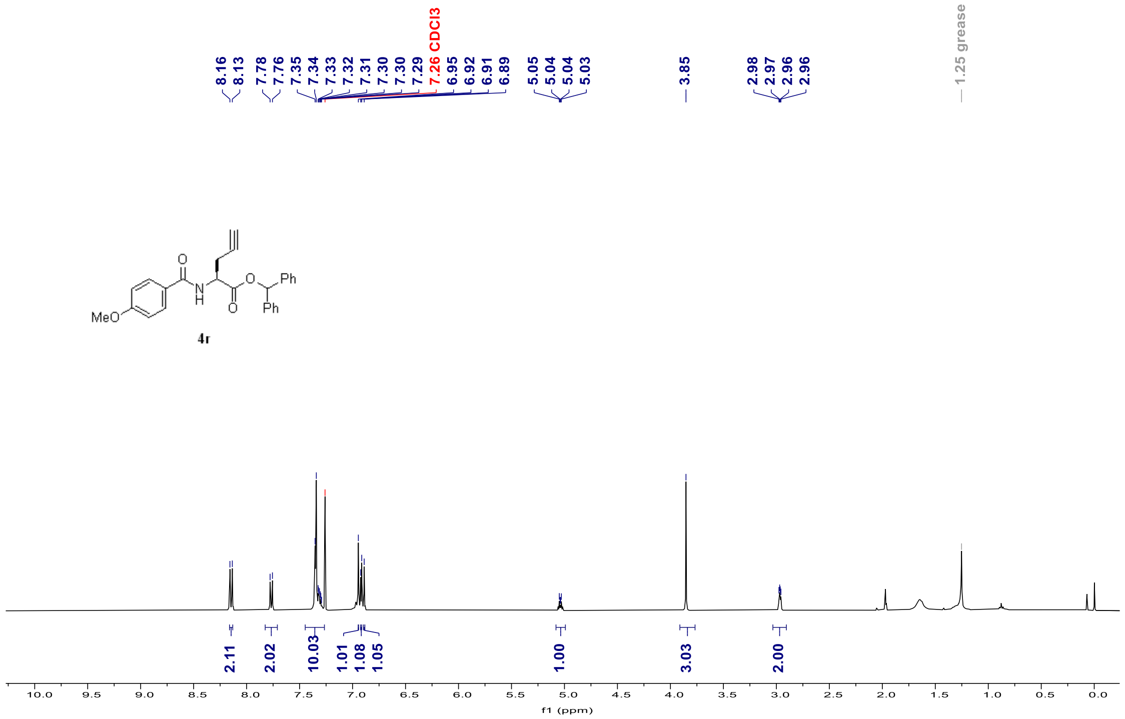

Figure S271. **^{1}H NMR** of the **4r** (400 MHz, CDCl$_3$)

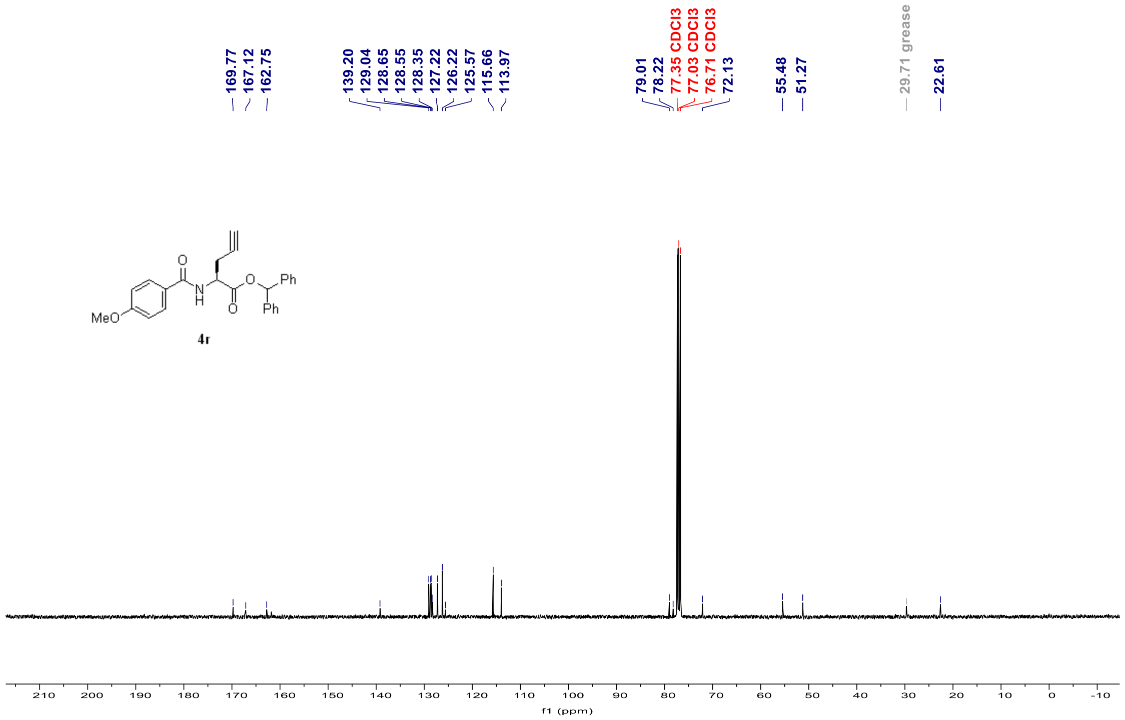

Figure S272. **^{13}C NMR** of the **4r** (101 MHz, CDCl$_3$)

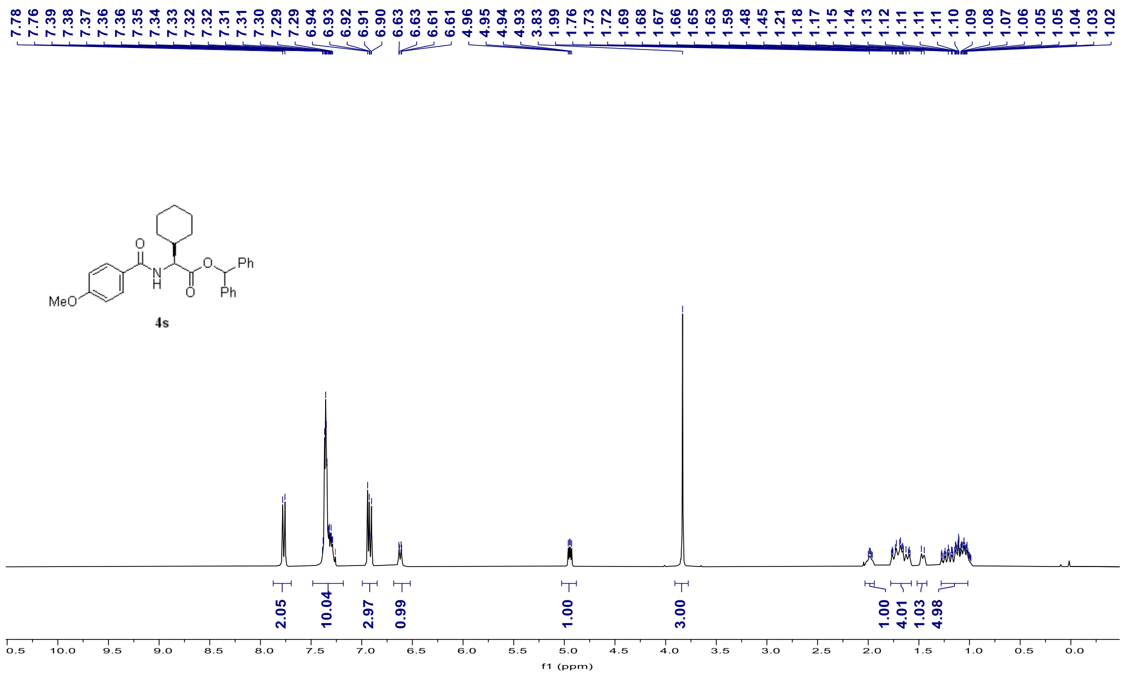

Figure S273. ^{1}**H NMR** of the **4s** (400 MHz, $CDCl_3$)

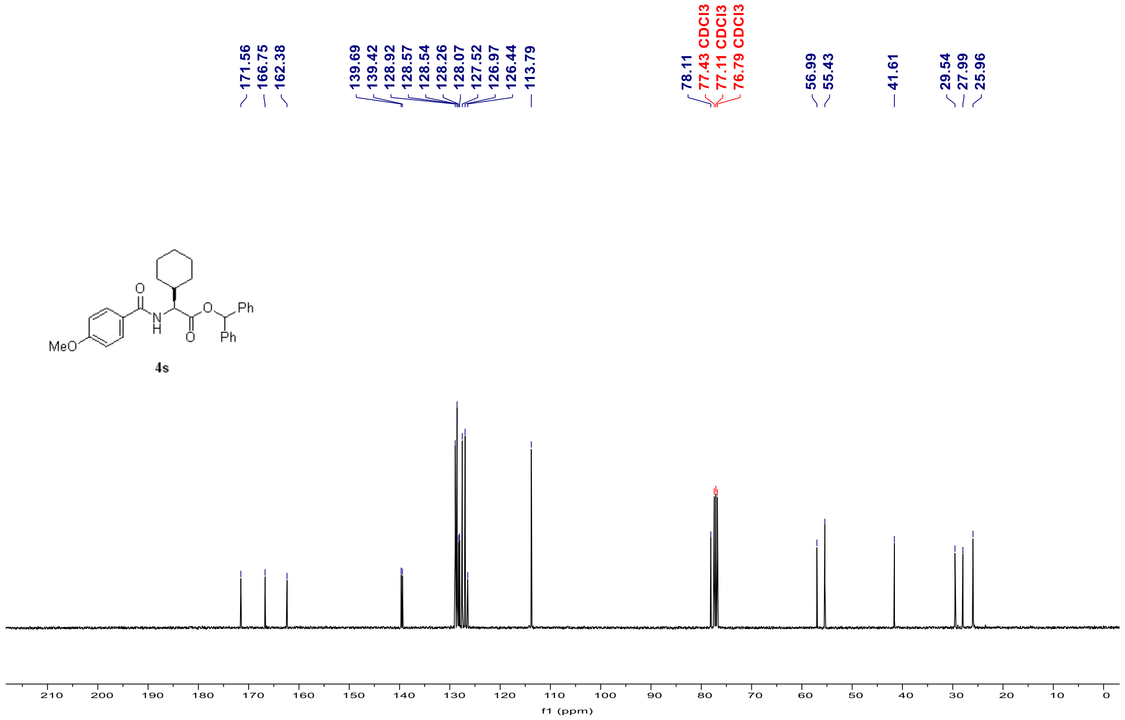

Figure S274. ^{13}**C NMR** of the **4s** (101 MHz, $CDCl_3$)

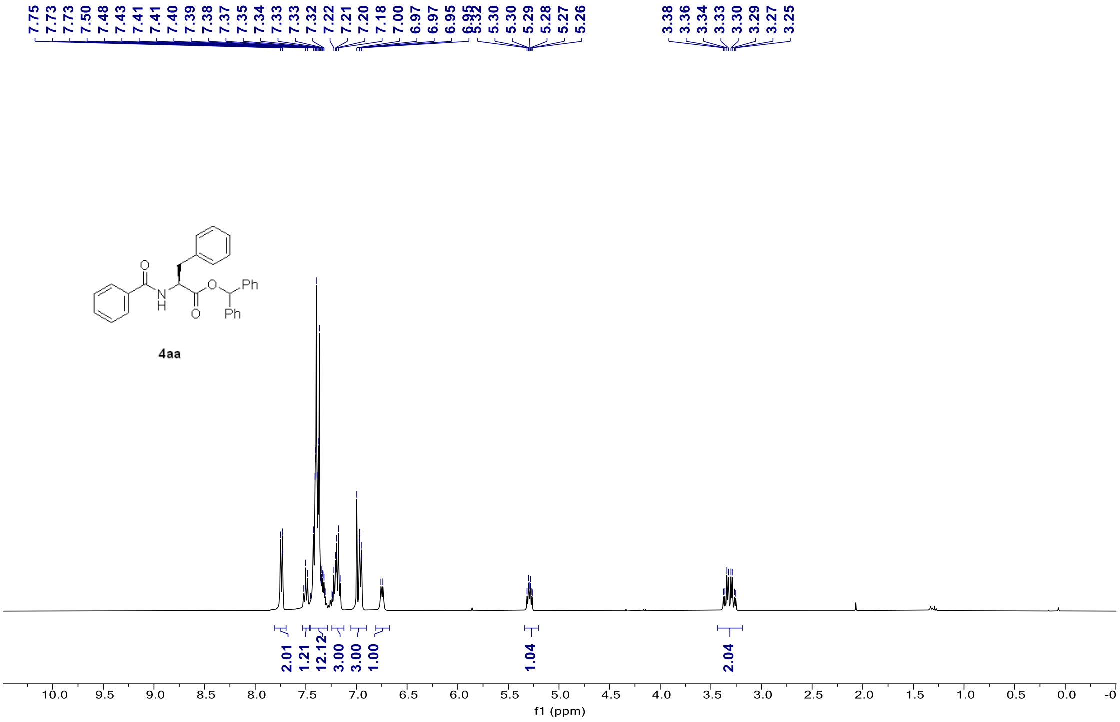

Figure S275. ^{1}**H NMR** of the **4aa** (400 MHz, $CDCl_3$**)**

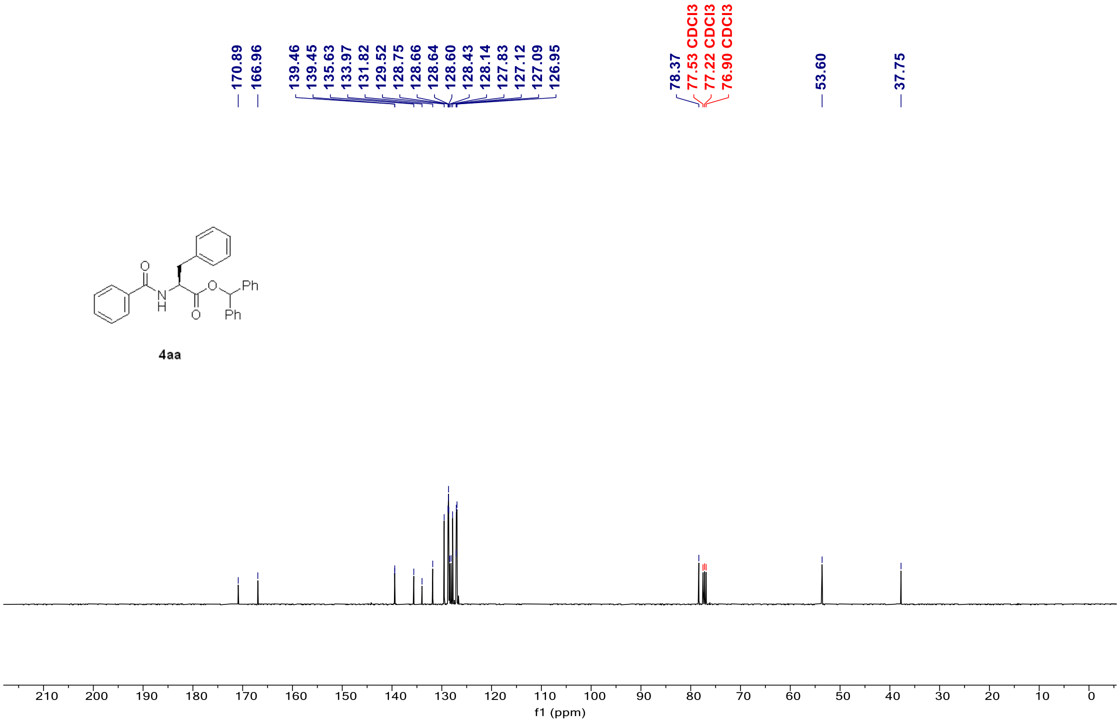

Figure S276. ^{13}**C NMR** of the **4aa** (101 MHz, $CDCl_3$**)**

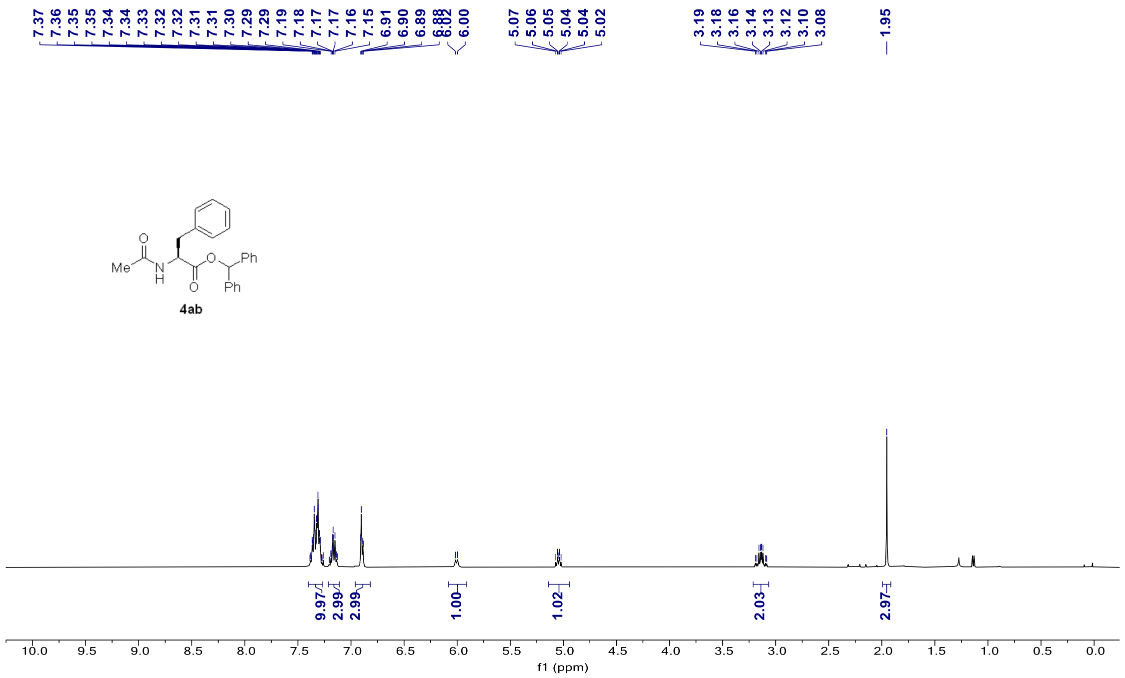

Figure S277. ^{1}**H NMR** of the **4ab** (400 MHz, $CDCl_3$)

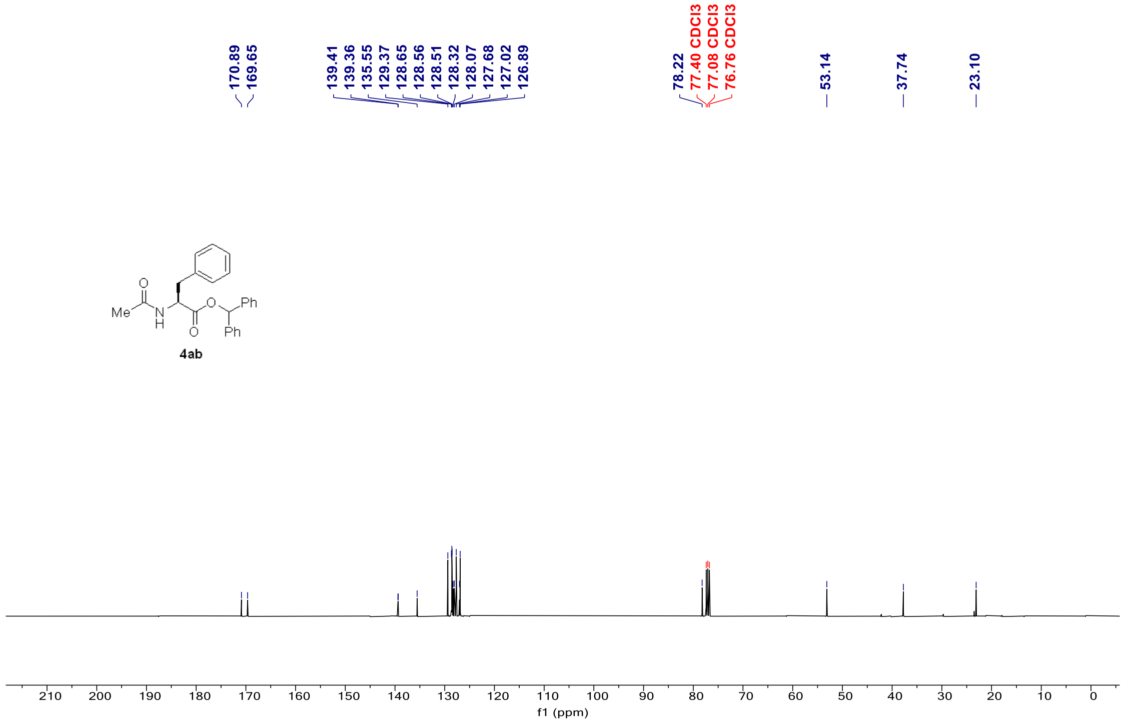

Figure S278. ^{13}**C NMR** of the **4ab** (101 MHz, $CDCl_3$)

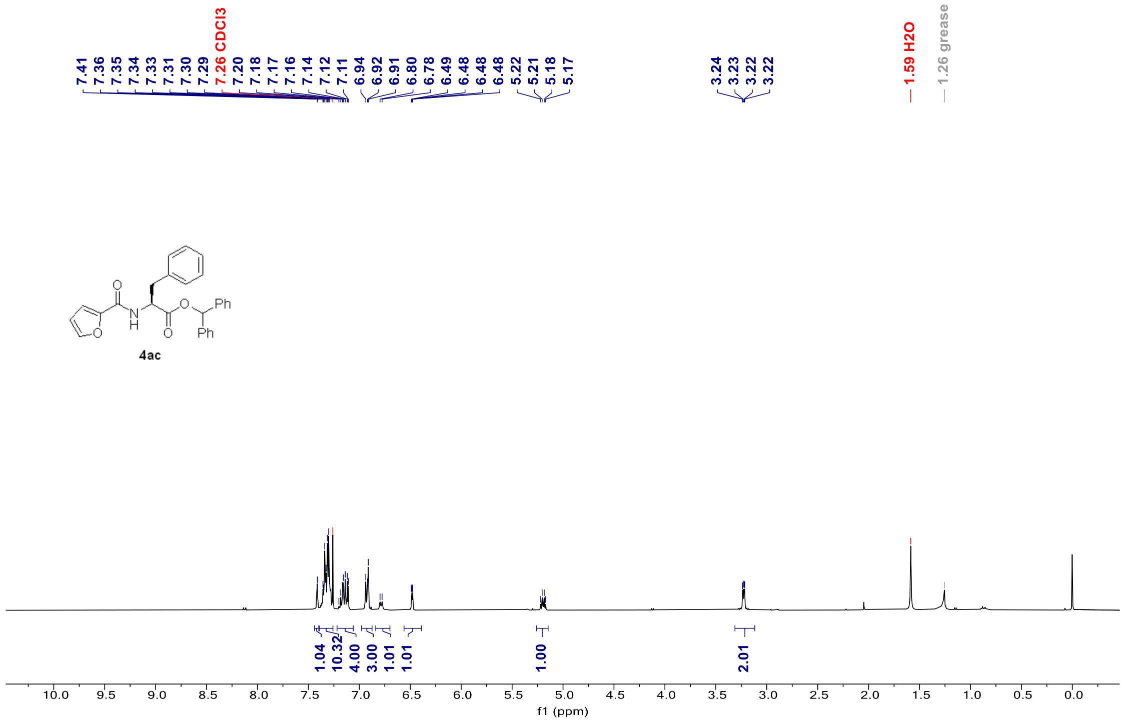

Figure S279. ^{1}**H NMR** of the **4ac** (400 MHz, CDCl$_3$)

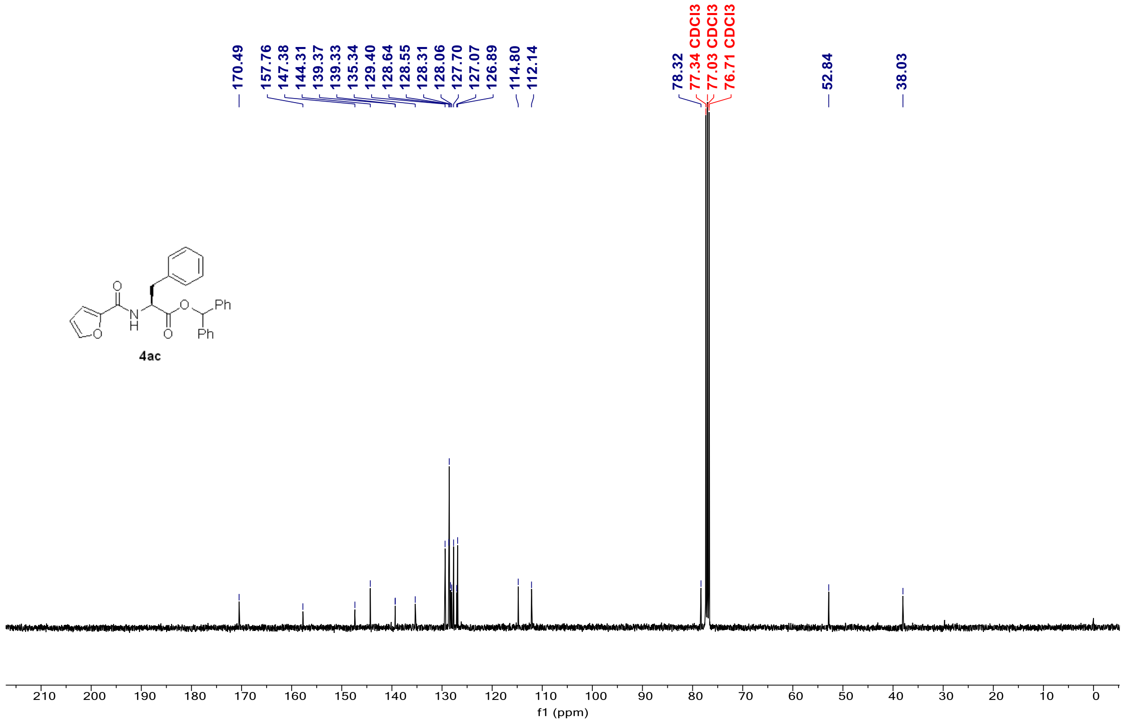

Figure S280. ^{13}**C NMR** of the **4ac** (101 MHz, CDCl$_3$)

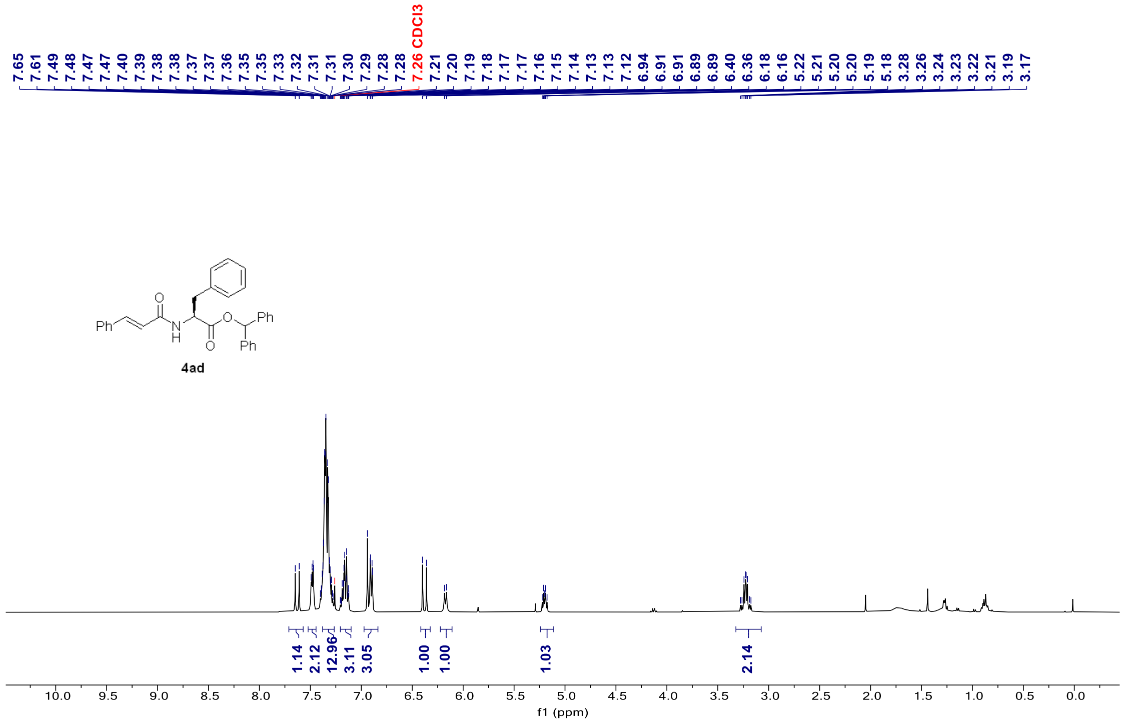

Figure S281. **^{1}H NMR** of the **4ad** (400 MHz, $CDCl_3$)

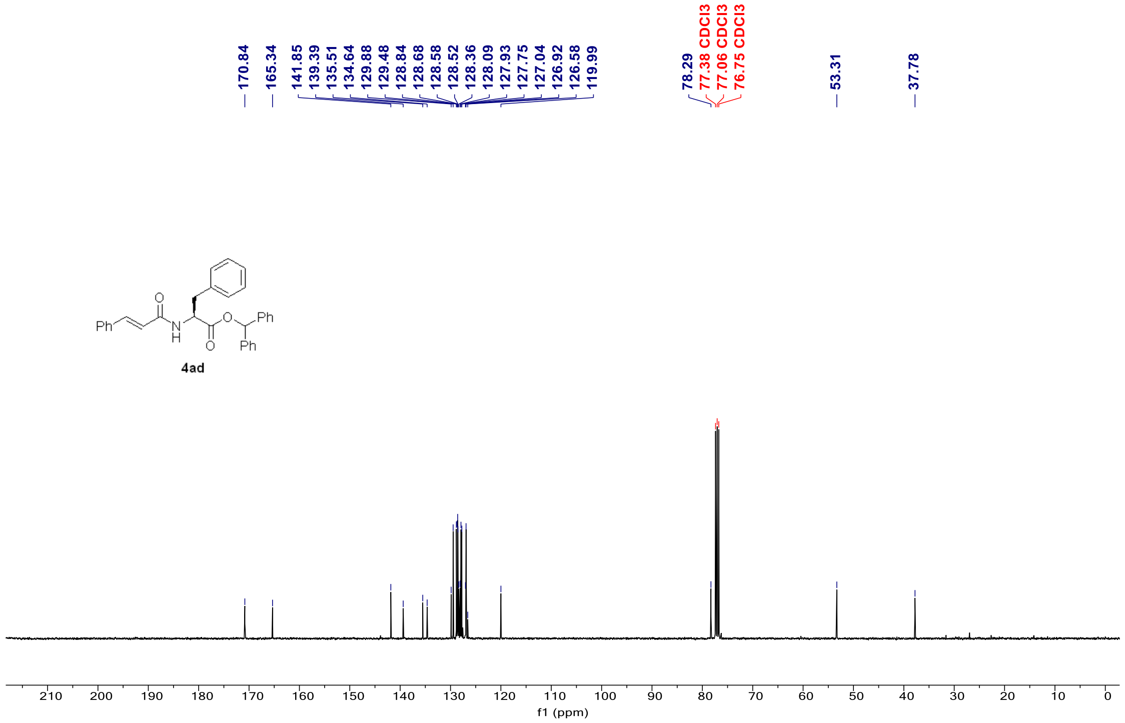

Figure S282. **^{13}C NMR** of the **4ad** (101 MHz, $CDCl_3$)

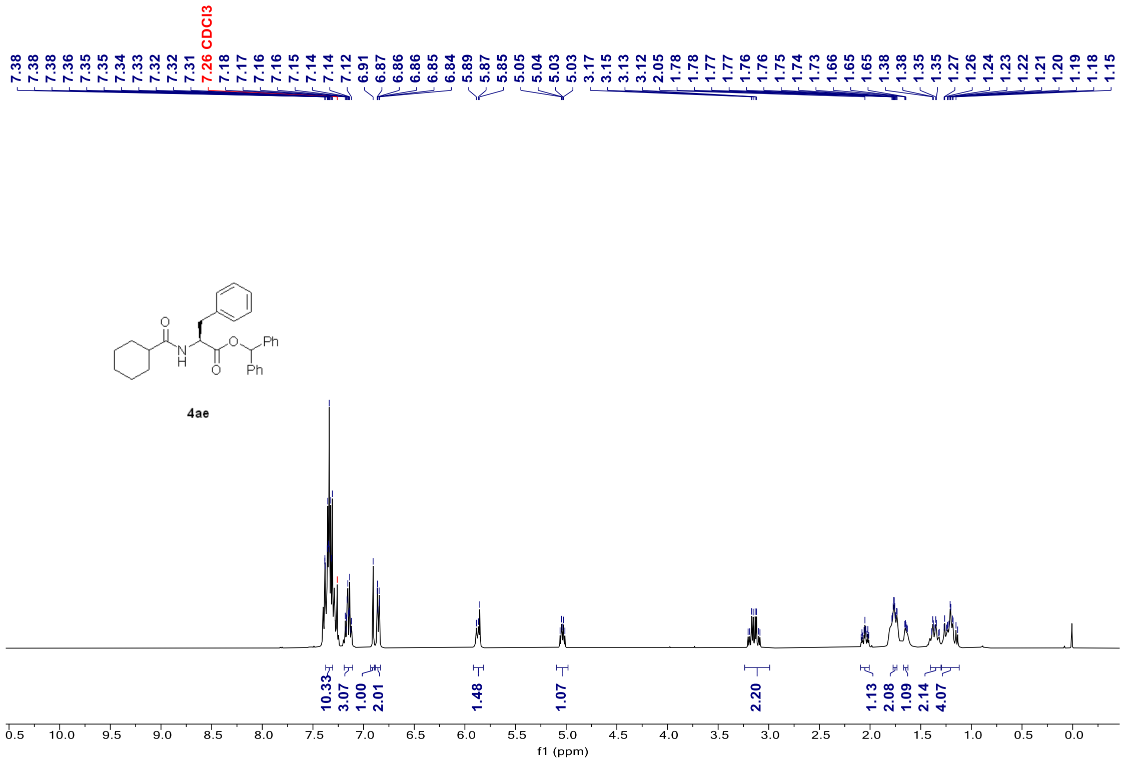

Figure S283. ^{1}**H NMR** of the **4ae** (400 MHz, $CDCl_3$)

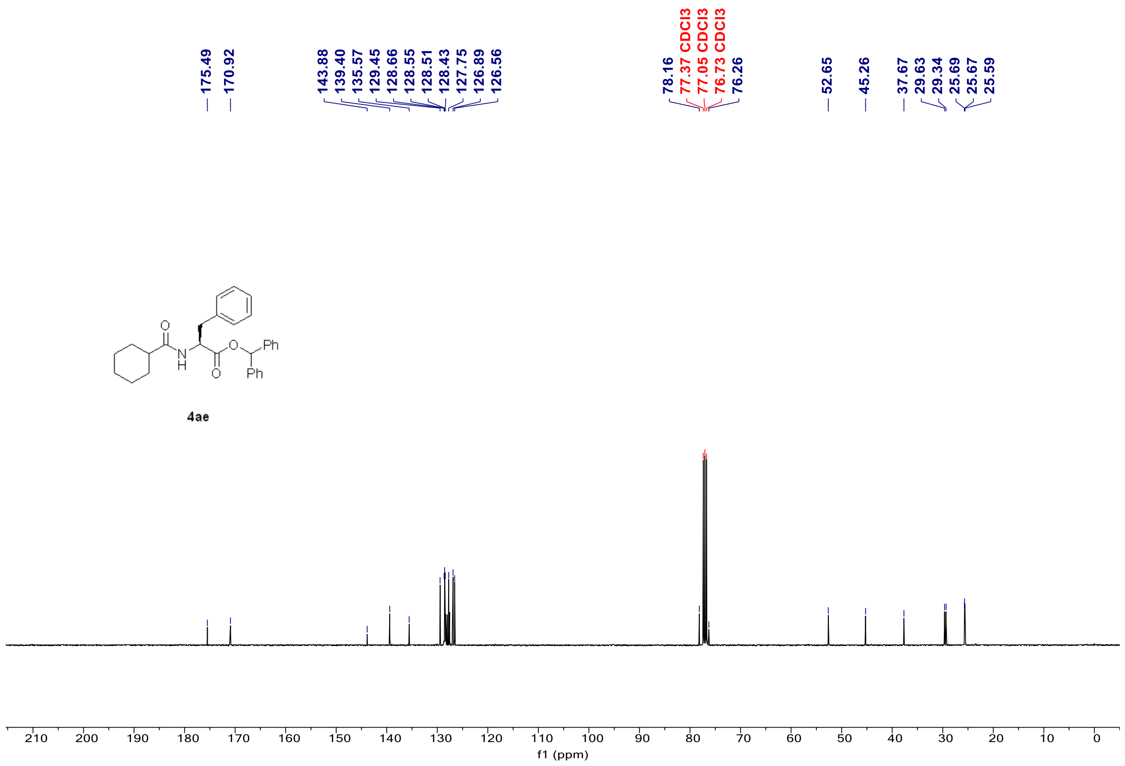

Figure S284. ^{13}**C NMR** of the **4ae** (101 MHz, $CDCl_3$)

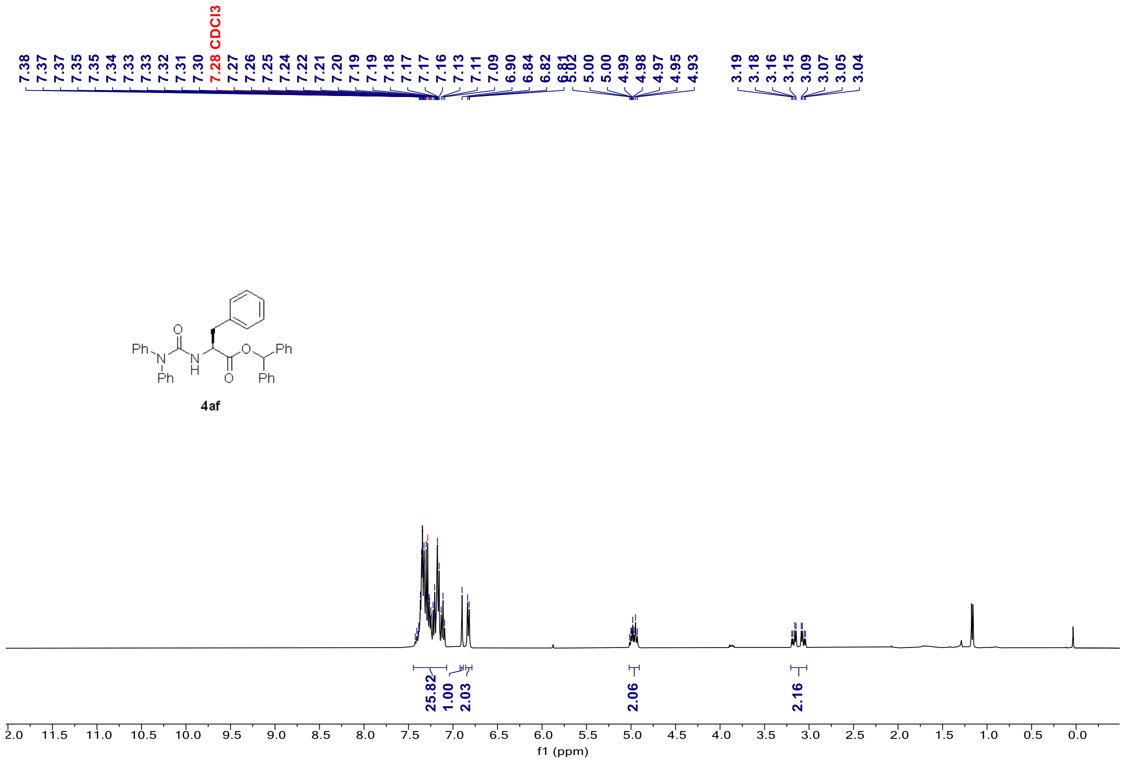

Figure S285. ^{1}**H NMR** of the **4af** (400 MHz, $CDCl_3$)

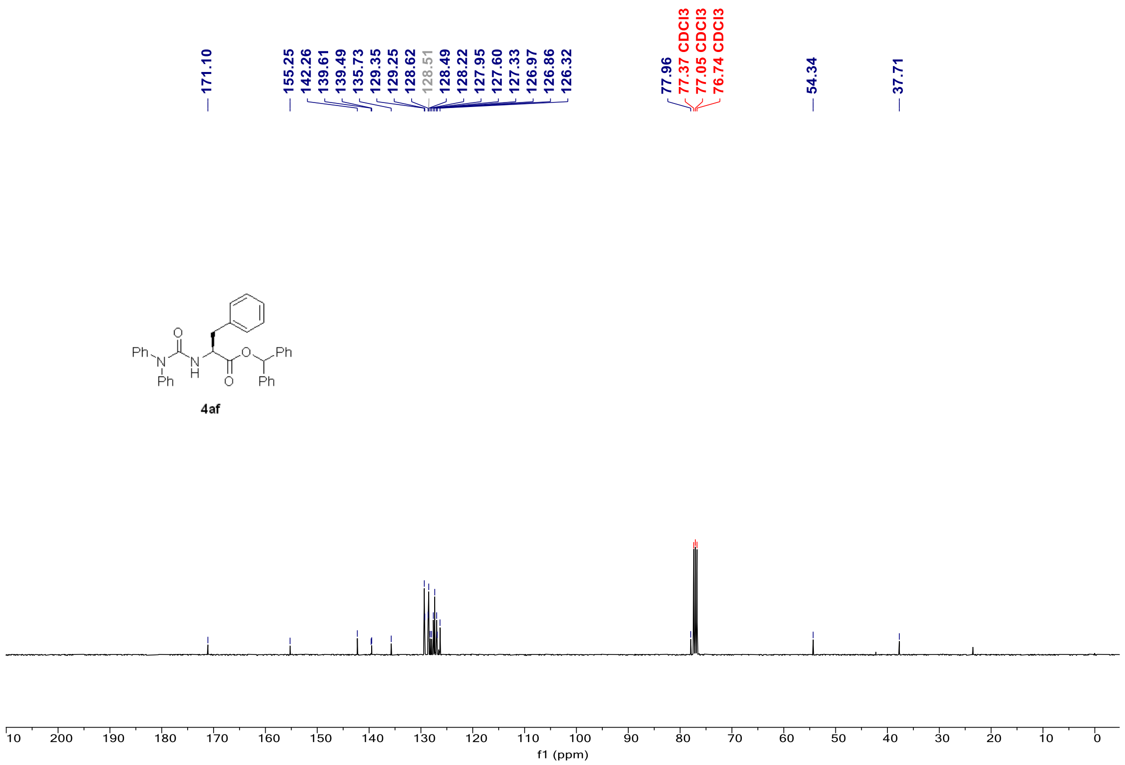

Figure S286. ^{13}**C NMR** of the **4af** (101 MHz, $CDCl_3$)

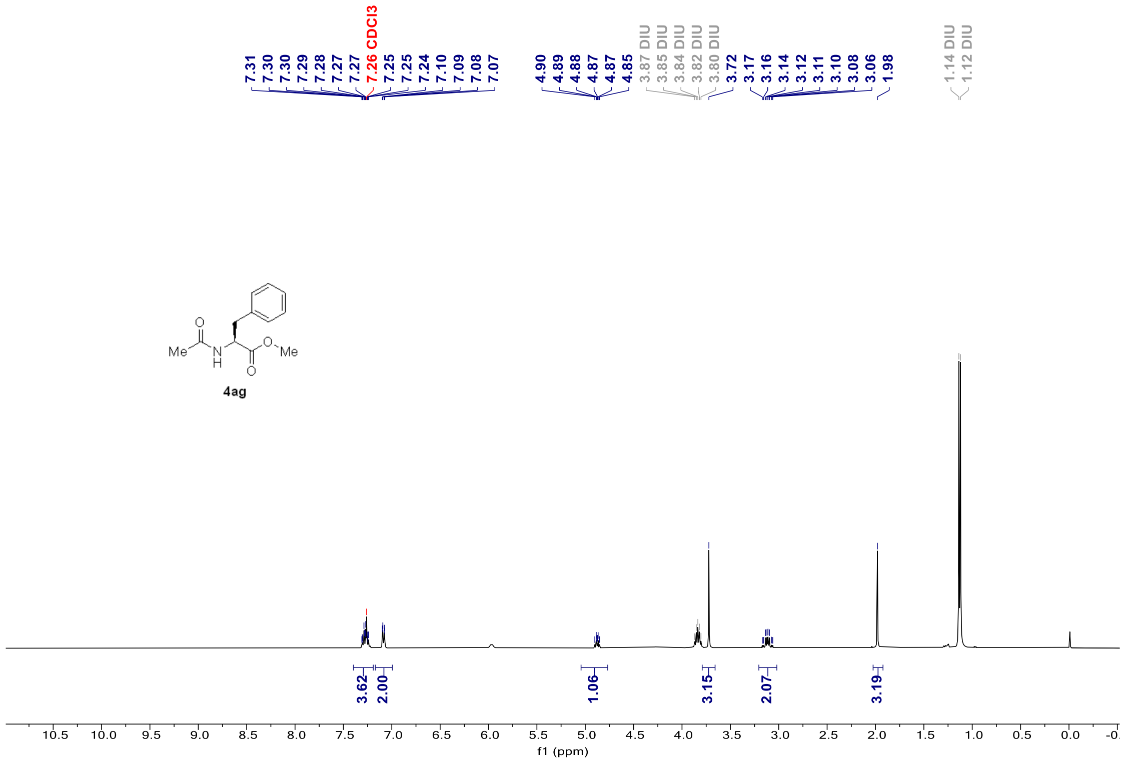

Figure S287. ^{1}**H NMR** of the **4ag** (400 MHz, $CDCl_3$)

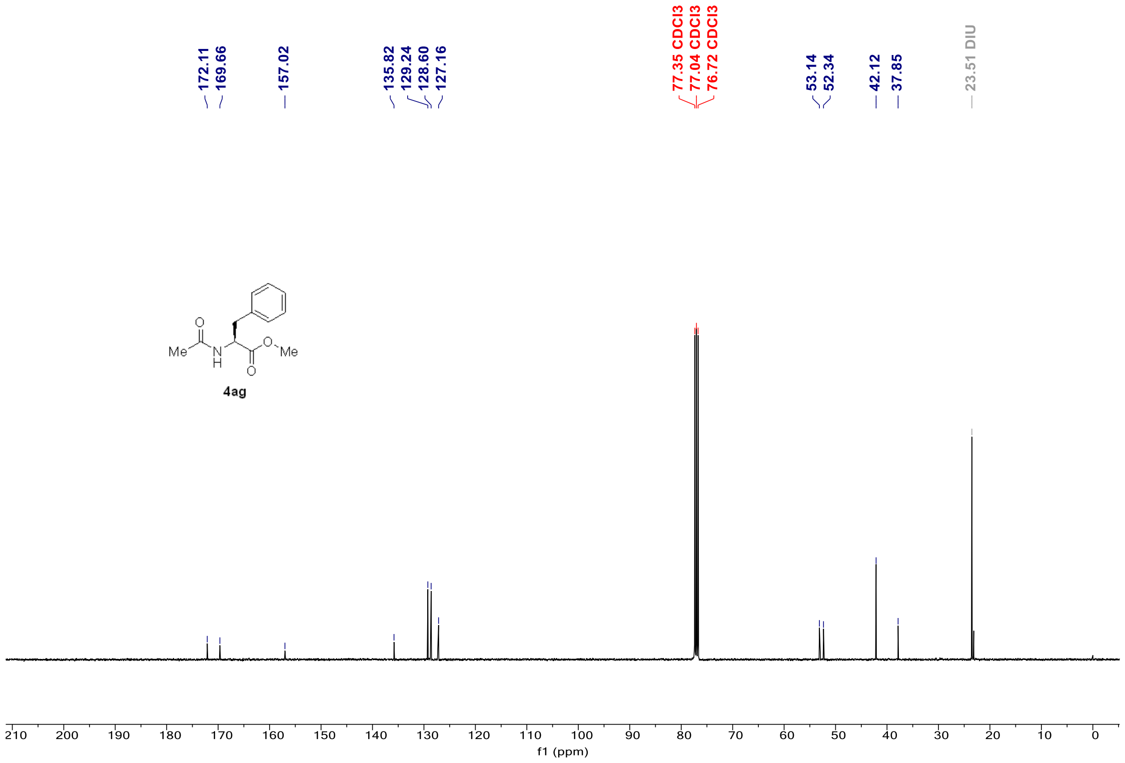

Figure S288. ^{13}**C NMR** of the **4ag** (101 MHz, $CDCl_3$)

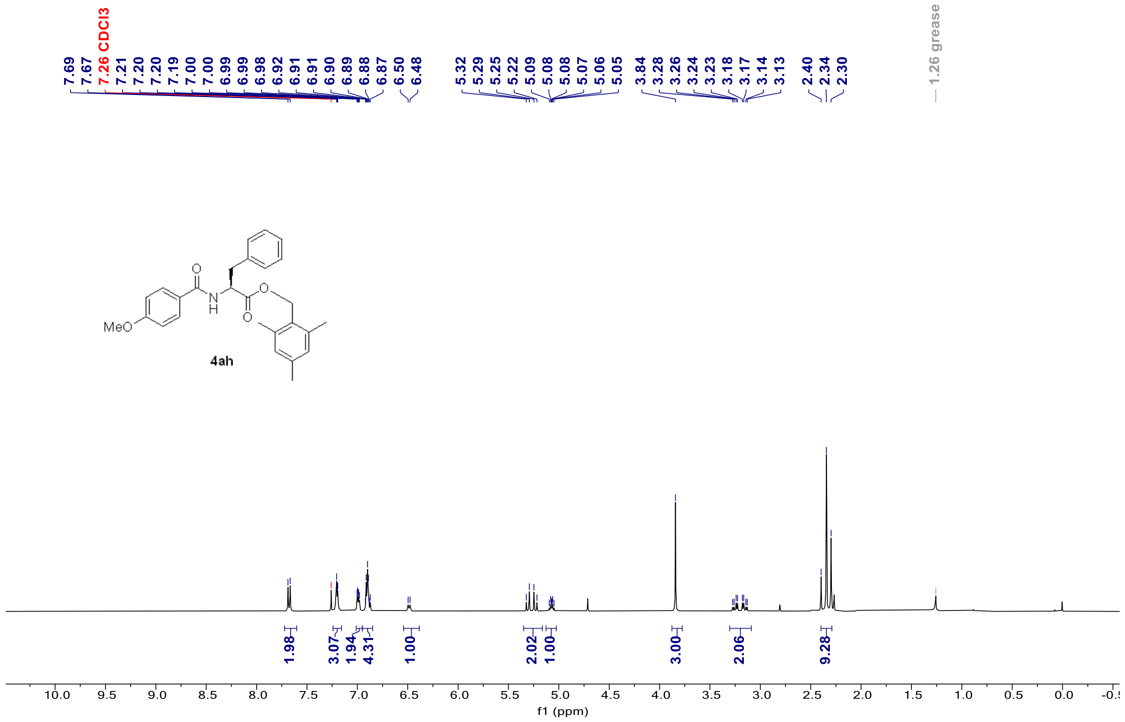

Figure S289. **^{1}H NMR** of the **4ah** (400 MHz, $CDCl_3$)

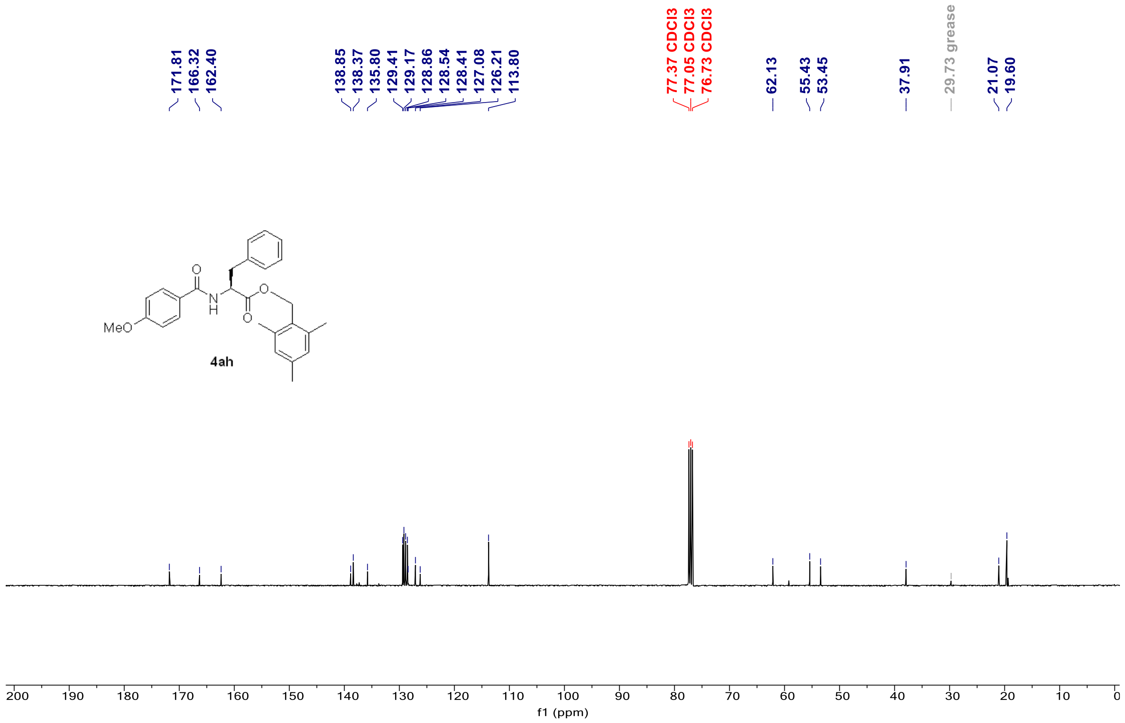

Figure S290. **^{13}C NMR** of the **4ah** (101 MHz, $CDCl_3$)

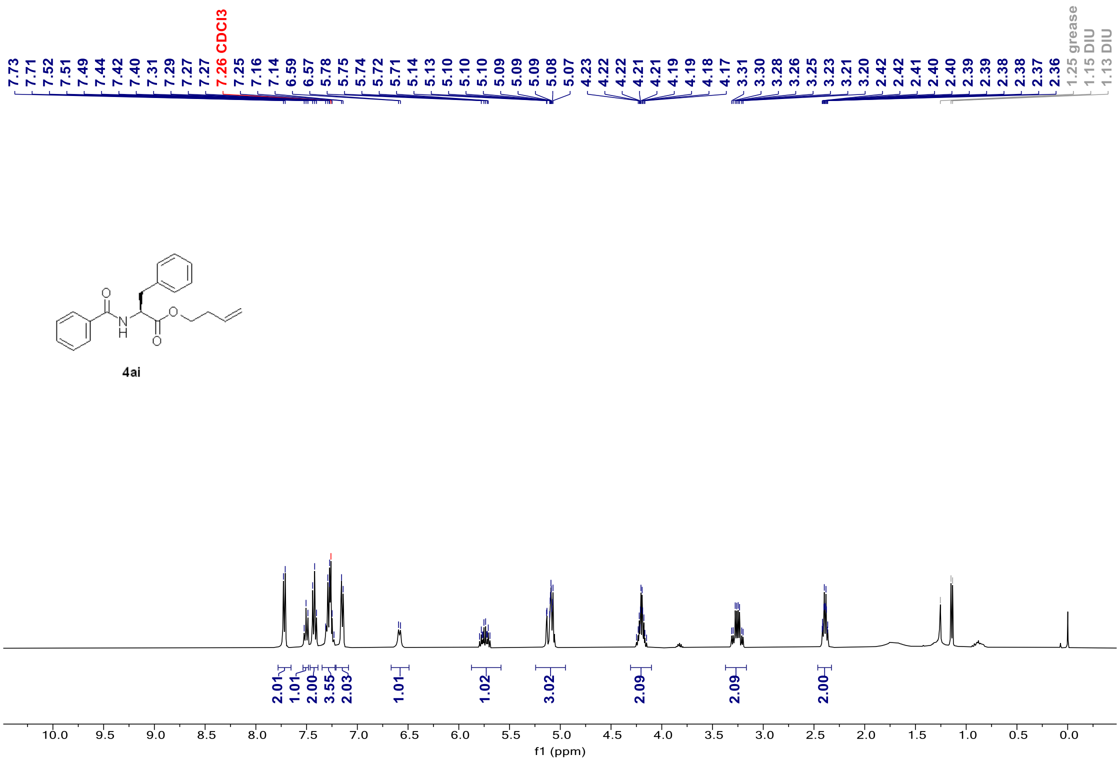

Figure S291. ^{1}**H NMR** of the **4ai** (400 MHz, $CDCl_3$)

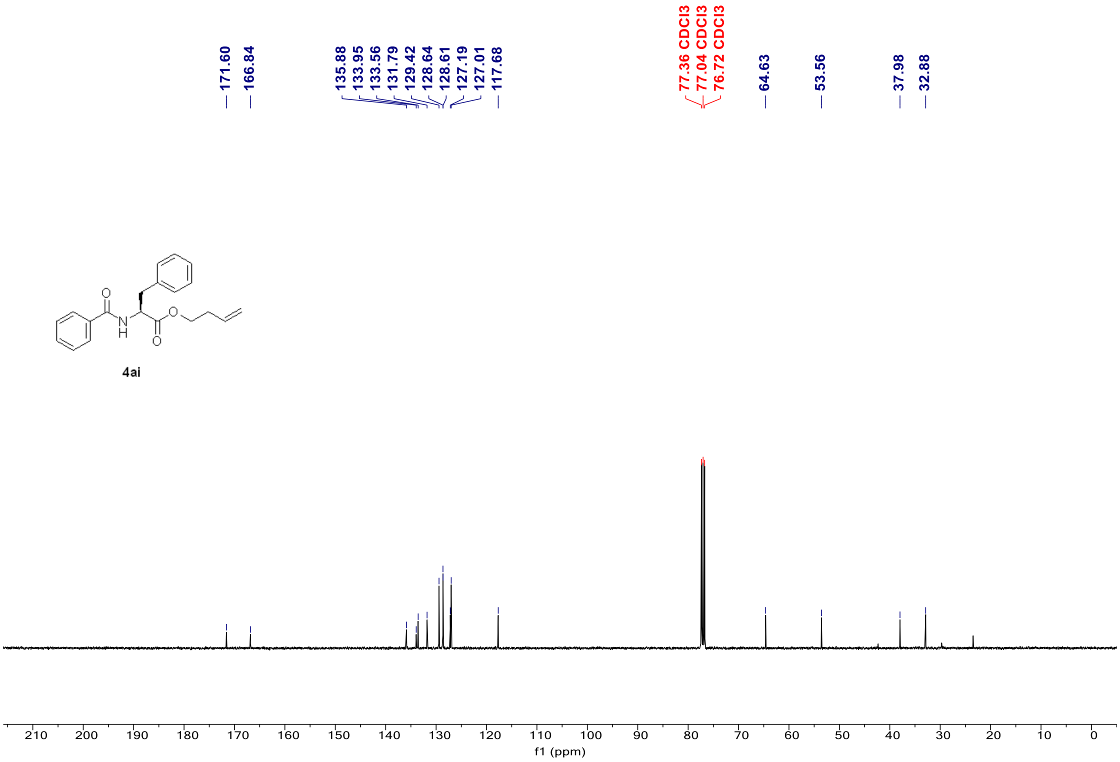

Figure S292. ^{13}**C NMR** of the **4ai** (101 MHz, $CDCl_3$)

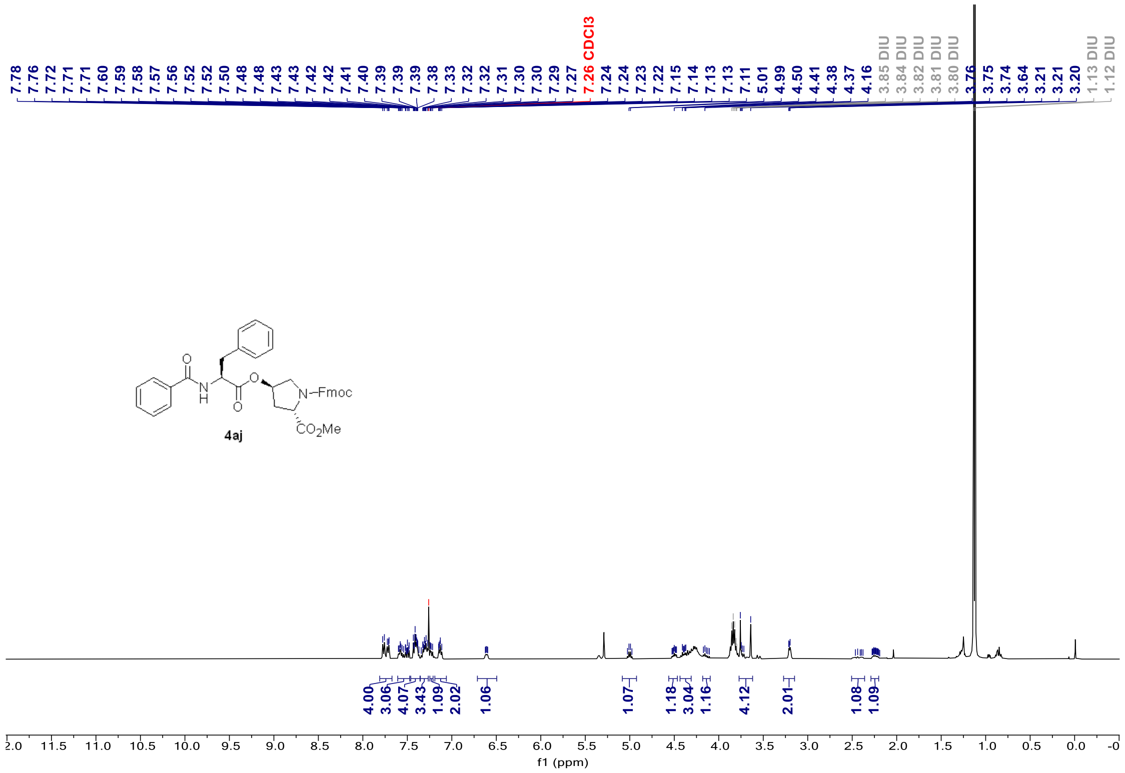

Figure S293. **^{1}H NMR** of the **4aj** (400 MHz, $CDCl_3$)

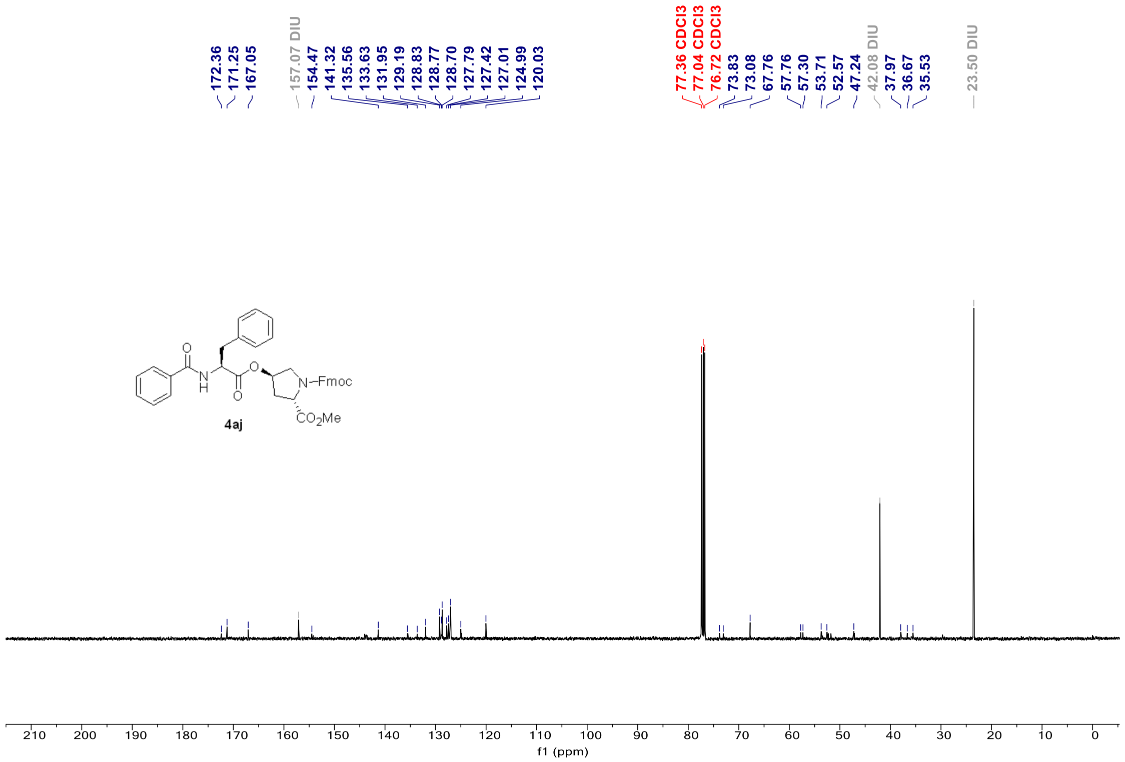

Figure S294. **^{13}C NMR** of the **4aj** (101 MHz, $CDCl_3$)

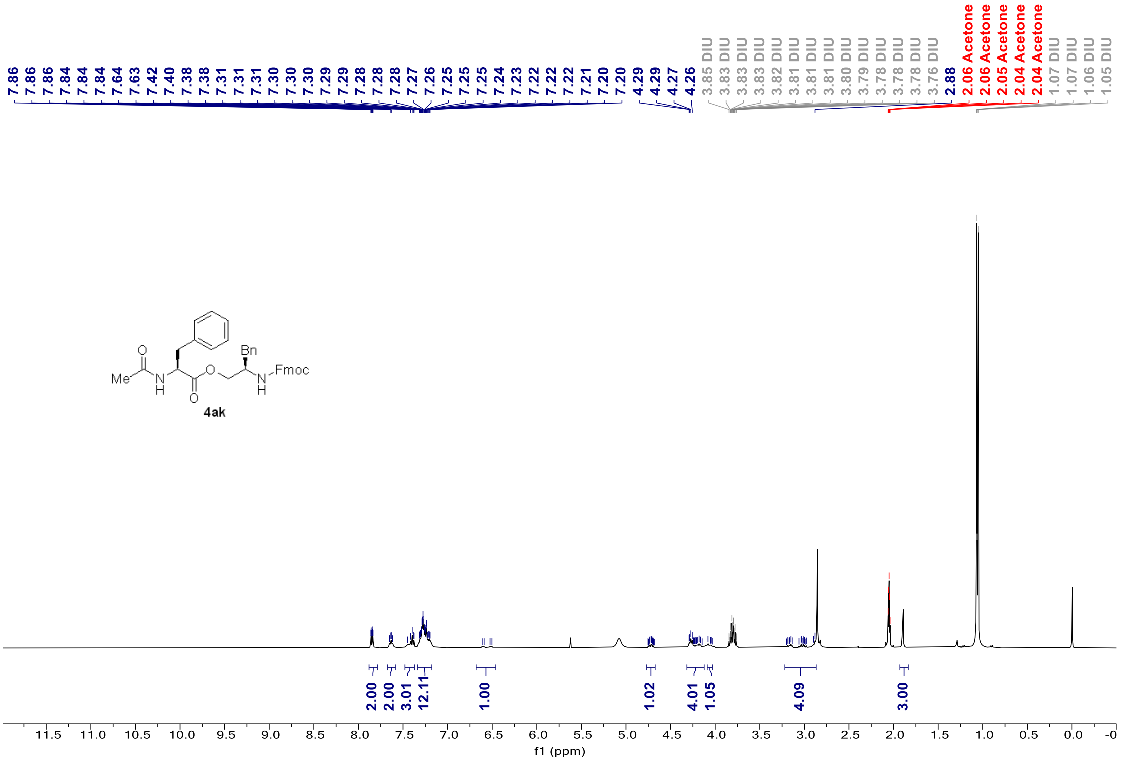

Figure S295. **^{1}H NMR** of the **4ak** (400 MHz, Acetone-d_6)

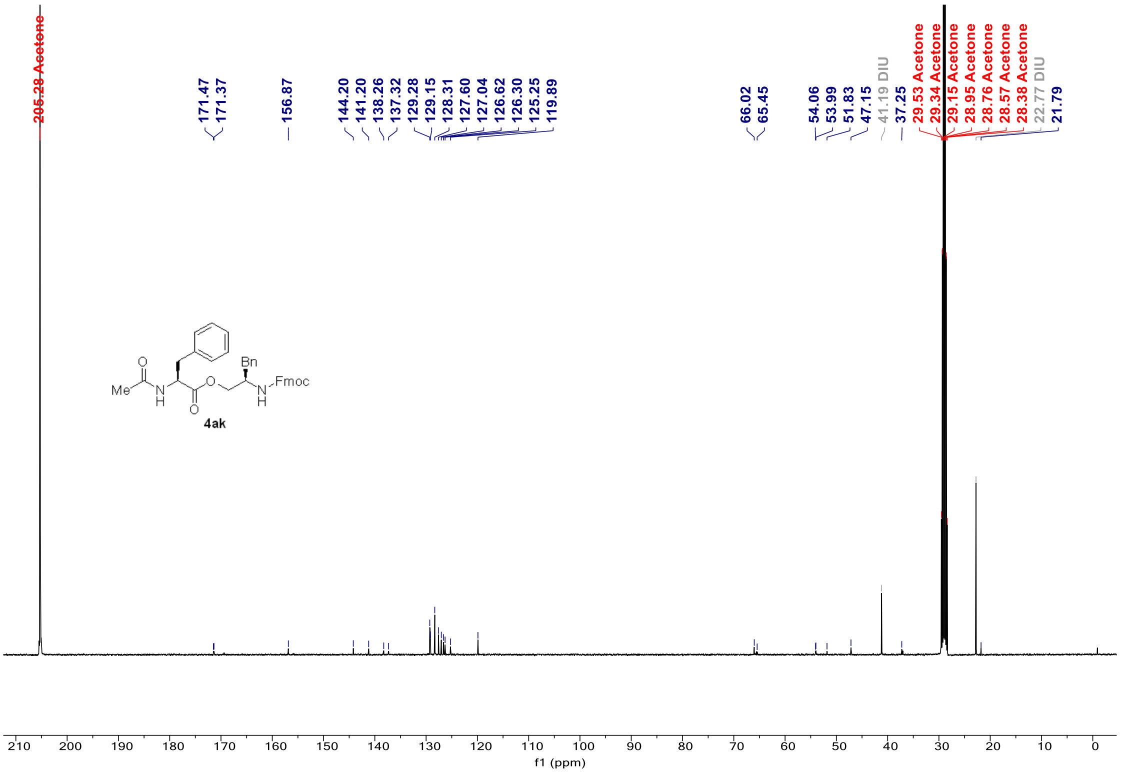

Figure S296. **^{13}C NMR** of the **4ak** (101 MHz, Acetone-d_6)

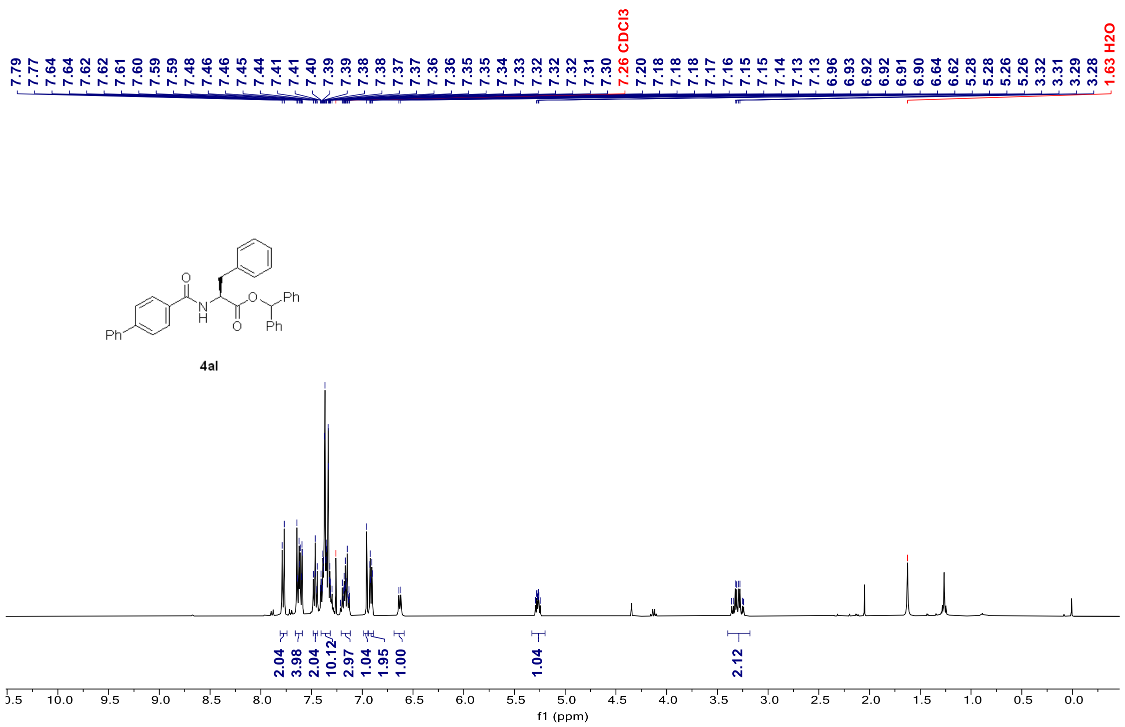

Figure S297. **^{1}H NMR** of the **4al** (400 MHz, $CDCl_3$)

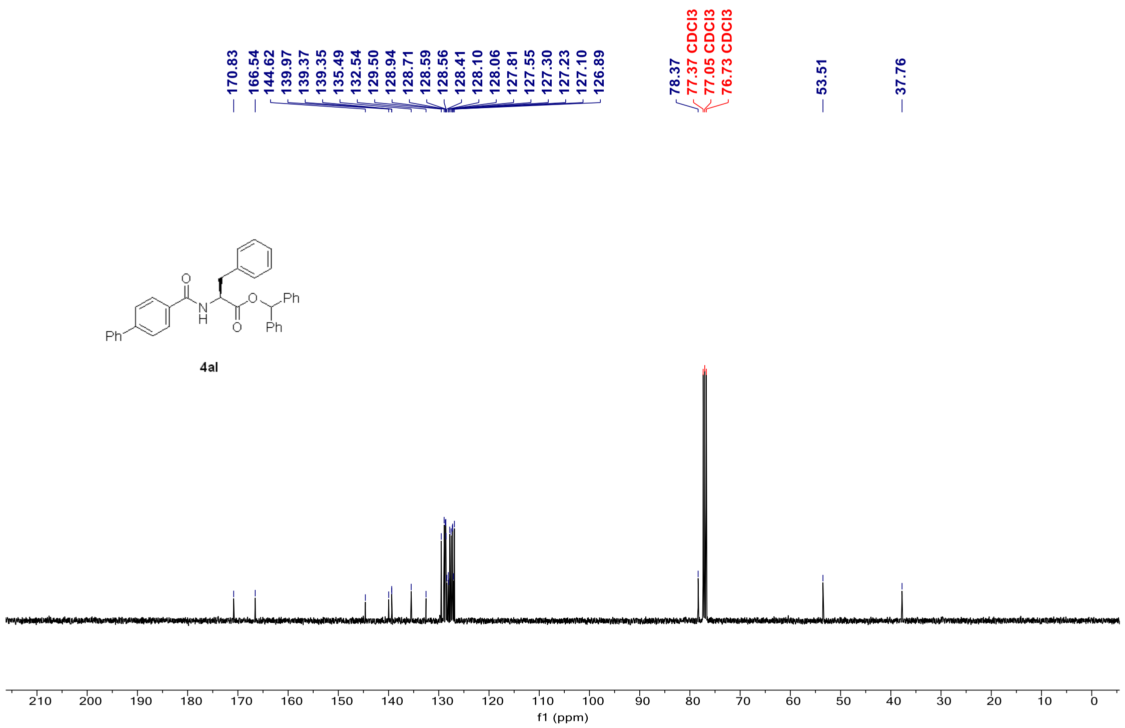

Figure S298. **^{13}C NMR** of the **4al** (101 MHz, $CDCl_3$)

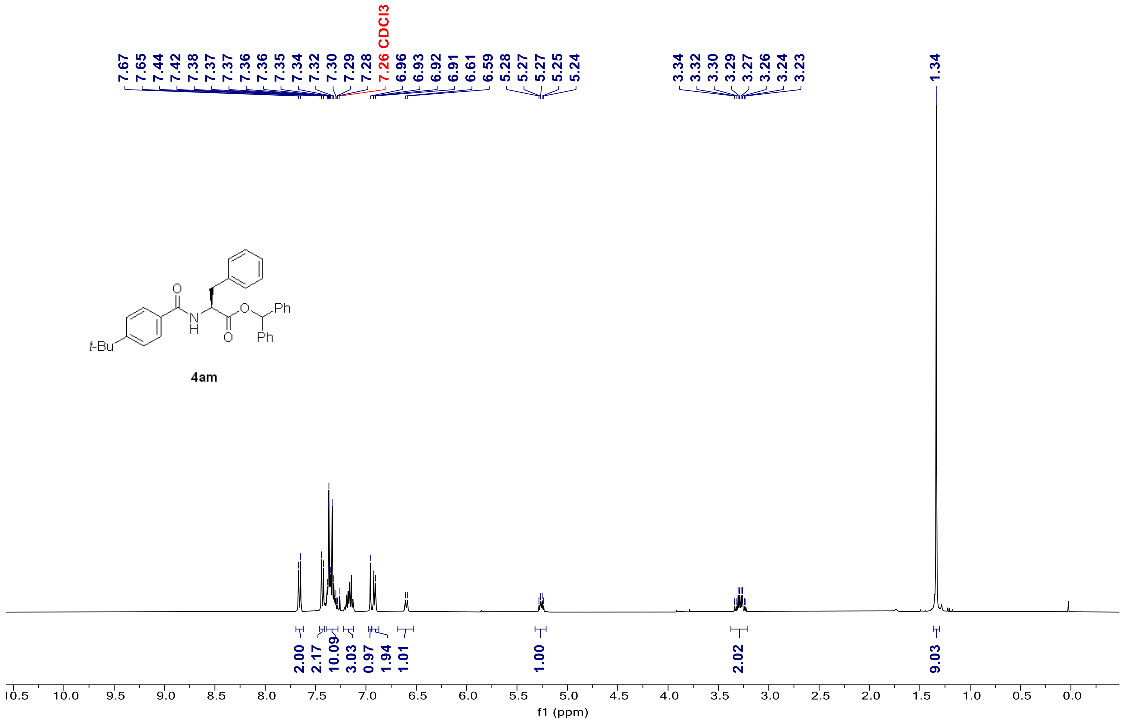

Figure S299. ^{1}**H NMR** of the **4am** (400 MHz, $CDCl_3$)

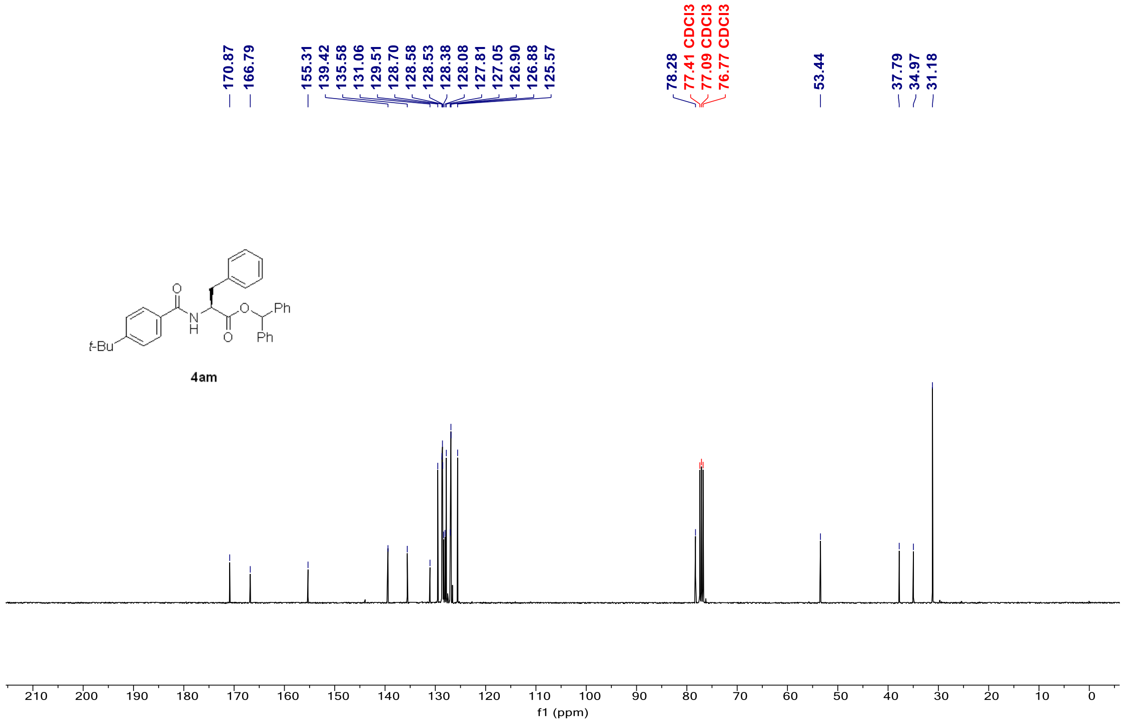

Figure S300. ^{13}**C NMR** of the **4am** (101 MHz, $CDCl_3$)

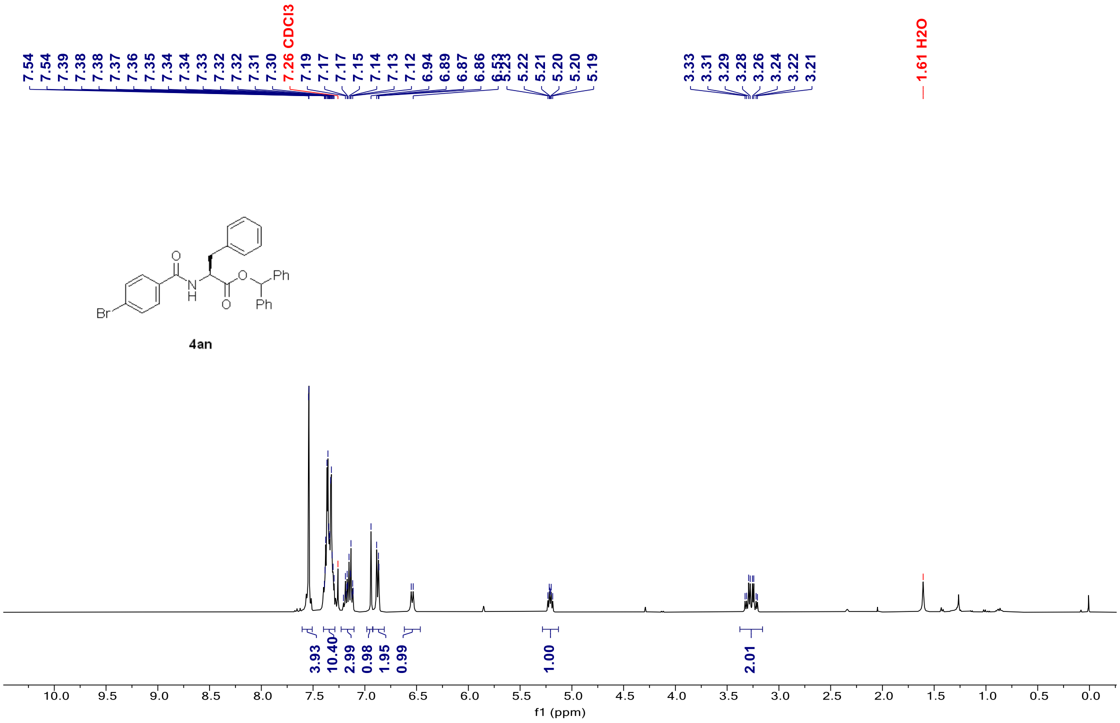

Figure S301. **^{1}H NMR** of the **4an** (400 MHz, CDCl$_3$**)**

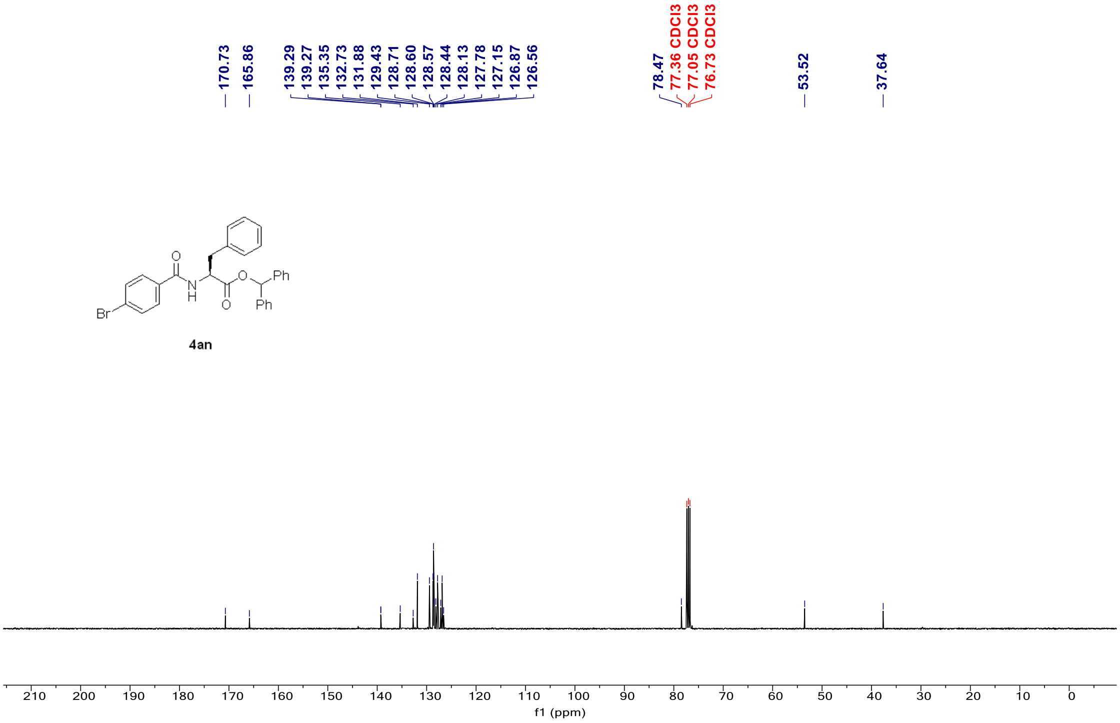

Figure S302. **^{13}C NMR** of the **4an** (101 MHz, CDCl$_3$**)**

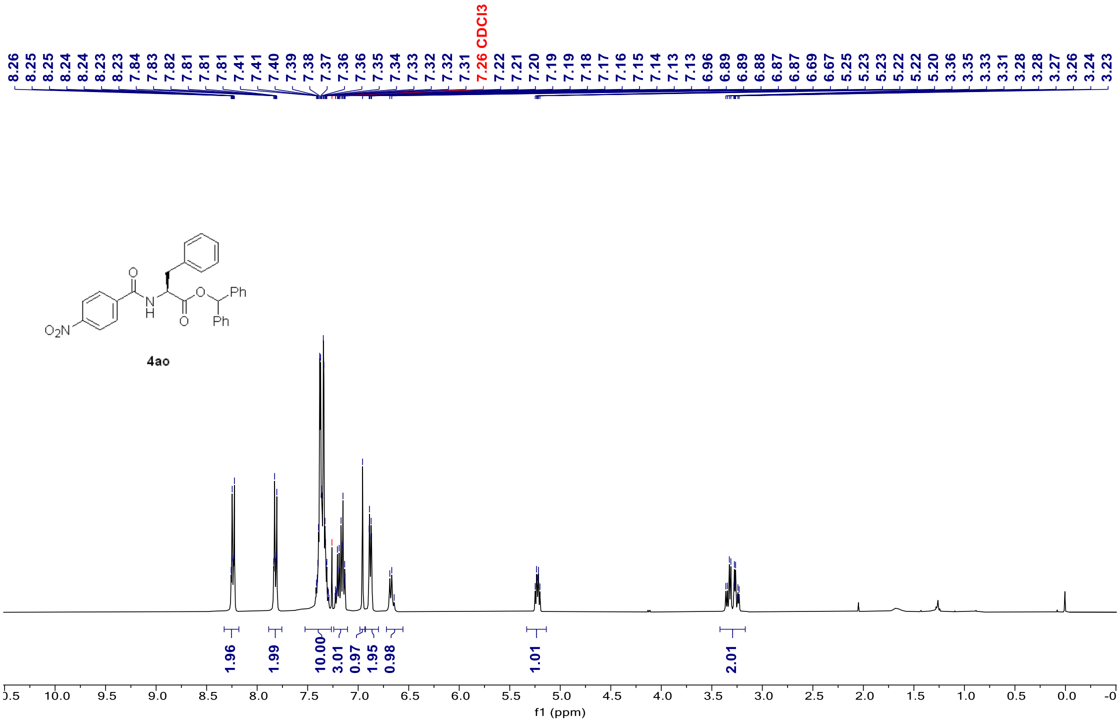

Figure S303. **^{1}H NMR** of the **4ao** (400 MHz, $CDCl_3$)

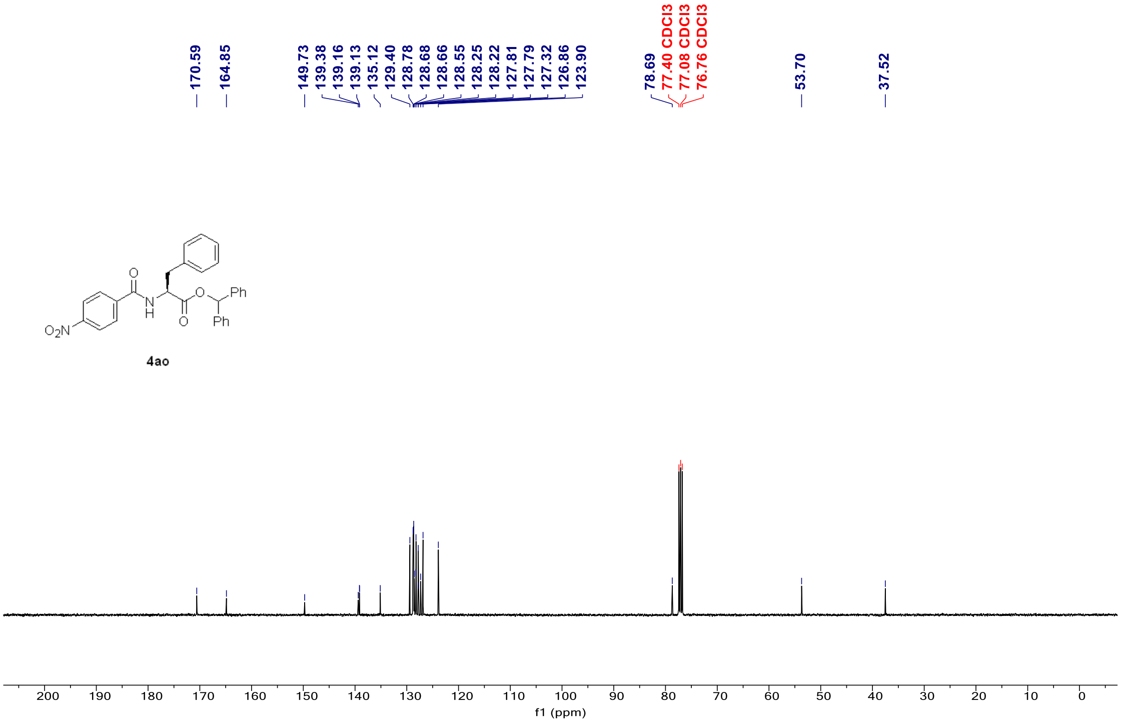

Figure S304. **^{13}C NMR** of the **4ao** (101 MHz, $CDCl_3$)

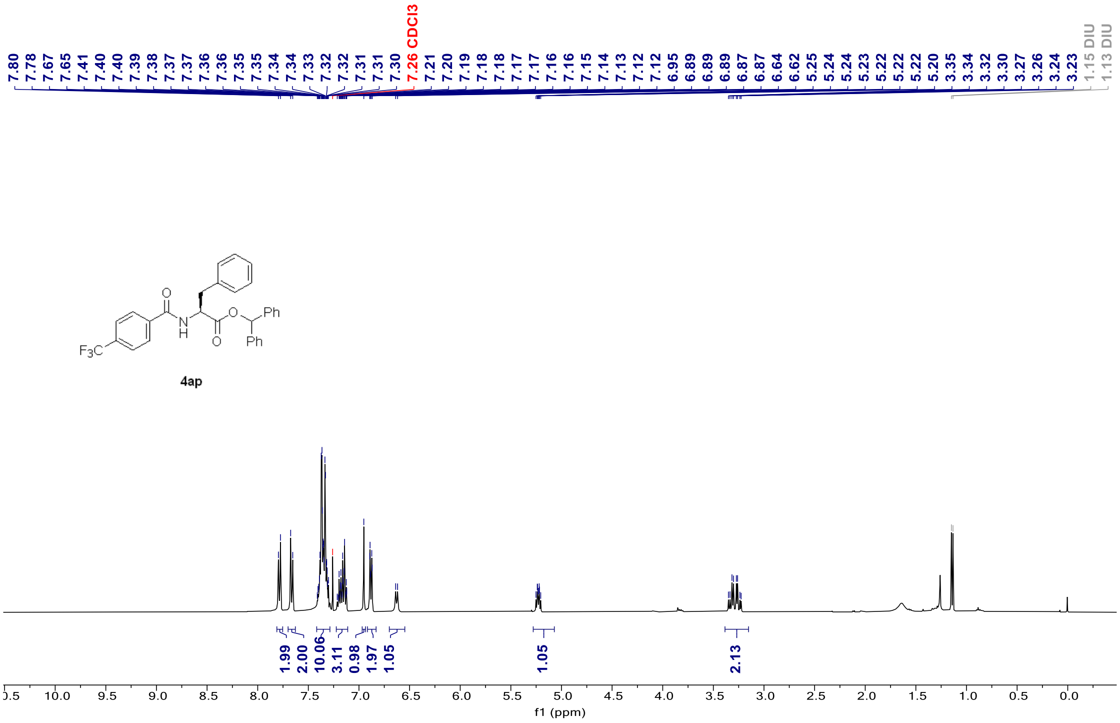

Figure S305. ^{1}H NMR of the **4ap** (400 MHz, $CDCl_3$)

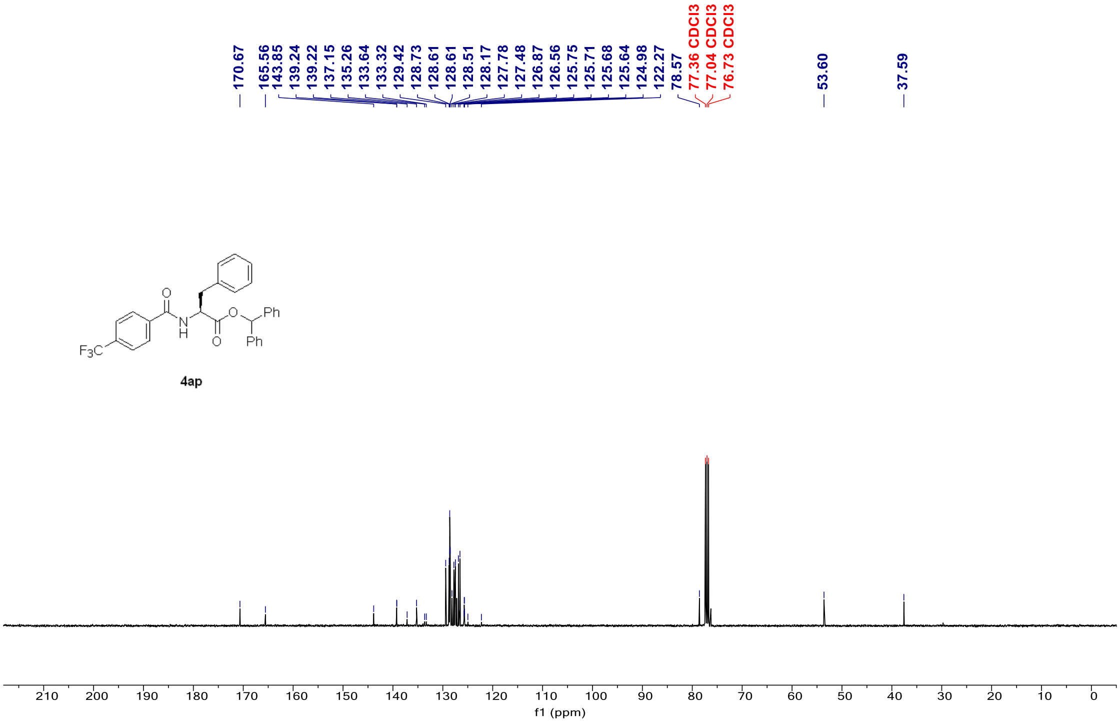

Figure S306. ^{13}C NMR of the **4ap** (101 MHz, $CDCl_3$)

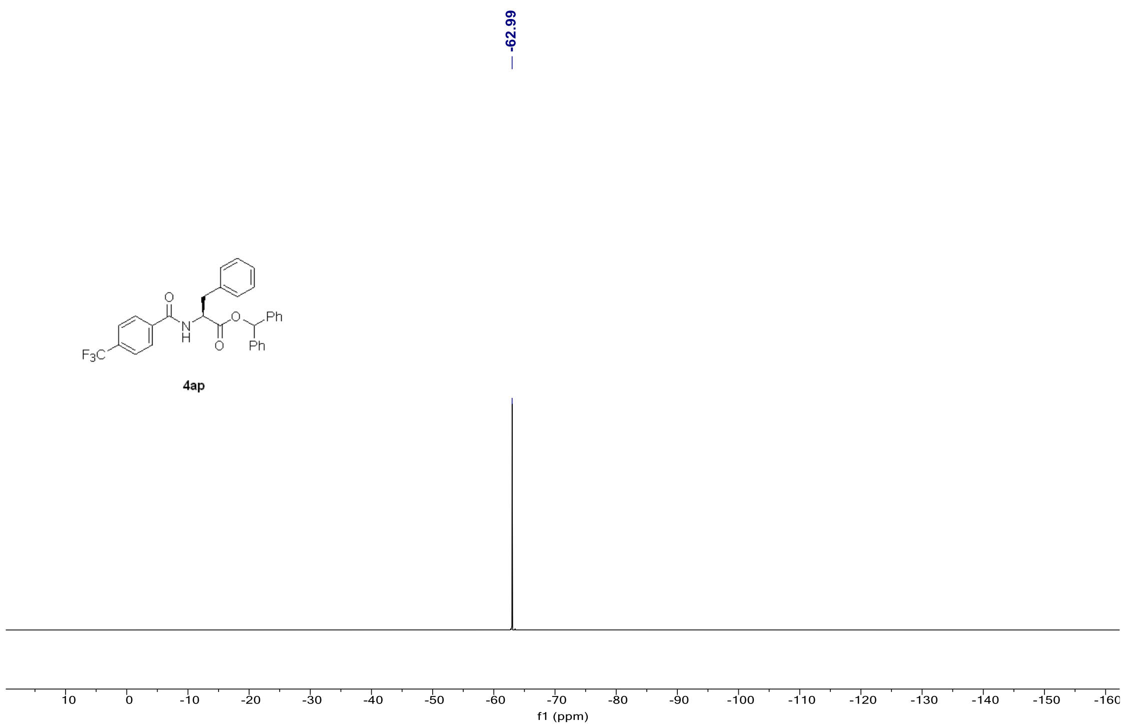

Figure S307. ^{19}F NMR of the **4ap** (376 MHz, $CDCl_3$)

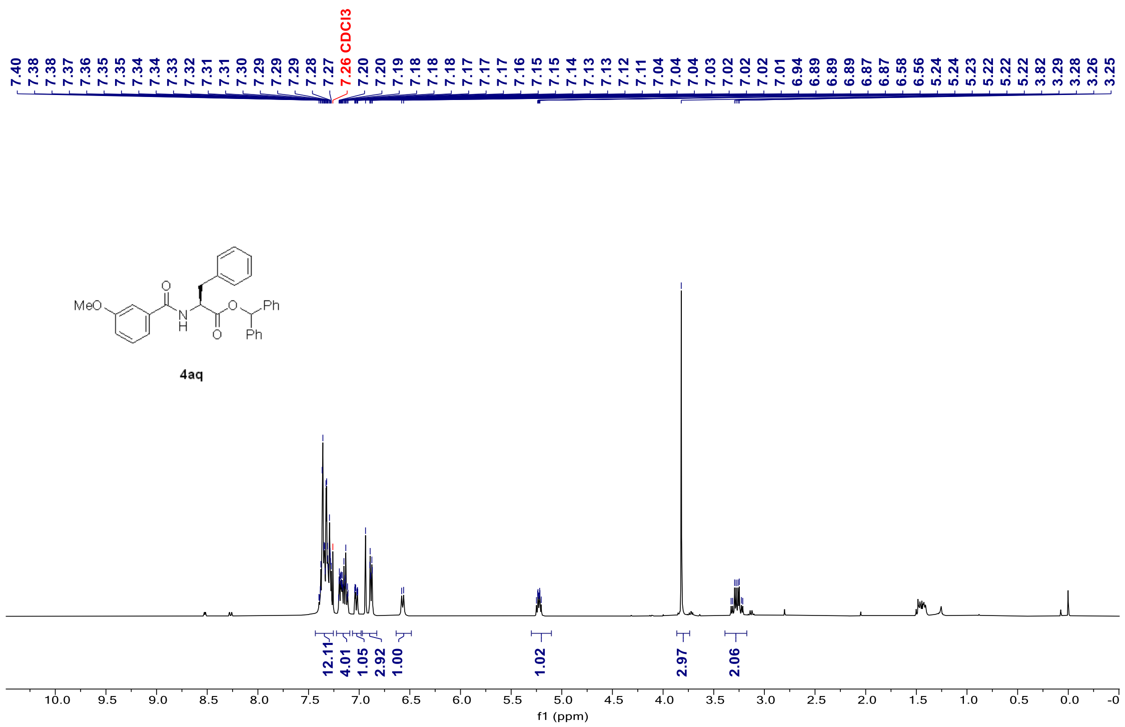

Figure S308. **^{1}H NMR** of the **4aq** (400 MHz, $CDCl_3$)

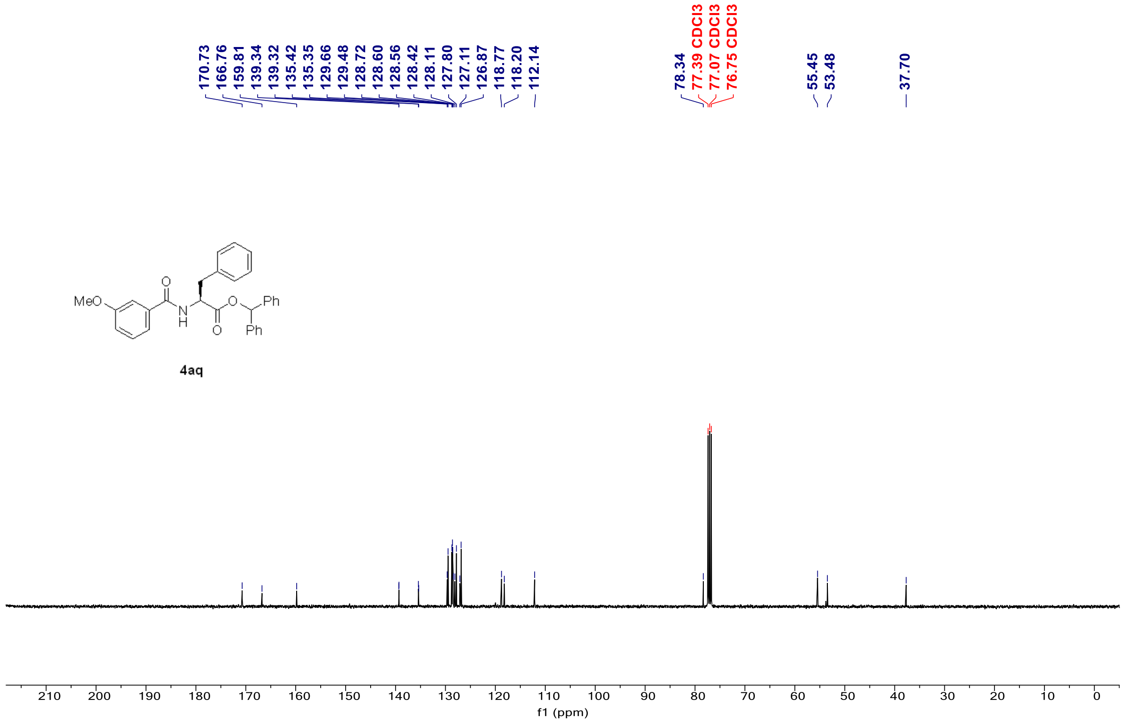

Figure S309. **^{13}C NMR** of the **4aq** (101 MHz, $CDCl_3$)

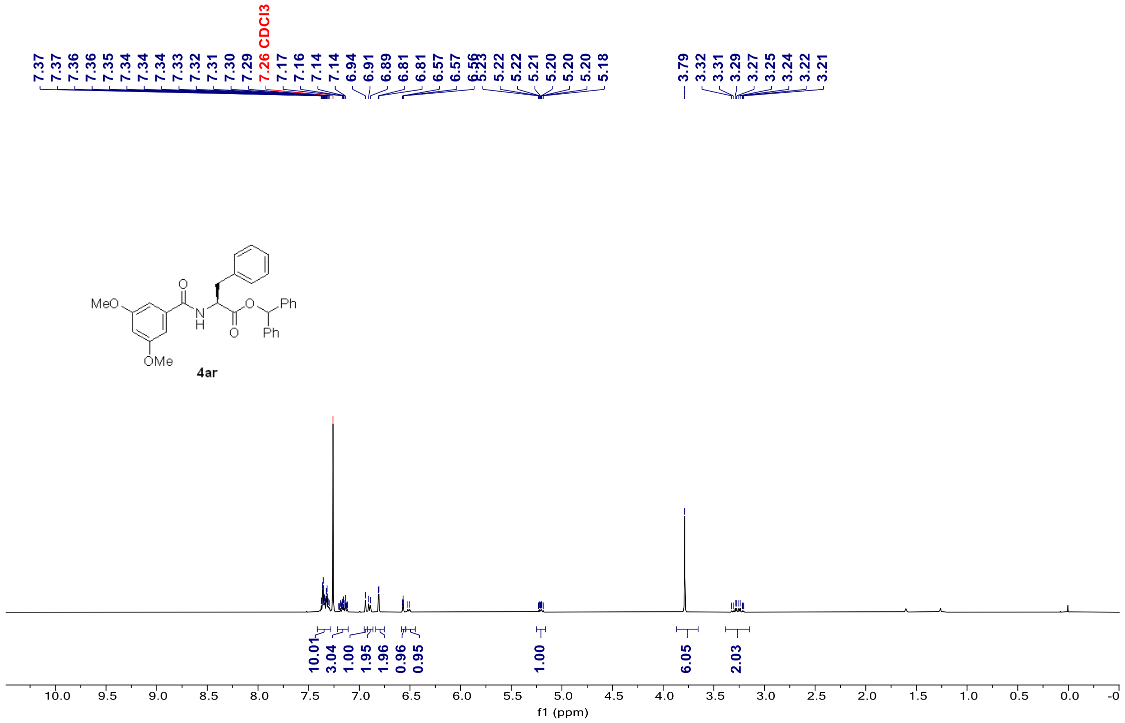

Figure S310. **^{1}H NMR** of the **4ar** (400 MHz, $CDCl_3$)

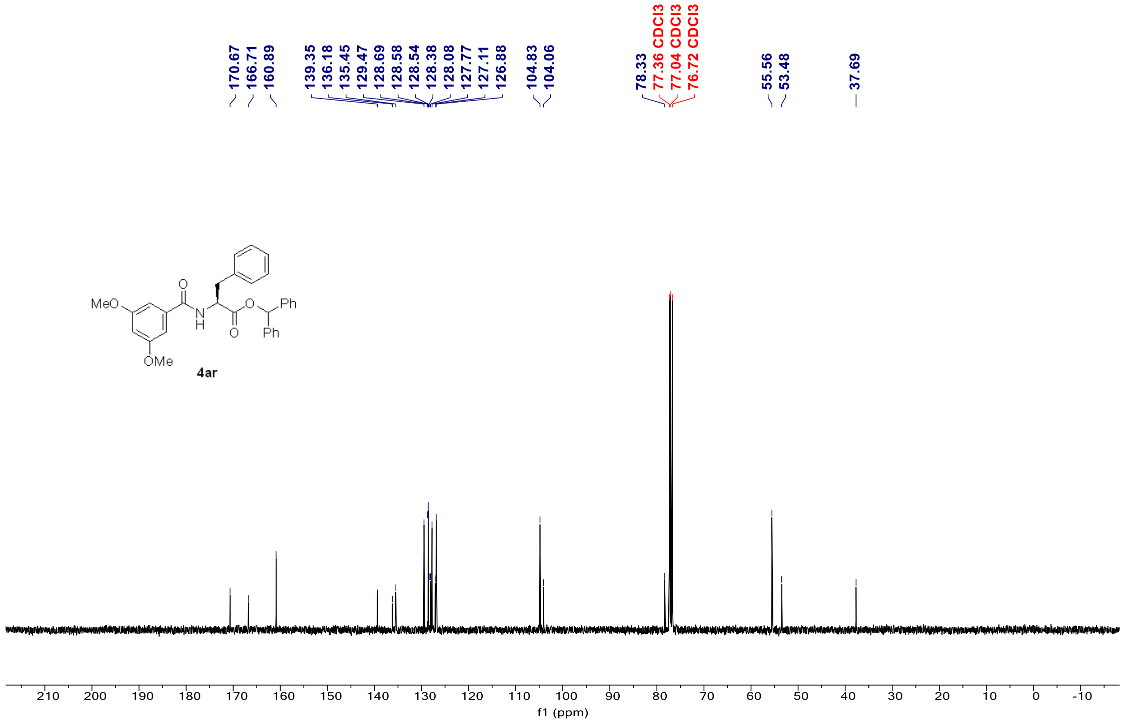

Figure S311. **^{13}C NMR** of the **4ar** (101 MHz, $CDCl_3$)

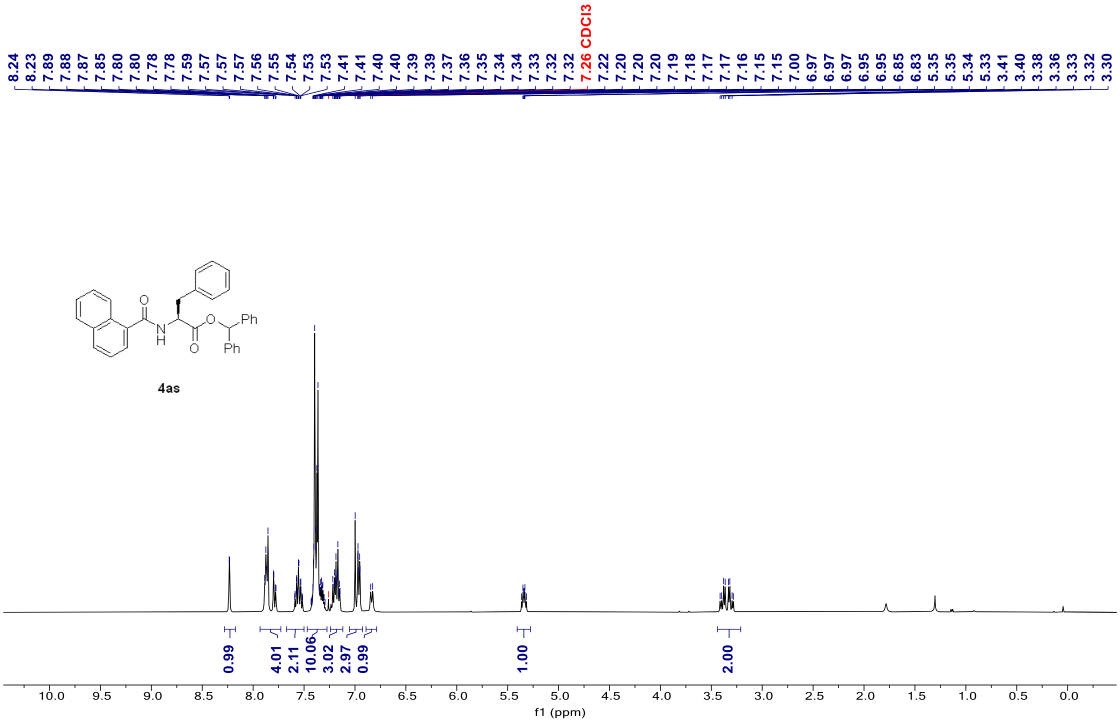

Figure S312. ¹H NMR of the **4as** (400 MHz, $CDCl_3$**)**

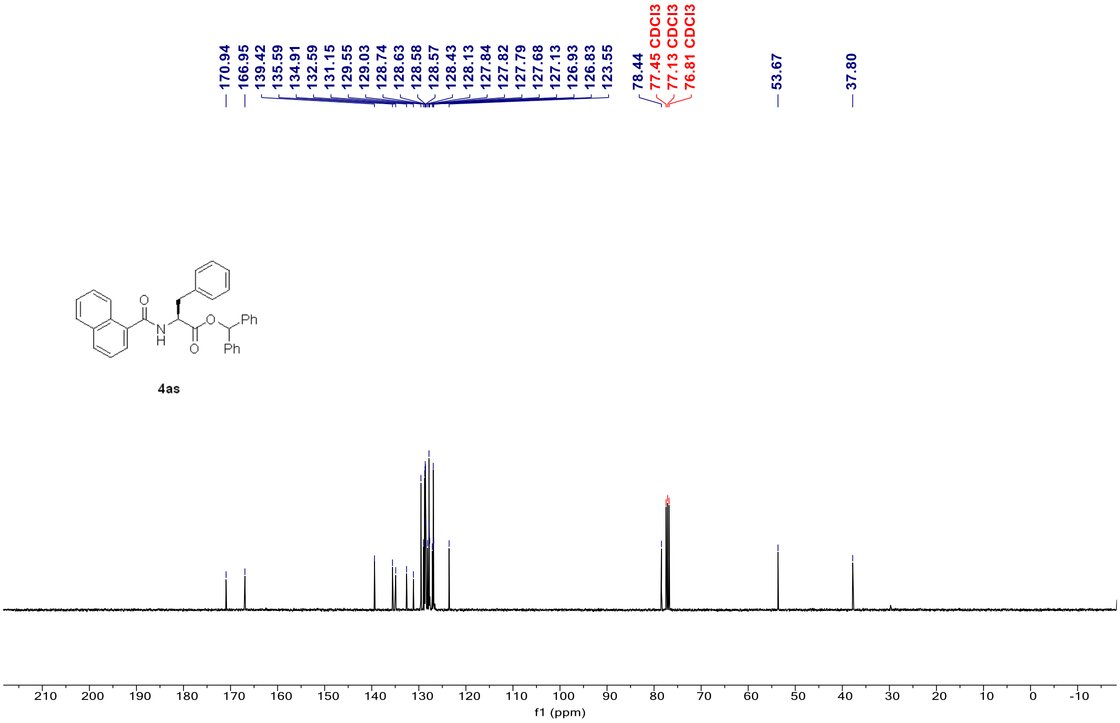

Figure S313. ¹³C NMR of the **4as** (101 MHz, $CDCl_3$**)**

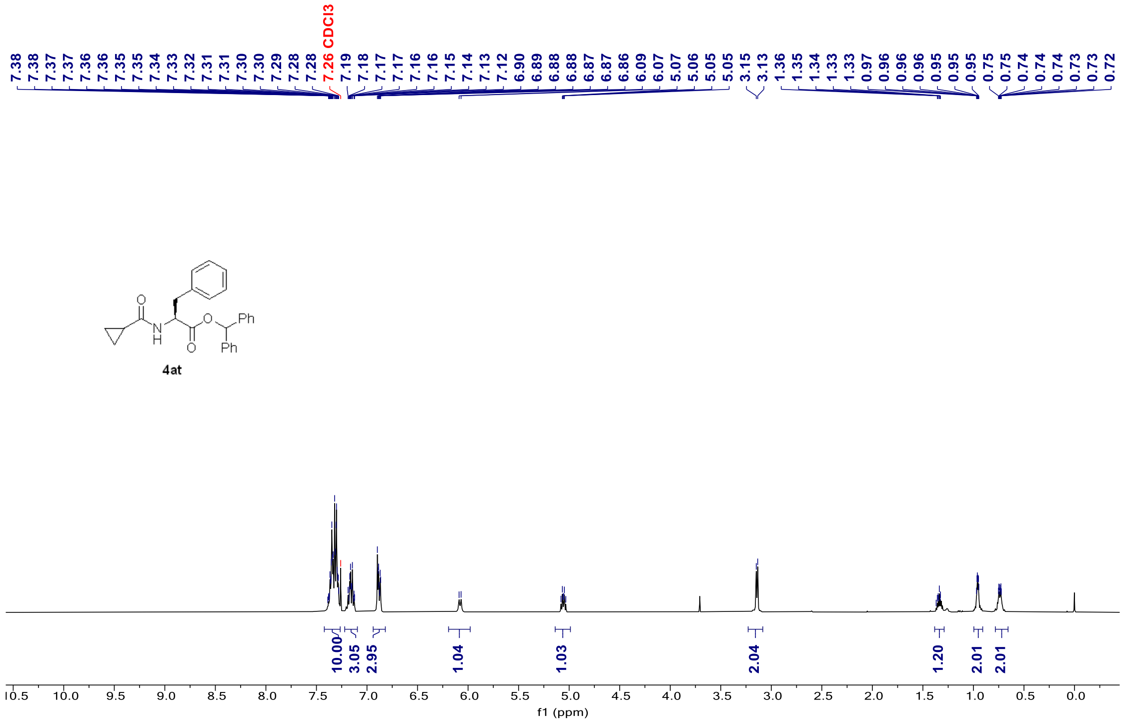

Figure S314. ^{1}**H NMR** of the **4at** (400 MHz, $CDCl_3$)

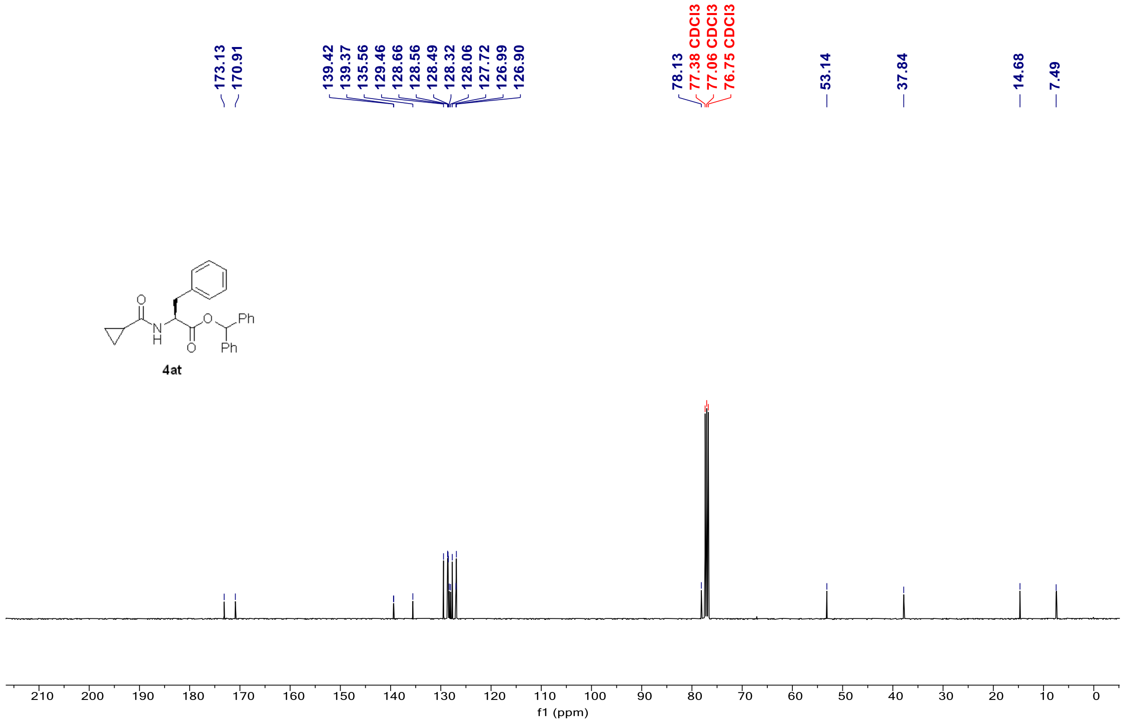

Figure S315. ^{13}**C NMR** of the **4at** (101 MHz, $CDCl_3$)

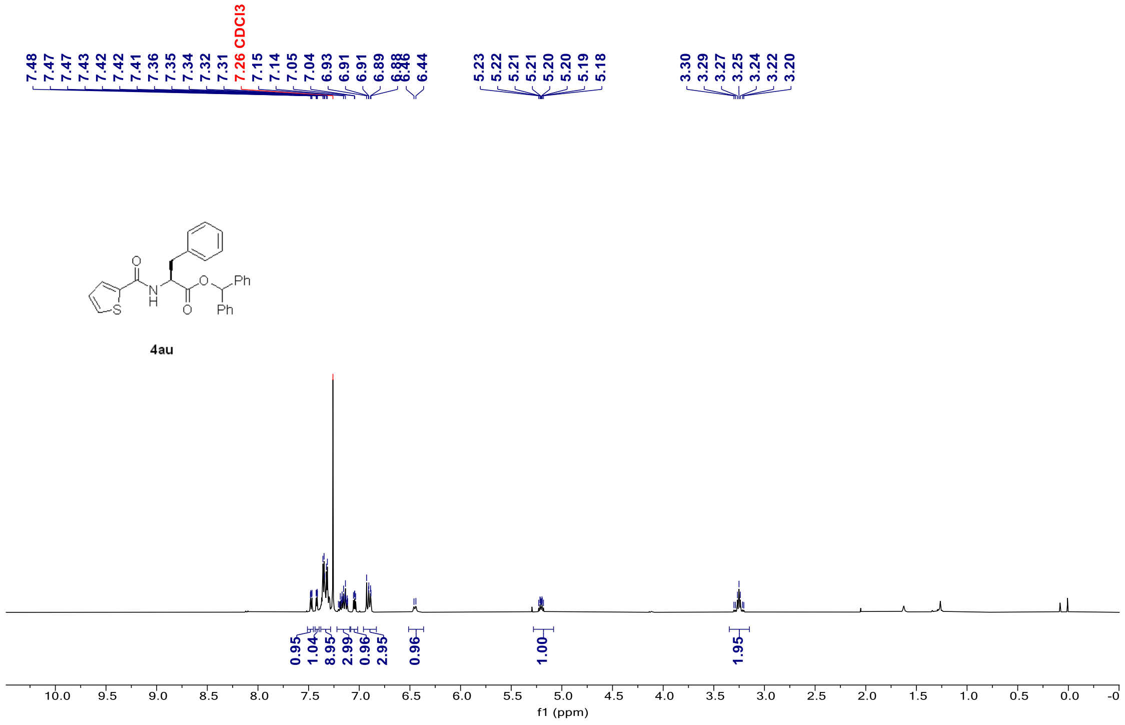

Figure S316. **^{1}H NMR** of the **4au** (400 MHz, $CDCl_3$)

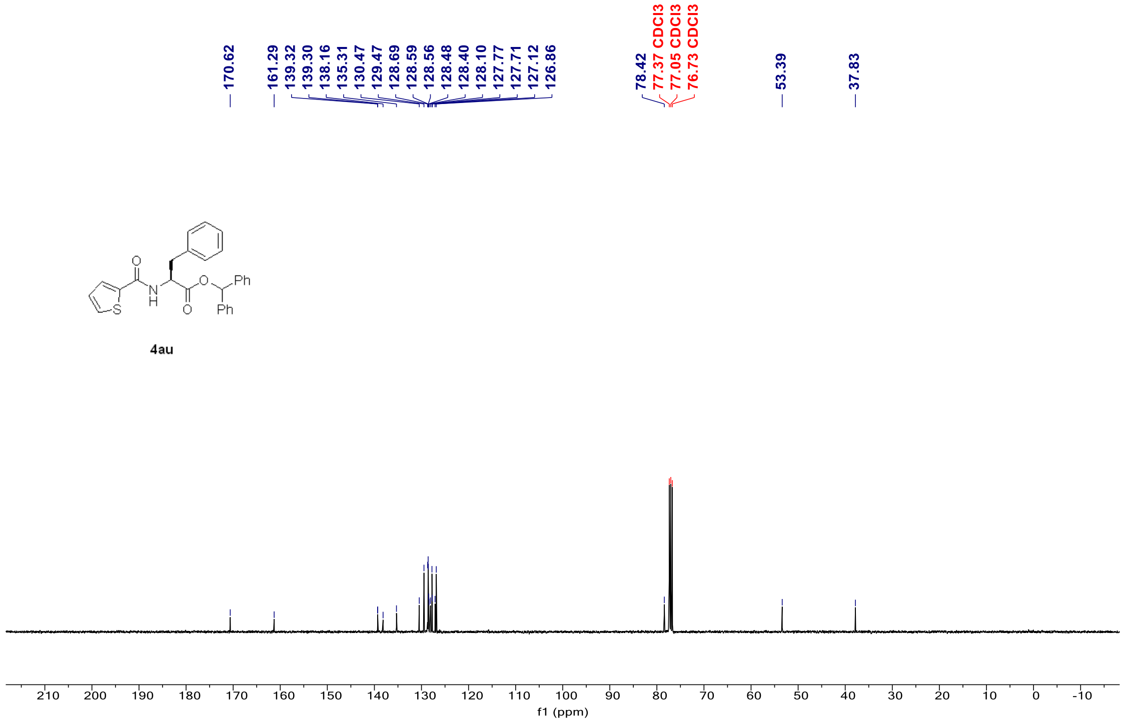

Figure S317. **^{13}C NMR** of the **4au** (101 MHz, $CDCl_3$)

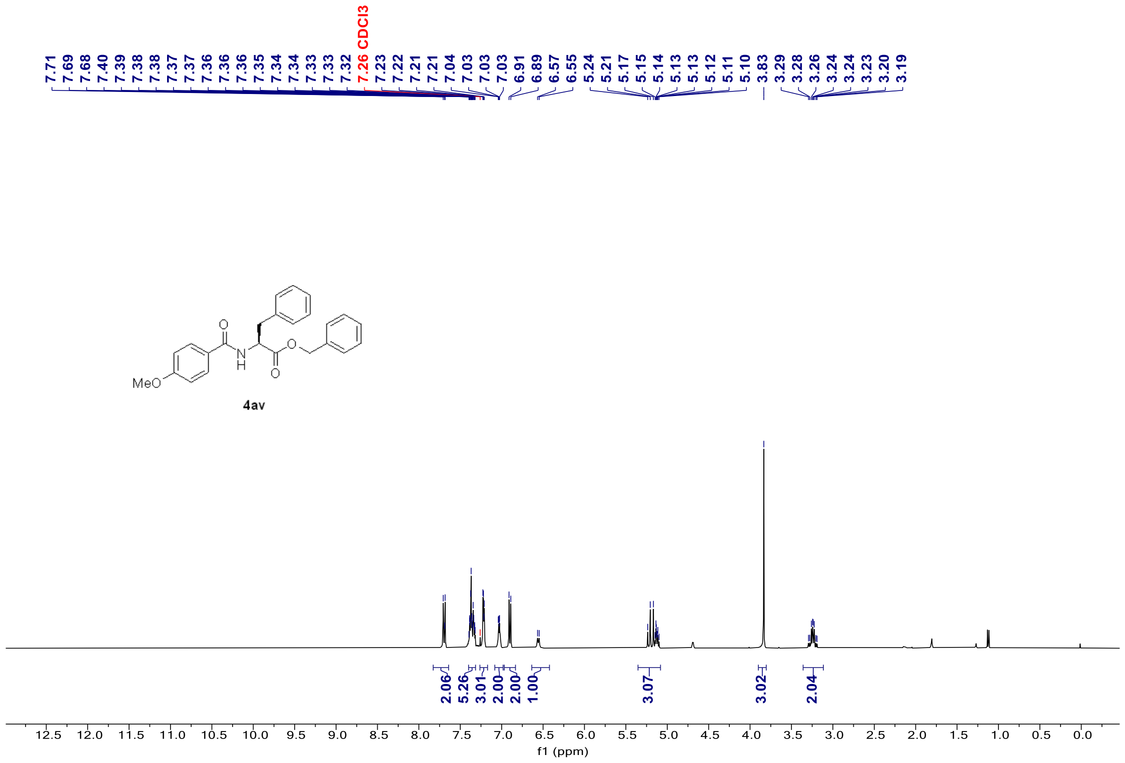

Figure S318. **^{1}H NMR** of the **4av** (400 MHz, $CDCl_3$)

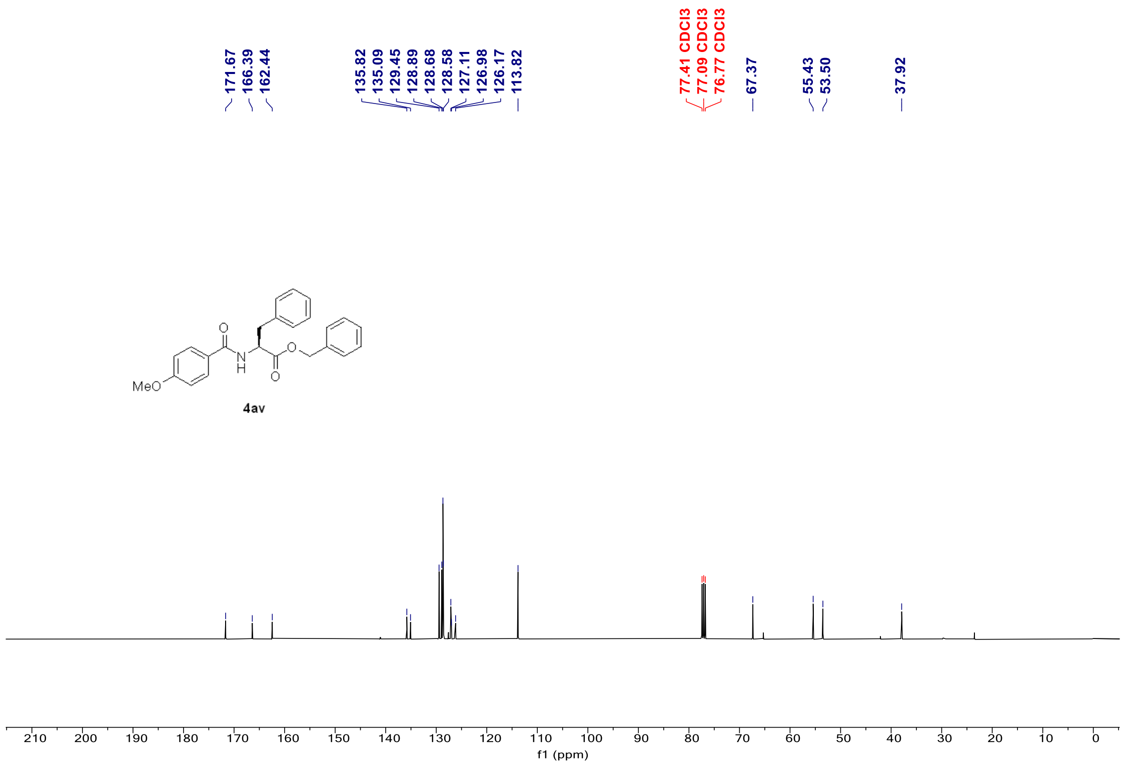

Figure S319. **^{13}C NMR** of the **4av** (101 MHz, $CDCl_3$)

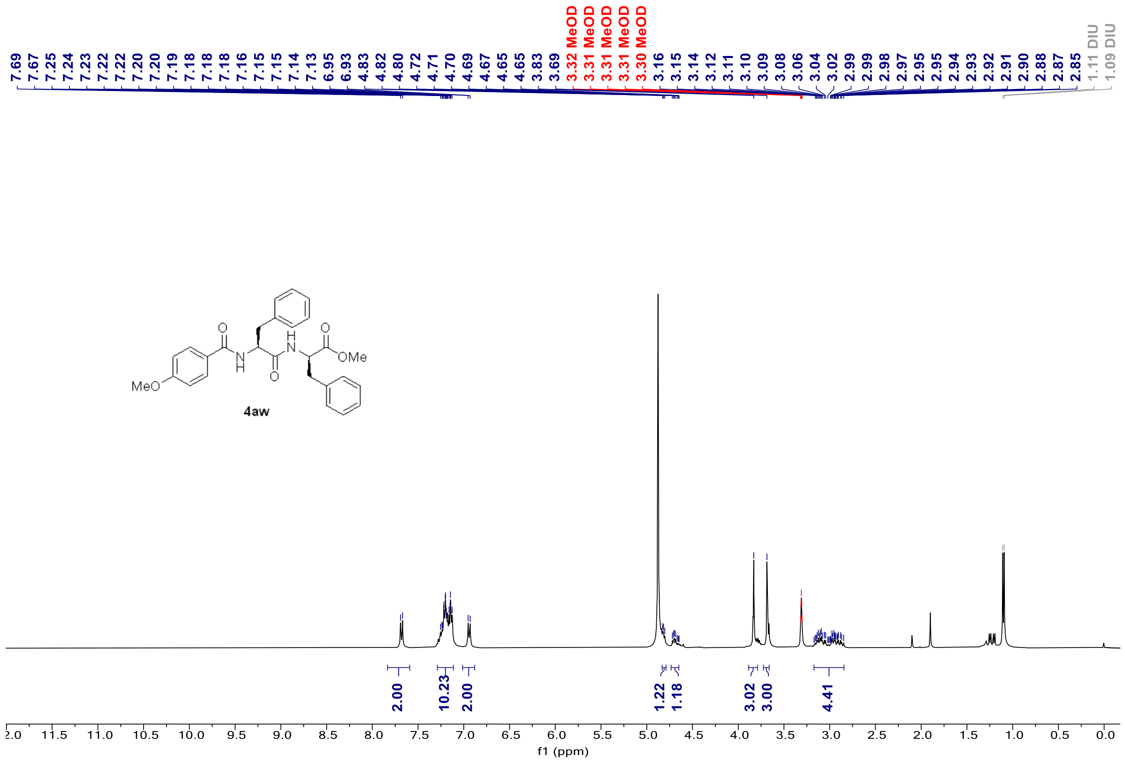

Figure S320. ^{1}**H NMR** of the **4aw** (400 MHz, Methanol-d_4)

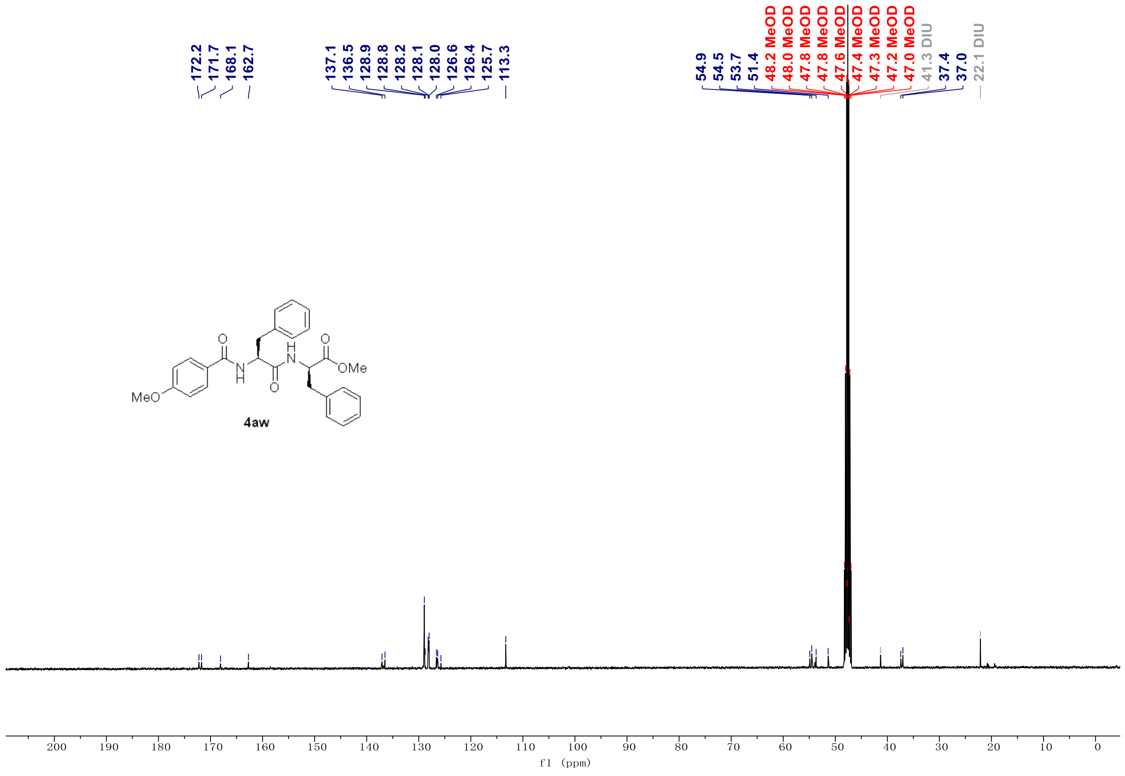

Figure S321. ^{13}**C NMR** of the **4aw** (101 MHz, Methanol-d_4)

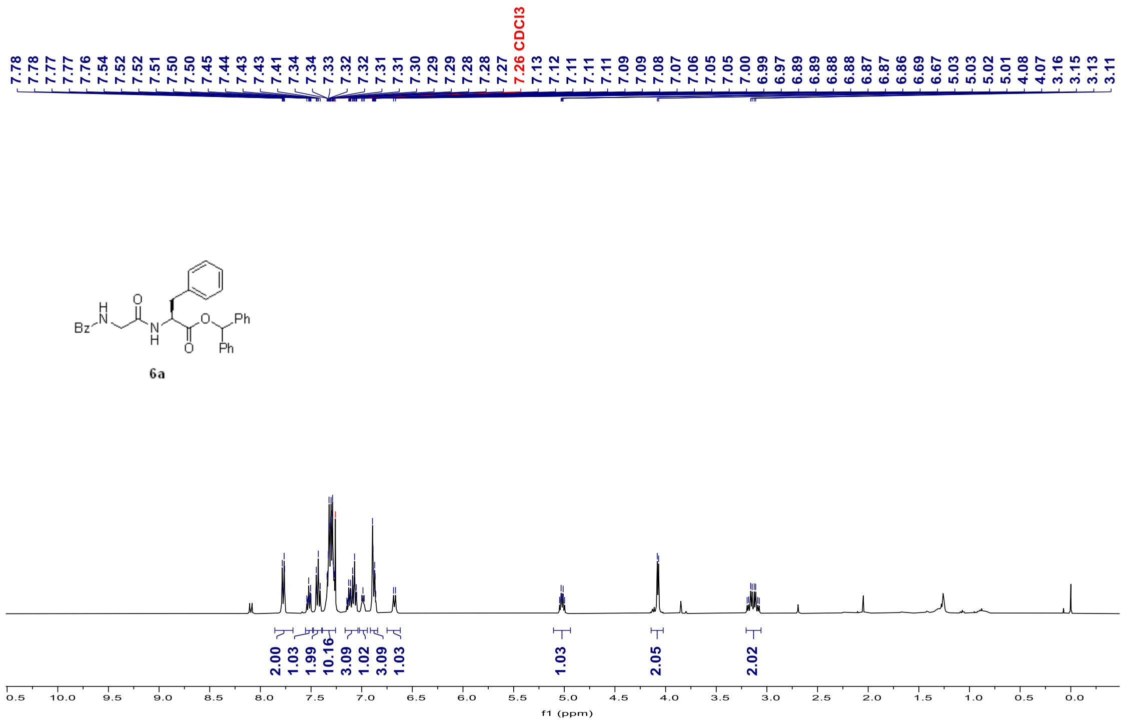

Figure S322. **^{1}H NMR** of the **6a** (400 MHz, $CDCl_3$)

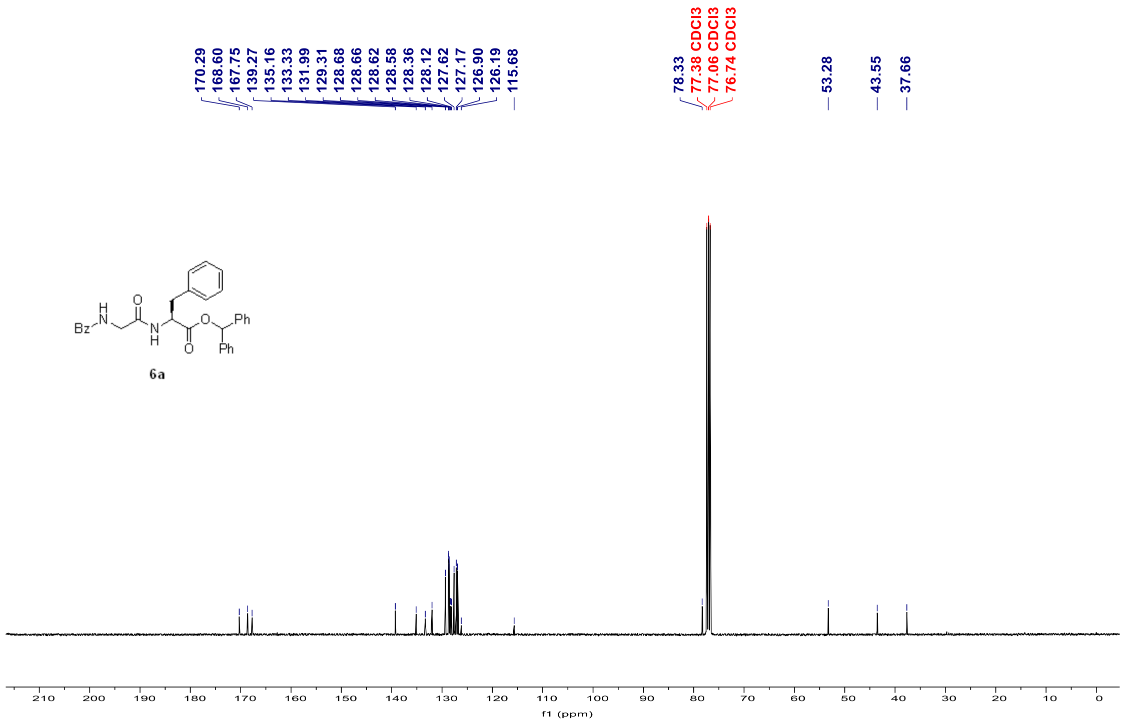

Figure S323. **^{13}C NMR** of the **6a** (101 MHz, $CDCl_3$)

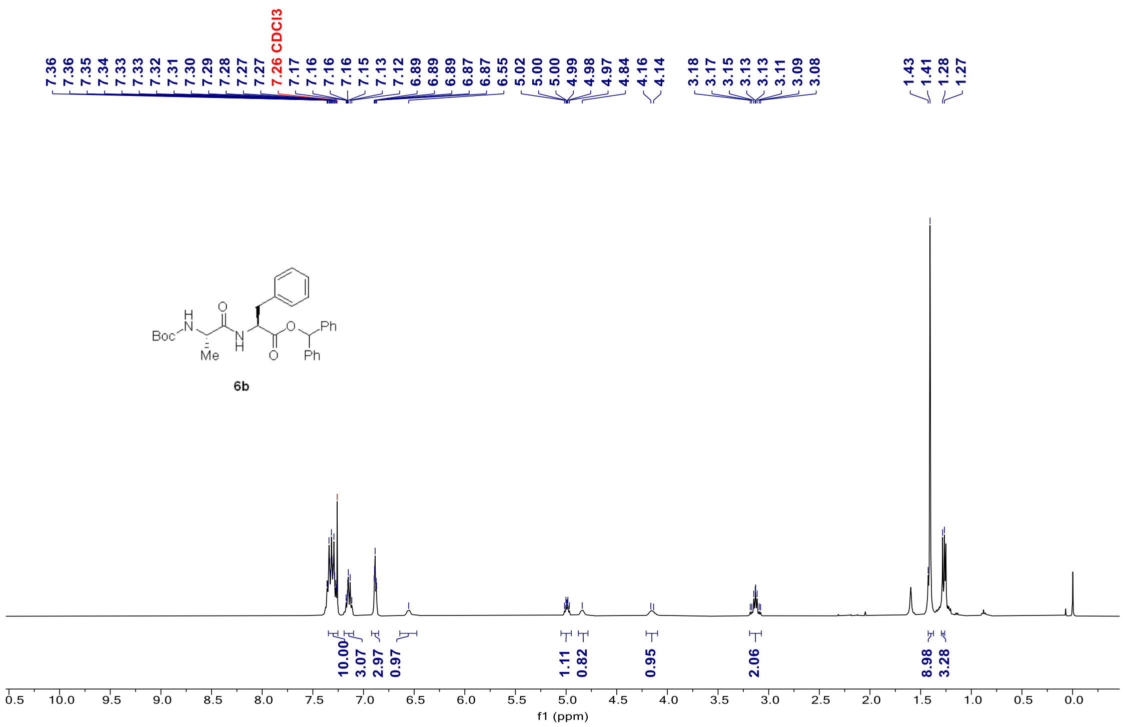

Figure S324. **^{1}H NMR** of the **6b** (400 MHz, CDCl3)

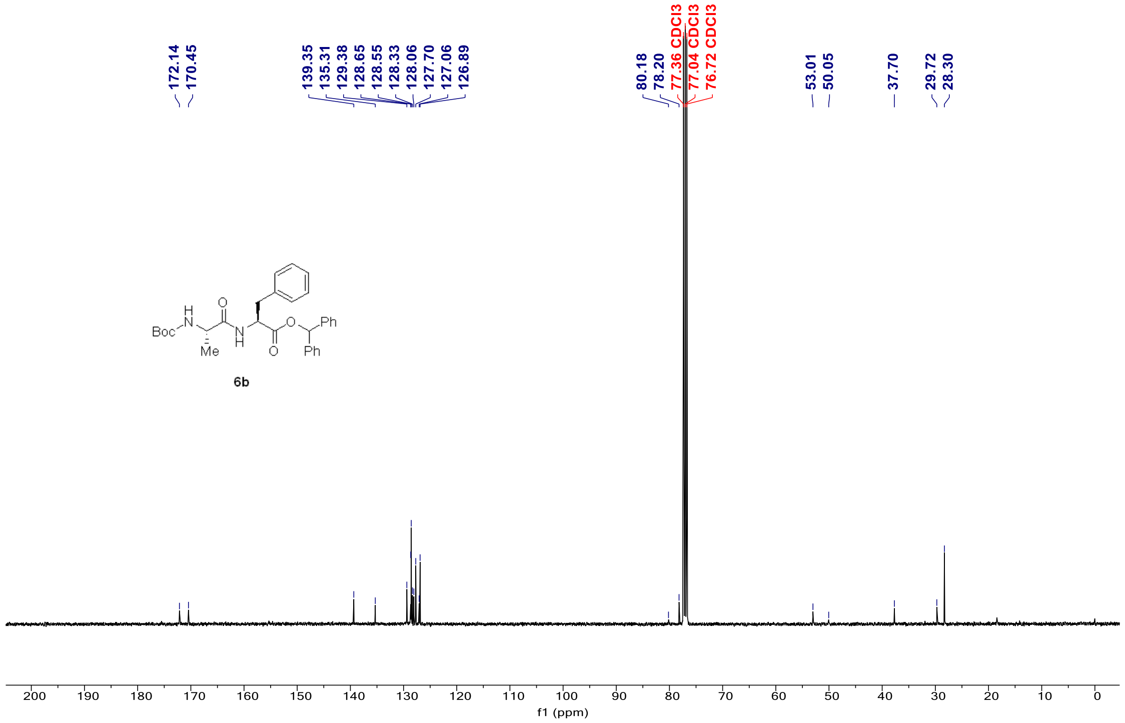

Figure S325. **^{13}C NMR** of the **6b** (101 MHz, CDCl3)

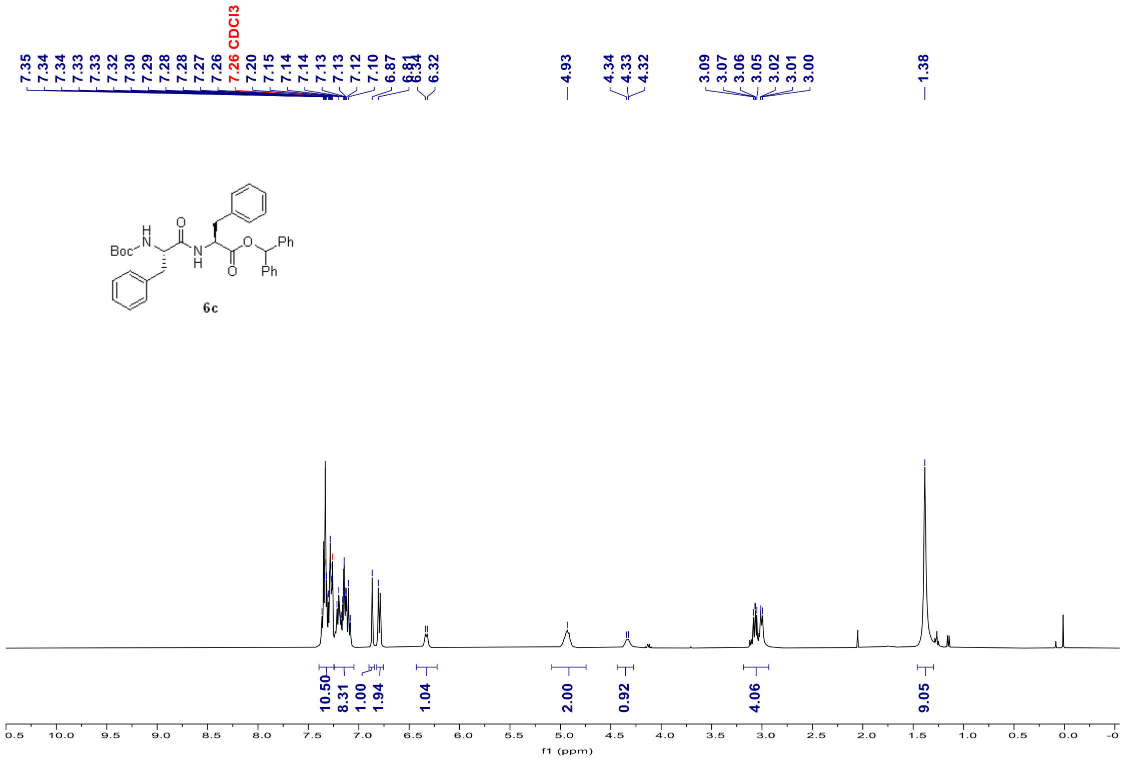

Figure S326. **^{1}H NMR** of the **6c** (400 MHz, CDCl$_3$**)**

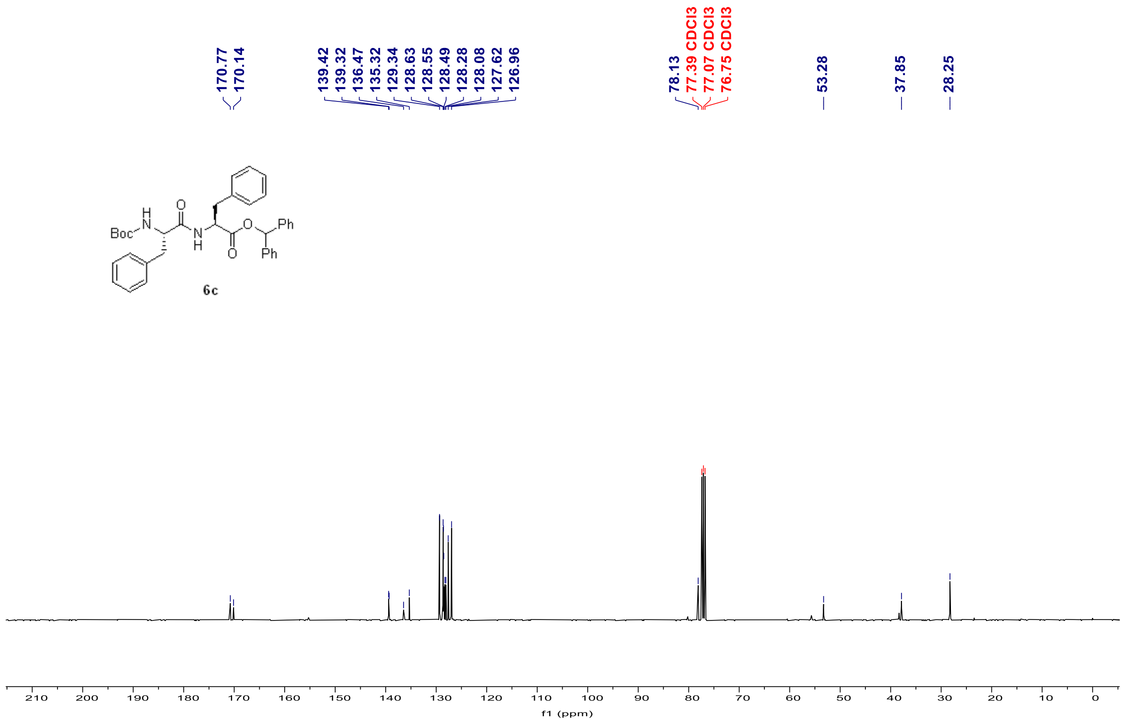

Figure S327. **^{13}C NMR** of the **6c** (101 MHz, CDCl$_3$**)**

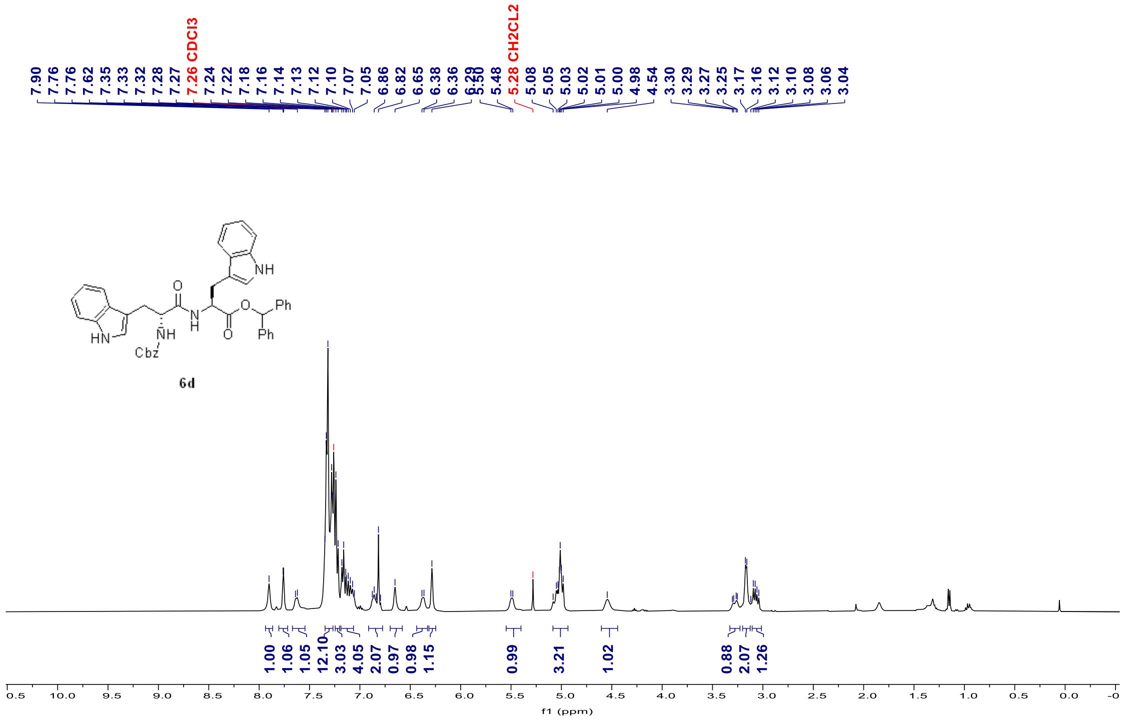

Figure S328. **^{1}H NMR** of the **6d** (400 MHz, $CDCl_3$)

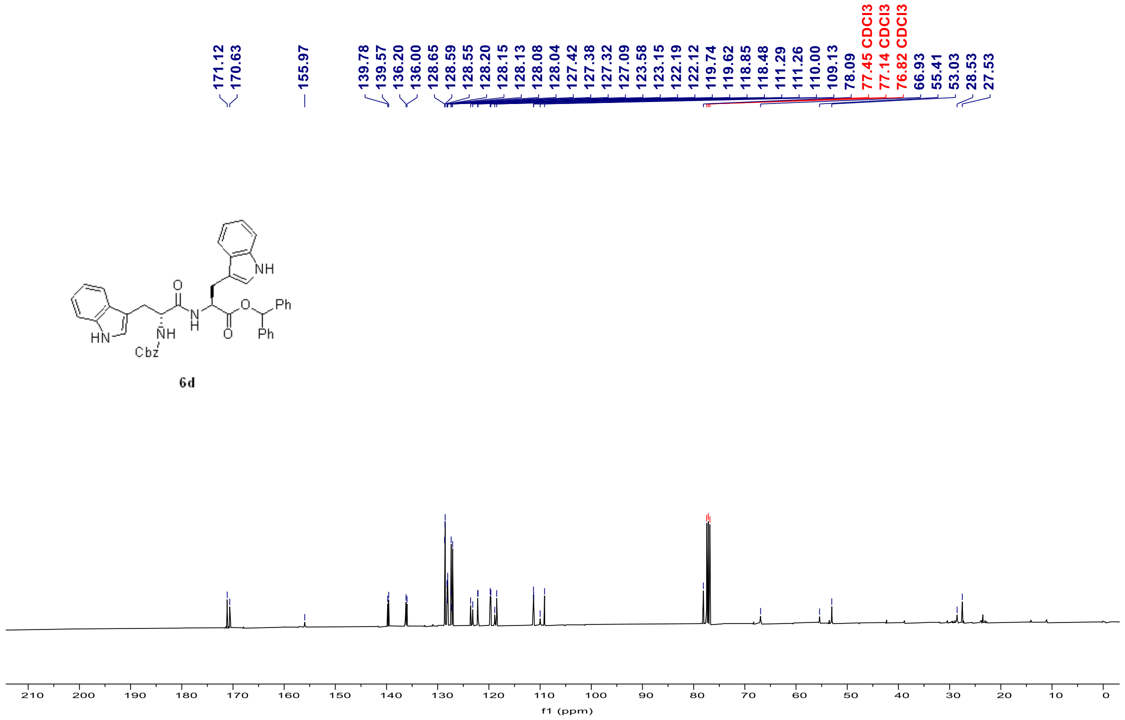

Figure S329. **^{13}C NMR** of the **6d** (101 MHz, $CDCl_3$)

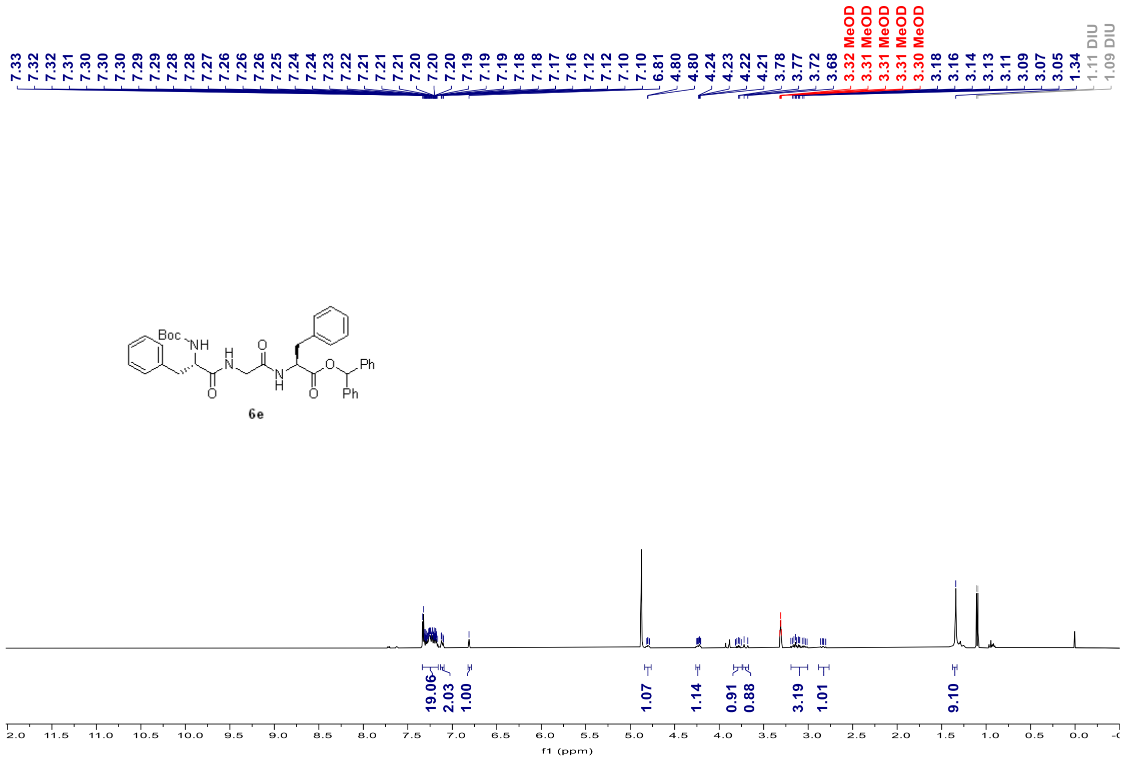

Figure S330. ^{1}**H NMR** of the **6e** (400 MHz, Methanol-d_4)

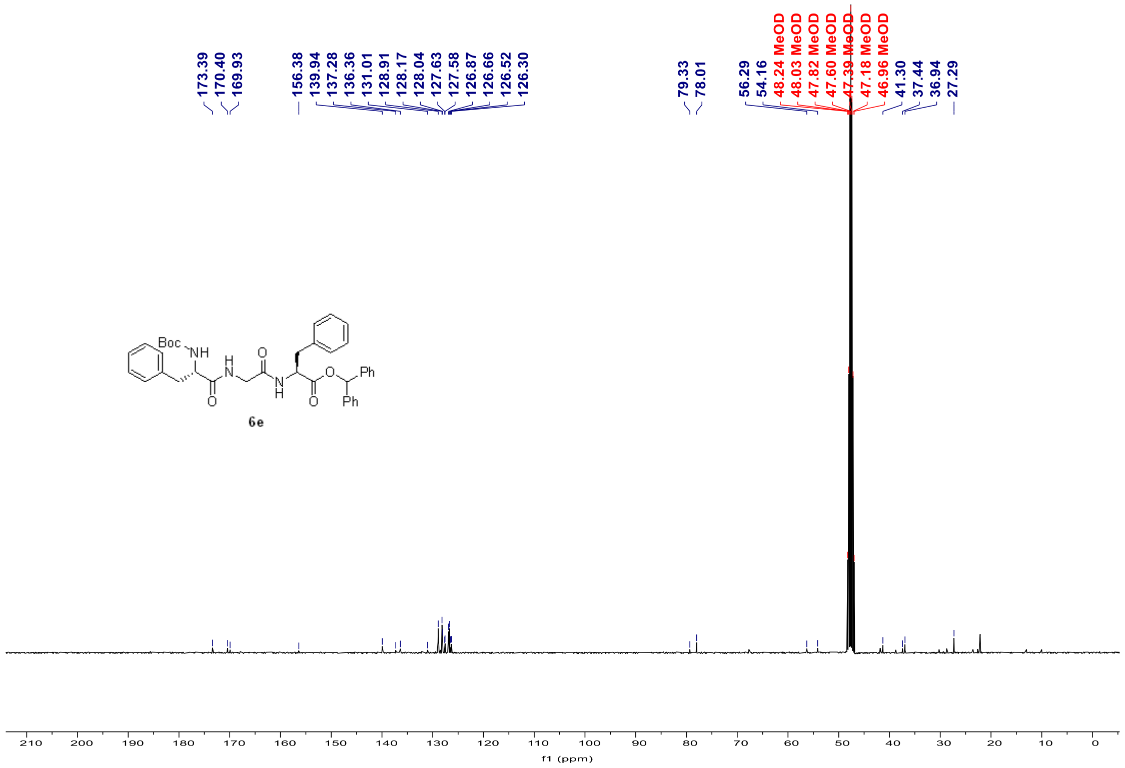

Figure S331. ^{13}**C NMR** of the **6e** (101 MHz, Methanol-d_4)

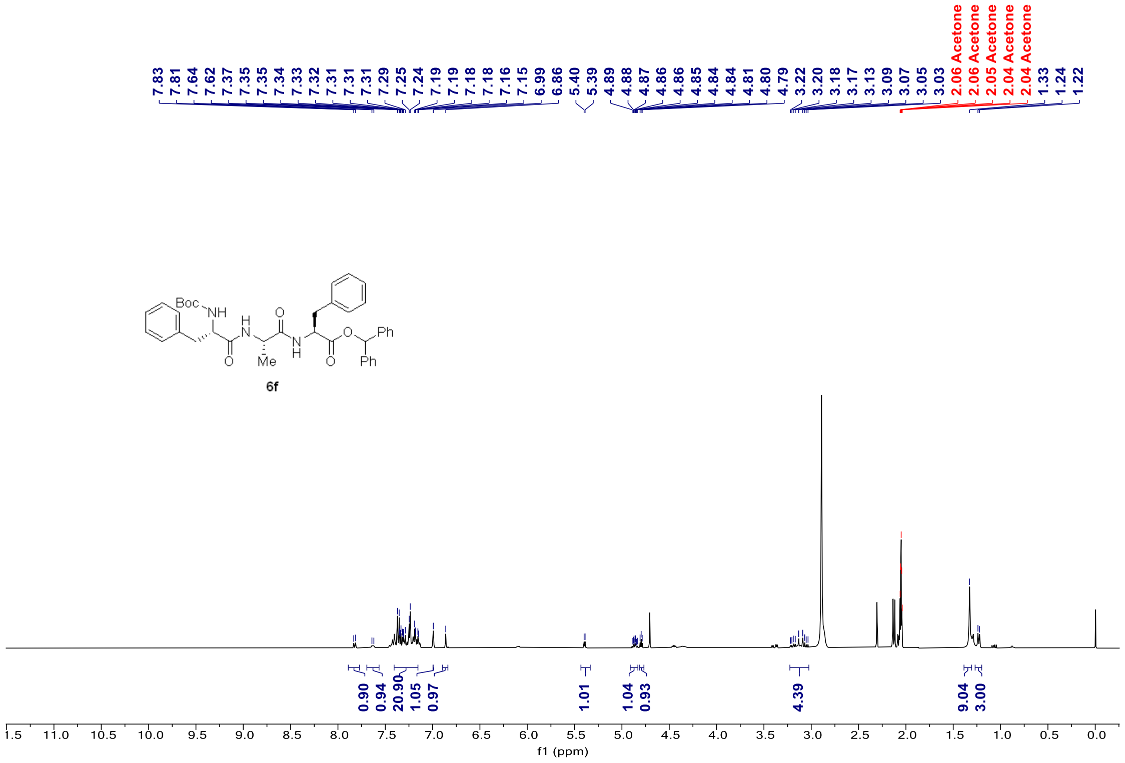

Figure S332. **^{1}H NMR** of the **6f** (400 MHz, Acetone-d_6)

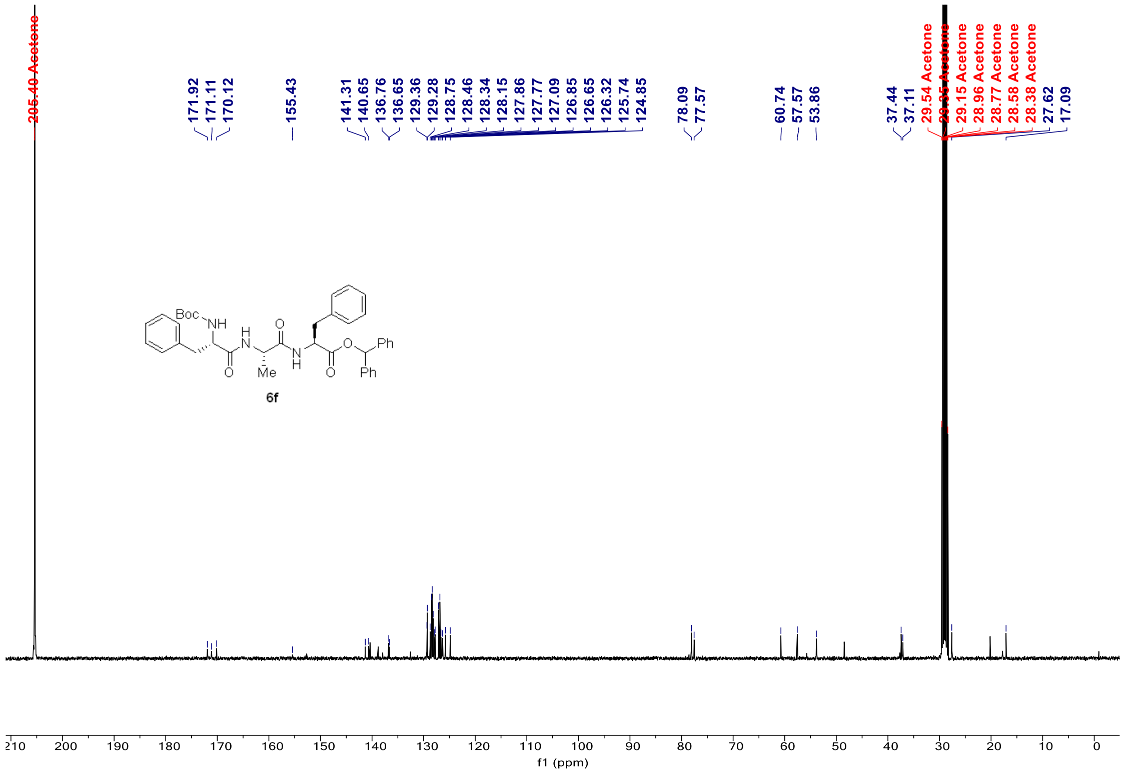

Figure S333. **^{13}C NMR** of the **6f** (101 MHz, Acetone-d_6)

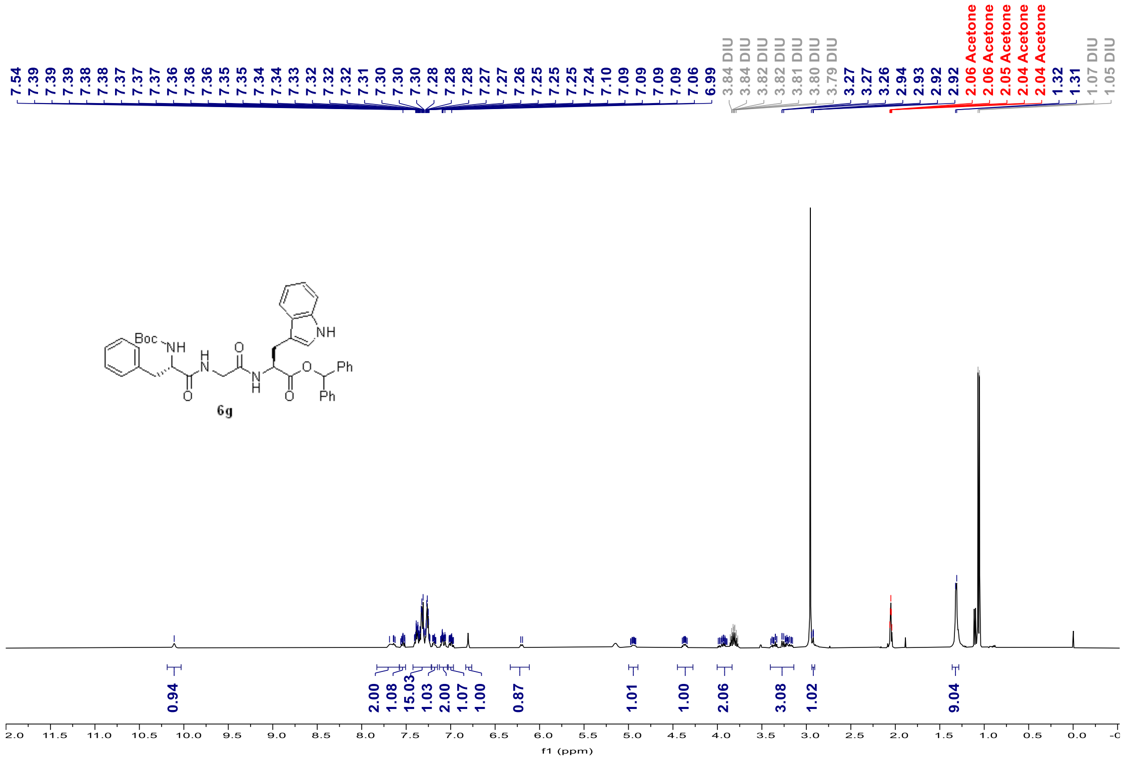

Figure S334 ^{1}H NMR of the **6g** (400 MHz, Acetone-d_6)

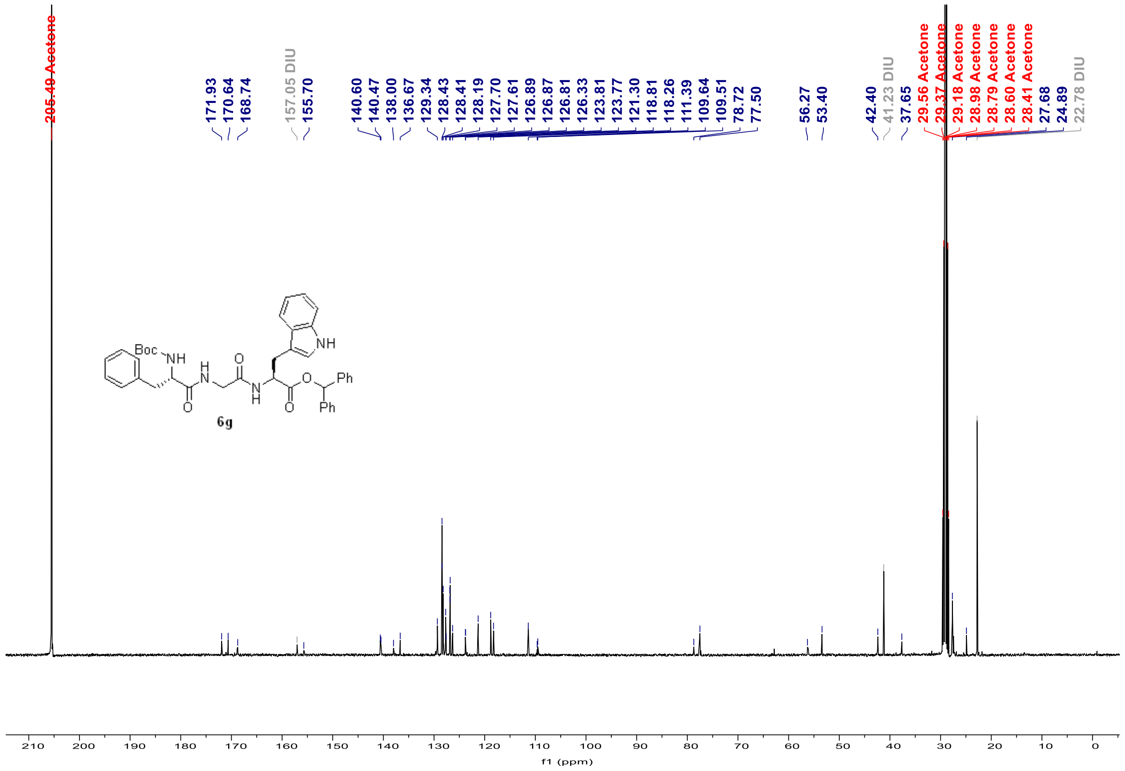

Figure S335. ^{13}C NMR of the **6g** (101 MHz, Acetone-d_6)

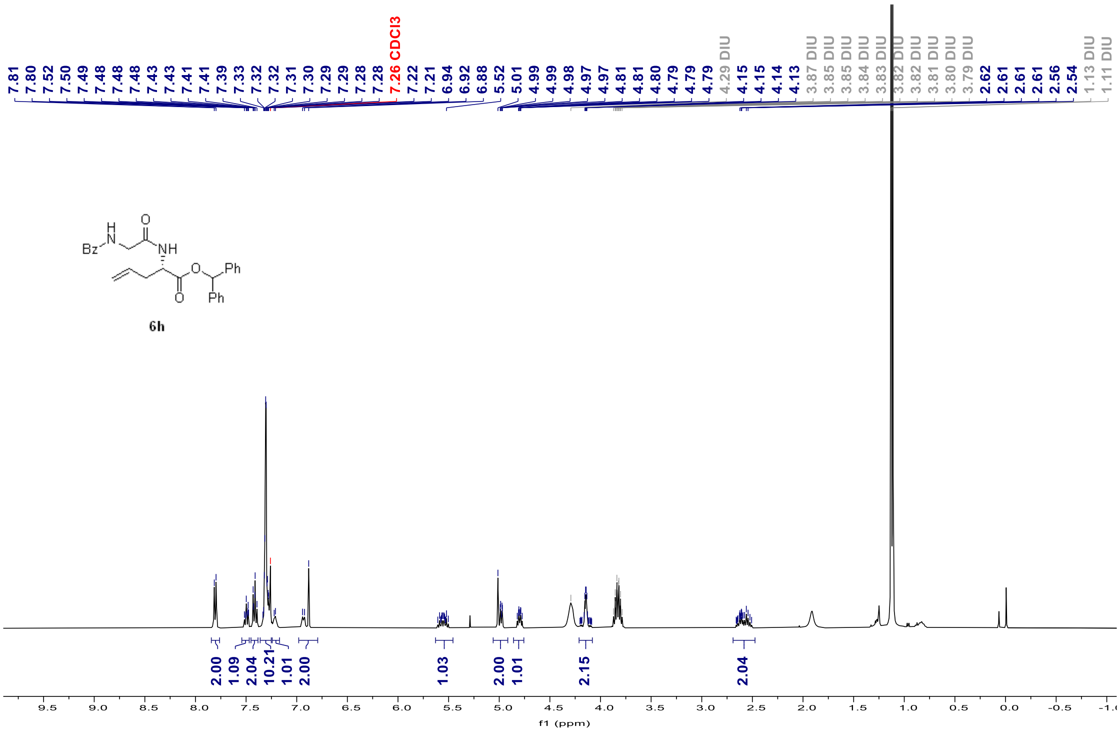

Figure S336. ^{1}H NMR of the **6h** (400 MHz, $CDCl_3$**)**

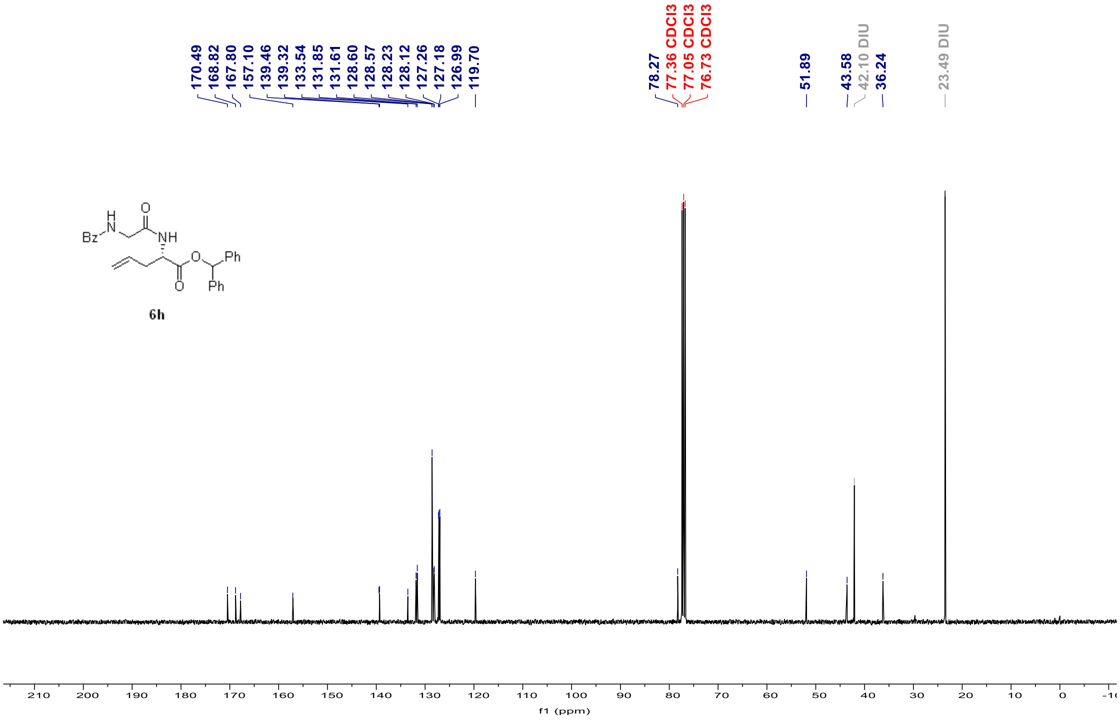

Figure S337. ^{13}C NMR of the **6h** (101 MHz, $CDCl_3$**)**

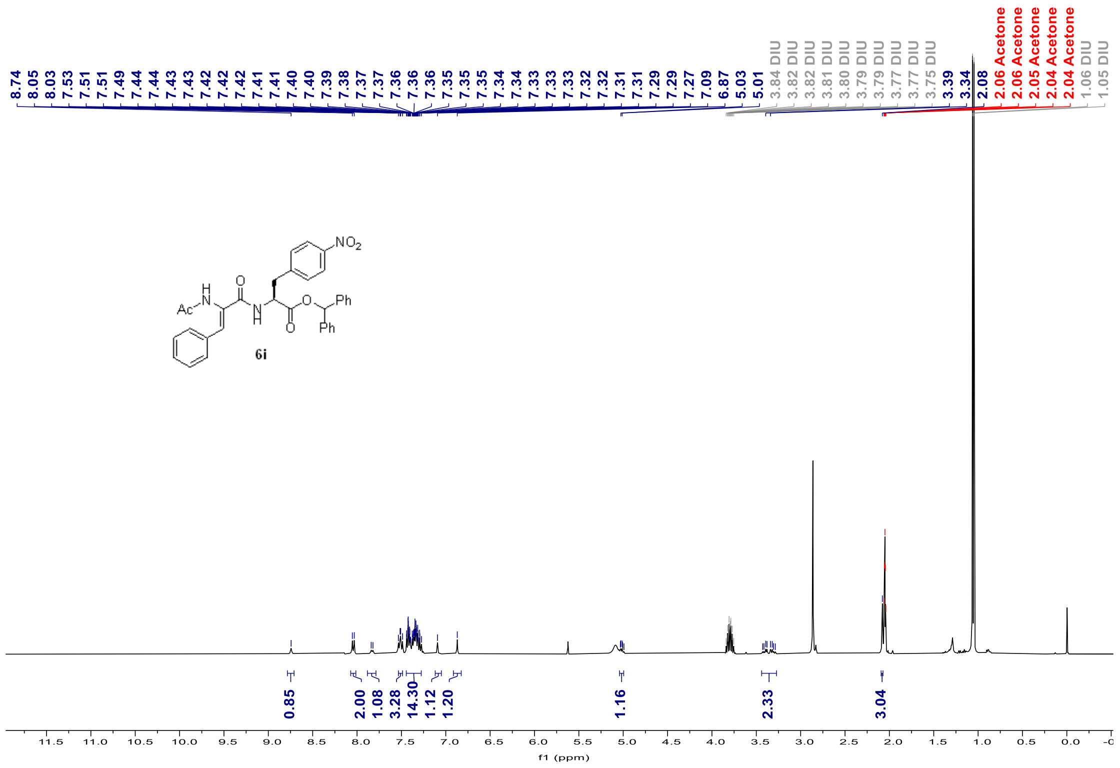

Figure S338. **^{1}H NMR** of the **6i** (400 MHz, Acetone-d_6)

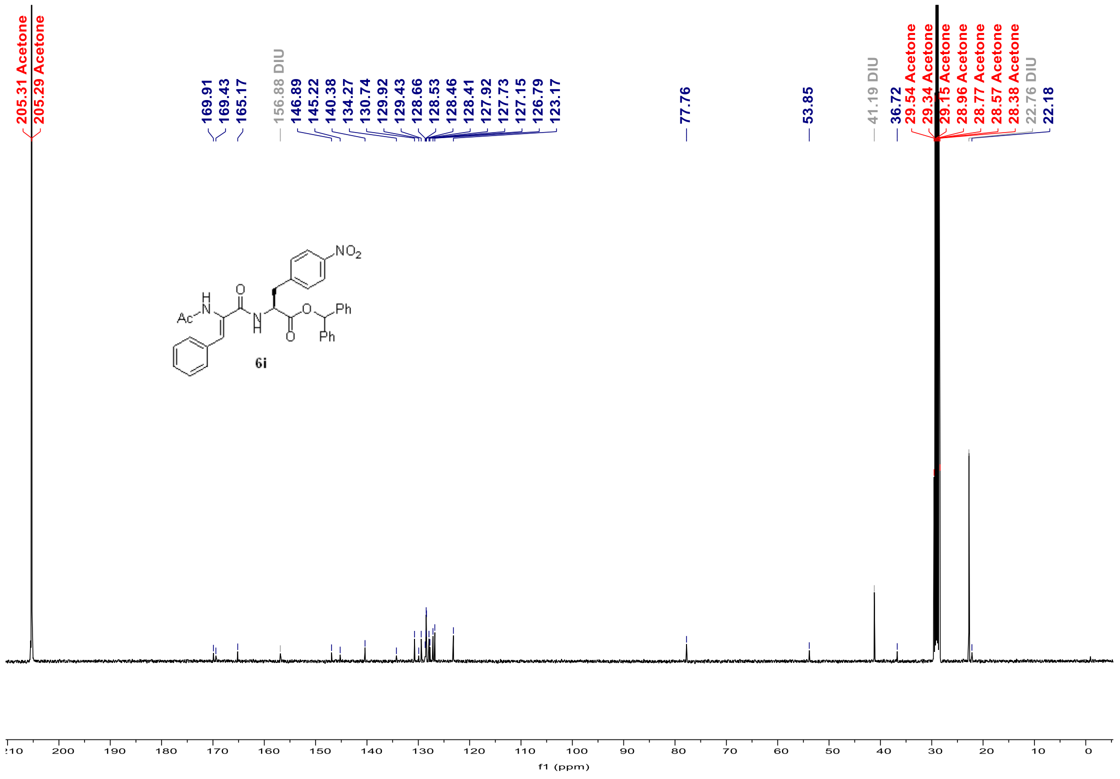

Figure S339. **^{13}C NMR** of the **6i** (101 MHz, Acetone-d_6)

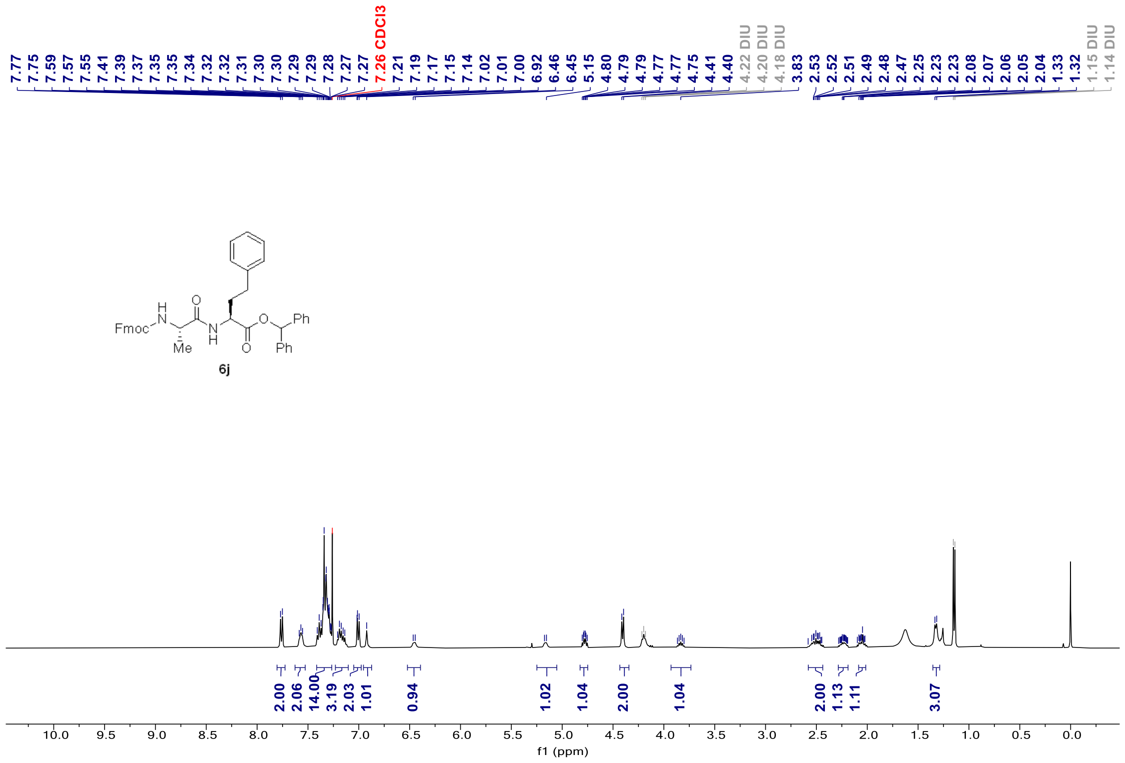

Figure S340. ^{1}H NMR of the 6j (400 MHz, $CDCl_3$)

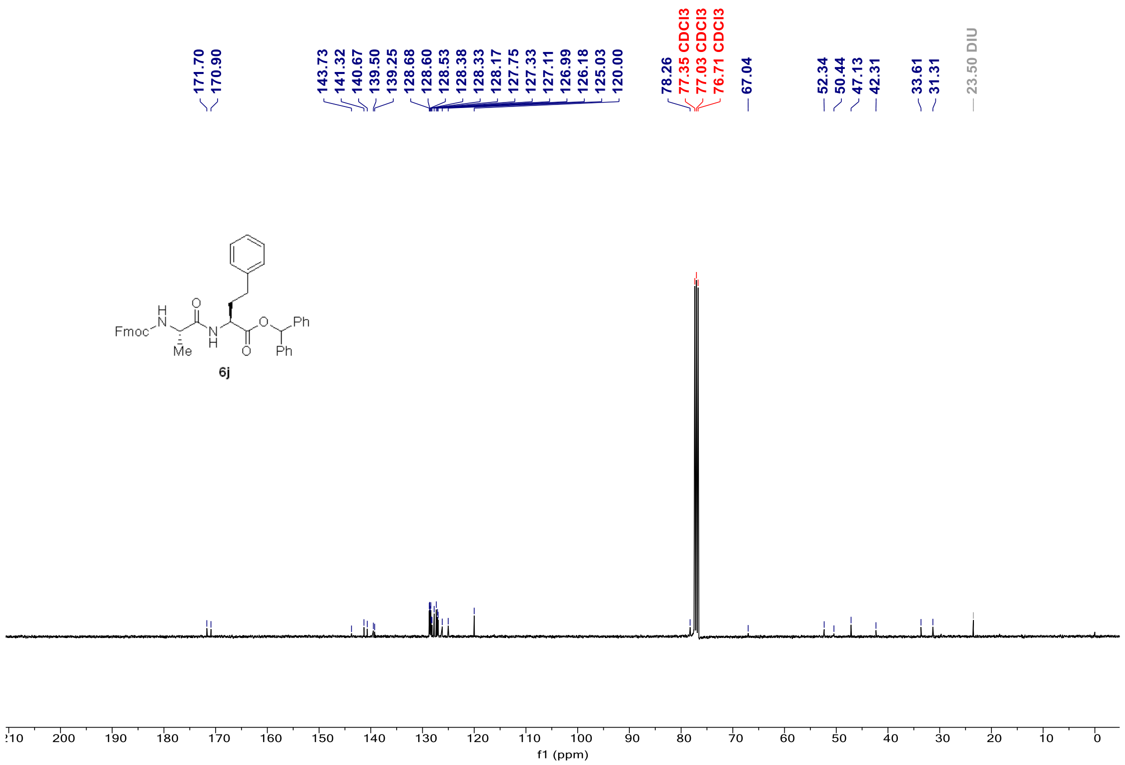

Figure S341. ^{13}C NMR of the 6j (101 MHz, $CDCl_3$)

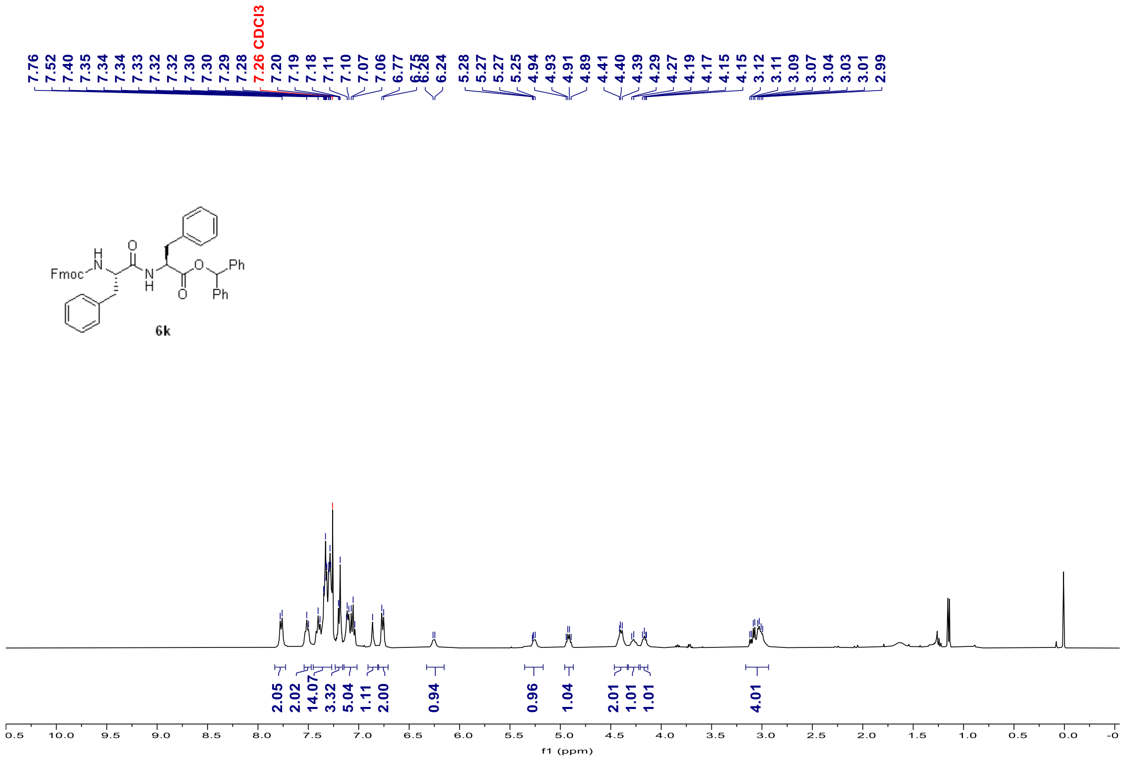

Figure S342. **^{1}H NMR** of the **6k** (400 MHz, $CDCl_3$)

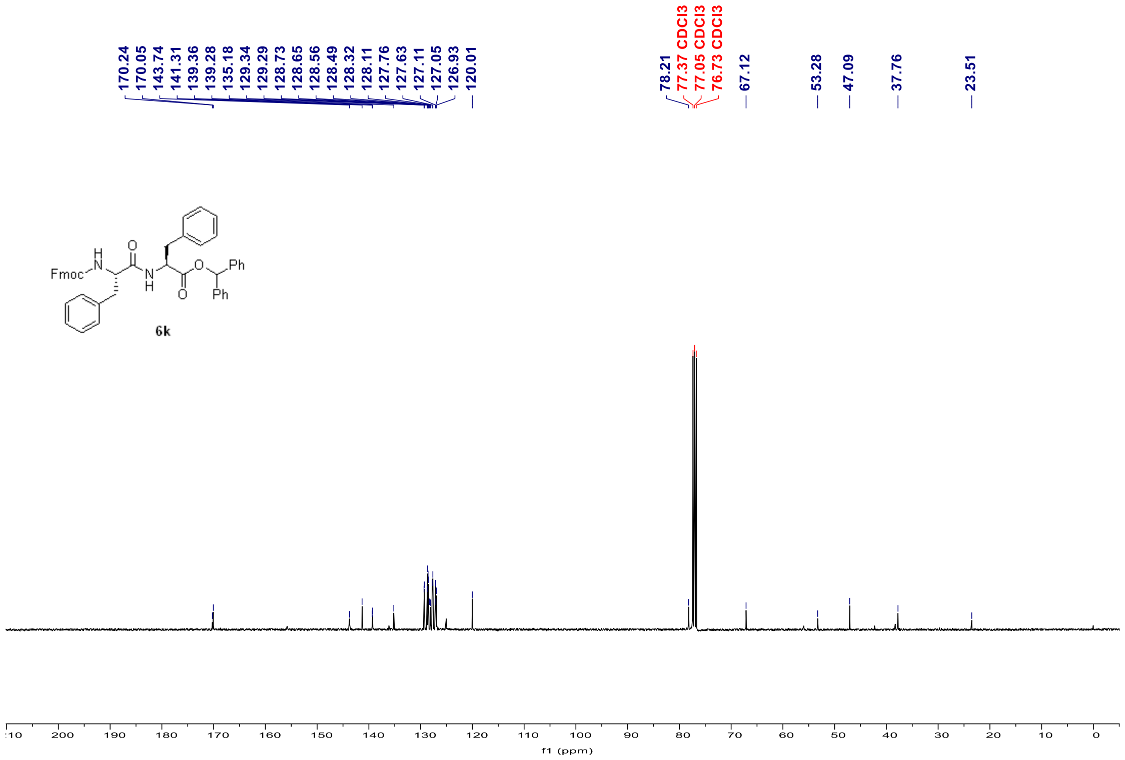

Figure S343. **^{13}C NMR** of the **6k** (101 MHz, $CDCl_3$)

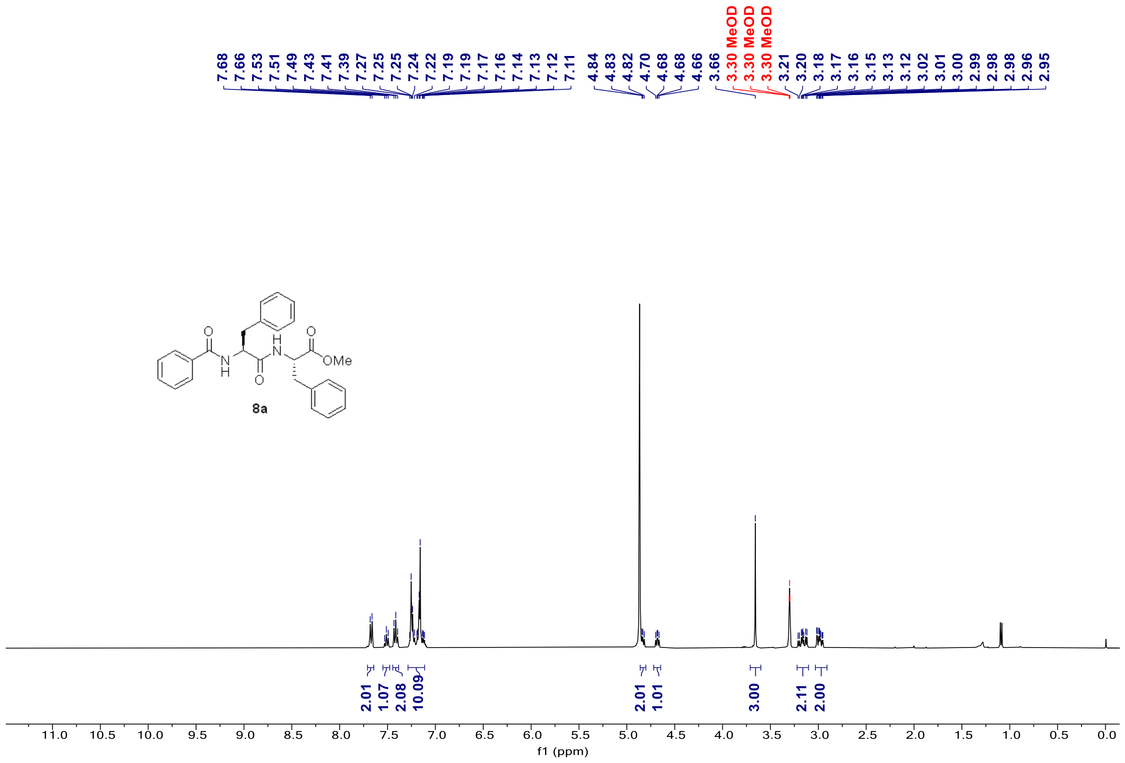

Figure S344. **^{1}H NMR** of the **8a** (400 MHz, Methanol-*d₄*)

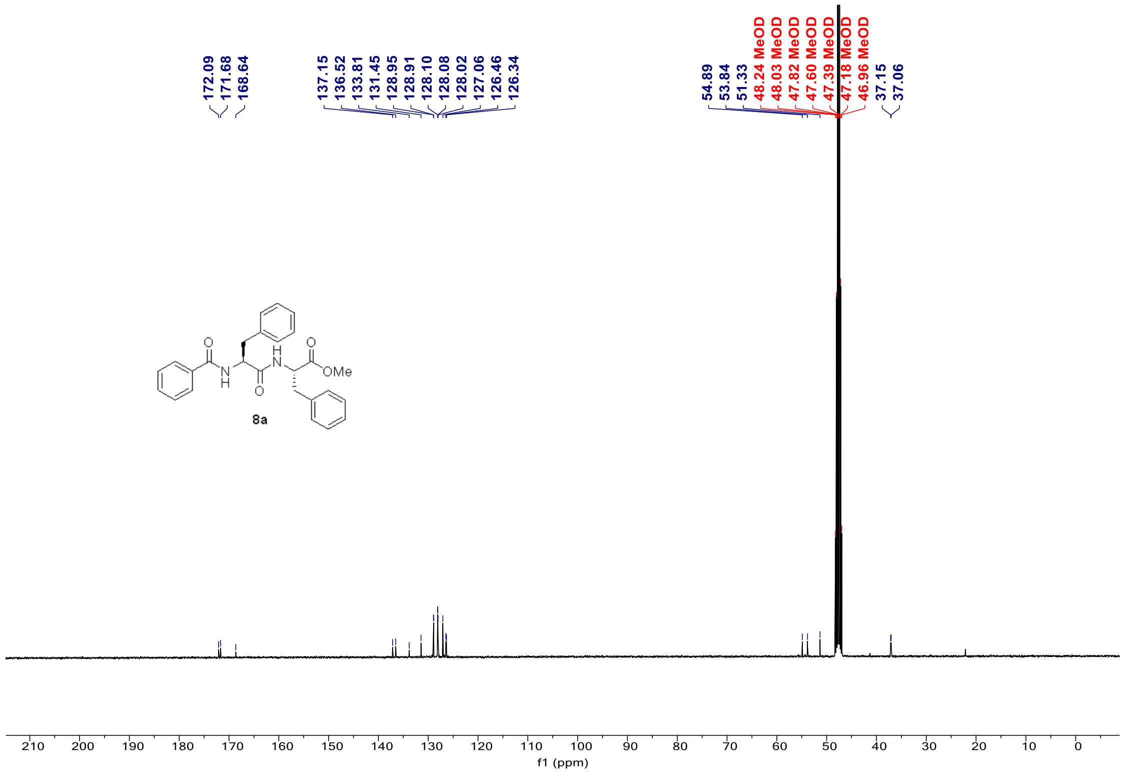

Figure S345. **^{13}C NMR** of the **8a** (101 MHz, Methanol-*d₄*)

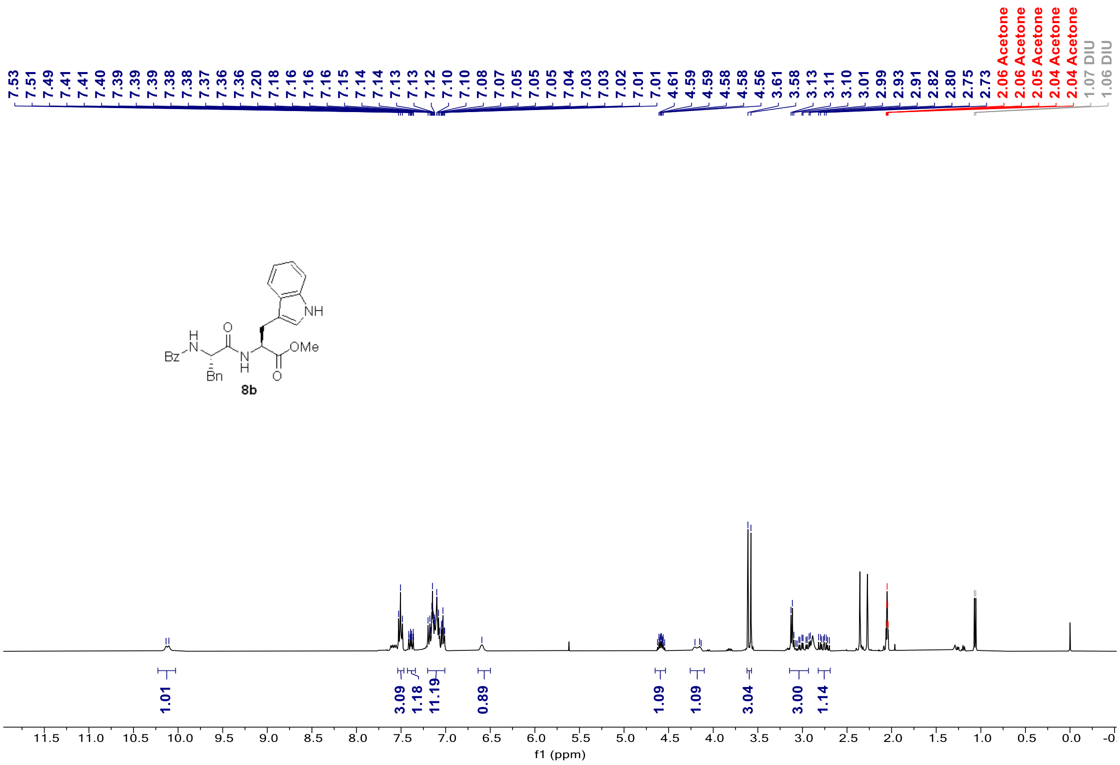

Figure S346. **^{1}H NMR** of the **8b** (400 MHz, Acetone-d_6)

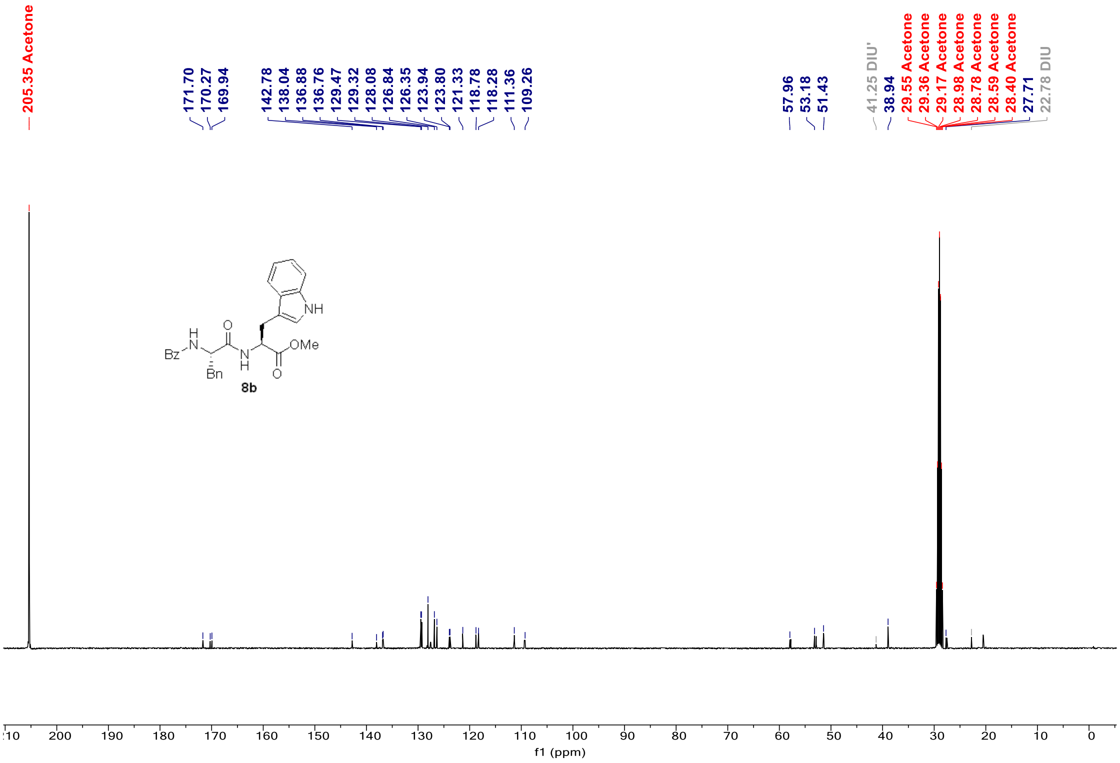

Figure S347. **^{13}C NMR** of the **8b** (101 MHz, Acetone-d_6)

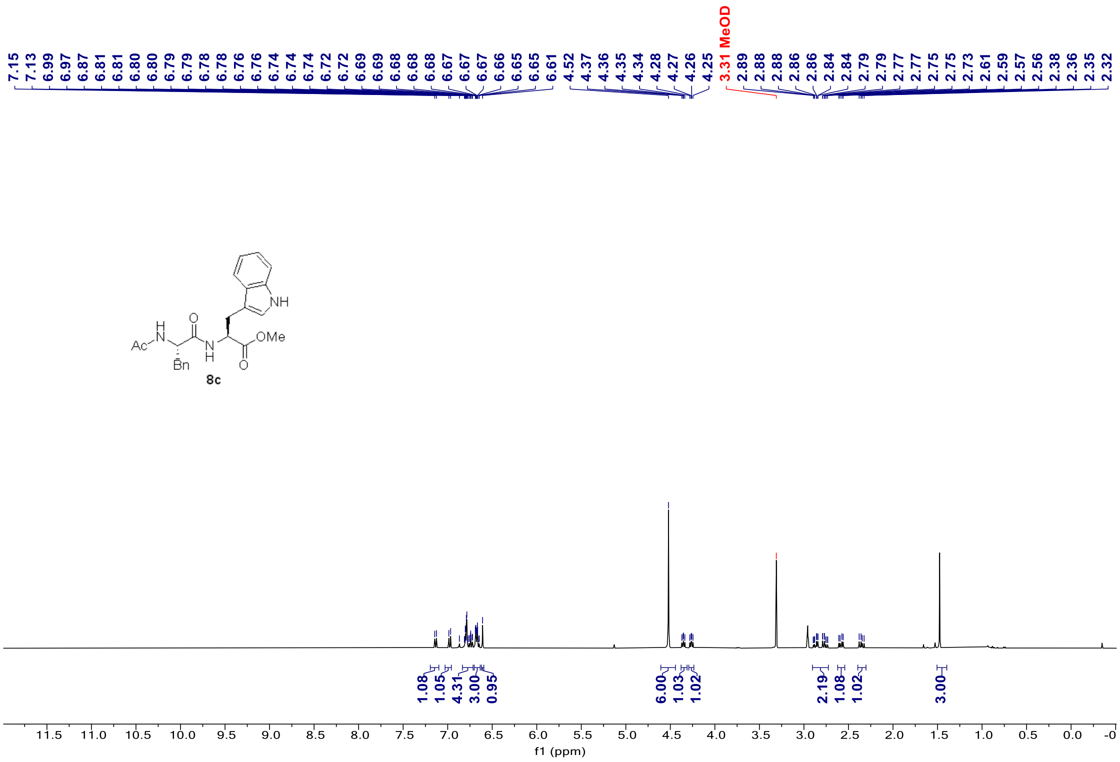

Figure S348. ^{1}**H NMR** of the **8c** (400 MHz, Methanol-*d*$_4$)

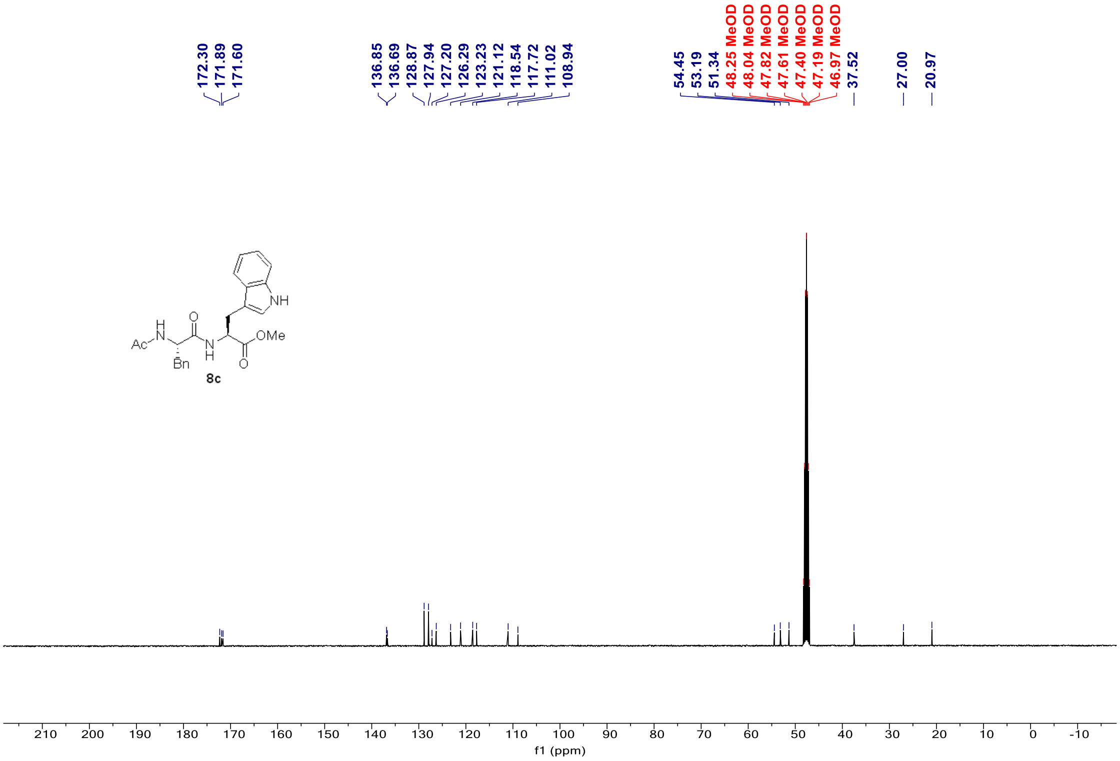

Figure S349. ^{13}**C NMR** of the **8c** (101 MHz, Methanol-*d*$_4$)

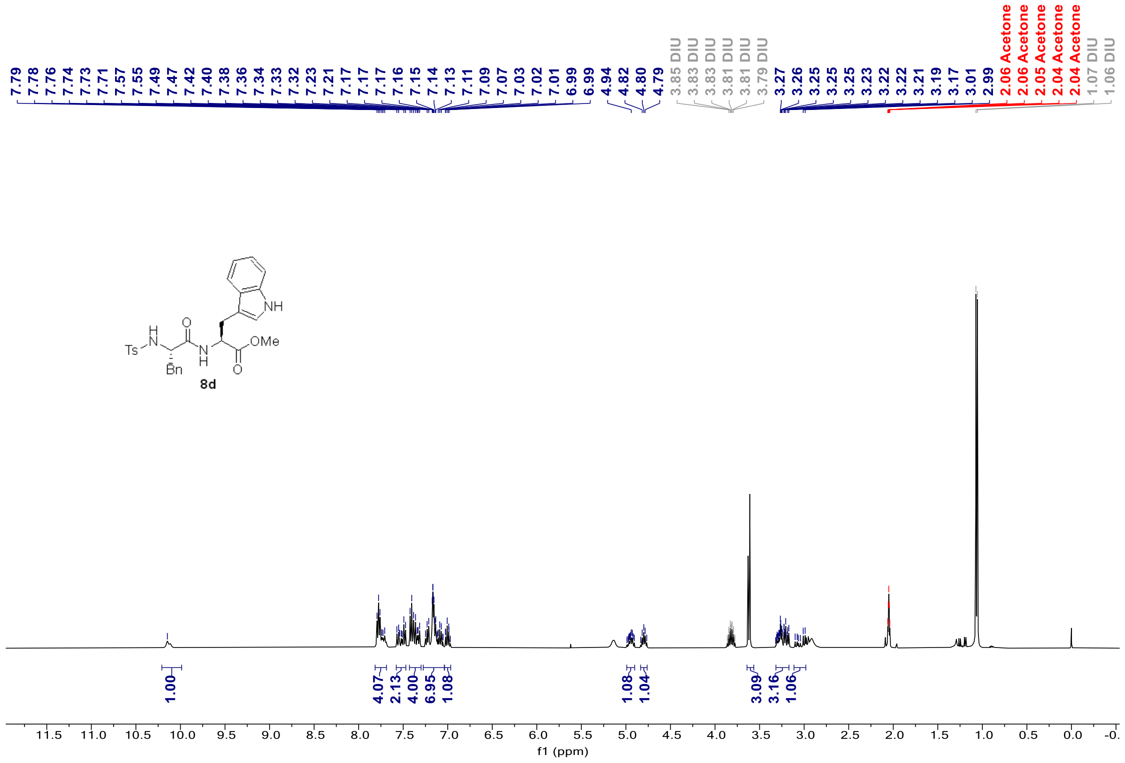

Figure S350. ^{1}**H NMR** of the **8d** (400 MHz, Acetone-d_6)

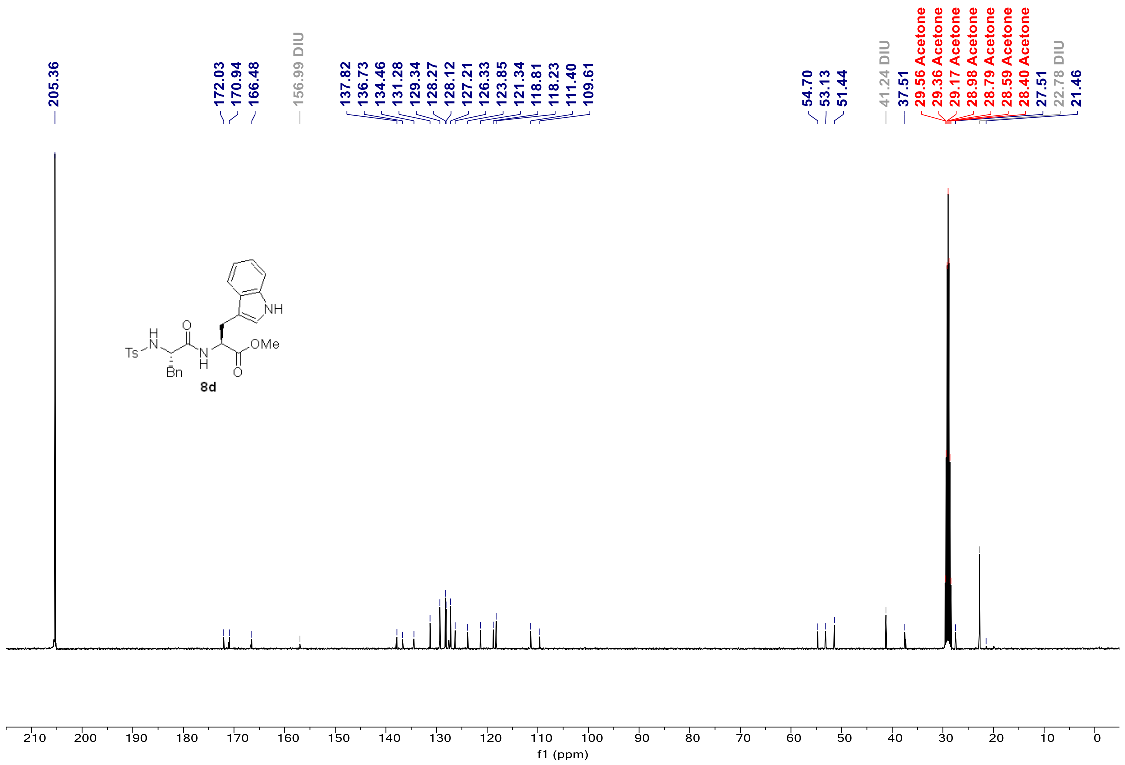

Figure S351. ^{13}**C NMR** of the **8d** (101 MHz, Acetone-d_6)

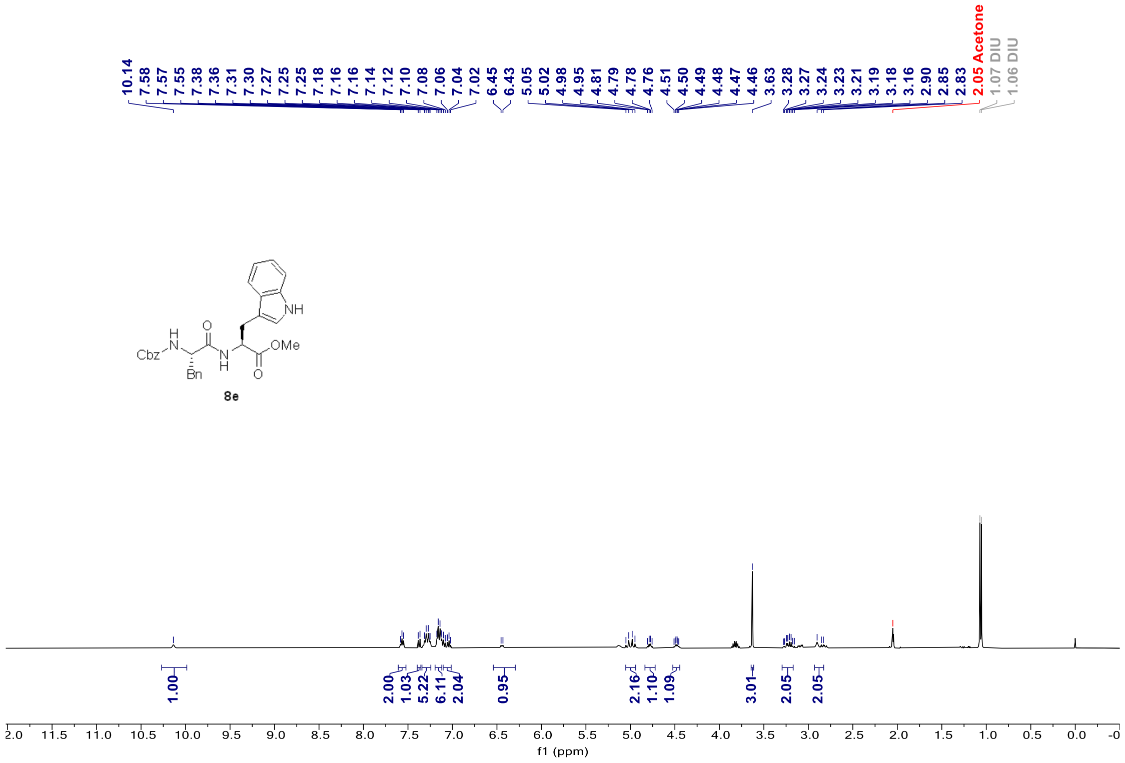

Figure S352. ^{1}**H NMR** of the **8e** (400 MHz, Acetone-d_6)

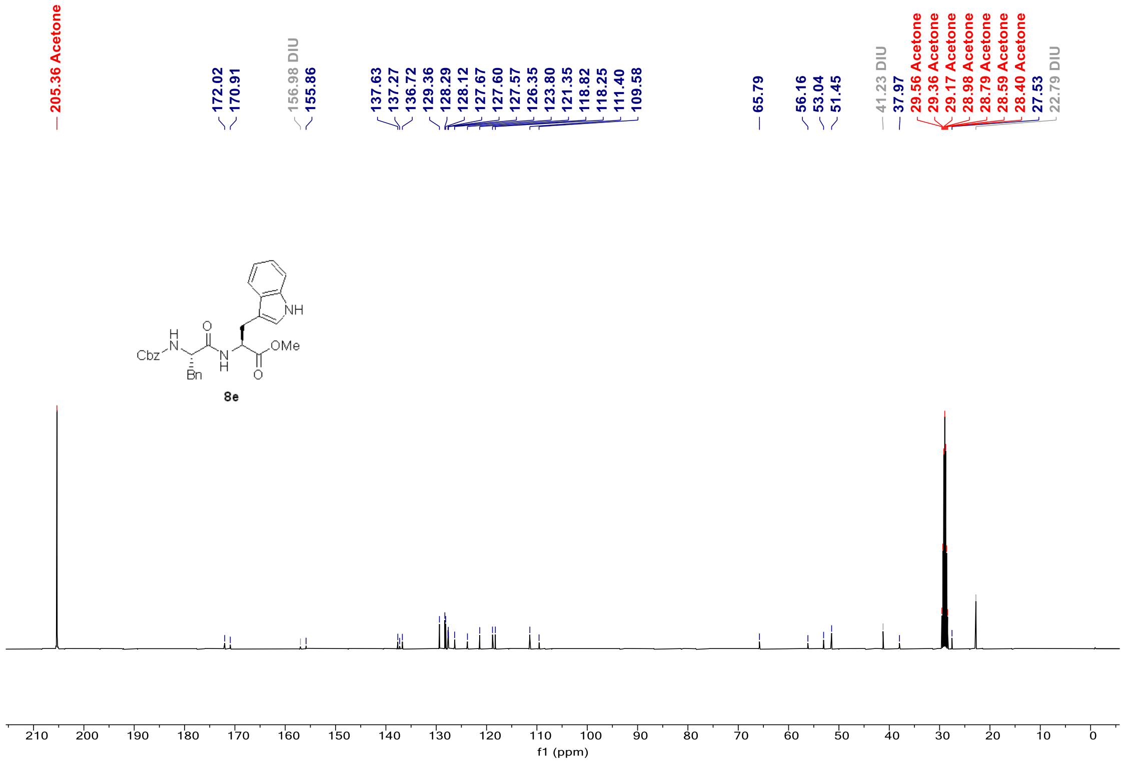

Figure S353. ^{13}**C NMR** of the **8e** (101 MHz, Acetone-d_6)

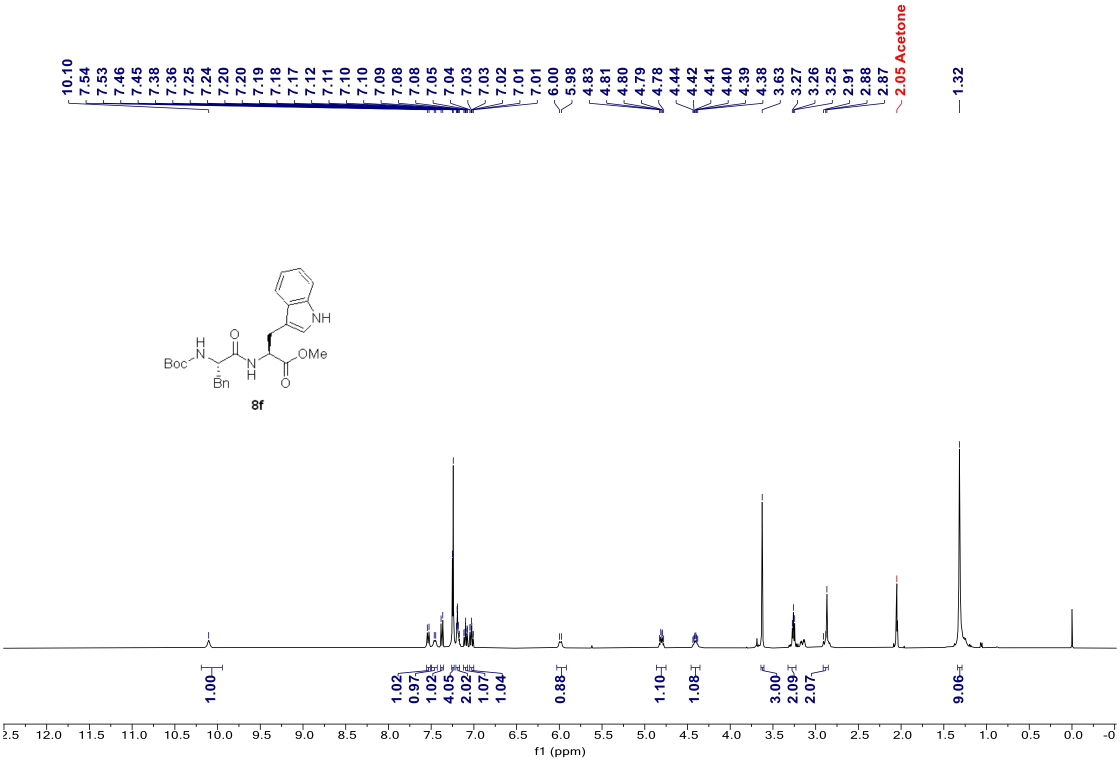

Figure S354. **^{1}H NMR** of the **8f** (400 MHz, Acetone-*d*$_6$)

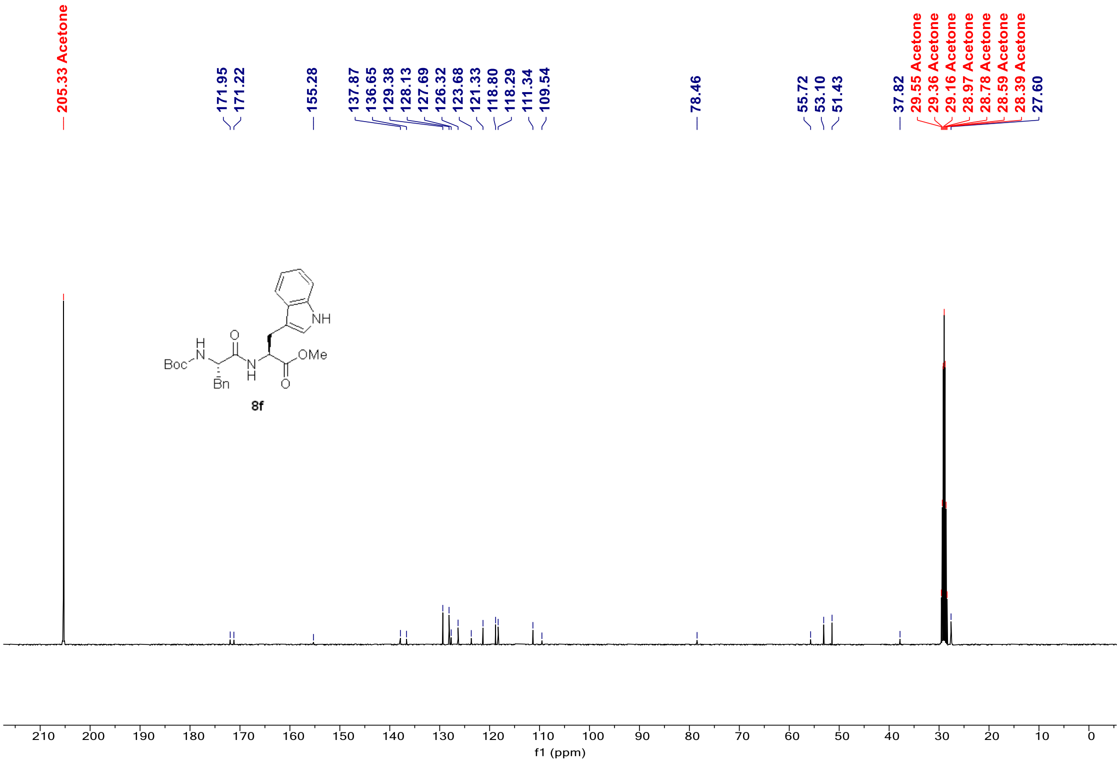

Figure S355. **^{13}C NMR** of the **8f** (101 MHz, Acetone-*d*$_6$)

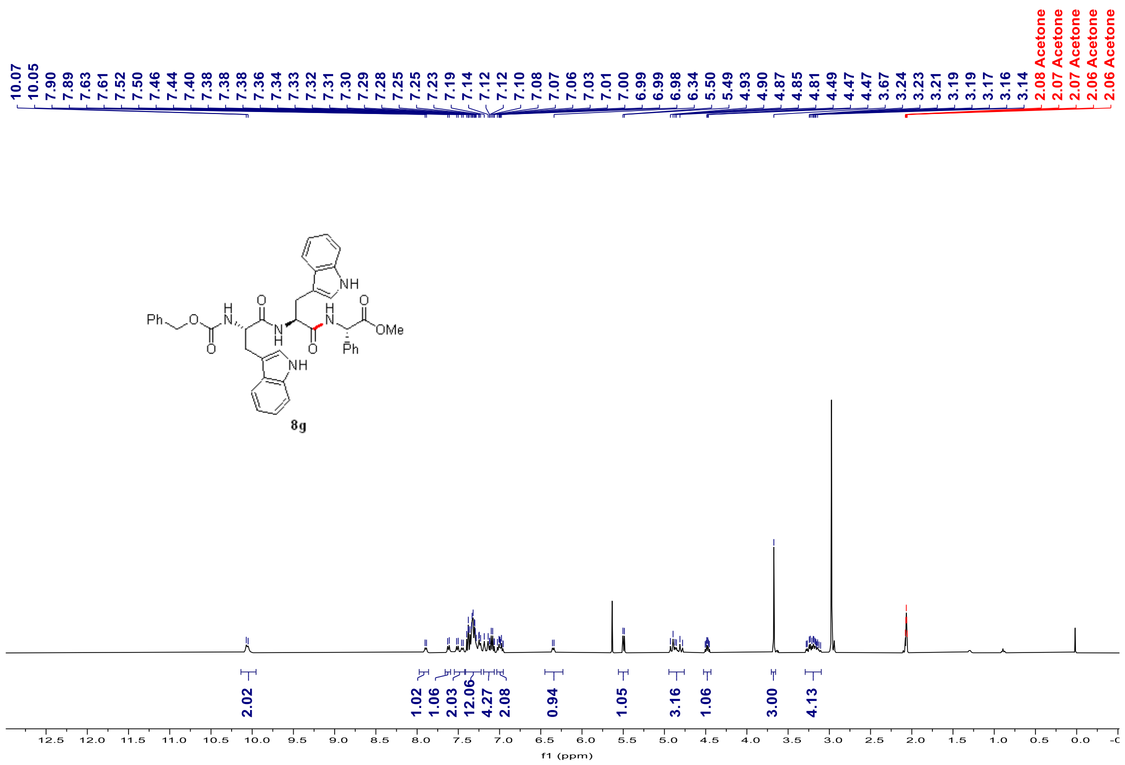

Figure S356. ^{1}H NMR of the **8g** (400 MHz, Acetone-*d6*)

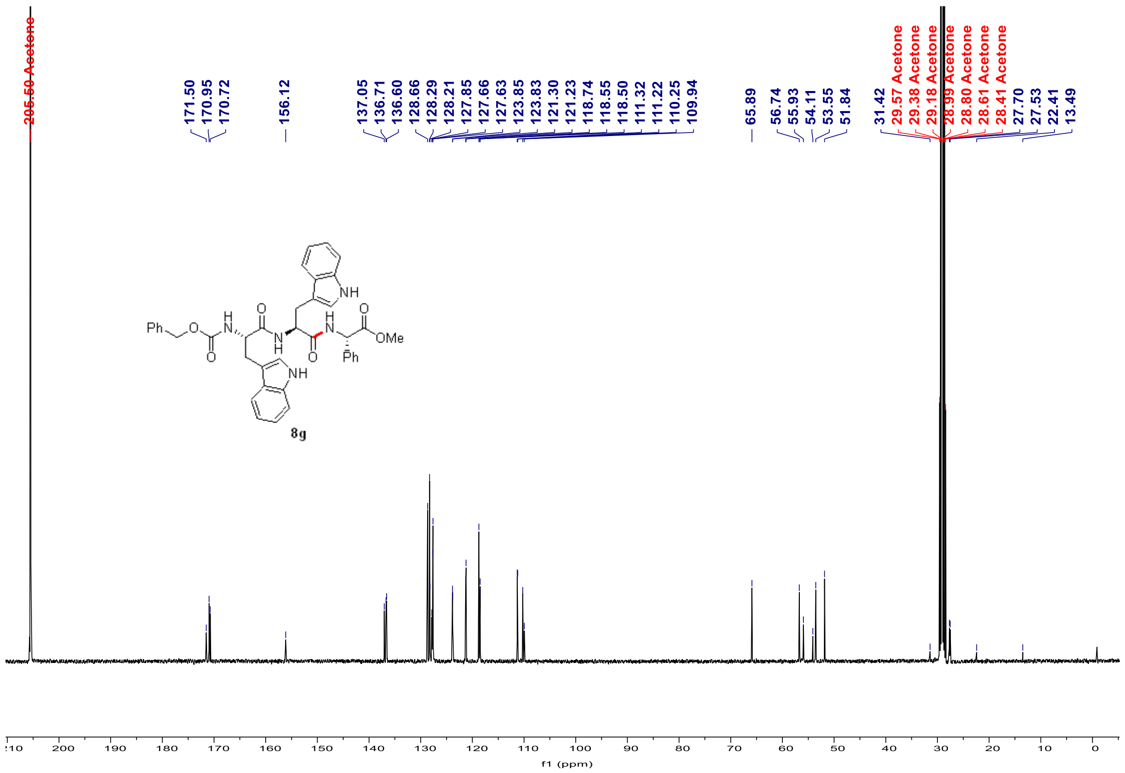

Figure S357. ^{13}C NMR of the **8g** (101 MHz, Acetone-*d6*)

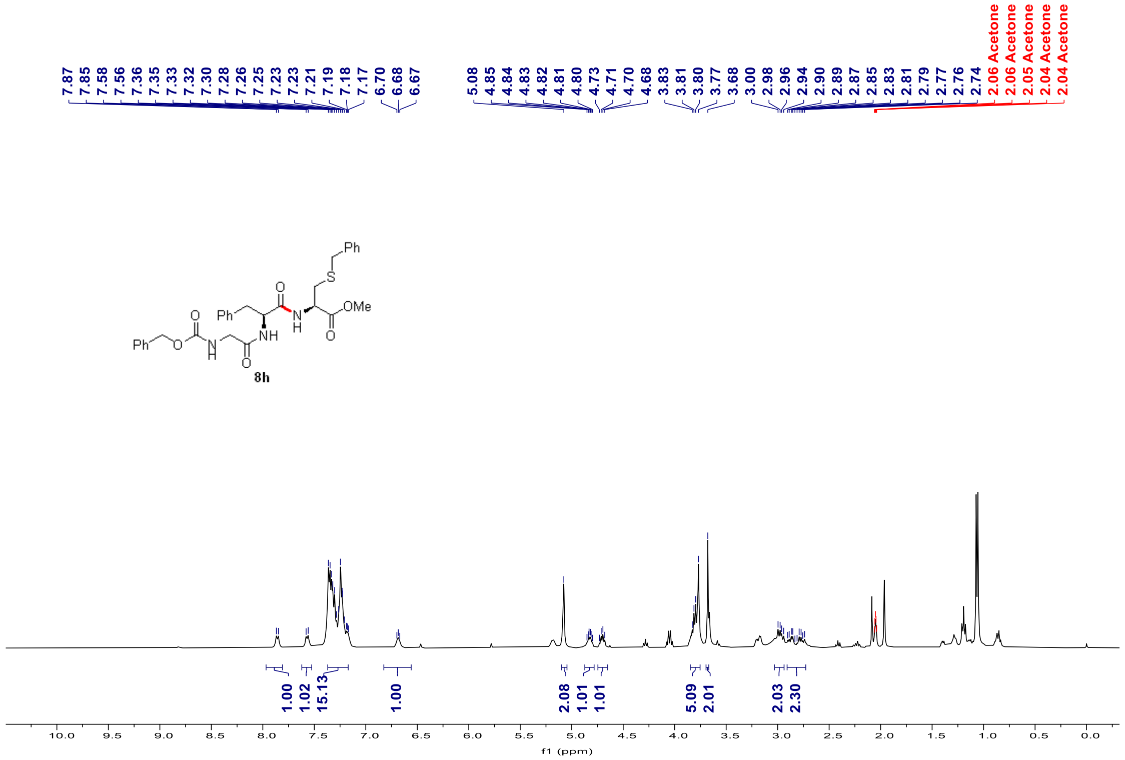

Figure S358. ^{1}**H NMR** of the **8h** (400 MHz, Acetone-*d6*)

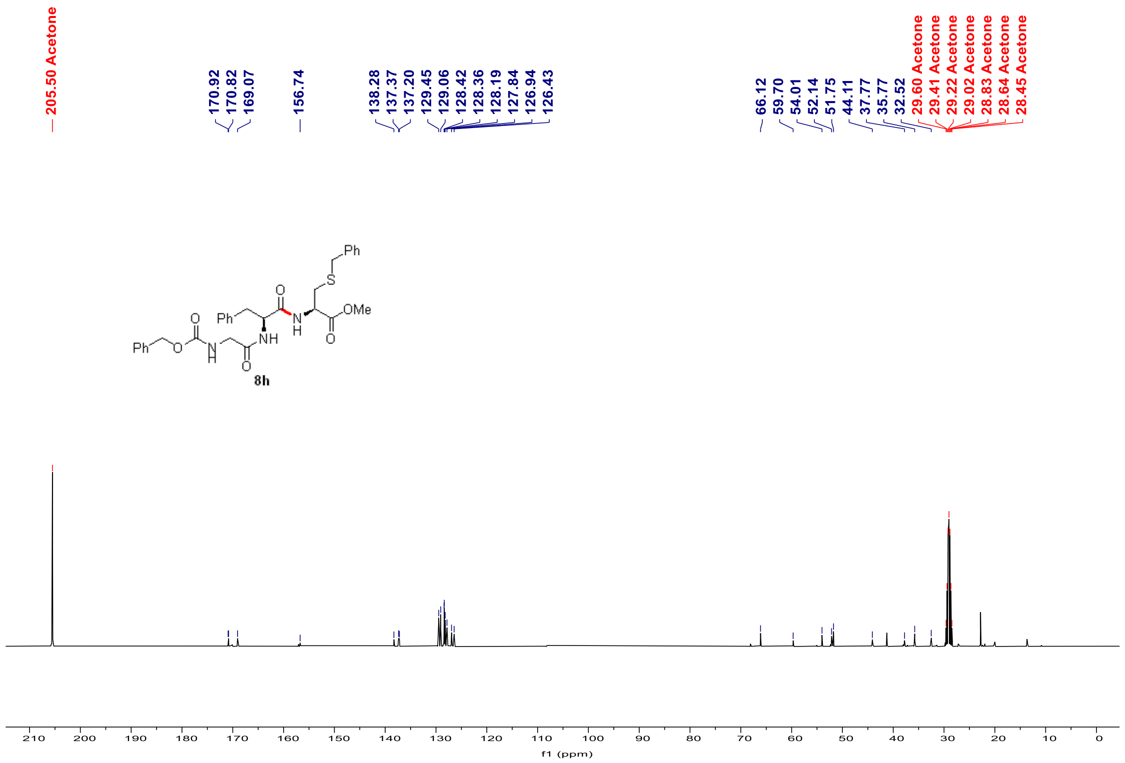

Figure S359. ^{13}**C NMR** of the **8h** (101 MHz, Acetone-*d6*)

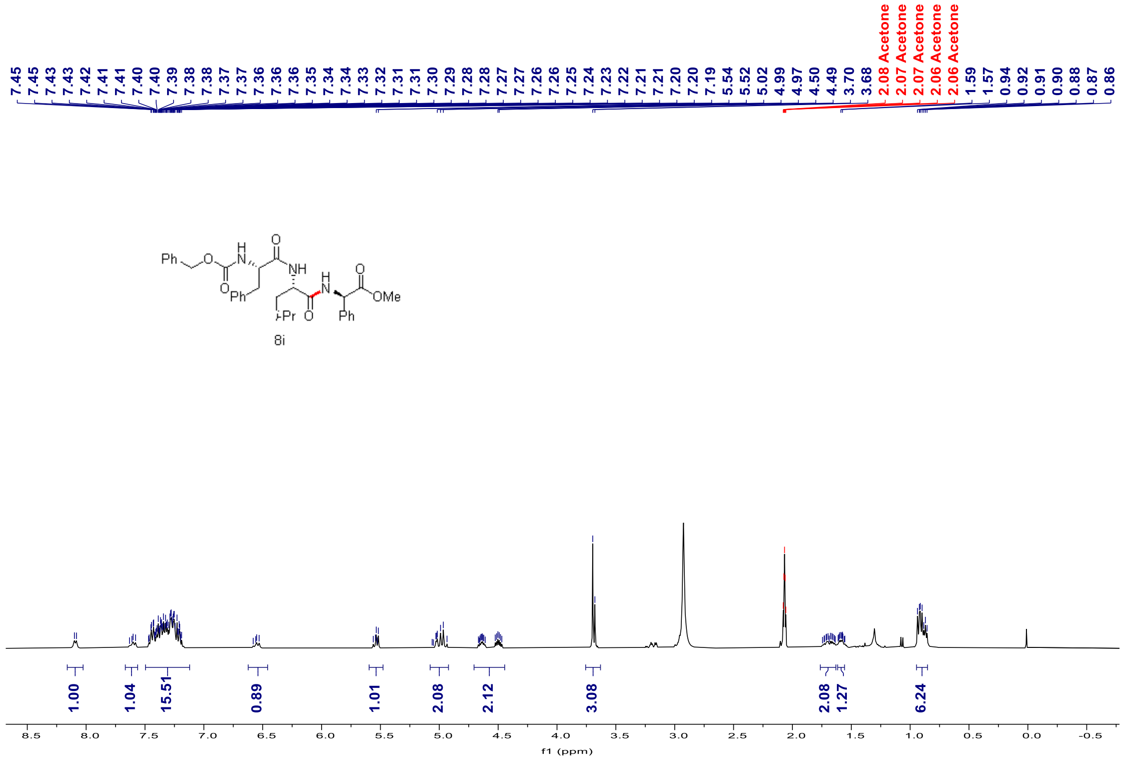

Figure S360. **^{1}H NMR** of the **8i** (400 MHz, Acetone-d_6)

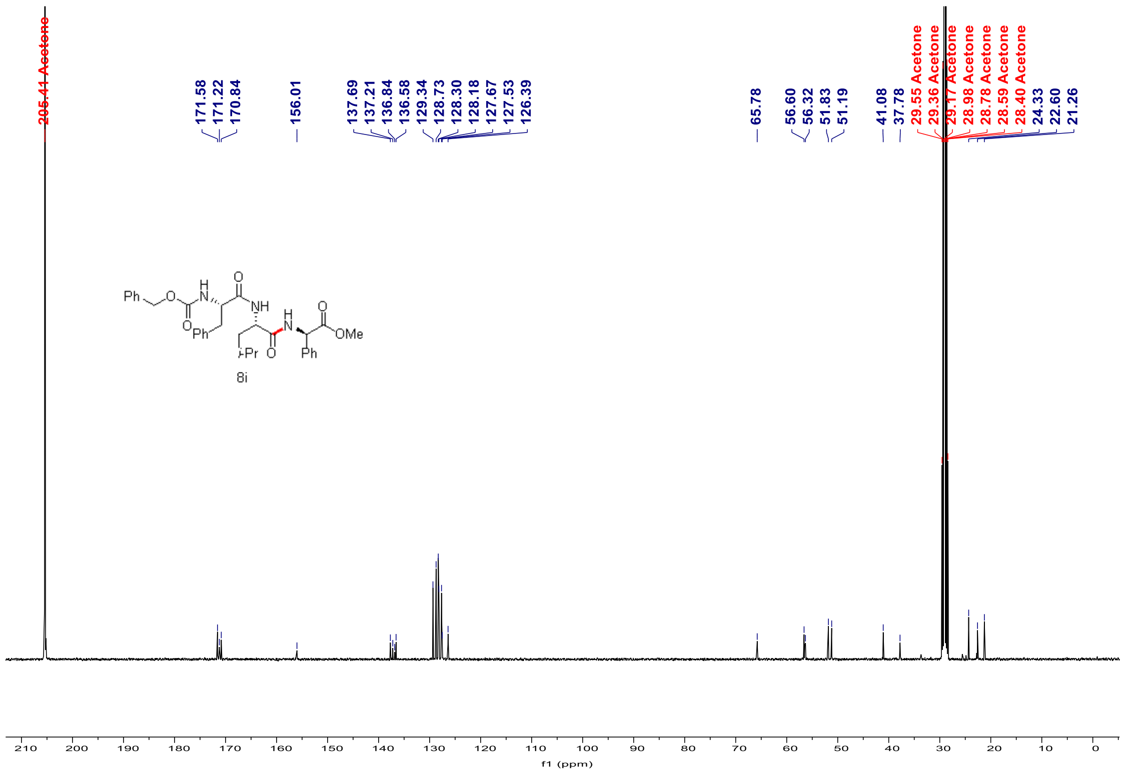

Figure S361. **^{13}C NMR** of the **8i** (101 MHz, Acetone-d_6)

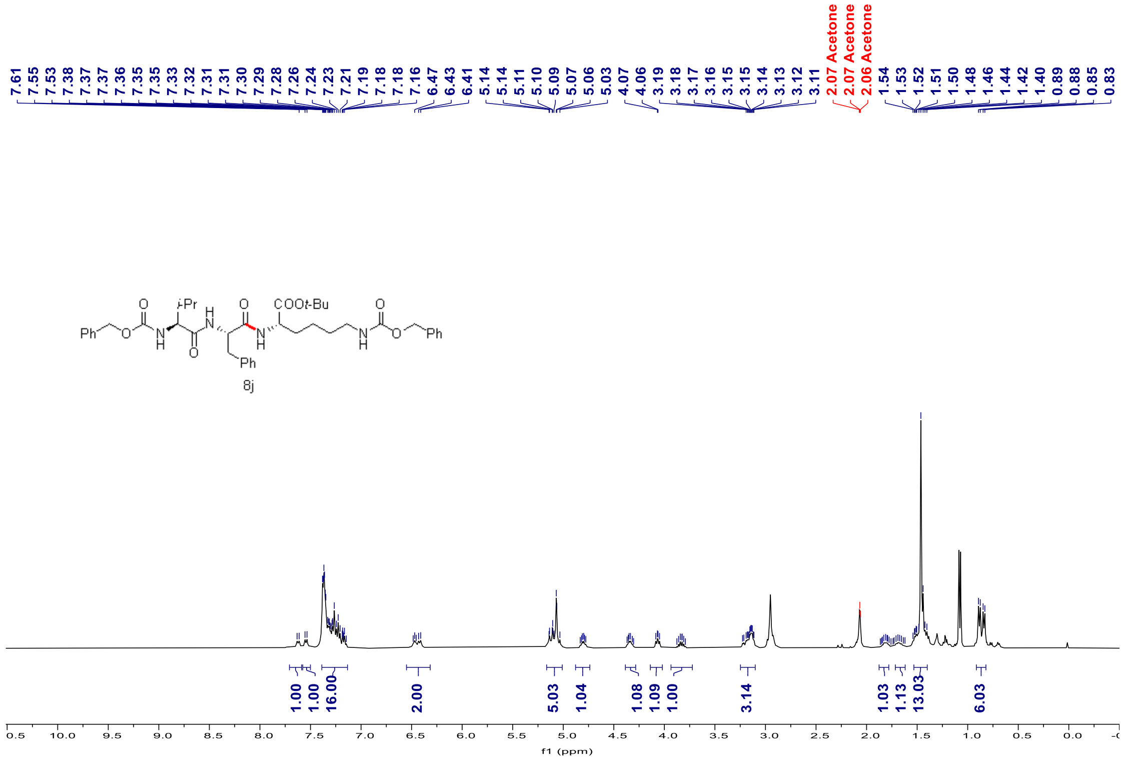

Figure S362. ^{1}**H NMR** of the **8j** (400 MHz, Acetone-d_6)

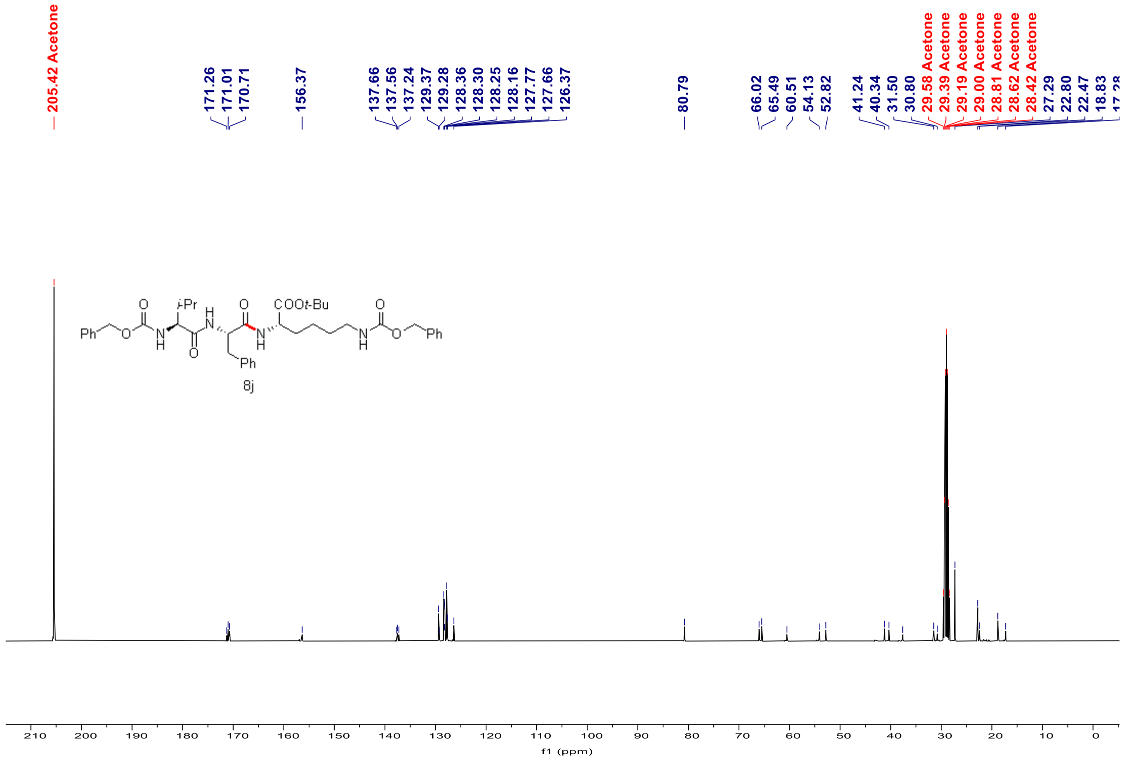

Figure S363. ^{13}**C NMR** of the **8j** (101 MHz, Acetone-d_6)

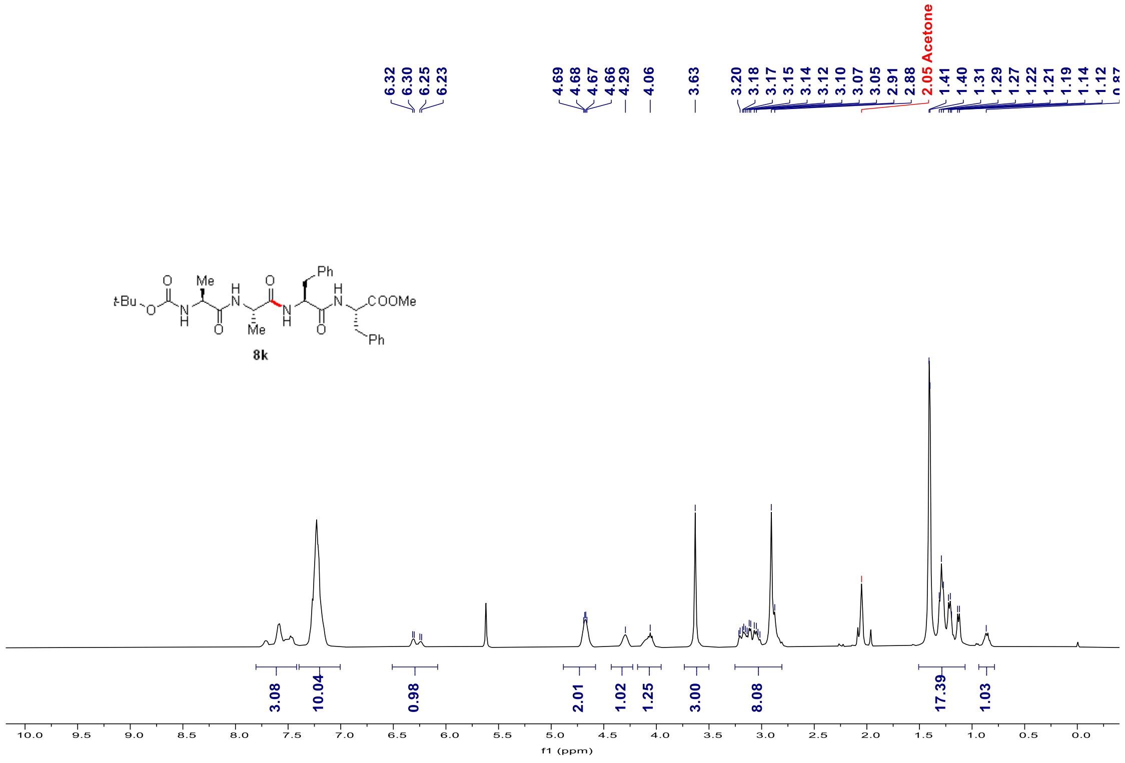

Figure S364. **^{1}H NMR** of the **8k** (400 MHz, Acetone-d_6)

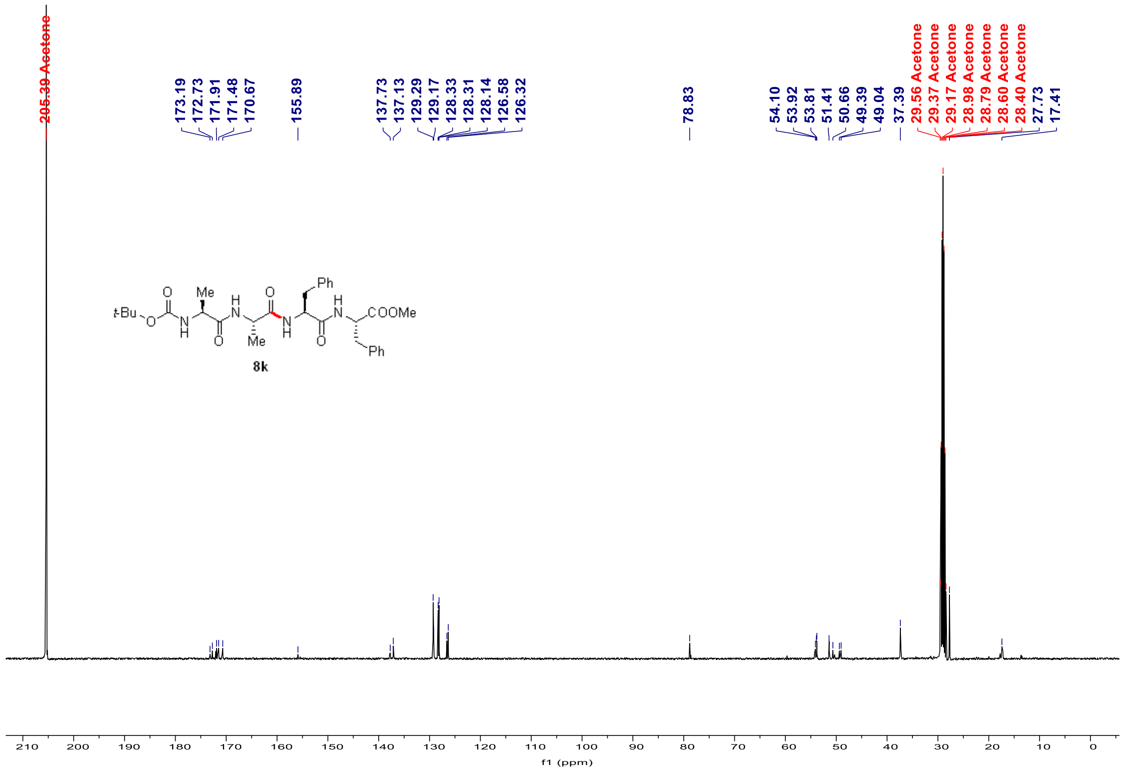

Figure S365. **^{13}C NMR** of the **8k** (101 MHz, Acetone-d_6)

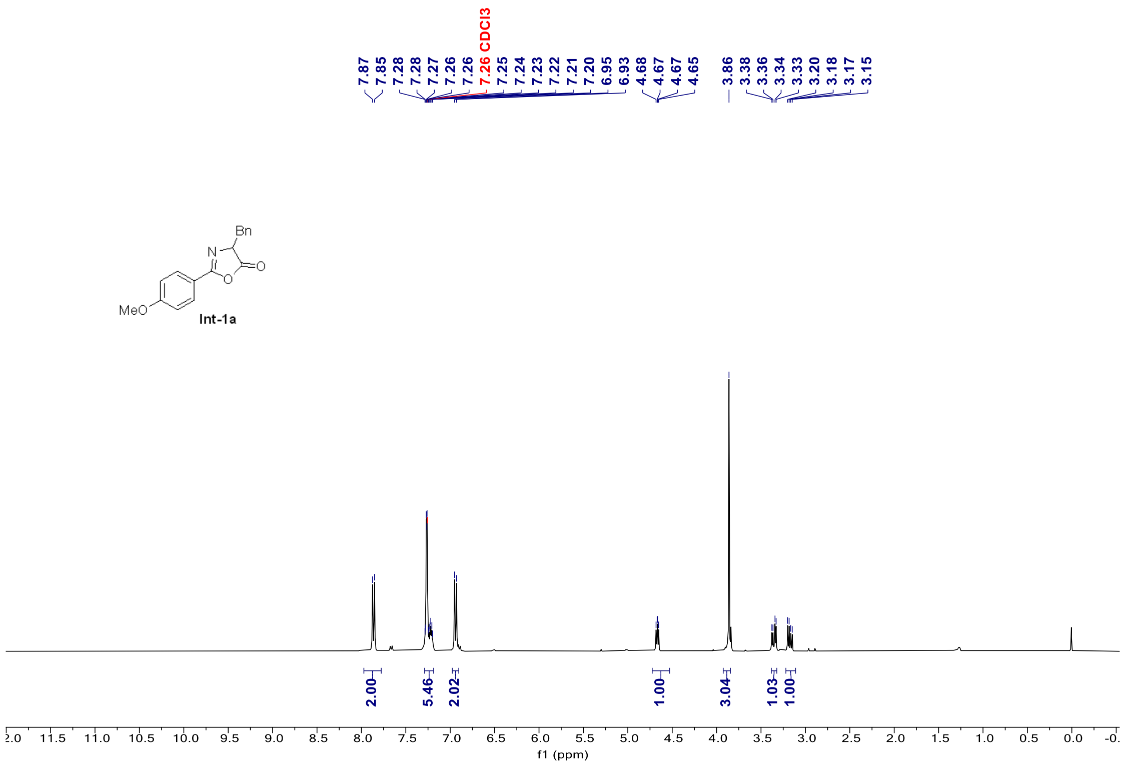

Figure S366. **^{1}H NMR** of the **Int-1a** (400 MHz, $CDCl_3$)

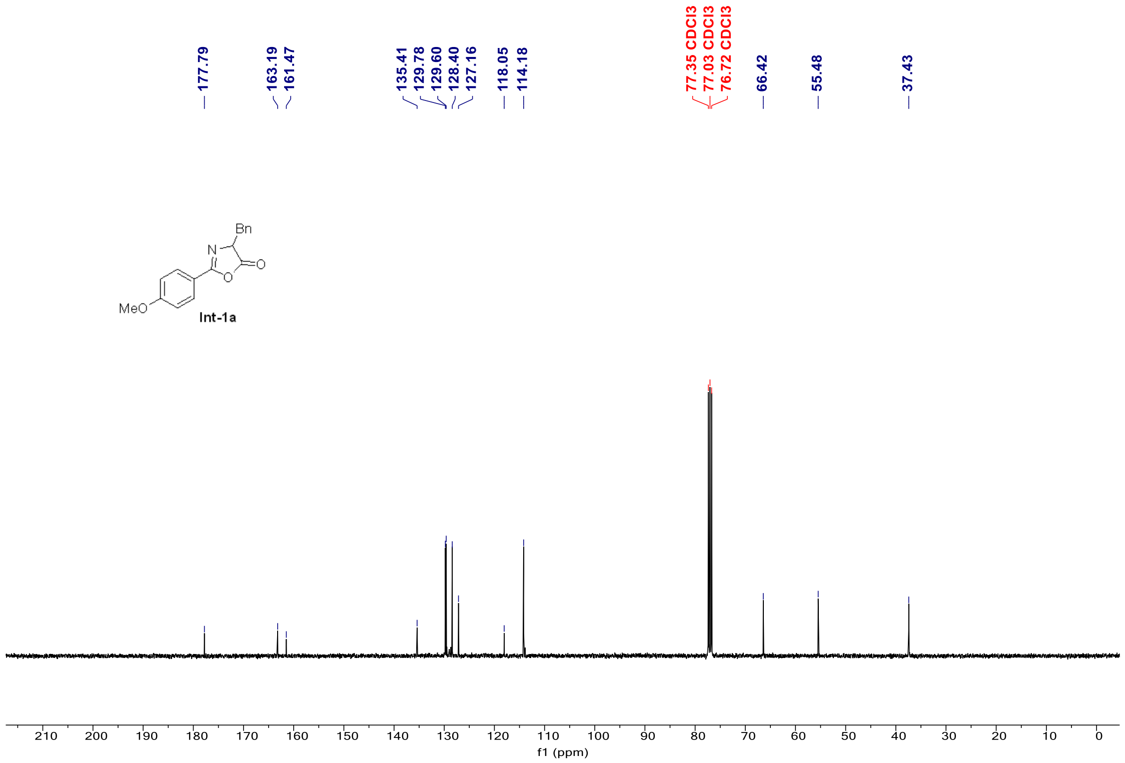

Figure S367. **^{13}C NMR** of the **Int-1a** (101 MHz, $CDCl_3$)

References

1. Oguri, T.; Kawai, N.; Shioiri, T.; Yamada, S. Amino Acids and Peptides. XXIX. A New Efficient Asymmetric Synthesis of α-Amino Acid Derivatives with Recycle of a Chiral Reagent-Asymmetric Alkylation of a Chiral Schiff Base from Glycine. *Chem. Pharm. Bull*. **26**, 803– 808 (1978).
2. Li, Z., Wang, B. Fan, S., Zhang, C. & Sun, J. Catalytic Enantioselective Nucleophilic Amination of α-Halo Carbonyl Compounds with Free Amines. *J. Am. Chem. Soc.* **147**, 576-584 (2025).
3. Canavelli, P., Islam, S. & Powner, M. W. Peptide ligation by chemoselective aminonitrile coupling in water. *Nature* **571**, 546–549 (2019).
4. Liang, J; Ruble, J. C., Fu, G. C. Dynamic Kinetic Resolutions Catalyzed by a Planar-Chiral Derivative of DMAP: Enantioselective Synthesis of Protected α-Amino Acids from Racemic Azlactones. *J. Org. Chem.* **63**, 3154–3155 (1998).
5. Xu, S., Jiang, D., Peng, Z., Hu, L., Liu, T., Zhao, L. & Zhao, J. Ynamide-Mediated Peptide Bond Formation: Mechanistic Study and Synthetic Applications. *Angew. Chem. Int. Ed.* **61**, e202212247 (2022).
6. Frisch, M. J.; Trucks, G. W.; Schlegel, H. B.; Scuseria, G. E.; Robb, M. A.; Cheeseman, J. R.; Scalmani, G.; Barone, V.; Petersson, G. A.; Nakatsuji, H.; Li, X.; Caricato, M.; Marenich, A. V.; Bloino, J.; Janesko, B. G.; Gomperts, R.; Mennucci, B.; Hratchian, H. P.; Ortiz, J. V.; Izmaylov, A. F.; Sonnenberg, J. L.; Williams, D. F.; Lipparini, F.; Egidi, F.; Goings, J.; Peng, B.; Petrone, A.; Henderson, T.; Ranasinghe, D.; Zakrzewski, V. G.; Gao, J.; Rega, N.; Zheng, G.; Liang, W,; Hada, M.; Ehara, M.; Toyota, K.; Fukuda, R.; Hasegawa, J.; Ishida, M.; Nakajima, T.; Honda, Y.; Kitao, O.; Nakai, H.; Vreven, T.; Throssell, K.; Montgomery Jr. J. A.; Peralta, J. E.; Ogliaro, F.; Bearpark, M. J.; Heyd, J. J.; Brothers, E. N.; Kudin, K. N.; Staroverov, V. N.; Keith, T. A.; Kobayashi, R.; Normand, J.; Raghavachari, K.; Rendell, A. P.; Burant, J. C.; Iyengar, S. S.; Tomasi, J.; Cossi, M.; Millam, J. M.; Klene, M.; Adamo, C.; Cammi, R.; Ochterski, J. W.; Martin, R. L.; Morokuma, K.; Farkas, O.; Foresman, J. B.; Fox, D. J., Gaussian 16 Rev. C.01, Wallingford, CT, 2016.
7. (a) Becke, A. D. A New Mixing of Hartree-Fock and Local Density-functional Theories. *J. Chem. Phys*. **98**, 1372 (1993). (b) Becke, A. D. Density-functional Thermochemistry. III. The Role of Exact Exchange. *J. Chem. Phys*. **98**, 5648 (1993). (c) Grimme, S.; Antony, J.; Ehrlich, S.; Krieg, H. A Consistent and

Accurate ab Initio Parametrization of Density Functional Dispersion Correction (DFT-D) for the 94 elements H-Pu. *J. Chem. Phys*. **132**, 154104 (2010).

8. Weigend, F.; Ahlrichs, R. Balanced Basis Sets of Split Valence, Triple Zeta Valence and Quadruple Zeta Valence Quality for H to Rn: Design and Assessment of Accuracy. *Phys. Chem. Chem. Phys.* **7**, 3297 (2005).
9. Marenich, A. V.; Cramer, C. J.; Truhlar. D. G. Universal Solvation Model Based on Solute Electron Density and on a Continuum Model of the Solvent Defined by the Bulk Dielectric Constant and Atomic Surface Tensions. *J. Phys. Chem. B*. **113**, 6378- 6396 (2009).
10. (a) Fukui, K. Formulation of the Reaction Coordinate, *J. Phys. Chem*., **74**, 4161-4163 (1970). (b) Fukui, K. The Path of Chemical Reactions - The IRC Approach, *Acc. Chem. Res.*, **14**, 363- 368 (2002).
11. Lu, T. & Chen, Q. Independent gradient model based on Hirshfeld partition: A new method for visual study of interactions in chemical systems. J. Comput. Chem. 2022, 43, 539–555.
12. Lu, T. & Chen, F. Multiwfn: a multifunctional wavefunction analyzer. J. Comput. Chem. 2012, 33, 580−592.
13. Humphrey, W., Dalke, A. & Schulten, K. VMD: visual molecular dynamics. J. Mol. Graph. 1996. 14, 33−38.
14. C. Y. Legault, CYLVIEW, 1.0b, Université de Sherbrooke, 2009; http://www.cylview.org
