# Supplementary material for: Stereoselective diversification of α-amino acids enabled by N-heterocyclic carbene catalysis
Source: Nat Commun. 2025 Oct 9;16:8991. doi: 10.1038/s41467-025-64024-7 (PMC12511568; doi:10.1038/s41467-025-64024-7)
Supplement: Supplementary file 2 — Description of Additional Supplementary Files [file 41467_2025_64024_MOESM2_ESM.pdf]

File Name: Supplementary Data 1
Description: X-ray CIF files of the compounds **4e** and **4al**

File Name: Supplementary Data 2
Description: Cartesian coordinates
